# Periplocymarin Alleviates Doxorubicin-Induced Heart Failure and Excessive Accumulation of Ceramides

**DOI:** 10.3389/fcvm.2021.732554

**Published:** 2021-11-19

**Authors:** Weijing Yun, Lei Qian, Ruqiang Yuan, Hu Xu

**Affiliations:** Advanced Institute for Medical Sciences, Dalian Medical University, Dalian, China

**Keywords:** periplocymarin, doxorubicin, heart failure, metabolomics, ceramide, apoptosis

## Abstract

Doxorubicin-driven cardiotoxicity could result in dilated cardiomyopathy and heart failure (HF). Previously, we showed that periplocymarin exerted a cardiotonic role by promoting calcium influx and attenuating myocardial fibrosis induced by isoproterenol (ISO) by improving the metabolism of cardiomyocytes. However, the impact of periplocymarin on doxorubicin (DOX)-triggered cardiomyopathy has not been investigated. In the current study, C57BL/6 mice were randomly divided into three groups, namely, the control, DOX, and DOX+periplocymarin groups. The cardiac function and apoptosis were measured. Our results revealed that periplocymarin administration greatly improved the DOX-induced cardiac dysfunction manifested by the ejection fraction (EF%), fractional shortening (FS%), left ventricular posterior wall thickness (LVPW), left ventricular anterior wall thickness (LVAW), left ventricular (LV) mass, and attenuated DOX-induced cardiomyocyte apoptosis assessed by hematoxylin and eosin (H&E) staining, terminal deoxynucleotidyl transferase dUTP nick end labeling (TUNEL) staining, and western blotting. Further study using H9c2 cells revealed that the pretreatment of periplocymarin suppressed DOX-induced apoptosis evidenced by annexin V staining. Moreover, liquid chromatography with tandem mass spectrometry (LC-MS/MS) analysis demonstrated that DOX lead to an accumulation in serum ceramide, and the pre-treatment of periplocymarin could reverse this phenomenon. Network pharmacology also demonstrated that ceramide metabolism was involved in the process. Consistently, real-time PCR showed that periplocymarin significantly abolished the induction of the genes involved in the *de novo* synthesis of ceramide, i.e., *CerS2, CerS4, CerS5*, and *CerS6*, and the induction was attributed to the treatment of DOX. Collectively, these results suggested that periplocymarin reduced cardiomyocyte apoptosis to protect hearts from DOX-induced cardiotoxicity and the *de novo* synthesis of ceramides was involved in this process.

## Introduction

Doxorubicin (DOX) has been approved for the treatment of diverse cancers involving leukemia, neuroblastoma, and breast cancer (1). Unfortunately, despite the effective antineoplastic feature of DOX, it also exhibits side effects. It has been reported that the dose-dependent cardiotoxicity of DOX often results in cardiac systolic dysfunction and ultimately leads to heart failure (HF). This potential risk greatly restricted the clinical application of DOX. Therefore, identifying novel therapeutic drugs to block or reverse DOX-induced cardiotoxicity are urgently needed to be developed (1–3).

Accumulating evidence has elucidated the underlying mechanism involved in the pathogenesis of DOX-induced cardiotoxicity (4). After birth, cardiomyocytes no longer enter the cell cycle and eventually differentiate into a mature state, hence their survival is crucial. Besides, increasing cardiomyocyte apoptosis will contribute to contractile tissue loss, decompensated hypertrophy, irreversible fibrosis, and eventually leading to HF (5). Accordingly, developing effective therapeutic drugs or targets that are capable of combatting cardiomyocytes apoptosis triggered by DOX is greatly warranted.

With the development of modern pharmacology, emerging evidence proved that Chinese medicine could enhance cardiac function (6), including Ginsenoside Rg1 (7), Astragaloside IV (8), and Tanshinone IIA (9). In addition, many traditional Chinese materia medica preparations, such as Qiliqiangxin capsules and Qishenyiqi dripping pills, can also enhance myocardial contractility and improve cardiac dysfunction, which have been widely applied in clinics (10, 11). Periplocymarin is a pharmacodynamic substance in Qiliqiangxin capsules, which is extracted from Periplocae Cortex (12). Periplocymarin shares the basic molecular structure of cardiac glucoside. Studies have demonstrated that periplocymarin has a strong anticancer activity characterized by the inhibition of tumor cell proliferation and the promotion of cell apoptosis (13, 14). We previously reported that periplocymarin improves cardiac function by elevating the cardiomyocyte calcium ion (Ca^2+^) concentration (15). Additionally, periplocymarin prevents isoproterenol (ISO)-induced cardiac fibrosis *via* improving the cardiomyocyte metabolism by targeting endothelial nitric oxide synthase (eNOS) and cyclooxygenase-2 (COX-2) (16). However, the role of periplocymarin on DOX-induced HF is largely unknown.

Ceramides are bioactive membrane lipids regulating signal transduction pathways that contain a basic skeleton of sphingosine which links to fatty acyl chains of different lengths (17). Ceramide accumulation is strongly associated with the pathogenesis of many diseases, such as diabetes, cardiomyopathy, and atherosclerosis (18). Serum ceramide levels have been shown to be accurate biomarkers of undesirable cardiovascular events in humans (19, 20). The study showed associations of higher plasma levels of N-palmitoyl-sphingosine (Cer16:0) and N-palmitoyl-sphingosyl-phosphorylcholine (SM16:0) with increased risk of HF and higher levels of N-behenoyl-sphingosine (Cer22:0), N-arachidoyl-sphingosyl-phosphorylcholine (SM20:0), N-behenoyl-sphingosyl-phosphorylcholine (SM22:0), and N-lignoceroyl-sphingosyl-phosphorylcholine (SM24:0) with decreased risk of HF (21). Previous investigations revealed that ceramides participate in the regulation of cardiac contractility and the process of cardiomyocyte apoptosis (22–24).

In the present study, we demonstrated that the administration of periplocymarin improved DOX-induced HF in mice, and we uncovered that periplocymarin ameliorated cardiomyocytes apoptosis and suppressed ceramides production induced by DOX both *in vivo* and *in vitro*.

## Materials and Methods

### Chemicals and Drugs

Periplocymarin was purchased from Sigma-Aldrich (St. Louis, Missouri, United States). Doxorubicin was purchased from MedChemExpress (New Jersey, United States). The purities of compounds were more than 98% determined by HPLC. Bcl-2, caspase-3, and cleaved caspase-3 antibodies were purchased from Cell Signaling Technology (Danvers, Massachusetts, United States). eIF5 antibody was purchased from Santa Cruz.

### Animals and Animal Models With HF

Adult (8–10 weeks) male C57BL/6 mice were used in the study. The mice were bred at the Animal Center of Dalian Medical University with a temperature of 23°C and 12-h light/dark cycles. The animals reached a fresh diet and sterile water freely. The animal protocol was approved by the Animal Care and Use Review Committee of Dalian Medical University and the procedure was in accordance with the Guide for the Care and Use of Laboratory Animals (National Institutes of Health). The mice were randomly divided into three groups, i.e., the control group (n = 5), DOX group (n = 7), and DOX plus periplocymarin group (n = 6). Firstly, the DOX plus periplocymarin group was injected with periplocymarin at a dose of 5 mg/kg for 3 days. Then, the DOX plus periplocymarin and DOX groups were abdominally injected with DOX (20 mg/kg) once to build the mouse model of HF (25). After that, the DOX plus periplocymarin group was injected with periplocymarin for 3 days. The control and DOX groups were injected with saline for 3 days.

### Cell Culture

#### Isolation and Culture of Cardiomyocyte

Cardiomyocytes were isolated and cultured from neonatal Sprague-Dawley (SD) rats as described previously (26). In brief, neonatal SD rat pups (within 2 days) were euthanized and the hearts were digested with 0.1% collagenase I. The cardiomyocytes were obtained by differential adhesion method: the digested cells were plated on 10 cm dishes for 3–4 h; the unattached cells were cardiomyocytes and they were moved to a new dish. The cardiomyocytes were cultured for 48 h in a Dulbecco's Modified Eagle Medium (DMEM) medium containing 10% fasting blood sugar (FBS). Then the cardiomyocytes were pretreated with 0.1 μM of periplocymarin for 24 h and then treated with 5 μM of DOX for 12 h in DMEM containing 1% FBS.

#### H9c2 Cell Line Culture

H9c2 cell line (rat embryonic ventricular myocyte) was purchased from the Shanghai Institute of Biochemistry and Cell Biology (China). The H9c2 cells were cultured in a DMEM medium containing 10% FBS at the condition of 37°C and 5% calcium dioxide (CO_2_) and the cells were passaged at 70–80% confluence (27). For the experiment, the H9c2 cells were pretreated with 0.1 μM of periplocymarin for 24 h and then treated with 5 μM of DOX for 12 h in DMEM containing 1% FBS.

### Flow Cytometry Analysis

Cell apoptosis was detected using an Annexin V-FITC apoptosis kit and following the provided procedure (Beyotime, Shanghai, China). After pretreatment with periplocymarin for 24 h, the cells were treated with 5 μM of DOX for 12 h. Then they were harvested using 0.25% trypsin and washed twice with cold phosphate-buffered saline (PBS). The harvested cells were centrifuged at 2,000 rpm/min for 5 min and the cells were resuspended with 1 × binding buffer to a density of 1 × 10^5^ cells/ml. Afterward, 100 μl of each sample was stained with an Annexin V-FITC staining solution in a 1.5 ml Eppendorf tube at room temperature and avoided light for 15 min. Then, 400 μl of 1 × binding buffer was added into the tubes. The apoptotic cell population was then quantified using a flow cytometer (BD FACSVerse, Becton, Dickinson and Company, United States) and Cell Quest Research Software (FlowJo_V10, Becton, Dickinson and Company, United States).

### Echocardiography

Echocardiography was performed using the Vevo 3,100 high-resolution imaging system (Fujifilm Visual Sonics Inc., Minato City, Tokyo, Japan) to analyze the cardiac function of the animals. The mice were anesthetized using isoflurane (1%) and the heart rate was controlled at 500 ± 30 b.p.m. The percentage of ejection fraction (EF%), fractional shortening (FS%), left ventricular posterior diastolic wall thickness (LVPW; d), left ventricular posterior systolic wall thickness (LVPW; s), left ventricular anterior diastolic wall thickness (LVAW; d), and left ventricular anterior systolic wall thickness (LVAW; s) were measured or calculated as previously described (15).

### Hematoxylin and Eosin (H&E) and Terminal Deoxynucleotidyl Transferase dUTP Nick end Labeling (TUNEL) Staining

The mice were euthanized with overdose CO_2_ inhalation and perfused with PBS through the heart, then the heart tissues were removed into 4% formalin. The hearts were embedded in paraffin and cut into 5 μm sections. Hematoxylin and eosin staining and TUNEL assay were performed according to the standard procedures.

### Real-Time PCR

The cells or heart tissues were lysed in TRIZOL reagent (Invitrogen, United States) and the total RNA was extracted. Then the RNA samples were quantified by NanoDrop 2000 (Thermo Fisher Scientific, United States) and were reverse transcribed to complementary DNA (cDNA). Real-time PCR primers were designed on the National Center for Biotechnology Information (NCBI) website using known sequences, and the primer pairs were listed in Supplementary Table 1. The internal standard control used was 18S. A mix of nucleoside triphosphates (NTP) and SYBR Green was used and the real-time PCR was performed on the LightCyler96 Sequence Detection System (Roche, Basel, Switzerland). The reaction condition was 94°C for 5 min, followed by 35 cycles of 94°C for 30 s, 60°C for 30 s, 72°C for 30 s, then extension at 72°C for 5 min (28).

### Western Blot

For western blot, the method was followed as previously reported (28). Briefly, the heart tissues or cells were lysed in a radioimmunoprecipitation assay (RIPA) buffer containing the protease and phosphatase inhibitor cocktail. After centrifugation at 12,000 g for 15 min at 4°C, the supernatant containing the total protein was quantified by a bicinchoninic acid (BCA) Assay Kit, the lysates were mixed with 5 × sodium dodecyl sulfate (SDS) loading buffer. After fractionated with 10% sodium dodecyl sulfate-polyacrylamide gel electrophoresis (SDS-PAGE), the proteins were transferred onto a nitrocellulose filter membrane. The primary antibodies were incubated overnight at 4°C and followed by incubation with an horseradish peroxidase (HRP)-labeled secondary antibody for 1 h at room temperature. The bands on the membranes were visualized after enhanced chemiluminescence (ECL) incubation using a Chemiluminescent Imaging System (Tanon 5200, China). Image J software (NIH) was used to analyze the intensities of the bands.

### Metabolomics Analysis

#### Metabolites Extraction

Serum samples (100 μl) were mixed with 300 μl of cooled methanol, vortexed for 60 s, then 600 μl of chloroform was added, and the solution was vortexed for 2 min and incubated for about 1 h at room temperature. The mix solution was centrifuged at 2,600 g for 10 min, and that chloroform phase was removed and dried under vacuum. Before analysis, the extracted sample was dissolved in 100 μl of chloroform/methanol (2:1, V/V) and then diluted two-fold with methanol.

Afterward, 300 μl cold MeOH was added to the cell samples, then, the cells were sucked out by a pipette. Two volumes of chloroform were added to each sample, and the solution was vortexed for 2 min and incubated for about 1 h at room temperature. After that, the process was the same as serum.

All samples of equal quantity were pooled together to generate quality control samples (QC) and inserted into the analysis batch every six samples to ensure consistent system performance and good metabolic profile quality.

#### Liquid Chromatography With Tandem Mass Spectrometry (LC-MS/MS) Analysis of Ceramides

All the experiments were performed on a Qtrap 5,500 mass spectrometer (SCIEX, United States) with electrospray ionization. Multiple reaction monitoring detection was used for the detection of metabolites. An acquity ultra performance liquid chromatography ethylene bridged hybrid (UPLC BEH) Amide Column (2.1 × 100 mm, 1.7 m) was employed for metabolites separation at 40°C. Mobile phase A was 10 mM ammonium acetate in water with 0.1% formic acid, and mobile phase B was 0.1% formic acid in acetonitrile. The gradient was from 95 to 80% B over 5 min, 80 to 60% B over 9 min, held for 3 min, then the column was returned to its starting condition. The flow rate was 0.2 ml/min and the injection volume was 2 μl. The collection was carried out in positive ion mode (Supplementary Table 2 for 31 metabolites).

#### Data Processing and Pathway Analysis

The retention time (RT), peak area, peak width, peak width at 50% height, and signal to noise ratio (S/N) were generated in MultiQuant 3.0 (SCIEX, United States). The software (Version 14.1, Umetrics, Umea, Sweden) was used to perform pattern recognition analysis on all peaks detected in each data file, including partial least squares discriminant analysis (PLS-DA) and orthogonal partial least squares discriminate analysis (OPLS-DA). In the OPLS-DA model, metabolites with variable importance in projection (VIP) > 1 were used as potential biomarkers. The database of MetaboAnalyst 4.0 (http://www.metaboanalyst.ca/) was used to predict the relevant pathway of potential biomarkers.

### Network Pharmacology Study and Gene Expression Omnibus (GEO) Database Validation

The potential targets of periplocymarin were gathered from the database Bioinformatics Analysis Tool for Molecular mechANism of Traditional Chinese Medicine (BATMAM-TCM) (http://bionet.ncpsb.org.cn/batman-tcm/). The HF-related genes were derived from the GeneCard database (https://www.GeneCards.org/). Then through jvenn (http://jvenn.toulouse.inra.fr/app/example.html), the intersections with periplocymarin and HF were analyzed. Potential periplocymarin targets and HF-related genes were imported into Cytoscape software (National Institute of General Medical Sciences (NIGMS), United States) to construct the network. The protein-protein interaction (PPI) networks of 246 intersecting genes were created by using the Search Tool for the Retrieval of Interacting Genes/Proteins (STRING) database (https://string-db.Org/) with a medium confidence level (interaction score > 0.40) and the Cytoscape software. The Gene Ontology (GO) and Kyoto Encyclopedia of Genes and Genomes (KEGG) pathway enrichment analyses of the intersecting genes were carried out using the database Metascape (https://metascape.org/gp/index.html#/main/step1). The RNA-sequencing data (GSE157282) was obtained from the GEO database and the gene expression (FPKM) of the control and DOX treated group was analyzed.

### Statistics

The experimental data were analyzed and plotted using GraphPad Prism 8.3.0 software (GraphPad Software Inc., San Diego, California, United States). The results are presented as the mean ± SEM. Student's *t*-test was used for two-group comparison, and ANOVA was used for multi-group comparison. *p* < 0.05 was considered significant.

## Results

### Periplocymarin Prevented the HF Induced by DOX

**Figure 1:**
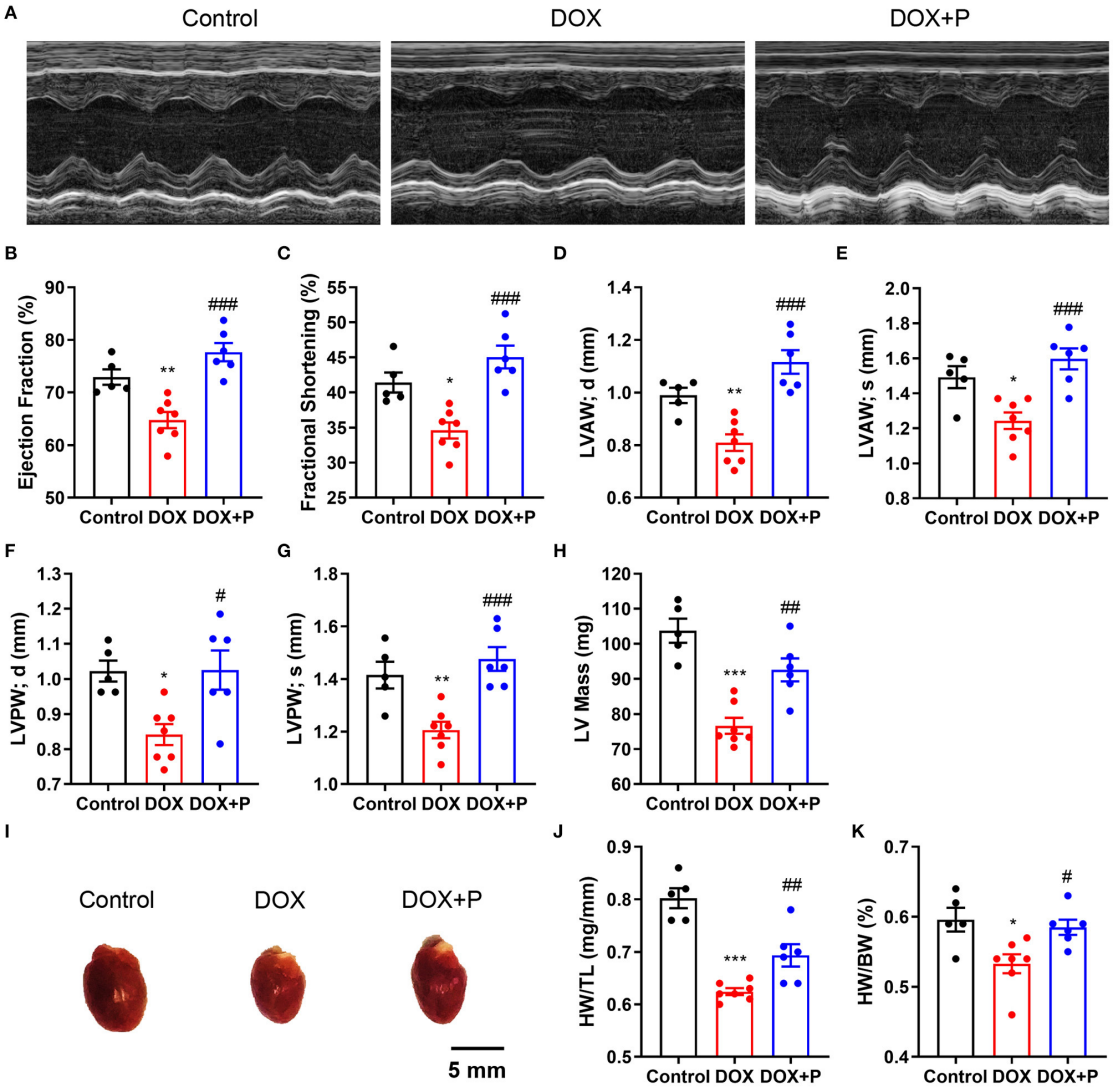
Effects of periplocymarin on cardiac function and heart weight after 3-day doxorubicin (DOX) treatment in mice. The cardiac function of the mice was determined by the echocardiography in M-mode on the aspect of the short axis. **(A)** Representative images of echocardiography. **(B–H)** The ejection fraction (EF%) **(B)**, fractional shortening (FS%) **(C)**, left ventricular anterior diastolic wall thickness (LVAW; d) **(D)**, left ventricular anterior systolic wall thickness (LVAW; s) **(E)**, left ventricular posterior diastolic wall thickness (LVPW; d) **(F)**, left ventricular posterior systolic wall thickness (LVPW; s) **(G)** and left ventricular mass **(H)** were calculated according to the M-mode echocardiography. Data are presented as mean ± SEM; *n* = 5–7, *p < 0.05, ^**^p < 0.01, ***p < 0.001 vs. control; ^#^*p* < 0.05, ^##^*p* < 0.01, ^###^*p* < 0.001 vs. DOX. **(I)** The mice were euthanized and the representative images of the hearts were shown. Scale bar, 5 mm. **(J)** The ratios of heart weights to tibia length were calculated. Data are presented as mean ± SEM; *n* = 5–7. ***p < 0.001 vs. control, ^##^*p* < 0.01 vs. DOX. **(K)** The ratios of heart weights to body weights were calculated. Data are presented as mean ± SEM; *n* = 5-7. *p < 0.05 vs. control, ^#^*p* < 0.05 vs. DOX.

To investigate the effect of periplocymarin on DOX-induced heart failure, we initially performed echocardiography to evaluate the cardiac function in three groups of mice (Figure 1A). As expected, both EF% and FS% in the DOX group were reduced compared with the vehicle. Intriguingly, periplocymarin treatment successfully rescued the decrease in EF% and FS% triggered by DOX (Figures 1B,C). Further analysis revealed that DOX treatment led to the thinning of the left ventricular wall characterized by reduced LVAW and LVPW in both the diastole and systole. To our surprise, the levels of LVAW and LVPW were markedly reversed by periplocymarin (Figures 1D–G). Correspondingly, the LV mass was significantly decreased in the DOX group, while this decrease was largely prevented by periplocymarin (Figure 1H). Moreover, the mice hearts were harvested, and the heart-to-body weight (HW/BW) ratio and heart weight-to-tibia length (HW/TL) ratio were also assessed. Consistent with the echocardiography results, the ratios of HW/BW and HW/TL were reduced after the DOX treatment, which were obviously reversed by the periplocymarin treatment (Figures 1I–K). Altogether, these data indicated that periplocymarin exerted an efficaciously protective effect on DOX-induced HF in mice.

### Periplocymarin Mitigated DOX-Induced Cardiomyocyte Apoptosis

**Figure 2:**
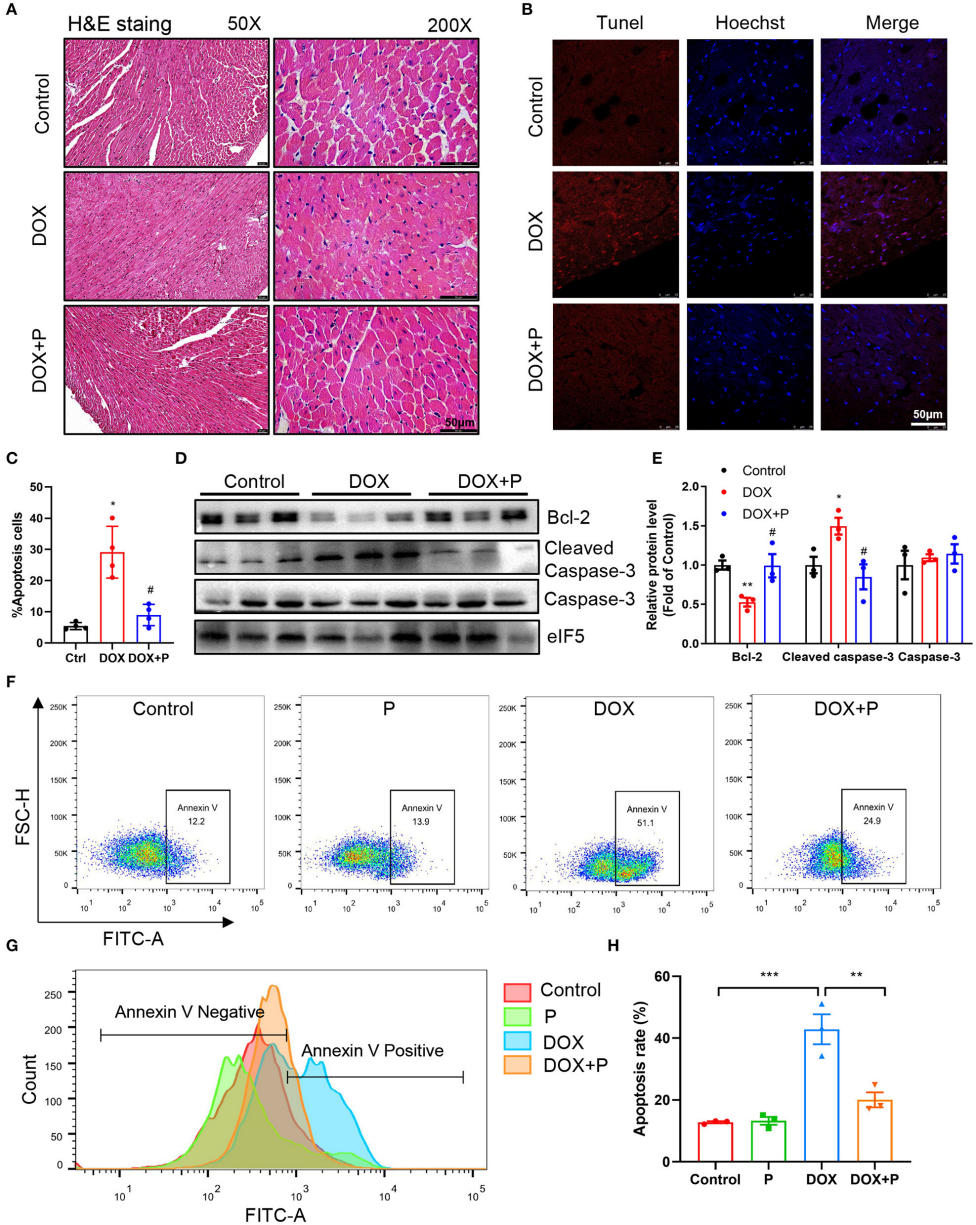
Periplocymarin improved cardiomyocyte apoptosis induced by DOX. **(A)** Representative micrographs of hematoxylin and eosin (H&E) staining. The left panel is 50X (Scale bar, 50 μm), the right panel is 200X (Scale bar, 50 μm). **(B,C)** Representative fluorescent images of terminal deoxynucleotidyl transferase dUTP nick end labeling (TUNEL) staining of the hearts **(B)**. Scale bar, 50 μm. Quantitative analysis of TUNEL staining in different groups **(C)**. Data are presented as mean ± SEM; *n* = 4, *p < 0.05 vs. control, #*p* < 0.05 vs. DOX. **(D)** Western blot analysis of Bcl-2, cleaved caspase-3, and caspase-3 protein levels in heart tissues. **(E)** Quantitative analysis of Bcl-2, cleaved caspase-3, and caspase-3 expression in **D**. Data are presented as mean ± SEM; *n* = 3, *p < 0.05 vs. control, **p < 0.001 vs. control, #*p* < 0.05 vs. DOX. **(F)** Flow cytometric scatterplot of Annexin-V-FLTC staining of H9c2 cell apoptosis. **(G)** Flow cytometric histogram of Annexin-V-FLTC staining of H9c2 cell apoptosis. **(H)** Quantitative analysis of apoptotic H9c2 cells in different groups using bar graphs. Data are presented as mean ± SEM; *n* = 3. **p < 0.01, ***p < 0.001.

To further investigate the cardiac protective effect of periplocymarin, H&E staining was performed and deranged cellular structures, edematous cardiomyocytes, and dissolved myofibers were observed in DOX-treated hearts. However, these abnormalities were notably ameliorated by periplocymarin (Figure 2A). To provide sufficient evidence to exhibit the damage and repair of cardiomyocytes, TUNEL staining was applied to examine the DOX-induced cytotoxicity and display the protective effect of periplocymarin on cardiomyocyte apoptosis. Indeed, TUNEL positive cardiomyocytes were markedly increased in the DOX-treated hearts, suggesting massive apoptosis after the DOX treatment. Oppositely, when co-treated with periplocymarin, this undesirable phenomenon was obviously rescued (Figures 2B,C). Further analysis of apoptotic proteins using western blot showed that the expression of Bcl-2 was significantly decreased in the DOX group, at the same time, the expression of cleaved caspase 3 was significantly upregulated in the DOX group, this phenomenon was reversed by the administration of periplocymarin (Figures 2D,E). Furthermore, *in vitro* study using H9c2 cells was performed to evaluate the cytotoxicity of periplocymarin and to confirm the antiapoptotic role of periplocymarin in DOX-treated cardiomyocytes. The Cell Counting Kit-8 (CCK-8) assay exhibited that periplocymarin had no cytotoxicity when the concentration was lower than 1 μM, and the IC_50_ was 84.74 μM (Supplementary Figure 1). According to the annexin V staining, periplocymarin treatment alone did not affect cell apoptosis. Doxorubicin alone caused the significant apoptosis of H9c2 by ~50% in comparison to the control. Importantly, treatment of the cells with periplocymarin dramatically counteracted the DOX-mediated apoptosis (Figures 2F–H). However, periplocymarin had an optimal antiapoptotic effect at the concentration of 0.1 μM (Supplementary Figure 2). Besides, DOX induced a massive accumulation of reactive oxygen species (ROS) in H9c2 cells, while periplocymarin significantly suppressed ROS production (Supplementary Figure 3). Overall, co-treatment with periplocymarin improved DOX-induced cardiac injury, especially cardiomyocytes apoptosis.

### Periplocymarin Regulated Ceramides Metabolism Disorder Induced by DOX

Recently, the role of ceramides in HF has been extensively investigated. Serum ceramides contents have been shown to be strongly associated with adverse cardiovascular disease outcomes (20). Accordingly, we tested serum ceramide levels in three groups of mice. Liquid chromatography-MS/MS was used to detect the serum ceramide content in the control, DOX, and DOX plus periplocymarin group. The relative standard deviation (RSD) of the peak areas of ceramides was 4.66–13.48% (Supplementary Table 3), which indicated that the method had qualified stability and repeatability.

**Figure 3:**
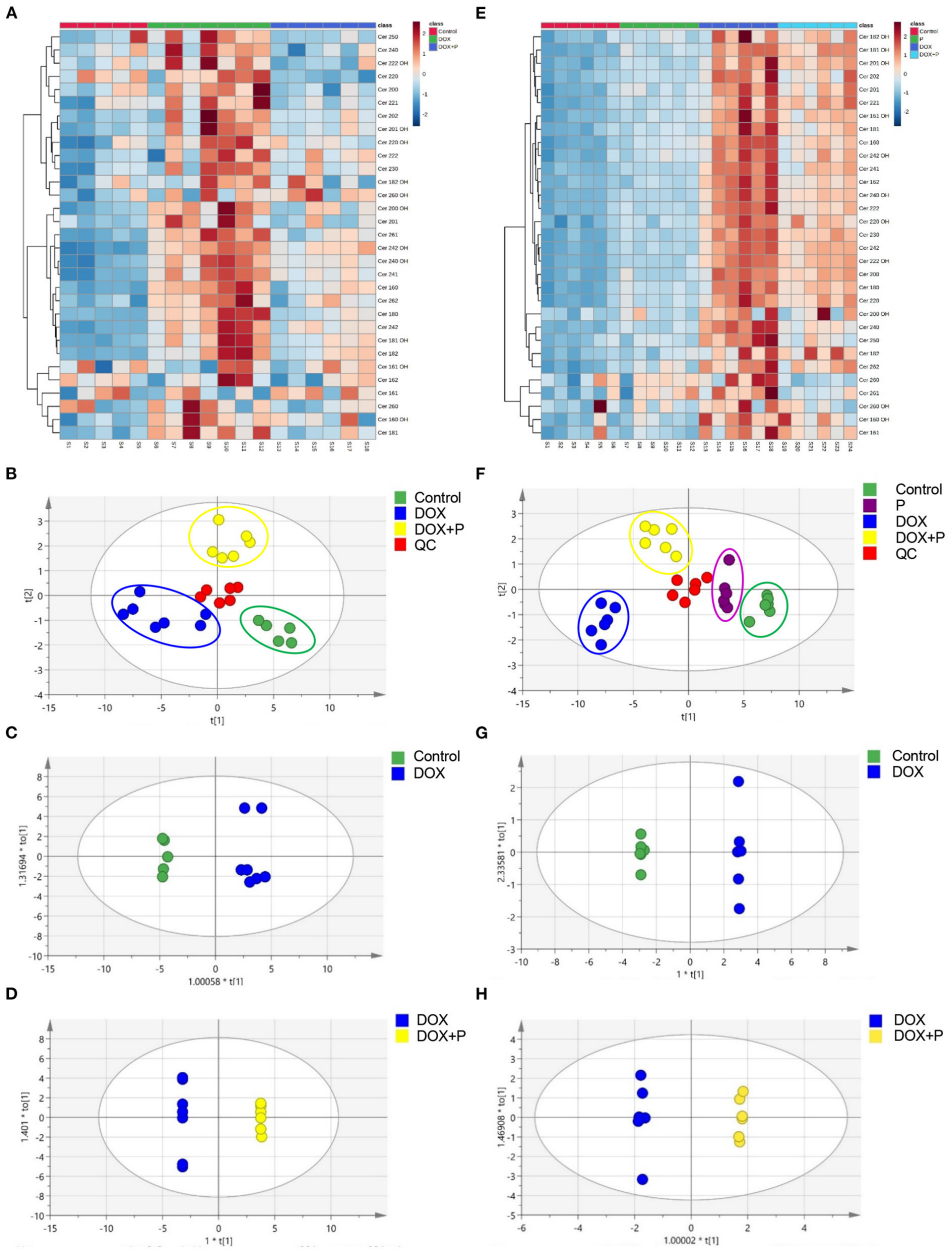
Periplocymarin regulated metabolic disorders induced by DOX. **(A–D)**. The ceramides in mice serum were measured by liquid chromatography with tandem mass spectrometry (LC-MS/MS). The cluster heatmap of ceramides was presented **(A)**. The score scatter plots of the quality control (QC) and the mice serum partial least squares discriminant analysis (PLS-DA) data were presented **(B)**. Paired comparison of orthogonal partial least squares discriminate analysis (OPLS-DA) score scatter plots between the control group and DOX group **(C)**. Paired comparisons of OPLS-DA score scatter plots between the DOX group and DOX + periplocymarin group **(D)**. **(E**–**H)** Ceramides in H9c2 cells were measured by LC-MS/MS. The cluster heatmap of ceramides in H9c2 cell **(E)**. The score scatter plots of the quality control (QC) and the H9c2 cells PLS-DA data were displayed **(F)**. Paired comparisons of OPLS-DA score scatter plots between the control and DOX groups **(G)**. Paired comparisons of OPLS-DA score scatter plots between the DOX and DOX + periplocymarin groups **(H)**. The dots of different colors represented different groups, the green dots represented control samples, the purple dots represented the periplocymarin group, the blue dots represented the six samples come from the DOX group, the yellow dots represented the six samples of DOX + periplocymarin group and the red dots represented QC samples.

The heatmap analysis was applied to visualize these potential biomarkers, with changes in color from deep red to navy blue indicating high to low relative levels of metabolites (Figures 3A,E). The PLS-DA method was used to analyze data. As shown in Figure 3B, a distinct aggregation state was observed among QC samples in PLS-DA, indicating that the experimental conditions were in a stable state. In addition, the sample points of different groups showed obvious separation. The results showed that there were significant differences among the three groups.

The OPLS-DA method was used to find identification ions which were helpful for the classification between groups. The score map of OPLS-DA showed significant separation between the control group and the DOX treated group (Figure 3C). The data (Supplementary Table 4) showed performance statistics of R^2^X = 0.898, R^2^Y = 0.902, and predictive parameter Q^2^Y = 0.836, suggesting that the models were valid and highly predictive. Metabolites with a VIP >1 in the OPLS-DA model could be selected as potential biomarkers. According to that, six metabolites were differentially expressed between the control and DOX group (Supplementary Table 5). A new OPLS-DA model was created to find the distinguishing metabolites between the DOX and the DOX+P group (Figure 3D). The prediction and sufficient reliability in this model were enough (Supplementary Table 4). According to the VIP values, 15 potential biomarkers were selected from the DOX group and DOX+P group (Supplementary Table 6).

To investigate whether periplocymarin can regulate ceramide metabolism *in vitro*, H9c2 cells were employed to assess the regulation of periplocymarin on ceramides metabolism. The stability and repeatability of the method were qualified with the RSD of the relative peak areas of ceramides were 3.37–13.07% (Supplementary Table 7).

Firstly, the PLS-DA method was used to analyze differences between the four groups. As shown in Figure 3F, the QC samples showed an aggregation state in PLS-DA, indicating that the experimental conditions were in a stable state. More importantly, the dots with different colors showed obvious separation, suggesting that there were significant differences between the four groups.

Then the OPLS-DA was used to analyze the metabolites and relationships among the different groups. As shown in Figure 3G, there was a distinct separation between the control and DOX groups. Another OPLS-DA model showed that metabolites in the DOX group and DOX+P group also had obvious differences (Figure 3H). According to the VIP-values (Supplementary Table 9), six potential biomarkers were selected from the control and DOX groups, and five potential biomarkers were selected from the DOX group and DOX+P group (Supplementary Table 10). In addition, the OPLS-DA model parameter is shown in Supplementary Table 8.

### Periplocymarin Attenuated the Increase of Ceramide-Induced by DOX *in vivo* and *in vitro*

**Figure 4:**
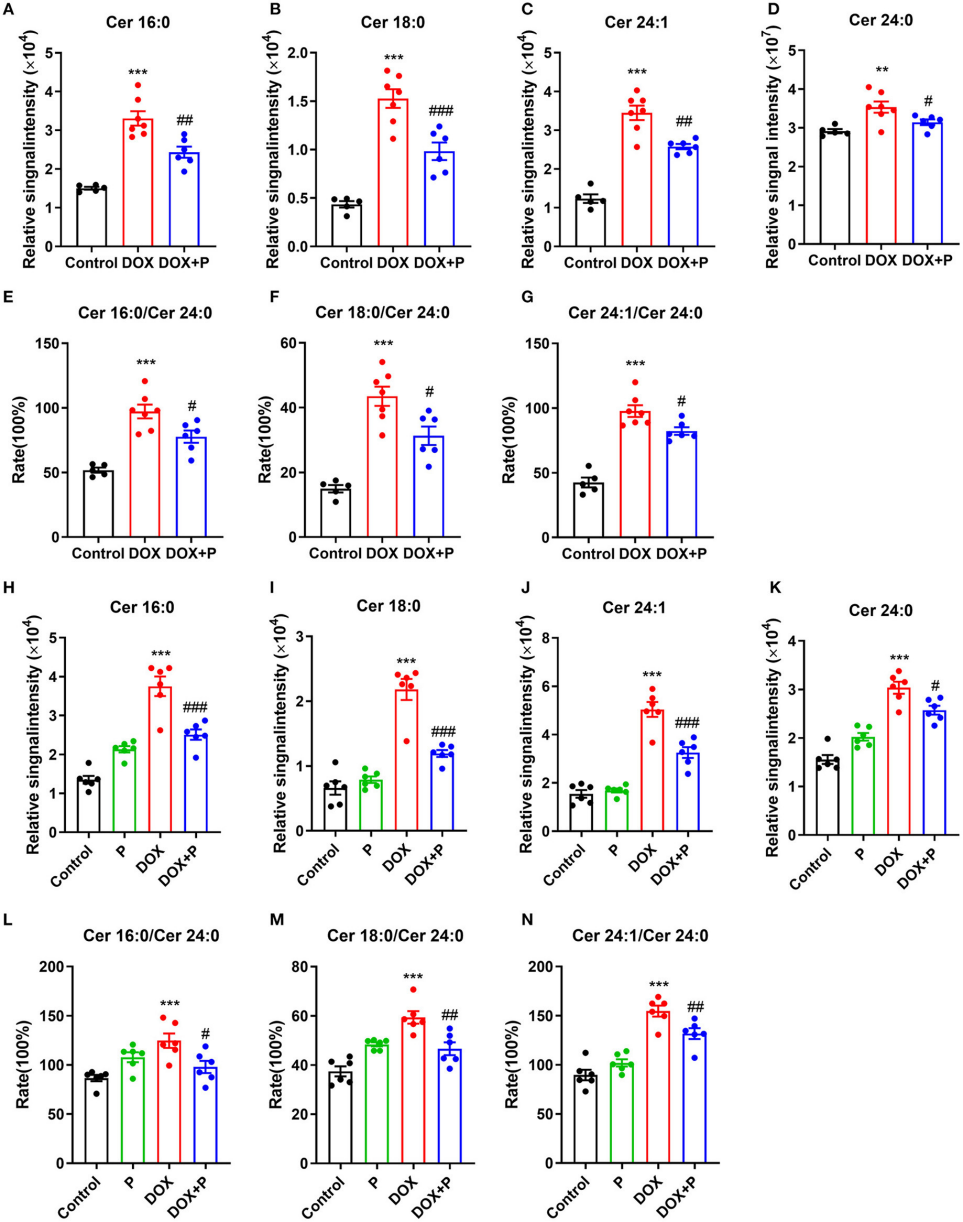
Periplocymarin regulated ceramides metabolism disorder induced by DOX. **(A–D)** Relative abundance of Cer16:0 **(A)**, Cer18:0 **(B)**, Cer24:1 **(C)**, and Cer24:0 **(D)** ceramides in mice serum. **(E–G)** The ratios of Cer16:0 to Cer24:0 **(E)**, Cer18:0 to Cer24:0 **(F)**, and Cer24:1 to Cer24:0 **(G)** in mice serum. Data are presented as mean ± SEM; *n* = 6, **p < 0.01, ***p < 0.001 vs. control; ^#^*p* < 0.05, ^##^*p* < 0.01, ^###^*p* < 0.001 vs. DOX. **(H–K)** Relative abundance of Cer16:0 **(H)**, Cer18:0 **(I)**, Cer24:1 **(J)**, and Cer24:0 **(K)** ceramides in H9c2 cells. **(L–N)** The ratios of Cer16:0 to Cer24:0 **(L)**, Cer18:0 to Cer24:0 **(M)** and Cer24:1 to Cer24:0 **(N)** in H9c2 cells. Data are presented as mean ± SEM; *n* = 6, ***p < 0.001 vs. control; ^#^*p* < 0.05, ^##^*p* < 0.01, ^###^*p* < 0.001 vs. DOX.

Based on the above analysis, we obtained three key metabolites that appeared in four analyses (Supplementary Tables 5, 6, 9, 10). To further verify the regulation of the periplocymarin on these three metabolites, we analyzed the relative peak areas of these three metabolites *in vivo* and *in vitro*. Certain ceramide species, such as Cer16:0 (N-palmitoyl-sphingosine), Cer18:0 (N-stearoylsphingosine), Cer24:1 (N-nervonoyl-sphingosine), and Cer24:0 (N-lignoceroyl-sphingosine), were significantly increased in response to DOX, the administration of periplocymarin notably decreased the levels of these three key metabolites (Figures 4A–D). Consistent with the observations of *in vivo* study, treatment with periplocymarin suppressed the DOX triggered elevation of Cer16:0, Cer18:0, Cer24:1, and Cer24:0 in H9c2 cells. However, periplocymarin alone did not decrease these ceramides compared with the control group (Figures 4H–K).

Clinical studies have shown that the ratios of Cer16:0, Cer18:0, Cer24:1, and Cer24:0 are strongly associated with the occurrence and development of cardiovascular disease, and positively correlated with the risk of cardiovascular disease. In our *in vivo* study, these ratios were significantly increased after DOX treatment, and they were reversed by the administration of periplocymarin (Figures 4E–G). And the *in vitro* experiments using H9c2 cells exhibited similar results (Figures 4L–N). Together with the foregoing observations, these data suggested that ceramide metabolic disturbance accounts for the beneficial effect of periplocymarin in DOX-induced HF.

### Target Network Analysis of Periplocymarin and HF

**Figure 5:**
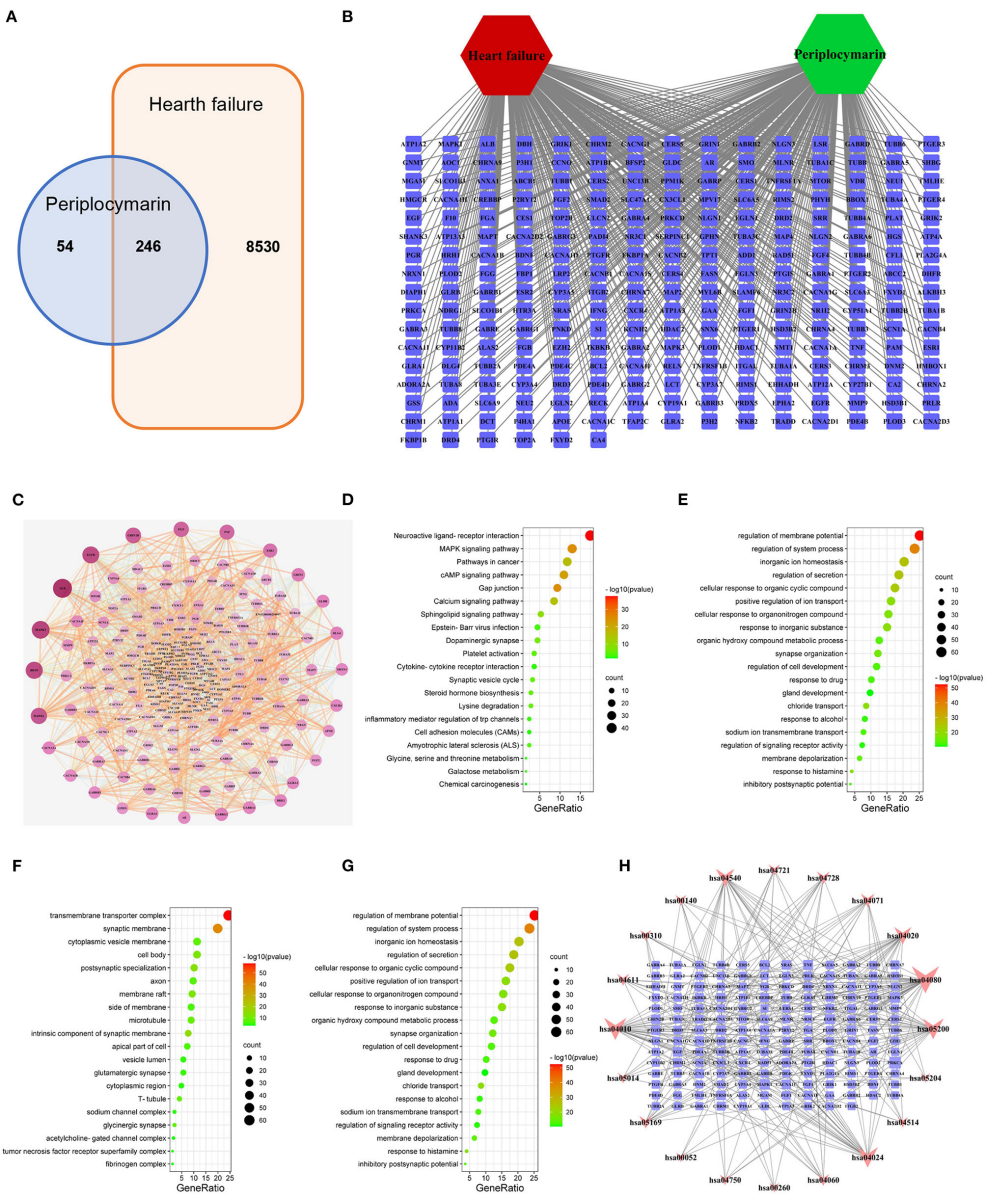
Network pharmacology to explore the mechanism of periplocymarin in the treatment of heart failure. **(A)** Intersection targets of periplocymarin and heart failure. Periplocymarin:300 targets; heart failure:8,770 targets. **(B)** The network of heart failure-targets-periplocymarin. Green hexagon represent compound, red hexagon represents disease, squares represent targets. **(C)** Protein-protein interaction network of 246 intersecting targets. The darkness of colors and sizes of circles represent the degree of freedom. **(D–G)** Bubble plot of Gene Ontology (GO) enrichment analysis of shared targets by periplocymarin and heart failure. Bubble plot: letters on the left are GO names, numbers on the bottom are the proportions of genes, sizes of the circles indicate the numbers of enriched genes, and colors reflect *p* values. The redder the colors are, the more enriched the genes are, and the smaller the *p* values are. The Kyoto Encyclopedia of Genes and Genomes (KEGG) pathway analyses **(D)**, biological process (BP) enrichment **(E)**, cellular components (CC) enrichment **(F)**, and molecular function (MF) enrichment **(G)** of the 246 intersecting genes. **(H)** Network of KEGG pathway and the 166 related targets. Squares represent the targets, inverted triangles represent the pathways; The area of nodes represents their degree, and the larger the area, the more important the node.

To further elucidate the underlying mechanism of how periplocymarin attenuates the DOX-induced HF, network pharmacology was performed. A total of 300 periplocymarin-related targets (Supplementary Table 11) were obtained through BATMAN-TCM prediction, and 8,776 HF-related targets (Supplementary Table 12) were obtained through the disease databases. Upon uploading targets of periplocymarin and HF to jvenn, 246 overlapping targets were found as shown in Figure 5A. The detailed information of these targets was shown in Figure 5B. These might be the targets of periplocymarin in the treatment of HF.

The STRING database was used to construct the PPI network of 246 shared targets by drugs and disease as shown in Figure 5C. The information derived from the STRING database was imported into Cytoscape3.7.2 software for further analysis, and a visualized PPI network was constructed, with the dot size and color reflecting the degree of freedom. The greater degree of freedom indicates that more biological functions were involved. The KEGG pathway and GO were analyzed by Metascape to clarify the mechanisms of how periplocymarin improved HF. The top 20 pathways were screened out according to the KEGG analysis (Supplementary Table 13). The results showed that the sphingolipid signaling pathway might be the related pathway for periplocymarin to treat HF, in which several key enzymes in ceramide biosynthesis were included (Figure 5D). The biological process enrichment analysis suggested that periplocymarin may improve HF by the regulation of membrane potential, system process, and inorganic ion homeostasis (Figure 5E). Besides, cellular component enrichment indicated that periplocymarin may regulate the transmembrane transporter complex, synaptic membrane, intrinsic component of the synaptic membrane, and postsynaptic specialization (Figure 5F). Molecular function enrichment suggested that periplocymarin may act on neurotransmitter receptor activity, gated channel activity, organic acid-binding, and structural constituent of the cytoskeleton (Figure 5G). The relationship between KEGG pathways and pathway-related genes was shown in Figure 5H. Squares represent the targets; inverted triangles represent the pathways. The area of the node represents its degree, and the larger the area, the more important the node. Ceramide biosynthesis enzymes, including CerS1, CerS2, CerS3, CerS4, and CerS5, were related to the ceramide biosynthesis pathway (has04071) (Figure 5H). These results further suggested that periplocymarin may improve HF by regulating the sphingolipid signaling and ceramide biosynthesis pathway.

### Periplocymarin Regulated the Expression of Enzymes Involved in the Production of Ceramide

**Figure 6:**
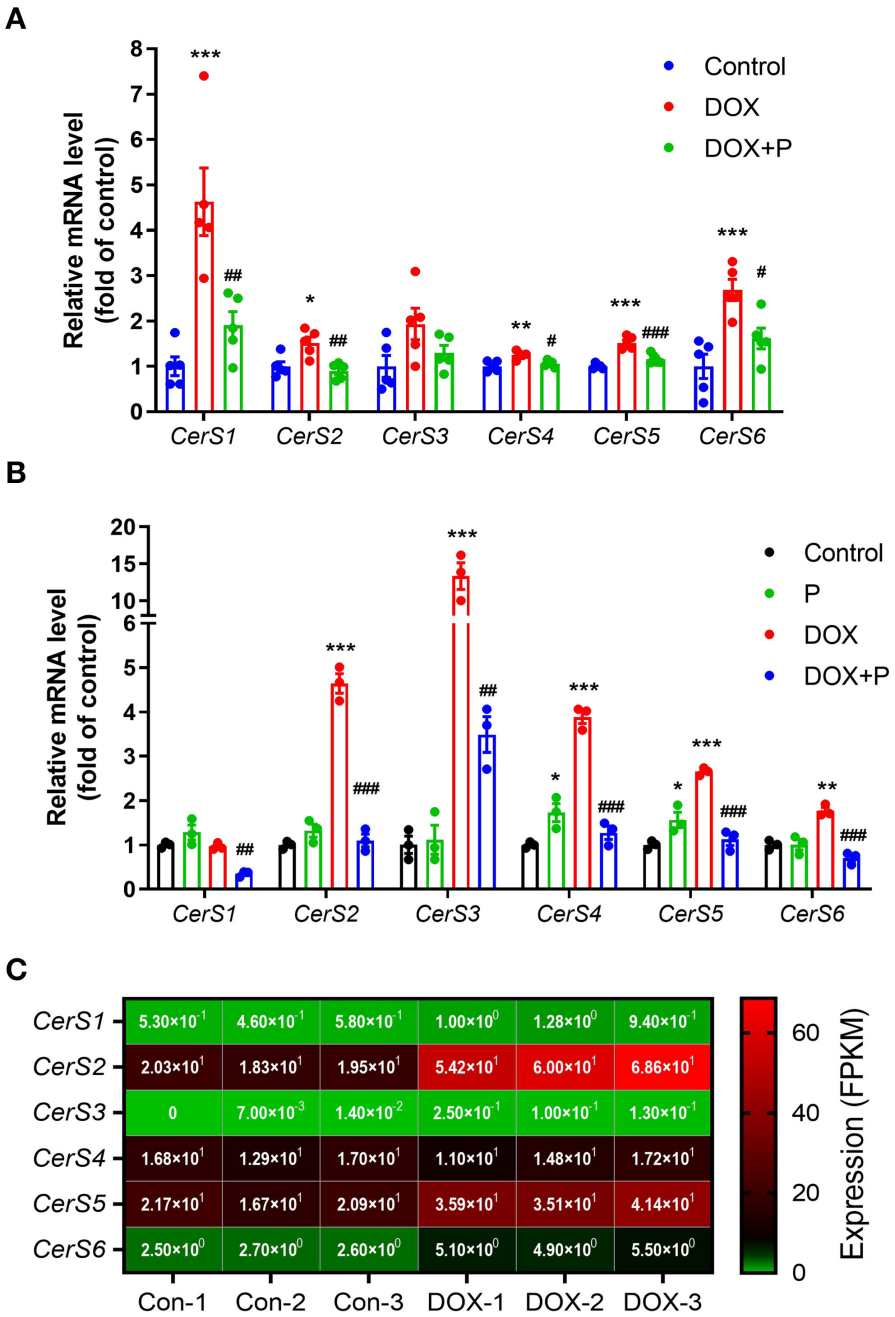
Periplocymarin regulated the expression of enzymes involved in ceramides metabolism. **(A)** *CerS1-6* messenger RNA (mRNA) expression in the heart tissues was measured by real-time PCR. Data are presented as mean ± SEM; *n* = 5, *p < 0.05, **p < 0.01, ***p < 0.001 vs. control; ^#^*p* < 0.05, ^##^*p*<0.01, ^###^*p*<0.001 vs. DOX. **(B)** *CerS1-6* mRNA expression in the primary cardiomyocytes was measured by real-time PCR. Data are presented as mean ± SEM; *n* = 3, *p < 0.05, **p < 0.01, ***p < 0.001 vs. control; ^##^*p* < 0.01, ^###^*p* < 0.001 vs. DOX. **(C)** The expression of *CerS1-6* in the RNA sequencing data (GSE157282).

We next aimed to determine the effect of periplocymarin on ceramide biosynthesis (Supplementary Figure 4). The analysis of several key enzymes in the ceramide biosynthesis pathway by real-time PCR revealed that the expression of *CerS1*-*6*, responsible for the ceramide synthesis. The expression of *CerS1, CerS2*, and *CerS4*-*6* in the messenger RNA (mRNA) level were increased in response to the DOX treatment *in vivo*, and administration of periplocymarin successfully decreased the level of *CerS1, CerS2*, and *CerS4*-*6* (Figure 6A). Similarly, *in vitro* study using primary cardiomyocytes revealed that except *CerS1*, the expression of *CerS2*-*6* were increased after DOX treatment, while pre-treatment with periplocymarin downregulated the mRNA levels of *CerS1-6* compared with the DOX group (Figure 6B). However, we also found that the expression of *Asah1* also increased after the treatment of DOX, and returned to normal after the treatment of periplocymarin (Supplementary Figure 5). *Asah1* encoded acid ceramidase, which was responsible for the metabolism of ceramide into sphingosine. The regulation of *Asah1* expression may be attributable to the fact that the body itself had a regulatory effect on the metabolism of ceramide. The expression of other genes related to ceramide syntheses, such as *Spt1*(encoding serin palmitoyl transferase), *Cerk*, and *ASMase*, showed no significant difference among the three groups (Supplementary Figure 5). In addition, we further validated our results using the published RNA-sequencing data from the GEO database (GSE157282). As shown in Figure 6C; Supplementary Figure 6, *CerS2, CerS4, CerS5*, and *Asah1* were relatively high-expressed, while *CerS1, CerS3*, and *CerS6* were relatively low-expressed in human induced pluripotent stem cell (hiPS)-derived cardiomyocytes. Strikingly, similar to our results, DOX treatment upregulated the expression of *CerS1, CerS2, CerS3, CerS5, CerS6*, and *Cerk*, except *CerS4* and *Asah1*. However, *Spt1* and *ASMase* were not detected in the RNA-sequencing data. Altogether, our findings implied that treatment with periplocymarin mainly regulated the expression *CerSs*, the enzymes related to ceramide synthesis, and thus amazingly rescuing DOX-induced ceramide accumulation.

## Discussion and Conclusion

The anthracycline antibiotic drug DOX, also known as Adriamycin, is widely used to treat malignant tumors, but its clinical application is greatly restricted by acute, sub-acute, or chronic side-effects (1). Doxorubicin exhibits a high affinity for cardiomyocytes, so DOX is very easy to lead to cardiotoxicity, causing arrhythmia, cardiomyopathy, and chronic heart failure (2, 3). Therefore, precise and effective therapy or drugs are urgently needed to counteract the cardiotoxicity of DOX. It is a common method to establish models of heart failure by abdominally injected DOX (29, 30). Periplocae Cortex is a traditional Chinese medicine applied in the cardiotonic formula of traditional Chinese medicine, which is widely used. Our group previously reported that periplocymarin is a bio-effective component of the Periplocae cortex with a strong cardiotonic effect (15). The present study demonstrated that the pre-treatment of periplocymarin significantly alleviates DOX-induced cardiomyopathy both *in vivo* and *in vitro*. The metabolomics analysis further revealed that periplocymarin treatment restored the metabolic disorders of ceramides induced by DOX. Periplocymarin may become a novel promising therapeutic agent to ameliorate cardiotoxicity and improve cardiac function in patients with DOX chemotherapy.

Ceramides were recognized as an important lipid second messenger, which mediates downstream events including cell proliferation, cell differentiation, cell cycle arrest, and cell apoptosis (31). Previous studies have demonstrated that ceramides are accumulated and ceramide biosynthesis enzymes are upregulated in the falling myocardium both in humans and in mice with HF (32). Besides, multiple investigations suggested that ceramides contribute to cardiomyocytes apoptosis. For instance, ceramide analogs are sufficient to trigger the apoptosis of cardiomyocytes (23) and palmitate-induced endogenous ceramides also initiate apoptosis of cardiomyocytes (20). Additionally, the overexpression of ceramide synthesis key enzymes, such as *CerS2*, promoted mitochondrial dysfunction and apoptosis in cardiomyocytes (33). More importantly, DOX exposure could induce ceramide generation in cultured adult rat ventricular myocytes and then lead to myocyte apoptosis (34). Based on these previous works, we firstly detected and found that DOX elevated the contents of Cer16:0, Cer18:0, and Cer24:1 *in vivo* and *in vitro*. More importantly, periplocymarin treatment obviously decreased the level of the ceramides. These results suggested that periplocymarin efficaciously inhibit DOX-induced ceramides metabolic disorders.

In recent years, the role of ceramides in cardiovascular disease has received more and more attention. In patients with or without coronary artery disease, it was found that elevated plasma ceramides concentrations are independently related to adverse cardiovascular events (35). Clinical studies have shown that risk prediction models based on plasma ceramides levels can effectively identify people at high risk of adverse cardiovascular events in the future, predict the recurrence of coronary events, and then optimize the secondary prevention strategy, especially for those who have reached the target of blood lipids. Some studies have demonstrated that a high ratio of Cer16:0/Cer24:0 is tightly associated with potentially adverse subclinical cardiac structure and function changes (36). Another study pointed that the ratio of Cer24:0/Cer16:0 may become a helpful biomarker for the risk of coronary heart disease, HF, and all-cause mortality in the community (37). The concentrations of Cer16:0, Cer22:0, Cer24:0, and Cer24:1 ceramides were found to be independent risk factors for the occurrence of primary end events of cardiovascular disease (38). By conducting a long-term follow-up on 495 patients with coronary angiography, Meeusen et al. found that Cer16:0, Cer18:0, and Cer24:1 ceramide levels and the ratios of Cer16:0/Cer24:0, Cer18:0/Cer24:0, and Cer24:1/Cer24:0 ceramides could predict the occurrence of major adverse cardiovascular events during the 4-year follow-up (35). In our study, we found the contents of Cer16:0, Cer18:0, and Cer24:1 were significantly increased after the treatment of DOX, periplocymarin decreased the levels of these metabolites, and the same trend was observed *in vivo* and *in vitro*. Cer16:0/Cer24:0, Cer18:0 /Cer24:0, and Cer24:1/Cer24:0 ceramide ratios were elevated in the DOX group, periplocymarin pre-treatment reversed this phenomenon. Combined with the echocardiography result, we confirmed that periplocymarin could improve metabolic disorders of ceramides and then improve HF.

Ceramides can be synthesized through the *de novo* pathway in the ER or the salvage pathway in lysosomes (39). The *de novo* pathway for ceramides synthesis with the condensation of serine and palmitate to form 3-keto-dihydroshingosine is catalyzed by the enzyme serine palmitoyltransferase (SPT) (39, 40). 3-Ketosphinganine reductase catalyzed 3-ketosphinganine converted to sphinganine. Sphinganine is then converted to ceramide by ceramide synthase (CerS) (40, 41). Six distinct CerS have been identified in mammalian cells that can be distinguished by their high specificity for different fatty acyl chain substrates (41). The next step of the pathway is the transition of dihydroceramide to ceramide, a reaction that is catalyzed by dihydroceramide desaturase (41). Ceramide can be resolved into sphingosine by acidic ceramidase (ASAH), at the same time, sphingosine can return to ceramide by CerS (41). The salvage pathway in the lysosomes consists of the degradation of sphingomyelin to ceramides by acid sphingomyelinase (ASMase) and ceramides to sphingosine by ceramidases (39, 42). Cells possess several isoforms of SMase, they have been classified into acid, neutral, and alkaline SMase according to their features. Acid sphingomyelinase and NSMase are mainly involved in signal transduction processes. Besides, sphinganine can be converted to ceramide-1-phosphate by Cerk. In our study, the expression of *Spt1, Cerk, Asah1, ASMase*, and *CerS1-6* were detected by Real-time PCR. No obvious difference was found in the expression of *Spt1, ASMase*, and *Cerk* among the three groups. However, the mRNA levels of *CerS2-6* were obviously elevated in response to DOX *in vivo* and *in vitro*. This elevation was significantly ameliorated after periplocymarin treatment. Mass-spectrometry analyses have shown that ceramides levels were increased in the DOX group, which was reversed by periplocymarin. Altogether, these results suggested that the treatment of DOX resulted in ceramide accumulation by up-regulating the expression of *CerS2*-*6*, which are responsible for encoding key enzymes in ceramide synthesis. The administration of periplocymarin successfully prevented this phenomenon. However, the acidic ceramidase (*Asah1*) expression was higher in the DOX group, and periplocymarin treatment reversed its levels to a normal level that was consistent with the literature (43). That may be a self-protection mechanism of the body because the Asah1 overexpression could reduce the concentrations of ceramides in the cells, thus limiting the harmful accumulation of ceramides in the liver (43).

Given that periplocymarin is a promising anti-cancer drug (13, 14), the use of periplocymarin in clinical patients receiving DOX chemotherapy may have advantages: firstly, it synergistically promotes the anti-cancer effect of DOX; secondly, it reduces the dose of DOX; last but not least, it improves DOX-induced cardiac dysfunction. However, whether and how periplocymarin can cooperate with DOX still needs further experimental verification.

In conclusion, our study provided first-hand evidence that periplocymarin ameliorated DOX-induced HF. Through combined metabolomic analysis study and experimental validation, we confirmed that periplocymarin regulated ceramide synthases gene expression to improve ceramides metabolism disorder, and ameliorated DOX-induced cardiomyocytes apoptosis and HF. Periplocymarin is promising to be a potent therapeutic agent for HF, especially DOX-induced HF.

## Data Availability Statement

The datasets presented in this study can be found in online repositories. The names of the repository/repositories and accession number(s) can be found in the article/Supplementary Material.

## Ethics Statement

The animal study was reviewed and approved by Animal Care and Use Review Committee of Dalian Medical University.

## Author Contributions

WY: conceptualization, methodology, data curation, formal analysis, funding acquisition, writing—original draft, and writing—review and editing. LQ: investigation, project administration, and resources. RY: methodology, investigation, software, and validation. HX: funding acquisition, supervision, and visualization. All authors contributed to the article and approved the submitted version.

## Funding

This work was supported by the National Natural Science Foundation of China Grants (81900267), Doctoral Scientific Research Initiation Fund of Liaoning Province (2019-BS-078), Dalian Youth Science and Technology Star Project (2019RQ103), and Scientific Research Fund Project of Liaoning Provincial Department of Education (LZ2019014, LZ2020053, LJKZ0840, and LJKZ0847).

## Conflict of Interest

The authors declare that the research was conducted in the absence of any commercial or financial relationships that could be construed as a potential conflict of interest.

## Publisher's Note

All claims expressed in this article are solely those of the authors and do not necessarily represent those of their affiliated organizations, or those of the publisher, the editors and the reviewers. Any product that may be evaluated in this article, or claim that may be made by its manufacturer, is not guaranteed or endorsed by the publisher.
