# Supplementary material for: Periplocymarin Alleviates Doxorubicin-Induced Heart Failure and Excessive Accumulation of Ceramides
Source: Front Cardiovasc Med. 2021 Nov 19;8:732554. doi: 10.3389/fcvm.2021.732554 (PMC8639694; doi:10.3389/fcvm.2021.732554)
Supplement: Supplementary file 1 [file Data_Sheet_1.pdf]

Periplocymarin alleviates doxorubicin-induced heart failure and excessive accumulation of ceramides

Weijing Yun[1, *], Lei Qian[1, *], Ruqiang Yuan[1, *], Hu Xu[1, #].

1. Advanced Institute for Medical Sciences, Dalian Medical University, Dalian 116044, China.

* These authors contributed equally to this work.

# Corresponding Author: Hu Xu, Ph.D. (xuhu1024@126.com), Advanced Institute for Medical Sciences, Dalian Medical University, Dalian, China.

Supplementary information

Supplementary method

1. Cell viability assay

H9c2 cells were seeded in a 96-well plate with 5000 cells/well, and treated with the following conditions: fresh culture medium alone (control), fresh culture medium with different concentrations (0-100μM) of periplocymarin. Cell viability was assessed by a Cell Counting Kit-8 (CCK-8; Dojindo Molecular Technologies, Gaithersburg, MD, USA) according to manufacturer's instructions. Briefly, after treatment, the CCK-8 solution was added to the culture medium and incubated at 37°C for 4 h. The absorbance was read at 450 nm with a microplate reader (Bio-Rad, Hercules, CA, USA). Cell viability was calculated by (experimental group absorbance value/control group absorbance value) × 100%.

2. Determination of intracellular ROS

The cellular ROS level was determined using ROS detection kits (Beyotime Biotechnology, China). In brief, following DOX and periplocymarin treatment, H9c2 cells were washed third with 1 washing buffer and then incubated with serum free DMEM (10 mM DCFH-DA) at 37 °C for 30 min. The fluorescence intensity of DCF was quantified by using the confocal microscope at Ex./Em. = 488/525 nm.

Supplementary data

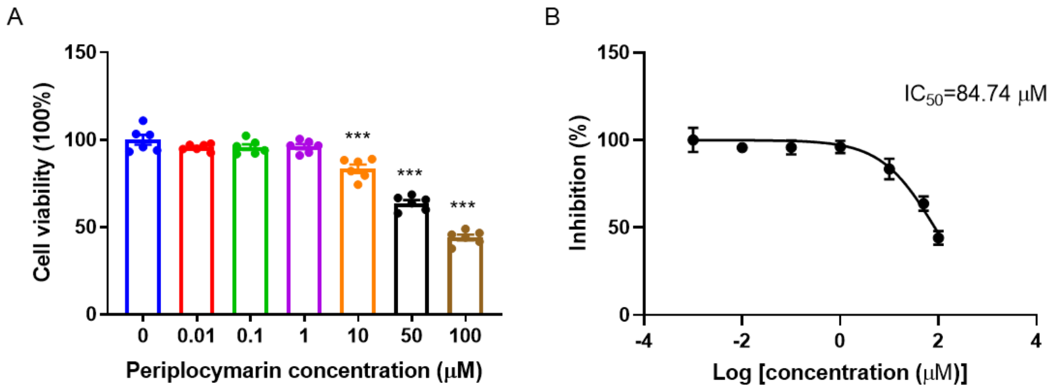

Fig. S1. Effect of periplocymarin on the cell viability of H9c2 cell. H9c2 cells were treated with different doses (0, 0.01, 0.1, 1, 10, 50 and 100 μM) of periplocymarin for 24 h. After treatment, cell viability of H9c2 cells was detected by CCK-8 assay (A). And the IC_{50} was calculated (B). Each experiment was repeated at least three times. Data shown are means ± SEM; n=6, ***p<0.001 vs. control.

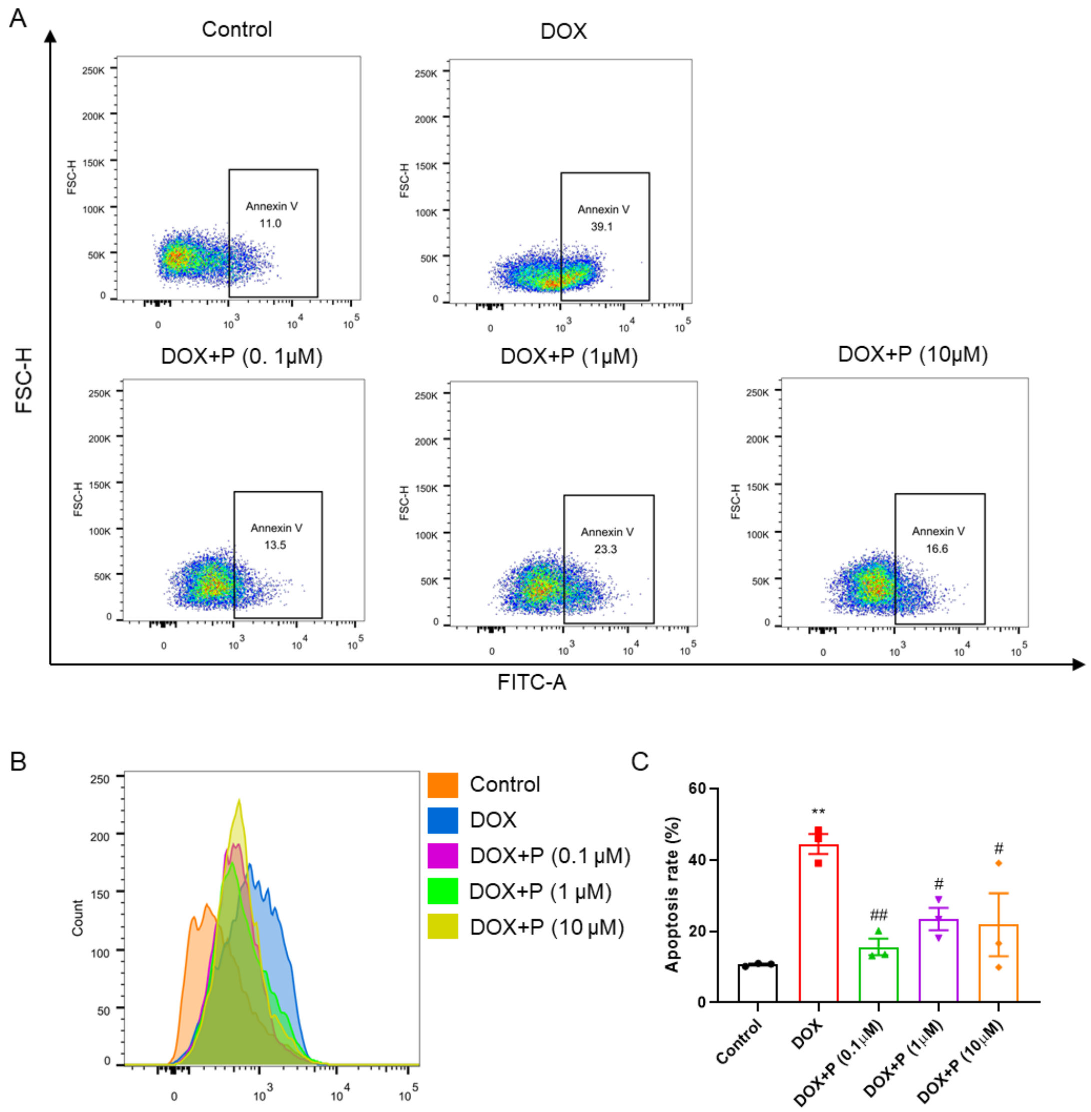

Fig. S2. Periplocymarin inhibits DOX-induced myocardial apoptosis in vitro.

(A). Flow cytometric scatterplot of Annexin-V-FLTC staining of cell apoptosis.

(B). Flow cytometric histogram of Annexin-V-FLTC staining of cell apoptosis.

(C). Quantitative analysis of apoptotic cells in different groups using bar graphs. Data shown are means ± SEM; n=3. **p<0.01 vs. control group, $^{\#}$p<0.01, $^{\#\#}$p<0.01 vs DOX group.

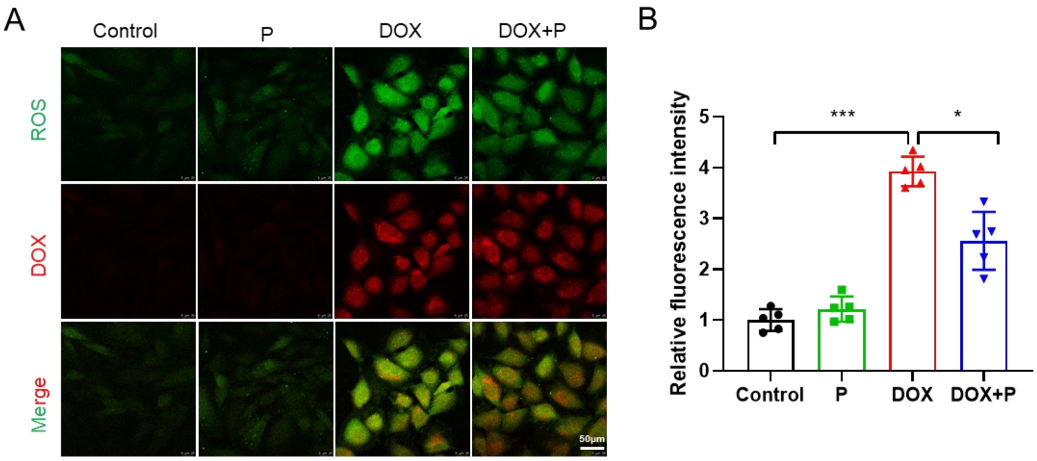

Fig. S3. The effect of DOX and periplocymarin on the production of ROS was measured by DCFH-DA staining via confocal microscope(A). And the quantitative analysis of mean DCF fluorescence intensity in different groups(B). Data shown are means ± SEM; n=5, *p<0.05, ***p < 0.001.

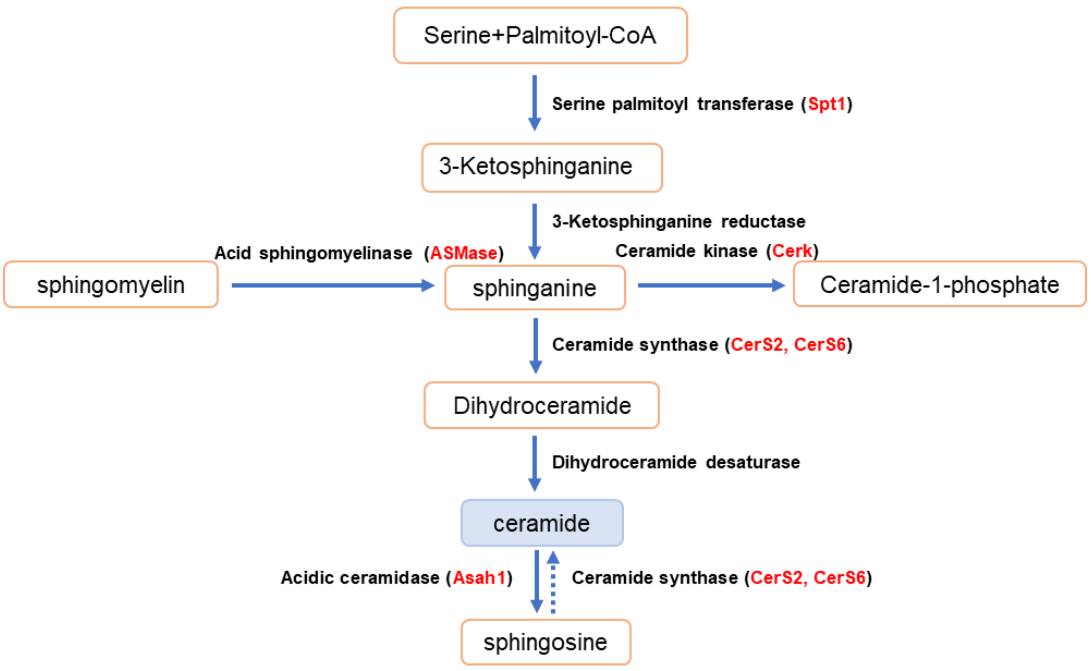

Fig. S4. Schematic representation of ceramide biosynthesis pathways.

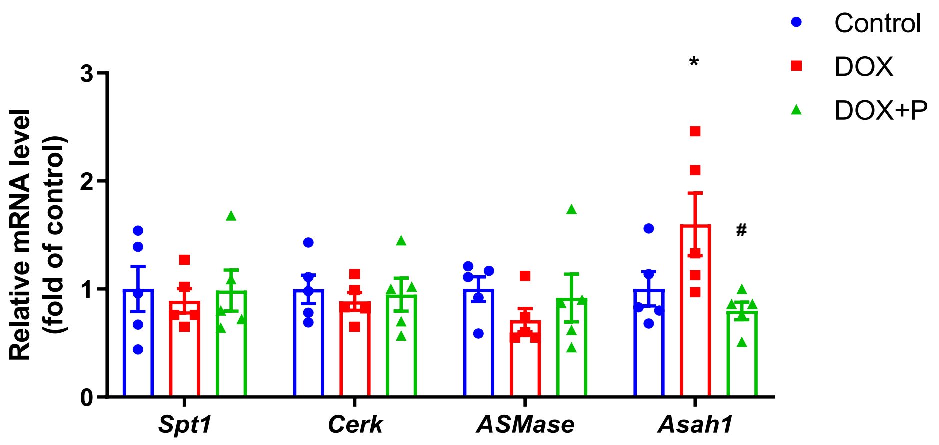

Fig. S5. Real-time PCR analysis of *Spt1, Cerk, ASMase* and *Asah1* mRNA levels in the hearts. Data shown are means ± SEM; n=5, *p<0.05 vs. control, #p < 0.05 vs. model.

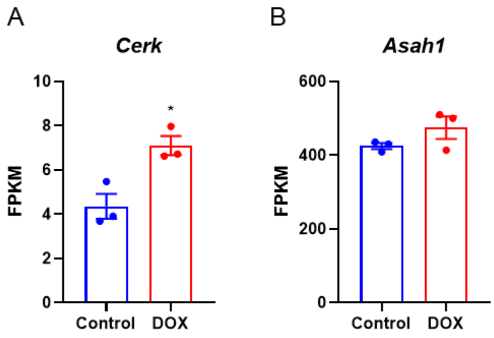

Fig. S6. The expressions of *Cerk* (A) and *Asah1* (B) in the RNA sequencing data (GSE157282).

Table S1. Primers for quantitative RT-PCR.

PCR production	Forward primer	Reverse primer
Rat Cers1	GTCTGTCGCCTATCTCCGTG	GTCCCTGAAAGGGTGTCCTG
Rat Cers2	TCCCTTTTCCCCTCCCTTCA	AGTCTGACACAGTCATGTTTTATTT
Rat Cers3	GCCCCGCAGCTTTTGAAG	CATTGGCTAGAGGTGTGGCA
Rat Cers4	TCTGTGTTCCTTCGTGGGTG	AGGGCCAAATTCAGGGTCTG
Rat Cers5	CAGCAGGTCTCTTCTCCGTG	AGCCAAGGCATCTTAGAGATAGA
Rat Cers6	GTTAGCCTGGAGCTGGTCTC	TCCCCTGCCCTCTGAAAATG
Mouse Cers1	AGAGCCCATGCCGAGTTAT	TCCAGGCGCTCTCAGGTAA
Mouse Cers2	TACGCGGGATGGAAGAACAC	TGGGCCATACGCAGAATGAA
Mouse Cers3	ACTTTCGAGGGTGGGTCTCT	AGCCAGTATCTCTCCGACCA
Mouse Cers4	TTCTGAACCTCCAGCATGTCG	GATCCTGCACACCCATCCAC
Mouse Cers5	CTGGTCGATGCCTTGGTTCT	CTCGCACCATGTTGTTGACG
Mouse Cers6	CGGCTGGGCATATTTCCTCT	AAAGGGTTCCACTTCCCAGC
Mouse ASAH1	AAAGTCTTCTCACCTGGGTCC	GATTTATGGTGTGCCACGGA
Mouse SPT1	GAGAGCCTGTCCTTTGGAGTC	AGCAGTATCCTTGACCGGAG
Mouse Cerk	TCGGTACTGGTGTCGGAGAT	CGGTCTTGAAGTCAGGCTCT
Mouse Asmase	GAGGAAACTCTGAGCCGACC	GTAGACCAGGTTGTGCCAGG
Mouse 18S	GAAACGGCTACCACATCCAAGG	GCCCTCCAATGGATCCTCGTTA

Table S2. Metabolites in positive ion mode by MRM assay.

Sequence	Q1	Q3	MRM Name	DP	EP	CE	CXP	Chemical Formula
1	620.7	264.3	Ceramide(d18:1/22:0 OH)	100	10	40	10	$C_{40}H_{79}NO_4$
2	572.7	264.3	Ceramide(d18:1/20:2)	100	10	40	10	$C_{38}H_{71}NO_3$
3	600.7	264.3	Ceramide(d18:1/22:2)	100	10	40	10	$C_{40}H_{75}NO_3$
4	576.57	264.3	Ceramide(d18:1/20:0)	100	10	40	10	$C_{38}H_{75}NO_3$
5	548.6	264.3	Ceramide(d18:1/18:0)	100	10	40	10	$C_{36}H_{71}NO_3$
6	604.6	264.3	Ceramide(d18:1/22:0)	100	10	40	10	$C_{40}H_{79}NO_3$
7	632.6	264.3	Ceramide(d18:1/24:0)	100	10	40	10	$C_{42}H_{83}NO_3$
8	630.7	264.3	Ceramide(d18:1/24:1)	100	10	40	10	$C_{42}H_{81}NO_3$
9	628.7	264.3	Ceramide(d18:1/24:2)	100	10	40	10	$C_{42}H_{79}NO_3$
10	646.7	264.3	Ceramide(d18:1/25:0)	100	10	40	10	$C_{43}H_{85}NO_3$
11	520.5	264.3	Ceramide(d18:1/16:0)	100	10	40	10	$C_{34}H_{67}NO_3$
12	536.7	264.3	Ceramide(d18:1/16:0 OH)	100	10	40	10	$C_{34}H_{67}NO_4$
13	518.5	264.3	Ceramide(d18:1/16:1)	100	10	40	10	$C_{34}H_{65}NO_3$
14	516.5	264.3	Ceramide(d18:1/16:2)	100	10	40	10	$C_{34}H_{63}NO_3$
15	562.7	264.3	Ceramide(d18:1/18:1 OH)	100	10	40	10	$C_{36}H_{69}NO_4$
16	546.7	264.3	Ceramide(d18:1/18:1)	100	10	40	10	$C_{36}H_{69}NO_3$
17	560.7	264.3	Ceramide(d18:1/18:2 OH)	100	10	40	10	$C_{36}H_{67}NO_4$
18	544.7	264.3	Ceramide(d18:1/18:2)	100	10	40	10	$C_{36}H_{67}NO_3$
19	592.7	264.3	Ceramide(d18:1/20:0 OH)	100	10	40	10	$C_{38}H_{75}NO_4$
20	590.7	264.3	Ceramide(d18:1/20:1 OH)	100	10	40	10	$C_{38}H_{73}NO_4$
21	574.7	264.3	Ceramide(d18:1/20:1)	100	10	40	10	$C_{38}H_{73}NO_3$
22	602.7	264.3	Ceramide(d18:1/22:1)	100	10	40	10	$C_{40}H_{77}NO_3$
23	616.7	264.3	Ceramide(d18:1/22:2 OH)	100	10	40	10	$C_{40}H_{75}NO_4$
24	618.7	264.3	Ceramide(d18:1/23:0) or Ceramide(d18:1/22:1 OH)	100	10	40	10	$C_{41}H_{81}NO_3$
25	644.7	264.3	Ceramide(d18:1/24:2 OH)	100	10	40	10	$C_{42}H_{79}NO_4$
26	676.7	264.3	Ceramide(d18:1/26:0 OH)	100	10	40	10	$C_{44}H_{87}NO_4$
27	660.7	264.3	Ceramide(d18:1/26:0)	100	10	40	10	$C_{44}H_{87}NO_3$
28	658.7	264.3	Ceramide(d18:1/26:1)	100	10	40	10	$C_{44}H_{85}NO_3$
29	656.7	264.3	Ceramide(d18:1/26:2)	100	10	40	10	$C_{44}H_{83}NO_3$
30	534.7	264.3	Ceramide(d18:1/16:1 OH)	100	10	40	10	$C_{34}H_{65}NO_4$
31	648.7	264.3	Ceramide(d18:1/24:0 OH)	100	10	40	10	$C_{42}H_{83}NO_4$

Table S3. extracted ions in six QC samples in serum.

Num.	Name	Peak area								RSD
1	Cer 16:1 OH	40957.33	42072.67	39095.00	47261.67	40753.00	42067.33	46722.67	44366.33	6.82
2	Cer 24:0 OH	17542287.67	20139209.33	18902600.33	22357009.33	25368105.00	23334785.00	22243591.33	22534429.00	11.75
3	Cer 22:0 OH	403225.00	464681.33	434717.33	502075.33	503635.33	565653.67	533035.33	530323.33	11.06
4	Cer 20:2	583076.00	657150.33	501883.67	734766.67	650536.00	610082.00	667626.67	639345.00	10.83
5	Cer 22:2	9095407.00	11675648.00	12032290.33	12569225.00	14091595.00	13294734.00	14048312.67	13984947.00	13.48
6	Cer 20:0	1208971.00	1364833.67	1329733.33	1269860.67	1544478.67	1453638.33	1528147.67	1495194.67	8.91
7	Cer 18:0	1172245.33	1048313.00	1043541.67	1141032.67	1230636.00	1427889.67	1186068.00	1011843.33	11.58
8	Cer 22:0	22291013.00	27458378.67	24482229.67	27089309.33	28500834.67	25956107.33	29594552.67	26172444.33	8.70
9	Cer 24:0	25678963.33	30858387.00	28164602.33	32706120.00	30346357.33	32286372.00	32331243.00	31509295.67	7.97
10	Cer 24:1	20284462.00	23611153.33	20960432.33	24423168.33	28180204.33	25683637.67	26694333.00	24894683.00	11.07
11	Cer 24:2	2040810.00	2188578.67	2050077.00	2066107.33	2798930.00	2118749.67	2303747.67	1979804.33	12.06
12	Cer 25:0	1004244.67	1123759.00	1092889.33	1152795.33	1161579.67	1019248.33	1048123.33	1125398.33	5.54
13	Cer 16:0	2337330.67	2214703.67	2358335.33	2400227.67	2755127.67	2335109.67	2507163.67	1960689.33	9.63
14	Cer 16:0 OH	120066.33	108016.67	114340.00	105309.00	122338.33	80302.00	115727.67	127318.67	13.07
15	Cer 16:1	251157.33	269641.67	280031.67	291481.00	284053.00	298575.33	291177.67	307934.00	6.23
16	Cer 16:2	46027.33	45642.00	43470.33	44446.67	40848.00	51395.00	44671.33	45314.00	6.58
17	Cer 18:1 OH	225475.67	229877.00	204522.67	233264.67	234492.67	235199.67	285298.33	231139.33	9.65
18	Cer 18:1	84510.67	72810.00	86941.00	69619.00	95085.33	66304.33	77806.33	83554.67	12.19
19	Cer 18:2 OH	40208.33	43546.33	35318.67	38673.33	44555.33	41619.67	50531.00	34773.67	12.57
20	Cer 18:2	203590.00	201002.67	206480.33	219748.33	208615.33	209347.67	200768.00	228791.33	4.66
21	Cer 20:0 OH	66459.67	64637.00	81735.33	68488.00	70650.33	79128.00	80926.00	69975.67	9.35
22	Cer 20:1 OH	542820.67	614854.00	598790.33	657460.33	680232.33	688509.67	706223.00	594825.33	8.88
23	Cer 20:1	65859.00	84900.33	75186.67	76121.00	75660.00	81995.67	73488.33	71185.67	7.86
24	Cer 22:1	1112775.33	1355044.00	1188919.67	1322446.00	1500953.67	1433221.33	1412779.00	1569929.33	11.23
25	Cer 22:2 OH	979632.33	1321039.00	1178052.33	1370225.33	1154753.67	1507951.67	1257452.00	1300267.00	12.58
26	Cer 23:0	10617832.67	12818032.33	12142866.67	13162491.00	14029670.00	14437224.67	13963362.67	11648370.67	10.26
27	Cer 24:2 OH	414285.33	337230.67	364557.00	389917.00	439423.67	449131.67	370489.00	383552.67	9.74
28	Cer 26:0 OH	54474.33	76545.33	65599.00	62544.67	63499.00	71544.33	75020.67	64676.67	10.92
29	Cer 26:0	279430.67	240734.67	295269.67	296960.33	230243.33	269717.00	243781.00	263029.33	9.49
30	Cer 26:1	171103.67	176815.33	150106.00	171783.67	208170.33	185492.33	172471.00	173909.67	9.24
31	Cer 26:2	40945.00	40135.67	47318.00	49208.00	55820.33	46351.33	45314.00	49924.00	10.76

Table S4. The model parameters of OPLS-DA in serum.

	R^2X	R^2Y	Q^2(cum)
Control VS DOX	0.898	0.902	0.863
DOXVS DOX+P	0.932	1	0.941

Table S5. VIP value of different metabolites come from control and DOX group in serum.

Name	VIP
Cer 24:1	3.49954
Cer 24:0 OH	3.32994
Cer 23:0	1.40985
Cer 24:2	1.25155
Cer 22:2	1.17972
Cer 16:0	1.07731

Table S6. VIP value of different metabolites come from DOX and DOX+P group in serum.

Name	VIP
Cer 22:0	1.45505
Cer 25:0	1.26429
Cer 24:1	1.2335
Cer 24:0	1.20111
Cer 26:1	1.18804
Cer 22:2 OH	1.12223
Cer 18:0	1.10362
Cer 20:0	1.09678
Cer 16:0	1.09228
Cer 20:1 OH	1.06412
Cer 20:2	1.06328
Cer 22:1	1.0523
Cer 22:0 OH	1.03578
Cer 24:2	1.02023
Cer 18:2 OH	1.00816

Table S7. extracted ions in six QC samples in H9C2 cell.

Num.	Name	Peak area						RSD
1	Cer 16:1 OH	14165.43	14793.71	15796.18	14480.90	14852.80	15844.48	4.60
2	Cer 24:0 OH	256230.67	296928.10	314923.32	345786.32	306094.43	358605.68	11.68
3	Cer 22:0 OH	12681.89	14440.39	13446.25	16155.97	15194.60	15060.63	8.70
4	Cer 20:2	2791.24	2729.74	2650.21	2865.83	2527.82	2649.15	4.42
5	Cer 22:2	26585.56	24496.78	25206.62	27625.12	26041.22	26167.40	4.17
6	Cer 20:0	44872.91	52191.53	50538.39	54513.34	51504.33	56801.25	7.84
7	Cer 18:0	96211.68	116910.22	120795.41	130497.07	118275.75	132562.38	10.89
8	Cer 22:0	111684.12	123218.55	132679.22	153618.32	136894.21	148012.66	11.55
9	Cer 24:0	236366.65	230651.23	257624.13	238819.05	269519.50	270289.00	6.99
10	Cer 24:1	272578.96	263203.57	299541.98	306601.58	283905.48	286669.74	5.67
11	Cer 24:2	15222.52	15752.90	18811.80	19368.57	18712.25	20123.36	11.20
12	Cer 25:0	6923.43	6961.64	8292.65	7886.68	8698.97	8640.55	10.09
13	Cer 16:0	192008.26	244966.79	247650.32	273275.96	257930.42	286927.69	13.07
14	Cer 16:0 OH	1343.58	1137.92	1289.08	1496.57	1353.26	1238.82	9.22
15	Cer 16:1	1562.06	1486.20	1573.48	1764.49	1759.08	1692.59	7.04
16	Cer 16:2	12084.54	13976.63	15276.49	16482.01	14172.08	16930.57	12.08
17	Cer 18:1 OH	2477.66	2761.07	2554.80	3156.52	3325.17	3248.75	12.65
18	Cer 18:1	3983.48	3885.15	3699.33	4554.74	4330.40	4182.10	7.60
19	Cer 18:2 OH	1103.54	1056.05	1005.92	1014.41	1032.44	1038.23	3.37
20	Cer 18:2	1997.23	1797.24	1892.46	2277.37	2249.83	2007.37	9.42
21	Cer 20:0 OH	38351.43	44129.10	39322.80	47845.47	39347.05	49631.56	11.23
22	Cer 20:1 OH	3385.55	4052.13	3482.63	3944.52	3915.19	4707.04	12.06
23	Cer 20:1	2369.33	2173.33	1825.64	2478.89	1944.75	2123.40	11.47
24	Cer 22:1	13169.34	13862.57	13295.58	16457.41	12694.06	13506.47	9.71
25	Cer 22:2 OH	11397.97	11999.50	13382.58	14087.05	13372.82	14935.29	9.91
26	Cer 23:0	29980.34	34558.55	34535.73	36055.62	38382.40	39229.42	9.34
27	Cer 24:2 OH	4714.03	5086.75	4854.01	6012.10	5108.58	5046.09	8.86
28	Cer 26:0 OH	4586.29	5654.14	4940.01	5593.88	5366.88	5038.29	7.98
29	Cer 26:0	7865.74	7651.88	7776.36	7805.65	7507.77	8955.63	6.56
30	Cer 26:1	10135.46	10511.32	9119.11	9500.62	9967.25	10070.82	5.02
31	Cer 26:2	1537.72	1746.38	1741.65	1838.68	1922.78	1798.53	7.35

Table S8. The model parameters of OPLS-DA in H9C2 cell.

	R^2X	R^2Y	Q^2(cum)
Control VS DOX	0.912	0.975	0.904
Control VS DOX+P	0.972	0.984	0.981

Table S9. VIP value of different metabolites come from control and DOX group in H9C2 cell.

Name	VIP
Cer 24:0 OH	2.57948
Cer 24:1	2.49988
Cer 16:0	2.12698
Cer 24:0	2.11194
Cer 22:0	1.57051
Cer 18:0	1.49148

Table S10. VIP value of different metabolites come from DOX and DOX+P group in H9C2 cell.

Name	VIP
Cer 24:0	2.5083
Cer 24:0 OH	2.25757
Cer 24:1	2.12897
Cer 16:0	1.95275
Cer 18:0	1.02906

Table S11. Periplocymarin related targets

**Gene**	Score
**ATP1A1**	122.778
**GLRA3**	80.882
**GABRB3**	80.882
**GLRA1**	23
**NRXN1**	22.373
**GLRA2**	22.373
**GLRB**	22.373
**GLRA4**	22.373
**ATP1A3**	15.365
**GABRB1**	15.365
**GABRB2**	15.365
**ATP1A2**	15.365
**FKBP1A**	8.253
**TOP2A**	8.253

**SLC47A1**	8.253
**ATP1A4**	8.253
**CYP3A4**	8.253
**TUBB**	8.253
**MAP2**	8.253
**TUBB4A**	8.253
**TUBA3E**	8.253
**CYP51A1**	8.253
**BCL2**	8.253
**TUBB2B**	8.253
**HMGCR**	8.253
**NR1I2**	8.253
**KCNH2**	8.253
**MTOR**	8.253
**TUBB4B**	8.253
**ITGB2**	8.253
**CYP3A7-CYP3A51P**	8.253
**TUBA3C**	8.253
**MAPT**	8.253
**TUBA1C**	8.253
**TUBB2A**	8.253
**TUBA1B**	8.253
**PADI4**	8.253
**MLNR**	8.253
**CYP3A7**	8.253
**SLCO1B3**	8.253
**ITGAL**	8.253
**TUBB1**	8.253
**TUBA4A**	8.253
**ALB**	8.253
**HDAC2**	8.253
**MAP4**	8.253
**ABCB1**	8.253
**FGF2**	8.253
**TUBA3D**	8.253
**SLCO1B1**	8.253
**TUBA1A**	8.253
**GPHN**	5.859
**GABRA2**	5.859
**CHRNA4**	5.859
**SHANK3**	5.859
**ADORA2A**	5.859
**GABRG3**	5.859

**IGSF9B**	5.859
**CX3CL1**	5.859
**RELN**	5.859
**APOE**	5.859
**GSS**	5.859
**NTSR1**	5.859
**GRIN3B**	5.859
**CHRNA7**	5.859
**UNC13B**	5.859
**GNMT**	5.859
**GABRE**	5.859
**NPAS4**	5.859
**GABRA3**	5.859
**GABRG1**	5.859
**ATP1B1**	5.859
**NR3C1**	5.859
**GABRA4**	5.859
**DRD2**	5.859
**DLG4**	5.859
**NLGN1**	5.859
**AOC1**	5.859
**HRH1**	5.859
**GLDC**	5.859
**GRIN2B**	5.859
**DRD4**	5.859
**RIMS2**	5.859
**NLGN2**	5.859
**ALAS2**	5.859
**SLC6A9**	5.859
**DIAPH1**	5.859
**DRD3**	5.859
**GRIN3A**	5.859
**BDNF**	5.859
**GABRA5**	5.859
**FXYD2**	5.859
**SLC6A5**	5.859
**GABRA6**	5.859
**GABRA1**	5.859
**GABRG2**	5.859
**RIMS1**	5.859
**CHRNA9**	5.859
**GRIK1**	5.859
**GRIK2**	5.859
**RAD51**	5.859

**GRIN1**	5.859
**SRR**	5.859
**NLGN3**	5.859
**CXCR4**	5.785
**GAA**	5.785
**MGAM**	5.785
**KDM5D**	5.785
**ALKBH3**	5.785
**OGFOD2**	5.785
**P3H3**	5.785
**TMLHE**	5.785
**ESR1**	5.785
**P4HTM**	5.785
**PLOD1**	5.785
**PTGIS**	5.785
**EGLN2**	5.785
**ALKBH2**	5.785
**BBOX1**	5.785
**PGR**	5.785
**AR**	5.785
**P2RY12**	5.785
**PTGIR**	5.785
**PRKCA**	5.785
**PAM**	5.785
**AMY2A**	5.785
**SLC23A1**	5.785
**OGFOD1**	5.785
**DHFR**	5.785
**DBH**	5.785
**EGLN3**	5.785
**SMO**	5.785
**PLOD3**	5.785
**P4HA1**	5.785
**SI**	5.785
**PTGFR**	5.785
**EGLN1**	5.785
**P3H2**	5.785
**LCT**	5.785
**LRP2**	5.785
**ANXA1**	5.785
**CYP19A1**	5.785
**PLOD2**	5.785
**P3H1**	5.785
**PRKCD**	5.785

**PHYH**	5.785
**NR3C2**	5.785
**TUBB3**	3.351
**HDAC1**	3.351
**CYP3A5**	3.351
**EHHADH**	3.351
**CHRM3**	3.351
**CHRM1**	3.351
**TUBA8**	3.351
**ATP4A**	3.351
**ATP12A**	3.351
**TUBB6**	3.351
**CYP3A43**	3.351
**TUBB8**	3.351
**CHRNA2**	3.351
**FKBP1B**	3.351
**HTR3A**	3.351
**ADA**	3.351
**CHRM2**	3.351
**TOP2B**	3.351
**CACNB1**	2.608
**CACNA2D1**	2.608
**PLAT**	2.608
**CACNA1H**	2.608
**SCN1A**	2.608
**PDE4A**	2.608
**NDRG1**	2.608
**MAPK1**	2.608
**SNX6**	2.608
**PRDX5**	2.608
**CACNA1F**	2.608
**GABRD**	2.608
**ESR2**	2.608
**FGF1**	2.608
**PRLR**	2.608
**CACNA1A**	2.608
**TNFRSF1B**	2.608
**CFL1**	2.608
**FXYD1**	2.608
**HGS**	2.608
**CACNA1I**	2.608
**NFKB2**	2.608
**CACNA2D2**	2.608
**SERPINC1**	2.608

**TNF**	2.608
**HSD3B2**	2.608
**SHBG**	2.608
**PTGER1**	2.608
**MMP9**	2.608
**CACNA1S**	2.608
**MAPK3**	2.608
**FGG**	2.608
**PTGER4**	2.608
**PDE4D**	2.608
**TRADD**	2.608
**GABRP**	2.608
**TPT1**	2.608
**CACNA2D3**	2.608
**NEU1**	2.608
**PLA2G4A**	2.608
**CACNB4**	2.608
**GTF2F2**	2.608
**F10**	2.608
**CACNA1B**	2.608
**SMAD2**	2.608
**CACNA1C**	2.608
**IFNG**	2.608
**EGF**	2.608
**CACNA1D**	2.608
**HSD3B1**	2.608
**CACNB3**	2.608
**CA4**	2.608
**FGF4**	2.608
**FGB**	2.608
**GABRQ**	2.608
**PDE4C**	2.608
**PTGER2**	2.608
**FASN**	2.608
**EZH2**	2.608
**PTGER3**	2.608
**CES1**	2.608
**CYP11B2**	2.608
**CACNA1G**	2.608
**FXYD7**	2.608
**CLCN2**	2.608
**IKBKB**	2.608
**NEU2**	2.608
**FGA**	2.608

**PDE4B**	2.608
**ADD1**	2.608
**CACNB2**	2.608
**TNFRSF1A**	2.608
**CA2**	2.608
**VDR**	2.608
**EGFR**	2.608
**CYP27B1**	2.608
**CACNG1**	2.608
**CERS1**	2.394
**CERS2**	2.394
**CERS3**	2.394
**CERS5**	2.394
**CERS4**	2.394
**CERS6**	2.394
**ZNF471**	2.394
**THOC5**	2.394
**RECK**	2.394
**CCNO**	2.394
**ZNF99**	2.394
**SPSB1**	2.394
**KRTAP10-11**	2.394
**CREBBP**	2.394
**ZC3H15**	2.394
**CCDC117**	2.394
**ATP13A3**	2.394
**DNM2**	2.394
**GBP2**	2.394
**BFSP2**	2.394
**COG3**	2.394
**RAB33A**	2.394
**LSR**	2.394
**PPM1K**	2.394
**RAB19**	2.394
**HMBOX1**	2.394
**NRAS**	2.394
**RAB41**	2.394
**ARMC2**	2.394
**SLC6A3**	2.394
**TFAP2C**	2.394
**ABCC2**	2.394
**DCT**	2.394
**PNKD**	2.394
**EPHA2**	2.394

**TBC1D10C**	2.394
**C17orf51**	2.394
**FDX1L**	2.394
**KRTAP4-3**	2.394
**FBP1**	2.394
**MPV17**	2.394
**SLAMF6**	2.394
**UAP1L1**	2.394
**KRTAP8-1**	2.394
**LOC101060321**	2.394
**VASH2**	2.394
**SLC25A33**	2.394
**NMT1**	2.394
**LRRC23**	2.394
**MTCH2**	2.394
**CEACAM19**	2.394
**ZNF366**	2.394
**C1orf137**	2.394
**C5orf66**	2.394
**MYL6B**	2.394
**FBN3**	2.394
**ANGEL2**	2.394
**TMEM179B**	2.394

Table S13. The top 20 pathways

GO	Description
hsa04080	Neuroactive ligand-receptor interaction
hsa04540	Gap junction
hsa04010	MAPK signaling pathway
has04024	cAMP signaling pathway
hsa05200	Pathways in cancer
hsa04020	Calcium signaling pathway
has04071	Sphingolipid signaling pathway
has04728	Dopaminergic synapse
hsa04721	Synaptic vesicle cycle
hsa00140	Steroid hormone biosynthesis
hsa00310	Lysine degradation
hsa04611	Platelet activation

hsa05014	Amyotrophic lateral sclerosis (ALS)
hsa05169	Epstein-Barr virus infection
hsa00052	Galactose metabolism
hsa04750	inflammatory mediator regulation of trp channels
hsa00260	Glycine, serine and threonine metabolism
hsa04060	Cytokine-cytokine receptor interaction
hsa04514	Cell adhesion molecules (CAMs)
has05204	Chemical carcinogenesis
