# Supplementary material for: Periplocymarin Alleviates Doxorubicin-Induced Heart Failure and Excessive Accumulation of Ceramides
Source: Front Cardiovasc Med. 2021 Nov 19;8:732554. doi: 10.3389/fcvm.2021.732554 (PMC8639694; doi:10.3389/fcvm.2021.732554)
Supplement: Supplementary file 2 [file Table_1.PDF]

Table S12. Heart failure related targets

Synbol	Description	Category	GIFtS	GC id	Score
FGFR1	Fibroblast Growth Factor Receptor 1	Protein Coding	55	GC08M038400	22.4955
FGFR3	Fibroblast Growth Factor Receptor 3	Protein Coding	55	GC04P001795	16.40915
IGF1R	Insulin Like Growth Factor 1 Receptor	Protein Coding	55	GC15P098648	12.82352
ERBB4	Erb-B2 Receptor Tyrosine Kinase 4	Protein Coding	55	GC02M211375	10.78865
PDGFRA	Platelet Derived Growth Factor Receptor Alpha	Protein Coding	55	GC04P054229	10.24418
PDGFRB	Platelet Derived Growth Factor Receptor Beta	Protein Coding	55	GC05M150113	7.001416
PTPN11	Protein Tyrosine Phosphatase Non-Receptor Type 11	Protein Coding	54	GC12P112418	46.39146
TP53	Tumor Protein P53	Protein Coding	54	GC17M007661	31.78357
BRAF	B-Raf Proto-Oncogene, Serine/Threonine Kinase	Protein Coding	54	GC07M140717	27.55928
RAF1	Raf-1 Proto-Oncogene, Serine/Threonine Kinase	Protein Coding	54	GC03M012583	27.14171
MAP2K1	Mitogen-Activated Protein Kinase Kinase 1	Protein Coding	54	GC15P066386	26.50997
CHEK2	Checkpoint Kinase 2	Protein Coding	54	GC22M028687	26.27215
JAK2	Janus Kinase 2	Protein Coding	54	GC09P004985	26.0323
MAP2K2	Mitogen-Activated Protein Kinase Kinase 2	Protein Coding	54	GC19M004090	24.95358
MMP2	Matrix Metallopeptidase 2	Protein Coding	54	GC16P055390	23.46244
RET	Ret Proto-Oncogene	Protein Coding	54	GC10P043081	22.25244
MMP9	Matrix Metallopeptidase 9	Protein Coding	54	GC20P046008	21.16925
INSR	Insulin Receptor	Protein Coding	54	GC19M007112	19.20445
TGFBR1	Transforming Growth Factor Beta Receptor 1	Protein Coding	54	GC09P099104	17.97719
CTNNB1	Catenin Beta 1	Protein Coding	54	GC03P041236	17.78444
KDR	Kinase Insert Domain Receptor	Protein Coding	54	GC04M055078	17.18671
AKT1	AKT Serine/Threonine Kinase 1	Protein Coding	54	GC14M104769	16.31845
STAT1	Signal Transducer And Activator Of Transcription 1	Protein Coding	54	GC02M190908	14.81837
ATM	ATM Serine/Threonine Kinase	Protein Coding	54	GC11P108222	13.31563
EGFR	Epidermal Growth Factor Receptor	Protein Coding	54	GC07P055019	12.56526
FGFR2	Fibroblast Growth Factor Receptor 2	Protein Coding	54	GC10M121478	12.20423
BTK	Bruton Tyrosine Kinase	Protein Coding	54	GC0XM101349	11.33111
MTOR	Mechanistic Target Of Rapamycin Kinase	Protein Coding	54	GC01M011106	11.25423
ERBB2	Erb-B2 Receptor Tyrosine Kinase 2	Protein Coding	54	GC17P039687	8.803243
AKT3	AKT Serine/Threonine Kinase 3	Protein Coding	54	GC01M243488	8.442693
PRKCD	Protein Kinase C Delta	Protein Coding	54	GC03P053156	8.322029
PPP3CA	Protein Phosphatase 3 Catalytic Subunit Alpha	Protein Coding	54	GC04M101024	8.07366
ERBB3	Erb-B2 Receptor Tyrosine Kinase 3	Protein Coding	54	GC12P056376	7.417435
EZH2	Enhancer Of Zeste 2 Polycomb Repressive Complex 2 Subunit	Protein Coding	54	GC07M148807	6.186692
AKT2	AKT Serine/Threonine Kinase 2	Protein Coding	54	GC19M040230	6.152356
JAK1	Janus Kinase 1	Protein Coding	54	GC01M064833	5.573421
MET	MET Proto-Oncogene, Receptor Tyrosine Kinase	Protein Coding	54	GC07P116672	4.501132
CDK4	Cyclin Dependent Kinase 4	Protein Coding	54	GC12M057743	3.799161
CDK5	Cyclin Dependent Kinase 5	Protein Coding	54	GC07M151053	3.683671
CDK6	Cyclin Dependent Kinase 6	Protein Coding	54	GC07M092604	3.165874
PIK3CD	Phosphatidylinositol-4,5-Bisphosphate 3-Kinase Catalytic Subunit Delta	Protein Coding	54	GC01P009629	2.723964
ABL1	ABL Proto-Oncogene 1, Non-Receptor Tyrosine Kinase	Protein Coding	53	GC09P130713	35.04947
PTEN	Phosphatase And Tensin Homolog	Protein Coding	53	GC10P087863	33.45667
PIK3CA	Phosphatidylinositol-4,5-Bisphosphate 3-Kinase Catalytic Subunit Alpha	Protein Coding	53	GC03P179148	30.45154
PPARG	Peroxisome Proliferator Activated Receptor Gamma	Protein Coding	53	GC03P012287	26.79068
ESR1	Estrogen Receptor 1	Protein Coding	53	GC06P151656	20.75763
STAT3	Signal Transducer And Activator Of Transcription 3	Protein Coding	53	GC17M042313	18.18649
CDH2	Cadherin 2	Protein Coding	53	GC18M027950	17.67587

HMOX1	Heme Oxygenase 1	Protein Coding	53	GC22P035380	16.02464
MYLK	Myosin Light Chain Kinase	Protein Coding	53	GC03M123610	14.17601
RAD51	RAD51 Recombinase	Protein Coding	53	GC15P040694	12.3909
KIT	KIT Proto-Oncogene, Receptor Tyrosine Kinase	Protein Coding	53	GC04P054657	11.70934
MDM2	MDM2 Proto-Oncogene	Protein Coding	53	GC12P068808	11.36692
RPS6KA3	Ribosomal Protein S6 Kinase A3	Protein Coding	53	GC0XM020149	10.74417
SLC2A1	Solute Carrier Family 2 Member 1	Protein Coding	53	GC01M042925	10.13715
AR	Androgen Receptor	Protein Coding	53	GC0XP067544	9.259001
IKBKB	Inhibitor Of Nuclear Factor Kappa B Kinase Subunit Beta	Protein Coding	53	GC08P042271	7.618749
DPYD	Dihydropyrimidine Dehydrogenase	Protein Coding	53	GC01M097015	6.928517
NTRK2	Neurotrophic Receptor Tyrosine Kinase 2	Protein Coding	53	GC09P084668	6.513509
DDC	Dopa Decarboxylase	Protein Coding	53	GC07M050458	6.417831
FLT3	Fms Related Receptor Tyrosine Kinase 3	Protein Coding	53	GC13M028003	4.323861
LCK	LCK Proto-Oncogene, Src Family Tyrosine Kinase	Protein Coding	53	GC01P032251	4.190559
TYK2	Tyrosine Kinase 2	Protein Coding	53	GC19M010350	3.439712
ADAM10	ADAM Metallopeptidase Domain 10	Protein Coding	53	GC15M058588	3.360528
IDH1	Isocitrate Dehydrogenase (NADP(+)) 1	Protein Coding	53	GC02M208236	2.817001
EPHA2	EPH Receptor A2	Protein Coding	53	GC01M016124	1.615132
MAPK1	Mitogen-Activated Protein Kinase 1	Protein Coding	52	GC22M021754	39.90771
TERT	Telomerase Reverse Transcriptase	Protein Coding	52	GC05M001253	39.53186
NOS3	Nitric Oxide Synthase 3	Protein Coding	52	GC07P150990	35.06408
TGFB1	Transforming Growth Factor Beta 1	Protein Coding	52	GC19M041301	30.80153
KRAS	KRAS Proto-Oncogene, GTPase	Protein Coding	52	GC12M025204	26.32568
SOD1	Superoxide Dismutase 1	Protein Coding	52	GC21P031659	22.25884
FLT4	Fms Related Receptor Tyrosine Kinase 4	Protein Coding	52	GC05M180607	22.12309
HRAS	HRas Proto-Oncogene, GTPase	Protein Coding	52	GC11M001078	20.88017
PSEN1	Presenilin 1	Protein Coding	52	GC14P073136	20.83368
MMP1	Matrix Metallopeptidase 1	Protein Coding	52	GC11M102810	18.89547
ATP2A2	ATPase Sarcoplasmic/Endoplasmic Reticulum Ca2+ Transporting 2	Protein Coding	52	GC12P110280	17.91092
CREBBP	CREB Binding Protein	Protein Coding	52	GC16M003745	17.16587
PRKAR1A	Protein Kinase CAMP-Dependent Type I Regulatory Subunit Alpha	Protein Coding	52	GC17P068545	17.12037
COMT	Catechol-O-Methyltransferase	Protein Coding	52	GC22P019941	16.68583
CFTR	CF Transmembrane Conductance Regulator	Protein Coding	52	GC07P117287	16.04282
HGF	Hepatocyte Growth Factor	Protein Coding	52	GC07M081699	15.58496
MAPK14	Mitogen-Activated Protein Kinase 14	Protein Coding	52	GC06P055339	14.19597
TLR2	Toll Like Receptor 2	Protein Coding	52	GC04P153684	14.15205
EPHB4	EPH Receptor B4	Protein Coding	52	GC07M100803	13.662
ADA	Adenosine Deaminase	Protein Coding	52	GC20M044620	13.5733
VDR	Vitamin D Receptor	Protein Coding	52	GC12M047841	12.72744
TH	Tyrosine Hydroxylase	Protein Coding	52	GC11M002163	11.3846
CXCR4	C-X-C Motif Chemokine Receptor 4	Protein Coding	52	GC02M136114	11.30857
CDKN2A	Cyclin Dependent Kinase Inhibitor 2A	Protein Coding	52	GC09M021967	11.21965
PRKCA	Protein Kinase C Alpha	Protein Coding	52	GC17P066302	10.72806
ABCB1	ATP Binding Cassette Subfamily B Member 1	Protein Coding	52	GC07M087504	10.12535
HSPB1	Heat Shock Protein Family B (Small) Member 1	Protein Coding	52	GC07P076302	10.09462
HDAC4	Histone Deacetylase 4	Protein Coding	52	GC02M239048	9.80567
DNMT3A	DNA Methyltransferase 3 Alpha	Protein Coding	52	GC02M025228	9.677424
NFKB1	Nuclear Factor Kappa B Subunit 1	Protein Coding	52	GC04P102501	9.403999
MMP14	Matrix Metallopeptidase 14	Protein Coding	52	GC14P026302	9.156852
CASP8	Caspase 8	Protein Coding	52	GC02P201233	9.100082
CTSD	Cathepsin D	Protein Coding	52	GC11M001752	8.939178
CCND1	Cyclin D1	Protein Coding	52	GC11P069641	8.590908
TLR3	Toll Like Receptor 3	Protein Coding	52	GC04P186059	7.963427

ADK	Adenosine Kinase	Protein Coding	52	GC10P074152	7.797109
PRKACA	Protein Kinase CAMP-Activated Catalytic Subunit Alpha	Protein Coding	52	GC19M014129	7.682183
ALK	ALK Receptor Tyrosine Kinase	Protein Coding	52	GC02M029190	7.500273
JAK3	Janus Kinase 3	Protein Coding	52	GC19M017824	7.072352
SRC	SRC Proto-Oncogene, Non-Receptor Tyrosine Kinase	Protein Coding	52	GC20P037344	6.85365
NTRK3	Neurotrophic Receptor Tyrosine Kinase 3	Protein Coding	52	GC15M087859	6.431217
CSNK2A1	Casein Kinase 2 Alpha 1	Protein Coding	52	GC20M000472	6.338788
APP	Amyloid Beta Precursor Protein	Protein Coding	52	GC21M025880	6.189873
NFKB2	Nuclear Factor Kappa B Subunit 2	Protein Coding	52	GC10P102394	6.169051
IDH2	Isocitrate Dehydrogenase (NADP(+)) 2	Protein Coding	52	GC15M090083	6.148547
CA2	Carbonic Anhydrase 2	Protein Coding	52	GC08P085463	5.491413
MAPK10	Mitogen-Activated Protein Kinase 10	Protein Coding	52	GC04M085990	5.330097
PLAU	Plasminogen Activator, Urokinase	Protein Coding	52	GC10P073909	5.284805
BCR	BCR Activator Of RhoGEF And GTPase	Protein Coding	52	GC22P023179	5.274552
AXL	AXL Receptor Tyrosine Kinase	Protein Coding	52	GC19P041219	5.01234
NT5E	5'-Nucleotidase Ecto	Protein Coding	52	GC06P085449	4.824818
PCNA	Proliferating Cell Nuclear Antigen	Protein Coding	52	GC20M005114	4.819314
LDHA	Lactate Dehydrogenase A	Protein Coding	52	GC11P018394	4.74956
GAD1	Glutamate Decarboxylase 1	Protein Coding	52	GC02P170813	4.68924
HDAC2	Histone Deacetylase 2	Protein Coding	52	GC06M113933	4.474967
CHUK	Component Of Inhibitor Of Nuclear Factor Kappa B Kinase Complex	Protein Coding	52	GC10M100188	4.456046
ATR	ATR Serine/Threonine Kinase	Protein Coding	52	GC03M142449	4.219328
ZAP70	Zeta Chain Of T Cell Receptor Associated Protein Kinase 70	Protein Coding	52	GC02P097696	4.032827
CDK2	Cyclin Dependent Kinase 2	Protein Coding	52	GC12P055966	3.798081
GRIN2A	Glutamate Ionotropic Receptor NMDA Type Subunit 2A	Protein Coding	52	GC16M009753	3.509576
HDAC6	Histone Deacetylase 6	Protein Coding	52	GC0XP048801	3.478621
ITK	IL2 Inducible T Cell Kinase	Protein Coding	52	GC05P157158	3.209816
UCHL1	Ubiquitin C-Terminal Hydrolase L1	Protein Coding	52	GC04P041256	2.828598
CSF1R	Colony Stimulating Factor 1 Receptor	Protein Coding	52	GC05M150053	2.757461
FGFR4	Fibroblast Growth Factor Receptor 4	Protein Coding	52	GC05P177086	2.646095
PLCG2	Phospholipase C Gamma 2	Protein Coding	52	GC16P081773	2.393744
PRKCG	Protein Kinase C Gamma	Protein Coding	52	GC19P053879	2.248861
AURKA	Aurora Kinase A	Protein Coding	52	GC20M056370	1.958206
EPHB2	EPH Receptor B2	Protein Coding	52	GC01P022710	1.528136
SCN5A	Sodium Voltage-Gated Channel Alpha Subunit 5	Protein Coding	51	GC03M038549	83.24096
PRKAG2	Protein Kinase AMP-Activated Non-Catalytic Subunit Gamma 2	Protein Coding	51	GC07M151556	56.95018
GJA1	Gap Junction Protein Alpha 1	Protein Coding	51	GC06P121436	56.23439
JAG1	Jagged Canonical Notch Ligand 1	Protein Coding	51	GC20M010637	56.15142
TNF	Tumor Necrosis Factor	Protein Coding	51	GC06P055202	36.75134
AGTR1	Angiotensin II Receptor Type 1	Protein Coding	51	GC03P148697	36.29478
NOTCH1	Notch Receptor 1	Protein Coding	51	GC09M136705	32.77985
APOE	Apolipoprotein E	Protein Coding	51	GC19P044906	29.49453
TEK	TEK Receptor Tyrosine Kinase	Protein Coding	51	GC09P027109	27.72603
NR5A1	Nuclear Receptor Subfamily 5 Group A Member 1	Protein Coding	51	GC09M124481	27.25827
SMAD4	SMAD Family Member 4	Protein Coding	51	GC18P051028	24.43342
SERPINE1	Serpin Family E Member 1	Protein Coding	51	GC07P101127	22.65792
MMP3	Matrix Metallopeptidase 3	Protein Coding	51	GC11M102835	21.44748
PTCH1	Patched 1	Protein Coding	51	GC09M095442	20.95475
TGFBR2	Transforming Growth Factor Beta Receptor 2	Protein Coding	51	GC03P030623	19.80687
TLR4	Toll Like Receptor 4	Protein Coding	51	GC09P117704	19.80662
PRKD1	Protein Kinase D1	Protein Coding	51	GC14M029576	19.54486
ICAM1	Intercellular Adhesion Molecule 1	Protein Coding	51	GC19P010270	18.61091
FOS	Fos Proto-Oncogene, AP-1 Transcription Factor Subunit	Protein Coding	51	GC14P075278	18.51714

BMPR1A	Bone Morphogenetic Protein Receptor Type 1A	Protein Coding	51	GC10P086756	18.4046
EP300	E1A Binding Protein P300	Protein Coding	51	GC22P041091	17.88151
ALDH2	Aldehyde Dehydrogenase 2 Family Member	Protein Coding	51	GC12P111766	17.746
MPO	Myeloperoxidase	Protein Coding	51	GC17M058269	17.58922
PCSK9	Proprotein Convertase Subtilisin/Kexin Type 9	Protein Coding	51	GC01P055039	17.18996
CASP3	Caspase 3	Protein Coding	51	GC04M184627	16.48058
CASR	Calcium Sensing Receptor	Protein Coding	51	GC03P122183	16.06041
FLT1	Fms Related Receptor Tyrosine Kinase 1	Protein Coding	51	GC13M028300	15.80145
FAS	Fas Cell Surface Death Receptor	Protein Coding	51	GC10P088969	15.60325
GNAS	GNAS Complex Locus	Protein Coding	51	GC20P058839	15.55873
COL1A1	Collagen Type I Alpha 1 Chain	Protein Coding	51	GC17M050183	15.28597
MME	Membrane Metalloendopeptidase	Protein Coding	51	GC03P155024	15.21592
BRCA1	BRCA1 DNA Repair Associated	Protein Coding	51	GC17M043044	14.78573
MYC	MYC Proto-Oncogene, BHLH Transcription Factor	Protein Coding	51	GC08P127735	14.59934
G6PD	Glucose-6-Phosphate Dehydrogenase	Protein Coding	51	GC0XM154531	13.74756
SLC9A1	Solute Carrier Family 9 Member A1	Protein Coding	51	GC01M027109	12.68094
GCK	Glucokinase	Protein Coding	51	GC07M044154	12.67608
SOD2	Superoxide Dismutase 2	Protein Coding	51	GC06M159669	12.57527
ITGA2B	Integrin Subunit Alpha 2b	Protein Coding	51	GC17M044442	12.1962
NR3C1	Nuclear Receptor Subfamily 3 Group C Member 1	Protein Coding	51	GC05M143277	12.15809
IL2RA	Interleukin 2 Receptor Subunit Alpha	Protein Coding	51	GC10M006010	12.11188
DBH	Dopamine Beta-Hydroxylase	Protein Coding	51	GC09P133636	12.09029
CYP3A4	Cytochrome P450 Family 3 Subfamily A Member 4	Protein Coding	51	GC07M099759	12.04126
DYRK1A	Dual Specificity Tyrosine Phosphorylation Regulated Kinase 1A	Protein Coding	51	GC21P037365	11.60606
CBL	Cbl Proto-Oncogene	Protein Coding	51	GC11P119206	11.52502
PRKG1	Protein Kinase CGMP-Dependent 1	Protein Coding	51	GC10P050991	11.40086
CALR	Calreticulin	Protein Coding	51	GC19P012938	10.72083
PTPRC	Protein Tyrosine Phosphatase Receptor Type C	Protein Coding	51	GC01P198607	10.53737
EGF	Epidermal Growth Factor	Protein Coding	51	GC04P109912	10.51334
MAP3K7	Mitogen-Activated Protein Kinase Kinase Kinase 7	Protein Coding	51	GC06M090513	10.47973
DLD	Dihydrolipoamide Dehydrogenase	Protein Coding	51	GC07P107890	10.47635
RARB	Retinoic Acid Receptor Beta	Protein Coding	51	GC03P024830	10.24688
DNMT3B	DNA Methyltransferase 3 Beta	Protein Coding	51	GC20P032762	9.952049
BCL2	BCL2 Apoptosis Regulator	Protein Coding	51	GC18M063123	9.724048
ALPL	Alkaline Phosphatase, Biomineralization Associated	Protein Coding	51	GC01P021508	9.690216
BLK	BLK Proto-Oncogene, Src Family Tyrosine Kinase	Protein Coding	51	GC08P011486	9.156414
VIM	Vimentin	Protein Coding	51	GC10P017227	9.095276
ADAM17	ADAM Metallopeptidase Domain 17	Protein Coding	51	GC02M009488	9.094537
BMPR1B	Bone Morphogenetic Protein Receptor Type 1B	Protein Coding	51	GC04P094757	8.782127
MAPK8	Mitogen-Activated Protein Kinase 8	Protein Coding	51	GC10P048306	8.344939
ACVR1	Activin A Receptor Type 1	Protein Coding	51	GC02M157736	8.327995
CASP1	Caspase 1	Protein Coding	51	GC11M105025	8.118779
HNF4A	Hepatocyte Nuclear Factor 4 Alpha	Protein Coding	51	GC20P044355	8.051538
TACR3	Tachykinin Receptor 3	Protein Coding	51	GC04M103586	7.989038
MAPT	Microtubule Associated Protein Tau	Protein Coding	51	GC17P045894	7.98013
ITGB1	Integrin Subunit Beta 1	Protein Coding	51	GC10M032890	7.842117
PGR	Progesterone Receptor	Protein Coding	51	GC11M101030	7.758314
ACVR2B	Activin A Receptor Type 2B	Protein Coding	51	GC03P038453	7.720028
DRD2	Dopamine Receptor D2	Protein Coding	51	GC11M113409	7.588826
PSMB8	Proteasome 20S Subunit Beta 8	Protein Coding	51	GC06M032840	7.546425
GRIN2B	Glutamate Ionotropic Receptor NMDA Type Subunit 2B	Protein Coding	51	GC12M013437	7.528903
AHCY	Adenosylhomocysteinase	Protein Coding	51	GC20M034341	7.458994
FTH1	Ferritin Heavy Chain 1	Protein Coding	51	GC11M061959	7.288177

SPARC	Secreted Protein Acidic And Cysteine Rich	Protein Coding	51	GC05M151661	7.222661
ACTN1	Actinin Alpha 1	Protein Coding	51	GC14M068874	6.961054
PFKM	Phosphofructokinase, Muscle	Protein Coding	51	GC12P048105	6.859891
CDC42	Cell Division Cycle 42	Protein Coding	51	GC01P022112	6.662259
PRKAA2	Protein Kinase AMP-Activated Catalytic Subunit Alpha 2	Protein Coding	51	GC01P056645	6.638632
NGF	Nerve Growth Factor	Protein Coding	51	GC01M115285	6.597648
FZD4	Frizzled Class Receptor 4	Protein Coding	51	GC11M086945	6.401958
ATP1A1	ATPase Na+/K+ Transporting Subunit Alpha 1	Protein Coding	51	GC01P116372	6.368464
THRB	Thyroid Hormone Receptor Beta	Protein Coding	51	GC03M024117	6.270998
GSTP1	Glutathione S-Transferase Pi 1	Protein Coding	51	GC11P067583	6.16314
PIK3R1	Phosphoinositide-3-Kinase Regulatory Subunit 1	Protein Coding	51	GC05P068215	6.034737
CACNA1H	Calcium Voltage-Gated Channel Subunit Alpha1 H	Protein Coding	51	GC16P001153	5.933612
PTPN1	Protein Tyrosine Phosphatase Non-Receptor Type 1	Protein Coding	51	GC20P050510	5.9299
CTSB	Cathepsin B	Protein Coding	51	GC08M011842	5.917904
MAOA	Monoamine Oxidase A	Protein Coding	51	GC0XP043654	5.781933
SNAP25	Synaptosome Associated Protein 25	Protein Coding	51	GC20P010218	5.703187
PRKCH	Protein Kinase C Eta	Protein Coding	51	GC14P061187	5.662296
NFKBIA	NFKB Inhibitor Alpha	Protein Coding	51	GC14M035401	5.558657
DNMT1	DNA Methyltransferase 1	Protein Coding	51	GC19M010133	5.408778
MMP13	Matrix Metallopeptidase 13	Protein Coding	51	GC11M102942	5.29451
ROCK1	Rho Associated Coiled-Coil Containing Protein Kinase 1	Protein Coding	51	GC18M020946	5.250692
GSK3B	Glycogen Synthase Kinase 3 Beta	Protein Coding	51	GC03M119821	5.125985
RAC2	Rac Family Small GTPase 2	Protein Coding	51	GC22M037227	5.051332
DDR2	Discoidin Domain Receptor Tyrosine Kinase 2	Protein Coding	51	GC01P162631	4.863748
RXRA	Retinoid X Receptor Alpha	Protein Coding	51	GC09P134317	4.599022
ABCG2	ATP Binding Cassette Subfamily G Member 2 (Junior Blood Group)	Protein Coding	51	GC04M088090	4.549934
CDH1	Cadherin 1	Protein Coding	51	GC16P068737	4.409263
DNM1	Dynamin 1	Protein Coding	51	GC09P128191	4.282455
MSH6	MutS Homolog 6	Protein Coding	51	GC02P047695	4.26857
RELA	RELA Proto-Oncogene, NF-KB Subunit	Protein Coding	51	GC11M065653	4.259816
MERTK	MER Proto-Oncogene, Tyrosine Kinase	Protein Coding	51	GC02P111898	3.982695
GRIA2	Glutamate Ionotropic Receptor AMPA Type Subunit 2	Protein Coding	51	GC04P157204	3.949049
GRM1	Glutamate Metabotropic Receptor 1	Protein Coding	51	GC06P145973	3.827963
SYK	Spleen Associated Tyrosine Kinase	Protein Coding	51	GC09P091373	3.770406
PIM1	Pim-1 Proto-Oncogene, Serine/Threonine Kinase	Protein Coding	51	GC06P055356	3.59666
GRIA3	Glutamate Ionotropic Receptor AMPA Type Subunit 3	Protein Coding	51	GC0XP123184	3.523802
SLC1A3	Solute Carrier Family 1 Member 3	Protein Coding	51	GC05P036620	3.492232
CAD	Carbamoyl-Phosphate Synthetase 2, Aspartate Transcarbamylase, And Dihydroorotase	Protein Coding	51	GC02P027217	3.443655
ITGB2	Integrin Subunit Beta 2	Protein Coding	51	GC21M044885	3.398953
TOP2A	DNA Topoisomerase II Alpha	Protein Coding	51	GC17M040388	3.34852
RPS6KA1	Ribosomal Protein S6 Kinase A1	Protein Coding	51	GC01P026540	3.044156
TBXAS1	Thromboxane A Synthase 1	Protein Coding	51	GC07P139777	3.036754
HK1	Hexokinase 1	Protein Coding	51	GC10P069269	2.877566
MYB	MYB Proto-Oncogene, Transcription Factor	Protein Coding	51	GC06P135180	2.876369
SPR	Sepiapterin Reductase	Protein Coding	51	GC02P072850	2.830243
GRIN1	Glutamate Ionotropic Receptor NMDA Type Subunit 1	Protein Coding	51	GC09P137138	2.765989
RARA	Retinoic Acid Receptor Alpha	Protein Coding	51	GC17P040309	2.676082
NEK2	NIMA Related Kinase 2	Protein Coding	51	GC01M211658	2.630929
TNFRSF10B	TNF Receptor Superfamily Member 10b	Protein Coding	51	GC08M023020	2.44852
CSNK1D	Casein Kinase 1 Delta	Protein Coding	51	GC17M082239	2.130938
PAK3	P21 (RAC1) Activated Kinase 3	Protein Coding	51	GC0XP110944	1.861871
PTPRF	Protein Tyrosine Phosphatase Receptor Type F	Protein Coding	51	GC01P043527	1.370426
ACE	Angiotensin I Converting Enzyme	Protein Coding	50	GC17P063477	61.05265

TNNT2	Troponin T2, Cardiac Type	Protein Coding	50	GC01M201359	52.35754
DSP	Desmoplakin	Protein Coding	50	GC06P007541	50.04069
TNNI3	Troponin I3, Cardiac Type	Protein Coding	50	GC19M055151	48.95369
KCNQ1	Potassium Voltage-Gated Channel Subfamily Q Member 1	Protein Coding	50	GC11P002444	47.39628
IL6	Interleukin 6	Protein Coding	50	GC07P022725	44.94719
TTR	Transthyretin	Protein Coding	50	GC18P031557	39.79792
AGT	Angiotensinogen	Protein Coding	50	GC01M230702	36.36314
ALB	Albumin	Protein Coding	50	GC04P073397	34.37361
BMPR2	Bone Morphogenetic Protein Receptor Type 2	Protein Coding	50	GC02P202376	33.88158
KCNH2	Potassium Voltage-Gated Channel Subfamily H Member 2	Protein Coding	50	GC07M150944	33.55603
TPM1	Tropomyosin 1	Protein Coding	50	GC15P078910	32.83193
CACNA1C	Calcium Voltage-Gated Channel Subunit Alpha1 C	Protein Coding	50	GC12P001970	32.75956
FLNA	Filamin A	Protein Coding	50	GC0XM154348	29.97549
MYL2	Myosin Light Chain 2	Protein Coding	50	GC12M110910	28.4017
NR2F2	Nuclear Receptor Subfamily 2 Group F Member 2	Protein Coding	50	GC15P096325	26.62963
TSC2	TSC Complex Subunit 2	Protein Coding	50	GC16P005579	26.61515
ADRB2	Adrenoceptor Beta 2	Protein Coding	50	GC05P148825	26.50452
IGF1	Insulin Like Growth Factor 1	Protein Coding	50	GC12M102395	26.34979
BRCA2	BRCA2 DNA Repair Associated	Protein Coding	50	GC13P032315	26.23956
GNAQ	G Protein Subunit Alpha Q	Protein Coding	50	GC09M077716	25.80118
TGFB2	Transforming Growth Factor Beta 2	Protein Coding	50	GC01P218345	24.91702
LDLR	Low Density Lipoprotein Receptor	Protein Coding	50	GC19P011091	24.13878
ITGB3	Integrin Subunit Beta 3	Protein Coding	50	GC17P047254	23.31316
CPT2	Carnitine Palmitoyltransferase 2	Protein Coding	50	GC01P053196	22.95444
WT1	WT1 Transcription Factor	Protein Coding	50	GC11M032365	22.49163
FN1	Fibronectin 1	Protein Coding	50	GC02M215360	22.40897
TNFRSF1A	TNF Receptor Superfamily Member 1A	Protein Coding	50	GC12M006328	22.29749
LPL	Lipoprotein Lipase	Protein Coding	50	GC08P019901	22.12784
EDNRA	Endothelin Receptor Type A	Protein Coding	50	GC04P147480	19.94889
TF	Transferrin	Protein Coding	50	GC03P133666	19.70341
ACVRL1	Activin A Receptor Like Type 1	Protein Coding	50	GC12P051906	19.58186
PTH1R	Parathyroid Hormone 1 Receptor	Protein Coding	50	GC03P046877	18.97197
SMARCA4	SWI/SNF Related, Matrix Associated, Actin Dependent Regulator Of Chromatin, Subfamily A, Member 4	Protein Coding	50	GC19P010932	18.62375
GATA3	GATA Binding Protein 3	Protein Coding	50	GC10P008045	18.13439
BMP4	Bone Morphogenetic Protein 4	Protein Coding	50	GC14M053949	17.9759
NOS2	Nitric Oxide Synthase 2	Protein Coding	50	GC17M027756	17.46309
PSEN2	Presenilin 2	Protein Coding	50	GC01P226870	17.45359
SERPINA1	Serpin Family A Member 1	Protein Coding	50	GC14M094376	17.3331
SHH	Sonic Hedgehog Signaling Molecule	Protein Coding	50	GC07M155799	17.23312
NOTCH2	Notch Receptor 2	Protein Coding	50	GC01M119911	17.23034
SLC25A4	Solute Carrier Family 25 Member 4	Protein Coding	50	GC04P185143	17.1219
MEF2C	Myocyte Enhancer Factor 2C	Protein Coding	50	GC05M088718	16.97543
GLI3	GLI Family Zinc Finger 3	Protein Coding	50	GC07M041960	16.03291
NF1	Neurofibromin 1	Protein Coding	50	GC17P031094	15.97479
PLAT	Plasminogen Activator, Tissue Type	Protein Coding	50	GC08M042174	15.93711
KCNK3	Potassium Two Pore Domain Channel Subfamily K Member 3	Protein Coding	50	GC02P026692	15.75677
EDNRB	Endothelin Receptor Type B	Protein Coding	50	GC13M077895	15.61403
CYP2C9	Cytochrome P450 Family 2 Subfamily C Member 9	Protein Coding	50	GC10P094938	15.05969
SMAD3	SMAD Family Member 3	Protein Coding	50	GC15P067063	14.91825
NRAS	NRAS Proto-Oncogene, GTPase	Protein Coding	50	GC01M114704	14.53802
CAMK2D	Calcium/Calmodulin Dependent Protein Kinase II Delta	Protein Coding	50	GC04M113452	13.87698
CD4	CD4 Molecule	Protein Coding	50	GC12P006786	13.81976
ACTB	Actin Beta	Protein Coding	50	GC07M005527	13.34085

CYP17A1	Cytochrome P450 Family 17 Subfamily A Member 1	Protein Coding	50	GC10M102830	12.8083
LEPR	Leptin Receptor	Protein Coding	50	GC01P065421	12.29724
CYP11A1	Cytochrome P450 Family 11 Subfamily A Member 1	Protein Coding	50	GC15M074337	11.85308
RRM2B	Ribonucleotide Reductase Regulatory TP53 Inducible Subunit M2B	Protein Coding	50	GC08M102204	11.81024
NFE2L2	Nuclear Factor, Erythroid 2 Like 2	Protein Coding	50	GC02M177227	11.79462
VCP	Valosin Containing Protein	Protein Coding	50	GC09M035056	11.7334
TRPV4	Transient Receptor Potential Cation Channel Subfamily V Member 4	Protein Coding	50	GC12M109783	11.70096
GYS1	Glycogen Synthase 1	Protein Coding	50	GC19M048970	11.68141
MYD88	MYD88 Innate Immune Signal Transduction Adaptor	Protein Coding	50	GC03P038139	11.61078
CYP19A1	Cytochrome P450 Family 19 Subfamily A Member 1	Protein Coding	50	GC15M051208	11.54159
LIMK1	LIM Domain Kinase 1	Protein Coding	50	GC07P074082	11.44395
PARP1	Poly(ADP-Ribose) Polymerase 1	Protein Coding	50	GC01M226360	11.29187
GSR	Glutathione-Disulfide Reductase	Protein Coding	50	GC08M030678	11.22714
CAT	Catalase	Protein Coding	50	GC11P034460	10.8495
MVK	Mevalonate Kinase	Protein Coding	50	GC12P109573	10.65042
NOS1	Nitric Oxide Synthase 1	Protein Coding	50	GC12M117208	10.61025
PDE3A	Phosphodiesterase 3A	Protein Coding	50	GC12P020294	10.59413
MYH9	Myosin Heavy Chain 9	Protein Coding	50	GC22M036281	10.4504
FSHR	Follicle Stimulating Hormone Receptor	Protein Coding	50	GC02M048953	10.25581
PLA2G7	Phospholipase A2 Group VII	Protein Coding	50	GC06M047382	10.11653
JUN	Jun Proto-Oncogene, AP-1 Transcription Factor Subunit	Protein Coding	50	GC01M058780	10.07837
EPAS1	Endothelial PAS Domain Protein 1	Protein Coding	50	GC02P046293	10.00135
IL6R	Interleukin 6 Receptor	Protein Coding	50	GC01P154405	9.855521
SNCA	Synuclein Alpha	Protein Coding	50	GC04M089724	9.806259
EIF2AK3	Eukaryotic Translation Initiation Factor 2 Alpha Kinase 3	Protein Coding	50	GC02M088556	9.658109
WNT5A	Wnt Family Member 5A	Protein Coding	50	GC03M055465	9.52868
CPT1A	Carnitine Palmitoyltransferase 1A	Protein Coding	50	GC11M068754	9.356894
NOTCH3	Notch Receptor 3	Protein Coding	50	GC19M015159	9.24761
CD19	CD19 Molecule	Protein Coding	50	GC16P032270	9.125484
ACTG1	Actin Gamma 1	Protein Coding	50	GC17M081509	9.107426
SMARCA2	SWI/SNF Related, Matrix Associated, Actin Dependent Regulator Of Chromatin, Subfamily A, Member 2	Protein Coding	50	GC09P001980	9.00527
PBX1	PBX Homeobox 1	Protein Coding	50	GC01P164524	8.994007
ALOX5	Arachidonate 5-Lipoxygenase	Protein Coding	50	GC10P045374	8.989513
CDKN1A	Cyclin Dependent Kinase Inhibitor 1A	Protein Coding	50	GC06P055348	8.843244
F10	Coagulation Factor X	Protein Coding	50	GC13P113122	8.825261
RAC1	Rac Family Small GTPase 1	Protein Coding	50	GC07P006377	8.736277
POR	Cytochrome P450 Oxidoreductase	Protein Coding	50	GC07P075899	8.706636
SIRT1	Sirtuin 1	Protein Coding	50	GC10P067884	8.643274
SLC2A2	Solute Carrier Family 2 Member 2	Protein Coding	50	GC03M170996	8.561977
CHRM3	Cholinergic Receptor Muscarinic 3	Protein Coding	50	GC01P239386	8.439431
DPP4	Dipeptidyl Peptidase 4	Protein Coding	50	GC02M161992	8.391326
LRRK2	Leucine Rich Repeat Kinase 2	Protein Coding	50	GC12P040196	8.303381
DNM2	Dynamin 2	Protein Coding	50	GC19P010718	8.228769
IRAK1	Interleukin 1 Receptor Associated Kinase 1	Protein Coding	50	GC0XM154010	8.077294
MAPK3	Mitogen-Activated Protein Kinase 3	Protein Coding	50	GC16M031188	7.933195
XIAP	X-Linked Inhibitor Of Apoptosis	Protein Coding	50	GC0XP123859	7.908162
MIF	Macrophage Migration Inhibitory Factor	Protein Coding	50	GC22P023894	7.837247
PDGFB	Platelet Derived Growth Factor Subunit B	Protein Coding	50	GC22M049027	7.833078
PDHA1	Pyruvate Dehydrogenase E1 Subunit Alpha 1	Protein Coding	50	GC0XP019343	7.740581
TUBB	Tubulin Beta Class I	Protein Coding	50	GC06P055181	7.734519
DHFR	Dihydrofolate Reductase	Protein Coding	50	GC05M080626	7.682227
YWHAE	Tyrosine 3-Monooxygenase/Tryptophan 5-Monooxygenase Activation Protein Epsilon	Protein Coding	50	GC17M001346	7.624144
GLUL	Glutamate-Ammonia Ligase	Protein Coding	50	GC01M182378	7.539698

DUSP6	Dual Specificity Phosphatase 6	Protein Coding	50	GC12M089347	7.417614
PRKCE	Protein Kinase C Epsilon	Protein Coding	50	GC02P045651	7.339571
PRDX1	Peroxiredoxin 1	Protein Coding	50	GC01M045511	7.329257
KCNMA1	Potassium Calcium-Activated Channel Subfamily M Alpha 1	Protein Coding	50	GC10M076869	7.178487
CYP11B1	Cytochrome P450 Family 11 Subfamily B Member 1	Protein Coding	50	GC08M142872	7.04149
PIK3R2	Phosphoinositide-3-Kinase Regulatory Subunit 2	Protein Coding	50	GC19P018153	6.941929
ESR2	Estrogen Receptor 2	Protein Coding	50	GC14M064084	6.893557
EXT1	Exostosin Glycosyltransferase 1	Protein Coding	50	GC08M117798	6.859998
P4HB	Prolyl 4-Hydroxylase Subunit Beta	Protein Coding	50	GC17M081843	6.808558
CREB1	CAMP Responsive Element Binding Protein 1	Protein Coding	50	GC02P207529	6.803981
PHGDH	Phosphoglycerate Dehydrogenase	Protein Coding	50	GC01P119660	6.777925
STK11	Serine/Threonine Kinase 11	Protein Coding	50	GC19P001177	6.769823
TBK1	TANK Binding Kinase 1	Protein Coding	50	GC12P064451	6.727894
SLC1A2	Solute Carrier Family 1 Member 2	Protein Coding	50	GC11M035252	6.686244
NPM1	Nucleophosmin 1	Protein Coding	50	GC05P171387	6.584521
CACNA1B	Calcium Voltage-Gated Channel Subunit Alpha1 B	Protein Coding	50	GC09P137877	6.520741
CASP7	Caspase 7	Protein Coding	50	GC10P113679	6.478548
ANPEP	Alanyl Aminopeptidase, Membrane	Protein Coding	50	GC15M089784	6.425012
BLM	BLM RecQ Like Helicase	Protein Coding	50	GC15P090717	6.312682
SMO	Smoothened, Frizzled Class Receptor	Protein Coding	50	GC07P130058	6.308393
BUB1	BUB1 Mitotic Checkpoint Serine/Threonine Kinase	Protein Coding	50	GC02M110637	6.268031
TNC	Tenascin C	Protein Coding	50	GC09M115019	6.100604
CTSK	Cathepsin K	Protein Coding	50	GC01M150837	6.008756
NTRK1	Neurotrophic Receptor Tyrosine Kinase 1	Protein Coding	50	GC01P156815	5.98376
THRA	Thyroid Hormone Receptor Alpha	Protein Coding	50	GC17P040058	5.863861
STAT5B	Signal Transducer And Activator Of Transcription 5B	Protein Coding	50	GC17M042199	5.80737
SLC6A3	Solute Carrier Family 6 Member 3	Protein Coding	50	GC05M001392	5.757867
SGK1	Serum/Glucocorticoid Regulated Kinase 1	Protein Coding	50	GC06M134169	5.754192
SLC5A1	Solute Carrier Family 5 Member 1	Protein Coding	50	GC22P032043	5.660302
BUB1B	BUB1 Mitotic Checkpoint Serine/Threonine Kinase B	Protein Coding	50	GC15P040161	5.626396
HSD11B1	Hydroxysteroid 11-Beta Dehydrogenase 1	Protein Coding	50	GC01P209686	5.608378
PROC	Protein C, Inactivator Of Coagulation Factors Va And VIIIa	Protein Coding	50	GC02P127418	5.597659
PRKDC	Protein Kinase, DNA-Activated, Catalytic Subunit	Protein Coding	50	GC08M047773	5.499947
ABCC1	ATP Binding Cassette Subfamily C Member 1	Protein Coding	50	GC16P015949	5.37346
CAMK2A	Calcium/Calmodulin Dependent Protein Kinase II Alpha	Protein Coding	50	GC05M150219	5.231374
YWHAG	Tyrosine 3-Monooxygenase/Tryptophan 5-Monooxygenase Activation Protein Gamma	Protein Coding	50	GC07M076327	5.130066
INPPL1	Inositol Polyphosphate Phosphatase Like 1	Protein Coding	50	GC11P072223	5.12216
VLDLR	Very Low Density Lipoprotein Receptor	Protein Coding	50	GC09P002611	5.10815
NQO1	NAD(P)H Quinone Dehydrogenase 1	Protein Coding	50	GC16M069706	5.097465
CTH	Cystathionine Gamma-Lyase	Protein Coding	50	GC01P070411	5.084268
EPHA4	EPH Receptor A4	Protein Coding	50	GC02M221418	5.049478
RPS6KB1	Ribosomal Protein S6 Kinase B1	Protein Coding	50	GC17P059893	5.044402
GLUD1	Glutamate Dehydrogenase 1	Protein Coding	50	GC10M087050	5.012873
PRKCQ	Protein Kinase C Theta	Protein Coding	50	GC10M006393	4.876119
AIFM1	Apoptosis Inducing Factor Mitochondria Associated 1	Protein Coding	50	GC0XM130129	4.712491
CCND2	Cyclin D2	Protein Coding	50	GC12P011795	4.660689
ITGA6	Integrin Subunit Alpha 6	Protein Coding	50	GC02P172427	4.638758
CD247	CD247 Molecule	Protein Coding	50	GC01M167399	4.559542
GRIA1	Glutamate Ionotropic Receptor AMPA Type Subunit 1	Protein Coding	50	GC05P153467	4.549689
ARG1	Arginase 1	Protein Coding	50	GC06P131473	4.535298
STK4	Serine/Threonine Kinase 4	Protein Coding	50	GC20P044966	4.516084
PIK3C3	Phosphatidylinositol 3-Kinase Catalytic Subunit Type 3	Protein Coding	50	GC18P041955	4.471125
CASP2	Caspase 2	Protein Coding	50	GC07P145813	4.400913

PRKACB	Protein Kinase CAMP-Activated Catalytic Subunit Beta	Protein Coding	50	GC01P084078	4.303589
POLE	DNA Polymerase Epsilon, Catalytic Subunit	Protein Coding	50	GC12M132637	4.294223
GRIN2D	Glutamate Ionotropic Receptor NMDA Type Subunit 2D	Protein Coding	50	GC19P048394	4.224753
RORA	RAR Related Orphan Receptor A	Protein Coding	50	GC15M060488	4.206495
HDAC1	Histone Deacetylase 1	Protein Coding	50	GC01P032292	4.18572
PPP2CA	Protein Phosphatase 2 Catalytic Subunit Alpha	Protein Coding	50	GC05M134194	4.146471
RIPK1	Receptor Interacting Serine/Threonine Kinase 1	Protein Coding	50	GC06P003073	4.140173
CAPN1	Calpain 1	Protein Coding	50	GC11P065300	4.13253
ACACA	Acetyl-CoA Carboxylase Alpha	Protein Coding	50	GC17M037084	4.028903
IFNGR1	Interferon Gamma Receptor 1	Protein Coding	50	GC06M137197	3.989902
WAS	WASP Actin Nucleation Promoting Factor	Protein Coding	50	GC0XP048676	3.97248
WNT7A	Wnt Family Member 7A	Protein Coding	50	GC03M016916	3.935903
PPIB	Peptidylprolyl Isomerase B	Protein Coding	50	GC15M064155	3.934706
MAP3K1	Mitogen-Activated Protein Kinase Kinase Kinase 1	Protein Coding	50	GC05P056815	3.787284
GRIA4	Glutamate Ionotropic Receptor AMPA Type Subunit 4	Protein Coding	50	GC11P105609	3.780648
PTK2B	Protein Tyrosine Kinase 2 Beta	Protein Coding	50	GC08P027311	3.778808
CACNA1G	Calcium Voltage-Gated Channel Subunit Alpha1 G	Protein Coding	50	GC17P050561	3.705323
PDPK1	3-Phosphoinositide Dependent Protein Kinase 1	Protein Coding	50	GC16P002537	3.687102
LYN	LYN Proto-Oncogene, Src Family Tyrosine Kinase	Protein Coding	50	GC08P055879	3.680766
EIF4E	Eukaryotic Translation Initiation Factor 4E	Protein Coding	50	GC04M098879	3.655698
STAT6	Signal Transducer And Activator Of Transcription 6	Protein Coding	50	GC12M057095	3.569502
MAPKAPK3	MAPK Activated Protein Kinase 3	Protein Coding	50	GC03P050611	3.538404
ESRRB	Estrogen Related Receptor Beta	Protein Coding	50	GC14P076310	3.528153
PLA2G4A	Phospholipase A2 Group IVA	Protein Coding	50	GC01P186798	3.485173
CSNK2B	Casein Kinase 2 Beta	Protein Coding	50	GC06P055205	3.477186
FASN	Fatty Acid Synthase	Protein Coding	50	GC17M082078	3.461732
CAMK2B	Calcium/Calmodulin Dependent Protein Kinase II Beta	Protein Coding	50	GC07M044217	3.452678
MGMT	O-6-Methylguanine-DNA Methyltransferase	Protein Coding	50	GC10P129467	3.406831
EGLN1	Egl-9 Family Hypoxia Inducible Factor 1	Protein Coding	50	GC01M231363	3.159219
RAD50	RAD50 Double Strand Break Repair Protein	Protein Coding	50	GC05P132556	3.124762
CHEK1	Checkpoint Kinase 1	Protein Coding	50	GC11P125625	3.09638
MAP2K3	Mitogen-Activated Protein Kinase Kinase 3	Protein Coding	50	GC17P029210	3.027504
PTPN6	Protein Tyrosine Phosphatase Non-Receptor Type 6	Protein Coding	50	GC12P011869	2.826239
FBP1	Fructose-Bisphosphatase 1	Protein Coding	50	GC09M094603	2.809341
CYLD	CYLD Lysine 63 Deubiquitinase	Protein Coding	50	GC16P050742	2.667722
IMPDH1	Inosine Monophosphate Dehydrogenase 1	Protein Coding	50	GC07M128392	2.645184
PLK1	Polo Like Kinase 1	Protein Coding	50	GC16P023958	2.579783
ENO3	Enolase 3	Protein Coding	50	GC17P004948	2.527329
ACAT1	Acetyl-CoA Acetyltransferase 1	Protein Coding	50	GC11P108121	2.522841
AURKB	Aurora Kinase B	Protein Coding	50	GC17M009194	2.486239
ANXA1	Annexin A1	Protein Coding	50	GC09P073151	2.473517
GSK3A	Glycogen Synthase Kinase 3 Alpha	Protein Coding	50	GC19M046852	2.437318
CHRNA4	Cholinergic Receptor Nicotinic Alpha 4 Subunit	Protein Coding	50	GC20M063343	2.400798
HDAC3	Histone Deacetylase 3	Protein Coding	50	GC05M141583	2.365309
DAPK1	Death Associated Protein Kinase 1	Protein Coding	50	GC09P087497	2.348815
TUBB3	Tubulin Beta 3 Class III	Protein Coding	50	GC16P089919	2.294303
GABRB3	Gamma-Aminobutyric Acid Type A Receptor Subunit Beta3	Protein Coding	50	GC15M026543	2.18755
PIK3CB	Phosphatidylinositol-4,5-Bisphosphate 3-Kinase Catalytic Subunit Beta	Protein Coding	50	GC03M138652	2.052343
SLC6A9	Solute Carrier Family 6 Member 9	Protein Coding	50	GC01M043991	1.992721
RDX	Radixin	Protein Coding	50	GC11M109864	1.313323
HCK	HCK Proto-Oncogene, Src Family Tyrosine Kinase	Protein Coding	50	GC20P032052	1.145049
CSNK1A1	Casein Kinase 1 Alpha 1	Protein Coding	50	GC05M149492	1.117669
GATA4	GATA Binding Protein 4	Protein Coding	49	GC08P011676	72.09328

ADRB1	Adrenoceptor Beta 1	Protein Coding	49	GC10P114044	41.49794
GLA	Galactosidase Alpha	Protein Coding	49	GC0XM101393	31.09844
F2	Coagulation Factor II, Thrombin	Protein Coding	49	GC11P046720	29.4141
IL1B	Interleukin 1 Beta	Protein Coding	49	GC02M112829	26.29006
MTHFR	Methylenetetrahydrofolate Reductase	Protein Coding	49	GC01M011785	26.1785
VEGFA	Vascular Endothelial Growth Factor A	Protein Coding	49	GC06P043770	24.93546
APOA1	Apolipoprotein A1	Protein Coding	49	GC11M116835	24.66955
CD36	CD36 Molecule	Protein Coding	49	GC07P080369	24.54151
SOS1	SOS Ras/Rac Guanine Nucleotide Exchange Factor 1	Protein Coding	49	GC02M038981	23.66106
VWF	Von Willebrand Factor	Protein Coding	49	GC12M005917	23.00064
KCNJ2	Potassium Inwardly Rectifying Channel Subfamily J Member 2	Protein Coding	49	GC17P070168	22.89325
PMS2	PMS1 Homolog 2, Mismatch Repair System Component	Protein Coding	49	GC07M005973	22.03437
CCL2	C-C Motif Chemokine Ligand 2	Protein Coding	49	GC17P034255	20.24362
FANCA	FA Complementation Group A	Protein Coding	49	GC16M089758	17.60921
ACTA2	Actin Alpha 2, Smooth Muscle	Protein Coding	49	GC10M088935	17.37923
MYLK2	Myosin Light Chain Kinase 2	Protein Coding	49	GC20P031819	17.27264
SERPINC1	Serpin Family C Member 1	Protein Coding	49	GC01M174251	17.15666
ACE2	Angiotensin Converting Enzyme 2	Protein Coding	49	GC0XM015494	16.96822
TP63	Tumor Protein P63	Protein Coding	49	GC03P189598	14.73364
B2M	Beta-2-Microglobulin	Protein Coding	49	GC15P044711	14.61516
GSN	Gelsolin	Protein Coding	49	GC09P121201	14.50884
CYP2D6	Cytochrome P450 Family 2 Subfamily D Member 6	Protein Coding	49	GC22M042126	14.27995
ABCA3	ATP Binding Cassette Subfamily A Member 3	Protein Coding	49	GC16M002275	13.95398
SLC6A2	Solute Carrier Family 6 Member 2	Protein Coding	49	GC16P055656	13.89124
RPS19	Ribosomal Protein S19	Protein Coding	49	GC19P041859	13.67966
IDS	Iduronate 2-Sulfatase	Protein Coding	49	GC0XM149476	13.27882
PAH	Phenylalanine Hydroxylase	Protein Coding	49	GC12M102836	12.91646
CACNA1S	Calcium Voltage-Gated Channel Subunit Alpha1 S	Protein Coding	49	GC01M201008	12.85694
PTGS2	Prostaglandin-Endoperoxide Synthase 2	Protein Coding	49	GC01M186640	12.36922
CYCS	Cytochrome C, Somatic	Protein Coding	49	GC07M025118	11.8252
NR1H4	Nuclear Receptor Subfamily 1 Group H Member 4	Protein Coding	49	GC12P100473	11.80117
RPL11	Ribosomal Protein L11	Protein Coding	49	GC01P023691	11.78334
CBS	Cystathionine Beta-Synthase	Protein Coding	49	GC21M043053	11.433
FLI1	Fli-1 Proto-Oncogene, ETS Transcription Factor	Protein Coding	49	GC11P128686	11.31041
DMPK	DM1 Protein Kinase	Protein Coding	49	GC19M045769	11.14548
COL2A1	Collagen Type II Alpha 1 Chain	Protein Coding	49	GC12M047972	10.92631
BCHE	Butyrylcholinesterase	Protein Coding	49	GC03M165772	10.45908
TRPC6	Transient Receptor Potential Cation Channel Subfamily C Member 6	Protein Coding	49	GC11M101451	10.43926
PDE4D	Phosphodiesterase 4D	Protein Coding	49	GC05M058969	10.31187
SQSTM1	Sequestosome 1	Protein Coding	49	GC05P179806	10.29245
F12	Coagulation Factor XII	Protein Coding	49	GC05M177402	10.26083
TFRC	Transferrin Receptor	Protein Coding	49	GC03M196027	10.23216
SCNN1B	Sodium Channel Epithelial 1 Subunit Beta	Protein Coding	49	GC16P023278	10.15719
DGKE	Diacylglycerol Kinase Epsilon	Protein Coding	49	GC17P056834	10.13832
P2RY12	Purinergic Receptor P2Y12	Protein Coding	49	GC03M151336	9.943155
ETS1	ETS Proto-Oncogene 1, Transcription Factor	Protein Coding	49	GC11M128458	9.588918
PLG	Plasminogen	Protein Coding	49	GC06P160702	9.549205
CHRM2	Cholinergic Receptor Muscarinic 2	Protein Coding	49	GC07P136868	9.492063
ADCY5	Adenylate Cyclase 5	Protein Coding	49	GC03M123282	9.334397
ANXA2	Annexin A2	Protein Coding	49	GC15M060347	9.193863
LRP5	LDL Receptor Related Protein 5	Protein Coding	49	GC11P068298	9.056308
RUNX1	RUNX Family Transcription Factor 1	Protein Coding	49	GC21M034787	8.819021
COL4A1	Collagen Type IV Alpha 1 Chain	Protein Coding	49	GC13M110148	8.797792

LRP6	LDL Receptor Related Protein 6	Protein Coding	49	GC12M015714	8.721567
PLD1	Phospholipase D1	Protein Coding	49	GC03M171600	8.675219
CASK	Calcium/Calmodulin Dependent Serine Protein Kinase	Protein Coding	49	GC0XM041514	8.297139
PIK3CG	Phosphatidylinositol-4,5-Bisphosphate 3-Kinase Catalytic Subunit Gamma	Protein Coding	49	GC07P106865	8.230271
MDH2	Malate Dehydrogenase 2	Protein Coding	49	GC07P076048	8.144625
IL2RB	Interleukin 2 Receptor Subunit Beta	Protein Coding	49	GC22M037125	8.110878
ROR2	Receptor Tyrosine Kinase Like Orphan Receptor 2	Protein Coding	49	GC09M091564	7.923568
PGM1	Phosphoglucomutase 1	Protein Coding	49	GC01P063593	7.816891
BAX	BCL2 Associated X, Apoptosis Regulator	Protein Coding	49	GC19P048954	7.714986
HPRT1	Hypoxanthine Phosphoribosyltransferase 1	Protein Coding	49	GC0XP134460	7.505013
STXBP1	Syntaxin Binding Protein 1	Protein Coding	49	GC09P127582	7.092999
PCK1	Phosphoenolpyruvate Carboxykinase 1	Protein Coding	49	GC20P057561	7.082088
TPO	Thyroid Peroxidase	Protein Coding	49	GC02P001374	6.928648
PGK1	Phosphoglycerate Kinase 1	Protein Coding	49	GC0XP077944	6.618314
RB1	RB Transcriptional Corepressor 1	Protein Coding	49	GC13P048303	6.521647
PYGL	Glycogen Phosphorylase L	Protein Coding	49	GC14M050857	6.241221
ITPR1	Inositol 1,4,5-Trisphosphate Receptor Type 1	Protein Coding	49	GC03P004486	6.069088
NCF1	Neutrophil Cytosolic Factor 1	Protein Coding	49	GC07P074773	6.024056
ATP2A1	ATPase Sarcoplasmic/Endoplasmic Reticulum Ca2+ Transporting 1	Protein Coding	49	GC16P032262	5.982415
UGT1A1	UDP Glucuronosyltransferase Family 1 Member A1	Protein Coding	49	GC02P233760	5.972539
ASS1	Argininosuccinate Synthase 1	Protein Coding	49	GC09P130444	5.812203
TUBA1A	Tubulin Alpha 1a	Protein Coding	49	GC12M049184	5.771887
ITGA4	Integrin Subunit Alpha 4	Protein Coding	49	GC02P181456	5.718184
KCNQ2	Potassium Voltage-Gated Channel Subfamily Q Member 2	Protein Coding	49	GC20M063400	5.640891
KRT18	Keratin 18	Protein Coding	49	GC12P052948	5.606485
MMP7	Matrix Metallopeptidase 7	Protein Coding	49	GC11M102425	5.581296
APC	APC Regulator Of WNT Signaling Pathway	Protein Coding	49	GC05P112707	5.552451
NR1H2	Nuclear Receptor Subfamily 1 Group H Member 2	Protein Coding	49	GC19P050329	5.502256
CYP1B1	Cytochrome P450 Family 1 Subfamily B Member 1	Protein Coding	49	GC02M038066	5.48844
USP8	Ubiquitin Specific Peptidase 8	Protein Coding	49	GC15P050424	5.483541
MAPK7	Mitogen-Activated Protein Kinase 7	Protein Coding	49	GC17P019379	5.400424
ITGB4	Integrin Subunit Beta 4	Protein Coding	49	GC17P075721	5.378666
PPP1CA	Protein Phosphatase 1 Catalytic Subunit Alpha	Protein Coding	49	GC11M069512	5.377098
AK2	Adenylate Kinase 2	Protein Coding	49	GC01M033007	5.296055
VRK1	VRK Serine/Threonine Kinase 1	Protein Coding	49	GC14P096797	5.056365
OPRM1	Opioid Receptor Mu 1	Protein Coding	49	GC06P154075	4.975053
MLH1	MutL Homolog 1	Protein Coding	49	GC03P036993	4.971702
QDPR	Quinoid Dihydropteridine Reductase	Protein Coding	49	GC04M017460	4.960157
USP7	Ubiquitin Specific Peptidase 7	Protein Coding	49	GC16M008892	4.876826
GNAO1	G Protein Subunit Alpha O1	Protein Coding	49	GC16P056231	4.837445
CSF2RB	Colony Stimulating Factor 2 Receptor Subunit Beta	Protein Coding	49	GC22P036913	4.762989
RAB7A	RAB7A, Member RAS Oncogene Family	Protein Coding	49	GC03P131442	4.760094
AXIN2	Axin 2	Protein Coding	49	GC17M065528	4.728275
PLCB3	Phospholipase C Beta 3	Protein Coding	49	GC11P064251	4.71481
GRB2	Growth Factor Receptor Bound Protein 2	Protein Coding	49	GC17M075318	4.703999
STIM1	Stromal Interaction Molecule 1	Protein Coding	49	GC11P003855	4.655814
TBXA2R	Thromboxane A2 Receptor	Protein Coding	49	GC19M003594	4.593489
RAB27A	RAB27A, Member RAS Oncogene Family	Protein Coding	49	GC15M055202	4.21863
PLCB1	Phospholipase C Beta 1	Protein Coding	49	GC20P008061	4.027015
SLC12A6	Solute Carrier Family 12 Member 6	Protein Coding	49	GC15M034229	4.008206
MSH2	MutS Homolog 2	Protein Coding	49	GC02P047402	3.991003
KCNN4	Potassium Calcium-Activated Channel Subfamily N Member 4	Protein Coding	49	GC19M046911	3.930002
SCD	Stearoyl-CoA Desaturase	Protein Coding	49	GC10P100347	3.92932

AHR	Aryl Hydrocarbon Receptor	Protein Coding	49	GC07P016916	3.927546
MAPK9	Mitogen-Activated Protein Kinase 9	Protein Coding	49	GC05M180234	3.856244
MAPKAPK2	MAPK Activated Protein Kinase 2	Protein Coding	49	GC01P206684	3.830369
SLC16A1	Solute Carrier Family 16 Member 1	Protein Coding	49	GC01M112926	3.812936
CYP2C8	Cytochrome P450 Family 2 Subfamily C Member 8	Protein Coding	49	GC10M095038	3.765188
IFNAR2	Interferon Alpha And Beta Receptor Subunit 2	Protein Coding	49	GC21P033229	3.604813
ODC1	Ornithine Decarboxylase 1	Protein Coding	49	GC02M010432	3.565423
PLCG1	Phospholipase C Gamma 1	Protein Coding	49	GC20P041136	3.558845
TOP1	DNA Topoisomerase I	Protein Coding	49	GC20P041028	3.546972
NF2	Neurofibromin 2	Protein Coding	49	GC22P029603	3.466426
RHEB	Ras Homolog, MTORC1 Binding	Protein Coding	49	GC07M151466	3.388181
POLH	DNA Polymerase Eta	Protein Coding	49	GC06P043576	3.319297
TBP	TATA-Box Binding Protein	Protein Coding	49	GC06P170554	3.191297
ZEB1	Zinc Finger E-Box Binding Homeobox 1	Protein Coding	49	GC10P031318	3.08133
CASP6	Caspase 6	Protein Coding	49	GC04M109688	3.055156
MAPK12	Mitogen-Activated Protein Kinase 12	Protein Coding	49	GC22M050246	2.82879
ITGA5	Integrin Subunit Alpha 5	Protein Coding	49	GC12M054398	2.750288
FGG	Fibrinogen Gamma Chain	Protein Coding	49	GC04M154604	2.690302
PRKCZ	Protein Kinase C Zeta	Protein Coding	49	GC01P002050	2.664246
GABBR2	Gamma-Aminobutyric Acid Type B Receptor Subunit 2	Protein Coding	49	GC09M098288	2.648025
PAK4	P21 (RAC1) Activated Kinase 4	Protein Coding	49	GC19P039125	2.630847
IDE	Insulin Degrading Enzyme	Protein Coding	49	GC10M092451	2.563358
ADCY1	Adenylate Cyclase 1	Protein Coding	49	GC07P045580	2.522258
IMPA1	Inositol Monophosphatase 1	Protein Coding	49	GC08M081656	2.520202
PAK1	P21 (RAC1) Activated Kinase 1	Protein Coding	49	GC11M077321	2.4943
SLC2A3	Solute Carrier Family 2 Member 3	Protein Coding	49	GC12M007919	2.390971
MST1R	Macrophage Stimulating 1 Receptor	Protein Coding	49	GC03M050077	2.308157
CDC25C	Cell Division Cycle 25C	Protein Coding	49	GC05M138285	2.299481
YWHAB	Tyrosine 3-Monooxygenase/Tryptophan 5-Monooxygenase Activation Protein Beta	Protein Coding	49	GC20P044885	2.211656
ALDOA	Aldolase, Fructose-Bisphosphate A	Protein Coding	49	GC16P030064	2.168838
PDXK	Pyridoxal Kinase	Protein Coding	49	GC21P043719	2.164847
PIP5K1C	Phosphatidylinositol-4-Phosphate 5-Kinase Type 1 Gamma	Protein Coding	49	GC19M003631	2.154803
GLRB	Glycine Receptor Beta	Protein Coding	49	GC04P157076	2.066104
NMNAT1	Nicotinamide Nucleotide Adenylyltransferase 1	Protein Coding	49	GC01P009944	1.978858
NCF4	Neutrophil Cytosolic Factor 4	Protein Coding	49	GC22P036860	1.977642
EEF2	Eukaryotic Translation Elongation Factor 2	Protein Coding	49	GC19M003976	1.806706
PTGER2	Prostaglandin E Receptor 2	Protein Coding	49	GC14P052314	1.76913
PRKAG1	Protein Kinase AMP-Activated Non-Catalytic Subunit Gamma 1	Protein Coding	49	GC12M049002	1.729927
EPHA7	EPH Receptor A7	Protein Coding	49	GC06M093240	1.525433
CCNE1	Cyclin E1	Protein Coding	49	GC19P029811	1.270621
YES1	YES Proto-Oncogene 1, Src Family Tyrosine Kinase	Protein Coding	49	GC18M000721	0.849847
PRKCI	Protein Kinase C Iota	Protein Coding	49	GC03P170222	0.627667
TTN	Titin	Protein Coding	48	GC02M178525	109.4066
GATA6	GATA Binding Protein 6	Protein Coding	48	GC18P022169	60.41695
LMNA	Lamin A/C	Protein Coding	48	GC01P156082	60.35249
MYH7	Myosin Heavy Chain 7	Protein Coding	48	GC14M023412	58.39816
RYR2	Ryanodine Receptor 2	Protein Coding	48	GC01P237042	42.357
REN	Renin	Protein Coding	48	GC01M204154	37.46292
INS	Insulin	Protein Coding	48	GC11M002159	34.55559
EDN1	Endothelin 1	Protein Coding	48	GC06P012256	34.36649
DES	Desmin	Protein Coding	48	GC02P219418	32.82232
CDK8	Cyclin Dependent Kinase 8	Protein Coding	48	GC13P026254	31.34324
IL10	Interleukin 10	Protein Coding	48	GC01M206767	31.10787

TAB2	TGF-Beta Activated Kinase 1 (MAP3K7) Binding Protein 2	Protein Coding	48	GC06P149218	29.53741
KCNJ5	Potassium Inwardly Rectifying Channel Subfamily J Member 5	Protein Coding	48	GC11P128891	28.54006
ACTN2	Actinin Alpha 2	Protein Coding	48	GC01P236686	28.49909
IFNG	Interferon Gamma	Protein Coding	48	GC12M068154	28.21553
VCL	Vinculin	Protein Coding	48	GC10P073995	27.43773
HCN4	Hyperpolarization Activated Cyclic Nucleotide Gated Potassium Channel 4	Protein Coding	48	GC15M073319	27.08376
TSC1	TSC Complex Subunit 1	Protein Coding	48	GC09M132891	27.05988
CAV1	Caveolin 1	Protein Coding	48	GC07P116524	25.67186
NR3C2	Nuclear Receptor Subfamily 3 Group C Member 2	Protein Coding	48	GC04M148078	24.64552
PITX2	Paired Like Homeodomain 2	Protein Coding	48	GC04M110617	23.97491
GAA	Alpha Glucosidase	Protein Coding	48	GC17P080101	23.85265
RPL5	Ribosomal Protein L5	Protein Coding	48	GC01P092832	23.70628
HLA-DRB1	Major Histocompatibility Complex, Class II, DR Beta 1	Protein Coding	48	GC06M032578	23.00988
JUP	Junction Plakoglobin	Protein Coding	48	GC17M041754	22.7336
ECE1	Endothelin Converting Enzyme 1	Protein Coding	48	GC01M021217	22.59888
ENPP1	Ectonucleotide Pyrophosphatase/Phosphodiesterase 1	Protein Coding	48	GC06P131808	22.55851
FGF8	Fibroblast Growth Factor 8	Protein Coding	48	GC10M101770	21.81313
CYP11B2	Cytochrome P450 Family 11 Subfamily B Member 2	Protein Coding	48	GC08M142910	21.32251
KCNJ11	Potassium Inwardly Rectifying Channel Subfamily J Member 11	Protein Coding	48	GC11M017385	21.32069
GALT	Galactose-1-Phosphate Uridylyltransferase	Protein Coding	48	GC09P035875	21.18244
LEP	Leptin	Protein Coding	48	GC07P128241	20.45584
SDHA	Succinate Dehydrogenase Complex Flavoprotein Subunit A	Protein Coding	48	GC05P000208	20.02023
TKT	Transketolase	Protein Coding	48	GC03M053224	19.8544
PIK3C2A	Phosphatidylinositol-4-Phosphate 3-Kinase Catalytic Subunit Type 2 Alpha	Protein Coding	48	GC11M017273	19.30478
BDNF	Brain Derived Neurotrophic Factor	Protein Coding	48	GC11M027654	19.06079
C3	Complement C3	Protein Coding	48	GC19M006677	18.94957
MEF2A	Myocyte Enhancer Factor 2A	Protein Coding	48	GC15P099565	18.93494
IGF2	Insulin Like Growth Factor 2	Protein Coding	48	GC11M002130	18.64835
ACTA1	Actin Alpha 1, Skeletal Muscle	Protein Coding	48	GC01M229431	18.52404
IL1RN	Interleukin 1 Receptor Antagonist	Protein Coding	48	GC02P116694	18.29916
CHAT	Choline O-Acetyltransferase	Protein Coding	48	GC10P049609	18.08404
F7	Coagulation Factor VII	Protein Coding	48	GC13P113105	18.00078
GDNF	Glial Cell Derived Neurotrophic Factor	Protein Coding	48	GC05M037812	17.83406
ABCA1	ATP Binding Cassette Subfamily A Member 1	Protein Coding	48	GC09M104781	17.564
FANCC	FA Complementation Group C	Protein Coding	48	GC09M095099	17.28323
POMC	Proopiomelanocortin	Protein Coding	48	GC02M025160	17.25946
HADHB	Hydroxyacyl-CoA Dehydrogenase Trifunctional Multienzyme Complex Subunit Beta	Protein Coding	48	GC02P026243	17.08168
SDHB	Succinate Dehydrogenase Complex Iron Sulfur Subunit B	Protein Coding	48	GC01M017238	17.00299
CAPN3	Calpain 3	Protein Coding	48	GC15P042359	16.99334
GBA	Glucosylceramidase Beta	Protein Coding	48	GC01M155234	16.69034
CD40LG	CD40 Ligand	Protein Coding	48	GC0XP136649	16.54499
GNB3	G Protein Subunit Beta 3	Protein Coding	48	GC12P006839	16.05494
CP	Ceruloplasmin	Protein Coding	48	GC03M149162	15.99263
SMPD1	Sphingomyelin Phosphodiesterase 1	Protein Coding	48	GC11P006390	15.1444
FGF2	Fibroblast Growth Factor 2	Protein Coding	48	GC04P122826	14.8358
SOX9	SRY-Box Transcription Factor 9	Protein Coding	48	GC17P072121	14.83576
ZEB2	Zinc Finger E-Box Binding Homeobox 2	Protein Coding	48	GC02M144384	14.77182
SLC4A1	Solute Carrier Family 4 Member 1 (Diego Blood Group)	Protein Coding	48	GC17M044355	14.73336
GLI2	GLI Family Zinc Finger 2	Protein Coding	48	GC02P120735	14.32301
HIF1A	Hypoxia Inducible Factor 1 Subunit Alpha	Protein Coding	48	GC14P061695	14.17591
PTPN22	Protein Tyrosine Phosphatase Non-Receptor Type 22	Protein Coding	48	GC01M113813	13.81904
TNFSF11	TNF Superfamily Member 11	Protein Coding	48	GC13P042562	13.78614
CACNA1D	Calcium Voltage-Gated Channel Subunit Alpha1 D	Protein Coding	48	GC03P053328	13.68966

NBN	Nibrin	Protein Coding	48	GC08M089933	13.50352
MPL	MPL Proto-Oncogene, Thrombopoietin Receptor	Protein Coding	48	GC01P043337	13.30669
GUSB	Glucuronidase Beta	Protein Coding	48	GC07M065960	13.30473
GLB1	Galactosidase Beta 1	Protein Coding	48	GC03M032963	13.28305
SCNN1A	Sodium Channel Epithelial 1 Subunit Alpha	Protein Coding	48	GC12M006346	12.87274
AVPR2	Arginine Vasopressin Receptor 2	Protein Coding	48	GC0XP153902	12.81612
COL1A2	Collagen Type I Alpha 2 Chain	Protein Coding	48	GC07P094394	12.74056
MBL2	Mannose Binding Lectin 2	Protein Coding	48	GC10M052760	12.68699
NFATC1	Nuclear Factor Of Activated T Cells 1	Protein Coding	48	GC18P079395	12.48894
DKC1	Dyskerin Pseudouridine Synthase 1	Protein Coding	48	GC0XP154762	12.4477
IFIH1	Interferon Induced With Helicase C Domain 1	Protein Coding	48	GC02M162267	12.34975
SLC6A4	Solute Carrier Family 6 Member 4	Protein Coding	48	GC17M030194	12.20228
GAPDH	Glyceraldehyde-3-Phosphate Dehydrogenase	Protein Coding	48	GC12P011841	12.10208
GLI1	GLI Family Zinc Finger 1	Protein Coding	48	GC12P057460	12.07896
ADORA1	Adenosine A1 Receptor	Protein Coding	48	GC01P203090	12.07876
CYBA	Cytochrome B-245 Alpha Chain	Protein Coding	48	GC16M088643	12.03308
CD40	CD40 Molecule	Protein Coding	48	GC20P046118	12.01425
NOD2	Nucleotide Binding Oligomerization Domain Containing 2	Protein Coding	48	GC16P050693	12.00856
PDX1	Pancreatic And Duodenal Homeobox 1	Protein Coding	48	GC13P027921	11.97585
FGA	Fibrinogen Alpha Chain	Protein Coding	48	GC04M154583	11.7918
SOX2	SRY-Box Transcription Factor 2	Protein Coding	48	GC03P181711	11.71671
NDUFS3	NADH:Ubiquinone Oxidoreductase Core Subunit S3	Protein Coding	48	GC11P047567	11.43018
LIPA	Lipase A, Lysosomal Acid Type	Protein Coding	48	GC10M089213	11.41037
AURKC	Aurora Kinase C	Protein Coding	48	GC19P057230	11.35859
PTGIS	Prostaglandin I2 Synthase	Protein Coding	48	GC20M049503	11.33631
HDAC8	Histone Deacetylase 8	Protein Coding	48	GC0XM072329	11.26694
CYP3A5	Cytochrome P450 Family 3 Subfamily A Member 5	Protein Coding	48	GC07M099648	11.21059
LBR	Lamin B Receptor	Protein Coding	48	GC01M225401	11.14715
HTR2A	5-Hydroxytryptamine Receptor 2A	Protein Coding	48	GC13M046831	11.12945
SMAD2	SMAD Family Member 2	Protein Coding	48	GC18M047809	11.11044
BRIP1	BRCA1 Interacting Helicase 1	Protein Coding	48	GC17M061679	11.05531
TNFRSF11B	TNF Receptor Superfamily Member 11b	Protein Coding	48	GC08M118923	11.01887
ALDH1A2	Aldehyde Dehydrogenase 1 Family Member A2	Protein Coding	48	GC15M067289	10.97073
HLA-A	Major Histocompatibility Complex, Class I, A	Protein Coding	48	GC06P055161	10.92293
RBPJ	Recombination Signal Binding Protein For Immunoglobulin Kappa J Region	Protein Coding	48	GC04P026165	10.8207
MC2R	Melanocortin 2 Receptor	Protein Coding	48	GC18M019097	10.54932
CYP2C19	Cytochrome P450 Family 2 Subfamily C Member 19	Protein Coding	48	GC10P094762	10.42584
GNA11	G Protein Subunit Alpha 11	Protein Coding	48	GC19P003094	10.4168
IRF5	Interferon Regulatory Factor 5	Protein Coding	48	GC07P128937	10.41446
IKBKG	Inhibitor Of Nuclear Factor Kappa B Kinase Regulatory Subunit Gamma	Protein Coding	48	GC0XP154541	10.30206
TXNRD2	Thioredoxin Reductase 2	Protein Coding	48	GC22M019863	10.2229
ENO2	Enolase 2	Protein Coding	48	GC12P006913	10.20132
PDCD1	Programmed Cell Death 1	Protein Coding	48	GC02M241849	10.00586
LIG4	DNA Ligase 4	Protein Coding	48	GC13M108207	9.97179
TPI1	Triosephosphate Isomerase 1	Protein Coding	48	GC12P011857	9.969728
NPR2	Natriuretic Peptide Receptor 2	Protein Coding	48	GC09P035782	9.848369
ATP1B1	ATPase Na+/K+ Transporting Subunit Beta 1	Protein Coding	48	GC01P169105	9.742409
FASLG	Fas Ligand	Protein Coding	48	GC01P172628	9.739789
CASP9	Caspase 9	Protein Coding	48	GC01M015491	9.66531
HSPA8	Heat Shock Protein Family A (Hsp70) Member 8	Protein Coding	48	GC11M123057	9.610582
IRS1	Insulin Receptor Substrate 1	Protein Coding	48	GC02M226731	9.477787
SLC12A1	Solute Carrier Family 12 Member 1	Protein Coding	48	GC15P048191	9.248837
APRT	Adenine Phosphoribosyltransferase	Protein Coding	48	GC16M088810	9.208698

HSP90AA1	Heat Shock Protein 90 Alpha Family Class A Member 1	Protein Coding	48	GC14M102080	9.148354
MAX	MYC Associated Factor X	Protein Coding	48	GC14M065009	8.906019
GATA2	GATA Binding Protein 2	Protein Coding	48	GC03M128479	8.796619
HSPD1	Heat Shock Protein Family D (Hsp60) Member 1	Protein Coding	48	GC02M197486	8.719957
MMP8	Matrix Metallopeptidase 8	Protein Coding	48	GC11M102617	8.684496
WWOX	WW Domain Containing Oxidoreductase	Protein Coding	48	GC16P078099	8.667822
PCCB	Propionyl-CoA Carboxylase Subunit Beta	Protein Coding	48	GC03P136250	8.606575
ITGA7	Integrin Subunit Alpha 7	Protein Coding	48	GC12M055684	8.588119
CYP27B1	Cytochrome P450 Family 27 Subfamily B Member 1	Protein Coding	48	GC12M057757	8.500025
TNFAIP3	TNF Alpha Induced Protein 3	Protein Coding	48	GC06P137866	8.492576
NAMPT	Nicotinamide Phosphoribosyltransferase	Protein Coding	48	GC07M106248	8.447054
KCNJ1	Potassium Inwardly Rectifying Channel Subfamily J Member 1	Protein Coding	48	GC11M128741	8.428244
PAX6	Paired Box 6	Protein Coding	48	GC11M031784	8.41086
ATP7B	ATPase Copper Transporting Beta	Protein Coding	48	GC13M051930	8.371384
TNFRSF1B	TNF Receptor Superfamily Member 1B	Protein Coding	48	GC01P012167	8.341489
WNT4	Wnt Family Member 4	Protein Coding	48	GC01M022190	8.186377
GFAP	Glial Fibrillary Acidic Protein	Protein Coding	48	GC17M044905	8.157051
GGT1	Gamma-Glutamyltransferase 1	Protein Coding	48	GC22P024583	8.143227
PLA2G6	Phospholipase A2 Group VI	Protein Coding	48	GC22M049636	8.131317
NRXN1	Neurexin 1	Protein Coding	48	GC02M049918	8.0992
VEGFC	Vascular Endothelial Growth Factor C	Protein Coding	48	GC04M176683	8.019835
CAMK2G	Calcium/Calmodulin Dependent Protein Kinase II Gamma	Protein Coding	48	GC10M073812	7.979231
YY1	YY1 Transcription Factor	Protein Coding	48	GC14P100238	7.938739
CD55	CD55 Molecule (Cromer Blood Group)	Protein Coding	48	GC01P207321	7.890712
DVL1	Dishevelled Segment Polarity Protein 1	Protein Coding	48	GC01M001335	7.87368
PCCA	Propionyl-CoA Carboxylase Subunit Alpha	Protein Coding	48	GC13P100089	7.774054
TLR5	Toll Like Receptor 5	Protein Coding	48	GC01M223132	7.71932
SCN9A	Sodium Voltage-Gated Channel Alpha Subunit 9	Protein Coding	48	GC02M166195	7.676087
FGF1	Fibroblast Growth Factor 1	Protein Coding	48	GC05M142555	7.666744
TFAP2A	Transcription Factor AP-2 Alpha	Protein Coding	48	GC06M010393	7.658914
ECHS1	Enoyl-CoA Hydratase, Short Chain 1	Protein Coding	48	GC10M133362	7.641699
CYP24A1	Cytochrome P450 Family 24 Subfamily A Member 1	Protein Coding	48	GC20M054153	7.600341
FCGR2B	Fc Fragment Of IgG Receptor IIb	Protein Coding	48	GC01P161663	7.590534
DNM1L	Dynamin 1 Like	Protein Coding	48	GC12P032679	7.559552
ERCC2	ERCC Excision Repair 2, TFIIH Core Complex Helicase Subunit	Protein Coding	48	GC19M045349	7.54204
UBE3A	Ubiquitin Protein Ligase E3A	Protein Coding	48	GC15M025333	7.403199
MFN2	Mitofusin 2	Protein Coding	48	GC01P011980	7.395424
NLRP3	NLR Family Pyrin Domain Containing 3	Protein Coding	48	GC01P247415	7.381324
PRKCB	Protein Kinase C Beta	Protein Coding	48	GC16P023967	7.36519
RHOA	Ras Homolog Family Member A	Protein Coding	48	GC03M049359	7.349468
USP9X	Ubiquitin Specific Peptidase 9 X-Linked	Protein Coding	48	GC0XP041085	7.331584
SCN3A	Sodium Voltage-Gated Channel Alpha Subunit 3	Protein Coding	48	GC02M165087	7.242191
MECOM	MDS1 And EVI1 Complex Locus	Protein Coding	48	GC03M169083	7.236889
SLC3A1	Solute Carrier Family 3 Member 1	Protein Coding	48	GC02P044275	7.223477
CHN1	Chimerin 1	Protein Coding	48	GC02M174799	7.211951
PYCR1	Pyrroline-5-Carboxylate Reductase 1	Protein Coding	48	GC17M081932	7.142602
RUNX2	RUNX Family Transcription Factor 2	Protein Coding	48	GC06P055427	7.057283
MSX2	Msh Homeobox 2	Protein Coding	48	GC05P174724	7.048959
OTC	Ornithine Transcarbamylase	Protein Coding	48	GC0XP038353	7.015935
KCNJ6	Potassium Inwardly Rectifying Channel Subfamily J Member 6	Protein Coding	48	GC21M037607	6.986918
PDHX	Pyruvate Dehydrogenase Complex Component X	Protein Coding	48	GC11P034894	6.933425
LIPE	Lipase E, Hormone Sensitive Type	Protein Coding	48	GC19M042401	6.925475
MCM4	Minichromosome Maintenance Complex Component 4	Protein Coding	48	GC08P047965	6.907182

KAT2B	Lysine Acetyltransferase 2B	Protein Coding	48	GC03P020043	6.87233
CDH5	Cadherin 5	Protein Coding	48	GC16P066366	6.871636
SLC12A2	Solute Carrier Family 12 Member 2	Protein Coding	48	GC05P128083	6.854401
AKR1B1	Aldo-Keto Reductase Family 1 Member B	Protein Coding	48	GC07M134442	6.820222
HEXB	Hexosaminidase Subunit Beta	Protein Coding	48	GC05P074640	6.81635
EPCAM	Epithelial Cell Adhesion Molecule	Protein Coding	48	GC02P047345	6.783137
GALK1	Galactokinase 1	Protein Coding	48	GC17M075751	6.730481
KCNB1	Potassium Voltage-Gated Channel Subfamily B Member 1	Protein Coding	48	GC20M049293	6.65266
GPX4	Glutathione Peroxidase 4	Protein Coding	48	GC19P001103	6.620316
SCN2A	Sodium Voltage-Gated Channel Alpha Subunit 2	Protein Coding	48	GC02P165194	6.577078
CYP27A1	Cytochrome P450 Family 27 Subfamily A Member 1	Protein Coding	48	GC02P218781	6.534291
LPIN1	Lipin 1	Protein Coding	48	GC02P011677	6.51646
MAT2A	Methionine Adenosyltransferase 2A	Protein Coding	48	GC02P085538	6.51175
ATP1A2	ATPase Na+/K+ Transporting Subunit Alpha 2	Protein Coding	48	GC01P160115	6.46145
CD3D	CD3d Molecule	Protein Coding	48	GC11M118338	6.45264
ADRA1A	Adrenoceptor Alpha 1A	Protein Coding	48	GC08M026747	6.449274
IKZF1	IKAROS Family Zinc Finger 1	Protein Coding	48	GC07P050303	6.421402
CD44	CD44 Molecule (Indian Blood Group)	Protein Coding	48	GC11P035139	6.416608
TLR7	Toll Like Receptor 7	Protein Coding	48	GC0XP012867	6.399683
ADCY6	Adenylate Cyclase 6	Protein Coding	48	GC12M048766	6.39565
GNRHR	Gonadotropin Releasing Hormone Receptor	Protein Coding	48	GC04M067737	6.388922
EPHX2	Epoxide Hydrolase 2	Protein Coding	48	GC08P027490	6.378898
SH2D1A	SH2 Domain Containing 1A	Protein Coding	48	GC0XP124227	6.377269
PRKAR1B	Protein Kinase CAMP-Dependent Type I Regulatory Subunit Beta	Protein Coding	48	GC07M000549	6.376172
EPOR	Erythropoietin Receptor	Protein Coding	48	GC19M011377	6.329104
GLS	Glutaminase	Protein Coding	48	GC02P190880	6.277811
FOXO1	Forkhead Box O1	Protein Coding	48	GC13M040555	6.277196
KRT5	Keratin 5	Protein Coding	48	GC12M052514	6.273873
SPTAN1	Spectrin Alpha, Non-Erythrocytic 1	Protein Coding	48	GC09P128552	6.270983
DDX3X	DEAD-Box Helicase 3 X-Linked	Protein Coding	48	GC0XP041333	6.266942
TYR	Tyrosinase	Protein Coding	48	GC11P089177	6.242204
AK1	Adenylate Kinase 1	Protein Coding	48	GC09M127866	6.241003
CTBP1	C-Terminal Binding Protein 1	Protein Coding	48	GC04M001211	6.129071
LYZ	Lysozyme	Protein Coding	48	GC12P069348	6.113035
ABCC2	ATP Binding Cassette Subfamily C Member 2	Protein Coding	48	GC10P099782	6.112041
PLA2G2A	Phospholipase A2 Group IIA	Protein Coding	48	GC01M019975	5.958162
IL2RG	Interleukin 2 Receptor Subunit Gamma	Protein Coding	48	GC0XM071108	5.951486
GNAI2	G Protein Subunit Alpha I2	Protein Coding	48	GC03P050226	5.927426
CDKN1B	Cyclin Dependent Kinase Inhibitor 1B	Protein Coding	48	GC12P012722	5.874437
TSHR	Thyroid Stimulating Hormone Receptor	Protein Coding	48	GC14P080954	5.799806
CXCR2	C-X-C Motif Chemokine Receptor 2	Protein Coding	48	GC02P218125	5.774536
TEAD1	TEA Domain Transcription Factor 1	Protein Coding	48	GC11P012674	5.770652
MAP3K5	Mitogen-Activated Protein Kinase Kinase Kinase 5	Protein Coding	48	GC06M136557	5.762843
PC	Pyruvate Carboxylase	Protein Coding	48	GC11M066848	5.658545
FZD2	Frizzled Class Receptor 2	Protein Coding	48	GC17P044557	5.632202
ACO2	Aconitase 2	Protein Coding	48	GC22P041448	5.62163
ENO1	Enolase 1	Protein Coding	48	GC01M008861	5.606219
ERCC3	ERCC Excision Repair 3, TFIIH Core Complex Helicase Subunit	Protein Coding	48	GC02M127257	5.588571
PAX3	Paired Box 3	Protein Coding	48	GC02M222199	5.544743
ATP2B3	ATPase Plasma Membrane Ca2+ Transporting 3	Protein Coding	48	GC0XP153517	5.511214
ADSL	Adenylosuccinate Lyase	Protein Coding	48	GC22P040346	5.499929
CDKN2B	Cyclin Dependent Kinase Inhibitor 2B	Protein Coding	48	GC09M022002	5.497993
TRPC3	Transient Receptor Potential Cation Channel Subfamily C Member 3	Protein Coding	48	GC04M121879	5.494346

GPX1	Glutathione Peroxidase 1	Protein Coding	48	GC03M049486	5.429
GCH1	GTP Cyclohydrolase 1	Protein Coding	48	GC14M054842	5.385294
PRKAB1	Protein Kinase AMP-Activated Non-Catalytic Subunit Beta 1	Protein Coding	48	GC12P119632	5.351735
KRT8	Keratin 8	Protein Coding	48	GC12M052897	5.324829
YAP1	Yes1 Associated Transcriptional Regulator	Protein Coding	48	GC11P102110	5.324244
CYP1A1	Cytochrome P450 Family 1 Subfamily A Member 1	Protein Coding	48	GC15M074719	5.323993
PTK2	Protein Tyrosine Kinase 2	Protein Coding	48	GC08M140657	5.320953
KLKB1	Kallikrein B1	Protein Coding	48	GC04P186208	5.263119
SYT1	Synaptotagmin 1	Protein Coding	48	GC12P078863	5.228981
GRIK2	Glutamate Ionotropic Receptor Kainate Type Subunit 2	Protein Coding	48	GC06P101181	5.150028
CYP2B6	Cytochrome P450 Family 2 Subfamily B Member 6	Protein Coding	48	GC19P040991	5.090544
EPHX1	Epoxide Hydrolase 1	Protein Coding	48	GC01P225810	5.087168
PNP	Purine Nucleoside Phosphorylase	Protein Coding	48	GC14P020468	5.081873
TPMT	Thiopurine S-Methyltransferase	Protein Coding	48	GC06M018128	5.073109
RASGRP1	RAS Guanyl Releasing Protein 1	Protein Coding	48	GC15M038488	5.059559
GNAI1	G Protein Subunit Alpha I1	Protein Coding	48	GC07P079769	5.05425
LAMB1	Laminin Subunit Beta 1	Protein Coding	48	GC07M107923	5.052819
LRP1	LDL Receptor Related Protein 1	Protein Coding	48	GC12P057128	4.987428
PLK4	Polo Like Kinase 4	Protein Coding	48	GC04P127880	4.986338
ESRRA	Estrogen Related Receptor Alpha	Protein Coding	48	GC11P064305	4.976031
HTR1A	5-Hydroxytryptamine Receptor 1A	Protein Coding	48	GC05M063960	4.972817
ST3GAL5	ST3 Beta-Galactoside Alpha-2,3-Sialyltransferase 5	Protein Coding	48	GC02M085839	4.962629
CYP2A6	Cytochrome P450 Family 2 Subfamily A Member 6	Protein Coding	48	GC19M040843	4.954749
CA12	Carbonic Anhydrase 12	Protein Coding	48	GC15M063321	4.937079
WNT10B	Wnt Family Member 10B	Protein Coding	48	GC12M048965	4.904746
ARHGDIA	Rho GDP Dissociation Inhibitor Alpha	Protein Coding	48	GC17M081867	4.901147
KAT2A	Lysine Acetyltransferase 2A	Protein Coding	48	GC17M042113	4.882589
ARF1	ADP Ribosylation Factor 1	Protein Coding	48	GC01P228082	4.817588
PSAT1	Phosphoserine Aminotransferase 1	Protein Coding	48	GC09P078297	4.811587
CDH11	Cadherin 11	Protein Coding	48	GC16M064943	4.72782
CD3G	CD3g Molecule	Protein Coding	48	GC11P118344	4.70062
FADD	Fas Associated Via Death Domain	Protein Coding	48	GC11P070203	4.647182
KRT14	Keratin 14	Protein Coding	48	GC17M041582	4.620577
STX1A	Syntaxin 1A	Protein Coding	48	GC07M073700	4.581769
CYSLTR2	Cysteinyl Leukotriene Receptor 2	Protein Coding	48	GC13P048653	4.534389
PPARD	Peroxisome Proliferator Activated Receptor Delta	Protein Coding	48	GC06P055324	4.51961
PCK2	Phosphoenolpyruvate Carboxykinase 2, Mitochondrial	Protein Coding	48	GC14P024094	4.514576
CSNK2A2	Casein Kinase 2 Alpha 2	Protein Coding	48	GC16M058157	4.498256
PNLIP	Pancreatic Lipase	Protein Coding	48	GC10P116545	4.493248
LMNB1	Lamin B1	Protein Coding	48	GC05P126776	4.46929
ADCY3	Adenylate Cyclase 3	Protein Coding	48	GC02M024819	4.456365
KDM1A	Lysine Demethylase 1A	Protein Coding	48	GC01P023019	4.44876
ANTXR2	ANTXR Cell Adhesion Molecule 2	Protein Coding	48	GC04M079901	4.431328
CTNNA1	Catenin Alpha 1	Protein Coding	48	GC05P138613	4.375085
HLA-DRA	Major Histocompatibility Complex, Class II, DR Alpha	Protein Coding	48	GC06P032439	4.369297
FTL	Ferritin Light Chain	Protein Coding	48	GC19P048965	4.346275
TGM2	Transglutaminase 2	Protein Coding	48	GC20M038127	4.340014
STAT2	Signal Transducer And Activator Of Transcription 2	Protein Coding	48	GC12M056341	4.272464
HSPA5	Heat Shock Protein Family A (Hsp70) Member 5	Protein Coding	48	GC09M125234	4.261473
HPGD	15-Hydroxyprostaglandin Dehydrogenase	Protein Coding	48	GC04M174490	4.25739
LEF1	Lymphoid Enhancer Binding Factor 1	Protein Coding	48	GC04M108047	4.234472
TRAF3	TNF Receptor Associated Factor 3	Protein Coding	48	GC14P106036	4.201003
GHSR	Growth Hormone Secretagogue Receptor	Protein Coding	48	GC03M172443	4.174014

PKLR	Pyruvate Kinase L/R	Protein Coding	48	GC01M155289	4.160909
GPT2	Glutamic--Pyruvic Transaminase 2	Protein Coding	48	GC16P046885	4.144284
SLC11A2	Solute Carrier Family 11 Member 2	Protein Coding	48	GC12M050952	4.119389
GK	Glycerol Kinase	Protein Coding	48	GC0XP030747	4.109521
BUB3	BUB3 Mitotic Checkpoint Protein	Protein Coding	48	GC10P123154	4.10587
CA8	Carbonic Anhydrase 8	Protein Coding	48	GC08M060187	4.073426
HDAC5	Histone Deacetylase 5	Protein Coding	48	GC17M044076	4.070316
CASP10	Caspase 10	Protein Coding	48	GC02P201182	4.034677
ROCK2	Rho Associated Coiled-Coil Containing Protein Kinase 2	Protein Coding	48	GC02M011227	4.025837
PRLR	Prolactin Receptor	Protein Coding	48	GC05M035048	4.018141
PRKAA1	Protein Kinase AMP-Activated Catalytic Subunit Alpha 1	Protein Coding	48	GC05M040759	4.011598
CEBPA	CCAAT Enhancer Binding Protein Alpha	Protein Coding	48	GC19M033299	4.005336
GPHN	Gephyrin	Protein Coding	48	GC14P066507	4.002674
CLDN1	Claudin 1	Protein Coding	48	GC03M190305	3.981822
ADORA2B	Adenosine A2b Receptor	Protein Coding	48	GC17P015927	3.938127
YWHAQ	Tyrosine 3-Monooxygenase/Tryptophan 5-Monooxygenase Activation Protein Theta	Protein Coding	48	GC02M009583	3.928472
PFN1	Profilin 1	Protein Coding	48	GC17M004945	3.925533
MCL1	MCL1 Apoptosis Regulator, BCL2 Family Member	Protein Coding	48	GC01M150561	3.924465
SIRT3	Sirtuin 3	Protein Coding	48	GC11M000215	3.910525
DUSP1	Dual Specificity Phosphatase 1	Protein Coding	48	GC05M172768	3.854333
IHH	Indian Hedgehog Signaling Molecule	Protein Coding	48	GC02M219054	3.853255
FAAH	Fatty Acid Amide Hydrolase	Protein Coding	48	GC01P046394	3.835813
NR1H3	Nuclear Receptor Subfamily 1 Group H Member 3	Protein Coding	48	GC11P047248	3.806275
SLCO1B1	Solute Carrier Organic Anion Transporter Family Member 1B1	Protein Coding	48	GC12P021132	3.800028
PI4KA	Phosphatidylinositol 4-Kinase Alpha	Protein Coding	48	GC22M020707	3.791286
ALDH7A1	Aldehyde Dehydrogenase 7 Family Member A1	Protein Coding	48	GC05M126541	3.770221
PROS1	Protein S	Protein Coding	48	GC03M093873	3.758625
DICER1	Dicer 1, Ribonuclease III	Protein Coding	48	GC14M095086	3.758605
NCF2	Neutrophil Cytosolic Factor 2	Protein Coding	48	GC01M183555	3.71955
GABRG2	Gamma-Aminobutyric Acid Type A Receptor Subunit Gamma2	Protein Coding	48	GC05P162000	3.693175
DLG4	Discs Large MAGUK Scaffold Protein 4	Protein Coding	48	GC17M007189	3.692902
COMP	Cartilage Oligomeric Matrix Protein	Protein Coding	48	GC19M018783	3.682879
CSK	C-Terminal Src Kinase	Protein Coding	48	GC15P074782	3.674388
HK2	Hexokinase 2	Protein Coding	48	GC02P074833	3.656359
MITF	Melanocyte Inducing Transcription Factor	Protein Coding	48	GC03P069788	3.65078
WNT3A	Wnt Family Member 3A	Protein Coding	48	GC01P228466	3.649712
HMOX2	Heme Oxygenase 2	Protein Coding	48	GC16P004474	3.636132
TYMS	Thymidylate Synthetase	Protein Coding	48	GC18P000657	3.634309
GCGR	Glucagon Receptor	Protein Coding	48	GC17P081804	3.603905
DIAPH1	Diaphanous Related Formin 1	Protein Coding	48	GC05M141516	3.598622
FYN	FYN Proto-Oncogene, Src Family Tyrosine Kinase	Protein Coding	48	GC06M111660	3.586632
HSPA9	Heat Shock Protein Family A (Hsp70) Member 9	Protein Coding	48	GC05M138554	3.559699
MAT1A	Methionine Adenosyltransferase 1A	Protein Coding	48	GC10M080271	3.555648
UBE2I	Ubiquitin Conjugating Enzyme E2 I	Protein Coding	48	GC16P005523	3.547546
IL4R	Interleukin 4 Receptor	Protein Coding	48	GC16P027325	3.534532
RHO	Rhodopsin	Protein Coding	48	GC03P131457	3.467937
FZD6	Frizzled Class Receptor 6	Protein Coding	48	GC08P103298	3.336462
MAPK11	Mitogen-Activated Protein Kinase 11	Protein Coding	48	GC22M050263	3.326463
GLDC	Glycine Decarboxylase	Protein Coding	48	GC09M006522	3.293749
SCP2	Sterol Carrier Protein 2	Protein Coding	48	GC01P052927	3.29289
GABRB2	Gamma-Aminobutyric Acid Type A Receptor Subunit Beta2	Protein Coding	48	GC05M161288	3.243827
LHCGR	Luteinizing Hormone/Choriogonadotropin Receptor	Protein Coding	48	GC02M048686	3.191145
PLCB4	Phospholipase C Beta 4	Protein Coding	48	GC20P009024	3.18967

PTGDR	Prostaglandin D2 Receptor	Protein Coding	48	GC14P052267	3.184685
PPIA	Peptidylprolyl Isomerase A	Protein Coding	48	GC07P044807	3.178316
ATF6	Activating Transcription Factor 6	Protein Coding	48	GC01P161766	3.173377
PRKACG	Protein Kinase CAMP-Activated Catalytic Subunit Gamma	Protein Coding	48	GC09M069013	3.158951
SLC11A1	Solute Carrier Family 11 Member 1	Protein Coding	48	GC02P218382	3.124388
PINK1	PTEN Induced Kinase 1	Protein Coding	48	GC01P020634	3.11921
SLC29A1	Solute Carrier Family 29 Member 1 (Augustine Blood Group)	Protein Coding	48	GC06P044219	3.100179
IRAK4	Interleukin 1 Receptor Associated Kinase 4	Protein Coding	48	GC12P043758	3.073303
CCND3	Cyclin D3	Protein Coding	48	GC06M041934	3.069279
FGR	FGR Proto-Oncogene, Src Family Tyrosine Kinase	Protein Coding	48	GC01M027622	3.049974
PTGIR	Prostaglandin I2 Receptor	Protein Coding	48	GC19M047072	3.034172
PIKFYVE	Phosphoinositide Kinase, FYVE-Type Zinc Finger Containing	Protein Coding	48	GC02P208266	3.024222
SLC1A1	Solute Carrier Family 1 Member 1	Protein Coding	48	GC09P004490	3.007112
YWHAZ	Tyrosine 3-Monooxygenase/Tryptophan 5-Monooxygenase Activation Protein Zeta	Protein Coding	48	GC08M100917	2.977967
AQP3	Aquaporin 3 (Gill Blood Group)	Protein Coding	48	GC09M033431	2.966317
DRD5	Dopamine Receptor D5	Protein Coding	48	GC04P009783	2.922802
CAPN2	Calpain 2	Protein Coding	48	GC01P223701	2.902884
ATP1A3	ATPase Na+/K+ Transporting Subunit Alpha 3	Protein Coding	48	GC19M041966	2.863317
MAP3K3	Mitogen-Activated Protein Kinase Kinase Kinase 3	Protein Coding	48	GC17P063622	2.861372
TUBG1	Tubulin Gamma 1	Protein Coding	48	GC17P042609	2.860412
HTR2C	5-Hydroxytryptamine Receptor 2C	Protein Coding	48	GC0XP114584	2.730914
ROR1	Receptor Tyrosine Kinase Like Orphan Receptor 1	Protein Coding	48	GC01P063774	2.721398
PTS	6-Pyruvoyltetrahydropterin Synthase	Protein Coding	48	GC11P112226	2.718461
CSNK1E	Casein Kinase 1 Epsilon	Protein Coding	48	GC22M048647	2.715701
BLNK	B Cell Linker	Protein Coding	48	GC10M096210	2.644173
KEAP1	Kelch Like ECH Associated Protein 1	Protein Coding	48	GC19M010486	2.618249
PHB	Prohibitin	Protein Coding	48	GC17M049404	2.614172
KIF11	Kinesin Family Member 11	Protein Coding	48	GC10P092574	2.601115
MAP2K6	Mitogen-Activated Protein Kinase Kinase 6	Protein Coding	48	GC17P069414	2.577499
TAP1	Transporter 1, ATP Binding Cassette Subfamily B Member	Protein Coding	48	GC06M046946	2.562773
CDC25A	Cell Division Cycle 25A	Protein Coding	48	GC03M048173	2.561294
RAB11A	RAB11A, Member RAS Oncogene Family	Protein Coding	48	GC15P077096	2.506039
PTPRJ	Protein Tyrosine Phosphatase Receptor Type J	Protein Coding	48	GC11P048002	2.490034
MAP4K4	Mitogen-Activated Protein Kinase Kinase Kinase Kinase 4	Protein Coding	48	GC02P101773	2.474691
SLC6A1	Solute Carrier Family 6 Member 1	Protein Coding	48	GC03P011049	2.457146
DIABLO	Diablo IAP-Binding Mitochondrial Protein	Protein Coding	48	GC12M122208	2.427403
ATP2B2	ATPase Plasma Membrane Ca2+ Transporting 2	Protein Coding	48	GC03M010324	2.372846
SIRT2	Sirtuin 2	Protein Coding	48	GC19M038878	2.367497
RRM1	Ribonucleotide Reductase Catalytic Subunit M1	Protein Coding	48	GC11P004115	2.350135
TXNRD1	Thioredoxin Reductase 1	Protein Coding	48	GC12P104215	2.317062
SIGMAR1	Sigma Non-Opioid Intracellular Receptor 1	Protein Coding	48	GC09M034634	2.31132
NR1D1	Nuclear Receptor Subfamily 1 Group D Member 1	Protein Coding	48	GC17M040092	2.311079
PTK6	Protein Tyrosine Kinase 6	Protein Coding	48	GC20M063528	2.308933
LIMK2	LIM Domain Kinase 2	Protein Coding	48	GC22P031212	2.300914
MC1R	Melanocortin 1 Receptor	Protein Coding	48	GC16P089912	2.286561
PLD2	Phospholipase D2	Protein Coding	48	GC17P004808	2.223238
MSN	Moesin	Protein Coding	48	GC0XP065588	2.200245
TLR1	Toll Like Receptor 1	Protein Coding	48	GC04M038793	2.173764
CALCR	Calcitonin Receptor	Protein Coding	48	GC07M093424	2.15953
RXRB	Retinoid X Receptor Beta	Protein Coding	48	GC06M033193	2.15373
GNAI3	G Protein Subunit Alpha I3	Protein Coding	48	GC01P109548	2.09895
GABBR1	Gamma-Aminobutyric Acid Type B Receptor Subunit 1	Protein Coding	48	GC06M029555	2.054587
FES	FES Proto-Oncogene, Tyrosine Kinase	Protein Coding	48	GC15P090883	2.044449

MALT1	MALT1 Paracaspase	Protein Coding	48	GC18P058671	2.025491
SLC18A2	Solute Carrier Family 18 Member A2	Protein Coding	48	GC10P117241	2.01153
PRMT1	Protein Arginine Methyltransferase 1	Protein Coding	48	GC19P049675	1.991766
FZD5	Frizzled Class Receptor 5	Protein Coding	48	GC02M207762	1.973502
GJB1	Gap Junction Protein Beta 1	Protein Coding	48	GC0XP071212	1.967724
AKR1C4	Aldo-Keto Reductase Family 1 Member C4	Protein Coding	48	GC10P005195	1.935942
HSD17B3	Hydroxysteroid 17-Beta Dehydrogenase 3	Protein Coding	48	GC09M096240	1.930955
EPHB1	EPH Receptor B1	Protein Coding	48	GC03P134598	1.915189
PPP2R1A	Protein Phosphatase 2 Scaffold Subunit Aalpha	Protein Coding	48	GC19P052189	1.87919
TUBB1	Tubulin Beta 1 Class VI	Protein Coding	48	GC20P059020	1.870794
TYRO3	TYRO3 Protein Tyrosine Kinase	Protein Coding	48	GC15P041557	1.855285
SPTLC2	Serine Palmitoyltransferase Long Chain Base Subunit 2	Protein Coding	48	GC14M077505	1.826188
TPH2	Tryptophan Hydroxylase 2	Protein Coding	48	GC12P071938	1.810075
MAPK13	Mitogen-Activated Protein Kinase 13	Protein Coding	48	GC06P055340	1.808386
MMP19	Matrix Metallopeptidase 19	Protein Coding	48	GC12M055835	1.741882
IRAK3	Interleukin 1 Receptor Associated Kinase 3	Protein Coding	48	GC12P066188	1.690512
OAT	Ornithine Aminotransferase	Protein Coding	48	GC10M124397	1.654791
PIK3C2B	Phosphatidylinositol-4-Phosphate 3-Kinase Catalytic Subunit Type 2 Beta	Protein Coding	48	GC01M204422	1.64451
PDE6A	Phosphodiesterase 6A	Protein Coding	48	GC05M149857	1.637119
FPR1	Formyl Peptide Receptor 1	Protein Coding	48	GC19M051745	1.632275
PTPN12	Protein Tyrosine Phosphatase Non-Receptor Type 12	Protein Coding	48	GC07P077537	1.603782
ADAM9	ADAM Metallopeptidase Domain 9	Protein Coding	48	GC08P038996	1.596719
GRM5	Glutamate Metabotropic Receptor 5	Protein Coding	48	GC11M088504	1.555746
SSTR2	Somatostatin Receptor 2	Protein Coding	48	GC17P073165	1.473995
TLR8	Toll Like Receptor 8	Protein Coding	48	GC0XP012924	1.455046
CDH3	Cadherin 3	Protein Coding	48	GC16P068637	1.425731
SPHK1	Sphingosine Kinase 1	Protein Coding	48	GC17P076376	1.403018
CARD11	Caspase Recruitment Domain Family Member 11	Protein Coding	48	GC07M002906	1.400302
YWHAH	Tyrosine 3-Monooxygenase/Tryptophan 5-Monooxygenase Activation Protein Eta	Protein Coding	48	GC22P031944	1.389272
MCM2	Minichromosome Maintenance Complex Component 2	Protein Coding	48	GC03P127598	1.300236
RARG	Retinoic Acid Receptor Gamma	Protein Coding	48	GC12M053210	1.274284
PLCD1	Phospholipase C Delta 1	Protein Coding	48	GC03M038008	1.263643
MTAP	Methylthioadenosine Phosphorylase	Protein Coding	48	GC09P021792	1.25509
ALDH1A1	Aldehyde Dehydrogenase 1 Family Member A1	Protein Coding	48	GC09M072900	1.228109
PIN1	Peptidylprolyl Cis/Trans Isomerase, NIMA-Interacting 1	Protein Coding	48	GC19P009835	1.188687
NR4A2	Nuclear Receptor Subfamily 4 Group A Member 2	Protein Coding	48	GC02M156324	1.186337
CDC34	Cell Division Cycle 34, Ubiqiutin Conjugating Enzyme	Protein Coding	48	GC19P000532	1.084738
UBE2C	Ubiquitin Conjugating Enzyme E2 C	Protein Coding	48	GC20P045812	0.915238
GRM6	Glutamate Metabotropic Receptor 6	Protein Coding	48	GC05M178978	0.857912
PPM1D	Protein Phosphatase, Mg2+/Mn2+ Dependent 1D	Protein Coding	48	GC17P060600	0.841744
RORB	RAR Related Orphan Receptor B	Protein Coding	48	GC09P074497	0.737195
TTK	TTK Protein Kinase	Protein Coding	48	GC06P080003	0.699553
RRM2	Ribonucleotide Reductase Regulatory Subunit M2	Protein Coding	48	GC02P010123	0.655336
MYH6	Myosin Heavy Chain 6	Protein Coding	47	GC14M023380	65.991
TBX5	T-Box Transcription Factor 5	Protein Coding	47	GC12M114353	59.67893
MYBPC3	Myosin Binding Protein C3	Protein Coding	47	GC11M068997	54.82572
NPPA	Natriuretic Peptide A	Protein Coding	47	GC01M011846	53.15208
DMD	Dystrophin	Protein Coding	47	GC0XM031097	40.26895
FBN1	Fibrillin 1	Protein Coding	47	GC15M048408	35.75937
CRP	C-Reactive Protein	Protein Coding	47	GC01M159719	32.46717
ENG	Endoglin	Protein Coding	47	GC09M127815	31.12584
RYR1	Ryanodine Receptor 1	Protein Coding	47	GC19P040452	30.35536
ZIC3	Zic Family Member 3	Protein Coding	47	GC0XP137566	30.09244

PARN	Poly(A)-Specific Ribonuclease	Protein Coding	47	GC16M014435	28.61008
DSG2	Desmoglein 2	Protein Coding	47	GC18P031498	27.91741
ABCC8	ATP Binding Cassette Subfamily C Member 8	Protein Coding	47	GC11M017392	26.36131
PMM2	Phosphomannomutase 2	Protein Coding	47	GC16P008788	25.64794
SMC1A	Structural Maintenance Of Chromosomes 1A	Protein Coding	47	GC0XM053374	23.57612
PKD2	Polycystin 2, Transient Receptor Potential Cation Channel	Protein Coding	47	GC04P088007	23.21308
FOXP3	Forkhead Box P3	Protein Coding	47	GC0XM049250	22.77121
BMP2	Bone Morphogenetic Protein 2	Protein Coding	47	GC20P006696	22.62379
HADHA	Hydroxyacyl-CoA Dehydrogenase Trifunctional Multienzyme Complex Subunit Alpha	Protein Coding	47	GC02M026190	20.62857
ISL1	ISL LIM Homeobox 1	Protein Coding	47	GC05P051383	19.92013
KCND3	Potassium Voltage-Gated Channel Subfamily D Member 3	Protein Coding	47	GC01M111770	19.0493
TGFB3	Transforming Growth Factor Beta 3	Protein Coding	47	GC14M075958	17.72285
ABCC6	ATP Binding Cassette Subfamily C Member 6	Protein Coding	47	GC16M016148	17.53897
PPARA	Peroxisome Proliferator Activated Receptor Alpha	Protein Coding	47	GC22P046150	17.52279
RAD54L	RAD54 Like	Protein Coding	47	GC01P046278	17.2191
CACNB2	Calcium Voltage-Gated Channel Auxiliary Subunit Beta 2	Protein Coding	47	GC10P018165	17.09242
ERCC1	ERCC Excision Repair 1, Endonuclease Non-Catalytic Subunit	Protein Coding	47	GC19M046972	16.83044
COL3A1	Collagen Type III Alpha 1 Chain	Protein Coding	47	GC02P188974	16.41445
SLC22A5	Solute Carrier Family 22 Member 5	Protein Coding	47	GC05P132369	16.2941
XDH	Xanthine Dehydrogenase	Protein Coding	47	GC02M031334	16.27909
EDN3	Endothelin 3	Protein Coding	47	GC20P059300	16.24931
TNNC1	Troponin C1, Slow Skeletal And Cardiac Type	Protein Coding	47	GC03M052452	15.98503
CETP	Cholesteryl Ester Transfer Protein	Protein Coding	47	GC16P056961	15.82676
ELANE	Elastase, Neutrophil Expressed	Protein Coding	47	GC19P000855	15.612
SPP1	Secreted Phosphoprotein 1	Protein Coding	47	GC04P087975	15.557
ADAMTS13	ADAM Metallopeptidase With Thrombospondin Type 1 Motif 13	Protein Coding	47	GC09P133414	15.4996
MTR	5-Methyltetrahydrofolate-Homocysteine Methyltransferase	Protein Coding	47	GC01P236795	15.49841
FXN	Frataxin	Protein Coding	47	GC09P069035	15.46715
SMAD6	SMAD Family Member 6	Protein Coding	47	GC15P066702	14.81324
GATA1	GATA Binding Protein 1	Protein Coding	47	GC0XP048786	14.66525
FHL1	Four And A Half LIM Domains 1	Protein Coding	47	GC0XP136146	14.64273
RASA1	RAS P21 Protein Activator 1	Protein Coding	47	GC05P087267	14.58189
PPP1CB	Protein Phosphatase 1 Catalytic Subunit Beta	Protein Coding	47	GC02P028752	14.54356
SMAD9	SMAD Family Member 9	Protein Coding	47	GC13M036844	14.48112
UQCRFS1	Ubiquinol-Cytochrome C Reductase, Rieske Iron-Sulfur Polypeptide 1	Protein Coding	47	GC19M029205	14.40313
ABCB11	ATP Binding Cassette Subfamily B Member 11	Protein Coding	47	GC02M168922	14.38885
F13A1	Coagulation Factor XIII A Chain	Protein Coding	47	GC06M006144	14.28835
FANCL	FA Complementation Group L	Protein Coding	47	GC02M058127	14.24912
AQP2	Aquaporin 2	Protein Coding	47	GC12P049950	14.14644
RIT1	Ras Like Without CAAX 1	Protein Coding	47	GC01M155897	14.11856
AVP	Arginine Vasopressin	Protein Coding	47	GC20M003082	13.99121
ITGAM	Integrin Subunit Alpha M	Protein Coding	47	GC16P032553	13.99076
HMGCR	3-Hydroxy-3-Methylglutaryl-CoA Reductase	Protein Coding	47	GC05P075336	13.9657
ANXA5	Annexin A5	Protein Coding	47	GC04M121667	13.89024
CCR5	C-C Motif Chemokine Receptor 5	Protein Coding	47	GC03P046383	13.70356
PSAP	Prosaposin	Protein Coding	47	GC10M071816	13.69593
PPARGC1A	PPARG Coactivator 1 Alpha	Protein Coding	47	GC04M023755	13.5255
SLC40A1	Solute Carrier Family 40 Member 1	Protein Coding	47	GC02M189560	13.49275
CACNA2D1	Calcium Voltage-Gated Channel Auxiliary Subunit Alpha2delta 1	Protein Coding	47	GC07M081946	13.46984
NPR1	Natriuretic Peptide Receptor 1	Protein Coding	47	GC01P153725	13.45291
SCN10A	Sodium Voltage-Gated Channel Alpha Subunit 10	Protein Coding	47	GC03M038713	13.43736
CDKN1C	Cyclin Dependent Kinase Inhibitor 1C	Protein Coding	47	GC11M002894	13.42865
NDUFV1	NADH:Ubiquinone Oxidoreductase Core Subunit V1	Protein Coding	47	GC11P067670	13.30645

LRP2	LDL Receptor Related Protein 2	Protein Coding	47	GC02M169127	13.29994
VKORC1	Vitamin K Epoxide Reductase Complex Subunit 1	Protein Coding	47	GC16M031116	13.25677
SCO1	Synthesis Of Cytochrome C Oxidase 1	Protein Coding	47	GC17M010672	13.22328
MEN1	Menin 1	Protein Coding	47	GC11M064803	13.13351
FANCD2	FA Complementation Group D2	Protein Coding	47	GC03P010026	12.99892
NRG1	Neuregulin 1	Protein Coding	47	GC08P031639	12.97312
AGPAT2	1-Acylglycerol-3-Phosphate O-Acyltransferase 2	Protein Coding	47	GC09M136673	12.96041
GHR	Growth Hormone Receptor	Protein Coding	47	GC05P042429	12.75416
LCAT	Lecithin-Cholesterol Acyltransferase	Protein Coding	47	GC16M067939	12.68986
TGIF1	TGFB Induced Factor Homeobox 1	Protein Coding	47	GC18P003411	12.66527
VHL	Von Hippel-Lindau Tumor Suppressor	Protein Coding	47	GC03P011015	12.6366
NPC1	NPC Intracellular Cholesterol Transporter 1	Protein Coding	47	GC18M023506	12.63027
SLC25A13	Solute Carrier Family 25 Member 13	Protein Coding	47	GC07M096120	12.2533
MRAS	Muscle RAS Oncogene Homolog	Protein Coding	47	GC03P138347	12.1098
CD46	CD46 Molecule	Protein Coding	47	GC01P207752	12.10338
FLNB	Filamin B	Protein Coding	47	GC03P058008	12.08319
PRODH	Proline Dehydrogenase 1	Protein Coding	47	GC22M018912	12.07787
PTGS1	Prostaglandin-Endoperoxide Synthase 1	Protein Coding	47	GC09P122370	12.03981
KDM6A	Lysine Demethylase 6A	Protein Coding	47	GC0XP044873	12.03918
HADH	Hydroxyacyl-CoA Dehydrogenase	Protein Coding	47	GC04P107989	11.99587
NDUFS7	NADH:Ubiquinone Oxidoreductase Core Subunit S7	Protein Coding	47	GC19P001635	11.85901
NKX2-1	NK2 Homeobox 1	Protein Coding	47	GC14M036516	11.52336
POU5F1	POU Class 5 Homeobox 1	Protein Coding	47	GC06M046826	11.48601
SLC25A20	Solute Carrier Family 25 Member 20	Protein Coding	47	GC03M048869	11.39688
ACHE	Acetylcholinesterase (Cartwright Blood Group)	Protein Coding	47	GC07M100889	11.33949
PAX2	Paired Box 2	Protein Coding	47	GC10P100735	11.28361
ASAH1	N-Acylsphingosine Amidohydrolase 1	Protein Coding	47	GC08M018055	11.22761
HNRNPA1	Heterogeneous Nuclear Ribonucleoprotein A1	Protein Coding	47	GC12P054280	11.11713
CFI	Complement Factor I	Protein Coding	47	GC04M109732	11.02
STAR	Steroidogenic Acute Regulatory Protein	Protein Coding	47	GC08M038145	10.98597
MIB1	MIB E3 Ubiquitin Protein Ligase 1	Protein Coding	47	GC18P021704	10.9254
ABCD1	ATP Binding Cassette Subfamily D Member 1	Protein Coding	47	GC0XP153724	10.87317
GJB2	Gap Junction Protein Beta 2	Protein Coding	47	GC13M020187	10.81794
TCF4	Transcription Factor 4	Protein Coding	47	GC18M055222	10.78433
RRAS2	RAS Related 2	Protein Coding	47	GC11M014299	10.70035
NR0B1	Nuclear Receptor Subfamily 0 Group B Member 1	Protein Coding	47	GC0XM030304	10.62329
STAT4	Signal Transducer And Activator Of Transcription 4	Protein Coding	47	GC02M191029	10.5689
HSD11B2	Hydroxysteroid 11-Beta Dehydrogenase 2	Protein Coding	47	GC16P067433	10.55497
FAH	Fumarylacetoacetate Hydrolase	Protein Coding	47	GC15P080152	10.48465
ATRX	ATRX Chromatin Remodeler	Protein Coding	47	GC0XM077504	10.47912
FHL2	Four And A Half LIM Domains 2	Protein Coding	47	GC02M105357	10.47568
PTH	Parathyroid Hormone	Protein Coding	47	GC11M013492	10.44198
CLU	Clusterin	Protein Coding	47	GC08M027596	10.39101
ACADM	Acyl-CoA Dehydrogenase Medium Chain	Protein Coding	47	GC01P075724	10.29205
SLC12A3	Solute Carrier Family 12 Member 3	Protein Coding	47	GC16P056865	10.27619
TJP2	Tight Junction Protein 2	Protein Coding	47	GC09P069121	10.24993
TALDO1	Transaldolase 1	Protein Coding	47	GC11P000775	10.16749
CYBB	Cytochrome B-245 Beta Chain	Protein Coding	47	GC0XP037780	10.08134
SETD2	SET Domain Containing 2, Histone Lysine Methyltransferase	Protein Coding	47	GC03M047033	10.01515
ACADS	Acyl-CoA Dehydrogenase Short Chain	Protein Coding	47	GC12P120922	9.970173
SCN1A	Sodium Voltage-Gated Channel Alpha Subunit 1	Protein Coding	47	GC02M165989	9.968833
NDUFS1	NADH:Ubiquinone Oxidoreductase Core Subunit S1	Protein Coding	47	GC02M206114	9.898447
TNFRSF13B	TNF Receptor Superfamily Member 13B	Protein Coding	47	GC17M016929	9.84614

F8	Coagulation Factor VIII	Protein Coding	47	GC0XM154835	9.809447
ACTN4	Actinin Alpha 4	Protein Coding	47	GC19P038647	9.726252
DVL3	Dishevelled Segment Polarity Protein 3	Protein Coding	47	GC03P184155	9.715116
HNRNPK	Heterogeneous Nuclear Ribonucleoprotein K	Protein Coding	47	GC09M086093	9.701031
MYCN	MYCN Proto-Oncogene, BHLH Transcription Factor	Protein Coding	47	GC02P015949	9.563142
COX4I1	Cytochrome C Oxidase Subunit 4I1	Protein Coding	47	GC16P085798	9.539335
HCFC1	Host Cell Factor C1	Protein Coding	47	GC0XM153947	9.456138
FGF10	Fibroblast Growth Factor 10	Protein Coding	47	GC05M044340	9.397043
DCTN1	Dynactin Subunit 1	Protein Coding	47	GC02M074361	9.394053
TWIST1	Twist Family BHLH Transcription Factor 1	Protein Coding	47	GC07M019020	9.316527
CUBN	Cubilin	Protein Coding	47	GC10M016824	9.212496
SIRT6	Sirtuin 6	Protein Coding	47	GC19M004174	9.18831
HSD17B4	Hydroxysteroid 17-Beta Dehydrogenase 4	Protein Coding	47	GC05P119452	9.165136
DHCR7	7-Dehydrocholesterol Reductase	Protein Coding	47	GC11M071428	9.077282
MUC1	Mucin 1, Cell Surface Associated	Protein Coding	47	GC01M155185	9.074157
SLC2A4	Solute Carrier Family 2 Member 4	Protein Coding	47	GC17P009071	8.968893
TPM3	Tropomyosin 3	Protein Coding	47	GC01M154127	8.895004
MUSK	Muscle Associated Receptor Tyrosine Kinase	Protein Coding	47	GC09P110668	8.806262
F9	Coagulation Factor IX	Protein Coding	47	GC0XP139530	8.789548
CCNH	Cyclin H	Protein Coding	47	GC05M087311	8.698235
HNRNPA2B1	Heterogeneous Nuclear Ribonucleoprotein A2/B1	Protein Coding	47	GC07M026174	8.655652
PSMA6	Proteasome 20S Subunit Alpha 6	Protein Coding	47	GC14P035278	8.633887
SCNN1G	Sodium Channel Epithelial 1 Subunit Gamma	Protein Coding	47	GC16P023182	8.599066
IGFBP7	Insulin Like Growth Factor Binding Protein 7	Protein Coding	47	GC04M057030	8.589165
CD79A	CD79a Molecule	Protein Coding	47	GC19P041877	8.568275
MYOD1	Myogenic Differentiation 1	Protein Coding	47	GC11P017741	8.564726
TRPM6	Transient Receptor Potential Cation Channel Subfamily M Member 6	Protein Coding	47	GC09M074725	8.538627
GYG1	Glycogenin 1	Protein Coding	47	GC03P148991	8.523703
SLC7A7	Solute Carrier Family 7 Member 7	Protein Coding	47	GC14M022773	8.409883
ABCB4	ATP Binding Cassette Subfamily B Member 4	Protein Coding	47	GC07M087401	8.364971
IL7R	Interleukin 7 Receptor	Protein Coding	47	GC05P035852	8.363272
FKBP1A	FKBP Prolyl Isomerase 1A	Protein Coding	47	GC20M001369	8.347362
AKR1D1	Aldo-Keto Reductase Family 1 Member D1	Protein Coding	47	GC07P138003	8.303344
CLTC	Clathrin Heavy Chain	Protein Coding	47	GC17P059619	8.276324
F2R	Coagulation Factor II Thrombin Receptor	Protein Coding	47	GC05P076716	8.238545
PYGM	Glycogen Phosphorylase, Muscle Associated	Protein Coding	47	GC11M064746	8.167618
NEDD4L	NEDD4 Like E3 Ubiquitin Protein Ligase	Protein Coding	47	GC18P058044	8.132479
FUCA1	Alpha-L-Fucosidase 1	Protein Coding	47	GC01M023845	8.095331
IL6ST	Interleukin 6 Cytokine Family Signal Transducer	Protein Coding	47	GC05M055935	8.091532
GNB1	G Protein Subunit Beta 1	Protein Coding	47	GC01M001785	7.962246
OTX2	Orthodenticle Homeobox 2	Protein Coding	47	GC14M056799	7.891281
DAG1	Dystroglycan 1	Protein Coding	47	GC03P049482	7.832101
KYNU	Kynureninase	Protein Coding	47	GC02P142877	7.817911
DCN	Decorin	Protein Coding	47	GC12M091140	7.785336
SCN8A	Sodium Voltage-Gated Channel Alpha Subunit 8	Protein Coding	47	GC12P051590	7.779497
LIFR	LIF Receptor Subunit Alpha	Protein Coding	47	GC05M038475	7.741809
CD8A	CD8a Molecule	Protein Coding	47	GC02M086784	7.721023
DCC	DCC Netrin 1 Receptor	Protein Coding	47	GC18P052340	7.700105
SUCLA2	Succinate-CoA Ligase ADP-Forming Subunit Beta	Protein Coding	47	GC13M047745	7.693885
ACTG2	Actin Gamma 2, Smooth Muscle	Protein Coding	47	GC02P073892	7.524788
CACNA1A	Calcium Voltage-Gated Channel Subunit Alpha1 A	Protein Coding	47	GC19M013206	7.448703
CTCF	CCCTC-Binding Factor	Protein Coding	47	GC16P067563	7.415608
KCNJ3	Potassium Inwardly Rectifying Channel Subfamily J Member 3	Protein Coding	47	GC02P154698	7.334656

SIN3A	SIN3 Transcription Regulator Family Member A	Protein Coding	47	GC15M075369	7.312516
KCNH1	Potassium Voltage-Gated Channel Subfamily H Member 1	Protein Coding	47	GC01M210678	7.282099
MC4R	Melanocortin 4 Receptor	Protein Coding	47	GC18M060371	7.237731
SLC4A4	Solute Carrier Family 4 Member 4	Protein Coding	47	GC04P071063	7.166667
TARDBP	TAR DNA Binding Protein	Protein Coding	47	GC01P011013	7.091522
HDAC9	Histone Deacetylase 9	Protein Coding	47	GC07P018086	7.026883
EXT2	Exostosin Glycosyltransferase 2	Protein Coding	47	GC11P044095	7.013392
ITGA2	Integrin Subunit Alpha 2	Protein Coding	47	GC05P052989	6.812703
ADH5	Alcohol Dehydrogenase 5 (Class III), Chi Polypeptide	Protein Coding	47	GC04M099070	6.793578
EEF1A2	Eukaryotic Translation Elongation Factor 1 Alpha 2	Protein Coding	47	GC20M063488	6.790446
HCN2	Hyperpolarization Activated Cyclic Nucleotide Gated Potassium And Sodium Channel 2	Protein Coding	47	GC19P000589	6.729919
NCAM1	Neural Cell Adhesion Molecule 1	Protein Coding	47	GC11P112961	6.65853
POMT1	Protein O-Mannosyltransferase 1	Protein Coding	47	GC09P131502	6.655975
ALAD	Aminolevulinate Dehydratase	Protein Coding	47	GC09M113386	6.641371
LAMC2	Laminin Subunit Gamma 2	Protein Coding	47	GC01P183186	6.624304
ADRA2B	Adrenoceptor Alpha 2B	Protein Coding	47	GC02M096112	6.591933
DLAT	Dihydrolipoamide S-Acetyltransferase	Protein Coding	47	GC11P112024	6.520257
UMPS	Uridine Monophosphate Synthetase	Protein Coding	47	GC03P124730	6.516314
GRHPR	Glyoxylate And Hydroxypyruvate Reductase	Protein Coding	47	GC09P037412	6.478292
ATP7A	ATPase Copper Transporting Alpha	Protein Coding	47	GC0XP077943	6.442934
BIN1	Bridging Integrator 1	Protein Coding	47	GC02M127048	6.426819
CRKL	CRK Like Proto-Oncogene, Adaptor Protein	Protein Coding	47	GC22P020917	6.410759
FECH	Ferrochelatase	Protein Coding	47	GC18M057544	6.371615
GOT2	Glutamic-Oxaloacetic Transaminase 2	Protein Coding	47	GC16M058707	6.358719
L1CAM	L1 Cell Adhesion Molecule	Protein Coding	47	GC0XM153864	6.31602
MYH10	Myosin Heavy Chain 10	Protein Coding	47	GC17M008474	6.308423
MS4A1	Membrane Spanning 4-Domains A1	Protein Coding	47	GC11P060475	6.282325
UQCRC2	Ubiquinol-Cytochrome C Reductase Core Protein 2	Protein Coding	47	GC16P021963	6.228486
PRNP	Prion Protein	Protein Coding	47	GC20P004686	6.220064
AXIN1	Axin 1	Protein Coding	47	GC16M000287	6.147067
DLST	Dihydrolipoamide S-Succinyltransferase	Protein Coding	47	GC14P074881	6.12253
STAMBP	STAM Binding Protein	Protein Coding	47	GC02P073828	6.108884
ADRA2A	Adrenoceptor Alpha 2A	Protein Coding	47	GC10P111077	6.089541
CSF3R	Colony Stimulating Factor 3 Receptor	Protein Coding	47	GC01M036466	6.055719
TGFBR3	Transforming Growth Factor Beta Receptor 3	Protein Coding	47	GC01M091680	6.040924
ACOX1	Acyl-CoA Oxidase 1	Protein Coding	47	GC17M075941	6.028801
ACP5	Acid Phosphatase 5, Tartrate Resistant	Protein Coding	47	GC19M011574	6.024085
DVL2	Dishevelled Segment Polarity Protein 2	Protein Coding	47	GC17M007225	6.013156
CD59	CD59 Molecule (CD59 Blood Group)	Protein Coding	47	GC11M033704	5.973887
BCL2L1	BCL2 Like 1	Protein Coding	47	GC20M031664	5.971744
SLC9A3	Solute Carrier Family 9 Member A3	Protein Coding	47	GC05M000472	5.887173
C5	Complement C5	Protein Coding	47	GC09M120952	5.863189
SLC19A1	Solute Carrier Family 19 Member 1	Protein Coding	47	GC21M045493	5.833153
C1S	Complement C1s	Protein Coding	47	GC12P011876	5.783624
ITPR3	Inositol 1,4,5-Trisphosphate Receptor Type 3	Protein Coding	47	GC06P033620	5.768302
SH2B3	SH2B Adaptor Protein 3	Protein Coding	47	GC12P111405	5.629678
HMGCL	3-Hydroxy-3-Methylglutaryl-CoA Lyase	Protein Coding	47	GC01M023801	5.60016
SLC26A3	Solute Carrier Family 26 Member 3	Protein Coding	47	GC07M107765	5.592792
NRP1	Neuropilin 1	Protein Coding	47	GC10M033177	5.553139
ALPP	Alkaline Phosphatase, Placental	Protein Coding	47	GC02P232378	5.544331
AQP4	Aquaporin 4	Protein Coding	47	GC18M026852	5.533883
PRPS1	Phosphoribosyl Pyrophosphate Synthetase 1	Protein Coding	47	GC0XP107628	5.533874
DDX58	DExD/H-Box Helicase 58	Protein Coding	47	GC09M032455	5.531783

PDHB	Pyruvate Dehydrogenase E1 Subunit Beta	Protein Coding	47	GC03M058428	5.52656
WNT3	Wnt Family Member 3	Protein Coding	47	GC17M046762	5.514174
CD28	CD28 Molecule	Protein Coding	47	GC02P203706	5.494244
NOG	Noggin	Protein Coding	47	GC17P056593	5.485706
SLC19A3	Solute Carrier Family 19 Member 3	Protein Coding	47	GC02M227685	5.474438
MASP1	MBL Associated Serine Protease 1	Protein Coding	47	GC03M187216	5.471326
CCR3	C-C Motif Chemokine Receptor 3	Protein Coding	47	GC03P046163	5.455648
CFLAR	CASP8 And FADD Like Apoptosis Regulator	Protein Coding	47	GC02P201117	5.43764
CD81	CD81 Molecule	Protein Coding	47	GC11P002414	5.435651
IRF8	Interferon Regulatory Factor 8	Protein Coding	47	GC16P085898	5.375089
FGB	Fibrinogen Beta Chain	Protein Coding	47	GC04P154572	5.325339
PCSK1	Proprotein Convertase Subtilisin/Kexin Type 1	Protein Coding	47	GC05M096391	5.30773
AVPR1A	Arginine Vasopressin Receptor 1A	Protein Coding	47	GC12M063142	5.28903
CNR1	Cannabinoid Receptor 1	Protein Coding	47	GC06M088139	5.288035
SLC9A6	Solute Carrier Family 9 Member A6	Protein Coding	47	GC0XP135985	5.283842
SLC5A2	Solute Carrier Family 5 Member 2	Protein Coding	47	GC16P032579	5.283168
FURIN	Furin, Paired Basic Amino Acid Cleaving Enzyme	Protein Coding	47	GC15P090868	5.276287
KLK3	Kallikrein Related Peptidase 3	Protein Coding	47	GC19P050854	5.260943
MTNR1B	Melatonin Receptor 1B	Protein Coding	47	GC11P092969	5.250726
ANTXR1	ANTXR Cell Adhesion Molecule 1	Protein Coding	47	GC02P068977	5.218274
DRD4	Dopamine Receptor D4	Protein Coding	47	GC11P000979	5.217536
AQP1	Aquaporin 1 (Colton Blood Group)	Protein Coding	47	GC07P030911	5.155128
ADORA3	Adenosine A3 Receptor	Protein Coding	47	GC01M111499	5.144438
XRCC6	X-Ray Repair Cross Complementing 6	Protein Coding	47	GC22P041622	5.121572
SOX5	SRY-Box Transcription Factor 5	Protein Coding	47	GC12M023529	5.094259
APAF1	Apoptotic Peptidase Activating Factor 1	Protein Coding	47	GC12P098645	5.089467
WNK1	WNK Lysine Deficient Protein Kinase 1	Protein Coding	47	GC12P000733	5.080208
LFNG	LFNG O-Fucosylpeptide 3-Beta-N-Acetylglucosaminyltransferase	Protein Coding	47	GC07P002512	5.069844
TRAF6	TNF Receptor Associated Factor 6	Protein Coding	47	GC11M036467	5.056568
CHRM1	Cholinergic Receptor Muscarinic 1	Protein Coding	47	GC11M069277	5.053566
PDP1	Pyruvate Dehydrogenase Phosphatase Catalytic Subunit 1	Protein Coding	47	GC08P093857	5.044238
TNFSF10	TNF Superfamily Member 10	Protein Coding	47	GC03M172505	5.024293
ARSA	Arylsulfatase A	Protein Coding	47	GC22M050622	5.019583
ITGAL	Integrin Subunit Alpha L	Protein Coding	47	GC16P030472	5.007076
DGAT1	Diacylglycerol O-Acyltransferase 1	Protein Coding	47	GC08M144316	4.952535
GLP1R	Glucagon Like Peptide 1 Receptor	Protein Coding	47	GC06P039048	4.946917
DDX6	DEAD-Box Helicase 6	Protein Coding	47	GC11M118748	4.936133
SUMO1	Small Ubiquitin Like Modifier 1	Protein Coding	47	GC02M202206	4.917221
PDE11A	Phosphodiesterase 11A	Protein Coding	47	GC02M177624	4.903483
LAMA1	Laminin Subunit Alpha 1	Protein Coding	47	GC18M006941	4.895816
BMP1	Bone Morphogenetic Protein 1	Protein Coding	47	GC08P022164	4.860617
SIK1	Salt Inducible Kinase 1	Protein Coding	47	GC21M043414	4.848804
NEFH	Neurofilament Heavy Chain	Protein Coding	47	GC22P029480	4.839335
IRF3	Interferon Regulatory Factor 3	Protein Coding	47	GC19M049659	4.83544
GCDH	Glutaryl-CoA Dehydrogenase	Protein Coding	47	GC19P012891	4.821562
VCAN	Versican	Protein Coding	47	GC05P083471	4.807734
ABCC4	ATP Binding Cassette Subfamily C Member 4	Protein Coding	47	GC13M095019	4.793793
ADRA1B	Adrenoceptor Alpha 1B	Protein Coding	47	GC05P159867	4.778055
PHKG2	Phosphorylase Kinase Catalytic Subunit Gamma 2	Protein Coding	47	GC16P032474	4.732093
C1R	Complement C1r	Protein Coding	47	GC12M007196	4.704608
TRPV1	Transient Receptor Potential Cation Channel Subfamily V Member 1	Protein Coding	47	GC17M003565	4.703118
PPP2R2B	Protein Phosphatase 2 Regulatory Subunit Bbeta	Protein Coding	47	GC05M146582	4.671443
ITGAV	Integrin Subunit Alpha V	Protein Coding	47	GC02P186589	4.669782

ENTPD1	Ectonucleoside Triphosphate Diphosphohydrolase 1	Protein Coding	47	GC10P095711	4.647143
ADRA1D	Adrenoceptor Alpha 1D	Protein Coding	47	GC20M004220	4.622108
NR5A2	Nuclear Receptor Subfamily 5 Group A Member 2	Protein Coding	47	GC01P199996	4.587155
PAX8	Paired Box 8	Protein Coding	47	GC02M113215	4.504353
EGLN3	Egl-9 Family Hypoxia Inducible Factor 3	Protein Coding	47	GC14M033924	4.47719
HTRA2	HtrA Serine Peptidase 2	Protein Coding	47	GC02P074529	4.463296
ASL	Argininosuccinate Lyase	Protein Coding	47	GC07P066075	4.462781
OXTR	Oxytocin Receptor	Protein Coding	47	GC03M008767	4.454534
CD3E	CD3e Molecule	Protein Coding	47	GC11P118304	4.4448
ITPA	Inosine Triphosphatase	Protein Coding	47	GC20P003284	4.440548
ILK	Integrin Linked Kinase	Protein Coding	47	GC11P006604	4.424975
UBA1	Ubiquitin Like Modifier Activating Enzyme 1	Protein Coding	47	GC0XP047190	4.419055
LDHB	Lactate Dehydrogenase B	Protein Coding	47	GC12M021635	4.395689
GPD2	Glycerol-3-Phosphate Dehydrogenase 2	Protein Coding	47	GC02P156435	4.379906
CUL3	Cullin 3	Protein Coding	47	GC02M224470	4.363338
CLCN2	Chloride Voltage-Gated Channel 2	Protein Coding	47	GC03M184346	4.328568
HYAL1	Hyaluronidase 1	Protein Coding	47	GC03M050299	4.318685
XPA	XPA, DNA Damage Recognition And Repair Factor	Protein Coding	47	GC09M097654	4.311731
FEN1	Flap Structure-Specific Endonuclease 1	Protein Coding	47	GC11P061793	4.305235
AMT	Aminomethyltransferase	Protein Coding	47	GC03M049484	4.292072
CIT	Citron Rho-Interacting Serine/Threonine Kinase	Protein Coding	47	GC12M119650	4.286277
CYP2R1	Cytochrome P450 Family 2 Subfamily R Member 1	Protein Coding	47	GC11M014877	4.277965
TPK1	Thiamin Pyrophosphokinase 1	Protein Coding	47	GC07M144451	4.274562
AMHR2	Anti-Mullerian Hormone Receptor Type 2	Protein Coding	47	GC12P053423	4.271845
FST	Follistatin	Protein Coding	47	GC05P053480	4.266909
PDK4	Pyruvate Dehydrogenase Kinase 4	Protein Coding	47	GC07M095583	4.225959
BECN1	Beclin 1	Protein Coding	47	GC17M042810	4.218086
ARAF	A-Raf Proto-Oncogene, Serine/Threonine Kinase	Protein Coding	47	GC0XP047562	4.217154
ABCC3	ATP Binding Cassette Subfamily C Member 3	Protein Coding	47	GC17P050634	4.216151
ITGA3	Integrin Subunit Alpha 3	Protein Coding	47	GC17P050055	4.208897
HTR3A	5-Hydroxytryptamine Receptor 3A	Protein Coding	47	GC11P113974	4.201302
MASP2	MBL Associated Serine Protease 2	Protein Coding	47	GC01M011026	4.185587
MSR1	Macrophage Scavenger Receptor 1	Protein Coding	47	GC08M016107	4.184314
DLC1	DLC1 Rho GTPase Activating Protein	Protein Coding	47	GC08M013083	4.167958
TACR1	Tachykinin Receptor 1	Protein Coding	47	GC02M075010	4.140045
ASNS	Asparagine Synthetase (Glutamine-Hydrolyzing)	Protein Coding	47	GC07M097854	4.108699
PARK7	Parkinsonism Associated Deglycase	Protein Coding	47	GC01P008012	4.049872
ACACB	Acetyl-CoA Carboxylase Beta	Protein Coding	47	GC12P109116	4.009852
GGCX	Gamma-Glutamyl Carboxylase	Protein Coding	47	GC02M085544	3.986571
PRKAR2A	Protein Kinase CAMP-Dependent Type II Regulatory Subunit Alpha	Protein Coding	47	GC03M048744	3.96915
ISG15	ISG15 Ubiquitin Like Modifier	Protein Coding	47	GC01P001001	3.955941
ITPR2	Inositol 1,4,5-Trisphosphate Receptor Type 2	Protein Coding	47	GC12M026336	3.947085
PSMB9	Proteasome 20S Subunit Beta 9	Protein Coding	47	GC06P055233	3.943291
NR1I2	Nuclear Receptor Subfamily 1 Group I Member 2	Protein Coding	47	GC03P119780	3.936009
NME1	NME/NM23 Nucleoside Diphosphate Kinase 1	Protein Coding	47	GC17P051154	3.927645
KAT5	Lysine Acetyltransferase 5	Protein Coding	47	GC11P065711	3.899264
TYMP	Thymidine Phosphorylase	Protein Coding	47	GC22M050525	3.89878
STAT5A	Signal Transducer And Activator Of Transcription 5A	Protein Coding	47	GC17P042287	3.887007
XPO1	Exportin 1	Protein Coding	47	GC02M061445	3.885734
CDK1	Cyclin Dependent Kinase 1	Protein Coding	47	GC10P060772	3.846567
ACVR2A	Activin A Receptor Type 2A	Protein Coding	47	GC02P147844	3.844863
MAP2K5	Mitogen-Activated Protein Kinase Kinase 5	Protein Coding	47	GC15P077101	3.843776
IRF1	Interferon Regulatory Factor 1	Protein Coding	47	GC05M132440	3.840347

PCYT1A	Phosphate Cytidylyltransferase 1A, Choline	Protein Coding	47	GC03M196214	3.83355
SLC25A1	Solute Carrier Family 25 Member 1	Protein Coding	47	GC22M019177	3.828106
BIRC5	Baculoviral IAP Repeat Containing 5	Protein Coding	47	GC17P078214	3.82405
CFL1	Cofilin 1	Protein Coding	47	GC11M065823	3.820603
VDAC1	Voltage Dependent Anion Channel 1	Protein Coding	47	GC05M133975	3.815786
PTGER3	Prostaglandin E Receptor 3	Protein Coding	47	GC01M070852	3.815734
PGD	Phosphogluconate Dehydrogenase	Protein Coding	47	GC01P010398	3.794916
GFPT1	Glutamine--Fructose-6-Phosphate Transaminase 1	Protein Coding	47	GC02M069283	3.794523
ETV6	ETS Variant Transcription Factor 6	Protein Coding	47	GC12P011649	3.791448
C1QA	Complement C1q A Chain	Protein Coding	47	GC01P022636	3.770857
LAT	Linker For Activation Of T Cells	Protein Coding	47	GC16P032276	3.767269
FOLH1	Folate Hydrolase 1	Protein Coding	47	GC11M069030	3.76142
SERPING1	Serpin Family G Member 1	Protein Coding	47	GC11P057597	3.761056
CES1	Carboxylesterase 1	Protein Coding	47	GC16M055836	3.74866
CDK9	Cyclin Dependent Kinase 9	Protein Coding	47	GC09P127852	3.734735
P2RY2	Purinergic Receptor P2Y2	Protein Coding	47	GC11P073202	3.689667
GALNT2	Polypeptide N-Acetylgalactosaminyltransferase 2	Protein Coding	47	GC01P230057	3.681892
CA4	Carbonic Anhydrase 4	Protein Coding	47	GC17P060149	3.643125
GABRA2	Gamma-Aminobutyric Acid Type A Receptor Subunit Alpha2	Protein Coding	47	GC04M046243	3.611363
SLC5A6	Solute Carrier Family 5 Member 6	Protein Coding	47	GC02M027201	3.58848
MYH2	Myosin Heavy Chain 2	Protein Coding	47	GC17M010521	3.568859
RPA1	Replication Protein A1	Protein Coding	47	GC17P001829	3.567089
HMGA1	High Mobility Group AT-Hook 1	Protein Coding	47	GC06P055309	3.512907
EFNB1	Ephrin B1	Protein Coding	47	GC0XP068828	3.48241
PRSS1	Serine Protease 1	Protein Coding	47	GC07P145775	3.465565
DDB2	Damage Specific DNA Binding Protein 2	Protein Coding	47	GC11P047237	3.458288
POLD1	DNA Polymerase Delta 1, Catalytic Subunit	Protein Coding	47	GC19P050385	3.457365
EIF4EBP1	Eukaryotic Translation Initiation Factor 4E Binding Protein 1	Protein Coding	47	GC08P038032	3.446054
TCF12	Transcription Factor 12	Protein Coding	47	GC15P056918	3.432365
EIF4G1	Eukaryotic Translation Initiation Factor 4 Gamma 1	Protein Coding	47	GC03P184314	3.41074
MDH1	Malate Dehydrogenase 1	Protein Coding	47	GC02P063557	3.331586
KCNK9	Potassium Two Pore Domain Channel Subfamily K Member 9	Protein Coding	47	GC08M139585	3.329487
FOLR1	Folate Receptor Alpha	Protein Coding	47	GC11P072190	3.321149
GABRA5	Gamma-Aminobutyric Acid Type A Receptor Subunit Alpha5	Protein Coding	47	GC15P026866	3.320755
CDKN2C	Cyclin Dependent Kinase Inhibitor 2C	Protein Coding	47	GC01P050960	3.30975
CTSH	Cathepsin H	Protein Coding	47	GC15M078925	3.301781
BACE1	Beta-Secretase 1	Protein Coding	47	GC11M117285	3.284858
CA1	Carbonic Anhydrase 1	Protein Coding	47	GC08M085327	3.272302
SLC25A12	Solute Carrier Family 25 Member 12	Protein Coding	47	GC02M171783	3.256255
F2RL3	F2R Like Thrombin Or Trypsin Receptor 3	Protein Coding	47	GC19P016888	3.208368
MAN1B1	Mannosidase Alpha Class 1B Member 1	Protein Coding	47	GC09P137086	3.2079
ATF4	Activating Transcription Factor 4	Protein Coding	47	GC22P039547	3.198173
CEL	Carboxyl Ester Lipase	Protein Coding	47	GC09P133061	3.18788
HSD3B2	Hydroxy-Delta-5-Steroid Dehydrogenase, 3 Beta- And Steroid Delta-Isomerase 2	Protein Coding	47	GC01P119414	3.179641
GLO1	Glyoxalase I	Protein Coding	47	GC06M047047	3.156507
AKR1C2	Aldo-Keto Reductase Family 1 Member C2	Protein Coding	47	GC10M004987	3.150896
CYP26B1	Cytochrome P450 Family 26 Subfamily B Member 1	Protein Coding	47	GC02M072129	3.122924
PAX9	Paired Box 9	Protein Coding	47	GC14P036657	3.096701
EPHA3	EPH Receptor A3	Protein Coding	47	GC03P089077	3.095619
ITGB6	Integrin Subunit Beta 6	Protein Coding	47	GC02M160099	3.072502
TRPV6	Transient Receptor Potential Cation Channel Subfamily V Member 6	Protein Coding	47	GC07M142871	3.070029
CD79B	CD79b Molecule	Protein Coding	47	GC17M063928	3.052755
IRF7	Interferon Regulatory Factor 7	Protein Coding	47	GC11M000612	3.045356

GAD2	Glutamate Decarboxylase 2	Protein Coding	47	GC10P026216	3.044271
RPS6KA2	Ribosomal Protein S6 Kinase A2	Protein Coding	47	GC06M166409	3.012014
HSP90B1	Heat Shock Protein 90 Beta Family Member 1	Protein Coding	47	GC12P103930	3.005677
VIPR1	Vasoactive Intestinal Peptide Receptor 1	Protein Coding	47	GC03P042490	2.963773
CSTB	Cystatin B	Protein Coding	47	GC21M043772	2.938738
C2	Complement C2	Protein Coding	47	GC06P031897	2.927277
ZBTB16	Zinc Finger And BTB Domain Containing 16	Protein Coding	47	GC11P114059	2.921218
SPRY2	Sprouty RTK Signaling Antagonist 2	Protein Coding	47	GC13M080335	2.920986
SLC20A2	Solute Carrier Family 20 Member 2	Protein Coding	47	GC08M042416	2.917325
BARD1	BRCA1 Associated RING Domain 1	Protein Coding	47	GC02M214725	2.907982
POLB	DNA Polymerase Beta	Protein Coding	47	GC08P042338	2.881646
LPAR1	Lysophosphatidic Acid Receptor 1	Protein Coding	47	GC09M110873	2.877419
SLC34A2	Solute Carrier Family 34 Member 2	Protein Coding	47	GC04P025657	2.85247
PRDX6	Peroxiredoxin 6	Protein Coding	47	GC01P173477	2.846648
PRKAB2	Protein Kinase AMP-Activated Non-Catalytic Subunit Beta 2	Protein Coding	47	GC01M147155	2.844693
PTGER4	Prostaglandin E Receptor 4	Protein Coding	47	GC05P040679	2.833682
PTPN2	Protein Tyrosine Phosphatase Non-Receptor Type 2	Protein Coding	47	GC18M019091	2.828301
RFC1	Replication Factor C Subunit 1	Protein Coding	47	GC04M039291	2.826279
PDK1	Pyruvate Dehydrogenase Kinase 1	Protein Coding	47	GC02P172555	2.81145
UNG	Uracil DNA Glycosylase	Protein Coding	47	GC12P109097	2.803279
CASP4	Caspase 4	Protein Coding	47	GC11M104942	2.794217
PLA2G1B	Phospholipase A2 Group IB	Protein Coding	47	GC12M120322	2.774573
GSS	Glutathione Synthetase	Protein Coding	47	GC20M034928	2.769941
RAB5A	RAB5A, Member RAS Oncogene Family	Protein Coding	47	GC03P019963	2.757648
UGDH	UDP-Glucose 6-Dehydrogenase	Protein Coding	47	GC04M039502	2.748183
BIRC3	Baculoviral IAP Repeat Containing 3	Protein Coding	47	GC11P102317	2.74452
NEDD4	NEDD4 E3 Ubiquitin Protein Ligase	Protein Coding	47	GC15M055826	2.738149
P2RY1	Purinergic Receptor P2Y1	Protein Coding	47	GC03P152835	2.726437
REL	REL Proto-Oncogene, NF-KB Subunit	Protein Coding	47	GC02P060881	2.723136
DDR1	Discoidin Domain Receptor Tyrosine Kinase 1	Protein Coding	47	GC06P055184	2.721838
MAP2K4	Mitogen-Activated Protein Kinase Kinase 4	Protein Coding	47	GC17P012020	2.701551
ABCB6	ATP Binding Cassette Subfamily B Member 6 (Langereis Blood Group)	Protein Coding	47	GC02M219209	2.693127
GRN	Granulin Precursor	Protein Coding	47	GC17P044345	2.668111
MMP10	Matrix Metallopeptidase 10	Protein Coding	47	GC11M102770	2.65867
ATIC	5-Aminoimidazole-4-Carboxamide Ribonucleotide Formyltransferase/IMP Cyclohydrolase	Protein Coding	47	GC02P215311	2.651895
BCAT1	Branched Chain Amino Acid Transaminase 1	Protein Coding	47	GC12M024732	2.639137
PRDX2	Peroxiredoxin 2	Protein Coding	47	GC19M012796	2.584688
GOT1	Glutamic-Oxaloacetic Transaminase 1	Protein Coding	47	GC10M099396	2.567981
PAX5	Paired Box 5	Protein Coding	47	GC09M036828	2.560974
ACLY	ATP Citrate Lyase	Protein Coding	47	GC17M041866	2.535277
FMO3	Flavin Containing Dimethylaniline Monoxygenase 3	Protein Coding	47	GC01P171090	2.515177
NEFL	Neurofilament Light Chain	Protein Coding	47	GC08M024950	2.507975
CACNB4	Calcium Voltage-Gated Channel Auxiliary Subunit Beta 4	Protein Coding	47	GC02M151832	2.500818
TOP2B	DNA Topoisomerase II Beta	Protein Coding	47	GC03M025598	2.499488
KRT17	Keratin 17	Protein Coding	47	GC17M041619	2.482228
A2M	Alpha-2-Macroglobulin	Protein Coding	47	GC12M009067	2.479291
BAP1	BRCA1 Associated Protein 1	Protein Coding	47	GC03M052401	2.432896
IL5RA	Interleukin 5 Receptor Subunit Alpha	Protein Coding	47	GC03M003066	2.428546
DPYS	Dihydropyrimidinase	Protein Coding	47	GC08M104331	2.424464
DBI	Diazepam Binding Inhibitor, Acyl-CoA Binding Protein	Protein Coding	47	GC02P119366	2.414801
CTSF	Cathepsin F	Protein Coding	47	GC11M069484	2.414751
ATP6V1B2	ATPase H+ Transporting V1 Subunit B2	Protein Coding	47	GC08P020197	2.403152
SORD	Sorbitol Dehydrogenase	Protein Coding	47	GC15P045023	2.347382

GAMT	Guanidinoacetate N-Methyltransferase	Protein Coding	47	GC19M001397	2.317857
SLC12A5	Solute Carrier Family 12 Member 5	Protein Coding	47	GC20P046021	2.310213
ITGB5	Integrin Subunit Beta 5	Protein Coding	47	GC03M124761	2.292427
TK1	Thymidine Kinase 1	Protein Coding	47	GC17M078175	2.282512
MYO6	Myosin VI	Protein Coding	47	GC06P075749	2.250311
TPT1	Tumor Protein, Translationally-Controlled 1	Protein Coding	47	GC13M045333	2.243631
ATF2	Activating Transcription Factor 2	Protein Coding	47	GC02M175072	2.242943
FKBP5	FKBP Prolyl Isomerase 5	Protein Coding	47	GC06M047025	2.233443
BAD	BCL2 Associated Agonist Of Cell Death	Protein Coding	47	GC11M069329	2.231061
CCKAR	Cholecystokinin A Receptor	Protein Coding	47	GC04M026483	2.223415
UBE2N	Ubiquitin Conjugating Enzyme E2 N	Protein Coding	47	GC12M093406	2.207489
PARP2	Poly(ADP-Ribose) Polymerase 2	Protein Coding	47	GC14P020343	2.204389
NPY1R	Neuropeptide Y Receptor Y1	Protein Coding	47	GC04M163323	2.2025
MAP2K7	Mitogen-Activated Protein Kinase Kinase 7	Protein Coding	47	GC19P007903	2.202219
ARHGEF2	Rho/Rac Guanine Nucleotide Exchange Factor 2	Protein Coding	47	GC01M155946	2.198112
ALDH5A1	Aldehyde Dehydrogenase 5 Family Member A1	Protein Coding	47	GC06P024494	2.195592
SPTLC1	Serine Palmitoyltransferase Long Chain Base Subunit 1	Protein Coding	47	GC09M092002	2.158349
OGG1	8-Oxoguanine DNA Glycosylase	Protein Coding	47	GC03P009751	2.150995
NCOA3	Nuclear Receptor Coactivator 3	Protein Coding	47	GC20P047501	2.144984
RIPK2	Receptor Interacting Serine/Threonine Kinase 2	Protein Coding	47	GC08P089758	2.138268
STK3	Serine/Threonine Kinase 3	Protein Coding	47	GC08M098372	2.137595
GABRA1	Gamma-Aminobutyric Acid Type A Receptor Subunit Alpha1	Protein Coding	47	GC05P161847	2.135029
BTRC	Beta-Transducin Repeat Containing E3 Ubiquitin Protein Ligase	Protein Coding	47	GC10P101354	2.123505
GPI	Glucose-6-Phosphate Isomerase	Protein Coding	47	GC19P034359	2.117496
NR4A3	Nuclear Receptor Subfamily 4 Group A Member 3	Protein Coding	47	GC09P099821	2.098226
LTA4H	Leukotriene A4 Hydrolase	Protein Coding	47	GC12M096000	2.082073
CHRNA2	Cholinergic Receptor Nicotinic Alpha 2 Subunit	Protein Coding	47	GC08M027459	2.049326
PRKD2	Protein Kinase D2	Protein Coding	47	GC19M046674	2.022269
GRM8	Glutamate Metabotropic Receptor 8	Protein Coding	47	GC07M126438	2.018685
GP9	Glycoprotein IX Platelet	Protein Coding	47	GC03P131453	2.002895
IMPDH2	Inosine Monophosphate Dehydrogenase 2	Protein Coding	47	GC03M049439	1.999614
FPR2	Formyl Peptide Receptor 2	Protein Coding	47	GC19P051752	1.976088
CACNA1E	Calcium Voltage-Gated Channel Subunit Alpha1 E	Protein Coding	47	GC01P181317	1.969434
PKN1	Protein Kinase N1	Protein Coding	47	GC19P014433	1.954303
DDX5	DEAD-Box Helicase 5	Protein Coding	47	GC17M064498	1.938672
PTPRA	Protein Tyrosine Phosphatase Receptor Type A	Protein Coding	47	GC20P002864	1.931305
SLC27A4	Solute Carrier Family 27 Member 4	Protein Coding	47	GC09P128340	1.927949
ACADSB	Acyl-CoA Dehydrogenase Short/Branched Chain	Protein Coding	47	GC10P123008	1.925869
LIG1	DNA Ligase 1	Protein Coding	47	GC19M048115	1.917196
PDE10A	Phosphodiesterase 10A	Protein Coding	47	GC06M165327	1.911297
FER	FER Tyrosine Kinase	Protein Coding	47	GC05P108747	1.903198
PDE8B	Phosphodiesterase 8B	Protein Coding	47	GC05P077180	1.896144
PISD	Phosphatidylserine Decarboxylase	Protein Coding	47	GC22M031696	1.873865
GDF5	Growth Differentiation Factor 5	Protein Coding	47	GC20M035433	1.867779
ARHGEF1	Rho Guanine Nucleotide Exchange Factor 1	Protein Coding	47	GC19P041883	1.860402
NR4A1	Nuclear Receptor Subfamily 4 Group A Member 1	Protein Coding	47	GC12P052022	1.854553
PSPH	Phosphoserine Phosphatase	Protein Coding	47	GC07M056010	1.824318
CAMKK2	Calcium/Calmodulin Dependent Protein Kinase Kinase 2	Protein Coding	47	GC12M121496	1.815091
PIK3R4	Phosphoinositide-3-Kinase Regulatory Subunit 4	Protein Coding	47	GC03M130678	1.772282
NT5C2	5'-Nucleotidase, Cytosolic II	Protein Coding	47	GC10M103088	1.76568
NUMB	NUMB Endocytic Adaptor Protein	Protein Coding	47	GC14M073275	1.761032
BCKDK	Branched Chain Keto Acid Dehydrogenase Kinase	Protein Coding	47	GC16P032547	1.75201
VAV1	Vav Guanine Nucleotide Exchange Factor 1	Protein Coding	47	GC19P006772	1.751882

MAD1L1	Mitotic Arrest Deficient 1 Like 1	Protein Coding	47	GC07M001815	1.727197
CBR1	Carbonyl Reductase 1	Protein Coding	47	GC21P036069	1.726304
CAMK4	Calcium/Calmodulin Dependent Protein Kinase IV	Protein Coding	47	GC05P111223	1.709928
PRKG2	Protein Kinase CGMP-Dependent 2	Protein Coding	47	GC04M081087	1.690747
AQP5	Aquaporin 5	Protein Coding	47	GC12P049961	1.671782
PRDX5	Peroxiredoxin 5	Protein Coding	47	GC11P064317	1.652952
TYRP1	Tyrosinase Related Protein 1	Protein Coding	47	GC09P012683	1.631444
CDK7	Cyclin Dependent Kinase 7	Protein Coding	47	GC05P069263	1.604106
PDE6B	Phosphodiesterase 6B	Protein Coding	47	GC04P000587	1.602695
ST3GAL3	ST3 Beta-Galactoside Alpha-2,3-Sialyltransferase 3	Protein Coding	47	GC01P043705	1.58528
CCNB1	Cyclin B1	Protein Coding	47	GC05P069167	1.582254
RDH5	Retinol Dehydrogenase 5	Protein Coding	47	GC12P055720	1.53663
WEE1	WEE1 G2 Checkpoint Kinase	Protein Coding	47	GC11P009573	1.534663
P4HA2	Prolyl 4-Hydroxylase Subunit Alpha 2	Protein Coding	47	GC05M132191	1.519392
HTR1D	5-Hydroxytryptamine Receptor 1D	Protein Coding	47	GC01M023191	1.491291
F2RL1	F2R Like Trypsin Receptor 1	Protein Coding	47	GC05P076818	1.47593
ACY1	Aminoacylase 1	Protein Coding	47	GC03P051983	1.398728
HDAC7	Histone Deacetylase 7	Protein Coding	47	GC12M047782	1.390324
NCSTN	Nicastrin	Protein Coding	47	GC01P160343	1.356709
PAK2	P21 (RAC1) Activated Kinase 2	Protein Coding	47	GC03P196739	1.348987
SFN	Stratifin	Protein Coding	47	GC01P026904	1.317006
ST14	ST14 Transmembrane Serine Protease Matriptase	Protein Coding	47	GC11P130159	1.297376
ACAT2	Acetyl-CoA Acetyltransferase 2	Protein Coding	47	GC06P159760	1.294659
EEF2K	Eukaryotic Elongation Factor 2 Kinase	Protein Coding	47	GC16P022217	1.278269
PLCB2	Phospholipase C Beta 2	Protein Coding	47	GC15M040278	1.274545
AMPD2	Adenosine Monophosphate Deaminase 2	Protein Coding	47	GC01P109616	1.271735
EPHA1	EPH Receptor A1	Protein Coding	47	GC07M143390	1.267236
UBE2D3	Ubiquitin Conjugating Enzyme E2 D3	Protein Coding	47	GC04M102794	1.255981
ALDH6A1	Aldehyde Dehydrogenase 6 Family Member A1	Protein Coding	47	GC14M074059	1.243801
DYRK1B	Dual Specificity Tyrosine Phosphorylation Regulated Kinase 1B	Protein Coding	47	GC19M039825	1.224474
CARM1	Coactivator Associated Arginine Methyltransferase 1	Protein Coding	47	GC19P010871	1.17617
NME2	NME/NM23 Nucleoside Diphosphate Kinase 2	Protein Coding	47	GC17P051165	1.062
MAP3K8	Mitogen-Activated Protein Kinase Kinase Kinase 8	Protein Coding	47	GC10P030458	1.048999
GRM3	Glutamate Metabotropic Receptor 3	Protein Coding	47	GC07P086643	0.994841
SIAH1	Siah E3 Ubiquitin Protein Ligase 1	Protein Coding	47	GC16M048357	0.980116
ATF1	Activating Transcription Factor 1	Protein Coding	47	GC12P050763	0.970037
ACVR1B	Activin A Receptor Type 1B	Protein Coding	47	GC12P051951	0.947721
GLRA1	Glycine Receptor Alpha 1	Protein Coding	47	GC05M151799	0.901504
AKR1C3	Aldo-Keto Reductase Family 1 Member C3	Protein Coding	47	GC10P005035	0.896086
SLC6A5	Solute Carrier Family 6 Member 5	Protein Coding	47	GC11P020599	0.860587
MKNK1	MAPK Interacting Serine/Threonine Kinase 1	Protein Coding	47	GC01M046557	0.81251
CDC25B	Cell Division Cycle 25B	Protein Coding	47	GC20P003794	0.790654
SET	SET Nuclear Proto-Oncogene	Protein Coding	47	GC09P128684	0.786816
MAP4K2	Mitogen-Activated Protein Kinase Kinase Kinase Kinase 2	Protein Coding	47	GC11M069340	0.697361
PSMB7	Proteasome 20S Subunit Beta 7	Protein Coding	47	GC09M124353	0.650218
GNAT2	G Protein Subunit Alpha Transducin 2	Protein Coding	47	GC01M109603	0.596362
PPP2R1B	Protein Phosphatase 2 Scaffold Subunit Abeta	Protein Coding	47	GC11M111695	0.437436
TRPM4	Transient Receptor Potential Cation Channel Subfamily M Member 4	Protein Coding	46	GC19P049157	52.1225
GJA5	Gap Junction Protein Alpha 5	Protein Coding	46	GC01M147756	33.65672
PKP2	Plakophilin 2	Protein Coding	46	GC12M032790	31.78223
CHD7	Chromodomain Helicase DNA Binding Protein 7	Protein Coding	46	GC08P060678	29.11175
APOB	Apolipoprotein B	Protein Coding	46	GC02M020956	27.12921
PKD1	Polycystin 1, Transient Receptor Potential Channel Interacting	Protein Coding	46	GC16M003057	26.18676

POLG	DNA Polymerase Gamma, Catalytic Subunit	Protein Coding	46	GC15M089365	24.73864
ERCC6	ERCC Excision Repair 6, Chromatin Remodeling Factor	Protein Coding	46	GC10M049454	23.44653
PON1	Paraoxonase 1	Protein Coding	46	GC07M095297	21.6056
NPY	Neuropeptide Y	Protein Coding	46	GC07P024290	19.06411
KCNA5	Potassium Voltage-Gated Channel Subfamily A Member 5	Protein Coding	46	GC12P005043	18.77316
SGCD	Sarcoglycan Delta	Protein Coding	46	GC05P155686	18.49668
VCAM1	Vascular Cell Adhesion Molecule 1	Protein Coding	46	GC01P100719	18.46938
MECP2	Methyl-CpG Binding Protein 2	Protein Coding	46	GC0XM154021	18.06594
CTLA4	Cytotoxic T-Lymphocyte Associated Protein 4	Protein Coding	46	GC02P203867	18.04834
HBB	Hemoglobin Subunit Beta	Protein Coding	46	GC11M005434	17.11439
ADRA2C	Adrenoceptor Alpha 2C	Protein Coding	46	GC04P003766	17.10166
CFH	Complement Factor H	Protein Coding	46	GC01P196621	17.01348
SELP	Selectin P	Protein Coding	46	GC01M169558	16.66196
DSC2	Desmocollin 2	Protein Coding	46	GC18M031058	16.5834
HLA-B	Major Histocompatibility Complex, Class I, B	Protein Coding	46	GC06M046832	16.56978
AMPD1	Adenosine Monophosphate Deaminase 1	Protein Coding	46	GC01M114673	16.5462
MYH11	Myosin Heavy Chain 11	Protein Coding	46	GC16M015704	15.59201
TBX3	T-Box Transcription Factor 3	Protein Coding	46	GC12M114670	15.00186
IL4	Interleukin 4	Protein Coding	46	GC05P132673	14.87042
FCGR2A	Fc Fragment Of IgG Receptor IIa	Protein Coding	46	GC01P161505	14.28978
FGF23	Fibroblast Growth Factor 23	Protein Coding	46	GC12M004368	14.2753
ERCC4	ERCC Excision Repair 4, Endonuclease Catalytic Subunit	Protein Coding	46	GC16P013920	14.23257
CXCL12	C-X-C Motif Chemokine Ligand 12	Protein Coding	46	GC10M044294	14.19641
LIPC	Lipase C, Hepatic Type	Protein Coding	46	GC15P058410	14.02045
IL1R1	Interleukin 1 Receptor Type 1	Protein Coding	46	GC02P102136	13.83947
EHMT1	Euchromatic Histone Lysine Methyltransferase 1	Protein Coding	46	GC09P137618	13.65637
ACADVL	Acyl-CoA Dehydrogenase Very Long Chain	Protein Coding	46	GC17P007219	13.24326
FOXP1	Forkhead Box P1	Protein Coding	46	GC03M070926	13.20486
DLL1	Delta Like Canonical Notch Ligand 1	Protein Coding	46	GC06M170282	12.75422
MSX1	Msh Homeobox 1	Protein Coding	46	GC04P004861	12.59908
UCP2	Uncoupling Protein 2	Protein Coding	46	GC11M073974	12.5967
SMC3	Structural Maintenance Of Chromosomes 3	Protein Coding	46	GC10P110567	12.48099
WFS1	Wolframin ER Transmembrane Glycoprotein	Protein Coding	46	GC04P006260	12.19278
SLC19A2	Solute Carrier Family 19 Member 2	Protein Coding	46	GC01M169463	11.92433
SOS2	SOS Ras/Rho Guanine Nucleotide Exchange Factor 2	Protein Coding	46	GC14M050117	11.75083
GATM	Glycine Amidinotransferase	Protein Coding	46	GC15M045361	11.69592
ANGPT2	Angiopoietin 2	Protein Coding	46	GC08M006499	11.67185
SOX10	SRY-Box Transcription Factor 10	Protein Coding	46	GC22M049686	11.39844
NNT	Nicotinamide Nucleotide Transhydrogenase	Protein Coding	46	GC05P043668	11.39068
AFP	Alpha Fetoprotein	Protein Coding	46	GC04P073431	11.38673
KL	Klotho	Protein Coding	46	GC13P033016	11.12069
DLL4	Delta Like Canonical Notch Ligand 4	Protein Coding	46	GC15P040929	10.98164
IGF2R	Insulin Like Growth Factor 2 Receptor	Protein Coding	46	GC06P159969	10.85637
S100B	S100 Calcium Binding Protein B	Protein Coding	46	GC21M048326	10.85576
AGXT	Alanine--Glyoxylate And Serine--Pyruvate Aminotransferase	Protein Coding	46	GC02P240868	10.75197
CHRNA1	Cholinergic Receptor Nicotinic Alpha 1 Subunit	Protein Coding	46	GC02M174747	10.52297
FH	Fumarate Hydratase	Protein Coding	46	GC01M241499	10.3507
ANGPT1	Angiopoietin 1	Protein Coding	46	GC08M107246	10.33677
HSPG2	Heparan Sulfate Proteoglycan 2	Protein Coding	46	GC01M021822	10.17426
BMP7	Bone Morphogenetic Protein 7	Protein Coding	46	GC20M057168	10.13171
TPM2	Tropomyosin 2	Protein Coding	46	GC09M035672	9.868153
SMARCB1	SWI/SNF Related, Matrix Associated, Actin Dependent Regulator Of Chromatin, Subfamily B, Member 1	Protein Coding	46	GC22P023786	9.851456
CFB	Complement Factor B	Protein Coding	46	GC06P031945	9.704746

GP1BA	Glycoprotein Ib Platelet Subunit Alpha	Protein Coding	46	GC17P004932	9.619515
CLCN7	Chloride Voltage-Gated Channel 7	Protein Coding	46	GC16M001444	9.617531
CKB	Creatine Kinase B	Protein Coding	46	GC14M103519	9.512398
XYLT2	Xylosyltransferase 2	Protein Coding	46	GC17P050347	9.422556
PHYH	Phytanoyl-CoA 2-Hydroxylase	Protein Coding	46	GC10M013277	9.417807
NDUFS8	NADH:Ubiquinone Oxidoreductase Core Subunit S8	Protein Coding	46	GC11P068030	9.390641
HNF1A	HNF1 Homeobox A	Protein Coding	46	GC12P120978	9.382927
ADRB3	Adrenoceptor Beta 3	Protein Coding	46	GC08M037962	9.078999
GPC3	Glypican 3	Protein Coding	46	GC0XM133535	9.073647
PTHLH	Parathyroid Hormone Like Hormone	Protein Coding	46	GC12M027959	9.046987
TRPS1	Transcriptional Repressor GATA Binding 1	Protein Coding	46	GC08M115408	8.99094
COL5A1	Collagen Type V Alpha 1 Chain	Protein Coding	46	GC09P134641	8.961746
RAD21	RAD21 Cohesin Complex Component	Protein Coding	46	GC08M116846	8.667648
SMARCE1	SWI/SNF Related, Matrix Associated, Actin Dependent Regulator Of Chromatin, Subfamily E, Member 1	Protein Coding	46	GC17M040624	8.601816
RAG1	Recombination Activating 1	Protein Coding	46	GC11P036520	8.539834
LMX1B	LIM Homeobox Transcription Factor 1 Beta	Protein Coding	46	GC09P126614	8.505762
ALDH18A1	Aldehyde Dehydrogenase 18 Family Member A1	Protein Coding	46	GC10M095605	8.485054
SREBF1	Sterol Regulatory Element Binding Transcription Factor 1	Protein Coding	46	GC17M017810	8.393436
NEK9	NIMA Related Kinase 9	Protein Coding	46	GC14M075079	8.369226
GABRD	Gamma-Aminobutyric Acid Type A Receptor Subunit Delta	Protein Coding	46	GC01P002019	8.350296
SLC2A9	Solute Carrier Family 2 Member 9	Protein Coding	46	GC04M009772	8.332355
TCF7L2	Transcription Factor 7 Like 2	Protein Coding	46	GC10P112950	8.308474
TNFRSF11A	TNF Receptor Superfamily Member 11a	Protein Coding	46	GC18P062325	8.286144
TRPM7	Transient Receptor Potential Cation Channel Subfamily M Member 7	Protein Coding	46	GC15M050552	8.267522
AMACR	Alpha-Methylacyl-CoA Racemase	Protein Coding	46	GC05M033986	8.164411
CSF2RA	Colony Stimulating Factor 2 Receptor Subunit Alpha	Protein Coding	46	GC0XP001333	8.158823
SUCLG1	Succinate-CoA Ligase GDP/ADP-Forming Subunit Alpha	Protein Coding	46	GC02M084423	8.082575
SLC6A8	Solute Carrier Family 6 Member 8	Protein Coding	46	GC0XP153688	7.942528
CYP7B1	Cytochrome P450 Family 7 Subfamily B Member 1	Protein Coding	46	GC08M064587	7.700056
GPC4	Glypican 4	Protein Coding	46	GC0XM133300	7.676484
MST1	Macrophage Stimulating 1	Protein Coding	46	GC03M049683	7.592974
ETFA	Electron Transfer Flavoprotein Subunit Alpha	Protein Coding	46	GC15M076215	7.366265
TGM1	Transglutaminase 1	Protein Coding	46	GC14M024249	7.29011
FDFT1	Farnesyl-Diphosphate Farnesyltransferase 1	Protein Coding	46	GC08P011795	7.199816
CYC1	Cytochrome C1	Protein Coding	46	GC08P144095	7.138948
CPS1	Carbamoyl-Phosphate Synthase 1	Protein Coding	46	GC02P210477	7.04788
EYA1	EYA Transcriptional Coactivator And Phosphatase 1	Protein Coding	46	GC08M071210	7.029633
SMARCC2	SWI/SNF Related, Matrix Associated, Actin Dependent Regulator Of Chromatin Subfamily C Member 2	Protein Coding	46	GC12M056206	6.954144
HPD	4-Hydroxyphenylpyruvate Dioxygenase	Protein Coding	46	GC12M121839	6.873634
KCNA1	Potassium Voltage-Gated Channel Subfamily A Member 1	Protein Coding	46	GC12P011800	6.857203
POLA1	DNA Polymerase Alpha 1, Catalytic Subunit	Protein Coding	46	GC0XP024693	6.835322
GZMB	Granzyme B	Protein Coding	46	GC14M024630	6.722486
ADORA2A	Adenosine A2a Receptor	Protein Coding	46	GC22P024417	6.717338
TIMP3	TIMP Metallopeptidase Inhibitor 3	Protein Coding	46	GC22P032800	6.670032
MMAB	Metabolism Of Cobalamin Associated B	Protein Coding	46	GC12M109553	6.649108
DHCR24	24-Dehydrocholesterol Reductase	Protein Coding	46	GC01M054849	6.616353
STRADA	STE20 Related Adaptor Alpha	Protein Coding	46	GC17M063683	6.412165
TXN	Thioredoxin	Protein Coding	46	GC09M110243	6.368489
SCARB1	Scavenger Receptor Class B Member 1	Protein Coding	46	GC12M124776	6.203468
KLF4	Kruppel Like Factor 4	Protein Coding	46	GC09M107484	6.135983
PDE6D	Phosphodiesterase 6D	Protein Coding	46	GC02M231732	6.133188
KCNA2	Potassium Voltage-Gated Channel Subfamily A Member 2	Protein Coding	46	GC01M110519	6.049456
PNKP	Polynucleotide Kinase 3'-Phosphatase	Protein Coding	46	GC19M049861	6.029655

TGFA	Transforming Growth Factor Alpha	Protein Coding	46	GC02M070447	6.024669
RELB	RELB Proto-Oncogene, NF-KB Subunit	Protein Coding	46	GC19P045002	5.995442
KRT19	Keratin 19	Protein Coding	46	GC17M041523	5.898638
SHC1	SHC Adaptor Protein 1	Protein Coding	46	GC01M154962	5.872075
OGDH	Oxoglutarate Dehydrogenase	Protein Coding	46	GC07P044606	5.76431
FGF17	Fibroblast Growth Factor 17	Protein Coding	46	GC08P022042	5.728161
VAPB	VAMP Associated Protein B And C	Protein Coding	46	GC20P058389	5.649422
HSD17B10	Hydroxysteroid 17-Beta Dehydrogenase 10	Protein Coding	46	GC0XM053431	5.636254
CYP2E1	Cytochrome P450 Family 2 Subfamily E Member 1	Protein Coding	46	GC10P133520	5.619465
CHKB	Choline Kinase Beta	Protein Coding	46	GC22M050578	5.611941
HCN1	Hyperpolarization Activated Cyclic Nucleotide Gated Potassium Channel 1	Protein Coding	46	GC05M045260	5.608838
SERPINH1	Serpin Family H Member 1	Protein Coding	46	GC11P075562	5.55515
ALDOB	Aldolase, Fructose-Bisphosphate B	Protein Coding	46	GC09M101420	5.525007
LAMB3	Laminin Subunit Beta 3	Protein Coding	46	GC01M209614	5.52446
UBE2L3	Ubiquitin Conjugating Enzyme E2 L3	Protein Coding	46	GC22P021549	5.38202
ANG	Angiogenin	Protein Coding	46	GC14P021583	5.358502
ESRRG	Estrogen Related Receptor Gamma	Protein Coding	46	GC01M216503	5.328353
PEPD	Peptidase D	Protein Coding	46	GC19M033386	5.286637
HNMT	Histamine N-Methyltransferase	Protein Coding	46	GC02P137964	5.178851
ITCH	Itchy E3 Ubiquitin Protein Ligase	Protein Coding	46	GC20P034363	5.139408
AMPD3	Adenosine Monophosphate Deaminase 3	Protein Coding	46	GC11P010309	5.133397
GRM7	Glutamate Metabotropic Receptor 7	Protein Coding	46	GC03P006770	5.078851
THBS2	Thrombospondin 2	Protein Coding	46	GC06M169215	5.061082
PPP2R5D	Protein Phosphatase 2 Regulatory Subunit B'Delta	Protein Coding	46	GC06P055406	4.971045
PSMB4	Proteasome 20S Subunit Beta 4	Protein Coding	46	GC01P151372	4.907325
DHODH	Dihydroorotate Dehydrogenase (Quinone)	Protein Coding	46	GC16P072008	4.907226
NUP62	Nucleoporin 62	Protein Coding	46	GC19M049906	4.830199
ALAS2	5'-Aminolevulinate Synthase 2	Protein Coding	46	GC0XM055009	4.817739
HELLS	Helicase, Lymphoid Specific	Protein Coding	46	GC10P094501	4.792903
CDC6	Cell Division Cycle 6	Protein Coding	46	GC17P040287	4.792455
SLC16A2	Solute Carrier Family 16 Member 2	Protein Coding	46	GC0XP074439	4.776995
PLOD3	Procollagen-Lysine,2-Oxoglutarate 5-Dioxygenase 3	Protein Coding	46	GC07M101205	4.742651
GNPAT	Glyceronephosphate O-Acyltransferase	Protein Coding	46	GC01P231241	4.697982
F11	Coagulation Factor XI	Protein Coding	46	GC04P186265	4.685108
ERN1	Endoplasmic Reticulum To Nucleus Signaling 1	Protein Coding	46	GC17M064039	4.662542
P2RX7	Purinergic Receptor P2X 7	Protein Coding	46	GC12P123575	4.590392
TRRAP	Transformation/Transcription Domain Associated Protein	Protein Coding	46	GC07P098877	4.563743
PPT1	Palmitoyl-Protein Thioesterase 1	Protein Coding	46	GC01M040072	4.495166
XBP1	X-Box Binding Protein 1	Protein Coding	46	GC22M028794	4.461245
SLC7A9	Solute Carrier Family 7 Member 9	Protein Coding	46	GC19M032830	4.448736
BID	BH3 Interacting Domain Death Agonist	Protein Coding	46	GC22M017734	4.415527
SERPIND1	Serpin Family D Member 1	Protein Coding	46	GC22P020944	4.322843
CHRNA7	Cholinergic Receptor Nicotinic Alpha 7 Subunit	Protein Coding	46	GC15P031923	4.287807
TCF3	Transcription Factor 3	Protein Coding	46	GC19M001609	4.276465
MYH14	Myosin Heavy Chain 14	Protein Coding	46	GC19P050192	4.259545
CRHR1	Corticotropin Releasing Hormone Receptor 1	Protein Coding	46	GC17P045784	4.250362
EXTL3	Exostosin Like Glycosyltransferase 3	Protein Coding	46	GC08P028602	4.249753
CTSL	Cathepsin L	Protein Coding	46	GC09P087725	4.238457
PHKA2	Phosphorylase Kinase Regulatory Subunit Alpha 2	Protein Coding	46	GC0XM018892	4.237765
ACAN	Aggrecan	Protein Coding	46	GC15P088813	4.198064
LNPEP	Leucyl And Cystinyl Aminopeptidase	Protein Coding	46	GC05P096935	4.197493
PKM	Pyruvate Kinase M1/2	Protein Coding	46	GC15M072199	4.157477
ANXA11	Annexin A11	Protein Coding	46	GC10M080150	4.155983

CAST	Calpastatin	Protein Coding	46	GC05P096525	4.132545
PNPO	Pyridoxamine 5'-Phosphate Oxidase	Protein Coding	46	GC17P047941	4.089218
CD151	CD151 Molecule (Raph Blood Group)	Protein Coding	46	GC11P000996	4.050822
NDST1	N-Deacetylase And N-Sulfotransferase 1	Protein Coding	46	GC05P150484	4.005911
OXCT1	3-Oxoacid CoA-Transferase 1	Protein Coding	46	GC05M041732	3.947787
TBX21	T-Box Transcription Factor 21	Protein Coding	46	GC17P047733	3.945825
PML	PML Nuclear Body Scaffold	Protein Coding	46	GC15P073994	3.898292
EIF2AK2	Eukaryotic Translation Initiation Factor 2 Alpha Kinase 2	Protein Coding	46	GC02M037099	3.895568
SYNJ1	Synaptojanin 1	Protein Coding	46	GC21M032628	3.895071
APPL1	Adaptor Protein, Phosphotyrosine Interacting With PH Domain And Leucine Zipper 1	Protein Coding	46	GC03P057227	3.865093
CCNA2	Cyclin A2	Protein Coding	46	GC04M121816	3.849517
SHMT2	Serine Hydroxymethyltransferase 2	Protein Coding	46	GC12P057229	3.833076
GMNN	Geminin DNA Replication Inhibitor	Protein Coding	46	GC06P024779	3.826602
PTPRO	Protein Tyrosine Phosphatase Receptor Type O	Protein Coding	46	GC12P015366	3.822159
HEXA	Hexosaminidase Subunit Alpha	Protein Coding	46	GC15M072340	3.783107
BRD4	Bromodomain Containing 4	Protein Coding	46	GC19M015236	3.777796
IVD	Isovaleryl-CoA Dehydrogenase	Protein Coding	46	GC15P040405	3.697371
WNT10A	Wnt Family Member 10A	Protein Coding	46	GC02P218880	3.661196
CYP1A2	Cytochrome P450 Family 1 Subfamily A Member 2	Protein Coding	46	GC15P074748	3.657945
DDIT3	DNA Damage Inducible Transcript 3	Protein Coding	46	GC12M057516	3.595712
VAMP1	Vesicle Associated Membrane Protein 1	Protein Coding	46	GC12M006462	3.567033
ANGPTL3	Angiopoietin Like 3	Protein Coding	46	GC01P062597	3.562154
PTPRB	Protein Tyrosine Phosphatase Receptor Type B	Protein Coding	46	GC12M070516	3.540554
ZIC1	Zic Family Member 1	Protein Coding	46	GC03P147393	3.535031
C1QBP	Complement C1q Binding Protein	Protein Coding	46	GC17M005432	3.531815
NGFR	Nerve Growth Factor Receptor	Protein Coding	46	GC17P049495	3.503374
CNR2	Cannabinoid Receptor 2	Protein Coding	46	GC01M023870	3.493738
CTSS	Cathepsin S	Protein Coding	46	GC01M150730	3.483032
RAP1A	RAP1A, Member Of RAS Oncogene Family	Protein Coding	46	GC01P111542	3.482747
UROD	Uroporphyrinogen Decarboxylase	Protein Coding	46	GC01P045023	3.47099
PSTPIP1	Proline-Serine-Threonine Phosphatase Interacting Protein 1	Protein Coding	46	GC15P076993	3.466028
ERG	ETS Transcription Factor ERG	Protein Coding	46	GC21M038367	3.37681
MAD2L1	Mitotic Arrest Deficient 2 Like 1	Protein Coding	46	GC04M120055	3.350767
KCNQ3	Potassium Voltage-Gated Channel Subfamily Q Member 3	Protein Coding	46	GC08M132120	3.331957
PDE1C	Phosphodiesterase 1C	Protein Coding	46	GC07M031616	3.324602
TGFBI	Transforming Growth Factor Beta Induced	Protein Coding	46	GC05P136027	3.304483
WNT1	Wnt Family Member 1	Protein Coding	46	GC12P049053	3.299917
ABAT	4-Aminobutyrate Aminotransferase	Protein Coding	46	GC16P008674	3.290673
ATP2A3	ATPase Sarcoplasmic/Endoplasmic Reticulum Ca2+ Transporting 3	Protein Coding	46	GC17M003923	3.262992
CTNND1	Catenin Delta 1	Protein Coding	46	GC11P057879	3.219904
OPRK1	Opioid Receptor Kappa 1	Protein Coding	46	GC08M053227	3.209032
RAC3	Rac Family Small GTPase 3	Protein Coding	46	GC17P082031	3.201588
MGLL	Monoglyceride Lipase	Protein Coding	46	GC03M127689	3.189276
TUBA4A	Tubulin Alpha 4a	Protein Coding	46	GC02M219249	3.156755
EHMT2	Euchromatic Histone Lysine Methyltransferase 2	Protein Coding	46	GC06M031879	3.155353
PDE2A	Phosphodiesterase 2A	Protein Coding	46	GC11M072576	3.094387
PLOD2	Procollagen-Lysine,2-Oxoglutarate 5-Dioxygenase 2	Protein Coding	46	GC03M146069	3.080236
MAG	Myelin Associated Glycoprotein	Protein Coding	46	GC19P035292	3.080003
EPB41	Erythrocyte Membrane Protein Band 4.1	Protein Coding	46	GC01P028887	3.050008
SUOX	Sulfite Oxidase	Protein Coding	46	GC12P055997	2.982728
XPC	XPC Complex Subunit, DNA Damage Recognition And Repair Factor	Protein Coding	46	GC03M016919	2.97063
MCM5	Minichromosome Maintenance Complex Component 5	Protein Coding	46	GC22P035400	2.964579
IDH3A	Isocitrate Dehydrogenase (NAD(+)) 3 Catalytic Subunit Alpha	Protein Coding	46	GC15P078131	2.947232

PITX1	Paired Like Homeodomain 1	Protein Coding	46	GC05M135027	2.912439
PKN2	Protein Kinase N2	Protein Coding	46	GC01P088684	2.903992
RASGRP2	RAS Guanyl Releasing Protein 2	Protein Coding	46	GC11M064726	2.897502
HRH1	Histamine Receptor H1	Protein Coding	46	GC03P011113	2.881293
IL10RA	Interleukin 10 Receptor Subunit Alpha	Protein Coding	46	GC11P117987	2.842506
ANGPTL4	Angiopoietin Like 4	Protein Coding	46	GC19P008363	2.824604
TXN2	Thioredoxin 2	Protein Coding	46	GC22M036467	2.813764
PCBD1	Pterin-4 Alpha-Carbinolamine Dehydratase 1	Protein Coding	46	GC10M070882	2.777885
NNMT	Nicotinamide N-Methyltransferase	Protein Coding	46	GC11P114257	2.774283
RANBP2	RAN Binding Protein 2	Protein Coding	46	GC02P108719	2.773327
LPAR6	Lysophosphatidic Acid Receptor 6	Protein Coding	46	GC13M048389	2.768354
STUB1	STIP1 Homology And U-Box Containing Protein 1	Protein Coding	46	GC16P005516	2.749067
MOG	Myelin Oligodendrocyte Glycoprotein	Protein Coding	46	GC06P055140	2.730354
ARG2	Arginase 2	Protein Coding	46	GC14P067619	2.692625
CRK	CRK Proto-Oncogene, Adaptor Protein	Protein Coding	46	GC17M001420	2.657474
SHMT1	Serine Hydroxymethyltransferase 1	Protein Coding	46	GC17M021636	2.632243
MMP11	Matrix Metallopeptidase 11	Protein Coding	46	GC22P023768	2.632077
TAF1	TATA-Box Binding Protein Associated Factor 1	Protein Coding	46	GC0XP071366	2.626168
PDIA3	Protein Disulfide Isomerase Family A Member 3	Protein Coding	46	GC15P043746	2.625429
AP3B1	Adaptor Related Protein Complex 3 Subunit Beta 1	Protein Coding	46	GC05M078000	2.609557
GNMT	Glycine N-Methyltransferase	Protein Coding	46	GC06P042960	2.604076
GPD1	Glycerol-3-Phosphate Dehydrogenase 1	Protein Coding	46	GC12P050105	2.54952
SLC9A3R1	SLC9A3 Regulator 1	Protein Coding	46	GC17P074749	2.536881
PRCP	Prolylcarboxypeptidase	Protein Coding	46	GC11M082823	2.533404
PPP3R1	Protein Phosphatase 3 Regulatory Subunit B, Alpha	Protein Coding	46	GC02M068143	2.520869
RORC	RAR Related Orphan Receptor C	Protein Coding	46	GC01M151806	2.501188
DCX	Doublecortin	Protein Coding	46	GC0XM111293	2.476307
PPP1CC	Protein Phosphatase 1 Catalytic Subunit Gamma	Protein Coding	46	GC12M110709	2.470512
TXK	TXK Tyrosine Kinase	Protein Coding	46	GC04M048069	2.453471
NFS1	NFS1 Cysteine Desulfurase	Protein Coding	46	GC20M035668	2.446653
KPNA2	Karyopherin Subunit Alpha 2	Protein Coding	46	GC17P068035	2.412676
COASY	Coenzyme A Synthase	Protein Coding	46	GC17P042561	2.363975
PFKP	Phosphofructokinase, Platelet	Protein Coding	46	GC10P003066	2.328373
E2F4	E2F Transcription Factor 4	Protein Coding	46	GC16P067192	2.31863
GALNT3	Polypeptide N-Acetylgalactosaminyltransferase 3	Protein Coding	46	GC02M165747	2.315941
HRH4	Histamine Receptor H4	Protein Coding	46	GC18P024460	2.309897
PXN	Paxillin	Protein Coding	46	GC12M120210	2.302793
EZR	Ezrin	Protein Coding	46	GC06M158765	2.289537
AICDA	Activation Induced Cytidine Deaminase	Protein Coding	46	GC12M008602	2.253651
SERPINI1	Serpin Family I Member 1	Protein Coding	46	GC03P167735	2.237568
HSP90AB1	Heat Shock Protein 90 Alpha Family Class B Member 1	Protein Coding	46	GC06P044246	2.214912
SSTR3	Somatostatin Receptor 3	Protein Coding	46	GC22M037204	2.135761
PSMB10	Proteasome 20S Subunit Beta 10	Protein Coding	46	GC16M067937	2.129506
MMP15	Matrix Metallopeptidase 15	Protein Coding	46	GC16P058025	2.099596
LSS	Lanosterol Synthase	Protein Coding	46	GC21M048323	2.091296
TUBB2A	Tubulin Beta 2A Class IIa	Protein Coding	46	GC06M003153	2.066672
GSTM3	Glutathione S-Transferase Mu 3	Protein Coding	46	GC01M109733	2.004937
SLC5A5	Solute Carrier Family 5 Member 5	Protein Coding	46	GC19P026646	2.003986
LATS1	Large Tumor Suppressor Kinase 1	Protein Coding	46	GC06M149658	1.990371
BPGM	Bisphosphoglycerate Mutase	Protein Coding	46	GC07P134646	1.986181
DGKB	Diacylglycerol Kinase Beta	Protein Coding	46	GC07M014145	1.977013
CISH	Cytokine Inducible SH2 Containing Protein	Protein Coding	46	GC03M050618	1.952833
IKBKE	Inhibitor Of Nuclear Factor Kappa B Kinase Subunit Epsilon	Protein Coding	46	GC01P206470	1.904228

NR2F1	Nuclear Receptor Subfamily 2 Group F Member 1	Protein Coding	46	GC05P093583	1.865056
TRPA1	Transient Receptor Potential Cation Channel Subfamily A Member 1	Protein Coding	46	GC08M072019	1.853898
CD22	CD22 Molecule	Protein Coding	46	GC19P035319	1.850138
DPYSL2	Dihydropyrimidinase Like 2	Protein Coding	46	GC08P026514	1.84043
LMAN1	Lectin, Mannose Binding 1	Protein Coding	46	GC18M059327	1.811803
ELOVL4	ELOVL Fatty Acid Elongase 4	Protein Coding	46	GC06M079914	1.798617
PHKB	Phosphorylase Kinase Regulatory Subunit Beta	Protein Coding	46	GC16P047436	1.791662
LATS2	Large Tumor Suppressor Kinase 2	Protein Coding	46	GC13M020973	1.756257
CTSC	Cathepsin C	Protein Coding	46	GC11M088211	1.72269
BCAT2	Branched Chain Amino Acid Transaminase 2	Protein Coding	46	GC19M048795	1.71107
BIRC2	Baculoviral IAP Repeat Containing 2	Protein Coding	46	GC11P102347	1.700933
EPS15	Epidermal Growth Factor Receptor Pathway Substrate 15	Protein Coding	46	GC01M051354	1.651404
OAS1	2'-5'-Oligoadenylate Synthetase 1	Protein Coding	46	GC12P112911	1.638536
FDPS	Farnesyl Diphosphate Synthase	Protein Coding	46	GC01P155308	1.587713
SLC27A2	Solute Carrier Family 27 Member 2	Protein Coding	46	GC15P050182	1.549622
TP73	Tumor Protein P73	Protein Coding	46	GC01P003652	1.539093
UBE2D1	Ubiquitin Conjugating Enzyme E2 D1	Protein Coding	46	GC10P058334	1.529994
ALDH1A3	Aldehyde Dehydrogenase 1 Family Member A3	Protein Coding	46	GC15P100877	1.438922
DOCK1	Dedicator Of Cytokinesis 1	Protein Coding	46	GC10P126905	1.410098
INPP5D	Inositol Polyphosphate-5-Phosphatase D	Protein Coding	46	GC02P233059	1.397936
EPS8	Epidermal Growth Factor Receptor Pathway Substrate 8	Protein Coding	46	GC12M015765	1.364397
RALA	RAS Like Proto-Oncogene A	Protein Coding	46	GC07P039622	1.358989
TEC	Tec Protein Tyrosine Kinase	Protein Coding	46	GC04M048137	1.336054
NQO2	N-Ribosyldihydronicotinamide:Quinone Reductase 2	Protein Coding	46	GC06P003014	1.299207
SAT1	Spermidine/Spermine N1-Acetyltransferase 1	Protein Coding	46	GC0XP023784	1.294487
MCM3	Minichromosome Maintenance Complex Component 3	Protein Coding	46	GC06M052264	1.291953
TLK2	Tousled Like Kinase 2	Protein Coding	46	GC17P062458	1.290613
SRPK1	SRSF Protein Kinase 1	Protein Coding	46	GC06M047030	1.267817
CNTN2	Contactin 2	Protein Coding	46	GC01P205043	1.264003
MATK	Megakaryocyte-Associated Tyrosine Kinase	Protein Coding	46	GC19M003777	1.263471
MVD	Mevalonate Diphosphate Decarboxylase	Protein Coding	46	GC16M088651	1.243696
UBE2B	Ubiquitin Conjugating Enzyme E2 B	Protein Coding	46	GC05P134371	1.238186
MAP3K12	Mitogen-Activated Protein Kinase Kinase Kinase 12	Protein Coding	46	GC12M053479	1.214028
PPP4C	Protein Phosphatase 4 Catalytic Subunit	Protein Coding	46	GC16P032401	1.211264
EIF2S3	Eukaryotic Translation Initiation Factor 2 Subunit Gamma	Protein Coding	46	GC0XP024054	1.177309
ABL2	ABL Proto-Oncogene 2, Non-Receptor Tyrosine Kinase	Protein Coding	46	GC01M179114	1.126846
GNAL	G Protein Subunit Alpha L	Protein Coding	46	GC18P011689	1.023819
MELK	Maternal Embryonic Leucine Zipper Kinase	Protein Coding	46	GC09P036572	1.023377
PRKD3	Protein Kinase D3	Protein Coding	46	GC02M037251	0.973076
ZYX	Zyxin	Protein Coding	46	GC07P143381	0.967557
CDK5R1	Cyclin Dependent Kinase 5 Regulatory Subunit 1	Protein Coding	46	GC17P032486	0.940822
EPHA8	EPH Receptor A8	Protein Coding	46	GC01P022563	0.806229
TRPV5	Transient Receptor Potential Cation Channel Subfamily V Member 5	Protein Coding	46	GC07M142908	0.803497
IDH3B	Isocitrate Dehydrogenase (NAD(+)) 3 Non-Catalytic Subunit Beta	Protein Coding	46	GC20M002658	0.491036
TNK2	Tyrosine Kinase Non Receptor 2	Protein Coding	46	GC03M195863	0.444722
NKX2-5	NK2 Homeobox 5	Protein Coding	45	GC05M173232	79.27037
HAND2	Heart And Neural Crest Derivatives Expressed 2	Protein Coding	45	GC04M173524	46.32991
PLN	Phospholamban	Protein Coding	45	GC06P118548	39.29464
SCN1B	Sodium Voltage-Gated Channel Beta Subunit 1	Protein Coding	45	GC19P035030	32.63956
FLNC	Filamin C	Protein Coding	45	GC07P128830	30.29327
MYL3	Myosin Light Chain 3	Protein Coding	45	GC03M046836	28.72418
ELN	Elastin	Protein Coding	45	GC07P074027	28.27973
FMR1	FMRP Translational Regulator 1	Protein Coding	45	GC0XP147925	27.41976

ABCC9	ATP Binding Cassette Subfamily C Member 9	Protein Coding	45	GC12M021797	25.30178
KCNE1	Potassium Voltage-Gated Channel Subfamily E Regulatory Subunit 1	Protein Coding	45	GC21M034446	25.14377
LAMP2	Lysosomal Associated Membrane Protein 2	Protein Coding	45	GC0XM120426	25.0331
BAG3	BAG Cochaperone 3	Protein Coding	45	GC10P119651	24.51595
ADM	Adrenomedullin	Protein Coding	45	GC11P010304	23.96802
B3GAT3	Beta-1,3-Glucuronyltransferase 3	Protein Coding	45	GC11M069250	23.71751
ADIPOQ	Adiponectin, C1Q And Collagen Domain Containing	Protein Coding	45	GC03P186842	22.6354
CORIN	Corin, Serine Peptidase	Protein Coding	45	GC04M047596	22.40737
MTM1	Myotubularin 1	Protein Coding	45	GC0XP150562	22.01797
CST3	Cystatin C	Protein Coding	45	GC20M023627	20.20129
SLC8A1	Solute Carrier Family 8 Member A1	Protein Coding	45	GC02M040078	19.95959
ADCY10	Adenylate Cyclase 10	Protein Coding	45	GC01M167809	19.94028
PRDM16	PR/SET Domain 16	Protein Coding	45	GC01P003068	19.80852
MYL4	Myosin Light Chain 4	Protein Coding	45	GC17P047189	19.77957
F3	Coagulation Factor III, Tissue Factor	Protein Coding	45	GC01M094530	19.7302
CRYAB	Crystallin Alpha B	Protein Coding	45	GC11M111908	19.57586
F5	Coagulation Factor V	Protein Coding	45	GC01M169511	19.53042
SLC17A5	Solute Carrier Family 17 Member 5	Protein Coding	45	GC06M073593	19.50614
KNG1	Kininogen 1	Protein Coding	45	GC03P186717	19.4918
LGALS3	Galectin 3	Protein Coding	45	GC14P055124	19.22866
CASQ2	Calsequestrin 2	Protein Coding	45	GC01M115700	19.17604
CX3CR1	C-X3-C Motif Chemokine Receptor 1	Protein Coding	45	GC03M039279	19.15659
HP	Haptoglobin	Protein Coding	45	GC16P072089	18.2248
EMD	Emerin	Protein Coding	45	GC0XP154379	17.85803
TIMP1	TIMP Metallopeptidase Inhibitor 1	Protein Coding	45	GC0XP047583	17.8125
BCS1L	BCS1 Homolog, Ubiquinol-Cytochrome C Reductase Complex Chaperone	Protein Coding	45	GC02P218658	17.2208
PDE5A	Phosphodiesterase 5A	Protein Coding	45	GC04M119494	17.10017
LOX	Lysyl Oxidase	Protein Coding	45	GC05M122063	16.81926
TBX2	T-Box Transcription Factor 2	Protein Coding	45	GC17P061399	16.37494
RBP4	Retinol Binding Protein 4	Protein Coding	45	GC10M093591	16.27862
SCO2	Synthesis Of Cytochrome C Oxidase 2	Protein Coding	45	GC22M050523	16.09343
IL2	Interleukin 2	Protein Coding	45	GC04M122451	16.07765
FOXC2	Forkhead Box C2	Protein Coding	45	GC16P086570	16.05221
SALL1	Spalt Like Transcription Factor 1	Protein Coding	45	GC16M051135	15.92782
IGFBP3	Insulin Like Growth Factor Binding Protein 3	Protein Coding	45	GC07M045912	15.84182
KCNJ8	Potassium Inwardly Rectifying Channel Subfamily J Member 8	Protein Coding	45	GC12M021764	15.81861
COX5A	Cytochrome C Oxidase Subunit 5A	Protein Coding	45	GC15M074919	15.23297
NDUFS4	NADH:Ubiquinone Oxidoreductase Subunit S4	Protein Coding	45	GC05P053560	15.19075
SDHD	Succinate Dehydrogenase Complex Subunit D	Protein Coding	45	GC11P112087	14.92268
INVS	Inversin	Protein Coding	45	GC09P100099	14.75344
TFAP2B	Transcription Factor AP-2 Beta	Protein Coding	45	GC06P055469	14.56769
CALM1	Calmodulin 1	Protein Coding	45	GC14P090396	14.44654
GBE1	1,4-Alpha-Glucan Branching Enzyme 1	Protein Coding	45	GC03M081489	14.35983
ARID1B	AT-Rich Interaction Domain 1B	Protein Coding	45	GC06P156777	14.25991
SEMA3A	Semaphorin 3A	Protein Coding	45	GC07M083955	13.92901
SLC2A10	Solute Carrier Family 2 Member 10	Protein Coding	45	GC20P046709	13.8206
SLC34A1	Solute Carrier Family 34 Member 1	Protein Coding	45	GC05P177380	13.62168
CS	Citrate Synthase	Protein Coding	45	GC12M056271	13.4312
TET2	Tet Methylcytosine Dioxygenase 2	Protein Coding	45	GC04P105145	13.42197
TMPO	Thymopoietin	Protein Coding	45	GC12P098515	13.22703
COL4A3	Collagen Type IV Alpha 3 Chain	Protein Coding	45	GC02P227164	13.0337
ROBO1	Roundabout Guidance Receptor 1	Protein Coding	45	GC03M078597	12.96709
TFR2	Transferrin Receptor 2	Protein Coding	45	GC07M100620	12.77126

PRF1	Perforin 1	Protein Coding	45	GC10M070597	12.64854
ADD1	Adducin 1	Protein Coding	45	GC04P002855	12.63436
SNTA1	Syntrophin Alpha 1	Protein Coding	45	GC20M033407	12.4943
NDUFS2	NADH:Ubiquinone Oxidoreductase Core Subunit S2	Protein Coding	45	GC01P161197	12.3952
NEU1	Neuraminidase 1	Protein Coding	45	GC06M031857	12.2967
NDUFA13	NADH:Ubiquinone Oxidoreductase Subunit A13	Protein Coding	45	GC19P019515	12.17236
CXCL10	C-X-C Motif Chemokine Ligand 10	Protein Coding	45	GC04M076021	12.15899
CKM	Creatine Kinase, M-Type	Protein Coding	45	GC19M045306	12.13726
LARS2	Leucyl-TRNA Synthetase 2, Mitochondrial	Protein Coding	45	GC03P045606	12.12394
STAG2	Stromal Antigen 2	Protein Coding	45	GC0XP123960	12.04041
MTTP	Microsomal Triglyceride Transfer Protein	Protein Coding	45	GC04P099563	12.00635
NDUFA12	NADH:Ubiquinone Oxidoreductase Subunit A12	Protein Coding	45	GC12M094898	11.98533
THBS1	Thrombospondin 1	Protein Coding	45	GC15P039581	11.89482
FBLN5	Fibulin 5	Protein Coding	45	GC14M091869	11.80904
COL11A2	Collagen Type XI Alpha 2 Chain	Protein Coding	45	GC06M033162	11.75403
PEX1	Peroxisomal Biogenesis Factor 1	Protein Coding	45	GC07M092487	11.74912
SCN4A	Sodium Voltage-Gated Channel Alpha Subunit 4	Protein Coding	45	GC17M063938	11.55047
CCR6	C-C Motif Chemokine Receptor 6	Protein Coding	45	GC06P167111	11.54095
SGSH	N-Sulfoglucosamine Sulfohydrolase	Protein Coding	45	GC17M080206	11.50951
IL13	Interleukin 13	Protein Coding	45	GC05P132656	11.47859
KMT2A	Lysine Methyltransferase 2A	Protein Coding	45	GC11P118436	11.45941
KCNE3	Potassium Voltage-Gated Channel Subfamily E Regulatory Subunit 3	Protein Coding	45	GC11M074454	11.41014
ABCG5	ATP Binding Cassette Subfamily G Member 5	Protein Coding	45	GC02M043806	11.14907
SKI	SKI Proto-Oncogene	Protein Coding	45	GC01P002228	11.14833
CALM2	Calmodulin 2	Protein Coding	45	GC02M047124	11.01468
MED12	Mediator Complex Subunit 12	Protein Coding	45	GC0XP071118	11.00834
PNPLA2	Patatin Like Phospholipase Domain Containing 2	Protein Coding	45	GC11P000994	10.98077
CDON	Cell Adhesion Associated, Oncogene Regulated	Protein Coding	45	GC11M125955	10.96308
RFC2	Replication Factor C Subunit 2	Protein Coding	45	GC07M074231	10.88456
NUP155	Nucleoporin 155	Protein Coding	45	GC05M037288	10.81992
WRN	WRN RecQ Like Helicase	Protein Coding	45	GC08P031033	10.81616
AGER	Advanced Glycosylation End-Product Specific Receptor	Protein Coding	45	GC06M032180	10.80636
PIK3C2G	Phosphatidylinositol-4-Phosphate 3-Kinase Catalytic Subunit Type 2 Gamma	Protein Coding	45	GC12P018242	10.74343
APOA5	Apolipoprotein A5	Protein Coding	45	GC11M116789	10.70161
NPHS1	NPHS1 Adhesion Molecule, Nephrin	Protein Coding	45	GC19M035825	10.59511
NONO	Non-POU Domain Containing Octamer Binding	Protein Coding	45	GC0XP071255	10.54376
DGUOK	Deoxyguanosine Kinase	Protein Coding	45	GC02P073926	10.51555
NDUFA10	NADH:Ubiquinone Oxidoreductase Subunit A10	Protein Coding	45	GC02M239893	10.4068
TCIRG1	T Cell Immune Regulator 1, ATPase H+ Transporting V0 Subunit A3	Protein Coding	45	GC11P068038	10.28549
TFAM	Transcription Factor A, Mitochondrial	Protein Coding	45	GC10P058385	10.17692
RPS10	Ribosomal Protein S10	Protein Coding	45	GC06M047017	10.16483
SEC24C	SEC24 Homolog C, COPII Coat Complex Component	Protein Coding	45	GC10P073744	10.07228
MSTN	Myostatin	Protein Coding	45	GC02M190055	10.02384
GALNS	Galactosamine (N-Acetyl)-6-Sulfatase	Protein Coding	45	GC16M088813	9.963951
LAMA4	Laminin Subunit Alpha 4	Protein Coding	45	GC06M112107	9.900773
NHP2	NHP2 Ribonucleoprotein	Protein Coding	45	GC05M178149	9.845206
OPA1	OPA1 Mitochondrial Dynamin Like GTPase	Protein Coding	45	GC03P193594	9.723253
ETFDH	Electron Transfer Flavoprotein Dehydrogenase	Protein Coding	45	GC04P158672	9.699948
SNRPN	Small Nuclear Ribonucleoprotein Polypeptide N	Protein Coding	45	GC15P024823	9.652233
YARS2	Tyrosyl-TRNA Synthetase 2	Protein Coding	45	GC12M032725	9.512262
ATP6V0A2	ATPase H+ Transporting V0 Subunit A2	Protein Coding	45	GC12P123712	9.486324
MDM4	MDM4 Regulator Of P53	Protein Coding	45	GC01P204516	9.453314
FTO	FTO Alpha-Ketoglutarate Dependent Dioxygenase	Protein Coding	45	GC16P053737	9.447497

NFATC4	Nuclear Factor Of Activated T Cells 4	Protein Coding	45	GC14P024365	9.423514
DLK1	Delta Like Non-Canonical Notch Ligand 1	Protein Coding	45	GC14P106102	9.41166
ASCL1	Achaete-Scute Family BHLH Transcription Factor 1	Protein Coding	45	GC12P102957	9.375169
MAD2L2	Mitotic Arrest Deficient 2 Like 2	Protein Coding	45	GC01M011674	9.369765
KIF1B	Kinesin Family Member 1B	Protein Coding	45	GC01P010210	9.338696
NDUFA6	NADH:Ubiquinone Oxidoreductase Subunit A6	Protein Coding	45	GC22M042085	9.314459
NDUFA1	NADH:Ubiquinone Oxidoreductase Subunit A1	Protein Coding	45	GC0XP119871	9.304491
ARID1A	AT-Rich Interaction Domain 1A	Protein Coding	45	GC01P026693	9.282681
HMGB1	High Mobility Group Box 1	Protein Coding	45	GC13M030456	9.270056
MTHFD1	Methylenetetrahydrofolate Dehydrogenase, Cyclohydrolase And Formyltetrahydrofolate Synthetase 1	Protein Coding	45	GC14P064388	9.235138
SLC25A3	Solute Carrier Family 25 Member 3	Protein Coding	45	GC12P098593	9.180526
AGL	Amylo-Alpha-1, 6-Glucosidase, 4-Alpha-Glucanotransferase	Protein Coding	45	GC01P099850	9.166743
CTNS	Cystinosin, Lysosomal Cystine Transporter	Protein Coding	45	GC17P003636	9.125908
KITLG	KIT Ligand	Protein Coding	45	GC12M088492	9.113304
PRTN3	Proteinase 3	Protein Coding	45	GC19P000840	9.100677
CLCN1	Chloride Voltage-Gated Channel 1	Protein Coding	45	GC07P143316	9.073758
NPR3	Natriuretic Peptide Receptor 3	Protein Coding	45	GC05P032689	9.06879
EIF2B2	Eukaryotic Translation Initiation Factor 2B Subunit Beta	Protein Coding	45	GC14P075002	9.057726
KCNN2	Potassium Calcium-Activated Channel Subfamily N Member 2	Protein Coding	45	GC05P114058	9.05526
RPL15	Ribosomal Protein L15	Protein Coding	45	GC03P023916	8.98769
GUCY2C	Guanylate Cyclase 2C	Protein Coding	45	GC12M014612	8.954433
PDE4A	Phosphodiesterase 4A	Protein Coding	45	GC19P010416	8.927561
RPS20	Ribosomal Protein S20	Protein Coding	45	GC08M056067	8.765656
NDUFV2	NADH:Ubiquinone Oxidoreductase Core Subunit V2	Protein Coding	45	GC18P009092	8.702698
ATP6AP2	ATPase H+ Transporting Accessory Protein 2	Protein Coding	45	GC0XP040582	8.649763
CR2	Complement C3d Receptor 2	Protein Coding	45	GC01P207454	8.644072
IL5	Interleukin 5	Protein Coding	45	GC05M132541	8.482678
RPL35A	Ribosomal Protein L35a	Protein Coding	45	GC03P197949	8.351566
EIF2AK4	Eukaryotic Translation Initiation Factor 2 Alpha Kinase 4	Protein Coding	45	GC15P039934	8.277233
SDHC	Succinate Dehydrogenase Complex Subunit C	Protein Coding	45	GC01P161314	8.262216
CALCRL	Calcitonin Receptor Like Receptor	Protein Coding	45	GC02M187341	8.204147
KCND2	Potassium Voltage-Gated Channel Subfamily D Member 2	Protein Coding	45	GC07P120273	8.194077
LAMA3	Laminin Subunit Alpha 3	Protein Coding	45	GC18P023689	8.169756
NDUFS6	NADH:Ubiquinone Oxidoreductase Subunit S6	Protein Coding	45	GC05P001801	8.146679
KCNN3	Potassium Calcium-Activated Channel Subfamily N Member 3	Protein Coding	45	GC01M154697	8.120504
OCRL	OCRL Inositol Polyphosphate-5-Phosphatase	Protein Coding	45	GC0XP129539	8.031328
LAMB2	Laminin Subunit Beta 2	Protein Coding	45	GC03M049121	8.020464
COL18A1	Collagen Type XVIII Alpha 1 Chain	Protein Coding	45	GC21P045405	8.01742
ETFB	Electron Transfer Flavoprotein Subunit Beta	Protein Coding	45	GC19M051345	8.007357
VIP	Vasoactive Intestinal Peptide	Protein Coding	45	GC06P152750	7.998825
GFM1	G Elongation Factor Mitochondrial 1	Protein Coding	45	GC03P158644	7.942462
PDE4B	Phosphodiesterase 4B	Protein Coding	45	GC01P065792	7.902467
CD14	CD14 Molecule	Protein Coding	45	GC05M140631	7.849314
HLA-G	Major Histocompatibility Complex, Class I, G	Protein Coding	45	GC06P055150	7.84232
CIITA	Class II Major Histocompatibility Complex Transactivator	Protein Coding	45	GC16P010879	7.802941
NDUFB9	NADH:Ubiquinone Oxidoreductase Subunit B9	Protein Coding	45	GC08P124539	7.775831
ATP6V1A	ATPase H+ Transporting V1 Subunit A	Protein Coding	45	GC03P113747	7.732832
HGD	Homogentisate 1,2-Dioxygenase	Protein Coding	45	GC03M120628	7.72481
SOX3	SRY-Box Transcription Factor 3	Protein Coding	45	GC0XM140502	7.712125
SAR1B	Secretion Associated Ras Related GTPase 1B	Protein Coding	45	GC05M134601	7.693676
KISS1R	KISS1 Receptor	Protein Coding	45	GC19P000917	7.684187
ERCC5	ERCC Excision Repair 5, Endonuclease	Protein Coding	45	GC13P102845	7.681026
CHD4	Chromodomain Helicase DNA Binding Protein 4	Protein Coding	45	GC12M006570	7.612814

ADAMTS1	ADAM Metallopeptidase With Thrombospondin Type 1 Motif 1	Protein Coding	45	GC21M026835	7.606214
PON3	Paraoxonase 3	Protein Coding	45	GC07M095359	7.589148
PAFAH1B1	Platelet Activating Factor Acetylhydrolase 1b Regulatory Subunit 1	Protein Coding	45	GC17P002593	7.587348
HMGA2	High Mobility Group AT-Hook 2	Protein Coding	45	GC12P065824	7.504805
BDKRB2	Bradykinin Receptor B2	Protein Coding	45	GC14P096205	7.475325
EFEMP1	EGF Containing Fibulin Extracellular Matrix Protein 1	Protein Coding	45	GC02M055865	7.422776
COL7A1	Collagen Type VII Alpha 1 Chain	Protein Coding	45	GC03M048564	7.416198
PLAUR	Plasminogen Activator, Urokinase Receptor	Protein Coding	45	GC19M043646	7.415114
CTSA	Cathepsin A	Protein Coding	45	GC20P045890	7.394985
HLA-C	Major Histocompatibility Complex, Class I, C	Protein Coding	45	GC06M046831	7.379174
AFG3L2	AFG3 Like Matrix AAA Peptidase Subunit 2	Protein Coding	45	GC18M012328	7.351159
CYP2J2	Cytochrome P450 Family 2 Subfamily J Member 2	Protein Coding	45	GC01M059893	7.329762
BAAT	Bile Acid-CoA:Amino Acid N-Acyltransferase	Protein Coding	45	GC09M101354	7.286491
GANAB	Glucosidase II Alpha Subunit	Protein Coding	45	GC11M069251	7.284859
CLN3	CLN3 Lysosomal/Endosomal Transmembrane Protein, Battenin	Protein Coding	45	GC16M028466	7.26825
WNT7B	Wnt Family Member 7B	Protein Coding	45	GC22M045920	7.260849
CYP21A2	Cytochrome P450 Family 21 Subfamily A Member 2	Protein Coding	45	GC06P055225	7.158361
SPTA1	Spectrin Alpha, Erythrocytic 1	Protein Coding	45	GC01M158610	7.111533
RPL18	Ribosomal Protein L18	Protein Coding	45	GC19M048615	7.100278
CCR2	C-C Motif Chemokine Receptor 2	Protein Coding	45	GC03P046356	7.082628
FARSB	Phenylalanyl-TRNA Synthetase Subunit Beta	Protein Coding	45	GC02M222570	7.059417
IRS2	Insulin Receptor Substrate 2	Protein Coding	45	GC13M109752	7.045216
MAN2B1	Mannosidase Alpha Class 2B Member 1	Protein Coding	45	GC19M012663	7.03054
CDC45	Cell Division Cycle 45	Protein Coding	45	GC22P019479	6.969818
MAF	MAF BZIP Transcription Factor	Protein Coding	45	GC16M079204	6.941296
ALOX5AP	Arachidonate 5-Lipoxygenase Activating Protein	Protein Coding	45	GC13P030713	6.859024
POLR1D	RNA Polymerase I And III Subunit D	Protein Coding	45	GC13P027620	6.810308
SYNGAP1	Synaptic Ras GTPase Activating Protein 1	Protein Coding	45	GC06P033419	6.744759
DYNC1H1	Dynein Cytoplasmic 1 Heavy Chain 1	Protein Coding	45	GC14P106032	6.692193
IL23R	Interleukin 23 Receptor	Protein Coding	45	GC01P067138	6.67076
RTN4	Reticulon 4	Protein Coding	45	GC02M054934	6.645849
PIGA	Phosphatidylinositol Glycan Anchor Biosynthesis Class A	Protein Coding	45	GC0XM015319	6.627389
GNB5	G Protein Subunit Beta 5	Protein Coding	45	GC15M067468	6.591752
CCR1	C-C Motif Chemokine Receptor 1	Protein Coding	45	GC03M046218	6.575223
POLR2A	RNA Polymerase II Subunit A	Protein Coding	45	GC17P009102	6.565845
ACAD8	Acyl-CoA Dehydrogenase Family Member 8	Protein Coding	45	GC11P134253	6.521083
APEX1	Apurinic/Apyrimidinic Endodeoxyribonuclease 1	Protein Coding	45	GC14P020455	6.470955
AGRN	Agrin	Protein Coding	45	GC01P001020	6.462268
ADAR	Adenosine Deaminase RNA Specific	Protein Coding	45	GC01M154582	6.460384
SF3B1	Splicing Factor 3b Subunit 1	Protein Coding	45	GC02M197393	6.435423
FADS2	Fatty Acid Desaturase 2	Protein Coding	45	GC11P061792	6.400865
MBP	Myelin Basic Protein	Protein Coding	45	GC18M076978	6.380712
UCP1	Uncoupling Protein 1	Protein Coding	45	GC04M140559	6.317394
CLPP	Caseinolytic Mitochondrial Matrix Peptidase Proteolytic Subunit	Protein Coding	45	GC19P006443	6.28265
TFPI	Tissue Factor Pathway Inhibitor	Protein Coding	45	GC02M187464	6.253784
FUS	FUS RNA Binding Protein	Protein Coding	45	GC16P031180	6.217331
EIF2B4	Eukaryotic Translation Initiation Factor 2B Subunit Delta	Protein Coding	45	GC02M027364	6.216559
PTGDS	Prostaglandin D2 Synthase	Protein Coding	45	GC09P137005	6.201519
BCL6	BCL6 Transcription Repressor	Protein Coding	45	GC03M187721	6.187384
SLC26A2	Solute Carrier Family 26 Member 2	Protein Coding	45	GC05P149944	6.151445
DCLRE1C	DNA Cross-Link Repair 1C	Protein Coding	45	GC10M014897	6.116423
IL12RB1	Interleukin 12 Receptor Subunit Beta 1	Protein Coding	45	GC19M018058	6.10958
APTX	Aprataxin	Protein Coding	45	GC09M032886	6.085224

RPL35	Ribosomal Protein L35	Protein Coding	45	GC09M124857	6.064148
FABP4	Fatty Acid Binding Protein 4	Protein Coding	45	GC08M081478	6.027002
SLC10A1	Solute Carrier Family 10 Member 1	Protein Coding	45	GC14M069775	5.968276
APOC2	Apolipoprotein C2	Protein Coding	45	GC19P044945	5.946964
PTCH2	Patched 2	Protein Coding	45	GC01M044819	5.887856
ABCD3	ATP Binding Cassette Subfamily D Member 3	Protein Coding	45	GC01P094418	5.876541
CYP4F2	Cytochrome P450 Family 4 Subfamily F Member 2	Protein Coding	45	GC19M015878	5.871181
MPI	Mannose Phosphate Isomerase	Protein Coding	45	GC15P074890	5.866132
ABHD5	Abhydrolase Domain Containing 5, Lysophosphatidic Acid Acyltransferase	Protein Coding	45	GC03P043707	5.861193
CYB5R3	Cytochrome B5 Reductase 3	Protein Coding	45	GC22M042617	5.840148
KLF5	Kruppel Like Factor 5	Protein Coding	45	GC13P073054	5.838417
PPP3CB	Protein Phosphatase 3 Catalytic Subunit Beta	Protein Coding	45	GC10M073436	5.818837
DKK1	Dickkopf WNT Signaling Pathway Inhibitor 1	Protein Coding	45	GC10P052314	5.808104
SPRY4	Sprouty RTK Signaling Antagonist 4	Protein Coding	45	GC05M142310	5.779898
SP1	Sp1 Transcription Factor	Protein Coding	45	GC12P053380	5.775655
OPTN	Optineurin	Protein Coding	45	GC10P013099	5.770185
SIX1	SIX Homeobox 1	Protein Coding	45	GC14M060643	5.699445
CTSG	Cathepsin G	Protein Coding	45	GC14M024573	5.679005
HRH2	Histamine Receptor H2	Protein Coding	45	GC05P175659	5.664162
TLR9	Toll Like Receptor 9	Protein Coding	45	GC03M052222	5.651963
ACSL4	Acyl-CoA Synthetase Long Chain Family Member 4	Protein Coding	45	GC0XM109624	5.651822
CPT1B	Carnitine Palmitoyltransferase 1B	Protein Coding	45	GC22M050569	5.567232
DPM1	Dolichyl-Phosphate Mannosyltransferase Subunit 1, Catalytic	Protein Coding	45	GC20M050934	5.56511
MMP12	Matrix Metallopeptidase 12	Protein Coding	45	GC11M102862	5.559022
WNT11	Wnt Family Member 11	Protein Coding	45	GC11M076186	5.551665
ADIPOR1	Adiponectin Receptor 1	Protein Coding	45	GC01M202940	5.531767
RBBP8	RB Binding Protein 8, Endonuclease	Protein Coding	45	GC18P022798	5.515396
CD274	CD274 Molecule	Protein Coding	45	GC09P005450	5.47959
GAL	Galanin And GMAP Prepropeptide	Protein Coding	45	GC11P068684	5.479414
SOCS3	Suppressor Of Cytokine Signaling 3	Protein Coding	45	GC17M078356	5.469984
CHRNB1	Cholinergic Receptor Nicotinic Beta 1 Subunit	Protein Coding	45	GC17P009098	5.389036
CXCR3	C-X-C Motif Chemokine Receptor 3	Protein Coding	45	GC0XM071615	5.377531
SNRPB	Small Nuclear Ribonucleoprotein Polypeptides B And B1	Protein Coding	45	GC20M002461	5.377384
CRAT	Carnitine O-Acetyltransferase	Protein Coding	45	GC09M129094	5.307362
HDC	Histidine Decarboxylase	Protein Coding	45	GC15M050241	5.282324
AIP	Aryl Hydrocarbon Receptor Interacting Protein	Protein Coding	45	GC11P067468	5.250143
HERC2	HECT And RLD Domain Containing E3 Ubiquitin Protein Ligase 2	Protein Coding	45	GC15M028111	5.247075
AIRE	Autoimmune Regulator	Protein Coding	45	GC21P044285	5.23963
CANT1	Calcium Activated Nucleotidase 1	Protein Coding	45	GC17M078992	5.235817
EHHADH	Enoyl-CoA Hydratase And 3-Hydroxyacyl CoA Dehydrogenase	Protein Coding	45	GC03M185190	5.218724
NFATC2	Nuclear Factor Of Activated T Cells 2	Protein Coding	45	GC20M051386	5.175163
HMBS	Hydroxymethylbilane Synthase	Protein Coding	45	GC11P119084	5.150557
VEGFB	Vascular Endothelial Growth Factor B	Protein Coding	45	GC11P064234	5.100254
PGAM2	Phosphoglycerate Mutase 2	Protein Coding	45	GC07M044062	5.092428
XRCC5	X-Ray Repair Cross Complementing 5	Protein Coding	45	GC02P216107	5.075332
FCGR3A	Fc Fragment Of IgG Receptor IIIa	Protein Coding	45	GC01M161541	5.071359
ADCY9	Adenylate Cyclase 9	Protein Coding	45	GC16M003953	5.042799
PRMT7	Protein Arginine Methyltransferase 7	Protein Coding	45	GC16P068363	5.038512
GALE	UDP-Galactose-4-Epimerase	Protein Coding	45	GC01M023795	5.037467
ATP6V1B1	ATPase H+ Transporting V1 Subunit B1	Protein Coding	45	GC02P070935	5.003951
FHIT	Fragile Histidine Triad Diadenosine Triphosphatase	Protein Coding	45	GC03M059747	5.001762
DPAGT1	Dolichyl-Phosphate N-Acetylglucosaminephosphotransferase 1	Protein Coding	45	GC11M119096	4.989464
GP6	Glycoprotein VI Platelet	Protein Coding	45	GC19M055013	4.982726

DDOST	Dolichyl-Diphosphooligosaccharide--Protein Glycosyltransferase Non-Catalytic Subunit	Protein Coding	45	GC01M020651	4.960511
TNFSF13B	TNF Superfamily Member 13b	Protein Coding	45	GC13P108251	4.959877
CHMP2B	Charged Multivesicular Body Protein 2B	Protein Coding	45	GC03P087227	4.936537
CRYAA	Crystallin Alpha A	Protein Coding	45	GC21P043169	4.933579
PPM1B	Protein Phosphatase, Mg2+/Mn2+ Dependent 1B	Protein Coding	45	GC02P044167	4.926708
SEC24D	SEC24 Homolog D, COPII Coat Complex Component	Protein Coding	45	GC04M118722	4.923567
TUFM	Tu Translation Elongation Factor, Mitochondrial	Protein Coding	45	GC16M029277	4.92113
TRIO	Trio Rho Guanine Nucleotide Exchange Factor	Protein Coding	45	GC05P014143	4.906826
NSDHL	NAD(P) Dependent Steroid Dehydrogenase-Like	Protein Coding	45	GC0XP152830	4.888424
CANX	Calnexin	Protein Coding	45	GC05P179678	4.878594
PNMT	Phenylethanolamine N-Methyltransferase	Protein Coding	45	GC17P039667	4.872728
CFL2	Cofilin 2	Protein Coding	45	GC14M034706	4.865085
SATB2	SATB Homeobox 2	Protein Coding	45	GC02M199269	4.817401
FADS1	Fatty Acid Desaturase 1	Protein Coding	45	GC11M061799	4.807359
CD27	CD27 Molecule	Protein Coding	45	GC12P011824	4.797257
RPL10	Ribosomal Protein L10	Protein Coding	45	GC0XP154389	4.792602
WNT2B	Wnt Family Member 2B	Protein Coding	45	GC01P112466	4.792132
TBL1XR1	TBL1X Receptor 1	Protein Coding	45	GC03M177019	4.781406
TNFSF15	TNF Superfamily Member 15	Protein Coding	45	GC09M114784	4.766306
TPH1	Tryptophan Hydroxylase 1	Protein Coding	45	GC11M018040	4.75988
PNPLA6	Patatin Like Phospholipase Domain Containing 6	Protein Coding	45	GC19P007534	4.747175
PTDSS1	Phosphatidylserine Synthase 1	Protein Coding	45	GC08P096261	4.737645
ATP2B1	ATPase Plasma Membrane Ca2+ Transporting 1	Protein Coding	45	GC12M089588	4.732946
STT3A	STT3 Oligosaccharyltransferase Complex Catalytic Subunit A	Protein Coding	45	GC11P125592	4.729828
KCNJ10	Potassium Inwardly Rectifying Channel Subfamily J Member 10	Protein Coding	45	GC01M159998	4.707903
ARL3	ADP Ribosylation Factor Like GTPase 3	Protein Coding	45	GC10M102673	4.700624
PAX7	Paired Box 7	Protein Coding	45	GC01P018631	4.686781
COL4A6	Collagen Type IV Alpha 6 Chain	Protein Coding	45	GC0XM108155	4.675633
DAO	D-Amino Acid Oxidase	Protein Coding	45	GC12P108859	4.669691
CR1	Complement C3b/C4b Receptor 1 (Knops Blood Group)	Protein Coding	45	GC01P207496	4.663899
POMGNT1	Protein O-Linked Mannose N-Acetylglucosaminyltransferase 1 (Beta 1,2-)	Protein Coding	45	GC01M046188	4.663396
RLBP1	Retinaldehyde Binding Protein 1	Protein Coding	45	GC15M089209	4.653583
FOXA2	Forkhead Box A2	Protein Coding	45	GC20M022581	4.611222
INHBA	Inhibin Subunit Beta A	Protein Coding	45	GC07M041668	4.575212
MEF2D	Myocyte Enhancer Factor 2D	Protein Coding	45	GC01M156463	4.560359
SERPINF1	Serpin Family F Member 1	Protein Coding	45	GC17P001761	4.517898
ISCU	Iron-Sulfur Cluster Assembly Enzyme	Protein Coding	45	GC12P108561	4.506897
KLF1	Kruppel Like Factor 1	Protein Coding	45	GC19M012884	4.493937
RPS14	Ribosomal Protein S14	Protein Coding	45	GC05M150443	4.491768
TAC3	Tachykinin Precursor 3	Protein Coding	45	GC12M057009	4.490714
ATP2B4	ATPase Plasma Membrane Ca2+ Transporting 4	Protein Coding	45	GC01P203626	4.47688
NOTCH4	Notch Receptor 4	Protein Coding	45	GC06M046911	4.471302
RICTOR	RPTOR Independent Companion Of MTOR Complex 2	Protein Coding	45	GC05M038937	4.46084
FZD1	Frizzled Class Receptor 1	Protein Coding	45	GC07P091264	4.446043
NFIA	Nuclear Factor I A	Protein Coding	45	GC01P060865	4.414341
ARRB2	Arrestin Beta 2	Protein Coding	45	GC17P004711	4.402006
COPB2	COPI Coat Complex Subunit Beta 2	Protein Coding	45	GC03M139355	4.397677
UGT2B7	UDP Glucuronosyltransferase Family 2 Member B7	Protein Coding	45	GC04P069051	4.364991
TNNI2	Troponin I2, Fast Skeletal Type	Protein Coding	45	GC11P001839	4.347838
MKI67	Marker Of Proliferation Ki-67	Protein Coding	45	GC10M128096	4.347814
MPDZ	Multiple PDZ Domain Crumbs Cell Polarity Complex Component	Protein Coding	45	GC09M013095	4.345497
S1PR1	Sphingosine-1-Phosphate Receptor 1	Protein Coding	45	GC01P101236	4.321205
AQP7	Aquaporin 7	Protein Coding	45	GC09M033384	4.313178

MGAT2	Alpha-1,6-Mannosyl-Glycoprotein 2-Beta-N-Acetylglucosaminyltransferase	Protein Coding	45	GC14P049620	4.311026
NLRP1	NLR Family Pyrin Domain Containing 1	Protein Coding	45	GC17M005499	4.279888
CDC73	Cell Division Cycle 73	Protein Coding	45	GC01P193121	4.278891
HSF1	Heat Shock Transcription Factor 1	Protein Coding	45	GC08P144291	4.267723
GRIN2C	Glutamate Ionotropic Receptor NMDA Type Subunit 2C	Protein Coding	45	GC17M074842	4.255948
B4GALT1	Beta-1,4-Galactosyltransferase 1	Protein Coding	45	GC09M033100	4.252995
SEMA4D	Semaphorin 4D	Protein Coding	45	GC09M089360	4.252288
EIF2S1	Eukaryotic Translation Initiation Factor 2 Subunit Alpha	Protein Coding	45	GC14P067359	4.239626
SLC12A4	Solute Carrier Family 12 Member 4	Protein Coding	45	GC16M067954	4.215108
STEAP3	STEAP3 Metalloreductase	Protein Coding	45	GC02P119222	4.210695
GFER	Growth Factor, Augmenter Of Liver Regeneration	Protein Coding	45	GC16P001984	4.205681
STXBP2	Syntaxin Binding Protein 2	Protein Coding	45	GC19P007637	4.193765
CPB2	Carboxypeptidase B2	Protein Coding	45	GC13M046053	4.177775
BTD	Biotinidase	Protein Coding	45	GC03P015967	4.168637
ATXN3	Ataxin 3	Protein Coding	45	GC14M095796	4.166193
CDK10	Cyclin Dependent Kinase 10	Protein Coding	45	GC16P089680	4.153739
NTN1	Netrin 1	Protein Coding	45	GC17P009021	4.125257
HTR4	5-Hydroxytryptamine Receptor 4	Protein Coding	45	GC05M148451	4.112545
CRYM	Crystallin Mu	Protein Coding	45	GC16M021238	4.096501
CFD	Complement Factor D	Protein Coding	45	GC19P000859	4.087578
COL4A2	Collagen Type IV Alpha 2 Chain	Protein Coding	45	GC13P110305	4.086798
CD38	CD38 Molecule	Protein Coding	45	GC04P015779	4.047471
HSPB8	Heat Shock Protein Family B (Small) Member 8	Protein Coding	45	GC12P119173	4.039878
WIPF1	WAS/WASL Interacting Protein Family Member 1	Protein Coding	45	GC02M174559	4.00301
SNAI2	Snail Family Transcriptional Repressor 2	Protein Coding	45	GC08M048917	4.002428
DRD1	Dopamine Receptor D1	Protein Coding	45	GC05M175440	3.987464
IDO1	Indoleamine 2,3-Dioxygenase 1	Protein Coding	45	GC08P039891	3.979544
CCR7	C-C Motif Chemokine Receptor 7	Protein Coding	45	GC17M040556	3.963297
CYB5A	Cytochrome B5 Type A	Protein Coding	45	GC18M074250	3.960115
NR1I3	Nuclear Receptor Subfamily 1 Group I Member 3	Protein Coding	45	GC01M161229	3.948344
ID2	Inhibitor Of DNA Binding 2	Protein Coding	45	GC02P008678	3.942055
HAAO	3-Hydroxyanthranilate 3,4-Dioxygenase	Protein Coding	45	GC02M042767	3.929552
GYPA	Glycophorin A (MNS Blood Group)	Protein Coding	45	GC04M144109	3.896911
TICAM1	Toll Like Receptor Adaptor Molecule 1	Protein Coding	45	GC19M004815	3.893686
CLEC7A	C-Type Lectin Domain Containing 7A	Protein Coding	45	GC12M015684	3.875379
SLC22A2	Solute Carrier Family 22 Member 2	Protein Coding	45	GC06M160173	3.865302
MYO5B	Myosin VB	Protein Coding	45	GC18M049822	3.847836
BAK1	BCL2 Antagonist/Killer 1	Protein Coding	45	GC06M033572	3.834992
WARS2	Tryptophanyl TRNA Synthetase 2, Mitochondrial	Protein Coding	45	GC01M119031	3.786078
FDXR	Ferredoxin Reductase	Protein Coding	45	GC17M074862	3.77743
AGA	Aspartylglucosaminidase	Protein Coding	45	GC04M177430	3.749671
PRPH	Peripherin	Protein Coding	45	GC12P049293	3.743964
KRT1	Keratin 1	Protein Coding	45	GC12M052674	3.733914
CHD1	Chromodomain Helicase DNA Binding Protein 1	Protein Coding	45	GC05M098853	3.730665
REST	RE1 Silencing Transcription Factor	Protein Coding	45	GC04P056907	3.727983
IL17RA	Interleukin 17 Receptor A	Protein Coding	45	GC22P017116	3.720686
CHRNA3	Cholinergic Receptor Nicotinic Alpha 3 Subunit	Protein Coding	45	GC15M078594	3.708708
ALOX15	Arachidonate 15-Lipoxygenase	Protein Coding	45	GC17M004630	3.70238
PRDM1	PR/SET Domain 1	Protein Coding	45	GC06P105993	3.699577
KLK1	Kallikrein 1	Protein Coding	45	GC19M050819	3.685194
OCLN	Occludin	Protein Coding	45	GC05P069492	3.677879
COX6A1	Cytochrome C Oxidase Subunit 6A1	Protein Coding	45	GC12P120439	3.671128
SLC22A6	Solute Carrier Family 22 Member 6	Protein Coding	45	GC11M069278	3.665086

CENPE	Centromere Protein E	Protein Coding	45	GC04M103105	3.618272
MCOLN1	Mucolipin TRP Cation Channel 1	Protein Coding	45	GC19P007523	3.603903
NLRC4	NLR Family CARD Domain Containing 4	Protein Coding	45	GC02M032224	3.601779
MCCC2	Methylcrotonyl-CoA Carboxylase Subunit 2	Protein Coding	45	GC05P071587	3.593278
HABP2	Hyaluronan Binding Protein 2	Protein Coding	45	GC10P113550	3.585933
SLC22A3	Solute Carrier Family 22 Member 3	Protein Coding	45	GC06P160348	3.557597
NOD1	Nucleotide Binding Oligomerization Domain Containing 1	Protein Coding	45	GC07M030424	3.557143
POFUT1	Protein O-Fucosyltransferase 1	Protein Coding	45	GC20P032207	3.54862
SDC4	Syndecan 4	Protein Coding	45	GC20M045325	3.544958
AKR1A1	Aldo-Keto Reductase Family 1 Member A1	Protein Coding	45	GC01P045550	3.519526
PYGB	Glycogen Phosphorylase B	Protein Coding	45	GC20P025248	3.519332
ADAM12	ADAM Metallopeptidase Domain 12	Protein Coding	45	GC10M126012	3.517106
ADARB1	Adenosine Deaminase RNA Specific B1	Protein Coding	45	GC21P045073	3.500484
RPS3	Ribosomal Protein S3	Protein Coding	45	GC11P076260	3.482907
IFNAR1	Interferon Alpha And Beta Receptor Subunit 1	Protein Coding	45	GC21P033324	3.4791
PTRH2	Peptidyl-TRNA Hydrolase 2	Protein Coding	45	GC17M059674	3.47545
C3AR1	Complement C3a Receptor 1	Protein Coding	45	GC12M008058	3.472087
TOR1A	Torsin Family 1 Member A	Protein Coding	45	GC09M129812	3.400696
PRKAR2B	Protein Kinase CAMP-Dependent Type II Regulatory Subunit Beta	Protein Coding	45	GC07P107044	3.400279
REV3L	REV3 Like, DNA Directed Polymerase Zeta Catalytic Subunit	Protein Coding	45	GC06M111299	3.386325
ENPEP	Glutamyl Aminopeptidase	Protein Coding	45	GC04P110365	3.385889
PYCR2	Pyrroline-5-Carboxylate Reductase 2	Protein Coding	45	GC01M225919	3.382275
RXFP1	Relaxin Family Peptide Receptor 1	Protein Coding	45	GC04P158315	3.377352
SNAI1	Snail Family Transcriptional Repressor 1	Protein Coding	45	GC20P049982	3.364567
SLC7A5	Solute Carrier Family 7 Member 5	Protein Coding	45	GC16M087830	3.351887
ICAM2	Intercellular Adhesion Molecule 2	Protein Coding	45	GC17M064002	3.344637
S1PR3	Sphingosine-1-Phosphate Receptor 3	Protein Coding	45	GC09P088991	3.342795
RUVBL1	RuvB Like AAA ATPase 1	Protein Coding	45	GC03M128064	3.341972
SLC44A1	Solute Carrier Family 44 Member 1	Protein Coding	45	GC09P105244	3.341529
PTPN3	Protein Tyrosine Phosphatase Non-Receptor Type 3	Protein Coding	45	GC09M109375	3.339618
KHK	Ketohexokinase	Protein Coding	45	GC02P027086	3.336841
ACO1	Aconitase 1	Protein Coding	45	GC09P032374	3.336132
PROM1	Prominin 1	Protein Coding	45	GC04M015965	3.309571
BMX	BMX Non-Receptor Tyrosine Kinase	Protein Coding	45	GC0XP015392	3.300786
SAG	S-Antigen Visual Arrestin	Protein Coding	45	GC02P233328	3.285895
FGF14	Fibroblast Growth Factor 14	Protein Coding	45	GC13M101710	3.283391
PABPN1	Poly(A) Binding Protein Nuclear 1	Protein Coding	45	GC14P026483	3.243377
ITGAX	Integrin Subunit Alpha X	Protein Coding	45	GC16P032567	3.24031
PLIN1	Perilipin 1	Protein Coding	45	GC15M089664	3.239485
SOAT1	Sterol O-Acyltransferase 1	Protein Coding	45	GC01P179262	3.229852
FBXW11	F-Box And WD Repeat Domain Containing 11	Protein Coding	45	GC05M171861	3.228627
RPSA	Ribosomal Protein SA	Protein Coding	45	GC03P039406	3.224594
TAT	Tyrosine Aminotransferase	Protein Coding	45	GC16M071565	3.221958
HTR1B	5-Hydroxytryptamine Receptor 1B	Protein Coding	45	GC06M077478	3.215703
TRAF2	TNF Receptor Associated Factor 2	Protein Coding	45	GC09P136881	3.212055
NGLY1	N-Glycanase 1	Protein Coding	45	GC03M025718	3.205441
SMS	Spermine Synthase	Protein Coding	45	GC0XP021958	3.200447
ATF3	Activating Transcription Factor 3	Protein Coding	45	GC01P212565	3.198698
SLC25A5	Solute Carrier Family 25 Member 5	Protein Coding	45	GC0XP119468	3.196057
UBA2	Ubiquitin Like Modifier Activating Enzyme 2	Protein Coding	45	GC19P034428	3.159551
DRD3	Dopamine Receptor D3	Protein Coding	45	GC03M114128	3.156979
TUBA8	Tubulin Alpha 8	Protein Coding	45	GC22P018110	3.155806
HOXA1	Homeobox A1	Protein Coding	45	GC07M027092	3.14801

RAN	RAN, Member RAS Oncogene Family	Protein Coding	45	GC12P130871	3.133386
ABCA4	ATP Binding Cassette Subfamily A Member 4	Protein Coding	45	GC01M093992	3.130388
SNCAIP	Synuclein Alpha Interacting Protein	Protein Coding	45	GC05P122311	3.125695
SGPL1	Sphingosine-1-Phosphate Lyase 1	Protein Coding	45	GC10P070815	3.117648
CYP26A1	Cytochrome P450 Family 26 Subfamily A Member 1	Protein Coding	45	GC10P093073	3.103843
PLAA	Phospholipase A2 Activating Protein	Protein Coding	45	GC09M026903	3.094187
ALS2	Alsin Rho Guanine Nucleotide Exchange Factor ALS2	Protein Coding	45	GC02M201701	3.083595
PTPRD	Protein Tyrosine Phosphatase Receptor Type D	Protein Coding	45	GC09M008307	3.080509
STS	Steroid Sulfatase	Protein Coding	45	GC0XP007146	3.078655
NCOR2	Nuclear Receptor Corepressor 2	Protein Coding	45	GC12M124324	3.046015
UBE2A	Ubiquitin Conjugating Enzyme E2 A	Protein Coding	45	GC0XP119574	3.039357
HMGCS2	3-Hydroxy-3-Methylglutaryl-CoA Synthase 2	Protein Coding	45	GC01M119747	3.027639
RAPGEF3	Rap Guanine Nucleotide Exchange Factor 3	Protein Coding	45	GC12M047736	3.026345
AP2M1	Adaptor Related Protein Complex 2 Subunit Mu 1	Protein Coding	45	GC03P184174	3.020843
MYO5A	Myosin VA	Protein Coding	45	GC15M067802	3.020731
CPA1	Carboxypeptidase A1	Protein Coding	45	GC07P130380	3.003742
TAP2	Transporter 2, ATP Binding Cassette Subfamily B Member	Protein Coding	45	GC06M032821	2.997978
OGT	O-Linked N-Acetylglucosamine (GlcNAc) Transferase	Protein Coding	45	GC0XP071534	2.991503
DFFA	DNA Fragmentation Factor Subunit Alpha	Protein Coding	45	GC01M010456	2.982933
IGFALS	Insulin Like Growth Factor Binding Protein Acid Labile Subunit	Protein Coding	45	GC16M001790	2.981542
BCL2L11	BCL2 Like 11	Protein Coding	45	GC02P111119	2.959277
SLIT2	Slit Guidance Ligand 2	Protein Coding	45	GC04P020287	2.943774
AASS	Aminoadipate-Semialdehyde Synthase	Protein Coding	45	GC07M122073	2.942234
TP53BP1	Tumor Protein P53 Binding Protein 1	Protein Coding	45	GC15M043403	2.940718
SLC12A7	Solute Carrier Family 12 Member 7	Protein Coding	45	GC05M001050	2.940717
CYP51A1	Cytochrome P450 Family 51 Subfamily A Member 1	Protein Coding	45	GC07M092112	2.935777
IGLL1	Immunoglobulin Lambda Like Polypeptide 1	Protein Coding	45	GC22M023573	2.923031
SOCS2	Suppressor Of Cytokine Signaling 2	Protein Coding	45	GC12P093569	2.900635
GJA8	Gap Junction Protein Alpha 8	Protein Coding	45	GC01P147902	2.879449
DCXR	Dicarbonyl And L-Xylulose Reductase	Protein Coding	45	GC17M082036	2.876945
ULK1	Unc-51 Like Autophagy Activating Kinase 1	Protein Coding	45	GC12P131894	2.873975
SLC7A11	Solute Carrier Family 7 Member 11	Protein Coding	45	GC04M138164	2.869571
CNTN1	Contactin 1	Protein Coding	45	GC12P040692	2.867103
GABARAP	GABA Type A Receptor-Associated Protein	Protein Coding	45	GC17M007240	2.86538
SORL1	Sortilin Related Receptor 1	Protein Coding	45	GC11P121452	2.859673
PGRMC1	Progesterone Receptor Membrane Component 1	Protein Coding	45	GC0XP119236	2.835039
GLRX	Glutaredoxin	Protein Coding	45	GC05M095752	2.825935
CCR4	C-C Motif Chemokine Receptor 4	Protein Coding	45	GC03P032951	2.824059
PFKFB3	6-Phosphofructo-2-Kinase/Fructose-2,6-Biphosphatase 3	Protein Coding	45	GC10P006144	2.81227
LIG3	DNA Ligase 3	Protein Coding	45	GC17P034980	2.812187
KRT16	Keratin 16	Protein Coding	45	GC17M041609	2.800075
FKBP4	FKBP Prolyl Isomerase 4	Protein Coding	45	GC12P002795	2.792339
NLGN1	Neuroligin 1	Protein Coding	45	GC03P173396	2.790362
SEMA4A	Semaphorin 4A	Protein Coding	45	GC01P156147	2.760791
MFGE8	Milk Fat Globule EGF And Factor V/VIII Domain Containing	Protein Coding	45	GC15M088898	2.760296
TFG	Trafficking From ER To Golgi Regulator	Protein Coding	45	GC03P100709	2.757755
TAL1	TAL BHLH Transcription Factor 1, Erythroid Differentiation Factor	Protein Coding	45	GC01M047216	2.753338
C1QB	Complement C1q B Chain	Protein Coding	45	GC01P022652	2.750389
CTBP2	C-Terminal Binding Protein 2	Protein Coding	45	GC10M124984	2.74201
SLC1A4	Solute Carrier Family 1 Member 4	Protein Coding	45	GC02P064988	2.732227
RHAG	Rh Associated Glycoprotein	Protein Coding	45	GC06M049605	2.7239
CPE	Carboxypeptidase E	Protein Coding	45	GC04P165361	2.713419
CACNA1F	Calcium Voltage-Gated Channel Subunit Alpha1 F	Protein Coding	45	GC0XM049205	2.701696

PDGFD	Platelet Derived Growth Factor D	Protein Coding	45	GC11M103907	2.700978
GRIK1	Glutamate Ionotropic Receptor Kainate Type Subunit 1	Protein Coding	45	GC21M029536	2.691761
GDI1	GDP Dissociation Inhibitor 1	Protein Coding	45	GC0XP154436	2.689173
BCKDHA	Branched Chain Keto Acid Dehydrogenase E1 Subunit Alpha	Protein Coding	45	GC19P041400	2.682316
VAPA	VAMP Associated Protein A	Protein Coding	45	GC18P009904	2.680515
STK24	Serine/Threonine Kinase 24	Protein Coding	45	GC13M098445	2.668013
IL1R2	Interleukin 1 Receptor Type 2	Protein Coding	45	GC02P101991	2.6603
ALAS1	5'-Aminolevulinate Synthase 1	Protein Coding	45	GC03P052198	2.638528
PBRM1	Polybromo 1	Protein Coding	45	GC03M052545	2.636278
CBX5	Chromobox 5	Protein Coding	45	GC12M054230	2.611372
LTB4R	Leukotriene B4 Receptor	Protein Coding	45	GC14P024311	2.610131
HCRTR2	Hypocretin Receptor 2	Protein Coding	45	GC06P055106	2.604452
FBXW7	F-Box And WD Repeat Domain Containing 7	Protein Coding	45	GC04M152321	2.594286
AOX1	Aldehyde Oxidase 1	Protein Coding	45	GC02P200585	2.592881
TCF7	Transcription Factor 7	Protein Coding	45	GC05P134114	2.583729
PAPSS2	3'-Phosphoadenosine 5'-Phosphosulfate Synthase 2	Protein Coding	45	GC10P087659	2.581858
CLDN14	Claudin 14	Protein Coding	45	GC21M036460	2.572677
GABRA3	Gamma-Aminobutyric Acid Type A Receptor Subunit Alpha3	Protein Coding	45	GC0XM152166	2.57207
TIAM1	TIAM Rac1 Associated GEF 1	Protein Coding	45	GC21M031118	2.570327
EOMES	Eomesodermin	Protein Coding	45	GC03M027715	2.565298
KCNQ4	Potassium Voltage-Gated Channel Subfamily Q Member 4	Protein Coding	45	GC01P040784	2.555249
EIF4A1	Eukaryotic Translation Initiation Factor 4A1	Protein Coding	45	GC17P007572	2.544221
ASPA	Aspartoacylase	Protein Coding	45	GC17P003472	2.538261
DLX5	Distal-Less Homeobox 5	Protein Coding	45	GC07M097020	2.528645
BCAR1	BCAR1 Scaffold Protein, Cas Family Member	Protein Coding	45	GC16M075228	2.508344
BRD2	Bromodomain Containing 2	Protein Coding	45	GC06P055236	2.503455
CACNG2	Calcium Voltage-Gated Channel Auxiliary Subunit Gamma 2	Protein Coding	45	GC22M036561	2.49441
PI4KB	Phosphatidylinositol 4-Kinase Beta	Protein Coding	45	GC01M151291	2.492706
C1QC	Complement C1q C Chain	Protein Coding	45	GC01P022643	2.487933
MCM7	Minichromosome Maintenance Complex Component 7	Protein Coding	45	GC07M100092	2.483016
TUBB2B	Tubulin Beta 2B Class IIb	Protein Coding	45	GC06M003224	2.479147
AMPH	Amphiphysin	Protein Coding	45	GC07M038782	2.467763
SLC29A2	Solute Carrier Family 29 Member 2	Protein Coding	45	GC11M069464	2.456785
LINGO1	Leucine Rich Repeat And Ig Domain Containing 1	Protein Coding	45	GC15M077613	2.441528
GALR1	Galanin Receptor 1	Protein Coding	45	GC18P077250	2.433717
SMURF1	SMAD Specific E3 Ubiquitin Protein Ligase 1	Protein Coding	45	GC07M099027	2.432356
GJB6	Gap Junction Protein Beta 6	Protein Coding	45	GC13M020221	2.411679
TMPRSS2	Transmembrane Serine Protease 2	Protein Coding	45	GC21M041464	2.397908
CCKBR	Cholecystokinin B Receptor	Protein Coding	45	GC11P006259	2.378284
CDH15	Cadherin 15	Protein Coding	45	GC16P089171	2.365963
MAPKAPK5	MAPK Activated Protein Kinase 5	Protein Coding	45	GC12P111842	2.338324
PDE7A	Phosphodiesterase 7A	Protein Coding	45	GC08M065720	2.328043
VIPR2	Vasoactive Intestinal Peptide Receptor 2	Protein Coding	45	GC07M159028	2.323468
RASSF1	Ras Association Domain Family Member 1	Protein Coding	45	GC03M050329	2.30684
PFKL	Phosphofructokinase, Liver Type	Protein Coding	45	GC21P044300	2.304879
BLVRA	Biliverdin Reductase A	Protein Coding	45	GC07P043758	2.302123
PEBP1	Phosphatidylethanolamine Binding Protein 1	Protein Coding	45	GC12P118135	2.263429
MAP3K14	Mitogen-Activated Protein Kinase Kinase Kinase 14	Protein Coding	45	GC17M045263	2.26
RNASEL	Ribonuclease L	Protein Coding	45	GC01M182542	2.256968
GJB3	Gap Junction Protein Beta 3	Protein Coding	45	GC01P034781	2.249944
OPRD1	Opioid Receptor Delta 1	Protein Coding	45	GC01P028812	2.240966
ROS1	ROS Proto-Oncogene 1, Receptor Tyrosine Kinase	Protein Coding	45	GC06M117287	2.232918
KIF5B	Kinesin Family Member 5B	Protein Coding	45	GC10M032035	2.229831

CHRNA5	Cholinergic Receptor Nicotinic Alpha 5 Subunit	Protein Coding	45	GC15P078565	2.227812
KRT6A	Keratin 6A	Protein Coding	45	GC12M052488	2.216555
ARHGAP26	Rho GTPase Activating Protein 26	Protein Coding	45	GC05P142770	2.206789
SDC2	Syndecan 2	Protein Coding	45	GC08P096495	2.193388
BCL10	BCL10 Immune Signaling Adaptor	Protein Coding	45	GC01M085265	2.189228
FGF5	Fibroblast Growth Factor 5	Protein Coding	45	GC04P080266	2.186583
MUTYH	MutY DNA Glycosylase	Protein Coding	45	GC01M045329	2.186182
CYP2C18	Cytochrome P450 Family 2 Subfamily C Member 18	Protein Coding	45	GC10P094684	2.175211
SKP2	S-Phase Kinase Associated Protein 2	Protein Coding	45	GC05P036151	2.172415
RDH12	Retinol Dehydrogenase 12	Protein Coding	45	GC14P067701	2.148901
TNFRSF10A	TNF Receptor Superfamily Member 10a	Protein Coding	45	GC08M023190	2.140307
LITAF	Lipopolysaccharide Induced TNF Factor	Protein Coding	45	GC16M011547	2.13726
PIAS1	Protein Inhibitor Of Activated STAT 1	Protein Coding	45	GC15P068054	2.129728
OSMR	Oncostatin M Receptor	Protein Coding	45	GC05P038845	2.128844
EGLN2	Egl-9 Family Hypoxia Inducible Factor 2	Protein Coding	45	GC19P041111	2.113937
NDRG1	N-Myc Downstream Regulated 1	Protein Coding	45	GC08M133237	2.106851
TNIK	TRAF2 And NCK Interacting Kinase	Protein Coding	45	GC03M171061	2.101355
HIPK2	Homeodomain Interacting Protein Kinase 2	Protein Coding	45	GC07M139561	2.08742
RDH11	Retinol Dehydrogenase 11	Protein Coding	45	GC14M067676	2.079354
NCOA1	Nuclear Receptor Coactivator 1	Protein Coding	45	GC02P024492	2.076177
FA2H	Fatty Acid 2-Hydroxylase	Protein Coding	45	GC16M074712	2.076052
PMVK	Phosphomevalonate Kinase	Protein Coding	45	GC01M154924	2.074651
CDC20	Cell Division Cycle 20	Protein Coding	45	GC01P043358	2.057374
HPSE	Heparanase	Protein Coding	45	GC04M083292	2.048493
NCOA2	Nuclear Receptor Coactivator 2	Protein Coding	45	GC08M070109	2.048032
FZD9	Frizzled Class Receptor 9	Protein Coding	45	GC07P073433	2.045262
RUVBL2	RuvB Like AAA ATPase 2	Protein Coding	45	GC19P048993	2.044344
MMP16	Matrix Metallopeptidase 16	Protein Coding	45	GC08M088032	2.042291
ALDH3A2	Aldehyde Dehydrogenase 3 Family Member A2	Protein Coding	45	GC17P019648	2.016049
ALDH4A1	Aldehyde Dehydrogenase 4 Family Member A1	Protein Coding	45	GC01M018871	2.013182
S1PR2	Sphingosine-1-Phosphate Receptor 2	Protein Coding	45	GC19M010223	2.001276
SRD5A1	Steroid 5 Alpha-Reductase 1	Protein Coding	45	GC05P006633	1.9996
MAPK6	Mitogen-Activated Protein Kinase 6	Protein Coding	45	GC15P051952	1.998229
KIF5A	Kinesin Family Member 5A	Protein Coding	45	GC12P057549	1.99527
GAB1	GRB2 Associated Binding Protein 1	Protein Coding	45	GC04P143336	1.99271
TNFRSF21	TNF Receptor Superfamily Member 21	Protein Coding	45	GC06M047231	1.987887
HGS	Hepatocyte Growth Factor-Regulated Tyrosine Kinase Substrate	Protein Coding	45	GC17P081683	1.982403
USP1	Ubiquitin Specific Peptidase 1	Protein Coding	45	GC01P062436	1.97106
PTH2R	Parathyroid Hormone 2 Receptor	Protein Coding	45	GC02P208359	1.943454
HINT1	Histidine Triad Nucleotide Binding Protein 1	Protein Coding	45	GC05M131159	1.925683
ADCY8	Adenylate Cyclase 8	Protein Coding	45	GC08M130780	1.919404
FOXM1	Forkhead Box M1	Protein Coding	45	GC12M002857	1.918506
SLC6A19	Solute Carrier Family 6 Member 19	Protein Coding	45	GC05P001201	1.909059
TNFRSF9	TNF Receptor Superfamily Member 9	Protein Coding	45	GC01M007915	1.90256
AMD1	Adenosylmethionine Decarboxylase 1	Protein Coding	45	GC06P110814	1.892501
ARF6	ADP Ribosylation Factor 6	Protein Coding	45	GC14P049895	1.889551
GADD45A	Growth Arrest And DNA Damage Inducible Alpha	Protein Coding	45	GC01P067685	1.884803
RPA2	Replication Protein A2	Protein Coding	45	GC01M027902	1.879477
RAP1B	RAP1B, Member Of RAS Oncogene Family	Protein Coding	45	GC12P068610	1.877414
FCER2	Fc Fragment Of IgE Receptor II	Protein Coding	45	GC19M007689	1.853097
WNT5B	Wnt Family Member 5B	Protein Coding	45	GC12P001529	1.846402
GABRB1	Gamma-Aminobutyric Acid Type A Receptor Subunit Beta1	Protein Coding	45	GC04P046949	1.84614
GRID2	Glutamate Ionotropic Receptor Delta Type Subunit 2	Protein Coding	45	GC04P092304	1.841924

NCK1	NCK Adaptor Protein 1	Protein Coding	45	GC03P136862	1.83768
ALDH1B1	Aldehyde Dehydrogenase 1 Family Member B1	Protein Coding	45	GC09P038392	1.832921
MAP3K6	Mitogen-Activated Protein Kinase Kinase Kinase 6	Protein Coding	45	GC01M027354	1.8118
CCNB2	Cyclin B2	Protein Coding	45	GC15P059105	1.811505
GMDS	GDP-Mannose 4,6-Dehydratase	Protein Coding	45	GC06M001624	1.797642
DGKZ	Diacylglycerol Kinase Zeta	Protein Coding	45	GC11P046332	1.78765
METAP2	Methionyl Aminopeptidase 2	Protein Coding	45	GC12P095473	1.772361
GGPS1	Geranylgeranyl Diphosphate Synthase 1	Protein Coding	45	GC01P235327	1.768029
NAT1	N-Acetyltransferase 1	Protein Coding	45	GC08P018179	1.761632
PSMB5	Proteasome 20S Subunit Beta 5	Protein Coding	45	GC14M023016	1.755573
PTPRN	Protein Tyrosine Phosphatase Receptor Type N	Protein Coding	45	GC02M219289	1.75022
SULT1E1	Sulfotransferase Family 1E Member 1	Protein Coding	45	GC04M069841	1.744881
ANXA4	Annexin A4	Protein Coding	45	GC02P069644	1.743322
GNAZ	G Protein Subunit Alpha Z	Protein Coding	45	GC22P023070	1.738071
CHRM5	Cholinergic Receptor Muscarinic 5	Protein Coding	45	GC15P033968	1.722566
GABRA4	Gamma-Aminobutyric Acid Type A Receptor Subunit Alpha4	Protein Coding	45	GC04M046836	1.707612
SLC20A1	Solute Carrier Family 20 Member 1	Protein Coding	45	GC02P116687	1.701532
PPP2R5C	Protein Phosphatase 2 Regulatory Subunit B'Gamma	Protein Coding	45	GC14P106421	1.689881
PTGFR	Prostaglandin F Receptor	Protein Coding	45	GC01P078303	1.666686
TRPC4	Transient Receptor Potential Cation Channel Subfamily C Member 4	Protein Coding	45	GC13M037636	1.664766
FUT2	Fucosyltransferase 2	Protein Coding	45	GC19P048695	1.657372
CBLB	Cbl Proto-Oncogene B	Protein Coding	45	GC03M105655	1.641025
NR2C2	Nuclear Receptor Subfamily 2 Group C Member 2	Protein Coding	45	GC03P014947	1.638093
SLC16A3	Solute Carrier Family 16 Member 3	Protein Coding	45	GC17P082217	1.636912
CAMK1	Calcium/Calmodulin Dependent Protein Kinase I	Protein Coding	45	GC03M009774	1.635955
WASF1	WASP Family Member 1	Protein Coding	45	GC06M110099	1.635292
TNFSF13	TNF Superfamily Member 13	Protein Coding	45	GC17P007558	1.614129
GCNT2	Glucosaminyl (N-Acetyl) Transferase 2 (I Blood Group)	Protein Coding	45	GC06P010492	1.600185
TLE1	TLE Family Member 1, Transcriptional Corepressor	Protein Coding	45	GC09M081583	1.598356
POU2F1	POU Class 2 Homeobox 1	Protein Coding	45	GC01P167190	1.586748
RAD23B	RAD23 Homolog B, Nucleotide Excision Repair Protein	Protein Coding	45	GC09P107283	1.575163
WWP2	WW Domain Containing E3 Ubiquitin Protein Ligase 2	Protein Coding	45	GC16P069796	1.572806
IL10RB	Interleukin 10 Receptor Subunit Beta	Protein Coding	45	GC21P033266	1.571838
EIF4A2	Eukaryotic Translation Initiation Factor 4A2	Protein Coding	45	GC03P186783	1.570074
SULT2A1	Sulfotransferase Family 2A Member 1	Protein Coding	45	GC19M047870	1.569096
PPP5C	Protein Phosphatase 5 Catalytic Subunit	Protein Coding	45	GC19P046346	1.547018
WIF1	WNT Inhibitory Factor 1	Protein Coding	45	GC12M065050	1.538922
ADH7	Alcohol Dehydrogenase 7 (Class IV), Mu Or Sigma Polypeptide	Protein Coding	45	GC04M099412	1.531132
IRF9	Interferon Regulatory Factor 9	Protein Coding	45	GC14P024161	1.530791
PSMA7	Proteasome 20S Subunit Alpha 7	Protein Coding	45	GC20M062136	1.517202
PDE4C	Phosphodiesterase 4C	Protein Coding	45	GC19M018218	1.510287
RPE65	Retinoid Isomerohydrolase RPE65	Protein Coding	45	GC01M068428	1.510271
CUL5	Cullin 5	Protein Coding	45	GC11P108008	1.508765
SLC6A6	Solute Carrier Family 6 Member 6	Protein Coding	45	GC03P014402	1.50251
PPM1A	Protein Phosphatase, Mg2+/Mn2+ Dependent 1A	Protein Coding	45	GC14P060245	1.49139
IL11RA	Interleukin 11 Receptor Subunit Alpha	Protein Coding	45	GC09P034650	1.490021
KCNJ13	Potassium Inwardly Rectifying Channel Subfamily J Member 13	Protein Coding	45	GC02M232765	1.46585
EPHB3	EPH Receptor B3	Protein Coding	45	GC03P184561	1.459911
LRAT	Lecithin Retinol Acyltransferase	Protein Coding	45	GC04P154626	1.45439
PSMB1	Proteasome 20S Subunit Beta 1	Protein Coding	45	GC06M170535	1.451764
PSMA4	Proteasome 20S Subunit Alpha 4	Protein Coding	45	GC15P078540	1.448673
KMO	Kynurenine 3-Monooxygenase	Protein Coding	45	GC01P241532	1.447082
SNAP23	Synaptosome Associated Protein 23	Protein Coding	45	GC15P042491	1.423382

TDG	Thymine DNA Glycosylase	Protein Coding	45	GC12P103965	1.416887
DCK	Deoxycytidine Kinase	Protein Coding	45	GC04P070992	1.412774
DUSP3	Dual Specificity Phosphatase 3	Protein Coding	45	GC17M043766	1.410314
PVR	PVR Cell Adhesion Molecule	Protein Coding	45	GC19P044644	1.402421
P2RY11	Purinergic Receptor P2Y11	Protein Coding	45	GC19P010184	1.382915
GPX2	Glutathione Peroxidase 2	Protein Coding	45	GC14M064939	1.36842
ADH4	Alcohol Dehydrogenase 4 (Class II), Pi Polypeptide	Protein Coding	45	GC04M099123	1.363953
GNA13	G Protein Subunit Alpha 13	Protein Coding	45	GC17M065009	1.360454
DOT1L	DOT1 Like Histone Lysine Methyltransferase	Protein Coding	45	GC19P002164	1.359399
CUL1	Cullin 1	Protein Coding	45	GC07P148697	1.348528
GALM	Galactose Mutarotase	Protein Coding	45	GC02P038665	1.346019
NLK	Nemo Like Kinase	Protein Coding	45	GC17P029295	1.342243
WDR5	WD Repeat Domain 5	Protein Coding	45	GC09P134135	1.337756
FOSL1	FOS Like 1, AP-1 Transcription Factor Subunit	Protein Coding	45	GC11M069434	1.327818
GRK6	G Protein-Coupled Receptor Kinase 6	Protein Coding	45	GC05P177403	1.309056
PTPRN2	Protein Tyrosine Phosphatase Receptor Type N2	Protein Coding	45	GC07M157539	1.30724
USP13	Ubiquitin Specific Peptidase 13	Protein Coding	45	GC03P179652	1.304174
SLC15A1	Solute Carrier Family 15 Member 1	Protein Coding	45	GC13M098683	1.303633
PSMA3	Proteasome 20S Subunit Alpha 3	Protein Coding	45	GC14P058244	1.302593
GABRA6	Gamma-Aminobutyric Acid Type A Receptor Subunit Alpha6	Protein Coding	45	GC05P161547	1.302415
NANS	N-Acetylneuraminate Synthase	Protein Coding	45	GC09P098056	1.295549
BTC	Betacellulin	Protein Coding	45	GC04M074744	1.292966
CA9	Carbonic Anhydrase 9	Protein Coding	45	GC09P035673	1.232465
DOCK2	Dedicator Of Cytokinesis 2	Protein Coding	45	GC05P169637	1.217712
BCL2L2	BCL2 Like 2	Protein Coding	45	GC14P026355	1.200272
CHRNB2	Cholinergic Receptor Nicotinic Beta 2 Subunit	Protein Coding	45	GC01P154568	1.199171
NSF	N-Ethylmaleimide Sensitive Factor, Vesicle Fusing ATPase	Protein Coding	45	GC17P046590	1.198108
HSPA6	Heat Shock Protein Family A (Hsp70) Member 6	Protein Coding	45	GC01P161524	1.191569
HSD17B2	Hydroxysteroid 17-Beta Dehydrogenase 2	Protein Coding	45	GC16P082068	1.19105
CTPS1	CTP Synthase 1	Protein Coding	45	GC01P040979	1.180421
NTHL1	Nth Like DNA Glycosylase 1	Protein Coding	45	GC16M003055	1.145792
TDP1	Tyrosyl-DNA Phosphodiesterase 1	Protein Coding	45	GC14P089954	1.143905
MARK2	Microtubule Affinity Regulating Kinase 2	Protein Coding	45	GC11P063838	1.130163
CTSZ	Cathepsin Z	Protein Coding	45	GC20M058995	1.125999
HAL	Histidine Ammonia-Lyase	Protein Coding	45	GC12M095972	1.12357
GLRA2	Glycine Receptor Alpha 2	Protein Coding	45	GC0XP014529	1.106148
RGS9	Regulator Of G Protein Signaling 9	Protein Coding	45	GC17P065104	1.10524
GIPR	Gastric Inhibitory Polypeptide Receptor	Protein Coding	45	GC19P045668	1.10034
CBFB	Core-Binding Factor Subunit Beta	Protein Coding	45	GC16P067063	1.075983
PAK6	P21 (RAC1) Activated Kinase 6	Protein Coding	45	GC15P040217	1.063615
UBE2D2	Ubiquitin Conjugating Enzyme E2 D2	Protein Coding	45	GC05P139526	1.061166
GNA14	G Protein Subunit Alpha 14	Protein Coding	45	GC09M077423	1.041662
TAOK1	TAO Kinase 1	Protein Coding	45	GC17P029654	1.02459
PSENEN	Presenilin Enhancer, Gamma-Secretase Subunit	Protein Coding	45	GC19P040365	1.023567
SPHK2	Sphingosine Kinase 2	Protein Coding	45	GC19P048619	1.018816
TUBB4A	Tubulin Beta 4A Class IVa	Protein Coding	45	GC19M006496	1.015165
RALBP1	RalA Binding Protein 1	Protein Coding	45	GC18P009465	0.989182
TDO2	Tryptophan 2,3-Dioxygenase	Protein Coding	45	GC04P155854	0.980142
ITPKB	Inositol-Trisphosphate 3-Kinase B	Protein Coding	45	GC01M226631	0.976222
DUSP10	Dual Specificity Phosphatase 10	Protein Coding	45	GC01M221701	0.976222
TFDP1	Transcription Factor Dp-1	Protein Coding	45	GC13P113584	0.96379
HTR3B	5-Hydroxytryptamine Receptor 3B	Protein Coding	45	GC11P113905	0.947305
PSMA1	Proteasome 20S Subunit Alpha 1	Protein Coding	45	GC11M014505	0.937009

SYN1	Synapsin I	Protein Coding	45	GC0XM047571	0.932395
CAMKK1	Calcium/Calmodulin Dependent Protein Kinase Kinase 1	Protein Coding	45	GC17M003860	0.930072
B4GALNT1	Beta-1,4-N-Acetyl-Galactosaminyltransferase 1	Protein Coding	45	GC12M057623	0.927006
GRAP2	GRB2 Related Adaptor Protein 2	Protein Coding	45	GC22P039901	0.906612
DGKA	Diacylglycerol Kinase Alpha	Protein Coding	45	GC12P055927	0.887979
PPP2R2A	Protein Phosphatase 2 Regulatory Subunit Balpha	Protein Coding	45	GC08P026292	0.88526
SIAH2	Siah E3 Ubiquitin Protein Ligase 2	Protein Coding	45	GC03M150741	0.839359
MTNR1A	Melatonin Receptor 1A	Protein Coding	45	GC04M186533	0.820761
GAK	Cyclin G Associated Kinase	Protein Coding	45	GC04M000849	0.803141
S100A10	S100 Calcium Binding Protein A10	Protein Coding	45	GC01M151955	0.793486
MAPRE1	Microtubule Associated Protein RP/EB Family Member 1	Protein Coding	45	GC20P032819	0.779749
TFF1	Trefoil Factor 1	Protein Coding	45	GC21M042362	0.778075
CCT5	Chaperonin Containing TCP1 Subunit 5	Protein Coding	45	GC05P010236	0.741344
FRK	Fyn Related Src Family Tyrosine Kinase	Protein Coding	45	GC06M115931	0.740964
USP14	Ubiquitin Specific Peptidase 14	Protein Coding	45	GC18P000158	0.702549
DHRS4	Dehydrogenase/Reductase 4	Protein Coding	45	GC14P023953	0.650211
GPR37	G Protein-Coupled Receptor 37	Protein Coding	45	GC07M124745	0.635757
ATP2C1	ATPase Secretory Pathway Ca2+ Transporting 1	Protein Coding	45	GC03P130850	0.629364
TUBB4B	Tubulin Beta 4B Class IVb	Protein Coding	45	GC09P137241	0.573719
STIP1	Stress Induced Phosphoprotein 1	Protein Coding	45	GC11P064196	0.542033
GLP2R	Glucagon Like Peptide 2 Receptor	Protein Coding	45	GC17P009822	0.529658
MAP4K5	Mitogen-Activated Protein Kinase Kinase Kinase Kinase 5	Protein Coding	45	GC14M050418	0.470739
GSTA3	Glutathione S-Transferase Alpha 3	Protein Coding	45	GC06M052896	0.272315
NPPB	Natriuretic Peptide B	Protein Coding	44	GC01M011858	56.41557
TBX1	T-Box Transcription Factor 1	Protein Coding	44	GC22P020087	38.92918
CITED2	Cbp/P300 Interacting Transactivator With Glu/Asp Rich Carboxy-Terminal Domain 2	Protein Coding	44	GC06M139371	30.00947
FABP3	Fatty Acid Binding Protein 3	Protein Coding	44	GC01M031365	28.62286
CAV3	Caveolin 3	Protein Coding	44	GC03P008733	24.61756
EYA4	EYA Transcriptional Coactivator And Phosphatase 4	Protein Coding	44	GC06P133240	23.836
HFE	Homeostatic Iron Regulator	Protein Coding	44	GC06P026087	23.7602
SCARB2	Scavenger Receptor Class B Member 2	Protein Coding	44	GC04M076158	23.6867
TLL1	Tolloid Like 1	Protein Coding	44	GC04P165873	23.56383
THBD	Thrombomodulin	Protein Coding	44	GC20M023026	22.88529
COL4A5	Collagen Type IV Alpha 5 Chain	Protein Coding	44	GC0XP108439	22.61462
HAMP	Hepcidin Antimicrobial Peptide	Protein Coding	44	GC19P040344	22.27149
MB	Myoglobin	Protein Coding	44	GC22M035606	20.08289
POLR1C	RNA Polymerase I And III Subunit C	Protein Coding	44	GC06P055413	19.65265
TSFM	Ts Translation Elongation Factor, Mitochondrial	Protein Coding	44	GC12P057778	19.61482
MEFV	MEFV Innate Immuity Regulator, Pyrin	Protein Coding	44	GC16M003757	18.49956
SURF1	SURF1 Cytochrome C Oxidase Assembly Factor	Protein Coding	44	GC09M133351	18.36863
SCN3B	Sodium Voltage-Gated Channel Beta Subunit 3	Protein Coding	44	GC11M123629	18.25071
NSD1	Nuclear Receptor Binding SET Domain Protein 1	Protein Coding	44	GC05P177134	18.0516
IL18	Interleukin 18	Protein Coding	44	GC11M112143	17.95632
CLCN5	Chloride Voltage-Gated Channel 5	Protein Coding	44	GC0XP049922	17.95263
LCN2	Lipocalin 2	Protein Coding	44	GC09P128149	17.87128
APOC3	Apolipoprotein C3	Protein Coding	44	GC11P116829	16.72515
COL4A4	Collagen Type IV Alpha 4 Chain	Protein Coding	44	GC02M226971	16.67414
AKAP9	A-Kinase Anchoring Protein 9	Protein Coding	44	GC07P091940	16.45245
NPHP1	Nephrocystin 1	Protein Coding	44	GC02M110122	16.2458
CD34	CD34 Molecule	Protein Coding	44	GC01M207880	15.83741
BMP6	Bone Morphogenetic Protein 6	Protein Coding	44	GC06P007726	15.68758
RETN	Resistin	Protein Coding	44	GC19P007669	15.49773
ACAD9	Acyl-CoA Dehydrogenase Family Member 9	Protein Coding	44	GC03P131450	15.39522

SELE	Selectin E	Protein Coding	44	GC01M169722	15.36139
PON2	Paraoxonase 2	Protein Coding	44	GC07M095404	15.23508
IL1A	Interleukin 1 Alpha	Protein Coding	44	GC02M112773	15.22256
AGTR2	Angiotensin II Receptor Type 2	Protein Coding	44	GC0XP116170	14.86297
MMACHC	Metabolism Of Cobalamin Associated C	Protein Coding	44	GC01P045500	14.81431
LAMA2	Laminin Subunit Alpha 2	Protein Coding	44	GC06P128863	14.63984
GHRL	Ghrelin And Obestatin Prepropeptide	Protein Coding	44	GC03M010285	14.62557
RAD51C	RAD51 Paralog C	Protein Coding	44	GC17P058692	14.40535
PRL	Prolactin	Protein Coding	44	GC06M022287	14.38787
SFTPB	Surfactant Protein B	Protein Coding	44	GC02M085657	14.26702
GRK5	G Protein-Coupled Receptor Kinase 5	Protein Coding	44	GC10P119207	14.20787
SCN4B	Sodium Voltage-Gated Channel Beta Subunit 4	Protein Coding	44	GC11M118134	14.18545
NEK1	NIMA Related Kinase 1	Protein Coding	44	GC04M169393	14.06988
GH1	Growth Hormone 1	Protein Coding	44	GC17M063917	13.66114
LTBP2	Latent Transforming Growth Factor Beta Binding Protein 2	Protein Coding	44	GC14M074498	13.52887
SCN2B	Sodium Voltage-Gated Channel Beta Subunit 2	Protein Coding	44	GC11M118163	13.21514
COX10	Cytochrome C Oxidase Assembly Factor Heme A:Farnesyltransferase COX10	Protein Coding	44	GC17P014069	13.18607
CMA1	Chymase 1	Protein Coding	44	GC14M024506	13.18264
PEX7	Peroxisomal Biogenesis Factor 7	Protein Coding	44	GC06P136822	13.14477
SFTPC	Surfactant Protein C	Protein Coding	44	GC08P022156	13.06334
OLR1	Oxidized Low Density Lipoprotein Receptor 1	Protein Coding	44	GC12M015685	13.02262
C4A	Complement C4A (Rodgers Blood Group)	Protein Coding	44	GC06P055224	12.9696
HNF1B	HNF1 Homeobox B	Protein Coding	44	GC17M037686	12.87329
SERPINA3	Serpin Family A Member 3	Protein Coding	44	GC14P094612	12.82391
CCL5	C-C Motif Chemokine Ligand 5	Protein Coding	44	GC17M035871	12.73251
APLNR	Apelin Receptor	Protein Coding	44	GC11M057233	12.72666
MYH3	Myosin Heavy Chain 3	Protein Coding	44	GC17M010628	12.68121
CLCNKB	Chloride Voltage-Gated Channel Kb	Protein Coding	44	GC01P016043	12.47503
RPS24	Ribosomal Protein S24	Protein Coding	44	GC10P078033	12.32036
PEX2	Peroxisomal Biogenesis Factor 2	Protein Coding	44	GC08M076980	12.26896
EFEMP2	EGF Containing Fibulin Extracellular Matrix Protein 2	Protein Coding	44	GC11M069431	12.19535
FANCG	FA Complementation Group G	Protein Coding	44	GC09M035073	12.18618
SOX4	SRY-Box Transcription Factor 4	Protein Coding	44	GC06P021593	12.18398
FIG4	FIG4 Phosphoinositide 5-Phosphatase	Protein Coding	44	GC06P109691	12.0893
SALL4	Spalt Like Transcription Factor 4	Protein Coding	44	GC20M051784	11.97322
GPD1L	Glycerol-3-Phosphate Dehydrogenase 1 Like	Protein Coding	44	GC03P032123	11.91646
GALC	Galactosylceramidase	Protein Coding	44	GC14M087837	11.91584
PHOX2B	Paired Like Homeobox 2B	Protein Coding	44	GC04M041746	11.88005
PPBP	Pro-Platelet Basic Protein	Protein Coding	44	GC04M073986	11.8312
HSPA1A	Heat Shock Protein Family A (Hsp70) Member 1A	Protein Coding	44	GC06P055219	11.77357
POSTN	Periostin	Protein Coding	44	GC13M037562	11.66178
ARSB	Arylsulfatase B	Protein Coding	44	GC05M078777	11.3509
RBCK1	RANBP2-Type And C3HC4-Type Zinc Finger Containing 1	Protein Coding	44	GC20P000407	11.29318
SKIV2L	Ski2 Like RNA Helicase	Protein Coding	44	GC06P055223	11.22987
CDH13	Cadherin 13	Protein Coding	44	GC16P082626	11.20783
UBE2T	Ubiquitin Conjugating Enzyme E2 T	Protein Coding	44	GC01M202332	11.1027
DYSF	Dysferlin	Protein Coding	44	GC02P071453	11.08673
DDX41	DEAD-Box Helicase 41	Protein Coding	44	GC05M177511	11.08021
ZIC2	Zic Family Member 2	Protein Coding	44	GC13P099981	11.03505
TNNT1	Troponin T1, Slow Skeletal Type	Protein Coding	44	GC19M055132	11.0097
TIMP2	TIMP Metallopeptidase Inhibitor 2	Protein Coding	44	GC17M078852	10.97837
RRAS	RAS Related	Protein Coding	44	GC19M049635	10.82967
AOC3	Amine Oxidase Copper Containing 3	Protein Coding	44	GC17P042851	10.44715

SLC29A3	Solute Carrier Family 29 Member 3	Protein Coding	44	GC10P071320	10.40891
PGF	Placental Growth Factor	Protein Coding	44	GC14M074941	10.3854
HTR2B	5-Hydroxytryptamine Receptor 2B	Protein Coding	44	GC02M231108	10.36029
PMPCA	Peptidase, Mitochondrial Processing Subunit Alpha	Protein Coding	44	GC09P136410	10.33991
XRCC4	X-Ray Repair Cross Complementing 4	Protein Coding	44	GC05P083077	10.30828
APOA2	Apolipoprotein A2	Protein Coding	44	GC01M161222	10.22089
SUFU	SUFU Negative Regulator Of Hedgehog Signaling	Protein Coding	44	GC10P102503	10.09154
PDE9A	Phosphodiesterase 9A	Protein Coding	44	GC21P042653	10.00624
APOH	Apolipoprotein H	Protein Coding	44	GC17M066212	9.914492
UCP3	Uncoupling Protein 3	Protein Coding	44	GC11M074000	9.866453
CCL11	C-C Motif Chemokine Ligand 11	Protein Coding	44	GC17P034285	9.832325
RREB1	Ras Responsive Element Binding Protein 1	Protein Coding	44	GC06P007107	9.811697
ACD	ACD Shelterin Complex Subunit And Telomerase Recruitment Factor	Protein Coding	44	GC16M067658	9.642193
HLA-DPB1	Major Histocompatibility Complex, Class II, DP Beta 1	Protein Coding	44	GC06P055237	9.490782
PEX14	Peroxisomal Biogenesis Factor 14	Protein Coding	44	GC01P010472	9.455439
PLCE1	Phospholipase C Epsilon 1	Protein Coding	44	GC10P093993	9.330445
MUS81	MUS81 Structure-Specific Endonuclease Subunit	Protein Coding	44	GC11P066258	9.295265
LDLRAP1	Low Density Lipoprotein Receptor Adaptor Protein 1	Protein Coding	44	GC01P025543	9.279179
GJA4	Gap Junction Protein Alpha 4	Protein Coding	44	GC01P034792	9.139734
SLC22A12	Solute Carrier Family 22 Member 12	Protein Coding	44	GC11P064609	9.137497
TAC1	Tachykinin Precursor 1	Protein Coding	44	GC07P097731	9.127626
LRP8	LDL Receptor Related Protein 8	Protein Coding	44	GC01M053243	9.106746
IGFBP1	Insulin Like Growth Factor Binding Protein 1	Protein Coding	44	GC07P046838	9.089514
SPOP	Speckle Type BTB/POZ Protein	Protein Coding	44	GC17M049598	9.068698
SLC39A8	Solute Carrier Family 39 Member 8	Protein Coding	44	GC04M102252	9.005608
AHSG	Alpha 2-HS Glycoprotein	Protein Coding	44	GC03P186612	8.905476
CSF2	Colony Stimulating Factor 2	Protein Coding	44	GC05P132073	8.805666
SMARCAL1	SWI/SNF Related, Matrix Associated, Actin Dependent Regulator Of Chromatin, Subfamily A Like 1	Protein Coding	44	GC02P216412	8.727587
GCLC	Glutamate-Cysteine Ligase Catalytic Subunit	Protein Coding	44	GC06M053497	8.655139
IL12B	Interleukin 12B	Protein Coding	44	GC05M159314	8.606498
GNE	Glucosamine (UDP-N-Acetyl)-2-Epimerase/N-Acetylmannosamine Kinase	Protein Coding	44	GC09M036214	8.568972
GC	GC Vitamin D Binding Protein	Protein Coding	44	GC04M071741	8.561975
C4B	Complement C4B (Chido Blood Group)	Protein Coding	44	GC06P032014	8.519609
SLC37A4	Solute Carrier Family 37 Member 4	Protein Coding	44	GC11M119024	8.472587
NPC1L1	NPC1 Like Intracellular Cholesterol Transporter 1	Protein Coding	44	GC07M044512	8.452458
GPR35	G Protein-Coupled Receptor 35	Protein Coding	44	GC02P240605	8.400156
MAFB	MAF BZIP Transcription Factor B	Protein Coding	44	GC20M040685	8.234821
THBS4	Thrombospondin 4	Protein Coding	44	GC05P079991	8.225578
AUH	AU RNA Binding Methylglutaconyl-CoA Hydratase	Protein Coding	44	GC09M091213	8.161004
POLR3A	RNA Polymerase III Subunit A	Protein Coding	44	GC10M077969	8.129007
IL12A	Interleukin 12A	Protein Coding	44	GC03P159988	8.087257
UQCRB	Ubiquinol-Cytochrome C Reductase Binding Protein	Protein Coding	44	GC08M096225	8.084478
UBR1	Ubiquitin Protein Ligase E3 Component N-Recognin 1	Protein Coding	44	GC15M042942	8.072392
PLAGL1	PLAG1 Like Zinc Finger 1	Protein Coding	44	GC06M143940	8.038343
UBE3B	Ubiquitin Protein Ligase E3B	Protein Coding	44	GC12P109477	8.029657
CARD14	Caspase Recruitment Domain Family Member 14	Protein Coding	44	GC17P080170	7.989122
AMH	Anti-Mullerian Hormone	Protein Coding	44	GC19P002251	7.941885
PURA	Purine Rich Element Binding Protein A	Protein Coding	44	GC05P140076	7.874142
TNXB	Tenascin XB	Protein Coding	44	GC06M046900	7.870105
SF3B4	Splicing Factor 3b Subunit 4	Protein Coding	44	GC01M149923	7.83174
ALG8	ALG8 Alpha-1,3-Glucosyltransferase	Protein Coding	44	GC11M078100	7.824267
MYBPC1	Myosin Binding Protein C1	Protein Coding	44	GC12P101568	7.790599
RCAN1	Regulator Of Calcineurin 1	Protein Coding	44	GC21M034513	7.731459

ATXN1	Ataxin 1	Protein Coding	44	GC06M016299	7.688787
GHRHR	Growth Hormone Releasing Hormone Receptor	Protein Coding	44	GC07P030938	7.608966
RELN	Reelin	Protein Coding	44	GC07M103471	7.5914
CRH	Corticotropin Releasing Hormone	Protein Coding	44	GC08M066176	7.578177
MAP1B	Microtubule Associated Protein 1B	Protein Coding	44	GC05P072107	7.56242
TRIM32	Tripartite Motif Containing 32	Protein Coding	44	GC09P116687	7.557446
GMPPB	GDP-Mannose Pyrophosphorylase B	Protein Coding	44	GC03M049716	7.554821
NDE1	NudE Neurodevelopment Protein 1	Protein Coding	44	GC16P015661	7.529681
JARID2	Jumonji And AT-Rich Interaction Domain Containing 2	Protein Coding	44	GC06P015265	7.512816
VANGL2	VANGL Planar Cell Polarity Protein 2	Protein Coding	44	GC01P160400	7.509497
SRP54	Signal Recognition Particle 54	Protein Coding	44	GC14P034981	7.504324
TCN2	Transcobalamin 2	Protein Coding	44	GC22P030606	7.489004
FLRT3	Fibronectin Leucine Rich Transmembrane Protein 3	Protein Coding	44	GC20M014322	7.375325
U2AF1	U2 Small Nuclear RNA Auxiliary Factor 1	Protein Coding	44	GC21M043092	7.306841
PROK2	Prokineticin 2	Protein Coding	44	GC03M071771	7.282185
SDC1	Syndecan 1	Protein Coding	44	GC02M020200	7.224493
SLC25A24	Solute Carrier Family 25 Member 24	Protein Coding	44	GC01M108134	7.223513
HIBCH	3-Hydroxyisobutyryl-CoA Hydrolase	Protein Coding	44	GC02M190189	7.2223
SOX6	SRY-Box Transcription Factor 6	Protein Coding	44	GC11M015949	7.185517
NR0B2	Nuclear Receptor Subfamily 0 Group B Member 2	Protein Coding	44	GC01M026922	7.171089
HUWE1	HECT, UBA And WWE Domain Containing E3 Ubiquitin Protein Ligase 1	Protein Coding	44	GC0XM053532	7.160389
MLX	MAX Dimerization Protein MLX	Protein Coding	44	GC17P042567	7.142956
CASQ1	Calsequestrin 1	Protein Coding	44	GC01P160190	7.125152
BCKDHB	Branched Chain Keto Acid Dehydrogenase E1 Subunit Beta	Protein Coding	44	GC06P080106	7.113696
FUT8	Fucosyltransferase 8	Protein Coding	44	GC14P065411	7.102467
SFTPD	Surfactant Protein D	Protein Coding	44	GC10M079937	7.089413
ANK3	Ankyrin 3	Protein Coding	44	GC10M060026	7.083866
GPX3	Glutathione Peroxidase 3	Protein Coding	44	GC05P150997	7.068248
POLR1A	RNA Polymerase I Subunit A	Protein Coding	44	GC02M086021	7.038406
CHRNE	Cholinergic Receptor Nicotinic Epsilon Subunit	Protein Coding	44	GC17M004897	7.030087
EBP	EBP Cholestenol Delta-Isomerase	Protein Coding	44	GC0XP048521	6.987325
IL3	Interleukin 3	Protein Coding	44	GC05P132060	6.947271
TXNL4A	Thioredoxin Like 4A	Protein Coding	44	GC18M079970	6.9156
POMT2	Protein O-Mannosyltransferase 2	Protein Coding	44	GC14M077274	6.915573
CXADR	CXADR Ig-Like Cell Adhesion Molecule	Protein Coding	44	GC21P017513	6.891815
FABP1	Fatty Acid Binding Protein 1	Protein Coding	44	GC02M088122	6.881074
HBEGF	Heparin Binding EGF Like Growth Factor	Protein Coding	44	GC05M140332	6.865393
CYP7A1	Cytochrome P450 Family 7 Subfamily A Member 1	Protein Coding	44	GC08M058476	6.844153
CSF1	Colony Stimulating Factor 1	Protein Coding	44	GC01P109911	6.807558
NID1	Nidogen 1	Protein Coding	44	GC01M235975	6.75461
RSPO2	R-Spondin 2	Protein Coding	44	GC08M107899	6.744713
RAB23	RAB23, Member RAS Oncogene Family	Protein Coding	44	GC06M057187	6.731538
INHA	Inhibin Subunit Alpha	Protein Coding	44	GC02P219569	6.706901
APBB1	Amyloid Beta Precursor Protein Binding Family B Member 1	Protein Coding	44	GC11M006396	6.705849
NDUFA4	NDUFA4 Mitochondrial Complex Associated	Protein Coding	44	GC07M010938	6.677272
IL17RD	Interleukin 17 Receptor D	Protein Coding	44	GC03M057089	6.604341
ALG1	ALG1 Chitobiosyldiphosphodolichol Beta-Mannosyltransferase	Protein Coding	44	GC16P005033	6.600845
AKAP13	A-Kinase Anchoring Protein 13	Protein Coding	44	GC15P085388	6.581455
DDX11	DEAD/H-Box Helicase 11	Protein Coding	44	GC12P031073	6.56104
SORT1	Sortilin 1	Protein Coding	44	GC01M109310	6.554314
TAGLN	Transgelin	Protein Coding	44	GC11P117199	6.553127
TSPO	Translocator Protein	Protein Coding	44	GC22P043151	6.552057
STK36	Serine/Threonine Kinase 36	Protein Coding	44	GC02P218672	6.541841

NARS2	Asparaginyl-TRNA Synthetase 2, Mitochondrial	Protein Coding	44	GC11M078435	6.515783
CNTNAP2	Contactin Associated Protein 2	Protein Coding	44	GC07P146116	6.507491
MAGI2	Membrane Associated Guanylate Kinase, WW And PDZ Domain Containing 2	Protein Coding	44	GC07M078017	6.506459
CD2AP	CD2 Associated Protein	Protein Coding	44	GC06P055437	6.457409
KAT6A	Lysine Acetyltransferase 6A	Protein Coding	44	GC08M041929	6.453182
SI	Sucrase-Isomaltase	Protein Coding	44	GC03M164978	6.44256
FGF12	Fibroblast Growth Factor 12	Protein Coding	44	GC03M192139	6.424629
HEY1	Hes Related Family BHLH Transcription Factor With YRPW Motif 1	Protein Coding	44	GC08M079764	6.379952
NDUFA9	NADH:Ubiquinone Oxidoreductase Subunit A9	Protein Coding	44	GC12P004649	6.308181
COL6A2	Collagen Type VI Alpha 2 Chain	Protein Coding	44	GC21P046098	6.289526
KCNA4	Potassium Voltage-Gated Channel Subfamily A Member 4	Protein Coding	44	GC11M030009	6.283066
DAXX	Death Domain Associated Protein	Protein Coding	44	GC06M033318	6.232013
PARVA	Parvin Alpha	Protein Coding	44	GC11P012398	6.231975
EPX	Eosinophil Peroxidase	Protein Coding	44	GC17P058192	6.231453
IRF6	Interferon Regulatory Factor 6	Protein Coding	44	GC01M209785	6.193283
IGFBP2	Insulin Like Growth Factor Binding Protein 2	Protein Coding	44	GC02P216632	6.185346
PLP1	Proteolipid Protein 1	Protein Coding	44	GC0XP103773	6.184122
SOX17	SRY-Box Transcription Factor 17	Protein Coding	44	GC08P054457	6.174992
DLG1	Discs Large MAGUK Scaffold Protein 1	Protein Coding	44	GC03M197042	6.169879
NDUFB10	NADH:Ubiquinone Oxidoreductase Subunit B10	Protein Coding	44	GC16P005573	6.168856
CDKL5	Cyclin Dependent Kinase Like 5	Protein Coding	44	GC0XP018425	6.154642
BCL11A	BAF Chromatin Remodeling Complex Subunit BCL11A	Protein Coding	44	GC02M060451	6.151353
NEUROD1	Neuronal Differentiation 1	Protein Coding	44	GC02M181673	6.127101
SH2B1	SH2B Adaptor Protein 1	Protein Coding	44	GC16P032268	6.098111
FOXP2	Forkhead Box P2	Protein Coding	44	GC07P114086	6.059308
SERPINF2	Serpin Family F Member 2	Protein Coding	44	GC17P001742	6.031898
SLC25A15	Solute Carrier Family 25 Member 15	Protein Coding	44	GC13P040789	5.998621
SOCS1	Suppressor Of Cytokine Signaling 1	Protein Coding	44	GC16M011255	5.956076
COL6A1	Collagen Type VI Alpha 1 Chain	Protein Coding	44	GC21P045981	5.947195
TBC1D4	TBC1 Domain Family Member 4	Protein Coding	44	GC13M075284	5.909931
ABCB7	ATP Binding Cassette Subfamily B Member 7	Protein Coding	44	GC0XM075053	5.897355
SLC46A1	Solute Carrier Family 46 Member 1	Protein Coding	44	GC17M031080	5.876679
DNASE1L3	Deoxyribonuclease 1 Like 3	Protein Coding	44	GC03M058192	5.849815
SEC23B	SEC23 Homolog B, COPII Coat Complex Component	Protein Coding	44	GC20P018507	5.799462
ATP6V1E1	ATPase H+ Transporting V1 Subunit E1	Protein Coding	44	GC22M017592	5.780791
FOXO3	Forkhead Box O3	Protein Coding	44	GC06P108559	5.714396
SEPSECS	Sep (O-Phosphoserine) TRNA:Sec (Selenocysteine) TRNA Synthase	Protein Coding	44	GC04M025121	5.684487
CKMT2	Creatine Kinase, Mitochondrial 2	Protein Coding	44	GC05P081232	5.677309
GNS	Glucosamine (N-Acetyl)-6-Sulfatase	Protein Coding	44	GC12M064713	5.6481
ARNT	Aryl Hydrocarbon Receptor Nuclear Translocator	Protein Coding	44	GC01M150809	5.574552
LRP4	LDL Receptor Related Protein 4	Protein Coding	44	GC11M068984	5.555186
NOL3	Nucleolar Protein 3	Protein Coding	44	GC16P067186	5.509201
LBP	Lipopolysaccharide Binding Protein	Protein Coding	44	GC20P038346	5.484121
TNFRSF13C	TNF Receptor Superfamily Member 13C	Protein Coding	44	GC22M048544	5.477283
SLC22A4	Solute Carrier Family 22 Member 4	Protein Coding	44	GC05P132294	5.474942
ATP6V0A4	ATPase H+ Transporting V0 Subunit A4	Protein Coding	44	GC07M138707	5.473962
HOXA13	Homeobox A13	Protein Coding	44	GC07M027347	5.461488
PFAS	Phosphoribosylformylglycinamidine Synthase	Protein Coding	44	GC17P008247	5.456665
COL17A1	Collagen Type XVII Alpha 1 Chain	Protein Coding	44	GC10M104031	5.395749
SLC39A4	Solute Carrier Family 39 Member 4	Protein Coding	44	GC08M144409	5.381556
ATP12A	ATPase H+/K+ Transporting Non-Gastric Alpha2 Subunit	Protein Coding	44	GC13P024680	5.377873
NAGA	Alpha-N-Acetylgalactosaminidase	Protein Coding	44	GC22M042058	5.36538
SLC5A7	Solute Carrier Family 5 Member 7	Protein Coding	44	GC02P107969	5.355899

BSG	Basigin (Ok Blood Group)	Protein Coding	44	GC19P000571	5.352329
M6PR	Mannose-6-Phosphate Receptor, Cation Dependent	Protein Coding	44	GC12M008969	5.351219
ERAP1	Endoplasmic Reticulum Aminopeptidase 1	Protein Coding	44	GC05M096760	5.348361
CACNB1	Calcium Voltage-Gated Channel Auxiliary Subunit Beta 1	Protein Coding	44	GC17M039173	5.338477
GPC6	Glypican 6	Protein Coding	44	GC13P093226	5.331788
RGS4	Regulator Of G Protein Signaling 4	Protein Coding	44	GC01P163038	5.310246
PAX4	Paired Box 4	Protein Coding	44	GC07M127610	5.302454
PIGQ	Phosphatidylinositol Glycan Anchor Biosynthesis Class Q	Protein Coding	44	GC16P005505	5.263219
SLC25A11	Solute Carrier Family 25 Member 11	Protein Coding	44	GC17M004937	5.253539
PLTP	Phospholipid Transfer Protein	Protein Coding	44	GC20M045898	5.245595
TNFRSF25	TNF Receptor Superfamily Member 25	Protein Coding	44	GC01M006460	5.239075
SMAD1	SMAD Family Member 1	Protein Coding	44	GC04P145481	5.232737
DDAH1	Dimethylarginine Dimethylaminohydrolase 1	Protein Coding	44	GC01M085318	5.231292
CLPB	Caseinolytic Mitochondrial Matrix Peptidase Chaperone Subunit B	Protein Coding	44	GC11M072286	5.205105
CHIT1	Chitinase 1	Protein Coding	44	GC01M203181	5.191239
GYS2	Glycogen Synthase 2	Protein Coding	44	GC12M021531	5.181007
KDM6B	Lysine Demethylase 6B	Protein Coding	44	GC17P007834	5.180019
AP1B1	Adaptor Related Protein Complex 1 Subunit Beta 1	Protein Coding	44	GC22M029327	5.179987
PPP1R12A	Protein Phosphatase 1 Regulatory Subunit 12A	Protein Coding	44	GC12M079773	5.177573
BDH1	3-Hydroxybutyrate Dehydrogenase 1	Protein Coding	44	GC03M197519	5.175067
LEFTY2	Left-Right Determination Factor 2	Protein Coding	44	GC01M225937	5.172751
TRH	Thyrotropin Releasing Hormone	Protein Coding	44	GC03P129974	5.165335
FGF9	Fibroblast Growth Factor 9	Protein Coding	44	GC13P021671	5.156599
ABCD4	ATP Binding Cassette Subfamily D Member 4	Protein Coding	44	GC14M074285	5.14945
CD86	CD86 Molecule	Protein Coding	44	GC03P122055	5.144756
PDGFA	Platelet Derived Growth Factor Subunit A	Protein Coding	44	GC07M000497	5.120582
CUX1	Cut Like Homeobox 1	Protein Coding	44	GC07P101815	5.113342
CPOX	Coproporphyrinogen Oxidase	Protein Coding	44	GC03M098576	5.104693
GPC1	Glypican 1	Protein Coding	44	GC02P240435	5.100681
NFATC3	Nuclear Factor Of Activated T Cells 3	Protein Coding	44	GC16P068119	5.100324
NFIB	Nuclear Factor I B	Protein Coding	44	GC09M014077	5.085374
HTT	Huntingtin	Protein Coding	44	GC04P003041	5.07717
COL6A3	Collagen Type VI Alpha 3 Chain	Protein Coding	44	GC02M237324	5.028591
CHI3L1	Chitinase 3 Like 1	Protein Coding	44	GC01M203148	5.015491
PPP1R1B	Protein Phosphatase 1 Regulatory Inhibitor Subunit 1B	Protein Coding	44	GC17P039626	5.00039
MAOB	Monoamine Oxidase B	Protein Coding	44	GC0XM043766	4.986892
KDM5C	Lysine Demethylase 5C	Protein Coding	44	GC0XM053176	4.962297
PALLD	Palladin, Cytoskeletal Associated Protein	Protein Coding	44	GC04P168497	4.937549
ORAI1	ORAI Calcium Release-Activated Calcium Modulator 1	Protein Coding	44	GC12P123580	4.897725
SPG7	SPG7 Matrix AAA Peptidase Subunit, Paraplegin	Protein Coding	44	GC16P089790	4.890097
AGPS	Alkylglycerone Phosphate Synthase	Protein Coding	44	GC02P177392	4.861251
VTN	Vitronectin	Protein Coding	44	GC17M031079	4.857717
ATG5	Autophagy Related 5	Protein Coding	44	GC06M106045	4.855865
SMAD7	SMAD Family Member 7	Protein Coding	44	GC18M048919	4.846852
MYL9	Myosin Light Chain 9	Protein Coding	44	GC20P036541	4.82095
EFNB2	Ephrin B2	Protein Coding	44	GC13M106489	4.785745
HSF2	Heat Shock Transcription Factor 2	Protein Coding	44	GC06P122399	4.768074
P2RX4	Purinergic Receptor P2X 4	Protein Coding	44	GC12P123577	4.745596
TJP1	Tight Junction Protein 1	Protein Coding	44	GC15M029699	4.743909
DISC1	DISC1 Scaffold Protein	Protein Coding	44	GC01P231626	4.721225
LTF	Lactotransferrin	Protein Coding	44	GC03M046435	4.71728
ACP1	Acid Phosphatase 1	Protein Coding	44	GC02P000254	4.714397
LAMA5	Laminin Subunit Alpha 5	Protein Coding	44	GC20M062307	4.709494

SLC39A14	Solute Carrier Family 39 Member 14	Protein Coding	44	GC08P022367	4.707688
DSG1	Desmoglein 1	Protein Coding	44	GC18P031318	4.707364
MYO1E	Myosin IE	Protein Coding	44	GC15M059132	4.706284
LMNB2	Lamin B2	Protein Coding	44	GC19M002470	4.705154
CYFIP2	Cytoplasmic FMR1 Interacting Protein 2	Protein Coding	44	GC05P157267	4.703887
GYPC	Glycophorin C (Gerbich Blood Group)	Protein Coding	44	GC02P126655	4.689248
STT3B	STT3 Oligosaccharyltransferase Complex Catalytic Subunit B	Protein Coding	44	GC03P031550	4.647542
GREM1	Gremlin 1, DAN Family BMP Antagonist	Protein Coding	44	GC15P033128	4.631705
RNASEH1	Ribonuclease H1	Protein Coding	44	GC02M003544	4.630427
PGM3	Phosphoglucomutase 3	Protein Coding	44	GC06M083161	4.612076
ATN1	Atrophin 1	Protein Coding	44	GC12P011868	4.609376
P2RX1	Purinergic Receptor P2X 1	Protein Coding	44	GC17M003896	4.59249
IL9	Interleukin 9	Protein Coding	44	GC05M135891	4.56352
GMPR	Guanosine Monophosphate Reductase	Protein Coding	44	GC06P016238	4.562368
RPS6	Ribosomal Protein S6	Protein Coding	44	GC09M019375	4.545978
EIF2AK1	Eukaryotic Translation Initiation Factor 2 Alpha Kinase 1	Protein Coding	44	GC07M006022	4.544479
UNC13D	Unc-13 Homolog D	Protein Coding	44	GC17M075827	4.531435
SIX6	SIX Homeobox 6	Protein Coding	44	GC14P060508	4.529675
UBQLN2	Ubiquilin 2	Protein Coding	44	GC0XP056563	4.529051
RHOQ	Ras Homolog Family Member Q	Protein Coding	44	GC02P046543	4.513768
MCCC1	Methylcrotonyl-CoA Carboxylase Subunit 1	Protein Coding	44	GC03M183015	4.511614
ITPKC	Inositol-Trisphosphate 3-Kinase C	Protein Coding	44	GC19P041025	4.505392
CEBPB	CCAAT Enhancer Binding Protein Beta	Protein Coding	44	GC20P050190	4.497319
DPP6	Dipeptidyl Peptidase Like 6	Protein Coding	44	GC07P153748	4.494804
PANK2	Pantothenate Kinase 2	Protein Coding	44	GC20P003887	4.476298
PDE3B	Phosphodiesterase 3B	Protein Coding	44	GC11P014643	4.467527
MDK	Midkine	Protein Coding	44	GC11P046380	4.465017
SYP	Synaptophysin	Protein Coding	44	GC0XM049187	4.457646
ARNT2	Aryl Hydrocarbon Receptor Nuclear Translocator 2	Protein Coding	44	GC15P080404	4.451525
FBLN1	Fibulin 1	Protein Coding	44	GC22P045502	4.439013
FOXG1	Forkhead Box G1	Protein Coding	44	GC14P028766	4.425432
LONP1	Lon Peptidase 1, Mitochondrial	Protein Coding	44	GC19M005691	4.423662
GAP43	Growth Associated Protein 43	Protein Coding	44	GC03P115623	4.385625
MLF1	Myeloid Leukemia Factor 1	Protein Coding	44	GC03P158571	4.357247
CXCR1	C-X-C Motif Chemokine Receptor 1	Protein Coding	44	GC02M218162	4.348122
RPS27A	Ribosomal Protein S27a	Protein Coding	44	GC02P055231	4.331159
CCM2	CCM2 Scaffold Protein	Protein Coding	44	GC07P044999	4.272449
DUOX2	Dual Oxidase 2	Protein Coding	44	GC15M045092	4.264553
SYT2	Synaptotagmin 2	Protein Coding	44	GC01M202559	4.243752
PRKCSH	Protein Kinase C Substrate 80K-H	Protein Coding	44	GC19P011435	4.225751
ARPC1B	Actin Related Protein 2/3 Complex Subunit 1B	Protein Coding	44	GC07P099374	4.219127
GABARAPL1	GABA Type A Receptor Associated Protein Like 1	Protein Coding	44	GC12P010212	4.217132
EIF2B5	Eukaryotic Translation Initiation Factor 2B Subunit Epsilon	Protein Coding	44	GC03P184135	4.214636
RIPK4	Receptor Interacting Serine/Threonine Kinase 4	Protein Coding	44	GC21M041739	4.208643
ACADL	Acyl-CoA Dehydrogenase Long Chain	Protein Coding	44	GC02M210187	4.203122
LPP	LIM Domain Containing Preferred Translocation Partner In Lipoma	Protein Coding	44	GC03P188153	4.200094
HSD3B1	Hydroxy-Delta-5-Steroid Dehydrogenase, 3 Beta- And Steroid Delta-Isomerase 1	Protein Coding	44	GC01P119507	4.175457
PDYN	Prodynorphin	Protein Coding	44	GC20M001978	4.160675
PIP5K1B	Phosphatidylinositol-4-Phosphate 5-Kinase Type 1 Beta	Protein Coding	44	GC09P068705	4.159816
PAM	Peptidylglycine Alpha-Amidating Monooxygenase	Protein Coding	44	GC05P102753	4.1569
IREB2	Iron Responsive Element Binding Protein 2	Protein Coding	44	GC15P078437	4.149734
SLC33A1	Solute Carrier Family 33 Member 1	Protein Coding	44	GC03M155821	4.138786
GTF2E2	General Transcription Factor IIE Subunit 2	Protein Coding	44	GC08M030578	4.122015

KCNC1	Potassium Voltage-Gated Channel Subfamily C Member 1	Protein Coding	44	GC11P017756	4.120757
FMO1	Flavin Containing Dimethylaniline Monoxygenase 1	Protein Coding	44	GC01P171248	4.05852
QKI	QKI, KH Domain Containing RNA Binding	Protein Coding	44	GC06P163414	4.042096
TAF15	TATA-Box Binding Protein Associated Factor 15	Protein Coding	44	GC17P035713	4.034181
LAMC1	Laminin Subunit Gamma 1	Protein Coding	44	GC01P182992	4.030171
NAT2	N-Acetyltransferase 2	Protein Coding	44	GC08P018391	4.02666
FXYD2	FXYD Domain Containing Ion Transport Regulator 2	Protein Coding	44	GC11M117800	4.017689
SEC63	SEC63 Homolog, Protein Translocation Regulator	Protein Coding	44	GC06M107867	3.993824
CLCN4	Chloride Voltage-Gated Channel 4	Protein Coding	44	GC0XP010085	3.991232
PTPN14	Protein Tyrosine Phosphatase Non-Receptor Type 14	Protein Coding	44	GC01M214348	3.974618
HLCS	Holocarboxylase Synthetase	Protein Coding	44	GC21M036750	3.971502
NMT1	N-Myristoyltransferase 1	Protein Coding	44	GC17P044958	3.938707
FAF1	Fas Associated Factor 1	Protein Coding	44	GC01M050439	3.937647
TSPAN12	Tetraspanin 12	Protein Coding	44	GC07M120787	3.930127
SH3GL2	SH3 Domain Containing GRB2 Like 2, Endophilin A1	Protein Coding	44	GC09P017569	3.929633
IRF4	Interferon Regulatory Factor 4	Protein Coding	44	GC06P000391	3.927938
RAPGEF4	Rap Guanine Nucleotide Exchange Factor 4	Protein Coding	44	GC02P172735	3.919166
EGR1	Early Growth Response 1	Protein Coding	44	GC05P138465	3.917998
SLC26A4	Solute Carrier Family 26 Member 4	Protein Coding	44	GC07P107660	3.910142
NRIP1	Nuclear Receptor Interacting Protein 1	Protein Coding	44	GC21M014961	3.905796
CLOCK	Clock Circadian Regulator	Protein Coding	44	GC04M055427	3.904148
FARS2	Phenylalanyl-TRNA Synthetase 2, Mitochondrial	Protein Coding	44	GC06P005261	3.892247
SPTBN1	Spectrin Beta, Non-Erythrocytic 1	Protein Coding	44	GC02P054456	3.891421
ROBO3	Roundabout Guidance Receptor 3	Protein Coding	44	GC11P124865	3.881628
WNK4	WNK Lysine Deficient Protein Kinase 4	Protein Coding	44	GC17P044667	3.853327
KCNH7	Potassium Voltage-Gated Channel Subfamily H Member 7	Protein Coding	44	GC02M162371	3.850026
TRPM1	Transient Receptor Potential Cation Channel Subfamily M Member 1	Protein Coding	44	GC15M031001	3.844151
CARTPT	CART Prepropeptide	Protein Coding	44	GC05P071719	3.842565
MGST2	Microsomal Glutathione S-Transferase 2	Protein Coding	44	GC04P139665	3.841892
RNASEH2A	Ribonuclease H2 Subunit A	Protein Coding	44	GC19P012896	3.841866
UBC	Ubiquitin C	Protein Coding	44	GC12M124911	3.835594
GAS6	Growth Arrest Specific 6	Protein Coding	44	GC13M113820	3.823129
LIPG	Lipase G, Endothelial Type	Protein Coding	44	GC18P049560	3.818558
CUL4B	Cullin 4B	Protein Coding	44	GC0XM120524	3.811038
EIF2B1	Eukaryotic Translation Initiation Factor 2B Subunit Alpha	Protein Coding	44	GC12M123620	3.786449
POT1	Protection Of Telomeres 1	Protein Coding	44	GC07M124822	3.781361
VASP	Vasodilator Stimulated Phosphoprotein	Protein Coding	44	GC19P045507	3.77903
MTHFS	Methenyltetrahydrofolate Synthetase	Protein Coding	44	GC15M079833	3.760291
A4GALT	Alpha 1,4-Galactosyltransferase (P Blood Group)	Protein Coding	44	GC22M042692	3.757419
PPIF	Peptidylprolyl Isomerase F	Protein Coding	44	GC10P085378	3.756
IGF2BP2	Insulin Like Growth Factor 2 MRNA Binding Protein 2	Protein Coding	44	GC03M185643	3.753466
FBL	Fibrillarin	Protein Coding	44	GC19M039834	3.745213
SEC23A	SEC23 Homolog A, COPII Coat Complex Component	Protein Coding	44	GC14M039031	3.734537
SLC16A7	Solute Carrier Family 16 Member 7	Protein Coding	44	GC12P059596	3.733234
CDT1	Chromatin Licensing And DNA Replication Factor 1	Protein Coding	44	GC16P088803	3.725168
FGF4	Fibroblast Growth Factor 4	Protein Coding	44	GC11M070438	3.702255
SOST	Sclerostin	Protein Coding	44	GC17M043753	3.702168
DCLK1	Doublecortin Like Kinase 1	Protein Coding	44	GC13M035768	3.694902
CHSY1	Chondroitin Sulfate Synthase 1	Protein Coding	44	GC15M101175	3.681625
BAMBI	BMP And Activin Membrane Bound Inhibitor	Protein Coding	44	GC10P028685	3.63184
SLC1A7	Solute Carrier Family 1 Member 7	Protein Coding	44	GC01M053087	3.624312
SLC5A3	Solute Carrier Family 5 Member 3	Protein Coding	44	GC21P034119	3.6161
LIAS	Lipoic Acid Synthetase	Protein Coding	44	GC04P039469	3.609636

TRPV3	Transient Receptor Potential Cation Channel Subfamily V Member 3	Protein Coding	44	GC17M003515	3.608094
RAB10	RAB10, Member RAS Oncogene Family	Protein Coding	44	GC02P026033	3.607813
ADH1B	Alcohol Dehydrogenase 1B (Class I), Beta Polypeptide	Protein Coding	44	GC04M099304	3.592238
BRCC3	BRCA1/BRCA2-Containing Complex Subunit 3	Protein Coding	44	GC0XP155071	3.575387
SPINK1	Serine Peptidase Inhibitor Kazal Type 1	Protein Coding	44	GC05M147825	3.558928
CRADD	CASP2 And RIPK1 Domain Containing Adaptor With Death Domain	Protein Coding	44	GC12P093677	3.557918
DAB2	DAB Adaptor Protein 2	Protein Coding	44	GC05M039371	3.555059
XRCC1	X-Ray Repair Cross Complementing 1	Protein Coding	44	GC19M043543	3.549915
SFRP4	Secreted Frizzled Related Protein 4	Protein Coding	44	GC07M037912	3.532295
PER2	Period Circadian Regulator 2	Protein Coding	44	GC02M238244	3.530651
CYSLTR1	Cysteinyl Leukotriene Receptor 1	Protein Coding	44	GC0XM078271	3.52896
UBB	Ubiquitin B	Protein Coding	44	GC17P016380	3.527614
STK39	Serine/Threonine Kinase 39	Protein Coding	44	GC02M167954	3.523954
HAVCR2	Hepatitis A Virus Cellular Receptor 2	Protein Coding	44	GC05M157063	3.521597
DNAJC5	DnaJ Heat Shock Protein Family (Hsp40) Member C5	Protein Coding	44	GC20P063895	3.510508
LGALS1	Galectin 1	Protein Coding	44	GC22P037675	3.508776
LOXL2	Lysyl Oxidase Like 2	Protein Coding	44	GC08M023296	3.505578
RPS2	Ribosomal Protein S2	Protein Coding	44	GC16M003048	3.495409
MCM6	Minichromosome Maintenance Complex Component 6	Protein Coding	44	GC02M135839	3.487376
MBTPS2	Membrane Bound Transcription Factor Peptidase, Site 2	Protein Coding	44	GC0XP021839	3.48573
PTK7	Protein Tyrosine Kinase 7 (Inactive)	Protein Coding	44	GC06P043076	3.483581
MAPKAP1	MAPK Associated Protein 1	Protein Coding	44	GC09M125437	3.472265
PMPCB	Peptidase, Mitochondrial Processing Subunit Beta	Protein Coding	44	GC07P103297	3.459615
EXO1	Exonuclease 1	Protein Coding	44	GC01P241847	3.458042
PDE1A	Phosphodiesterase 1A	Protein Coding	44	GC02M182140	3.457249
SLC25A21	Solute Carrier Family 25 Member 21	Protein Coding	44	GC14M036677	3.455487
ORC1	Origin Recognition Complex Subunit 1	Protein Coding	44	GC01M052372	3.446943
HES1	Hes Family BHLH Transcription Factor 1	Protein Coding	44	GC03P194136	3.444179
TMPRSS6	Transmembrane Serine Protease 6	Protein Coding	44	GC22M037066	3.43851
PLA2G5	Phospholipase A2 Group V	Protein Coding	44	GC01P020028	3.433203
ABCC5	ATP Binding Cassette Subfamily C Member 5	Protein Coding	44	GC03M183919	3.432581
HTRA1	HtrA Serine Peptidase 1	Protein Coding	44	GC10P122461	3.420322
KPNB1	Karyopherin Subunit Beta 1	Protein Coding	44	GC17P047649	3.409275
TLK1	Tousled Like Kinase 1	Protein Coding	44	GC02M170990	3.401035
CFP	Complement Factor Properdin	Protein Coding	44	GC0XM047624	3.400492
MSMO1	Methylsterol Monooxygenase 1	Protein Coding	44	GC04P165327	3.388346
TRIM28	Tripartite Motif Containing 28	Protein Coding	44	GC19P058544	3.376757
AGRP	Agouti Related Neuropeptide	Protein Coding	44	GC16M067482	3.371566
XPNPEP2	X-Prolyl Aminopeptidase 2	Protein Coding	44	GC0XP129738	3.341149
C5AR1	Complement C5a Receptor 1	Protein Coding	44	GC19P047290	3.321642
VAV3	Vav Guanine Nucleotide Exchange Factor 3	Protein Coding	44	GC01M107571	3.312758
KCNK1	Potassium Two Pore Domain Channel Subfamily K Member 1	Protein Coding	44	GC01P233614	3.309997
TRAF3IP2	TRAF3 Interacting Protein 2	Protein Coding	44	GC06M111555	3.306129
PNPT1	Polyribonucleotide Nucleotidyltransferase 1	Protein Coding	44	GC02M055634	3.272121
USF1	Upstream Transcription Factor 1	Protein Coding	44	GC01M161039	3.271864
UBTF	Upstream Binding Transcription Factor	Protein Coding	44	GC17M044205	3.258316
LPAR3	Lysophosphatidic Acid Receptor 3	Protein Coding	44	GC01M084811	3.25398
VAMP2	Vesicle Associated Membrane Protein 2	Protein Coding	44	GC17M009187	3.248811
SMYD2	SET And MYND Domain Containing 2	Protein Coding	44	GC01P214281	3.214677
PLA2G10	Phospholipase A2 Group X	Protein Coding	44	GC16M014672	3.200002
GFRA1	GDNF Family Receptor Alpha 1	Protein Coding	44	GC10M116056	3.198158
ALOX12	Arachidonate 12-Lipoxygenase, 12S Type	Protein Coding	44	GC17P006995	3.195235
JAM3	Junctional Adhesion Molecule 3	Protein Coding	44	GC11P134068	3.191403

SERPINA6	Serpin Family A Member 6	Protein Coding	44	GC14M095823	3.190239
NUP98	Nucleoporin 98 And 96 Precursor	Protein Coding	44	GC11M003671	3.182796
SLC18A3	Solute Carrier Family 18 Member A3	Protein Coding	44	GC10P049610	3.181518
EFNA5	Ephrin A5	Protein Coding	44	GC05M107376	3.178241
KLF6	Kruppel Like Factor 6	Protein Coding	44	GC10M003779	3.178065
IQGAP1	IQ Motif Containing GTPase Activating Protein 1	Protein Coding	44	GC15P090388	3.168692
COL9A3	Collagen Type IX Alpha 3 Chain	Protein Coding	44	GC20P062816	3.16242
ACOX2	Acyl-CoA Oxidase 2	Protein Coding	44	GC03M058490	3.152705
SRD5A3	Steroid 5 Alpha-Reductase 3	Protein Coding	44	GC04P055346	3.148641
CNDP1	Carnosine Dipeptidase 1	Protein Coding	44	GC18P074534	3.140715
PRPF3	Pre-MRNA Processing Factor 3	Protein Coding	44	GC01P150321	3.118991
CARD9	Caspase Recruitment Domain Family Member 9	Protein Coding	44	GC09M136361	3.107021
PPOX	Protoporphyrinogen Oxidase	Protein Coding	44	GC01P161184	3.104373
SLCO1B3	Solute Carrier Organic Anion Transporter Family Member 1B3	Protein Coding	44	GC12P020810	3.102207
TNKS	Tankyrase	Protein Coding	44	GC08P009555	3.098027
TIMM8A	Translocase Of Inner Mitochondrial Membrane 8A	Protein Coding	44	GC0XM101345	3.090918
EFNA1	Ephrin A1	Protein Coding	44	GC01P155127	3.08874
ECM1	Extracellular Matrix Protein 1	Protein Coding	44	GC01P150508	3.085191
GRK4	G Protein-Coupled Receptor Kinase 4	Protein Coding	44	GC04P002963	3.082743
EEF1A1	Eukaryotic Translation Elongation Factor 1 Alpha 1	Protein Coding	44	GC06M073515	3.082668
WNT2	Wnt Family Member 2	Protein Coding	44	GC07M117276	3.080512
DDX1	DEAD-Box Helicase 1	Protein Coding	44	GC02P015591	3.076366
POU1F1	POU Class 1 Homeobox 1	Protein Coding	44	GC03M087259	3.069552
RAMP1	Receptor Activity Modifying Protein 1	Protein Coding	44	GC02P237858	3.061434
GBF1	Golgi Brefeldin A Resistant Guanine Nucleotide Exchange Factor 1	Protein Coding	44	GC10P102245	3.057567
TNNT3	Troponin T3, Fast Skeletal Type	Protein Coding	44	GC11P001920	3.051852
BEST1	Bestrophin 1	Protein Coding	44	GC11P061949	3.030311
FZD7	Frizzled Class Receptor 7	Protein Coding	44	GC02P202034	3.025588
SLC22A1	Solute Carrier Family 22 Member 1	Protein Coding	44	GC06P160121	3.024236
ELOVL5	ELOVL Fatty Acid Elongase 5	Protein Coding	44	GC06M053240	3.000614
NCOR1	Nuclear Receptor Corepressor 1	Protein Coding	44	GC17M016029	2.995603
KDM5B	Lysine Demethylase 5B	Protein Coding	44	GC01M202696	2.995302
TNFRSF12A	TNF Receptor Superfamily Member 12A	Protein Coding	44	GC16P003018	2.99043
GUCY2D	Guanylate Cyclase 2D, Retinal	Protein Coding	44	GC17P008002	2.982198
PDK2	Pyruvate Dehydrogenase Kinase 2	Protein Coding	44	GC17P050095	2.971796
ARRB1	Arrestin Beta 1	Protein Coding	44	GC11M075261	2.970446
KCNQ5	Potassium Voltage-Gated Channel Subfamily Q Member 5	Protein Coding	44	GC06P072621	2.966852
EWSR1	EWS RNA Binding Protein 1	Protein Coding	44	GC22P029269	2.966658
DGAT2	Diacylglycerol O-Acyltransferase 2	Protein Coding	44	GC11P075759	2.96057
EMX2	Empty Spiracles Homeobox 2	Protein Coding	44	GC10P117542	2.952031
MC3R	Melanocortin 3 Receptor	Protein Coding	44	GC20P056248	2.950393
NRP2	Neuropilin 2	Protein Coding	44	GC02P205681	2.950308
MPZ	Myelin Protein Zero	Protein Coding	44	GC01M161304	2.947016
CCL20	C-C Motif Chemokine Ligand 20	Protein Coding	44	GC02P227814	2.934269
MRPS16	Mitochondrial Ribosomal Protein S16	Protein Coding	44	GC10M073248	2.91442
RAB2A	RAB2A, Member RAS Oncogene Family	Protein Coding	44	GC08P060516	2.91431
E2F1	E2F Transcription Factor 1	Protein Coding	44	GC20M033675	2.900418
ABCG1	ATP Binding Cassette Subfamily G Member 1	Protein Coding	44	GC21P042199	2.899267
NEDD9	Neural Precursor Cell Expressed, Developmentally Down-Regulated 9	Protein Coding	44	GC06M011183	2.894075
PTAFR	Platelet Activating Factor Receptor	Protein Coding	44	GC01M028147	2.890718
POLR3B	RNA Polymerase III Subunit B	Protein Coding	44	GC12P106357	2.876225
CHD2	Chromodomain Helicase DNA Binding Protein 2	Protein Coding	44	GC15P092900	2.864921
UGT8	UDP Glycosyltransferase 8	Protein Coding	44	GC04P114598	2.864561

ACAA1	Acetyl-CoA Acyltransferase 1	Protein Coding	44	GC03M038103	2.864406
FSHB	Follicle Stimulating Hormone Subunit Beta	Protein Coding	44	GC11P030210	2.85525
CBR3	Carbonyl Reductase 3	Protein Coding	44	GC21P036134	2.851292
S100A4	S100 Calcium Binding Protein A4	Protein Coding	44	GC01M153543	2.84597
TRAP1	TNF Receptor Associated Protein 1	Protein Coding	44	GC16M003667	2.838635
SRR	Serine Racemase	Protein Coding	44	GC17P002303	2.837923
COLEC11	Collectin Subfamily Member 11	Protein Coding	44	GC02P003594	2.83285
RBL2	RB Transcriptional Corepressor Like 2	Protein Coding	44	GC16P053433	2.82544
ZDHHC9	Zinc Finger DHHC-Type Palmitoyltransferase 9	Protein Coding	44	GC0XM129803	2.814287
CYP2U1	Cytochrome P450 Family 2 Subfamily U Member 1	Protein Coding	44	GC04P107931	2.803968
GOSR2	Golgi SNAP Receptor Complex Member 2	Protein Coding	44	GC17P046924	2.77705
PLIN2	Perilipin 2	Protein Coding	44	GC09M019127	2.76406
KCNH5	Potassium Voltage-Gated Channel Subfamily H Member 5	Protein Coding	44	GC14M062699	2.755761
SERPINB2	Serpin Family B Member 2	Protein Coding	44	GC18P063871	2.726389
IL12RB2	Interleukin 12 Receptor Subunit Beta 2	Protein Coding	44	GC01P067307	2.714305
PER3	Period Circadian Regulator 3	Protein Coding	44	GC01P007785	2.703877
HARS2	Histidyl-TRNA Synthetase 2, Mitochondrial	Protein Coding	44	GC05P143875	2.699594
PSMD4	Proteasome 26S Subunit Ubiquitin Receptor, Non-ATPase 4	Protein Coding	44	GC01P151256	2.690946
MTHFD2	Methylenetetrahydrofolate Dehydrogenase (NADP+ Dependent) 2, Methenyltetrahydrofolate Cyclohydrolase	Protein Coding	44	GC02P074186	2.688494
INPP5K	Inositol Polyphosphate-5-Phosphatase K	Protein Coding	44	GC17M001494	2.687083
AZGP1	Alpha-2-Glycoprotein 1, Zinc-Binding	Protein Coding	44	GC07M099967	2.686445
MGAT1	Alpha-1,3-Mannosyl-Glycoprotein 2-Beta-N-Acetylglucosaminyltransferase	Protein Coding	44	GC05M180936	2.678488
SLC7A1	Solute Carrier Family 7 Member 1	Protein Coding	44	GC13M029509	2.666215
GPR68	G Protein-Coupled Receptor 68	Protein Coding	44	GC14M091232	2.656241
GABARAPL2	GABA Type A Receptor Associated Protein Like 2	Protein Coding	44	GC16P075566	2.654346
KREMEN1	Kringle Containing Transmembrane Protein 1	Protein Coding	44	GC22P029073	2.651861
CA14	Carbonic Anhydrase 14	Protein Coding	44	GC01P150258	2.645988
PRDX3	Peroxiredoxin 3	Protein Coding	44	GC10M119167	2.6459
ENTPD5	Ectonucleoside Triphosphate Diphosphohydrolase 5 (Inactive)	Protein Coding	44	GC14M073958	2.635334
CTTN	Cortactin	Protein Coding	44	GC11P070398	2.632207
FGF3	Fibroblast Growth Factor 3	Protein Coding	44	GC11M070491	2.632169
HYOU1	Hypoxia Up-Regulated 1	Protein Coding	44	GC11M119045	2.630545
DNAJC6	DnaJ Heat Shock Protein Family (Hsp40) Member C6	Protein Coding	44	GC01P065248	2.615771
TLR6	Toll Like Receptor 6	Protein Coding	44	GC04M038828	2.607621
CD47	CD47 Molecule	Protein Coding	44	GC03M108043	2.60322
CD33	CD33 Molecule	Protein Coding	44	GC19P051212	2.590419
LNX1	Ligand Of Numb-Protein X 1	Protein Coding	44	GC04M053459	2.585358
MSH3	MutS Homolog 3	Protein Coding	44	GC05P080654	2.579024
SLCO2B1	Solute Carrier Organic Anion Transporter Family Member 2B1	Protein Coding	44	GC11P076259	2.578036
MSRB3	Methionine Sulfoxide Reductase B3	Protein Coding	44	GC12P065279	2.54858
TNFRSF17	TNF Receptor Superfamily Member 17	Protein Coding	44	GC16P011965	2.543565
SELENBP1	Selenium Binding Protein 1	Protein Coding	44	GC01M151364	2.531811
GPNMB	Glycoprotein Nmb	Protein Coding	44	GC07P023238	2.531684
CACNA1I	Calcium Voltage-Gated Channel Subunit Alpha1 I	Protein Coding	44	GC22P039570	2.529122
NRF1	Nuclear Respiratory Factor 1	Protein Coding	44	GC07P129611	2.52564
DDIT4	DNA Damage Inducible Transcript 4	Protein Coding	44	GC10P072273	2.513475
NUP214	Nucleoporin 214	Protein Coding	44	GC09P131125	2.513062
TRIM25	Tripartite Motif Containing 25	Protein Coding	44	GC17M056836	2.509337
CES2	Carboxylesterase 2	Protein Coding	44	GC16P066934	2.477925
PLXNA1	Plexin A1	Protein Coding	44	GC03P126988	2.456362
GAB2	GRB2 Associated Binding Protein 2	Protein Coding	44	GC11M078215	2.454618
SOAT2	Sterol O-Acyltransferase 2	Protein Coding	44	GC12P053103	2.454422
RPL21	Ribosomal Protein L21	Protein Coding	44	GC13P027251	2.441622

ATG16L1	Autophagy Related 16 Like 1	Protein Coding	44	GC02P233220	2.435684
CASP5	Caspase 5	Protein Coding	44	GC11M104995	2.427927
RUNX3	RUNX Family Transcription Factor 3	Protein Coding	44	GC01M024899	2.421446
EGR2	Early Growth Response 2	Protein Coding	44	GC10M062811	2.41483
COL10A1	Collagen Type X Alpha 1 Chain	Protein Coding	44	GC06M116118	2.412211
CTNNA2	Catenin Alpha 2	Protein Coding	44	GC02P079185	2.410353
PAX1	Paired Box 1	Protein Coding	44	GC20P021705	2.391899
RPL22	Ribosomal Protein L22	Protein Coding	44	GC01M006179	2.389294
EIF4A3	Eukaryotic Translation Initiation Factor 4A3	Protein Coding	44	GC17M080135	2.385961
CYP4A11	Cytochrome P450 Family 4 Subfamily A Member 11	Protein Coding	44	GC01M046929	2.383992
GCSH	Glycine Cleavage System Protein H	Protein Coding	44	GC16M081081	2.382455
XPR1	Xenotropic And Polytropic Retrovirus Receptor 1	Protein Coding	44	GC01P180632	2.377306
SLIT1	Slit Guidance Ligand 1	Protein Coding	44	GC10M096998	2.371754
PDCD6IP	Programmed Cell Death 6 Interacting Protein	Protein Coding	44	GC03P033798	2.353488
ARSG	Arylsulfatase G	Protein Coding	44	GC17P068259	2.349484
MTMR14	Myotubularin Related Protein 14	Protein Coding	44	GC03P009649	2.343055
KIF23	Kinesin Family Member 23	Protein Coding	44	GC15P069414	2.337933
CNP	2',3'-Cyclic Nucleotide 3' Phosphodiesterase	Protein Coding	44	GC17P041966	2.32536
CA5A	Carbonic Anhydrase 5A	Protein Coding	44	GC16M087882	2.317784
MBTPS1	Membrane Bound Transcription Factor Peptidase, Site 1	Protein Coding	44	GC16M084053	2.316047
RHOH	Ras Homolog Family Member H	Protein Coding	44	GC04P040192	2.313941
BMI1	BMI1 Proto-Oncogene, Polycomb Ring Finger	Protein Coding	44	GC10P022326	2.313077
UQCRC1	Ubiquinol-Cytochrome C Reductase Core Protein 1	Protein Coding	44	GC03M048598	2.305777
GPX7	Glutathione Peroxidase 7	Protein Coding	44	GC01P052602	2.302178
DAB1	DAB Adaptor Protein 1	Protein Coding	44	GC01M056994	2.294578
DNAJB1	DnaJ Heat Shock Protein Family (Hsp40) Member B1	Protein Coding	44	GC19M014514	2.282182
CD9	CD9 Molecule	Protein Coding	44	GC12P011813	2.282028
HDAC11	Histone Deacetylase 11	Protein Coding	44	GC03P013478	2.272834
PRKAG3	Protein Kinase AMP-Activated Non-Catalytic Subunit Gamma 3	Protein Coding	44	GC02M218823	2.269202
ENPP2	Ectonucleotide Pyrophosphatase/Phosphodiesterase 2	Protein Coding	44	GC08M119556	2.267234
SLC30A2	Solute Carrier Family 30 Member 2	Protein Coding	44	GC01M026048	2.260217
NLRP12	NLR Family Pyrin Domain Containing 12	Protein Coding	44	GC19M053793	2.247172
SIRPA	Signal Regulatory Protein Alpha	Protein Coding	44	GC20P001894	2.234781
TSPAN7	Tetraspanin 7	Protein Coding	44	GC0XP038561	2.233714
PTPN13	Protein Tyrosine Phosphatase Non-Receptor Type 13	Protein Coding	44	GC04P086594	2.212128
CRY1	Cryptochrome Circadian Regulator 1	Protein Coding	44	GC12M106991	2.211958
TRAF1	TNF Receptor Associated Factor 1	Protein Coding	44	GC09M120902	2.204473
TNKS2	Tankyrase 2	Protein Coding	44	GC10P091798	2.195145
FXR1	FMR1 Autosomal Homolog 1	Protein Coding	44	GC03P180868	2.192703
CNGA3	Cyclic Nucleotide Gated Channel Subunit Alpha 3	Protein Coding	44	GC02P098329	2.179497
EEA1	Early Endosome Antigen 1	Protein Coding	44	GC12M092770	2.16002
NOP56	NOP56 Ribonucleoprotein	Protein Coding	44	GC20P003251	2.15795
ID1	Inhibitor Of DNA Binding 1, HLH Protein	Protein Coding	44	GC20P031605	2.154853
TOLLIP	Toll Interacting Protein	Protein Coding	44	GC11M001274	2.15171
TTBK2	Tau Tubulin Kinase 2	Protein Coding	44	GC15M042738	2.149576
MTMR2	Myotubularin Related Protein 2	Protein Coding	44	GC11M095822	2.148917
DKK2	Dickkopf WNT Signaling Pathway Inhibitor 2	Protein Coding	44	GC04M106921	2.138488
SERPINB8	Serpin Family B Member 8	Protein Coding	44	GC18P063969	2.130477
TLE4	TLE Family Member 4, Transcriptional Corepressor	Protein Coding	44	GC09P079571	2.129466
DNM3	Dynamin 3	Protein Coding	44	GC01P171841	2.120309
RPTOR	Regulatory Associated Protein Of MTOR Complex 1	Protein Coding	44	GC17P080544	2.113073
SLC25A19	Solute Carrier Family 25 Member 19	Protein Coding	44	GC17M075273	2.102506
AQP9	Aquaporin 9	Protein Coding	44	GC15P058138	2.101985

IRF2	Interferon Regulatory Factor 2	Protein Coding	44	GC04M184387	2.101655
PDGFC	Platelet Derived Growth Factor C	Protein Coding	44	GC04M156760	2.097953
NLRP7	NLR Family Pyrin Domain Containing 7	Protein Coding	44	GC19M054923	2.090116
TRHR	Thyrotropin Releasing Hormone Receptor	Protein Coding	44	GC08P109084	2.083061
AVPR1B	Arginine Vasopressin Receptor 1B	Protein Coding	44	GC01M206109	2.078032
FGF19	Fibroblast Growth Factor 19	Protein Coding	44	GC11M070382	2.07688
PRSS8	Serine Protease 8	Protein Coding	44	GC16M031152	2.069953
MYO1C	Myosin IC	Protein Coding	44	GC17M001464	2.066301
ESD	Esterase D	Protein Coding	44	GC13M046771	2.065822
ARF4	ADP Ribosylation Factor 4	Protein Coding	44	GC03M057559	2.056681
SMARCC1	SWI/SNF Related, Matrix Associated, Actin Dependent Regulator Of Chromatin Subfamily C Member 1	Protein Coding	44	GC03M047585	2.055252
B3GAT1	Beta-1,3-Glucuronyltransferase 1	Protein Coding	44	GC11M134378	2.054331
TLE3	TLE Family Member 3, Transcriptional Corepressor	Protein Coding	44	GC15M070047	2.054105
SLC32A1	Solute Carrier Family 32 Member 1	Protein Coding	44	GC20P038724	2.051759
PADI4	Peptidyl Arginine Deiminase 4	Protein Coding	44	GC01P017308	2.045783
IFNGR2	Interferon Gamma Receptor 2	Protein Coding	44	GC21P033402	2.043825
GNGT1	G Protein Subunit Gamma Transducin 1	Protein Coding	44	GC07P093591	2.042977
FZD8	Frizzled Class Receptor 8	Protein Coding	44	GC10M035638	2.041193
CRB1	Crumbs Cell Polarity Complex Component 1	Protein Coding	44	GC01P197170	2.039725
FZD3	Frizzled Class Receptor 3	Protein Coding	44	GC08P028494	2.026656
RBBP4	RB Binding Protein 4, Chromatin Remodeling Factor	Protein Coding	44	GC01P032651	2.023812
CBX3	Chromobox 3	Protein Coding	44	GC07P026201	2.020833
ACSS2	Acyl-CoA Synthetase Short Chain Family Member 2	Protein Coding	44	GC20P034873	2.015197
ALPI	Alkaline Phosphatase, Intestinal	Protein Coding	44	GC02P232456	2.013223
RANBP1	RAN Binding Protein 1	Protein Coding	44	GC22P020115	2.010827
ENPP3	Ectonucleotide Pyrophosphatase/Phosphodiesterase 3	Protein Coding	44	GC06P131617	1.99561
POLL	DNA Polymerase Lambda	Protein Coding	44	GC10M101578	1.987903
HNF4G	Hepatocyte Nuclear Factor 4 Gamma	Protein Coding	44	GC08P075407	1.987069
SUV39H1	Suppressor Of Variegation 3-9 Homolog 1	Protein Coding	44	GC0XP048699	1.976894
SLC1A5	Solute Carrier Family 1 Member 5	Protein Coding	44	GC19M047159	1.96537
LAP3	Leucine Aminopeptidase 3	Protein Coding	44	GC04P017578	1.938678
DMGDH	Dimethylglycine Dehydrogenase	Protein Coding	44	GC05M078997	1.921262
SLK	STE20 Like Kinase	Protein Coding	44	GC10P103967	1.907776
HOXB1	Homeobox B1	Protein Coding	44	GC17M048528	1.903778
USP2	Ubiquitin Specific Peptidase 2	Protein Coding	44	GC11M119355	1.9017
SPINT2	Serine Peptidase Inhibitor, Kunitz Type 2	Protein Coding	44	GC19P038244	1.895489
NCK2	NCK Adaptor Protein 2	Protein Coding	44	GC02P105744	1.892683
RAB3A	RAB3A, Member RAS Oncogene Family	Protein Coding	44	GC19M018196	1.886786
SKIL	SKI Like Proto-Oncogene	Protein Coding	44	GC03P170357	1.880324
ARHGAP1	Rho GTPase Activating Protein 1	Protein Coding	44	GC11M068978	1.874851
CCS	Copper Chaperone For Superoxide Dismutase	Protein Coding	44	GC11P066593	1.866088
SLC7A6	Solute Carrier Family 7 Member 6	Protein Coding	44	GC16P068263	1.856323
ARHGEF10	Rho Guanine Nucleotide Exchange Factor 10	Protein Coding	44	GC08P001823	1.855775
TIA1	TIA1 Cytotoxic Granule Associated RNA Binding Protein	Protein Coding	44	GC02M070209	1.855724
ASIC1	Acid Sensing Ion Channel Subunit 1	Protein Coding	44	GC12P050057	1.842093
STK38	Serine/Threonine Kinase 38	Protein Coding	44	GC06M036493	1.831201
SKP1	S-Phase Kinase Associated Protein 1	Protein Coding	44	GC05M134148	1.821543
SFPQ	Splicing Factor Proline And Glutamine Rich	Protein Coding	44	GC01M035176	1.809636
NME3	NME/NM23 Nucleoside Diphosphate Kinase 3	Protein Coding	44	GC16M001770	1.807217
ADAMTS18	ADAM Metallopeptidase With Thrombospondin Type 1 Motif 18	Protein Coding	44	GC16M077316	1.794442
LGR5	Leucine Rich Repeat Containing G Protein-Coupled Receptor 5	Protein Coding	44	GC12P071439	1.787274
PTTG1	PTTG1 Regulator Of Sister Chromatid Separation, Securin	Protein Coding	44	GC05P160422	1.784419
NPY2R	Neuropeptide Y Receptor Y2	Protein Coding	44	GC04P155173	1.778772

SLC4A2	Solute Carrier Family 4 Member 2	Protein Coding	44	GC07P151057	1.773142
C8B	Complement C8 Beta Chain	Protein Coding	44	GC01M056929	1.768106
GSTO2	Glutathione S-Transferase Omega 2	Protein Coding	44	GC10P104268	1.761789
SERPINB6	Serpin Family B Member 6	Protein Coding	44	GC06M002948	1.759146
UPB1	Beta-Ureidopropionase 1	Protein Coding	44	GC22P024494	1.758674
CORO1A	Coronin 1A	Protein Coding	44	GC16P032415	1.756106
P2RY4	Pyrimidinergic Receptor P2Y4	Protein Coding	44	GC0XM070258	1.74961
PRMT5	Protein Arginine Methyltransferase 5	Protein Coding	44	GC14M022920	1.743156
PSMA5	Proteasome 20S Subunit Alpha 5	Protein Coding	44	GC01M109399	1.724138
GSTO1	Glutathione S-Transferase Omega 1	Protein Coding	44	GC10P104235	1.718866
PCSK7	Proprotein Convertase Subtilisin/Kexin Type 7	Protein Coding	44	GC11M117199	1.718082
RSPO1	R-Spondin 1	Protein Coding	44	GC01M037612	1.712054
KRT13	Keratin 13	Protein Coding	44	GC17M041500	1.710185
HSPA1L	Heat Shock Protein Family A (Hsp70) Member 1 Like	Protein Coding	44	GC06M031809	1.709721
DGKG	Diacylglycerol Kinase Gamma	Protein Coding	44	GC03M186105	1.684653
FSCN1	Fascin Actin-Bundling Protein 1	Protein Coding	44	GC07P005592	1.677001
TIRAP	TIR Domain Containing Adaptor Protein	Protein Coding	44	GC11P126284	1.667153
TANK	TRAF Family Member Associated NFKB Activator	Protein Coding	44	GC02P161136	1.648145
UGP2	UDP-Glucose Pyrophosphorylase 2	Protein Coding	44	GC02P063840	1.647588
C9	Complement C9	Protein Coding	44	GC05M039320	1.643049
PSMD9	Proteasome 26S Subunit, Non-ATPase 9	Protein Coding	44	GC12P123591	1.642468
FTCD	Formimidoyltransferase Cyclodeaminase	Protein Coding	44	GC21M048321	1.630962
HTR6	5-Hydroxytryptamine Receptor 6	Protein Coding	44	GC01P019666	1.629275
MAP1LC3A	Microtubule Associated Protein 1 Light Chain 3 Alpha	Protein Coding	44	GC20P034546	1.626067
CAPN5	Calpain 5	Protein Coding	44	GC11P077066	1.625519
CYP4B1	Cytochrome P450 Family 4 Subfamily B Member 1	Protein Coding	44	GC01P046757	1.614385
ALDH3A1	Aldehyde Dehydrogenase 3 Family Member A1	Protein Coding	44	GC17M019737	1.595713
PAFAH1B2	Platelet Activating Factor Acetylhydrolase 1b Catalytic Subunit 2	Protein Coding	44	GC11P117144	1.582837
NUAK1	NUAK Family Kinase 1	Protein Coding	44	GC12M106063	1.577571
FBP2	Fructose-Bisphosphatase 2	Protein Coding	44	GC09M094558	1.573709
SERPINA5	Serpin Family A Member 5	Protein Coding	44	GC14P094563	1.569647
BAIAP2	BAR/IMD Domain Containing Adaptor Protein 2	Protein Coding	44	GC17P081035	1.560277
CRX	Cone-Rod Homeobox	Protein Coding	44	GC19P047819	1.557257
SLC2A5	Solute Carrier Family 2 Member 5	Protein Coding	44	GC01M009036	1.555298
PPP6C	Protein Phosphatase 6 Catalytic Subunit	Protein Coding	44	GC09M125147	1.550936
UCHL3	Ubiquitin C-Terminal Hydrolase L3	Protein Coding	44	GC13P075550	1.544728
HDAC10	Histone Deacetylase 10	Protein Coding	44	GC22M050245	1.537007
SLC25A6	Solute Carrier Family 25 Member 6	Protein Coding	44	GC0XM001386	1.519361
HSPH1	Heat Shock Protein Family H (Hsp110) Member 1	Protein Coding	44	GC13M031134	1.515501
CRABP2	Cellular Retinoic Acid Binding Protein 2	Protein Coding	44	GC01M156701	1.512628
SPI1	Spi-1 Proto-Oncogene	Protein Coding	44	GC11M068998	1.511541
VPS35	VPS35 Retromer Complex Component	Protein Coding	44	GC16M046766	1.504168
PSMD2	Proteasome 26S Subunit Ubiquitin Receptor, Non-ATPase 2	Protein Coding	44	GC03P184298	1.501974
TACR2	Tachykinin Receptor 2	Protein Coding	44	GC10M069403	1.499377
TECR	Trans-2,3-Enoyl-CoA Reductase	Protein Coding	44	GC19P014504	1.499284
FCER1G	Fc Fragment Of IgE Receptor Ig	Protein Coding	44	GC01P161215	1.496095
IDI1	Isopentenyl-Diphosphate Delta Isomerase 1	Protein Coding	44	GC10M001039	1.492798
NUDT1	Nudix Hydrolase 1	Protein Coding	44	GC07P002242	1.492528
GTF2H1	General Transcription Factor IIH Subunit 1	Protein Coding	44	GC11P018323	1.492213
ABCA7	ATP Binding Cassette Subfamily A Member 7	Protein Coding	44	GC19P001040	1.490729
CNDP2	Carnosine Dipeptidase 2	Protein Coding	44	GC18P074495	1.481469
HCRTR1	Hypocretin Receptor 1	Protein Coding	44	GC01P031587	1.479345
ARHGEF12	Rho Guanine Nucleotide Exchange Factor 12	Protein Coding	44	GC11P120336	1.470166

STK10	Serine/Threonine Kinase 10	Protein Coding	44	GC05M172042	1.463572
XRN2	5'-3' Exoribonuclease 2	Protein Coding	44	GC20P021303	1.463295
TAPBP	TAP Binding Protein	Protein Coding	44	GC06M033299	1.459895
GGH	Gamma-Glutamyl Hydrolase	Protein Coding	44	GC08M063015	1.444557
UBE2G1	Ubiquitin Conjugating Enzyme E2 G1	Protein Coding	44	GC17M004270	1.441293
STMN1	Stathmin 1	Protein Coding	44	GC01M025884	1.423545
PEA15	Proliferation And Apoptosis Adaptor Protein 15	Protein Coding	44	GC01P160205	1.422858
PIGK	Phosphatidylinositol Glycan Anchor Biosynthesis Class K	Protein Coding	44	GC01M077088	1.421317
IL1RAP	Interleukin 1 Receptor Accessory Protein	Protein Coding	44	GC03P190514	1.421204
MSRA	Methionine Sulfoxide Reductase A	Protein Coding	44	GC08P010054	1.417079
CDX2	Caudal Type Homeobox 2	Protein Coding	44	GC13M027962	1.407699
DYRK2	Dual Specificity Tyrosine Phosphorylation Regulated Kinase 2	Protein Coding	44	GC12P067558	1.406083
EIF5A	Eukaryotic Translation Initiation Factor 5A	Protein Coding	44	GC17P007306	1.398696
PREX1	Phosphatidylinositol-3,4,5-Trisphosphate Dependent Rac Exchange Factor 1	Protein Coding	44	GC20M048624	1.396102
TRADD	TNFRSF1A Associated Via Death Domain	Protein Coding	44	GC16M067154	1.38723
CLIP1	CAP-Gly Domain Containing Linker Protein 1	Protein Coding	44	GC12M122271	1.385378
PHKA1	Phosphorylase Kinase Regulatory Subunit Alpha 1	Protein Coding	44	GC0XM072578	1.382327
SV2A	Synaptic Vesicle Glycoprotein 2A	Protein Coding	44	GC01M149903	1.380257
NUMA1	Nuclear Mitotic Apparatus Protein 1	Protein Coding	44	GC11M072002	1.37436
PPID	Peptidylprolyl Isomerase D	Protein Coding	44	GC04M158709	1.358914
CD2	CD2 Molecule	Protein Coding	44	GC01P116754	1.358177
CD226	CD226 Molecule	Protein Coding	44	GC18M069831	1.356326
GNG2	G Protein Subunit Gamma 2	Protein Coding	44	GC14P051826	1.355857
MORF4L1	Mortality Factor 4 Like 1	Protein Coding	44	GC15P078810	1.347094
RGS10	Regulator Of G Protein Signaling 10	Protein Coding	44	GC10M119499	1.345573
GFRA2	GDNF Family Receptor Alpha 2	Protein Coding	44	GC08M021690	1.344301
PLK2	Polo Like Kinase 2	Protein Coding	44	GC05M058453	1.332697
PSMD14	Proteasome 26S Subunit, Non-ATPase 14	Protein Coding	44	GC02P161308	1.326787
CSNK1G1	Casein Kinase 1 Gamma 1	Protein Coding	44	GC15M064165	1.320171
KIF2C	Kinesin Family Member 2C	Protein Coding	44	GC01P044739	1.317229
RNASET2	Ribonuclease T2	Protein Coding	44	GC06M166924	1.315539
AHCYL1	Adenosylhomocysteinase Like 1	Protein Coding	44	GC01P109984	1.315235
LRPAP1	LDL Receptor Related Protein Associated Protein 1	Protein Coding	44	GC04M003508	1.314512
MCFD2	Multiple Coagulation Factor Deficiency 2, ER Cargo Receptor Complex Subunit	Protein Coding	44	GC02M046901	1.309368
CD74	CD74 Molecule	Protein Coding	44	GC05M150378	1.305893
P2RX2	Purinergic Receptor P2X 2	Protein Coding	44	GC12P132618	1.30045
VNN1	Vanin 1	Protein Coding	44	GC06M132680	1.296297
HAO1	Hydroxyacid Oxidase 1	Protein Coding	44	GC20M007863	1.292701
TUB	TUB Bipartite Transcription Factor	Protein Coding	44	GC11P008040	1.290223
ROM1	Retinal Outer Segment Membrane Protein 1	Protein Coding	44	GC11P062611	1.288505
SUV39H2	Suppressor Of Variegation 3-9 Homolog 2	Protein Coding	44	GC10P014878	1.288213
PTPRZ1	Protein Tyrosine Phosphatase Receptor Type Z1	Protein Coding	44	GC07P121873	1.287112
MMP20	Matrix Metallopeptidase 20	Protein Coding	44	GC11M102576	1.282523
NTF4	Neurotrophin 4	Protein Coding	44	GC19M049098	1.269864
TRIM33	Tripartite Motif Containing 33	Protein Coding	44	GC01M114392	1.269451
SLC8A3	Solute Carrier Family 8 Member A3	Protein Coding	44	GC14M070044	1.266249
SSTR1	Somatostatin Receptor 1	Protein Coding	44	GC14P038207	1.265752
ELK1	ETS Transcription Factor ELK1	Protein Coding	44	GC0XM047635	1.261949
AMFR	Autocrine Motility Factor Receptor	Protein Coding	44	GC16M056361	1.25754
RPS6KA6	Ribosomal Protein S6 Kinase A6	Protein Coding	44	GC0XM084058	1.249264
HR	HR Lysine Demethylase And Nuclear Receptor Corepressor	Protein Coding	44	GC08M022114	1.24437
PRSS3	Serine Protease 3	Protein Coding	44	GC09P033750	1.241417
NFE2L1	Nuclear Factor, Erythroid 2 Like 1	Protein Coding	44	GC17P048049	1.240028

CHRNB4	Cholinergic Receptor Nicotinic Beta 4 Subunit	Protein Coding	44	GC15M078624	1.236807
PANX1	Pannexin 1	Protein Coding	44	GC11P094128	1.228101
CDC14A	Cell Division Cycle 14A	Protein Coding	44	GC01P100351	1.214037
BRS3	Bombesin Receptor Subtype 3	Protein Coding	44	GC0XP136482	1.192441
ACTR2	Actin Related Protein 2	Protein Coding	44	GC02P065227	1.186992
TRAF5	TNF Receptor Associated Factor 5	Protein Coding	44	GC01P211326	1.185135
SNW1	SNW Domain Containing 1	Protein Coding	44	GC14M077717	1.174347
CNTFR	Ciliary Neurotrophic Factor Receptor	Protein Coding	44	GC09M034551	1.170924
TACSTD2	Tumor Associated Calcium Signal Transducer 2	Protein Coding	44	GC01M058575	1.163791
ITM2B	Integral Membrane Protein 2B	Protein Coding	44	GC13P048233	1.163048
ST6GAL1	ST6 Beta-Galactoside Alpha-2,6-Sialyltransferase 1	Protein Coding	44	GC03P186930	1.156532
ARHGDIB	Rho GDP Dissociation Inhibitor Beta	Protein Coding	44	GC12M014942	1.152001
SERPINB5	Serpin Family B Member 5	Protein Coding	44	GC18P063476	1.138964
RNGTT	RNA Guanylyltransferase And 5'-Phosphatase	Protein Coding	44	GC06M088609	1.13797
NAGK	N-Acetylglucosamine Kinase	Protein Coding	44	GC02P071064	1.136788
TACC3	Transforming Acidic Coiled-Coil Containing Protein 3	Protein Coding	44	GC04P001723	1.132785
DCPS	Decapping Enzyme, Scavenger	Protein Coding	44	GC11P126303	1.103542
PICALM	Phosphatidylinositol Binding Clathrin Assembly Protein	Protein Coding	44	GC11M085957	1.101262
SLC14A1	Solute Carrier Family 14 Member 1 (Kidd Blood Group)	Protein Coding	44	GC18P045687	1.100302
KRT4	Keratin 4	Protein Coding	44	GC12M052806	1.094435
POU2F2	POU Class 2 Homeobox 2	Protein Coding	44	GC19M042086	1.093992
UGT1A9	UDP Glucuronosyltransferase Family 1 Member A9	Protein Coding	44	GC02P233671	1.09164
TNFRSF14	TNF Receptor Superfamily Member 14	Protein Coding	44	GC01P002555	1.080802
ATP6V0D1	ATPase H+ Transporting V0 Subunit D1	Protein Coding	44	GC16M067438	1.06505
PTPRT	Protein Tyrosine Phosphatase Receptor Type T	Protein Coding	44	GC20M042072	1.059236
RGS14	Regulator Of G Protein Signaling 14	Protein Coding	44	GC05P177357	1.053102
ARHGAP5	Rho GTPase Activating Protein 5	Protein Coding	44	GC14P032076	1.048046
MYO9B	Myosin IXB	Protein Coding	44	GC19P026629	1.048004
TFE3	Transcription Factor Binding To IGHM Enhancer 3	Protein Coding	44	GC0XM049028	1.035114
KCNC3	Potassium Voltage-Gated Channel Subfamily C Member 3	Protein Coding	44	GC19M050322	1.033986
SNCB	Synuclein Beta	Protein Coding	44	GC05M176620	1.029049
KIF22	Kinesin Family Member 22	Protein Coding	44	GC16P032374	1.01926
CSPG4	Chondroitin Sulfate Proteoglycan 4	Protein Coding	44	GC15M075674	1.004914
PBK	PDZ Binding Kinase	Protein Coding	44	GC08M027809	1.000199
GDA	Guanine Deaminase	Protein Coding	44	GC09P072114	0.997424
TYROBP	Transmembrane Immune Signaling Adaptor TYROBP	Protein Coding	44	GC19M035904	0.985197
SQLE	Squalene Epoxidase	Protein Coding	44	GC08P124998	0.984903
CHST6	Carbohydrate Sulfotransferase 6	Protein Coding	44	GC16M075472	0.984241
TRIB3	Tribbles Pseudokinase 3	Protein Coding	44	GC20P000361	0.95504
HSD17B1	Hydroxysteroid 17-Beta Dehydrogenase 1	Protein Coding	44	GC17P042548	0.952959
RPIA	Ribose 5-Phosphate Isomerase A	Protein Coding	44	GC02P088691	0.952299
SLC6A12	Solute Carrier Family 6 Member 12	Protein Coding	44	GC12M000169	0.948942
PDE6C	Phosphodiesterase 6C	Protein Coding	44	GC10P093612	0.945328
VAV2	Vav Guanine Nucleotide Exchange Factor 2	Protein Coding	44	GC09M133761	0.937705
CCR8	C-C Motif Chemokine Receptor 8	Protein Coding	44	GC03P039330	0.937228
ETV4	ETS Variant Transcription Factor 4	Protein Coding	44	GC17M043527	0.935872
PGAM1	Phosphoglycerate Mutase 1	Protein Coding	44	GC10P097426	0.929907
KSR1	Kinase Suppressor Of Ras 1	Protein Coding	44	GC17P027456	0.923882
PLA2G4C	Phospholipase A2 Group IVC	Protein Coding	44	GC19M048047	0.904758
VDAC3	Voltage Dependent Anion Channel 3	Protein Coding	44	GC08P042392	0.902061
SH3GL1	SH3 Domain Containing GRB2 Like 1, Endophilin A2	Protein Coding	44	GC19M004360	0.901154
MMP24	Matrix Metallopeptidase 24	Protein Coding	44	GC20P035226	0.891506
CYP26C1	Cytochrome P450 Family 26 Subfamily C Member 1	Protein Coding	44	GC10P093060	0.884183

BLMH	Bleomycin Hydrolase	Protein Coding	44	GC17M030248	0.881892
ARHGEF7	Rho Guanine Nucleotide Exchange Factor 7	Protein Coding	44	GC13P111114	0.880206
TRPM8	Transient Receptor Potential Cation Channel Subfamily M Member 8	Protein Coding	44	GC02P233917	0.87745
ING1	Inhibitor Of Growth Family Member 1	Protein Coding	44	GC13P110712	0.849909
PLK3	Polo Like Kinase 3	Protein Coding	44	GC01P044799	0.844835
HSPA2	Heat Shock Protein Family A (Hsp70) Member 2	Protein Coding	44	GC14P064535	0.839069
HSD17B7	Hydroxysteroid 17-Beta Dehydrogenase 7	Protein Coding	44	GC01P162790	0.833201
PSMD7	Proteasome 26S Subunit, Non-ATPase 7	Protein Coding	44	GC16P074296	0.831498
KLK2	Kallikrein Related Peptidase 2	Protein Coding	44	GC19P050861	0.829916
ARPC3	Actin Related Protein 2/3 Complex Subunit 3	Protein Coding	44	GC12M110434	0.825649
DRG1	Developmentally Regulated GTP Binding Protein 1	Protein Coding	44	GC22P031399	0.813504
MLPH	Melanophilin	Protein Coding	44	GC02P237485	0.80213
SLC36A2	Solute Carrier Family 36 Member 2	Protein Coding	44	GC05M151309	0.799005
F11R	F11 Receptor	Protein Coding	44	GC01M160995	0.78935
FERMT3	FERM Domain Containing Kindlin 3	Protein Coding	44	GC11P064206	0.789228
PTGES3	Prostaglandin E Synthase 3	Protein Coding	44	GC12M056667	0.766478
VDAC2	Voltage Dependent Anion Channel 2	Protein Coding	44	GC10P075210	0.760984
MKNK2	MAPK Interacting Serine/Threonine Kinase 2	Protein Coding	44	GC19M002037	0.740964
ITGB7	Integrin Subunit Beta 7	Protein Coding	44	GC12M053191	0.737507
HPN	Hepsin	Protein Coding	44	GC19P035040	0.732612
PDE1B	Phosphodiesterase 1B	Protein Coding	44	GC12P054549	0.722828
GIT1	GIT ArfGAP 1	Protein Coding	44	GC17M029573	0.715754
PRDX4	Peroxiredoxin 4	Protein Coding	44	GC0XP023665	0.71478
GSTA4	Glutathione S-Transferase Alpha 4	Protein Coding	44	GC06M052977	0.709829
PSMA2	Proteasome 20S Subunit Alpha 2	Protein Coding	44	GC07M042916	0.684587
CMPK1	Cytidine/Uridine Monophosphate Kinase 1	Protein Coding	44	GC01P047333	0.654328
CDA	Cytidine Deaminase	Protein Coding	44	GC01P020588	0.63146
FUT3	Fucosyltransferase 3 (Lewis Blood Group)	Protein Coding	44	GC19M005843	0.630323
DNAJB2	DnaJ Heat Shock Protein Family (Hsp40) Member B2	Protein Coding	44	GC02P219279	0.595117
POLR2E	RNA Polymerase II, I And III Subunit E	Protein Coding	44	GC19M001086	0.581428
CYP4F3	Cytochrome P450 Family 4 Subfamily F Member 3	Protein Coding	44	GC19P015640	0.549357
PPAT	Phosphoribosyl Pyrophosphate Amidotransferase	Protein Coding	44	GC04M056393	0.543983
CYP2S1	Cytochrome P450 Family 2 Subfamily S Member 1	Protein Coding	44	GC19P041193	0.537362
RPN1	Ribophorin I	Protein Coding	44	GC03M128619	0.535527
SETD7	SET Domain Containing 7, Histone Lysine Methyltransferase	Protein Coding	44	GC04M139495	0.535527
ADCY2	Adenylate Cyclase 2	Protein Coding	44	GC05P007396	0.506227
CTRC	Chymotrypsin C	Protein Coding	44	GC01P015438	0.484603
AKR1B10	Aldo-Keto Reductase Family 1 Member B10	Protein Coding	44	GC07P134527	0.401535
CYP3A7	Cytochrome P450 Family 3 Subfamily A Member 7	Protein Coding	44	GC07M099705	0.382196
TRAF4	TNF Receptor Associated Factor 4	Protein Coding	44	GC17P029346	0.364537
NET1	Neuroepithelial Cell Transforming 1	Protein Coding	44	GC10P005444	0.359139
TBX20	T-Box Transcription Factor 20	Protein Coding	43	GC07M035237	48.11577
NODAL	Nodal Growth Differentiation Factor	Protein Coding	43	GC10M070431	44.59635
ACTC1	Actin Alpha Cardiac Muscle 1	Protein Coding	43	GC15M034788	39.27125
HAND1	Heart And Neural Crest Derivatives Expressed 1	Protein Coding	43	GC05M154475	36.35664
ZFPM2	Zinc Finger Protein, FOG Family Member 2	Protein Coding	43	GC08P104590	30.52378
ANKRD1	Ankyrin Repeat Domain 1	Protein Coding	43	GC10M090912	27.63391
CSRP3	Cysteine And Glycine Rich Protein 3	Protein Coding	43	GC11M019160	26.79244
STAG3	Stromal Antigen 3	Protein Coding	43	GC07P100177	25.62607
DTNA	Dystrobrevin Alpha	Protein Coding	43	GC18P034493	25.50667
FANCM	FA Complementation Group M	Protein Coding	43	GC14P045135	25.4917
TCAP	Titin-Cap	Protein Coding	43	GC17P039664	23.37869
SARS2	Seryl-TRNA Synthetase 2, Mitochondrial	Protein Coding	43	GC19M046734	20.99208

GPT	Glutamic--Pyruvic Transaminase	Protein Coding	43	GC08P144502	20.62925
BRDT	Bromodomain Testis Associated	Protein Coding	43	GC01P091949	20.15336
XRCC2	X-Ray Repair Cross Complementing 2	Protein Coding	43	GC07M152644	19.18498
FOXF1	Forkhead Box F1	Protein Coding	43	GC16P086510	18.82795
NAGLU	N-Acetyl-Alpha-Glucosaminidase	Protein Coding	43	GC17P042537	18.50356
AARS2	Alanyl-TRNA Synthetase 2, Mitochondrial	Protein Coding	43	GC06M044297	17.7887
RPS26	Ribosomal Protein S26	Protein Coding	43	GC12P056043	17.58853
CLCN6	Chloride Voltage-Gated Channel 6	Protein Coding	43	GC01P011806	17.52562
SBDS	SBDS Ribosome Maturation Factor	Protein Coding	43	GC07M066987	17.07701
LTA	Lymphotoxin Alpha	Protein Coding	43	GC06P055200	16.39583
FOXH1	Forkhead Box H1	Protein Coding	43	GC08M144473	16.28459
FOXC1	Forkhead Box C1	Protein Coding	43	GC06P001610	16.12465
MIPEP	Mitochondrial Intermediate Peptidase	Protein Coding	43	GC13M023730	15.56963
LZTR1	Leucine Zipper Like Transcription Regulator 1	Protein Coding	43	GC22P020996	15.45158
CALCA	Calcitonin Related Polypeptide Alpha	Protein Coding	43	GC11M014945	15.31234
PEX6	Peroxisomal Biogenesis Factor 6	Protein Coding	43	GC06M042963	15.0729
PLOD1	Procollagen-Lysine,2-Oxoglutarate 5-Dioxygenase 1	Protein Coding	43	GC01P011934	14.91063
HLA-DQB1	Major Histocompatibility Complex, Class II, DQ Beta 1	Protein Coding	43	GC06M046939	14.67871
MGP	Matrix Gla Protein	Protein Coding	43	GC12M015756	14.60804
GDF15	Growth Differentiation Factor 15	Protein Coding	43	GC19P026656	14.58843
HIRA	Histone Cell Cycle Regulator	Protein Coding	43	GC22M019318	14.54476
FANCI	FA Complementation Group I	Protein Coding	43	GC15P089243	14.47008
BCOR	BCL6 Corepressor	Protein Coding	43	GC0XM040049	14.42255
ZMPSTE24	Zinc Metallopeptidase STE24	Protein Coding	43	GC01P040258	14.13836
TRPM3	Transient Receptor Potential Cation Channel Subfamily M Member 3	Protein Coding	43	GC09M070529	13.76112
KAT6B	Lysine Acetyltransferase 6B	Protein Coding	43	GC10P074840	13.54854
DIAPH2	Diaphanous Related Formin 2	Protein Coding	43	GC0XP096684	13.53939
SYCP3	Synaptonemal Complex Protein 3	Protein Coding	43	GC12M101728	13.51195
SEMA3E	Semaphorin 3E	Protein Coding	43	GC07M083363	13.47676
PEX5	Peroxisomal Biogenesis Factor 5	Protein Coding	43	GC12P011881	13.47474
MTRR	5-Methyltetrahydrofolate-Homocysteine Methyltransferase Reductase	Protein Coding	43	GC05P007851	13.44784
CHST3	Carbohydrate Sulfotransferase 3	Protein Coding	43	GC10P071964	12.94561
UMOD	Uromodulin	Protein Coding	43	GC16M020344	12.78173
PALB2	Partner And Localizer Of BRCA2	Protein Coding	43	GC16M023603	12.77865
GTF2IRD1	GTF2I Repeat Domain Containing 1	Protein Coding	43	GC07P074468	12.7093
CALM3	Calmodulin 3	Protein Coding	43	GC19P046601	12.68946
BSCL2	BSCL2 Lipid Droplet Biogenesis Associated, Seipin	Protein Coding	43	GC11M069260	12.68747
OFD1	OFD1 Centriole And Centriolar Satellite Protein	Protein Coding	43	GC0XP013734	12.63294
SST	Somatostatin	Protein Coding	43	GC03M187668	12.58593
AGK	Acylglycerol Kinase	Protein Coding	43	GC07P141551	12.58472
HBA1	Hemoglobin Subunit Alpha 1	Protein Coding	43	GC16P005490	12.47423
FANCF	FA Complementation Group F	Protein Coding	43	GC11M022600	12.34473
IL17A	Interleukin 17A	Protein Coding	43	GC06P052186	12.22016
SEMA3C	Semaphorin 3C	Protein Coding	43	GC07M080742	12.17418
IDUA	Alpha-L-Iduronidase	Protein Coding	43	GC04P000986	12.15953
MLXIPL	MLX Interacting Protein Like	Protein Coding	43	GC07M073593	12.11409
FANCE	FA Complementation Group E	Protein Coding	43	GC06P055327	11.90715
HBG2	Hemoglobin Subunit Gamma 2	Protein Coding	43	GC11M005442	11.73469
PLEC	Plectin	Protein Coding	43	GC08M143917	11.61116
COX15	Cytochrome C Oxidase Assembly Homolog COX15	Protein Coding	43	GC10M099696	11.56967
GP1BB	Glycoprotein Ib Platelet Subunit Beta	Protein Coding	43	GC22P020083	11.50209
CHRND	Cholinergic Receptor Nicotinic Delta Subunit	Protein Coding	43	GC02P232525	11.48222
ASXL1	ASXL Transcriptional Regulator 1	Protein Coding	43	GC20P032364	11.44833

MID1	Midline 1	Protein Coding	43	GC0XM010445	11.37922
GTF2I	General Transcription Factor IIi	Protein Coding	43	GC07P074658	11.27421
ERCC8	ERCC Excision Repair 8, CSA Ubiquitin Ligase Complex Subunit	Protein Coding	43	GC05M060874	11.22623
SIX3	SIX Homeobox 3	Protein Coding	43	GC02P044941	11.18836
GAS1	Growth Arrest Specific 1	Protein Coding	43	GC09M086944	11.15794
KCNAB2	Potassium Voltage-Gated Channel Subfamily A Regulatory Beta Subunit 2	Protein Coding	43	GC01P006020	11.12133
PTX3	Pentraxin 3	Protein Coding	43	GC03P157436	10.96313
HLA-DQA1	Major Histocompatibility Complex, Class II, DQ Alpha 1	Protein Coding	43	GC06P055231	10.85544
HBA2	Hemoglobin Subunit Alpha 2	Protein Coding	43	GC16P005491	10.78942
NFIX	Nuclear Factor I X	Protein Coding	43	GC19P012996	10.76156
SELL	Selectin L	Protein Coding	43	GC01M169690	10.7508
HSPA4	Heat Shock Protein Family A (Hsp70) Member 4	Protein Coding	43	GC05P133051	10.7481
CHGA	Chromogranin A	Protein Coding	43	GC14P092925	10.66649
NAA10	N-Alpha-Acetyltransferase 10, NatA Catalytic Subunit	Protein Coding	43	GC0XM153929	10.59051
ELAC2	ElaC Ribonuclease Z 2	Protein Coding	43	GC17M012991	10.52832
SCYL1	SCY1 Like Pseudokinase 1	Protein Coding	43	GC11P065525	9.903081
PEX3	Peroxisomal Biogenesis Factor 3	Protein Coding	43	GC06P143450	9.894615
LIPT1	Lipoyltransferase 1	Protein Coding	43	GC02P099169	9.818179
APOL1	Apolipoprotein L1	Protein Coding	43	GC22P036253	9.753121
PEX10	Peroxisomal Biogenesis Factor 10	Protein Coding	43	GC01M002403	9.749228
DARS2	Aspartyl-TRNA Synthetase 2, Mitochondrial	Protein Coding	43	GC01P173824	9.732334
THPO	Thrombopoietin	Protein Coding	43	GC03M184371	9.700348
BGN	Biglycan	Protein Coding	43	GC0XP153494	9.662901
RPL26	Ribosomal Protein L26	Protein Coding	43	GC17M008377	9.63543
COX6B1	Cytochrome C Oxidase Subunit 6B1	Protein Coding	43	GC19P040358	9.469806
RAPSN	Receptor Associated Protein Of The Synapse	Protein Coding	43	GC11M069002	9.46434
MATR3	Matrin 3	Protein Coding	43	GC05P139274	9.445094
TCOF1	Treacle Ribosome Biogenesis Factor 1	Protein Coding	43	GC05P150358	9.435309
XYLT1	Xylosyltransferase 1	Protein Coding	43	GC16M017101	9.246506
GJC1	Gap Junction Protein Gamma 1	Protein Coding	43	GC17M044800	9.238067
NFU1	NFU1 Iron-Sulfur Cluster Scaffold	Protein Coding	43	GC02M069395	9.233553
PEX19	Peroxisomal Biogenesis Factor 19	Protein Coding	43	GC01M160276	9.226178
ABCG8	ATP Binding Cassette Subfamily G Member 8	Protein Coding	43	GC02P043828	9.178281
ANK1	Ankyrin 1	Protein Coding	43	GC08M041653	9.173423
B4GALT7	Beta-1,4-Galactosyltransferase 7	Protein Coding	43	GC05P177600	9.151137
EFTUD2	Elongation Factor Tu GTP Binding Domain Containing 2	Protein Coding	43	GC17M044852	9.116962
PPIG	Peptidylprolyl Isomerase G	Protein Coding	43	GC02P169584	9.09871
GNRH1	Gonadotropin Releasing Hormone 1	Protein Coding	43	GC08M025419	9.056637
GCLM	Glutamate-Cysteine Ligase Modifier Subunit	Protein Coding	43	GC01M093885	9.042592
MYL1	Myosin Light Chain 1	Protein Coding	43	GC02M210290	8.998009
GDF2	Growth Differentiation Factor 2	Protein Coding	43	GC10P047322	8.985116
SDCCAG8	SHH Signaling And Ciliogenesis Regulator SDCCAG8	Protein Coding	43	GC01P243255	8.966749
TREX1	Three Prime Repair Exonuclease 1	Protein Coding	43	GC03P048466	8.932953
FCN3	Ficolin 3	Protein Coding	43	GC01M027380	8.926012
LETM1	Leucine Zipper And EF-Hand Containing Transmembrane Protein 1	Protein Coding	43	GC04M001811	8.886051
NDUFB8	NADH:Ubiquinone Oxidoreductase Subunit B8	Protein Coding	43	GC10M100523	8.881374
SLC27A6	Solute Carrier Family 27 Member 6	Protein Coding	43	GC05P128538	8.847546
VANGL1	VANGL Planar Cell Polarity Protein 1	Protein Coding	43	GC01P115641	8.815559
SNAP29	Synaptosome Associated Protein 29	Protein Coding	43	GC22P020859	8.8027
HYAL2	Hyaluronidase 2	Protein Coding	43	GC03M050317	8.802577
TAF4B	TATA-Box Binding Protein Associated Factor 4b	Protein Coding	43	GC18P026225	8.708097
GRIP1	Glutamate Receptor Interacting Protein 1	Protein Coding	43	GC12M066347	8.680069
NDUFB3	NADH:Ubiquinone Oxidoreductase Subunit B3	Protein Coding	43	GC02P201071	8.677813

SGCG	Sarcoglycan Gamma	Protein Coding	43	GC13P023160	8.667818
DNASE1	Deoxyribonuclease 1	Protein Coding	43	GC16P003611	8.660263
MLYCD	Malonyl-CoA Decarboxylase	Protein Coding	43	GC16P083899	8.581043
GSTM1	Glutathione S-Transferase Mu 1	Protein Coding	43	GC01P109687	8.544142
TRIM21	Tripartite Motif Containing 21	Protein Coding	43	GC11M004384	8.520299
ASPH	Aspartate Beta-Hydroxylase	Protein Coding	43	GC08M061500	8.442205
CDH23	Cadherin Related 23	Protein Coding	43	GC10P071396	8.428151
ATXN2	Ataxin 2	Protein Coding	43	GC12M111443	8.412964
PUF60	Poly(U) Binding Splicing Factor 60	Protein Coding	43	GC08M143816	8.115017
MEIS2	Meis Homeobox 2	Protein Coding	43	GC15M036889	8.042154
RPS17	Ribosomal Protein S17	Protein Coding	43	GC15M082536	8.041869
CLDN16	Claudin 16	Protein Coding	43	GC03P190290	8.036846
TPP1	Tripeptidyl Peptidase 1	Protein Coding	43	GC11M006620	7.926374
SGCA	Sarcoglycan Alpha	Protein Coding	43	GC17P050164	7.881289
NOX4	NADPH Oxidase 4	Protein Coding	43	GC11M089324	7.859065
SLC10A2	Solute Carrier Family 10 Member 2	Protein Coding	43	GC13M103043	7.779664
COL5A2	Collagen Type V Alpha 2 Chain	Protein Coding	43	GC02M189031	7.678233
RECQL4	RecQ Like Helicase 4	Protein Coding	43	GC08M144512	7.672241
HSD3B7	Hydroxy-Delta-5-Steroid Dehydrogenase, 3 Beta- And Steroid Delta-Isomerase 7	Protein Coding	43	GC16P030985	7.667516
FCGR3B	Fc Fragment Of IgG Receptor IIIb	Protein Coding	43	GC01M161623	7.659304
PNPLA8	Patatin Like Phospholipase Domain Containing 8	Protein Coding	43	GC07M108470	7.497484
NDUFAF4	NADH:Ubiquinone Oxidoreductase Complex Assembly Factor 4	Protein Coding	43	GC06M096889	7.472967
ADNP	Activity Dependent Neuroprotector Homeobox	Protein Coding	43	GC20M050888	7.462585
GDF6	Growth Differentiation Factor 6	Protein Coding	43	GC08M096142	7.455109
EPM2A	EPM2A Glucan Phosphatase, Laforin	Protein Coding	43	GC06M145382	7.401968
VAC14	VAC14 Component Of PIKFYVE Complex	Protein Coding	43	GC16M070688	7.391281
RPL31	Ribosomal Protein L31	Protein Coding	43	GC02P100985	7.388346
VRK2	VRK Serine/Threonine Kinase 2	Protein Coding	43	GC02P057907	7.385612
PDHA2	Pyruvate Dehydrogenase E1 Subunit Alpha 2	Protein Coding	43	GC04P095840	7.315903
MYLK3	Myosin Light Chain Kinase 3	Protein Coding	43	GC16M046762	7.312938
ATRIP	ATR Interacting Protein	Protein Coding	43	GC03P048449	7.301533
SMN2	Survival Of Motor Neuron 2, Centromeric	Protein Coding	43	GC05P070049	7.275197
APOA4	Apolipoprotein A4	Protein Coding	43	GC11M116820	7.231635
TG	Thyroglobulin	Protein Coding	43	GC08P132866	7.22609
COX4I2	Cytochrome C Oxidase Subunit 4I2	Protein Coding	43	GC20P031637	7.097304
ARL6	ADP Ribosylation Factor Like GTPase 6	Protein Coding	43	GC03P097764	7.096289
RPS27	Ribosomal Protein S27	Protein Coding	43	GC01P153991	7.058127
MEOX1	Mesenchyme Homeobox 1	Protein Coding	43	GC17M043640	7.055943
SAA1	Serum Amyloid A1	Protein Coding	43	GC11P018267	7.00151
PROCR	Protein C Receptor	Protein Coding	43	GC20P035233	6.991487
SPINK5	Serine Peptidase Inhibitor Kazal Type 5	Protein Coding	43	GC05P148025	6.936431
PLXNA2	Plexin A2	Protein Coding	43	GC01M208023	6.930818
RAG2	Recombination Activating 2	Protein Coding	43	GC11M036575	6.92966
KCNJ12	Potassium Inwardly Rectifying Channel Subfamily J Member 12	Protein Coding	43	GC17P029212	6.889999
LIF	LIF Interleukin 6 Family Cytokine	Protein Coding	43	GC22M030240	6.823872
RPS7	Ribosomal Protein S7	Protein Coding	43	GC02P003575	6.823018
HS6ST1	Heparan Sulfate 6-O-Sulfotransferase 1	Protein Coding	43	GC02M128236	6.804616
TRPC1	Transient Receptor Potential Cation Channel Subfamily C Member 1	Protein Coding	43	GC03P142724	6.801733
ANXA6	Annexin A6	Protein Coding	43	GC05M151100	6.7952
KHDRBS1	KH RNA Binding Domain Containing, Signal Transduction Associated 1	Protein Coding	43	GC01P032013	6.778256
ITGA8	Integrin Subunit Alpha 8	Protein Coding	43	GC10M015513	6.764942
FBLN2	Fibulin 2	Protein Coding	43	GC03P013565	6.760892
SRI	Sorcin	Protein Coding	43	GC07M088205	6.736607

ARX	Aristaless Related Homeobox	Protein Coding	43	GC0XM025003	6.604744
SSB	Small RNA Binding Exonuclease Protection Factor La	Protein Coding	43	GC02P169791	6.598024
FLII	FLII Actin Remodeling Protein	Protein Coding	43	GC17M018244	6.592294
TRIP11	Thyroid Hormone Receptor Interactor 11	Protein Coding	43	GC14M091965	6.511117
CHD3	Chromodomain Helicase DNA Binding Protein 3	Protein Coding	43	GC17P009121	6.507253
NAGS	N-Acetylglutamate Synthase	Protein Coding	43	GC17P044004	6.447983
FABP2	Fatty Acid Binding Protein 2	Protein Coding	43	GC04M119317	6.44683
SMARCD1	SWI/SNF Related, Matrix Associated, Actin Dependent Regulator Of Chromatin, Subfamily D, Member 1	Protein Coding	43	GC12P050085	6.419042
NUP107	Nucleoporin 107	Protein Coding	43	GC12P068686	6.413463
BANF1	BAF Nuclear Assembly Factor 1	Protein Coding	43	GC11P066002	6.400542
RPL27	Ribosomal Protein L27	Protein Coding	43	GC17P042998	6.385046
DDAH2	Dimethylarginine Dimethylaminohydrolase 2	Protein Coding	43	GC06M031727	6.256718
PRICKLE1	Prickle Planar Cell Polarity Protein 1	Protein Coding	43	GC12M042456	6.25062
CD96	CD96 Molecule	Protein Coding	43	GC03P111292	6.226281
LRPPRC	Leucine Rich Pentatricopeptide Repeat Containing	Protein Coding	43	GC02M043850	6.200116
MTO1	Mitochondrial TRNA Translation Optimization 1	Protein Coding	43	GC06P073461	6.165373
CD163	CD163 Molecule	Protein Coding	43	GC12M007471	6.157758
SUMF1	Sulfatase Modifying Factor 1	Protein Coding	43	GC03M003700	6.150311
ATP6AP1	ATPase H+ Transporting Accessory Protein 1	Protein Coding	43	GC0XP154428	6.14975
PARD3	Par-3 Family Cell Polarity Regulator	Protein Coding	43	GC10M034110	6.130447
LTC4S	Leukotriene C4 Synthase	Protein Coding	43	GC05P179793	6.049424
CTNND2	Catenin Delta 2	Protein Coding	43	GC05M010971	6.021803
RPS15A	Ribosomal Protein S15a	Protein Coding	43	GC16M018781	5.986929
NDUFA2	NADH:Ubiquinone Oxidoreductase Subunit A2	Protein Coding	43	GC05M140653	5.981228
COL11A1	Collagen Type XI Alpha 1 Chain	Protein Coding	43	GC01M102876	5.908679
AMBP	Alpha-1-Microglobulin/Bikunin Precursor	Protein Coding	43	GC09M114060	5.887187
PSMD12	Proteasome 26S Subunit, Non-ATPase 12	Protein Coding	43	GC17M067337	5.885044
NSUN2	NOP2/Sun RNA Methyltransferase 2	Protein Coding	43	GC05M006599	5.883298
SEC31A	SEC31 Homolog A, COPII Coat Complex Component	Protein Coding	43	GC04M082818	5.874059
ICOS	Inducible T Cell Costimulator	Protein Coding	43	GC02P203937	5.853437
UTS2R	Urotensin 2 Receptor	Protein Coding	43	GC17P082374	5.846787
SPRED1	Sprouty Related EVH1 Domain Containing 1	Protein Coding	43	GC15P038252	5.824506
ADIPOR2	Adiponectin Receptor 2	Protein Coding	43	GC12P001670	5.812893
SLC39A13	Solute Carrier Family 39 Member 13	Protein Coding	43	GC11P047407	5.804297
PDSS1	Decaprenyl Diphosphate Synthase Subunit 1	Protein Coding	43	GC10P026697	5.75209
CLDN19	Claudin 19	Protein Coding	43	GC01M042742	5.742637
OCA2	OCA2 Melanosomal Transmembrane Protein	Protein Coding	43	GC15M027754	5.713248
ABCA12	ATP Binding Cassette Subfamily A Member 12	Protein Coding	43	GC02M214931	5.677693
NDN	Necdin, MAGE Family Member	Protein Coding	43	GC15M023686	5.661896
HAX1	HCLS1 Associated Protein X-1	Protein Coding	43	GC01P154273	5.649642
SARDH	Sarcosine Dehydrogenase	Protein Coding	43	GC09M133663	5.615244
RARS2	Arginyl-TRNA Synthetase 2, Mitochondrial	Protein Coding	43	GC06M087514	5.590071
MRPL3	Mitochondrial Ribosomal Protein L3	Protein Coding	43	GC03M131462	5.574922
ALG6	ALG6 Alpha-1,3-Glucosyltransferase	Protein Coding	43	GC01P063367	5.50917
CCK	Cholecystokinin	Protein Coding	43	GC03M042274	5.461078
IL21	Interleukin 21	Protein Coding	43	GC04M122612	5.428909
MAPRE2	Microtubule Associated Protein RP/EB Family Member 2	Protein Coding	43	GC18P034976	5.385983
ACSF3	Acyl-CoA Synthetase Family Member 3	Protein Coding	43	GC16P089088	5.383157
PPP1R3A	Protein Phosphatase 1 Regulatory Subunit 3A	Protein Coding	43	GC07M113876	5.37997
RGS2	Regulator Of G Protein Signaling 2	Protein Coding	43	GC01P192809	5.372952
PROX1	Prospero Homeobox 1	Protein Coding	43	GC01P213983	5.370775
NDP	Norrin Cystine Knot Growth Factor NDP	Protein Coding	43	GC0XM043948	5.26811
IL7	Interleukin 7	Protein Coding	43	GC08M078689	5.260712

ARNTL	Aryl Hydrocarbon Receptor Nuclear Translocator Like	Protein Coding	43	GC11P013276	5.252441
TFEB	Transcription Factor EB	Protein Coding	43	GC06M047093	5.224891
KIF1C	Kinesin Family Member 1C	Protein Coding	43	GC17P004998	5.214714
KRIT1	KRIT1 Ankyrin Repeat Containing	Protein Coding	43	GC07M092198	5.212023
CLDN5	Claudin 5	Protein Coding	43	GC22M019523	5.211869
CDKN3	Cyclin Dependent Kinase Inhibitor 3	Protein Coding	43	GC14P054398	5.204963
CHST11	Carbohydrate Sulfotransferase 11	Protein Coding	43	GC12P104455	5.202031
FRZB	Frizzled Related Protein	Protein Coding	43	GC02M182833	5.195598
FGFRL1	Fibroblast Growth Factor Receptor Like 1	Protein Coding	43	GC04P001058	5.194297
RAB1B	RAB1B, Member RAS Oncogene Family	Protein Coding	43	GC11P066722	5.18935
ORC4	Origin Recognition Complex Subunit 4	Protein Coding	43	GC02M147930	5.179927
SSR4	Signal Sequence Receptor Subunit 4	Protein Coding	43	GC0XP153793	5.164808
SLCO2A1	Solute Carrier Organic Anion Transporter Family Member 2A1	Protein Coding	43	GC03M133932	5.153104
PIGO	Phosphatidylinositol Glycan Anchor Biosynthesis Class O	Protein Coding	43	GC09M035088	5.123737
IGBP1	Immunoglobulin Binding Protein 1	Protein Coding	43	GC0XP070133	5.08256
LTBP1	Latent Transforming Growth Factor Beta Binding Protein 1	Protein Coding	43	GC02P032949	5.059715
ELAVL1	ELAV Like RNA Binding Protein 1	Protein Coding	43	GC19M007958	5.044666
COQ7	Coenzyme Q7, Hydroxylase	Protein Coding	43	GC16P019067	5.032852
ADD3	Adducin 3	Protein Coding	43	GC10P109996	5.015781
DDB1	Damage Specific DNA Binding Protein 1	Protein Coding	43	GC11M069196	5.002128
DHPS	Deoxyhypusine Synthase	Protein Coding	43	GC19M013051	4.987686
S100A9	S100 Calcium Binding Protein A9	Protein Coding	43	GC01P153357	4.958222
ERF	ETS2 Repressor Factor	Protein Coding	43	GC19M042247	4.924588
TWIST2	Twist Family BHLH Transcription Factor 2	Protein Coding	43	GC02P238848	4.908947
IL18R1	Interleukin 18 Receptor 1	Protein Coding	43	GC02P102311	4.906329
DNAJB6	DnaJ Heat Shock Protein Family (Hsp40) Member B6	Protein Coding	43	GC07P157335	4.901154
MMADHC	Metabolism Of Cobalamin Associated D	Protein Coding	43	GC02M149569	4.883592
PIGV	Phosphatidylinositol Glycan Anchor Biosynthesis Class V	Protein Coding	43	GC01P026787	4.875278
TRPM2	Transient Receptor Potential Cation Channel Subfamily M Member 2	Protein Coding	43	GC21P044350	4.85738
CGA	Glycoprotein Hormones, Alpha Polypeptide	Protein Coding	43	GC06M087085	4.840911
FOXE1	Forkhead Box E1	Protein Coding	43	GC09P097853	4.836518
NDUFA8	NADH:Ubiquinone Oxidoreductase Subunit A8	Protein Coding	43	GC09M122132	4.832689
COQ6	Coenzyme Q6, Monooxygenase	Protein Coding	43	GC14P073949	4.753434
FABP6	Fatty Acid Binding Protein 6	Protein Coding	43	GC05P160187	4.710402
H6PD	Hexose-6-Phosphate Dehydrogenase/Glucose 1-Dehydrogenase	Protein Coding	43	GC01P009234	4.710387
TUSC3	Tumor Suppressor Candidate 3	Protein Coding	43	GC08P015417	4.696296
TRMT1	TRNA Methyltransferase 1	Protein Coding	43	GC19M013104	4.695011
MX1	MX Dynamin Like GTPase 1	Protein Coding	43	GC21P041420	4.683966
SPRY1	Sprouty RTK Signaling Antagonist 1	Protein Coding	43	GC04P123396	4.664636
SFTPA1	Surfactant Protein A1	Protein Coding	43	GC10P086009	4.663832
HAPLN1	Hyaluronan And Proteoglycan Link Protein 1	Protein Coding	43	GC05M083637	4.65402
ATG7	Autophagy Related 7	Protein Coding	43	GC03P011273	4.650947
TERF1	Telomeric Repeat Binding Factor 1	Protein Coding	43	GC08P073003	4.617228
MOGS	Mannosyl-Oligosaccharide Glucosidase	Protein Coding	43	GC02M074461	4.614408
ANLN	Anillin Actin Binding Protein	Protein Coding	43	GC07P036389	4.60153
GNB4	G Protein Subunit Beta 4	Protein Coding	43	GC03M179397	4.581988
ROBO2	Roundabout Guidance Receptor 2	Protein Coding	43	GC03P075955	4.564888
CACNA2D2	Calcium Voltage-Gated Channel Auxiliary Subunit Alpha2delta 2	Protein Coding	43	GC03M050433	4.564649
IGFBP6	Insulin Like Growth Factor Binding Protein 6	Protein Coding	43	GC12P053097	4.551368
SAMHD1	SAM And HD Domain Containing Deoxynucleoside Triphosphate Triphosphohydrolase 1	Protein Coding	43	GC20M036890	4.518644
GNA15	G Protein Subunit Alpha 15	Protein Coding	43	GC19P003173	4.510127
SLC30A8	Solute Carrier Family 30 Member 8	Protein Coding	43	GC08P116950	4.508947
CX3CL1	C-X3-C Motif Chemokine Ligand 1	Protein Coding	43	GC16P057372	4.475326

LAMP1	Lysosomal Associated Membrane Protein 1	Protein Coding	43	GC13P113297	4.475194
PUS1	Pseudouridine Synthase 1	Protein Coding	43	GC12P131929	4.468436
CPLX1	Complexin 1	Protein Coding	43	GC04M000784	4.438279
KLHL3	Kelch Like Family Member 3	Protein Coding	43	GC05M137617	4.435513
MEOX2	Mesenchyme Homeobox 2	Protein Coding	43	GC07M015617	4.426245
APOD	Apolipoprotein D	Protein Coding	43	GC03M195568	4.420343
CNBP	CCHC-Type Zinc Finger Nucleic Acid Binding Protein	Protein Coding	43	GC03M129167	4.414376
DHDDS	Dehydrodolichyl Diphosphate Synthase Subunit	Protein Coding	43	GC01P026432	4.389885
TIE1	Tyrosine Kinase With Immunoglobulin Like And EGF Like Domains 1	Protein Coding	43	GC01P043300	4.388289
GM2A	GM2 Ganglioside Activator	Protein Coding	43	GC05P151229	4.372289
HHEX	Hematopoietically Expressed Homeobox	Protein Coding	43	GC10P092689	4.346951
BBOX1	Gamma-Butyrobetaine Hydroxylase 1	Protein Coding	43	GC11P027019	4.341123
CDCA7	Cell Division Cycle Associated 7	Protein Coding	43	GC02P173354	4.334473
NLGN3	Neuroligin 3	Protein Coding	43	GC0XP071144	4.311371
ICMT	Isoprenylcysteine Carboxyl Methyltransferase	Protein Coding	43	GC01M006222	4.309658
UBA5	Ubiquitin Like Modifier Activating Enzyme 5	Protein Coding	43	GC03P132654	4.303225
RNF31	Ring Finger Protein 31	Protein Coding	43	GC14P024146	4.291743
MLH3	MutL Homolog 3	Protein Coding	43	GC14M075013	4.247439
HPS1	HPS1 Biogenesis Of Lysosomal Organelles Complex 3 Subunit 1	Protein Coding	43	GC10M098416	4.232967
OSM	Oncostatin M	Protein Coding	43	GC22M030262	4.218149
SLC13A5	Solute Carrier Family 13 Member 5	Protein Coding	43	GC17M006684	4.204786
RIN2	Ras And Rab Interactor 2	Protein Coding	43	GC20P019759	4.201153
PTN	Pleiotrophin	Protein Coding	43	GC07M137227	4.194372
COL9A1	Collagen Type IX Alpha 1 Chain	Protein Coding	43	GC06M070215	4.191119
PARS2	Prolyl-TRNA Synthetase 2, Mitochondrial	Protein Coding	43	GC01M054756	4.189811
PHF21A	PHD Finger Protein 21A	Protein Coding	43	GC11M068967	4.168992
PRRX1	Paired Related Homeobox 1	Protein Coding	43	GC01P170662	4.163432
EARS2	Glutamyl-TRNA Synthetase 2, Mitochondrial	Protein Coding	43	GC16M023527	4.15647
MARS2	Methionyl-TRNA Synthetase 2, Mitochondrial	Protein Coding	43	GC02P197705	4.129502
ITGA1	Integrin Subunit Alpha 1	Protein Coding	43	GC05P052788	4.12754
CADM1	Cell Adhesion Molecule 1	Protein Coding	43	GC11M115169	4.113969
TFB1M	Transcription Factor B1, Mitochondrial	Protein Coding	43	GC06M155247	4.112154
AP2B1	Adaptor Related Protein Complex 2 Subunit Beta 1	Protein Coding	43	GC17P035578	4.102136
CRHR2	Corticotropin Releasing Hormone Receptor 2	Protein Coding	43	GC07M030651	4.099103
ADAMTS5	ADAM Metallopeptidase With Thrombospondin Type 1 Motif 5	Protein Coding	43	GC21M026918	4.0938
VARS2	Valyl-TRNA Synthetase 2, Mitochondrial	Protein Coding	43	GC06P055186	4.066949
IL16	Interleukin 16	Protein Coding	43	GC15P081159	4.056955
BDKRB1	Bradykinin Receptor B1	Protein Coding	43	GC14P096272	4.050279
CXCL1	C-X-C Motif Chemokine Ligand 1	Protein Coding	43	GC04P073869	4.032602
TPR	Translocated Promoter Region, Nuclear Basket Protein	Protein Coding	43	GC01M186319	4.027646
EXOSC3	Exosome Component 3	Protein Coding	43	GC09M037772	4.021449
KCNMB1	Potassium Calcium-Activated Channel Subfamily M Regulatory Beta Subunit 1	Protein Coding	43	GC05M170374	4.012144
OPCML	Opioid Binding Protein/Cell Adhesion Molecule Like	Protein Coding	43	GC11M132405	4.008875
ADH1C	Alcohol Dehydrogenase 1C (Class I), Gamma Polypeptide	Protein Coding	43	GC04M099336	3.999
S100A8	S100 Calcium Binding Protein A8	Protein Coding	43	GC01M153391	3.986601
DIS3L2	DIS3 Like 3'-5' Exoribonuclease 2	Protein Coding	43	GC02P231961	3.978403
LPIN2	Lipin 2	Protein Coding	43	GC18M002916	3.964144
RFXANK	Regulatory Factor X Associated Ankyrin Containing Protein	Protein Coding	43	GC19P019192	3.963863
CD1D	CD1d Molecule	Protein Coding	43	GC01P158178	3.963227
DLGAP1	DLG Associated Protein 1	Protein Coding	43	GC18M003488	3.956759
HRH3	Histamine Receptor H3	Protein Coding	43	GC20M062214	3.940104
ADCY4	Adenylate Cyclase 4	Protein Coding	43	GC14M024318	3.938103
IGFBP5	Insulin Like Growth Factor Binding Protein 5	Protein Coding	43	GC02M216672	3.911756

ACTL6B	Actin Like 6B	Protein Coding	43	GC07M100643	3.903396
IL1RAPL1	Interleukin 1 Receptor Accessory Protein Like 1	Protein Coding	43	GC0XP028605	3.899833
IGFBP4	Insulin Like Growth Factor Binding Protein 4	Protein Coding	43	GC17P040443	3.895717
MAP2	Microtubule Associated Protein 2	Protein Coding	43	GC02P209424	3.889032
MLST8	MTOR Associated Protein, LST8 Homolog	Protein Coding	43	GC16P002204	3.870158
RIPK3	Receptor Interacting Serine/Threonine Kinase 3	Protein Coding	43	GC14M024336	3.864462
OSGEP	O-Sialoglycoprotein Endopeptidase	Protein Coding	43	GC14M020446	3.850727
NME7	NME/NM23 Family Member 7	Protein Coding	43	GC01M169101	3.796964
MFAP2	Microfibril Associated Protein 2	Protein Coding	43	GC01M016974	3.793511
BHMT	Betaine--Homocysteine S-Methyltransferase	Protein Coding	43	GC05P079111	3.779867
NEUROD2	Neuronal Differentiation 2	Protein Coding	43	GC17M039603	3.779221
KIF1A	Kinesin Family Member 1A	Protein Coding	43	GC02M240713	3.768138
STAG1	Stromal Antigen 1	Protein Coding	43	GC03M136336	3.76427
SETDB1	SET Domain Bifurcated Histone Lysine Methyltransferase 1	Protein Coding	43	GC01P150926	3.759526
AIMP1	Aminoacyl TRNA Synthetase Complex Interacting Multifunctional Protein 1	Protein Coding	43	GC04P106315	3.755517
DLG2	Discs Large MAGUK Scaffold Protein 2	Protein Coding	43	GC11M083455	3.744632
MEIS1	Meis Homeobox 1	Protein Coding	43	GC02P066433	3.741254
RPL13A	Ribosomal Protein L13a	Protein Coding	43	GC19P049487	3.735892
NRG3	Neuregulin 3	Protein Coding	43	GC10P085390	3.702772
PPP1R1A	Protein Phosphatase 1 Regulatory Inhibitor Subunit 1A	Protein Coding	43	GC12M054576	3.699354
CD209	CD209 Molecule	Protein Coding	43	GC19M007739	3.696862
RND3	Rho Family GTPase 3	Protein Coding	43	GC02M150468	3.660965
CDK5RAP2	CDK5 Regulatory Subunit Associated Protein 2	Protein Coding	43	GC09M120388	3.65911
P2RX3	Purinergic Receptor P2X 3	Protein Coding	43	GC11P057356	3.650642
CD63	CD63 Molecule	Protein Coding	43	GC12M055725	3.64032
NTF3	Neurotrophin 3	Protein Coding	43	GC12P005432	3.63121
RNF168	Ring Finger Protein 168	Protein Coding	43	GC03M196468	3.63021
TRPV2	Transient Receptor Potential Cation Channel Subfamily V Member 2	Protein Coding	43	GC17P016415	3.629441
USP25	Ubiquitin Specific Peptidase 25	Protein Coding	43	GC21P015730	3.625014
TREM2	Triggering Receptor Expressed On Myeloid Cells 2	Protein Coding	43	GC06M047080	3.618482
EMP2	Epithelial Membrane Protein 2	Protein Coding	43	GC16M010541	3.611422
MNX1	Motor Neuron And Pancreas Homeobox 1	Protein Coding	43	GC07M156994	3.604544
BNIP3	BCL2 Interacting Protein 3	Protein Coding	43	GC10M131966	3.600295
FGF7	Fibroblast Growth Factor 7	Protein Coding	43	GC15P049423	3.598653
S100A6	S100 Calcium Binding Protein A6	Protein Coding	43	GC01M153535	3.586281
TMEM38B	Transmembrane Protein 38B	Protein Coding	43	GC09P105694	3.583121
SEC61A1	SEC61 Translocon Subunit Alpha 1	Protein Coding	43	GC03P128051	3.579462
DSC3	Desmocollin 3	Protein Coding	43	GC18M030990	3.572442
TRIT1	TRNA Isopentenyltransferase 1	Protein Coding	43	GC01M039842	3.569894
HS3ST1	Heparan Sulfate-Glucosamine 3-Sulfotransferase 1	Protein Coding	43	GC04M011394	3.565581
MOCOS	Molybdenum Cofactor Sulfurase	Protein Coding	43	GC18P036187	3.563218
SELPLG	Selectin P Ligand	Protein Coding	43	GC12M108621	3.546343
TNFRSF8	TNF Receptor Superfamily Member 8	Protein Coding	43	GC01P012063	3.528215
DBT	Dihydrolipoamide Branched Chain Transacylase E2	Protein Coding	43	GC01M100186	3.523399
TMLHE	Trimethyllysine Hydroxylase, Epsilon	Protein Coding	43	GC0XM155489	3.522696
DLG3	Discs Large MAGUK Scaffold Protein 3	Protein Coding	43	GC0XP070444	3.515022
PYCARD	PYD And CARD Domain Containing	Protein Coding	43	GC16M031201	3.512931
LOXL1	Lysyl Oxidase Like 1	Protein Coding	43	GC15P073925	3.511554
ADCYAP1	Adenylate Cyclase Activating Polypeptide 1	Protein Coding	43	GC18P000895	3.507304
PYY	Peptide YY	Protein Coding	43	GC17M043952	3.506058
ADAM19	ADAM Metallopeptidase Domain 19	Protein Coding	43	GC05M157395	3.497419
EIF2B3	Eukaryotic Translation Initiation Factor 2B Subunit Gamma	Protein Coding	43	GC01M044850	3.479013
SLC4A7	Solute Carrier Family 4 Member 7	Protein Coding	43	GC03M027372	3.476336

LYVE1	Lymphatic Vessel Endothelial Hyaluronan Receptor 1	Protein Coding	43	GC11M010713	3.472934
MYF6	Myogenic Factor 6	Protein Coding	43	GC12P080707	3.456764
SRGAP1	SLIT-ROBO Rho GTPase Activating Protein 1	Protein Coding	43	GC12P063844	3.453023
RPL12	Ribosomal Protein L12	Protein Coding	43	GC09M127447	3.447706
MOCS2	Molybdenum Cofactor Synthesis 2	Protein Coding	43	GC05M053095	3.432144
CRBN	Cereblon	Protein Coding	43	GC03M003144	3.425873
RALGDS	Ral Guanine Nucleotide Dissociation Stimulator	Protein Coding	43	GC09M133097	3.424641
UGCG	UDP-Glucose Ceramide Glucosyltransferase	Protein Coding	43	GC09P111896	3.418541
KIRREL3	Kirre Like Nephrin Family Adhesion Molecule 3	Protein Coding	43	GC11M126423	3.407501
GMPPA	GDP-Mannose Pyrophosphorylase A	Protein Coding	43	GC02P219498	3.405137
NEUROG3	Neurogenin 3	Protein Coding	43	GC10M069571	3.404798
RBX1	Ring-Box 1	Protein Coding	43	GC22P040951	3.402723
PODXL	Podocalyxin Like	Protein Coding	43	GC07M131500	3.393787
SPTBN2	Spectrin Beta, Non-Erythrocytic 2	Protein Coding	43	GC11M069493	3.393021
FGF13	Fibroblast Growth Factor 13	Protein Coding	43	GC0XM138615	3.392181
ADAMTS2	ADAM Metallopeptidase With Thrombospondin Type 1 Motif 2	Protein Coding	43	GC05M179110	3.38943
LEFTY1	Left-Right Determination Factor 1	Protein Coding	43	GC01M225887	3.387391
HPGDS	Hematopoietic Prostaglandin D Synthase	Protein Coding	43	GC04M094298	3.386978
DOCK8	Dedicator Of Cytokinesis 8	Protein Coding	43	GC09P000214	3.383749
MTMR3	Myotubularin Related Protein 3	Protein Coding	43	GC22P029885	3.377762
CREM	CAMP Responsive Element Modulator	Protein Coding	43	GC10P035126	3.372083
SLC4A5	Solute Carrier Family 4 Member 5	Protein Coding	43	GC02M074217	3.359715
GRB10	Growth Factor Receptor Bound Protein 10	Protein Coding	43	GC07M050590	3.349615
ATXN10	Ataxin 10	Protein Coding	43	GC22P045673	3.342958
CNOT2	CCR4-NOT Transcription Complex Subunit 2	Protein Coding	43	GC12P070242	3.33249
HNRNPU	Heterogeneous Nuclear Ribonucleoprotein U	Protein Coding	43	GC01M244844	3.331662
PER1	Period Circadian Regulator 1	Protein Coding	43	GC17M009186	3.328653
LMO1	LIM Domain Only 1	Protein Coding	43	GC11M008224	3.326951
EED	Embryonic Ectoderm Development	Protein Coding	43	GC11P086244	3.309541
GPAA1	Glycosylphosphatidylinositol Anchor Attachment 1	Protein Coding	43	GC08P144082	3.304708
MANBA	Mannosidase Beta	Protein Coding	43	GC04M102631	3.295294
KRT10	Keratin 10	Protein Coding	43	GC17M040818	3.291094
SEC13	SEC13 Homolog, Nuclear Pore And COPII Coat Complex Component	Protein Coding	43	GC03M010293	3.28008
ETHE1	ETHE1 Persulfide Dioxygenase	Protein Coding	43	GC19M043506	3.277994
HBG1	Hemoglobin Subunit Gamma 1	Protein Coding	43	GC11M005431	3.267973
ELAVL2	ELAV Like RNA Binding Protein 2	Protein Coding	43	GC09M023690	3.264386
PDGFRL	Platelet Derived Growth Factor Receptor Like	Protein Coding	43	GC08P017576	3.262412
PDCD10	Programmed Cell Death 10	Protein Coding	43	GC03M167683	3.259984
ATP8A1	ATPase Phospholipid Transporting 8A1	Protein Coding	43	GC04M042410	3.258858
SECISBP2	SECIS Binding Protein 2	Protein Coding	43	GC09P089318	3.243493
PREP	Prolyl Endopeptidase	Protein Coding	43	GC06M105277	3.241482
STX3	Syntaxin 3	Protein Coding	43	GC11P059713	3.229614
ANXA7	Annexin A7	Protein Coding	43	GC10M073375	3.229563
SREBF2	Sterol Regulatory Element Binding Transcription Factor 2	Protein Coding	43	GC22P041833	3.228648
LHX1	LIM Homeobox 1	Protein Coding	43	GC17P036937	3.227115
HACE1	HECT Domain And Ankyrin Repeat Containing E3 Ubiquitin Protein Ligase 1	Protein Coding	43	GC06M104728	3.216046
CUL7	Cullin 7	Protein Coding	43	GC06M043037	3.201623
ASH2L	ASH2 Like, Histone Lysine Methyltransferase Complex Subunit	Protein Coding	43	GC08P038104	3.198695
RPL7	Ribosomal Protein L7	Protein Coding	43	GC08M073290	3.187868
COL14A1	Collagen Type XIV Alpha 1 Chain	Protein Coding	43	GC08P120070	3.163747
HHAT	Hedgehog Acyltransferase	Protein Coding	43	GC01P210328	3.153445
KRT7	Keratin 7	Protein Coding	43	GC12P052232	3.144893
NUDC	Nuclear Distribution C, Dynein Complex Regulator	Protein Coding	43	GC01P026925	3.131884

NFAT5	Nuclear Factor Of Activated T Cells 5	Protein Coding	43	GC16P069565	3.127236
NIN	Ninein	Protein Coding	43	GC14M050719	3.125196
PTBP1	Polypyrimidine Tract Binding Protein 1	Protein Coding	43	GC19P000797	3.123062
THY1	Thy-1 Cell Surface Antigen	Protein Coding	43	GC11M119417	3.113819
FMO5	Flavin Containing Dimethylaniline Monoxygenase 5	Protein Coding	43	GC01M147175	3.107175
PTPRM	Protein Tyrosine Phosphatase Receptor Type M	Protein Coding	43	GC18P007557	3.106402
UBR5	Ubiquitin Protein Ligase E3 Component N-Recognin 5	Protein Coding	43	GC08M102252	3.075475
SFRP1	Secreted Frizzled Related Protein 1	Protein Coding	43	GC08M041262	3.075167
RXRG	Retinoid X Receptor Gamma	Protein Coding	43	GC01M165401	3.072722
MYOC	Myocilin	Protein Coding	43	GC01M171604	3.072594
TNFRSF6B	TNF Receptor Superfamily Member 6b	Protein Coding	43	GC20P063696	3.070648
WWTR1	WW Domain Containing Transcription Regulator 1	Protein Coding	43	GC03M149517	3.068424
AP2S1	Adaptor Related Protein Complex 2 Subunit Sigma 1	Protein Coding	43	GC19M046838	3.067548
BCL11B	BAF Chromatin Remodeling Complex Subunit BCL11B	Protein Coding	43	GC14M099169	3.058333
GLYCTK	Glycerate Kinase	Protein Coding	43	GC03P052288	3.04612
ST3GAL4	ST3 Beta-Galactoside Alpha-2,3-Sialyltransferase 4	Protein Coding	43	GC11P126355	3.040015
COMMD1	Copper Metabolism Domain Containing 1	Protein Coding	43	GC02P061888	3.031724
JAG2	Jagged Canonical Notch Ligand 2	Protein Coding	43	GC14M105140	3.029398
CLCN3	Chloride Voltage-Gated Channel 3	Protein Coding	43	GC04P169612	3.014117
CDH4	Cadherin 4	Protein Coding	43	GC20P061252	3.013101
PABPC1	Poly(A) Binding Protein Cytoplasmic 1	Protein Coding	43	GC08M100685	3.009261
ST3GAL1	ST3 Beta-Galactoside Alpha-2,3-Sialyltransferase 1	Protein Coding	43	GC08M133454	3.006745
GDAP1	Ganglioside Induced Differentiation Associated Protein 1	Protein Coding	43	GC08P074315	3.005033
PSMC5	Proteasome 26S Subunit, ATPase 5	Protein Coding	43	GC17P063827	3.004497
ETS2	ETS Proto-Oncogene 2, Transcription Factor	Protein Coding	43	GC21P038805	3.002012
RASGRP3	RAS Guanyl Releasing Protein 3	Protein Coding	43	GC02P033436	2.995849
SLC7A8	Solute Carrier Family 7 Member 8	Protein Coding	43	GC14M023125	2.99075
HRG	Histidine Rich Glycoprotein	Protein Coding	43	GC03P186660	2.986786
PRPF6	Pre-MRNA Processing Factor 6	Protein Coding	43	GC20P063981	2.979982
CD70	CD70 Molecule	Protein Coding	43	GC19M006583	2.974469
CD1A	CD1a Molecule	Protein Coding	43	GC01P158255	2.969621
FERMT1	FERM Domain Containing Kindlin 1	Protein Coding	43	GC20M006074	2.952585
NCL	Nucleolin	Protein Coding	43	GC02M231453	2.951441
GNB2	G Protein Subunit Beta 2	Protein Coding	43	GC07P100673	2.937145
REV1	REV1 DNA Directed Polymerase	Protein Coding	43	GC02M099383	2.92324
NFASC	Neurofascin	Protein Coding	43	GC01P204828	2.911128
MED23	Mediator Complex Subunit 23	Protein Coding	43	GC06M131573	2.90834
AGTPBP1	ATP/GTP Binding Carboxypeptidase 1	Protein Coding	43	GC09M085546	2.889842
HIPK3	Homeodomain Interacting Protein Kinase 3	Protein Coding	43	GC11P033278	2.885817
SRSF1	Serine And Arginine Rich Splicing Factor 1	Protein Coding	43	GC17M058000	2.879513
ALOX12B	Arachidonate 12-Lipoxygenase, 12R Type	Protein Coding	43	GC17M009184	2.873181
CAV2	Caveolin 2	Protein Coding	43	GC07P116287	2.869207
FRS2	Fibroblast Growth Factor Receptor Substrate 2	Protein Coding	43	GC12P069471	2.865854
KDM3B	Lysine Demethylase 3B	Protein Coding	43	GC05P138352	2.865343
PPM1K	Protein Phosphatase, Mg2+/Mn2+ Dependent 1K	Protein Coding	43	GC04M088258	2.859594
TERF2IP	TERF2 Interacting Protein	Protein Coding	43	GC16P075647	2.84355
GSC	Goosecoid Homeobox	Protein Coding	43	GC14M094768	2.83906
CENPJ	Centromere Protein J	Protein Coding	43	GC13M024882	2.831918
GATAD2B	GATA Zinc Finger Domain Containing 2B	Protein Coding	43	GC01M153805	2.823582
UVRAG	UV Radiation Resistance Associated	Protein Coding	43	GC11P075815	2.820766
MATN3	Matrilin 3	Protein Coding	43	GC02M019992	2.808078
TSHB	Thyroid Stimulating Hormone Subunit Beta	Protein Coding	43	GC01P115029	2.805723
TAB1	TGF-Beta Activated Kinase 1 (MAP3K7) Binding Protein 1	Protein Coding	43	GC22P039403	2.799277

NHEJ1	Non-Homologous End Joining Factor 1	Protein Coding	43	GC02M219086	2.79819
SMARCD2	SWI/SNF Related, Matrix Associated, Actin Dependent Regulator Of Chromatin, Subfamily D, Member 2	Protein Coding	43	GC17M063832	2.795428
AGPAT1	1-Acylglycerol-3-Phosphate O-Acyltransferase 1	Protein Coding	43	GC06M032168	2.776614
RB1CC1	RB1 Inducible Coiled-Coil 1	Protein Coding	43	GC08M052622	2.754658
KCNT1	Potassium Sodium-Activated Channel Subfamily T Member 1	Protein Coding	43	GC09P135702	2.743222
CENPF	Centromere Protein F	Protein Coding	43	GC01P214603	2.734216
CD244	CD244 Molecule	Protein Coding	43	GC01M160830	2.732484
MAN2A1	Mannosidase Alpha Class 2A Member 1	Protein Coding	43	GC05P109689	2.729322
NDUFA5	NADH:Ubiquinone Oxidoreductase Subunit A5	Protein Coding	43	GC07M123536	2.725751
ENDOG	Endonuclease G	Protein Coding	43	GC09P128818	2.71682
COL9A2	Collagen Type IX Alpha 2 Chain	Protein Coding	43	GC01M040300	2.716103
CALB1	Calbindin 1	Protein Coding	43	GC08M090058	2.715374
DYNLL1	Dynein Light Chain LC8-Type 1	Protein Coding	43	GC12P120469	2.70639
ALOXE3	Arachidonate Lipoxygenase 3	Protein Coding	43	GC17M009179	2.695503
MTHFD1L	Methylenetetrahydrofolate Dehydrogenase (NADP+ Dependent) 1 Like	Protein Coding	43	GC06P150865	2.687102
PTPRG	Protein Tyrosine Phosphatase Receptor Type G	Protein Coding	43	GC03P061561	2.668629
JMJD6	Jumonji Domain Containing 6, Arginine Demethylase And Lysine Hydroxylase	Protein Coding	43	GC17M076718	2.666898
MCEE	Methylmalonyl-CoA Epimerase	Protein Coding	43	GC02M071110	2.644545
SLC30A10	Solute Carrier Family 30 Member 10	Protein Coding	43	GC01M219685	2.644271
SRD5A2	Steroid 5 Alpha-Reductase 2	Protein Coding	43	GC02M031522	2.64281
GJA3	Gap Junction Protein Alpha 3	Protein Coding	43	GC13M020139	2.640265
POLI	DNA Polymerase Iota	Protein Coding	43	GC18P054274	2.639801
UROS	Uroporphyrinogen III Synthase	Protein Coding	43	GC10M125784	2.638892
INPP4A	Inositol Polyphosphate-4-Phosphatase Type I A	Protein Coding	43	GC02P098465	2.637525
TGM3	Transglutaminase 3	Protein Coding	43	GC20P002296	2.637081
VAMP7	Vesicle Associated Membrane Protein 7	Protein Coding	43	GC0XP155881	2.629489
CNNM2	Cyclin And CBS Domain Divalent Metal Cation Transport Mediator 2	Protein Coding	43	GC10P102918	2.627993
MGST3	Microsomal Glutathione S-Transferase 3	Protein Coding	43	GC01P165632	2.621567
POLR2B	RNA Polymerase II Subunit B	Protein Coding	43	GC04P056977	2.613894
SFRP2	Secreted Frizzled Related Protein 2	Protein Coding	43	GC04M153780	2.613221
GUCA1A	Guanylate Cyclase Activator 1A	Protein Coding	43	GC06P055395	2.612862
CDK19	Cyclin Dependent Kinase 19	Protein Coding	43	GC06M110609	2.610679
TP53BP2	Tumor Protein P53 Binding Protein 2	Protein Coding	43	GC01M223779	2.606098
AIM2	Absent In Melanoma 2	Protein Coding	43	GC01M159062	2.604963
ADAMTS4	ADAM Metallopeptidase With Thrombospondin Type 1 Motif 4	Protein Coding	43	GC01M161184	2.603196
STX16	Syntaxin 16	Protein Coding	43	GC20P058652	2.591095
LMX1A	LIM Homeobox Transcription Factor 1 Alpha	Protein Coding	43	GC01M165171	2.588558
XRCC3	X-Ray Repair Cross Complementing 3	Protein Coding	43	GC14M103697	2.562662
MYL6	Myosin Light Chain 6	Protein Coding	43	GC12P056158	2.554189
CDO1	Cysteine Dioxygenase Type 1	Protein Coding	43	GC05M115804	2.550935
SUCLG2	Succinate-CoA Ligase GDP-Forming Subunit Beta	Protein Coding	43	GC03M067358	2.548009
INPP5A	Inositol Polyphosphate-5-Phosphatase A	Protein Coding	43	GC10P132537	2.538757
GPAM	Glycerol-3-Phosphate Acyltransferase, Mitochondrial	Protein Coding	43	GC10M112148	2.536535
CCL21	C-C Motif Chemokine Ligand 21	Protein Coding	43	GC09M034709	2.528486
SP3	Sp3 Transcription Factor	Protein Coding	43	GC02M173882	2.52625
CACNG1	Calcium Voltage-Gated Channel Auxiliary Subunit Gamma 1	Protein Coding	43	GC17P067044	2.509425
SMAD5	SMAD Family Member 5	Protein Coding	43	GC05P136132	2.502806
TULP1	TUB Like Protein 1	Protein Coding	43	GC06M047024	2.501509
PLD3	Phospholipase D Family Member 3	Protein Coding	43	GC19P040348	2.497701
RNMT	RNA Guanine-7 Methyltransferase	Protein Coding	43	GC18P014026	2.491984
GART	Phosphoribosylglycinamide Formyltransferase, Phosphoribosylglycinamide Synthetase, Phosphoribosylaminoimidazole Syr	Protein Coding	43	GC21M033503	2.478272
ALCAM	Activated Leukocyte Cell Adhesion Molecule	Protein Coding	43	GC03P105366	2.471622
WASL	WASP Like Actin Nucleation Promoting Factor	Protein Coding	43	GC07M123681	2.466294

RXFP2	Relaxin Family Peptide Receptor 2	Protein Coding	43	GC13P031739	2.464121
JAM2	Junctional Adhesion Molecule 2	Protein Coding	43	GC21P025639	2.464048
RPL7A	Ribosomal Protein L7a	Protein Coding	43	GC09P133348	2.457261
PPM1G	Protein Phosphatase, Mg2+/Mn2+ Dependent 1G	Protein Coding	43	GC02M027399	2.449631
P2RY6	Pyrimidinergic Receptor P2Y6	Protein Coding	43	GC11P073264	2.446933
C6	Complement C6	Protein Coding	43	GC05M041142	2.443799
IQSEC1	IQ Motif And Sec7 Domain ArfGEF 1	Protein Coding	43	GC03M016907	2.440522
ID3	Inhibitor Of DNA Binding 3, HLH Protein	Protein Coding	43	GC01M023557	2.43388
PTPRU	Protein Tyrosine Phosphatase Receptor Type U	Protein Coding	43	GC01P029236	2.427727
LAMC3	Laminin Subunit Gamma 3	Protein Coding	43	GC09P131009	2.418412
GJD2	Gap Junction Protein Delta 2	Protein Coding	43	GC15M034751	2.410852
RAD18	RAD18 E3 Ubiquitin Protein Ligase	Protein Coding	43	GC03M008775	2.410018
SGCE	Sarcoglycan Epsilon	Protein Coding	43	GC07M094585	2.404994
DUSP5	Dual Specificity Phosphatase 5	Protein Coding	43	GC10P110497	2.399853
COPA	COPI Coat Complex Subunit Alpha	Protein Coding	43	GC01M160288	2.394543
MASTL	Microtubule Associated Serine/Threonine Kinase Like	Protein Coding	43	GC10P027154	2.374316
DIAPH3	Diaphanous Related Formin 3	Protein Coding	43	GC13M059665	2.364578
IL13RA1	Interleukin 13 Receptor Subunit Alpha 1	Protein Coding	43	GC0XP118727	2.362596
LMO2	LIM Domain Only 2	Protein Coding	43	GC11M033858	2.361539
CBX2	Chromobox 2	Protein Coding	43	GC17P079778	2.35245
LPCAT2	Lysophosphatidylcholine Acyltransferase 2	Protein Coding	43	GC16P055510	2.349956
HECW2	HECT, C2 And WW Domain Containing E3 Ubiquitin Protein Ligase 2	Protein Coding	43	GC02M196194	2.341279
INPP5B	Inositol Polyphosphate-5-Phosphatase B	Protein Coding	43	GC01M037860	2.335093
ARHGEF6	Rac/Cdc42 Guanine Nucleotide Exchange Factor 6	Protein Coding	43	GC0XM136665	2.332768
BRD7	Bromodomain Containing 7	Protein Coding	43	GC16M050313	2.327608
CNTNAP1	Contactin Associated Protein 1	Protein Coding	43	GC17P042680	2.311741
CDIPT	CDP-Diacylglycerol--Inositol 3-Phosphatidyltransferase	Protein Coding	43	GC16M031170	2.310886
ITIH4	Inter-Alpha-Trypsin Inhibitor Heavy Chain 4	Protein Coding	43	GC03M052812	2.297456
TFAP2C	Transcription Factor AP-2 Gamma	Protein Coding	43	GC20P056629	2.294245
TGFB1I1	Transforming Growth Factor Beta 1 Induced Transcript 1	Protein Coding	43	GC16P032576	2.283803
DUSP4	Dual Specificity Phosphatase 4	Protein Coding	43	GC08M029334	2.274953
DCT	Dopachrome Tautomerase	Protein Coding	43	GC13M094436	2.266967
TESK1	Testis Associated Actin Remodelling Kinase 1	Protein Coding	43	GC09P035605	2.266891
SLC7A2	Solute Carrier Family 7 Member 2	Protein Coding	43	GC08P017497	2.249542
FGD4	FYVE, RhoGEF And PH Domain Containing 4	Protein Coding	43	GC12P032407	2.216525
DNMT3L	DNA Methyltransferase 3 Like	Protein Coding	43	GC21M044246	2.205209
PNKD	PNKD Metallo-Beta-Lactamase Domain Containing	Protein Coding	43	GC02P218270	2.19764
ABCC11	ATP Binding Cassette Subfamily C Member 11	Protein Coding	43	GC16M048166	2.19142
UBE2Q2	Ubiquitin Conjugating Enzyme E2 Q2	Protein Coding	43	GC15P075843	2.173892
LCT	Lactase	Protein Coding	43	GC02M135787	2.169004
LDHC	Lactate Dehydrogenase C	Protein Coding	43	GC11P018433	2.162623
LGR6	Leucine Rich Repeat Containing G Protein-Coupled Receptor 6	Protein Coding	43	GC01P202193	2.157681
HNRNPD	Heterogeneous Nuclear Ribonucleoprotein D	Protein Coding	43	GC04M082352	2.15625
CD82	CD82 Molecule	Protein Coding	43	GC11P044586	2.155453
RRAGC	Ras Related GTP Binding C	Protein Coding	43	GC01M038847	2.154813
NCALD	Neurocalcin Delta	Protein Coding	43	GC08M101704	2.149676
INHBB	Inhibin Subunit Beta B	Protein Coding	43	GC02P120444	2.146071
KDM5A	Lysine Demethylase 5A	Protein Coding	43	GC12M000280	2.140767
DROSHA	Drosha Ribonuclease III	Protein Coding	43	GC05M031401	2.122772
PRPF4	Pre-MRNA Processing Factor 4	Protein Coding	43	GC09P113275	2.120581
DAGLA	Diacylglycerol Lipase Alpha	Protein Coding	43	GC11P061680	2.110471
LIN28B	Lin-28 Homolog B	Protein Coding	43	GC06P104943	2.108234
DPEP1	Dipeptidase 1	Protein Coding	43	GC16P089613	2.103202

PRIM1	DNA Primase Subunit 1	Protein Coding	43	GC12M056731	2.102788
RAD23A	RAD23 Homolog A, Nucleotide Excision Repair Protein	Protein Coding	43	GC19P012954	2.094702
CALD1	Caldesmon 1	Protein Coding	43	GC07P134744	2.087281
MAP4K3	Mitogen-Activated Protein Kinase Kinase Kinase Kinase 3	Protein Coding	43	GC02M039249	2.084673
EPB41L1	Erythrocyte Membrane Protein Band 4.1 Like 1	Protein Coding	43	GC20P036064	2.081971
RAPGEF1	Rap Guanine Nucleotide Exchange Factor 1	Protein Coding	43	GC09M131576	2.078895
UBA7	Ubiquitin Like Modifier Activating Enzyme 7	Protein Coding	43	GC03M049805	2.073261
CNOT3	CCR4-NOT Transcription Complex Subunit 3	Protein Coding	43	GC19P056416	2.066281
CUL2	Cullin 2	Protein Coding	43	GC10M035046	2.057254
KLK4	Kallikrein Related Peptidase 4	Protein Coding	43	GC19M050907	2.053793
ALOX15B	Arachidonate 15-Lipoxygenase Type B	Protein Coding	43	GC17P008039	2.052585
RPL9	Ribosomal Protein L9	Protein Coding	43	GC04M039452	2.047141
TNFRSF4	TNF Receptor Superfamily Member 4	Protein Coding	43	GC01M001211	2.034724
RNF2	Ring Finger Protein 2	Protein Coding	43	GC01P185045	2.030979
EFNB3	Ephrin B3	Protein Coding	43	GC17P009116	2.024564
CERK	Ceramide Kinase	Protein Coding	43	GC22M046684	2.022637
NR2E3	Nuclear Receptor Subfamily 2 Group E Member 3	Protein Coding	43	GC15P071792	2.016012
P2RY14	Purinergic Receptor P2Y14	Protein Coding	43	GC03M151212	2.013566
SAR1A	Secretion Associated Ras Related GTPase 1A	Protein Coding	43	GC10M070147	2.011923
HMMR	Hyaluronan Mediated Motility Receptor	Protein Coding	43	GC05P163480	2.009065
POMP	Proteasome Maturation Protein	Protein Coding	43	GC13P028659	2.007856
RTN4IP1	Reticulon 4 Interacting Protein 1	Protein Coding	43	GC06M106571	1.99959
APLP2	Amyloid Beta Precursor Like Protein 2	Protein Coding	43	GC11P130069	1.997367
RPL19	Ribosomal Protein L19	Protein Coding	43	GC17P039200	1.9968
NEK7	NIMA Related Kinase 7	Protein Coding	43	GC01P198156	1.993348
WWC1	WW And C2 Domain Containing 1	Protein Coding	43	GC05P168291	1.990625
EPB41L3	Erythrocyte Membrane Protein Band 4.1 Like 3	Protein Coding	43	GC18M005382	1.987793
CPT1C	Carnitine Palmitoyltransferase 1C	Protein Coding	43	GC19P049690	1.98776
TOPBP1	DNA Topoisomerase II Binding Protein 1	Protein Coding	43	GC03M133600	1.975582
FABP7	Fatty Acid Binding Protein 7	Protein Coding	43	GC06P122761	1.972409
ATP13A2	ATPase Cation Transporting 13A2	Protein Coding	43	GC01M016985	1.97237
PLS3	Plastin 3	Protein Coding	43	GC0XP115560	1.971734
S1PR4	Sphingosine-1-Phosphate Receptor 4	Protein Coding	43	GC19P003178	1.965928
MN1	MN1 Proto-Oncogene, Transcriptional Regulator	Protein Coding	43	GC22M027748	1.961891
RANGAP1	Ran GTPase Activating Protein 1	Protein Coding	43	GC22M041244	1.960875
LCP1	Lymphocyte Cytosolic Protein 1	Protein Coding	43	GC13M046132	1.960058
FUT6	Fucosyltransferase 6	Protein Coding	43	GC19M005830	1.952486
IFI16	Interferon Gamma Inducible Protein 16	Protein Coding	43	GC01P158969	1.952366
RPS5	Ribosomal Protein S5	Protein Coding	43	GC19P058386	1.949766
USH1C	USH1 Protein Network Component Harmonin	Protein Coding	43	GC11M017494	1.94318
CYTH1	Cytohesin 1	Protein Coding	43	GC17M078674	1.936759
GNA12	G Protein Subunit Alpha 12	Protein Coding	43	GC07M002728	1.934407
CHMP4B	Charged Multivesicular Body Protein 4B	Protein Coding	43	GC20P033843	1.930518
TUBA1B	Tubulin Alpha 1b	Protein Coding	43	GC12M049127	1.926763
PSMC1	Proteasome 26S Subunit, ATPase 1	Protein Coding	43	GC14P090256	1.92437
CPA6	Carboxypeptidase A6	Protein Coding	43	GC08M067422	1.922161
SLC24A4	Solute Carrier Family 24 Member 4	Protein Coding	43	GC14P092322	1.91529
SLC28A1	Solute Carrier Family 28 Member 1	Protein Coding	43	GC15P084884	1.909499
CBX4	Chromobox 4	Protein Coding	43	GC17M079833	1.905112
SNRPE	Small Nuclear Ribonucleoprotein Polypeptide E	Protein Coding	43	GC01P203877	1.903366
RASGRF1	Ras Protein Specific Guanine Nucleotide Releasing Factor 1	Protein Coding	43	GC15M078959	1.900143
TSG101	Tumor Susceptibility 101	Protein Coding	43	GC11M018468	1.895914
KAT7	Lysine Acetyltransferase 7	Protein Coding	43	GC17P049788	1.894569

JUND	JunD Proto-Oncogene, AP-1 Transcription Factor Subunit	Protein Coding	43	GC19M018279	1.892264
DLX3	Distal-Less Homeobox 3	Protein Coding	43	GC17M049990	1.891371
C7	Complement C7	Protein Coding	43	GC05P040909	1.888564
RNF5	Ring Finger Protein 5	Protein Coding	43	GC06P055228	1.885837
CCNG1	Cyclin G1	Protein Coding	43	GC05P163438	1.881374
PCBP1	Poly(RC) Binding Protein 1	Protein Coding	43	GC02P070087	1.870741
CLIC5	Chloride Intracellular Channel 5	Protein Coding	43	GC06M045880	1.870719
RPS13	Ribosomal Protein S13	Protein Coding	43	GC11M017274	1.868321
TEAD4	TEA Domain Transcription Factor 4	Protein Coding	43	GC12P002959	1.862686
NCS1	Neuronal Calcium Sensor 1	Protein Coding	43	GC09P130172	1.862391
DUT	Deoxyuridine Triphosphatase	Protein Coding	43	GC15P048331	1.849418
FAR1	Fatty Acyl-CoA Reductase 1	Protein Coding	43	GC11P013668	1.848052
POLK	DNA Polymerase Kappa	Protein Coding	43	GC05P075511	1.847873
ABCB8	ATP Binding Cassette Subfamily B Member 8	Protein Coding	43	GC07P151028	1.845093
FCER1A	Fc Fragment Of IgE Receptor Ia	Protein Coding	43	GC01P159259	1.840351
FBXO11	F-Box Protein 11	Protein Coding	43	GC02M047789	1.839876
RTN4R	Reticulon 4 Receptor	Protein Coding	43	GC22M020241	1.832302
GRIK4	Glutamate Ionotropic Receptor Kainate Type Subunit 4	Protein Coding	43	GC11P120512	1.825574
XPNPEP1	X-Prolyl Aminopeptidase 1	Protein Coding	43	GC10M109864	1.823427
HOXA10	Homeobox A10	Protein Coding	43	GC07M027346	1.823059
WWP1	WW Domain Containing E3 Ubiquitin Protein Ligase 1	Protein Coding	43	GC08P086342	1.820919
IL3RA	Interleukin 3 Receptor Subunit Alpha	Protein Coding	43	GC0XP001336	1.813977
JUNB	JunB Proto-Oncogene, AP-1 Transcription Factor Subunit	Protein Coding	43	GC19P012791	1.813843
TRIP12	Thyroid Hormone Receptor Interactor 12	Protein Coding	43	GC02M229763	1.805426
CAPNS1	Calpain Small Subunit 1	Protein Coding	43	GC19P040383	1.804797
B3GALNT1	Beta-1,3-N-Acetylgalactosaminyltransferase 1 (Globoside Blood Group)	Protein Coding	43	GC03M161083	1.801588
ESPL1	Extra Spindle Pole Bodies Like 1, Separase	Protein Coding	43	GC12P053268	1.795951
LGMN	Legumain	Protein Coding	43	GC14M092703	1.794493
RPL13	Ribosomal Protein L13	Protein Coding	43	GC16P089802	1.792763
MYO7A	Myosin VIIA	Protein Coding	43	GC11P077128	1.7863
EDA2R	Ectodysplasin A2 Receptor	Protein Coding	43	GC0XM066595	1.783005
OPHN1	Oligophrenin 1	Protein Coding	43	GC0XM067949	1.766493
CNGB1	Cyclic Nucleotide Gated Channel Subunit Beta 1	Protein Coding	43	GC16M057884	1.766347
SLC22A18	Solute Carrier Family 22 Member 18	Protein Coding	43	GC11P002899	1.76562
CLDN10	Claudin 10	Protein Coding	43	GC13P095433	1.762966
EEF1D	Eukaryotic Translation Elongation Factor 1 Delta	Protein Coding	43	GC08M143579	1.743176
NFKBIB	NFKB Inhibitor Beta	Protein Coding	43	GC19P038899	1.736425
ENTPD6	Ectonucleoside Triphosphate Diphosphohydrolase 6	Protein Coding	43	GC20P025196	1.731347
TNFRSF18	TNF Receptor Superfamily Member 18	Protein Coding	43	GC01M001203	1.73019
BRSK1	BR Serine/Threonine Kinase 1	Protein Coding	43	GC19P056473	1.727919
E2F2	E2F Transcription Factor 2	Protein Coding	43	GC01M023541	1.726436
WNT6	Wnt Family Member 6	Protein Coding	43	GC02P218859	1.723181
SLC13A3	Solute Carrier Family 13 Member 3	Protein Coding	43	GC20M046557	1.72313
RAD17	RAD17 Checkpoint Clamp Loader Component	Protein Coding	43	GC05P069369	1.716172
MNAT1	MNAT1 Component Of CDK Activating Kinase	Protein Coding	43	GC14P060734	1.715957
ACP2	Acid Phosphatase 2, Lysosomal	Protein Coding	43	GC11M068994	1.709731
AREG	Amphiregulin	Protein Coding	43	GC04P074445	1.709488
CAPN9	Calpain 9	Protein Coding	43	GC01P230747	1.706426
MT2A	Metallothionein 2A	Protein Coding	43	GC16P056658	1.703984
SEMA7A	Semaphorin 7A (John Milton Hagen Blood Group)	Protein Coding	43	GC15M074409	1.702596
GALNT14	Polypeptide N-Acetylgalactosaminyltransferase 14	Protein Coding	43	GC02M030888	1.700862
KHSRP	KH-Type Splicing Regulatory Protein	Protein Coding	43	GC19M006413	1.699285
RNF216	Ring Finger Protein 216	Protein Coding	43	GC07M005620	1.694081

LIPH	Lipase H	Protein Coding	43	GC03M185506	1.686943
COL8A2	Collagen Type VIII Alpha 2 Chain	Protein Coding	43	GC01M036095	1.68344
PRPF31	Pre-MRNA Processing Factor 31	Protein Coding	43	GC19P056418	1.681831
SENP1	SUMO Specific Peptidase 1	Protein Coding	43	GC12M048042	1.678272
ERLIN1	ER Lipid Raft Associated 1	Protein Coding	43	GC10M100150	1.668031
PCM1	Pericentriolar Material 1	Protein Coding	43	GC08P017922	1.664333
SMURF2	SMAD Specific E3 Ubiquitin Protein Ligase 2	Protein Coding	43	GC17M064542	1.66336
MPC1	Mitochondrial Pyruvate Carrier 1	Protein Coding	43	GC06M166364	1.653977
BAG1	BAG Cochaperone 1	Protein Coding	43	GC09M033245	1.653213
RAP1GDS1	Rap1 GTPase-GDP Dissociation Stimulator 1	Protein Coding	43	GC04P098261	1.649948
ACTR3	Actin Related Protein 3	Protein Coding	43	GC02P113889	1.648223
UBQLN1	Ubiquilin 1	Protein Coding	43	GC09M083659	1.646947
CEACAM1	CEA Cell Adhesion Molecule 1	Protein Coding	43	GC19M042507	1.646535
RPL10A	Ribosomal Protein L10a	Protein Coding	43	GC06P055328	1.643943
FKBP8	FKBP Prolyl Isomerase 8	Protein Coding	43	GC19M018503	1.641717
DNTT	DNA Nucleotidylexotransferase	Protein Coding	43	GC10P096304	1.641584
EFNA4	Ephrin A4	Protein Coding	43	GC01P155063	1.639747
RBL1	RB Transcriptional Corepressor Like 1	Protein Coding	43	GC20M036996	1.638759
EEF1B2	Eukaryotic Translation Elongation Factor 1 Beta 2	Protein Coding	43	GC02P206159	1.636693
HIP1	Huntingtin Interacting Protein 1	Protein Coding	43	GC07M075533	1.630816
BCL9	BCL9 Transcription Coactivator	Protein Coding	43	GC01P147541	1.6295
PUM1	Pumilio RNA Binding Family Member 1	Protein Coding	43	GC01M030931	1.627818
SLC31A1	Solute Carrier Family 31 Member 1	Protein Coding	43	GC09P113221	1.625954
NEFM	Neurofilament Medium Chain	Protein Coding	43	GC08P024913	1.622574
VPS4A	Vacuolar Protein Sorting 4 Homolog A	Protein Coding	43	GC16P069311	1.622356
MBD4	Methyl-CpG Binding Domain 4, DNA Glycosylase	Protein Coding	43	GC03M129430	1.614065
TARS2	Threonyl-TRNA Synthetase 2, Mitochondrial	Protein Coding	43	GC01P150502	1.611859
P2RY13	Purinergic Receptor P2Y13	Protein Coding	43	GC03M151326	1.603261
RPL4	Ribosomal Protein L4	Protein Coding	43	GC15M066498	1.601163
ADAM22	ADAM Metallopeptidase Domain 22	Protein Coding	43	GC07P087934	1.590823
METTL1	Methyltransferase Like 1	Protein Coding	43	GC12M057768	1.587707
LY96	Lymphocyte Antigen 96	Protein Coding	43	GC08P073991	1.584349
UBE2E3	Ubiquitin Conjugating Enzyme E2 E3	Protein Coding	43	GC02P180967	1.581456
VAMP3	Vesicle Associated Membrane Protein 3	Protein Coding	43	GC01P007765	1.579937
CIC	Capicua Transcriptional Repressor	Protein Coding	43	GC19P042268	1.57902
BTAF1	B-TFIID TATA-Box Binding Protein Associated Factor 1	Protein Coding	43	GC10P091923	1.574021
PAFAH1B3	Platelet Activating Factor Acetylhydrolase 1b Catalytic Subunit 3	Protein Coding	43	GC19M042297	1.569415
NAE1	NEDD8 Activating Enzyme E1 Subunit 1	Protein Coding	43	GC16M066803	1.562533
SLC3A2	Solute Carrier Family 3 Member 2	Protein Coding	43	GC11P062856	1.562437
ADH1A	Alcohol Dehydrogenase 1A (Class I), Alpha Polypeptide	Protein Coding	43	GC04M099276	1.562178
PSME3	Proteasome Activator Subunit 3	Protein Coding	43	GC17P042824	1.551885
GIPC1	GIPC PDZ Domain Containing Family Member 1	Protein Coding	43	GC19M014450	1.545193
PSMC3	Proteasome 26S Subunit, ATPase 3	Protein Coding	43	GC11M069001	1.535386
RHOT1	Ras Homolog Family Member T1	Protein Coding	43	GC17P032142	1.529621
ACVR1C	Activin A Receptor Type 1C	Protein Coding	43	GC02M157526	1.52746
MINK1	Misshapen Like Kinase 1	Protein Coding	43	GC17P004833	1.524693
GZMA	Granzyme A	Protein Coding	43	GC05P055102	1.523474
FPGS	Folylpolyglutamate Synthase	Protein Coding	43	GC09P127794	1.512443
FOXO4	Forkhead Box O4	Protein Coding	43	GC0XP071095	1.509242
SLC6A11	Solute Carrier Family 6 Member 11	Protein Coding	43	GC03P011027	1.508807
GCAT	Glycine C-Acetyltransferase	Protein Coding	43	GC22P037807	1.502766
KPNA3	Karyopherin Subunit Alpha 3	Protein Coding	43	GC13M049699	1.499147
PDCD4	Programmed Cell Death 4	Protein Coding	43	GC10P110871	1.498506

SLC22A7	Solute Carrier Family 22 Member 7	Protein Coding	43	GC06P055408	1.496314
RAB33B	RAB33B, Member RAS Oncogene Family	Protein Coding	43	GC04P139453	1.493689
PSMB2	Proteasome 20S Subunit Beta 2	Protein Coding	43	GC01M035599	1.474161
EDC3	Enhancer Of MRNA Decapping 3	Protein Coding	43	GC15M074631	1.469529
YME1L1	YME1 Like 1 ATPase	Protein Coding	43	GC10M027110	1.468679
EIF3A	Eukaryotic Translation Initiation Factor 3 Subunit A	Protein Coding	43	GC10M119034	1.462634
WNT9A	Wnt Family Member 9A	Protein Coding	43	GC01M227920	1.457425
RYK	Receptor Like Tyrosine Kinase	Protein Coding	43	GC03M134065	1.454817
FZR1	Fizzy And Cell Division Cycle 20 Related 1	Protein Coding	43	GC19P003506	1.448471
SLC22A8	Solute Carrier Family 22 Member 8	Protein Coding	43	GC11M069279	1.443051
BNIP3L	BCL2 Interacting Protein 3 Like	Protein Coding	43	GC08P026296	1.442665
VTI1B	Vesicle Transport Through Interaction With T-SNAREs 1B	Protein Coding	43	GC14M067647	1.441342
ABCB5	ATP Binding Cassette Subfamily B Member 5	Protein Coding	43	GC07P020615	1.438698
ZFYVE9	Zinc Finger FYVE-Type Containing 9	Protein Coding	43	GC01P052142	1.437387
IYD	Iodotyrosine Deiodinase	Protein Coding	43	GC06P150368	1.43686
SEPHS1	Selenophosphate Synthetase 1	Protein Coding	43	GC10M013317	1.435249
DGKQ	Diacylglycerol Kinase Theta	Protein Coding	43	GC04M000942	1.418169
KCNJ15	Potassium Inwardly Rectifying Channel Subfamily J Member 15	Protein Coding	43	GC21P038157	1.414762
SHANK2	SH3 And Multiple Ankyrin Repeat Domains 2	Protein Coding	43	GC11M070467	1.40972
TST	Thiosulfate Sulfurtransferase	Protein Coding	43	GC22M037010	1.408611
CETN2	Centrin 2	Protein Coding	43	GC0XM152827	1.408595
GSTA1	Glutathione S-Transferase Alpha 1	Protein Coding	43	GC06M052791	1.398104
DCTD	DCMP Deaminase	Protein Coding	43	GC04M182890	1.397598
GABRG3	Gamma-Aminobutyric Acid Type A Receptor Subunit Gamma3	Protein Coding	43	GC15P026971	1.38661
DFFB	DNA Fragmentation Factor Subunit Beta	Protein Coding	43	GC01P003797	1.375411
PTPN5	Protein Tyrosine Phosphatase Non-Receptor Type 5	Protein Coding	43	GC11M018727	1.366596
SND1	Staphylococcal Nuclease And Tudor Domain Containing 1	Protein Coding	43	GC07P127652	1.365154
PAICS	Phosphoribosylaminoimidazole Carboxylase And Phosphoribosylaminoimidazolesuccinocarboxamide Synthase	Protein Coding	43	GC04P056410	1.359472
PDIA4	Protein Disulfide Isomerase Family A Member 4	Protein Coding	43	GC07M149003	1.35273
MC5R	Melanocortin 5 Receptor	Protein Coding	43	GC18P013824	1.351181
MCF2L	MCF.2 Cell Line Derived Transforming Sequence Like	Protein Coding	43	GC13P112894	1.349725
PSME2	Proteasome Activator Subunit 2	Protein Coding	43	GC14M024143	1.343981
P2RX6	Purinergic Receptor P2X 6	Protein Coding	43	GC22P021183	1.342427
ARFGAP1	ADP Ribosylation Factor GTPase Activating Protein 1	Protein Coding	43	GC20P063272	1.337968
DDX20	DEAD-Box Helicase 20	Protein Coding	43	GC01P111755	1.331783
SCNN1D	Sodium Channel Epithelial 1 Subunit Delta	Protein Coding	43	GC01P001280	1.33102
SNX5	Sorting Nexin 5	Protein Coding	43	GC20M017989	1.330691
LCP2	Lymphocyte Cytosolic Protein 2	Protein Coding	43	GC05M170246	1.328276
MFN1	Mitofusin 1	Protein Coding	43	GC03P179347	1.328005
RPS12	Ribosomal Protein S12	Protein Coding	43	GC06P132814	1.320846
PRKRA	Protein Activator Of Interferon Induced Protein Kinase EIF2AK2	Protein Coding	43	GC02M178431	1.320386
SULT1A1	Sulfotransferase Family 1A Member 1	Protein Coding	43	GC16M028606	1.319872
EDEM1	ER Degradation Enhancing Alpha-Mannosidase Like Protein 1	Protein Coding	43	GC03P005187	1.319427
LGI1	Leucine Rich Glioma Inactivated 1	Protein Coding	43	GC10P093757	1.297507
NEK3	NIMA Related Kinase 3	Protein Coding	43	GC13M052132	1.297166
LASP1	LIM And SH3 Protein 1	Protein Coding	43	GC17P038869	1.28668
UCHL5	Ubiquitin C-Terminal Hydrolase L5	Protein Coding	43	GC01M193012	1.281788
CHIA	Chitinase Acidic	Protein Coding	43	GC01P111291	1.281257
GLUD2	Glutamate Dehydrogenase 2	Protein Coding	43	GC0XP121047	1.281094
RAB1A	RAB1A, Member RAS Oncogene Family	Protein Coding	43	GC02M065072	1.277939
HAGH	Hydroxyacylglutathione Hydrolase	Protein Coding	43	GC16M001795	1.276918
HCLS1	Hematopoietic Cell-Specific Lyn Substrate 1	Protein Coding	43	GC03M121631	1.271911
TPP2	Tripeptidyl Peptidase 2	Protein Coding	43	GC13P102596	1.25713

COPS5	COP9 Signalosome Subunit 5	Protein Coding	43	GC08M067043	1.244869
EIF5	Eukaryotic Translation Initiation Factor 5	Protein Coding	43	GC14P103333	1.239389
STAM	Signal Transducing Adaptor Molecule	Protein Coding	43	GC10P017651	1.238333
RGR	Retinal G Protein Coupled Receptor	Protein Coding	43	GC10P085402	1.23532
RACGAP1	Rac GTPase Activating Protein 1	Protein Coding	43	GC12M049978	1.229995
STX4	Syntaxin 4	Protein Coding	43	GC16P032515	1.227676
ARPC2	Actin Related Protein 2/3 Complex Subunit 2	Protein Coding	43	GC02P218217	1.225886
SLC38A2	Solute Carrier Family 38 Member 2	Protein Coding	43	GC12M046358	1.217816
RPS9	Ribosomal Protein S9	Protein Coding	43	GC19P056806	1.214077
GPR50	G Protein-Coupled Receptor 50	Protein Coding	43	GC0XP151176	1.213
STK17B	Serine/Threonine Kinase 17b	Protein Coding	43	GC02M196133	1.212577
SSTR4	Somatostatin Receptor 4	Protein Coding	43	GC20P023035	1.199625
ATP6V1G3	ATPase H+ Transporting V1 Subunit G3	Protein Coding	43	GC01M198492	1.198682
FOSB	FosB Proto-Oncogene, AP-1 Transcription Factor Subunit	Protein Coding	43	GC19P045467	1.196872
MPST	Mercaptopyruvate Sulfurtransferase	Protein Coding	43	GC22P037019	1.192437
KIF2A	Kinesin Family Member 2A	Protein Coding	43	GC05P062306	1.187315
AIPL1	Aryl Hydrocarbon Receptor Interacting Protein Like 1	Protein Coding	43	GC17M006393	1.175994
AK3	Adenylate Kinase 3	Protein Coding	43	GC09M004703	1.163288
POU3F2	POU Class 3 Homeobox 2	Protein Coding	43	GC06P098834	1.156319
S100A11	S100 Calcium Binding Protein A11	Protein Coding	43	GC01M152032	1.152702
DHTKD1	Dehydrogenase E1 And Transketolase Domain Containing 1	Protein Coding	43	GC10P012068	1.139075
RAB11B	RAB11B, Member RAS Oncogene Family	Protein Coding	43	GC19P008393	1.127774
TARBP2	TARBP2 Subunit Of RISC Loading Complex	Protein Coding	43	GC12P053499	1.12641
MBD3	Methyl-CpG Binding Domain Protein 3	Protein Coding	43	GC19M002160	1.125607
QPRT	Quinolinate Phosphoribosyltransferase	Protein Coding	43	GC16P032366	1.125043
CRYBA4	Crystallin Beta A4	Protein Coding	43	GC22P026881	1.115191
SLC25A10	Solute Carrier Family 25 Member 10	Protein Coding	43	GC17P081712	1.112718
MR1	Major Histocompatibility Complex, Class I-Related	Protein Coding	43	GC01P181033	1.111144
UBE2V1	Ubiquitin Conjugating Enzyme E2 V1	Protein Coding	43	GC20M050082	1.100244
BST1	Bone Marrow Stromal Cell Antigen 1	Protein Coding	43	GC04P015704	1.098644
LILRB1	Leukocyte Immunoglobulin Like Receptor B1	Protein Coding	43	GC19P056438	1.097496
UAP1	UDP-N-Acetylglucosamine Pyrophosphorylase 1	Protein Coding	43	GC01P162561	1.096617
ADAM8	ADAM Metallopeptidase Domain 8	Protein Coding	43	GC10M133262	1.092386
GLS2	Glutaminase 2	Protein Coding	43	GC12M056470	1.091968
NME4	NME/NM23 Nucleoside Diphosphate Kinase 4	Protein Coding	43	GC16P000396	1.090327
VPS29	VPS29 Retromer Complex Component	Protein Coding	43	GC12M110491	1.084897
PSMD3	Proteasome 26S Subunit, Non-ATPase 3	Protein Coding	43	GC17P039980	1.084389
MTA1	Metastasis Associated 1	Protein Coding	43	GC14P105419	1.076727
ANP32A	Acidic Nuclear Phosphoprotein 32 Family Member A	Protein Coding	43	GC15M068778	1.07578
TPSAB1	Tryptase Alpha/Beta 1	Protein Coding	43	GC16P001240	1.07458
CRTC1	CREB Regulated Transcription Coactivator 1	Protein Coding	43	GC19P026660	1.072927
PSMD11	Proteasome 26S Subunit, Non-ATPase 11	Protein Coding	43	GC17P032444	1.068819
PCYT2	Phosphate Cytidylyltransferase 2, Ethanolamine	Protein Coding	43	GC17M081900	1.06538
KCNC4	Potassium Voltage-Gated Channel Subfamily C Member 4	Protein Coding	43	GC01P110211	1.065301
RAD54B	RAD54 Homolog B	Protein Coding	43	GC08M094371	1.064282
EPHA6	EPH Receptor A6	Protein Coding	43	GC03P096814	1.053914
CCNK	Cyclin K	Protein Coding	43	GC14P099483	1.044312
KEL	Kell Metallo-Endopeptidase (Kell Blood Group)	Protein Coding	43	GC07M142964	1.040401
CLTA	Clathrin Light Chain A	Protein Coding	43	GC09P036190	1.038781
OTX1	Orthodenticle Homeobox 1	Protein Coding	43	GC02P063050	1.035994
DGKI	Diacylglycerol Kinase Iota	Protein Coding	43	GC07M137381	1.035748
CYP4F12	Cytochrome P450 Family 4 Subfamily F Member 12	Protein Coding	43	GC19P015672	1.028058
MGST1	Microsomal Glutathione S-Transferase 1	Protein Coding	43	GC12P016347	1.026894

UBE2V2	Ubiquitin Conjugating Enzyme E2 V2	Protein Coding	43	GC08P047998	1.019955
KRT20	Keratin 20	Protein Coding	43	GC17M040875	1.017225
TNFRSF10D	TNF Receptor Superfamily Member 10d	Protein Coding	43	GC08M023135	1.002628
MCAT	Malonyl-CoA-Acyl Carrier Protein Transacylase	Protein Coding	43	GC22M043132	0.999035
USP11	Ubiquitin Specific Peptidase 11	Protein Coding	43	GC0XP047232	0.996571
ASRGL1	Asparaginase And Isoaspartyl Peptidase 1	Protein Coding	43	GC11P062337	0.995931
CCNE2	Cyclin E2	Protein Coding	43	GC08M094879	0.992559
CPB1	Carboxypeptidase B1	Protein Coding	43	GC03P148791	0.985945
AP1S2	Adaptor Related Protein Complex 1 Subunit Sigma 2	Protein Coding	43	GC0XM015825	0.98586
TNK1	Tyrosine Kinase Non Receptor 1	Protein Coding	43	GC17P007380	0.985678
POLR2D	RNA Polymerase II Subunit D	Protein Coding	43	GC02M128042	0.973725
SUMO3	Small Ubiquitin Like Modifier 3	Protein Coding	43	GC21M044805	0.970779
BMP3	Bone Morphogenetic Protein 3	Protein Coding	43	GC04P081030	0.965265
SOCS5	Suppressor Of Cytokine Signaling 5	Protein Coding	43	GC02P046698	0.962427
RPN2	Ribophorin II	Protein Coding	43	GC20P037178	0.958641
PARD6A	Par-6 Family Cell Polarity Regulator Alpha	Protein Coding	43	GC16P067661	0.953706
PGC	Progastricsin	Protein Coding	43	GC06M041736	0.952451
MAGED1	MAGE Family Member D1	Protein Coding	43	GC0XP051803	0.94959
KLK15	Kallikrein Related Peptidase 15	Protein Coding	43	GC19M050825	0.948931
MAP3K13	Mitogen-Activated Protein Kinase Kinase Kinase 13	Protein Coding	43	GC03P185282	0.948356
IKZF3	IKAROS Family Zinc Finger 3	Protein Coding	43	GC17M039759	0.944929
CLTB	Clathrin Light Chain B	Protein Coding	43	GC05M176392	0.932664
PMP2	Peripheral Myelin Protein 2	Protein Coding	43	GC08M081440	0.919918
CEBPE	CCAAT Enhancer Binding Protein Epsilon	Protein Coding	43	GC14M023117	0.913879
SLC6A13	Solute Carrier Family 6 Member 13	Protein Coding	43	GC12M000200	0.908939
IL24	Interleukin 24	Protein Coding	43	GC01P206897	0.892902
TOPORS	TOP1 Binding Arginine/Serine Rich Protein, E3 Ubiquitin Ligase	Protein Coding	43	GC09M032540	0.89177
HTR5A	5-Hydroxytryptamine Receptor 5A	Protein Coding	43	GC07P155070	0.891023
PFKFB1	6-Phosphofructo-2-Kinase/Fructose-2,6-Biphosphatase 1	Protein Coding	43	GC0XM054932	0.890548
CKS1B	CDC28 Protein Kinase Regulatory Subunit 1B	Protein Coding	43	GC01P154974	0.888721
SLC27A5	Solute Carrier Family 27 Member 5	Protein Coding	43	GC19M058479	0.887378
PLCD4	Phospholipase C Delta 4	Protein Coding	43	GC02P218608	0.88713
FOXA1	Forkhead Box A1	Protein Coding	43	GC14M037589	0.886432
GPR143	G Protein-Coupled Receptor 143	Protein Coding	43	GC0XM009725	0.886136
RPA3	Replication Protein A3	Protein Coding	43	GC07M007637	0.876291
ABI1	Abl Interactor 1	Protein Coding	43	GC10M026746	0.876246
KLK6	Kallikrein Related Peptidase 6	Protein Coding	43	GC19M050958	0.875737
TREH	Trehalase	Protein Coding	43	GC11M118657	0.868183
COCH	Cochlin	Protein Coding	43	GC14P030874	0.851058
PIAS4	Protein Inhibitor Of Activated STAT 4	Protein Coding	43	GC19P004007	0.848433
PTGR1	Prostaglandin Reductase 1	Protein Coding	43	GC09M111549	0.842943
SLC28A2	Solute Carrier Family 28 Member 2	Protein Coding	43	GC15P045252	0.839309
BRSK2	BR Serine/Threonine Kinase 2	Protein Coding	43	GC11P001389	0.838175
GABRR1	Gamma-Aminobutyric Acid Type A Receptor Subunit Rho1	Protein Coding	43	GC06M089177	0.829656
PLA2G2D	Phospholipase A2 Group IID	Protein Coding	43	GC01M020111	0.827045
RBMX	RNA Binding Motif Protein X-Linked	Protein Coding	43	GC0XM136848	0.82128
IQGAP2	IQ Motif Containing GTPase Activating Protein 2	Protein Coding	43	GC05P076403	0.82013
GRK1	G Protein-Coupled Receptor Kinase 1	Protein Coding	43	GC13P113645	0.808675
APBA2	Amyloid Beta Precursor Protein Binding Family A Member 2	Protein Coding	43	GC15P029286	0.805343
CHRNA9	Cholinergic Receptor Nicotinic Alpha 9 Subunit	Protein Coding	43	GC04P040337	0.783881
POLR2J	RNA Polymerase II Subunit J	Protein Coding	43	GC07M102473	0.781176
LSP1	Lymphocyte Specific Protein 1	Protein Coding	43	GC11P001852	0.778513
GTF2B	General Transcription Factor IIB	Protein Coding	43	GC01M088853	0.735013

PRMT3	Protein Arginine Methyltransferase 3	Protein Coding	43	GC11P020409	0.713376
SERPINB3	Serpin Family B Member 3	Protein Coding	43	GC18M063655	0.7082
IL31RA	Interleukin 31 Receptor A	Protein Coding	43	GC05P055840	0.702177
CA6	Carbonic Anhydrase 6	Protein Coding	43	GC01P008945	0.689711
POLR2C	RNA Polymerase II Subunit C	Protein Coding	43	GC16P057462	0.686421
GRPR	Gastrin Releasing Peptide Receptor	Protein Coding	43	GC0XP016141	0.674788
CTSE	Cathepsin E	Protein Coding	43	GC01M206009	0.660449
PSMB6	Proteasome 20S Subunit Beta 6	Protein Coding	43	GC17P004796	0.650218
RPE	Ribulose-5-Phosphate-3-Epimerase	Protein Coding	43	GC02P210002	0.645324
OLA1	Obg Like ATPase 1	Protein Coding	43	GC02M174072	0.638848
MYBL2	MYB Proto-Oncogene Like 2	Protein Coding	43	GC20P043667	0.62005
CHRNA6	Cholinergic Receptor Nicotinic Alpha 6 Subunit	Protein Coding	43	GC08M042752	0.618801
RAB5B	RAB5B, Member RAS Oncogene Family	Protein Coding	43	GC12P055973	0.608642
CYP2F1	Cytochrome P450 Family 2 Subfamily F Member 1	Protein Coding	43	GC19P041114	0.555041
INSRR	Insulin Receptor Related Receptor	Protein Coding	43	GC01M156840	0.542826
DOK2	Docking Protein 2	Protein Coding	43	GC08M021908	0.488359
SLC5A8	Solute Carrier Family 5 Member 8	Protein Coding	43	GC12M101155	0.457159
RHCG	Rh Family C Glycoprotein	Protein Coding	43	GC15M089471	0.401535
PSME1	Proteasome Activator Subunit 1	Protein Coding	43	GC14P024136	0.325914
ALDH3B1	Aldehyde Dehydrogenase 3 Family Member B1	Protein Coding	43	GC11P068027	0.272315
SLURP1	Secreted LY6/PLAUR Domain Containing 1	Protein Coding	43	GC08M142740	0.272315
TBX4	T-Box Transcription Factor 4	Protein Coding	42	GC17P061451	30.10983
LDB3	LIM Domain Binding 3	Protein Coding	42	GC10P086666	29.65737
RERE	Arginine-Glutamic Acid Dipeptide Repeats	Protein Coding	42	GC01M008364	24.83842
INPP5E	Inositol Polyphosphate-5-Phosphatase E	Protein Coding	42	GC09M136428	23.89851
KCNE2	Potassium Voltage-Gated Channel Subfamily E Regulatory Subunit 2	Protein Coding	42	GC21P034364	22.21654
LPA	Lipoprotein(A)	Protein Coding	42	GC06M160531	22.05887
PPA2	Inorganic Pyrophosphatase 2	Protein Coding	42	GC04M105369	19.56586
CXCL8	C-X-C Motif Chemokine Ligand 8	Protein Coding	42	GC04P073740	19.49537
CDK13	Cyclin Dependent Kinase 13	Protein Coding	42	GC07P040149	18.88773
MYOCD	Myocardin	Protein Coding	42	GC17P012665	18.88748
TINF2	TERF1 Interacting Nuclear Factor 2	Protein Coding	42	GC14M024234	18.26061
MYOT	Myotilin	Protein Coding	42	GC05P137867	17.48589
IGHMBP2	Immunoglobulin Mu DNA Binding Protein 2	Protein Coding	42	GC11P068903	16.42241
PLCZ1	Phospholipase C Zeta 1	Protein Coding	42	GC12M018604	15.93719
AFF4	AF4/FMR2 Family Member 4	Protein Coding	42	GC05M132875	15.78778
ALMS1	ALMS1 Centrosome And Basal Body Associated Protein	Protein Coding	42	GC02P073385	15.6395
DNAJC19	DnaJ Heat Shock Protein Family (Hsp40) Member C19	Protein Coding	42	GC03M180983	15.52757
KMT2D	Lysine Methyltransferase 2D	Protein Coding	42	GC12M049018	15.13841
SRF	Serum Response Factor	Protein Coding	42	GC06P043171	14.77978
SHOC2	SHOC2 Leucine Rich Repeat Scaffold Protein	Protein Coding	42	GC10P110919	14.61045
TRIM37	Tripartite Motif Containing 37	Protein Coding	42	GC17M058982	14.53855
DNAH11	Dynein Axonemal Heavy Chain 11	Protein Coding	42	GC07P021543	14.2015
POLG2	DNA Polymerase Gamma 2, Accessory Subunit	Protein Coding	42	GC17M064477	13.48728
BBS2	Bardet-Biedl Syndrome 2	Protein Coding	42	GC16M056467	12.87107
PF4	Platelet Factor 4	Protein Coding	42	GC04M073980	12.7009
TBX18	T-Box Transcription Factor 18	Protein Coding	42	GC06M084666	12.601
MPV17	Mitochondrial Inner Membrane Protein MPV17	Protein Coding	42	GC02M027309	12.21983
AHI1	Abelson Helper Integration Site 1	Protein Coding	42	GC06M135283	11.97301
TK2	Thymidine Kinase 2	Protein Coding	42	GC16M066508	11.92556
FLCN	Folliculin	Protein Coding	42	GC17M017206	11.86707
PICK1	Protein Interacting With PRKCA 1	Protein Coding	42	GC22P038056	11.61791
NEK8	NIMA Related Kinase 8	Protein Coding	42	GC17P028725	11.32855

NPHS2	NPHS2 Stomatin Family Member, Podocin	Protein Coding	42	GC01M179519	11.25805
TDGF1	Teratocarcinoma-Derived Growth Factor 1	Protein Coding	42	GC03P046576	11.11709
SLC26A8	Solute Carrier Family 26 Member 8	Protein Coding	42	GC06M047031	11.10981
MADD	MAP Kinase Activating Death Domain	Protein Coding	42	GC11P047290	10.94644
KMT2C	Lysine Methyltransferase 2C	Protein Coding	42	GC07M152134	10.8063
SOX11	SRY-Box Transcription Factor 11	Protein Coding	42	GC02P005703	10.58785
ALG9	ALG9 Alpha-1,2-Mannosyltransferase	Protein Coding	42	GC11M111979	10.43029
PREPL	Prolyl Endopeptidase Like	Protein Coding	42	GC02M044281	10.40052
MTFMT	Mitochondrial Methionyl-TRNA Formyltransferase	Protein Coding	42	GC15M065001	10.31567
HAVCR1	Hepatitis A Virus Cellular Receptor 1	Protein Coding	42	GC05M157028	9.892607
LGALS2	Galectin 2	Protein Coding	42	GC22M037570	9.885687
NDUFAF1	NADH:Ubiquinone Oxidoreductase Complex Assembly Factor 1	Protein Coding	42	GC15M041387	9.738609
LTBP3	Latent Transforming Growth Factor Beta Binding Protein 3	Protein Coding	42	GC11M065538	9.50705
BBS4	Bardet-Biedl Syndrome 4	Protein Coding	42	GC15P072686	9.451375
HCCS	Holocytochrome C Synthase	Protein Coding	42	GC0XP011111	9.367452
IQCB1	IQ Motif Containing B1	Protein Coding	42	GC03M121769	9.169977
GOPC	Golgi Associated PDZ And Coiled-Coil Motif Containing	Protein Coding	42	GC06M117560	9.115255
CFHR1	Complement Factor H Related 1	Protein Coding	42	GC01P196822	9.107987
ZFHX3	Zinc Finger Homeobox 3	Protein Coding	42	GC16M072782	9.047967
PROKR2	Prokineticin Receptor 2	Protein Coding	42	GC20M005325	9.043825
ARID2	AT-Rich Interaction Domain 2	Protein Coding	42	GC12P045729	8.678627
SMN1	Survival Of Motor Neuron 1, Telomeric	Protein Coding	42	GC05P070924	8.556351
VPS33B	VPS33B Late Endosome And Lysosome Associated	Protein Coding	42	GC15M090998	8.434881
IFNB1	Interferon Beta 1	Protein Coding	42	GC09M021077	8.289925
S100A1	S100 Calcium Binding Protein A1	Protein Coding	42	GC01P153627	8.239047
C9orf72	C9orf72-SMCR8 Complex Subunit	Protein Coding	42	GC09M027588	7.891469
IARS2	Isoleucyl-TRNA Synthetase 2, Mitochondrial	Protein Coding	42	GC01P220094	7.801478
TRAF7	TNF Receptor Associated Factor 7	Protein Coding	42	GC16P005588	7.702885
ADAMTS10	ADAM Metallopeptidase With Thrombospondin Type 1 Motif 10	Protein Coding	42	GC19M008580	7.701241
SPEG	Striated Muscle Enriched Protein Kinase	Protein Coding	42	GC02P219434	7.700571
ADAMTSL1	ADAMTS Like 1	Protein Coding	42	GC09P017906	7.679827
RAMP2	Receptor Activity Modifying Protein 2	Protein Coding	42	GC17P042758	7.521454
SDHAF2	Succinate Dehydrogenase Complex Assembly Factor 2	Protein Coding	42	GC11P061430	7.504877
PTF1A	Pancreas Associated Transcription Factor 1a	Protein Coding	42	GC10P023194	7.457473
DGCR8	DGCR8 Microprocessor Complex Subunit	Protein Coding	42	GC22P020080	7.361435
MFAP5	Microfibril Associated Protein 5	Protein Coding	42	GC12M008637	7.336842
RPS29	Ribosomal Protein S29	Protein Coding	42	GC14M049570	7.16817
COQ9	Coenzyme Q9	Protein Coding	42	GC16P057447	7.066826
NPC2	NPC Intracellular Cholesterol Transporter 2	Protein Coding	42	GC14M074476	7.065322
ARFGEF2	ADP Ribosylation Factor Guanine Nucleotide Exchange Factor 2	Protein Coding	42	GC20P048921	7.060148
CHKA	Choline Kinase Alpha	Protein Coding	42	GC11M068052	7.03777
ALG2	ALG2 Alpha-1,3/1,6-Mannosyltransferase	Protein Coding	42	GC09M099216	6.888496
ROBO4	Roundabout Guidance Receptor 4	Protein Coding	42	GC11M124883	6.869812
DPF2	Double PHD Fingers 2	Protein Coding	42	GC11P065351	6.80849
PCNT	Pericentrin	Protein Coding	42	GC21P046324	6.792992
HOXD13	Homeobox D13	Protein Coding	42	GC02P176092	6.7721
CATSPER2	Cation Channel Sperm Associated 2	Protein Coding	42	GC15M043628	6.748589
CHST14	Carbohydrate Sulfotransferase 14	Protein Coding	42	GC15P040470	6.739023
DNAL1	Dynein Axonemal Light Chain 1	Protein Coding	42	GC14P073644	6.715905
CEP57	Centrosomal Protein 57	Protein Coding	42	GC11P095789	6.633479
UQCRQ	Ubiquinol-Cytochrome C Reductase Complex III Subunit VII	Protein Coding	42	GC05P132866	6.603085
EDAR	Ectodysplasin A Receptor	Protein Coding	42	GC02M108894	6.554439
MRPS22	Mitochondrial Ribosomal Protein S22	Protein Coding	42	GC03P139005	6.496555

CCNO	Cyclin O	Protein Coding	42	GC05M055231	6.268296
MUC5B	Mucin 5B, Oligomeric Mucus/Gel-Forming	Protein Coding	42	GC11P001244	6.246887
TOP3A	DNA Topoisomerase III Alpha	Protein Coding	42	GC17M018271	6.141516
KCNAB1	Potassium Voltage-Gated Channel Subfamily A Member Regulatory Beta Subunit 1	Protein Coding	42	GC03P156037	6.121867
CELF2	CUGBP Elav-Like Family Member 2	Protein Coding	42	GC10P010462	5.932603
MGAM	Maltase-Glucoamylase	Protein Coding	42	GC07P145925	5.920896
DMRT1	Doublesex And Mab-3 Related Transcription Factor 1	Protein Coding	42	GC09P000831	5.915781
FCN2	Ficolin 2	Protein Coding	42	GC09P134864	5.837862
DNA2	DNA Replication Helicase/Nuclease 2	Protein Coding	42	GC10M068414	5.823908
TBR1	T-Box Brain Transcription Factor 1	Protein Coding	42	GC02P161416	5.731599
FAM20C	FAM20C Golgi Associated Secretory Pathway Kinase	Protein Coding	42	GC07P000192	5.64746
RYR3	Ryanodine Receptor 3	Protein Coding	42	GC15P033310	5.645195
ASCC1	Activating Signal Cointegrator 1 Complex Subunit 1	Protein Coding	42	GC10M072096	5.568421
LHX4	LIM Homeobox 4	Protein Coding	42	GC01P180230	5.463642
DTNBP1	Dystrobrevin Binding Protein 1	Protein Coding	42	GC06M015470	5.450791
MRE11	MRE11 Homolog, Double Strand Break Repair Nuclease	Protein Coding	42	GC11M094875	5.367863
KLF15	Kruppel Like Factor 15	Protein Coding	42	GC03M126293	5.345297
DSTYK	Dual Serine/Threonine And Tyrosine Protein Kinase	Protein Coding	42	GC01M205111	5.296996
AAAS	Aladin WD Repeat Nucleoporin	Protein Coding	42	GC12M053307	5.259627
MPDU1	Mannose-P-Dolichol Utilization Defect 1	Protein Coding	42	GC17P007583	5.238617
CIB1	Calcium And Integrin Binding 1	Protein Coding	42	GC15M090229	5.1675
DLL3	Delta Like Canonical Notch Ligand 3	Protein Coding	42	GC19P039498	5.116744
COL12A1	Collagen Type XII Alpha 1 Chain	Protein Coding	42	GC06M075084	5.088801
PKP1	Plakophilin 1	Protein Coding	42	GC01P201283	5.075875
DECR1	2,4-Dienoyl-CoA Reductase 1	Protein Coding	42	GC08P090001	5.074478
FOXN1	Forkhead Box N1	Protein Coding	42	GC17P028506	5.06321
KISS1	KiSS-1 Metastasis Suppressor	Protein Coding	42	GC01M204190	5.061994
HPSE2	Heparanase 2 (Inactive)	Protein Coding	42	GC10M098457	5.047883
CRLF1	Cytokine Receptor Like Factor 1	Protein Coding	42	GC19M018572	4.843728
ACTL6A	Actin Like 6A	Protein Coding	42	GC03P179562	4.788052
NADSYN1	NAD Synthetase 1	Protein Coding	42	GC11P071454	4.767478
ARCN1	Archain 1	Protein Coding	42	GC11P118572	4.757965
ATP4A	ATPase H+/K+ Transporting Subunit Alpha	Protein Coding	42	GC19M047080	4.735036
CSGALNACT1	Chondroitin Sulfate N-Acetylgalactosaminyltransferase 1	Protein Coding	42	GC08M019404	4.694181
TNIP1	TNFAIP3 Interacting Protein 1	Protein Coding	42	GC05M151029	4.679158
RNF13	Ring Finger Protein 13	Protein Coding	42	GC03P149812	4.659188
HAS2	Hyaluronan Synthase 2	Protein Coding	42	GC08M121594	4.64067
GCKR	Glucokinase Regulator	Protein Coding	42	GC02P027496	4.618509
IAPP	Islet Amyloid Polypeptide	Protein Coding	42	GC12P021354	4.606965
KAT8	Lysine Acetyltransferase 8	Protein Coding	42	GC16P032545	4.555861
SLC6A20	Solute Carrier Family 6 Member 20	Protein Coding	42	GC03M045755	4.517224
PRPF8	Pre-MRNA Processing Factor 8	Protein Coding	42	GC17M001650	4.488684
CAPN10	Calpain 10	Protein Coding	42	GC02P240586	4.446715
SCRIB	Scribble Planar Cell Polarity Protein	Protein Coding	42	GC08M143893	4.382749
DST	Dystonin	Protein Coding	42	GC06M056457	4.30316
CD80	CD80 Molecule	Protein Coding	42	GC03M119524	4.294144
NXN	Nucleoredoxin	Protein Coding	42	GC17M000799	4.258074
ZBTB17	Zinc Finger And BTB Domain Containing 17	Protein Coding	42	GC01M015943	4.24133
VPS11	VPS11 Core Subunit Of CORVET And HOPS Complexes	Protein Coding	42	GC11P119067	4.191331
SLC34A3	Solute Carrier Family 34 Member 3	Protein Coding	42	GC09P137230	4.153121
RFX5	Regulatory Factor X5	Protein Coding	42	GC01M151340	4.134438
PBX3	PBX Homeobox 3	Protein Coding	42	GC09P125747	4.125494
CA3	Carbonic Anhydrase 3	Protein Coding	42	GC08P085373	4.120676

SMARCD3	SWI/SNF Related, Matrix Associated, Actin Dependent Regulator Of Chromatin, Subfamily D, Member 3	Protein Coding	42	GC07M151238	4.103626
BHLHE40	Basic Helix-Loop-Helix Family Member E40	Protein Coding	42	GC03P004980	4.095433
TRPC5	Transient Receptor Potential Cation Channel Subfamily C Member 5	Protein Coding	42	GC0XM111774	4.074678
TLN1	Talin 1	Protein Coding	42	GC09M035696	4.073605
IL21R	Interleukin 21 Receptor	Protein Coding	42	GC16P027413	4.072236
BPI	Bactericidal Permeability Increasing Protein	Protein Coding	42	GC20P038304	4.039881
RHOG	Ras Homolog Family Member G	Protein Coding	42	GC11M003848	4.03168
IL17F	Interleukin 17F	Protein Coding	42	GC06M052209	4.028411
BMP5	Bone Morphogenetic Protein 5	Protein Coding	42	GC06M055728	4.013843
RNF8	Ring Finger Protein 8	Protein Coding	42	GC06P055359	3.999697
ACKR3	Atypical Chemokine Receptor 3	Protein Coding	42	GC02P236537	3.987646
TPD52	Tumor Protein D52	Protein Coding	42	GC08M079920	3.980852
RGS6	Regulator Of G Protein Signaling 6	Protein Coding	42	GC14P071867	3.971102
DMP1	Dentin Matrix Acidic Phosphoprotein 1	Protein Coding	42	GC04P087650	3.864394
PHOX2A	Paired Like Homeobox 2A	Protein Coding	42	GC11M072239	3.823494
KIF3A	Kinesin Family Member 3A	Protein Coding	42	GC05M132689	3.820851
GJC2	Gap Junction Protein Gamma 2	Protein Coding	42	GC01P228471	3.7919
CABIN1	Calcineurin Binding Protein 1	Protein Coding	42	GC22P024011	3.787342
ST8SIA2	ST8 Alpha-N-Acetyl-Neuraminide Alpha-2,8-Sialyltransferase 2	Protein Coding	42	GC15P092393	3.691423
NPAS2	Neuronal PAS Domain Protein 2	Protein Coding	42	GC02P100820	3.654299
BTG1	BTG Anti-Proliferation Factor 1	Protein Coding	42	GC12M092140	3.637992
LARP7	La Ribonucleoprotein 7, Transcriptional Regulator	Protein Coding	42	GC04P112636	3.627311
FTSJ1	FtsJ RNA 2'-O-Methyltransferase 1	Protein Coding	42	GC0XP048476	3.611736
ALG3	ALG3 Alpha-1,3- Mannosyltransferase	Protein Coding	42	GC03M184244	3.609875
CISD2	CDGSH Iron Sulfur Domain 2	Protein Coding	42	GC04P102868	3.592381
FLVCR2	FLVCR Heme Transporter 2	Protein Coding	42	GC14P075578	3.571872
HNRNPC	Heterogeneous Nuclear Ribonucleoprotein C	Protein Coding	42	GC14M021210	3.559523
CHRD	Chordin	Protein Coding	42	GC03P184380	3.54401
SH3KBP1	SH3 Domain Containing Kinase Binding Protein 1	Protein Coding	42	GC0XM019552	3.516529
SIK3	SIK Family Kinase 3	Protein Coding	42	GC11M116843	3.507767
SP7	Sp7 Transcription Factor	Protein Coding	42	GC12M053326	3.477754
SVIL	Supervillin	Protein Coding	42	GC10M031105	3.438235
ITPK1	Inositol-Tetrakisphosphate 1-Kinase	Protein Coding	42	GC14M092936	3.425023
WNT9B	Wnt Family Member 9B	Protein Coding	42	GC17P046833	3.387837
NRXN3	Neurexin 3	Protein Coding	42	GC14P077980	3.380167
LTK	Leukocyte Receptor Tyrosine Kinase	Protein Coding	42	GC15M041503	3.362621
PTP4A3	Protein Tyrosine Phosphatase 4A3	Protein Coding	42	GC08P141391	3.355923
TRIM5	Tripartite Motif Containing 5	Protein Coding	42	GC11M005753	3.342764
RPL8	Ribosomal Protein L8	Protein Coding	42	GC08M144821	3.331875
CEACAM5	CEA Cell Adhesion Molecule 5	Protein Coding	42	GC19P041709	3.320553
DMC1	DNA Meiotic Recombinase 1	Protein Coding	42	GC22M048519	3.303635
GSTZ1	Glutathione S-Transferase Zeta 1	Protein Coding	42	GC14P077320	3.297508
DAAM1	Dishevelled Associated Activator Of Morphogenesis 1	Protein Coding	42	GC14P059188	3.250204
HHIP	Hedgehog Interacting Protein	Protein Coding	42	GC04P144645	3.238893
PTGDR2	Prostaglandin D2 Receptor 2	Protein Coding	42	GC11M060850	3.218536
RPLP0	Ribosomal Protein Lateral Stalk Subunit P0	Protein Coding	42	GC12M120196	3.199529
SLIT3	Slit Guidance Ligand 3	Protein Coding	42	GC05M168661	3.177511
RPLP2	Ribosomal Protein Lateral Stalk Subunit P2	Protein Coding	42	GC11P000984	3.171037
PERP	P53 Apoptosis Effector Related To PMP22	Protein Coding	42	GC06M138088	3.166299
TDP2	Tyrosyl-DNA Phosphodiesterase 2	Protein Coding	42	GC06M024651	3.154409
KLRD1	Killer Cell Lectin Like Receptor D1	Protein Coding	42	GC12P010226	3.150889
MED1	Mediator Complex Subunit 1	Protein Coding	42	GC17M039404	3.144341
EHD1	EH Domain Containing 1	Protein Coding	42	GC11M069343	3.125283

PLEKHM1	Pleckstrin Homology And RUN Domain Containing M1	Protein Coding	42	GC17M045435	3.120058
CNTF	Ciliary Neurotrophic Factor	Protein Coding	42	GC11P058622	3.108138
SALL2	Spalt Like Transcription Factor 2	Protein Coding	42	GC14M021521	3.033072
ZP3	Zona Pellucida Glycoprotein 3	Protein Coding	42	GC07P076398	3.019599
LUM	Lumican	Protein Coding	42	GC12M091102	2.997677
NOX1	NADPH Oxidase 1	Protein Coding	42	GC0XM100843	2.986122
FGF18	Fibroblast Growth Factor 18	Protein Coding	42	GC05P171419	2.982879
ASAH2	N-Acylsphingosine Amidohydrolase 2	Protein Coding	42	GC10M050182	2.973333
CXCL13	C-X-C Motif Chemokine Ligand 13	Protein Coding	42	GC04P077511	2.94631
PHF8	PHD Finger Protein 8	Protein Coding	42	GC0XM053936	2.915673
CREB3L1	CAMP Responsive Element Binding Protein 3 Like 1	Protein Coding	42	GC11P046299	2.901645
PIGB	Phosphatidylinositol Glycan Anchor Biosynthesis Class B	Protein Coding	42	GC15P055318	2.855133
RAD52	RAD52 Homolog, DNA Repair Protein	Protein Coding	42	GC12M000912	2.846374
WNT16	Wnt Family Member 16	Protein Coding	42	GC07P121325	2.798061
PKD2L1	Polycystin 2 Like 1, Transient Receptor Potential Cation Channel	Protein Coding	42	GC10M100288	2.792998
LOXL3	Lysyl Oxidase Like 3	Protein Coding	42	GC02M074532	2.787391
IL22	Interleukin 22	Protein Coding	42	GC12M068248	2.784217
SYN3	Synapsin III	Protein Coding	42	GC22M032512	2.770432
PMS1	PMS1 Homolog 1, Mismatch Repair System Component	Protein Coding	42	GC02P189784	2.762096
STK33	Serine/Threonine Kinase 33	Protein Coding	42	GC11M008335	2.755264
DGKH	Diacylglycerol Kinase Eta	Protein Coding	42	GC13P042040	2.754732
STIL	STIL Centriolar Assembly Protein	Protein Coding	42	GC01M047250	2.726913
TCF7L1	Transcription Factor 7 Like 1	Protein Coding	42	GC02P085133	2.718533
PABPC4	Poly(A) Binding Protein Cytoplasmic 4	Protein Coding	42	GC01M039560	2.712547
CNN1	Calponin 1	Protein Coding	42	GC19P011539	2.7079
SCN11A	Sodium Voltage-Gated Channel Alpha Subunit 11	Protein Coding	42	GC03M038862	2.699327
ICAM3	Intercellular Adhesion Molecule 3	Protein Coding	42	GC19M010335	2.695589
KDM2B	Lysine Demethylase 2B	Protein Coding	42	GC12M121402	2.693637
ELMO1	Engulfment And Cell Motility 1	Protein Coding	42	GC07M036860	2.680638
OPLAH	5-Oxoprolinase, ATP-Hydrolysing	Protein Coding	42	GC08M144051	2.672109
LRIG1	Leucine Rich Repeats And Immunoglobulin Like Domains 1	Protein Coding	42	GC03M066379	2.660629
UGT1A6	UDP Glucuronosyltransferase Family 1 Member A6	Protein Coding	42	GC02P233691	2.655355
SLC30A5	Solute Carrier Family 30 Member 5	Protein Coding	42	GC05P069093	2.648874
MAP4	Microtubule Associated Protein 4	Protein Coding	42	GC03M047850	2.645588
SNCG	Synuclein Gamma	Protein Coding	42	GC10P086957	2.643631
CAPZA2	Capping Actin Protein Of Muscle Z-Line Subunit Alpha 2	Protein Coding	42	GC07P116811	2.638445
RAPGEF2	Rap Guanine Nucleotide Exchange Factor 2	Protein Coding	42	GC04P159106	2.618512
SLC47A1	Solute Carrier Family 47 Member 1	Protein Coding	42	GC17P019495	2.61094
XPO5	Exportin 5	Protein Coding	42	GC06M043522	2.584839
VPS37A	VPS37A Subunit Of ESCRT-I	Protein Coding	42	GC08P017246	2.581525
TRNT1	TRNA Nucleotidyl Transferase 1	Protein Coding	42	GC03P003126	2.559843
HOXA11	Homeobox A11	Protein Coding	42	GC07M027343	2.539498
DUOX1	Dual Oxidase 1	Protein Coding	42	GC15P045129	2.531964
CIDEC	Cell Death Inducing DFFA Like Effector C	Protein Coding	42	GC03M009866	2.530465
FCGR1A	Fc Fragment Of IgG Receptor Ia	Protein Coding	42	GC01P149754	2.519976
PANK1	Pantothenate Kinase 1	Protein Coding	42	GC10M089579	2.501659
MSRB2	Methionine Sulfoxide Reductase B2	Protein Coding	42	GC10P023053	2.478143
MTF1	Metal Regulatory Transcription Factor 1	Protein Coding	42	GC01M037810	2.477537
KCNH6	Potassium Voltage-Gated Channel Subfamily H Member 6	Protein Coding	42	GC17P063523	2.473936
MIP	Major Intrinsic Protein Of Lens Fiber	Protein Coding	42	GC12M056449	2.464649
CALU	Calumenin	Protein Coding	42	GC07P128739	2.461381
SEMA3F	Semaphorin 3F	Protein Coding	42	GC03P050167	2.451499
ITSN1	Intersectin 1	Protein Coding	42	GC21P033642	2.450644

ADAM23	ADAM Metallopeptidase Domain 23	Protein Coding	42	GC02P206443	2.442639
CXCR5	C-X-C Motif Chemokine Receptor 5	Protein Coding	42	GC11P118908	2.432995
CCT3	Chaperonin Containing TCP1 Subunit 3	Protein Coding	42	GC01M156308	2.42725
MXI1	MAX Interactor 1, Dimerization Protein	Protein Coding	42	GC10P110208	2.389299
RCC1	Regulator Of Chromosome Condensation 1	Protein Coding	42	GC01P028505	2.378953
SP100	SP100 Nuclear Antigen	Protein Coding	42	GC02P230415	2.368051
CACNG5	Calcium Voltage-Gated Channel Auxiliary Subunit Gamma 5	Protein Coding	42	GC17P066835	2.367553
ZMYND11	Zinc Finger MYND-Type Containing 11	Protein Coding	42	GC10P000134	2.366177
MICB	MHC Class I Polypeptide-Related Sequence B	Protein Coding	42	GC06P055197	2.35998
PLS1	Plastin 1	Protein Coding	42	GC03P142596	2.347886
UNC119	Unc-119 Lipid Binding Chaperone	Protein Coding	42	GC17M028546	2.322089
SLC14A2	Solute Carrier Family 14 Member 2	Protein Coding	42	GC18P045167	2.312973
CDC27	Cell Division Cycle 27	Protein Coding	42	GC17M047117	2.30771
CAPZA1	Capping Actin Protein Of Muscle Z-Line Subunit Alpha 1	Protein Coding	42	GC01P112619	2.304953
HOXA5	Homeobox A5	Protein Coding	42	GC07M027142	2.301592
RPL3	Ribosomal Protein L3	Protein Coding	42	GC22M048526	2.286248
HSF4	Heat Shock Transcription Factor 4	Protein Coding	42	GC16P067164	2.274401
NDEL1	NudE Neurodevelopment Protein 1 Like 1	Protein Coding	42	GC17P009152	2.263227
CAMP	Cathelicidin Antimicrobial Peptide	Protein Coding	42	GC03P048342	2.260959
ADAM15	ADAM Metallopeptidase Domain 15	Protein Coding	42	GC01P155023	2.26008
SBF1	SET Binding Factor 1	Protein Coding	42	GC22M050445	2.251667
OXSR1	Oxidative Stress Responsive Kinase 1	Protein Coding	42	GC03P038183	2.22944
SLC30A9	Solute Carrier Family 30 Member 9	Protein Coding	42	GC04P041992	2.221517
POLRMT	RNA Polymerase Mitochondrial	Protein Coding	42	GC19M000617	2.211807
ALDH9A1	Aldehyde Dehydrogenase 9 Family Member A1	Protein Coding	42	GC01M165670	2.208362
LGALS8	Galectin 8	Protein Coding	42	GC01P236518	2.196667
BACH2	BTB Domain And CNC Homolog 2	Protein Coding	42	GC06M089926	2.191127
CLIC1	Chloride Intracellular Channel 1	Protein Coding	42	GC06M046892	2.170879
EIF4H	Eukaryotic Translation Initiation Factor 4H	Protein Coding	42	GC07P074174	2.169286
PDCD1LG2	Programmed Cell Death 1 Ligand 2	Protein Coding	42	GC09P005510	2.161244
SERPINE2	Serpin Family E Member 2	Protein Coding	42	GC02M223975	2.161062
AP3B2	Adaptor Related Protein Complex 3 Subunit Beta 2	Protein Coding	42	GC15M083288	2.1608
EXOC5	Exocyst Complex Component 5	Protein Coding	42	GC14M057200	2.123945
TBC1D7	TBC1 Domain Family Member 7	Protein Coding	42	GC06M013266	2.121078
LIPF	Lipase F, Gastric Type	Protein Coding	42	GC10P088664	2.118608
AGPAT5	1-Acylglycerol-3-Phosphate O-Acyltransferase 5	Protein Coding	42	GC08P006708	2.116289
UNC13B	Unc-13 Homolog B	Protein Coding	42	GC09P035161	2.107276
KPNA1	Karyopherin Subunit Alpha 1	Protein Coding	42	GC03M122421	2.10431
ESAM	Endothelial Cell Adhesion Molecule	Protein Coding	42	GC11M124752	2.103598
CSRP1	Cysteine And Glycine Rich Protein 1	Protein Coding	42	GC01M201484	2.098094
SLC35A3	Solute Carrier Family 35 Member A3	Protein Coding	42	GC01P099968	2.091556
TBX15	T-Box Transcription Factor 15	Protein Coding	42	GC01M118883	2.083774
PCBP2	Poly(RC) Binding Protein 2	Protein Coding	42	GC12P053452	2.064173
SLC2A6	Solute Carrier Family 2 Member 6	Protein Coding	42	GC09M133471	2.048595
RAB35	RAB35, Member RAS Oncogene Family	Protein Coding	42	GC12M120096	2.035941
CRY2	Cryptochrome Circadian Regulator 2	Protein Coding	42	GC11P046088	2.033656
PFN2	Profilin 2	Protein Coding	42	GC03M149964	2.032864
APOBEC3G	Apolipoprotein B MRNA Editing Enzyme Catalytic Subunit 3G	Protein Coding	42	GC22P039078	2.026975
RPS3A	Ribosomal Protein S3A	Protein Coding	42	GC04P151099	2.009954
SNX9	Sorting Nexin 9	Protein Coding	42	GC06P157685	2.006174
ENAH	ENAH Actin Regulator	Protein Coding	42	GC01M225486	2.002631
CAMK1D	Calcium/Calmodulin Dependent Protein Kinase ID	Protein Coding	42	GC10P012349	1.998123
HERC1	HECT And RLD Domain Containing E3 Ubiquitin Protein Ligase Family Member 1	Protein Coding	42	GC15M063608	1.972686

SSTR5	Somatostatin Receptor 5	Protein Coding	42	GC16P001072	1.943183
RXFP3	Relaxin Family Peptide Receptor 3	Protein Coding	42	GC05P033937	1.942071
HIBADH	3-Hydroxyisobutyrate Dehydrogenase	Protein Coding	42	GC07M027525	1.941822
SLAMF7	SLAM Family Member 7	Protein Coding	42	GC01P160709	1.937372
CYP46A1	Cytochrome P450 Family 46 Subfamily A Member 1	Protein Coding	42	GC14P099684	1.907371
MLC1	Modulator Of VRAC Current 1	Protein Coding	42	GC22M050059	1.903518
SRM	Spermidine Synthase	Protein Coding	42	GC01M011054	1.90178
VAMP8	Vesicle Associated Membrane Protein 8	Protein Coding	42	GC02P085561	1.901495
AKAP8	A-Kinase Anchoring Protein 8	Protein Coding	42	GC19M015354	1.898219
SNRNP200	Small Nuclear Ribonucleoprotein U5 Subunit 200	Protein Coding	42	GC02M096363	1.8958
PTGES	Prostaglandin E Synthase	Protein Coding	42	GC09M129738	1.894783
FMO4	Flavin Containing Dimethylaniline Monoxygenase 4	Protein Coding	42	GC01P171315	1.891191
ITGB8	Integrin Subunit Beta 8	Protein Coding	42	GC07P020329	1.887231
BRD3	Bromodomain Containing 3	Protein Coding	42	GC09M134030	1.882037
HLA-E	Major Histocompatibility Complex, Class I, E	Protein Coding	42	GC06P055176	1.87764
EXOC7	Exocyst Complex Component 7	Protein Coding	42	GC17M076080	1.845269
IL1RL2	Interleukin 1 Receptor Like 2	Protein Coding	42	GC02P102186	1.831381
SMOC2	SPARC Related Modular Calcium Binding 2	Protein Coding	42	GC06P168441	1.813616
KIFAP3	Kinesin Associated Protein 3	Protein Coding	42	GC01M169921	1.797819
HLA-DMB	Major Histocompatibility Complex, Class II, DM Beta	Protein Coding	42	GC06M032934	1.789022
SORBS1	Sorbin And SH3 Domain Containing 1	Protein Coding	42	GC10M095311	1.77897
CLDN4	Claudin 4	Protein Coding	42	GC07P073799	1.777472
GBP1	Guanylate Binding Protein 1	Protein Coding	42	GC01M089052	1.76792
RNF125	Ring Finger Protein 125	Protein Coding	42	GC18P032025	1.728584
SLC17A8	Solute Carrier Family 17 Member 8	Protein Coding	42	GC12P100357	1.717636
TRIM27	Tripartite Motif Containing 27	Protein Coding	42	GC06M028903	1.70418
MSI2	Musashi RNA Binding Protein 2	Protein Coding	42	GC17P057255	1.687095
CCL7	C-C Motif Chemokine Ligand 7	Protein Coding	42	GC17P034270	1.683549
KIDINS220	Kinase D Interacting Substrate 220	Protein Coding	42	GC02M008715	1.677295
FABP5	Fatty Acid Binding Protein 5	Protein Coding	42	GC08P081282	1.676217
SULF1	Sulfatase 1	Protein Coding	42	GC08P069466	1.659108
LTBR	Lymphotoxin Beta Receptor	Protein Coding	42	GC12P006375	1.639248
GSTM2	Glutathione S-Transferase Mu 2	Protein Coding	42	GC01P109668	1.635105
CDKL1	Cyclin Dependent Kinase Like 1	Protein Coding	42	GC14M050330	1.619398
ARHGAP9	Rho GTPase Activating Protein 9	Protein Coding	42	GC12M057472	1.616395
ATP1B2	ATPase Na+/K+ Transporting Subunit Beta 2	Protein Coding	42	GC17P009113	1.615064
SCD5	Stearoyl-CoA Desaturase 5	Protein Coding	42	GC04M082629	1.605949
PRKX	Protein Kinase X-Linked	Protein Coding	42	GC0XM003604	1.597194
NMB	Neuromedin B	Protein Coding	42	GC15M084655	1.581587
ABCA2	ATP Binding Cassette Subfamily A Member 2	Protein Coding	42	GC09M137007	1.565152
CSGALNACT2	Chondroitin Sulfate N-Acetylgalactosaminyltransferase 2	Protein Coding	42	GC10P043138	1.562855
SLC17A7	Solute Carrier Family 17 Member 7	Protein Coding	42	GC19M049429	1.557192
PSMB3	Proteasome 20S Subunit Beta 3	Protein Coding	42	GC17P038752	1.557099
MLKL	Mixed Lineage Kinase Domain Like Pseudokinase	Protein Coding	42	GC16M074672	1.546646
BACH1	BTB Domain And CNC Homolog 1	Protein Coding	42	GC21P029194	1.545909
JPH3	Junctophilin 3	Protein Coding	42	GC16P087601	1.539269
RING1	Ring Finger Protein 1	Protein Coding	42	GC06P033208	1.526191
COX5B	Cytochrome C Oxidase Subunit 5B	Protein Coding	42	GC02P097628	1.518799
PDE8A	Phosphodiesterase 8A	Protein Coding	42	GC15P085364	1.48437
POLR2F	RNA Polymerase II, I And III Subunit F	Protein Coding	42	GC22P037952	1.480959
GPSM2	G Protein Signaling Modulator 2	Protein Coding	42	GC01P108875	1.471946
NDC80	NDC80 Kinetochore Complex Component	Protein Coding	42	GC18P002571	1.44687
WNT8B	Wnt Family Member 8B	Protein Coding	42	GC10P100463	1.441401

RPL23A	Ribosomal Protein L23a	Protein Coding	42	GC17P028719	1.415044
KCNK4	Potassium Two Pore Domain Channel Subfamily K Member 4	Protein Coding	42	GC11P064292	1.41225
SLC6A17	Solute Carrier Family 6 Member 17	Protein Coding	42	GC01P110150	1.409826
ATP6V0C	ATPase H+ Transporting V0 Subunit C	Protein Coding	42	GC16P002513	1.407732
NLRP2	NLR Family Pyrin Domain Containing 2	Protein Coding	42	GC19P054953	1.402438
FLOT1	Flotillin 1	Protein Coding	42	GC06M046803	1.397296
IL15RA	Interleukin 15 Receptor Subunit Alpha	Protein Coding	42	GC10M005943	1.374467
DNAJA1	DnaJ Heat Shock Protein Family (Hsp40) Member A1	Protein Coding	42	GC09P033025	1.373883
SIRT7	Sirtuin 7	Protein Coding	42	GC17M081911	1.369205
DOCK7	Dedicator Of Cytokinesis 7	Protein Coding	42	GC01M062454	1.364119
CHD1L	Chromodomain Helicase DNA Binding Protein 1 Like	Protein Coding	42	GC01P147203	1.357763
PDZK1	PDZ Domain Containing 1	Protein Coding	42	GC01M145670	1.355334
CTDP1	CTD Phosphatase Subunit 1	Protein Coding	42	GC18P079679	1.349097
UGT1A4	UDP Glucuronosyltransferase Family 1 Member A4	Protein Coding	42	GC02P233718	1.343771
DUSP2	Dual Specificity Phosphatase 2	Protein Coding	42	GC02M096271	1.343518
CDC37	Cell Division Cycle 37, HSP90 Cochaperone	Protein Coding	42	GC19M010391	1.333778
PELI1	Pellino E3 Ubiquitin Protein Ligase 1	Protein Coding	42	GC02M064092	1.333771
TM7SF2	Transmembrane 7 Superfamily Member 2	Protein Coding	42	GC11P065111	1.333596
AADAC	Arylacetamide Deacetylase	Protein Coding	42	GC03P151813	1.302693
ARHGEF11	Rho Guanine Nucleotide Exchange Factor 11	Protein Coding	42	GC01M156904	1.289667
CYB5R1	Cytochrome B5 Reductase 1	Protein Coding	42	GC01M202964	1.275576
ATP6V1C2	ATPase H+ Transporting V1 Subunit C2	Protein Coding	42	GC02P010713	1.274517
ARHGAP4	Rho GTPase Activating Protein 4	Protein Coding	42	GC0XM153907	1.264438
KLC1	Kinesin Light Chain 1	Protein Coding	42	GC14P106043	1.264113
OSBPL2	Oxysterol Binding Protein Like 2	Protein Coding	42	GC20P062231	1.260716
CUL4A	Cullin 4A	Protein Coding	42	GC13P113208	1.251045
CLDN7	Claudin 7	Protein Coding	42	GC17M007259	1.248819
CRABP1	Cellular Retinoic Acid Binding Protein 1	Protein Coding	42	GC15P078340	1.238496
TUBB6	Tubulin Beta 6 Class V	Protein Coding	42	GC18P012307	1.238446
EREG	Epiregulin	Protein Coding	42	GC04P074366	1.237321
EIF1AX	Eukaryotic Translation Initiation Factor 1A X-Linked	Protein Coding	42	GC0XM020124	1.228827
HLA-F	Major Histocompatibility Complex, Class I, F	Protein Coding	42	GC06P055144	1.228258
PARL	Presenilin Associated Rhomboid Like	Protein Coding	42	GC03M183826	1.198326
IL17B	Interleukin 17B	Protein Coding	42	GC05M149371	1.191687
IGF2BP3	Insulin Like Growth Factor 2 MRNA Binding Protein 3	Protein Coding	42	GC07M023316	1.186558
NXF1	Nuclear RNA Export Factor 1	Protein Coding	42	GC11M069264	1.182868
MLLT10	MLLT10 Histone Lysine Methyltransferase DOT1L Cofactor	Protein Coding	42	GC10P021524	1.178127
MAFA	MAF BZIP Transcription Factor A	Protein Coding	42	GC08M143419	1.168471
PASK	PAS Domain Containing Serine/Threonine Kinase	Protein Coding	42	GC02M241106	1.159353
HIP1R	Huntingtin Interacting Protein 1 Related	Protein Coding	42	GC12P122834	1.157081
DCTN2	Dynactin Subunit 2	Protein Coding	42	GC12M057570	1.152288
PGK2	Phosphoglycerate Kinase 2	Protein Coding	42	GC06M049785	1.148968
MS4A2	Membrane Spanning 4-Domains A2	Protein Coding	42	GC11P060088	1.147643
PGM2	Phosphoglucomutase 2	Protein Coding	42	GC04P037865	1.145872
ARPC5	Actin Related Protein 2/3 Complex Subunit 5	Protein Coding	42	GC01M183621	1.144217
WNT8A	Wnt Family Member 8A	Protein Coding	42	GC05P138091	1.131886
AIMP2	Aminoacyl TRNA Synthetase Complex Interacting Multifunctional Protein 2	Protein Coding	42	GC07P006016	1.124573
RCHY1	Ring Finger And CHY Zinc Finger Domain Containing 1	Protein Coding	42	GC04M075479	1.121078
C8A	Complement C8 Alpha Chain	Protein Coding	42	GC01P056854	1.081353
GCNT1	Glucosaminyl (N-Acetyl) Transferase 1	Protein Coding	42	GC09P076420	1.080772
UBE2S	Ubiquitin Conjugating Enzyme E2 S	Protein Coding	42	GC19M055399	1.079234
ARL2	ADP Ribosylation Factor Like GTPase 2	Protein Coding	42	GC11P065015	1.072289
CDH6	Cadherin 6	Protein Coding	42	GC05P031193	1.052761

CTNNBIP1	Catenin Beta Interacting Protein 1	Protein Coding	42	GC01M009848	1.045396
ATG12	Autophagy Related 12	Protein Coding	42	GC05M115828	1.039356
PLA2G12A	Phospholipase A2 Group XIIA	Protein Coding	42	GC04M109712	1.035847
LOXL4	Lysyl Oxidase Like 4	Protein Coding	42	GC10M098247	1.02583
ATL1	Atlastin GTPase 1	Protein Coding	42	GC14P050532	1.020638
FHL3	Four And A Half LIM Domains 3	Protein Coding	42	GC01M037997	1.009555
KDELR2	KDEL Endoplasmic Reticulum Protein Retention Receptor 2	Protein Coding	42	GC07M006447	1.002547
SNX1	Sorting Nexin 1	Protein Coding	42	GC15P064094	0.99186
CCL19	C-C Motif Chemokine Ligand 19	Protein Coding	42	GC09M034692	0.968834
MYO3A	Myosin IIIA	Protein Coding	42	GC10P025934	0.953812
AP4B1	Adaptor Related Protein Complex 4 Subunit Beta 1	Protein Coding	42	GC01M113894	0.953752
RP2	RP2 Activator Of ARL3 GTPase	Protein Coding	42	GC0XP046836	0.947557
SP4	Sp4 Transcription Factor	Protein Coding	42	GC07P021434	0.945306
GPRC5A	G Protein-Coupled Receptor Class C Group 5 Member A	Protein Coding	42	GC12P012939	0.942539
CPD	Carboxypeptidase D	Protein Coding	42	GC17P030378	0.934715
NRCAM	Neuronal Cell Adhesion Molecule	Protein Coding	42	GC07M108147	0.920547
GSTA2	Glutathione S-Transferase Alpha 2	Protein Coding	42	GC06M052750	0.919694
NPEPPS	Aminopeptidase Puromycin Sensitive	Protein Coding	42	GC17P047522	0.90541
RAB5C	RAB5C, Member RAS Oncogene Family	Protein Coding	42	GC17M042124	0.905079
RANBP9	RAN Binding Protein 9	Protein Coding	42	GC06M013621	0.899874
IL17RB	Interleukin 17 Receptor B	Protein Coding	42	GC03P053855	0.864671
CD84	CD84 Molecule	Protein Coding	42	GC01M160541	0.862008
GSTM5	Glutathione S-Transferase Mu 5	Protein Coding	42	GC01P109711	0.861863
CAMK1G	Calcium/Calmodulin Dependent Protein Kinase IG	Protein Coding	42	GC01P209583	0.854777
PRIM2	DNA Primase Subunit 2	Protein Coding	42	GC06P057314	0.84234
UCK2	Uridine-Cytidine Kinase 2	Protein Coding	42	GC01P165796	0.836329
TPX2	TPX2 Microtubule Nucleation Factor	Protein Coding	42	GC20P031739	0.817075
CYP2A13	Cytochrome P450 Family 2 Subfamily A Member 13	Protein Coding	42	GC19P041088	0.810181
ITGB3BP	Integrin Subunit Beta 3 Binding Protein	Protein Coding	42	GC01M063440	0.788823
KLK5	Kallikrein Related Peptidase 5	Protein Coding	42	GC19M050943	0.778747
BRAP	BRCA1 Associated Protein	Protein Coding	42	GC12M111642	0.778051
UBE2M	Ubiquitin Conjugating Enzyme E2 M	Protein Coding	42	GC19M058555	0.764646
GNG12	G Protein Subunit Gamma 12	Protein Coding	42	GC01M067701	0.75791
EIF4EBP2	Eukaryotic Translation Initiation Factor 4E Binding Protein 2	Protein Coding	42	GC10P070404	0.722006
MPG	N-Methylpurine DNA Glycosylase	Protein Coding	42	GC16P005486	0.715187
IRAK2	Interleukin 1 Receptor Associated Kinase 2	Protein Coding	42	GC03P011014	0.707934
BATF	Basic Leucine Zipper ATF-Like Transcription Factor	Protein Coding	42	GC14P075523	0.705712
GLYAT	Glycine-N-Acyltransferase	Protein Coding	42	GC11M069098	0.690326
ENTPD3	Ectonucleoside Triphosphate Diphosphohydrolase 3	Protein Coding	42	GC03P040403	0.671432
TGM5	Transglutaminase 5	Protein Coding	42	GC15M043273	0.659442
ARR3	Arrestin 3	Protein Coding	42	GC0XP070269	0.63146
EXOSC2	Exosome Component 2	Protein Coding	42	GC09P130693	0.612599
CD37	CD37 Molecule	Protein Coding	42	GC19P049335	0.611148
CLDN2	Claudin 2	Protein Coding	42	GC0XP106900	0.575959
SULT4A1	Sulfotransferase Family 4A Member 1	Protein Coding	42	GC22M043824	0.537362
NAPSA	Napsin A Aspartic Peptidase	Protein Coding	42	GC19M050417	0.471663
FLOT2	Flotillin 2	Protein Coding	42	GC17M031085	0.413307
E2F5	E2F Transcription Factor 5	Protein Coding	42	GC08P085177	0.350188
RTEL1	Regulator Of Telomere Elongation Helicase 1	Protein Coding	41	GC20P063658	46.50262
TMEM43	Transmembrane Protein 43	Protein Coding	41	GC03P014124	31.30201
BRF1	BRF1 RNA Polymerase III Transcription Initiation Factor Subunit	Protein Coding	41	GC14M105345	26.72366
CERS1	Ceramide Synthase 1	Protein Coding	41	GC19M018868	26.06376
ANK2	Ankyrin 2	Protein Coding	41	GC04P112706	23.91219

MYPN	Myopalladin	Protein Coding	41	GC10P068106	23.8623
EPO	Erythropoietin	Protein Coding	41	GC07P100720	23.2576
CEP290	Centrosomal Protein 290	Protein Coding	41	GC12M088049	23.02426
MKS1	MKS Transition Zone Complex Subunit 1	Protein Coding	41	GC17M058205	22.19519
PIGL	Phosphatidylinositol Glycan Anchor Biosynthesis Class L	Protein Coding	41	GC17P016217	21.25952
TRDN	Triadin	Protein Coding	41	GC06M123198	20.4888
FOXL2	Forkhead Box L2	Protein Coding	41	GC03M138944	20.43124
NIPBL	NIPBL Cohesin Loading Factor	Protein Coding	41	GC05P036876	19.06902
NPPC	Natriuretic Peptide C	Protein Coding	41	GC02M231921	19.04237
FKRP	Fukutin Related Protein	Protein Coding	41	GC19P046746	17.17496
BMP15	Bone Morphogenetic Protein 15	Protein Coding	41	GC0XP050910	16.97572
COQ2	Coenzyme Q2, Polyprenyltransferase	Protein Coding	41	GC04M083261	15.46774
UTS2	Urotensin 2	Protein Coding	41	GC01M007843	15.39859
MCM8	Minichromosome Maintenance 8 Homologous Recombination Repair Factor	Protein Coding	41	GC20P005988	15.24173
PLXND1	Plexin D1	Protein Coding	41	GC03M129555	15.08967
PECAM1	Platelet And Endothelial Cell Adhesion Molecule 1	Protein Coding	41	GC17M064319	14.88853
BBS10	Bardet-Biedl Syndrome 10	Protein Coding	41	GC12M076344	14.43979
BGLAP	Bone Gamma-Carboxyglutamate Protein	Protein Coding	41	GC01P156242	14.14751
MSH5	MutS Homolog 5	Protein Coding	41	GC06P055215	13.89456
NEB	Nebulin	Protein Coding	41	GC02M151485	13.70421
NDUFAF2	NADH:Ubiquinone Oxidoreductase Complex Assembly Factor 2	Protein Coding	41	GC05P060945	13.69427
RBM10	RNA Binding Motif Protein 10	Protein Coding	41	GC0XP047206	13.69221
FBN2	Fibrillin 2	Protein Coding	41	GC05M128257	13.37914
DNAI1	Dynein Axonemal Intermediate Chain 1	Protein Coding	41	GC09P034457	13.35229
BAZ1B	Bromodomain Adjacent To Zinc Finger Domain 1B	Protein Coding	41	GC07M073440	13.25539
TNNI3K	TNNI3 Interacting Kinase	Protein Coding	41	GC01P074235	13.13039
SETBP1	SET Binding Protein 1	Protein Coding	41	GC18P044680	13.04693
SOD3	Superoxide Dismutase 3	Protein Coding	41	GC04P024798	12.94084
TRIP4	Thyroid Hormone Receptor Interactor 4	Protein Coding	41	GC15P077081	12.88442
GNPTAB	N-Acetylglucosamine-1-Phosphate Transferase Subunits Alpha And Beta	Protein Coding	41	GC12M101745	12.84978
KCNIP2	Potassium Voltage-Gated Channel Interacting Protein 2	Protein Coding	41	GC10M101825	12.82012
FKBP1B	FKBP Prolyl Isomerase 1B	Protein Coding	41	GC02P024033	12.79601
RASA2	RAS P21 Protein Activator 2	Protein Coding	41	GC03P141487	12.4146
STRA6	Signaling Receptor And Transporter Of Retinol STRA6	Protein Coding	41	GC15M074179	12.28366
PAPPA	Pappalysin 1	Protein Coding	41	GC09P116231	12.07341
PACS1	Phosphofurin Acidic Cluster Sorting Protein 1	Protein Coding	41	GC11P066320	11.97941
NPHP4	Nephrocystin 4	Protein Coding	41	GC01M005863	11.95648
SYNE1	Spectrin Repeat Containing Nuclear Envelope Protein 1	Protein Coding	41	GC06M152121	11.47619
TNFSF4	TNF Superfamily Member 4	Protein Coding	41	GC01M173183	11.15595
COG2	Component Of Oligomeric Golgi Complex 2	Protein Coding	41	GC01P230642	10.98885
GCG	Glucagon	Protein Coding	41	GC02M162142	10.98418
MCTP2	Multiple C2 And Transmembrane Domain Containing 2	Protein Coding	41	GC15P100162	10.75619
VPS33A	VPS33A Core Subunit Of CORVET And HOPS Complexes	Protein Coding	41	GC12M122229	10.58932
G6PC3	Glucose-6-Phosphatase Catalytic Subunit 3	Protein Coding	41	GC17P044070	10.5164
PEX26	Peroxisomal Biogenesis Factor 26	Protein Coding	41	GC22P018079	10.48487
SPTB	Spectrin Beta, Erythrocytic	Protein Coding	41	GC14M064746	10.38521
MTX2	Metaxin 2	Protein Coding	41	GC02P176269	10.03683
COX8A	Cytochrome C Oxidase Subunit 8A	Protein Coding	41	GC11P063994	9.958894
ARHGAP31	Rho GTPase Activating Protein 31	Protein Coding	41	GC03P119294	9.869809
ADAMTSL2	ADAMTS Like 2	Protein Coding	41	GC09P133603	9.709505
PEX13	Peroxisomal Biogenesis Factor 13	Protein Coding	41	GC02P061017	9.601372
RPGR	Retinitis Pigmentosa GTPase Regulator	Protein Coding	41	GC0XM038269	9.308945
WRAP53	WD Repeat Containing Antisense To TP53	Protein Coding	41	GC17P009114	9.263154

GTPBP3	GTP Binding Protein 3, Mitochondrial	Protein Coding	41	GC19P026637	9.191625
BVES	Blood Vessel Epicardial Substance	Protein Coding	41	GC06M105096	9.184483
NOP10	NOP10 Ribonucleoprotein	Protein Coding	41	GC15M034341	9.14475
COX7B	Cytochrome C Oxidase Subunit 7B	Protein Coding	41	GC0XP077899	9.132338
PEX11B	Peroxisomal Biogenesis Factor 11 Beta	Protein Coding	41	GC01M145911	9.123713
DSCAM	DS Cell Adhesion Molecule	Protein Coding	41	GC21M040010	9.110813
MYH8	Myosin Heavy Chain 8	Protein Coding	41	GC17M010390	9.044922
RGS5	Regulator Of G Protein Signaling 5	Protein Coding	41	GC01M163111	8.976332
GLE1	GLE1 RNA Export Mediator	Protein Coding	41	GC09P128504	8.91974
CLCNKA	Chloride Voltage-Gated Channel Ka	Protein Coding	41	GC01P016018	8.8829
KLHL7	Kelch Like Family Member 7	Protein Coding	41	GC07P023105	8.871189
JMJD1C	Jumonji Domain Containing 1C	Protein Coding	41	GC10M063167	8.842406
AFF2	AF4/FMR2 Family Member 2	Protein Coding	41	GC0XP148500	8.82333
TRIM63	Tripartite Motif Containing 63	Protein Coding	41	GC01M026062	8.758202
MEGF10	Multiple EGF Like Domains 10	Protein Coding	41	GC05P127230	8.610518
SGCB	Sarcoglycan Beta	Protein Coding	41	GC04M052019	8.542591
CHRNG	Cholinergic Receptor Nicotinic Gamma Subunit	Protein Coding	41	GC02P232539	8.416663
LMOD1	Leiomodin 1	Protein Coding	41	GC01M201896	8.341604
SHBG	Sex Hormone Binding Globulin	Protein Coding	41	GC17P007613	8.269176
INF2	Inverted Formin 2	Protein Coding	41	GC14P106060	8.181899
TNNI1	Troponin I1, Slow Skeletal Type	Protein Coding	41	GC01M201404	8.159119
BSND	Barttin CLCNK Type Accessory Subunit Beta	Protein Coding	41	GC01P054998	8.151485
CEP164	Centrosomal Protein 164	Protein Coding	41	GC11P117314	8.144897
OXT	Oxytocin/Neurophysin I Prepropeptide	Protein Coding	41	GC20P003068	8.114353
PDSS2	Decaprenyl Diphosphate Synthase Subunit 2	Protein Coding	41	GC06M107152	8.078566
IL15	Interleukin 15	Protein Coding	41	GC04P141636	7.997693
FGD1	FYVE, RhoGEF And PH Domain Containing 1	Protein Coding	41	GC0XM054488	7.95754
CCT7	Chaperonin Containing TCP1 Subunit 7	Protein Coding	41	GC02P073233	7.920975
POGZ	Pogo Transposable Element Derived With ZNF Domain	Protein Coding	41	GC01M151375	7.822339
PQBP1	Polyglutamine Binding Protein 1	Protein Coding	41	GC0XP048890	7.742347
RBM8A	RNA Binding Motif Protein 8A	Protein Coding	41	GC01M145921	7.633444
MMAA	Metabolism Of Cobalamin Associated A	Protein Coding	41	GC04P145607	7.606259
IL1RL1	Interleukin 1 Receptor Like 1	Protein Coding	41	GC02P102294	7.598774
HCRT	Hypocretin Neuropeptide Precursor	Protein Coding	41	GC17M042185	7.483093
SMCHD1	Structural Maintenance Of Chromosomes Flexible Hinge Domain Containing 1	Protein Coding	41	GC18P002649	7.465818
SETX	Senataxin	Protein Coding	41	GC09M132261	7.464951
NHLRC1	NHL Repeat Containing E3 Ubiquitin Protein Ligase 1	Protein Coding	41	GC06M018120	7.433634
TMPRSS15	Transmembrane Serine Protease 15	Protein Coding	41	GC21M018269	7.420706
EMG1	EMG1 N1-Specific Pseudouridine Methyltransferase	Protein Coding	41	GC12P006970	7.319441
SEMA5A	Semaphorin 5A	Protein Coding	41	GC05M009036	7.312991
KDM4C	Lysine Demethylase 4C	Protein Coding	41	GC09P006720	7.262097
TNFSF12	TNF Superfamily Member 12	Protein Coding	41	GC17P009104	7.233636
XPNPEP3	X-Prolyl Aminopeptidase 3	Protein Coding	41	GC22P040857	7.222133
PHEX	Phosphate Regulating Endopeptidase Homolog X-Linked	Protein Coding	41	GC0XP022032	7.191319
GALNT11	Polypeptide N-Acetylgalactosaminyltransferase 11	Protein Coding	41	GC07P152025	7.179328
RAB3GAP1	RAB3 GTPase Activating Protein Catalytic Subunit 1	Protein Coding	41	GC02P135052	7.150242
EDN2	Endothelin 2	Protein Coding	41	GC01M041478	7.117826
CELSR1	Cadherin EGF LAG Seven-Pass G-Type Receptor 1	Protein Coding	41	GC22M046360	7.094145
ZMIZ1	Zinc Finger MIZ-Type Containing 1	Protein Coding	41	GC10P079068	7.089925
SCGB1A1	Secretoglobin Family 1A Member 1	Protein Coding	41	GC11P062405	7.04652
DSE	Dermatan Sulfate Epimerase	Protein Coding	41	GC06P116255	7.045091
HSPB2	Heat Shock Protein Family B (Small) Member 2	Protein Coding	41	GC11P111913	6.981806
SFTPA2	Surfactant Protein A2	Protein Coding	41	GC10M079638	6.830184

CRB2	Crumbs Cell Polarity Complex Component 2	Protein Coding	41	GC09P123356	6.819173
DHX30	DExH-Box Helicase 30	Protein Coding	41	GC03P047802	6.732937
GBA2	Glucosylceramidase Beta 2	Protein Coding	41	GC09M035736	6.723912
AGGF1	Angiogenic Factor With G-Patch And FHA Domains 1	Protein Coding	41	GC05P077029	6.694956
TRIP13	Thyroid Hormone Receptor Interactor 13	Protein Coding	41	GC05P000892	6.609565
MYH1	Myosin Heavy Chain 1	Protein Coding	41	GC17M010492	6.592595
PIBF1	Progesterone Immunomodulatory Binding Factor 1	Protein Coding	41	GC13P072782	6.545411
CCBE1	Collagen And Calcium Binding EGF Domains 1	Protein Coding	41	GC18M059430	6.542602
SNX10	Sorting Nexin 10	Protein Coding	41	GC07P026291	6.50098
RNASE3	Ribonuclease A Family Member 3	Protein Coding	41	GC14P020891	6.464322
NDUFC2	NADH:Ubiquinone Oxidoreductase Subunit C2	Protein Coding	41	GC11M078068	6.384011
IFNA2	Interferon Alpha 2	Protein Coding	41	GC09M021384	6.305368
SRSF2	Serine And Arginine Rich Splicing Factor 2	Protein Coding	41	GC17M076734	6.291948
LRBA	LPS Responsive Beige-Like Anchor Protein	Protein Coding	41	GC04M150264	6.283139
FLAD1	Flavin Adenine Dinucleotide Synthetase 1	Protein Coding	41	GC01P154983	6.251727
HLA-DPA1	Major Histocompatibility Complex, Class II, DP Alpha 1	Protein Coding	41	GC06M033064	6.245814
DPM3	Dolichyl-Phosphate Mannosyltransferase Subunit 3, Regulatory	Protein Coding	41	GC01M155112	6.178913
EBF3	EBF Transcription Factor 3	Protein Coding	41	GC10M129835	6.119553
APOC1	Apolipoprotein C1	Protein Coding	41	GC19P044914	6.044149
CEP55	Centrosomal Protein 55	Protein Coding	41	GC10P093496	6.030347
CCL4	C-C Motif Chemokine Ligand 4	Protein Coding	41	GC17P036103	5.94773
NUP93	Nucleoporin 93	Protein Coding	41	GC16P056769	5.904356
COG4	Component Of Oligomeric Golgi Complex 4	Protein Coding	41	GC16M070482	5.897601
TIMP4	TIMP Metallopeptidase Inhibitor 4	Protein Coding	41	GC03M012153	5.892622
EXOSC8	Exosome Component 8	Protein Coding	41	GC13P036998	5.704188
NRTN	Neurturin	Protein Coding	41	GC19P005805	5.679829
IL11	Interleukin 11	Protein Coding	41	GC19M055364	5.659267
TAF6	TATA-Box Binding Protein Associated Factor 6	Protein Coding	41	GC07M100107	5.638702
EDA	Ectodysplasin A	Protein Coding	41	GC0XP069618	5.632435
SLC35A2	Solute Carrier Family 35 Member A2	Protein Coding	41	GC0XM048903	5.630281
LEMD3	LEM Domain Containing 3	Protein Coding	41	GC12P065169	5.611306
LRIG2	Leucine Rich Repeats And Immunoglobulin Like Domains 2	Protein Coding	41	GC01P113073	5.608316
NUP85	Nucleoporin 85	Protein Coding	41	GC17P075205	5.581138
ATP11A	ATPase Phospholipid Transporting 11A	Protein Coding	41	GC13P112690	5.566597
WDR26	WD Repeat Domain 26	Protein Coding	41	GC01M224385	5.531725
PIGN	Phosphatidylinositol Glycan Anchor Biosynthesis Class N	Protein Coding	41	GC18M061905	5.524626
FBXO32	F-Box Protein 32	Protein Coding	41	GC08M123498	5.472401
TSPYL1	TSPY Like 1	Protein Coding	41	GC06M116268	5.460422
FSTL1	Follistatin Like 1	Protein Coding	41	GC03M120392	5.402993
NTS	Neurotensin	Protein Coding	41	GC12P085876	5.390988
PORCN	Porcupine O-Acyltransferase	Protein Coding	41	GC0XP048514	5.385214
AKAP6	A-Kinase Anchoring Protein 6	Protein Coding	41	GC14P032203	5.353431
CNOT1	CCR4-NOT Transcription Complex Subunit 1	Protein Coding	41	GC16M058519	5.336243
UNC13A	Unc-13 Homolog A	Protein Coding	41	GC19M017603	5.267994
GDF3	Growth Differentiation Factor 3	Protein Coding	41	GC12M007689	5.218441
IFT88	Intraflagellar Transport 88	Protein Coding	41	GC13P020566	5.187069
HIC1	HIC ZBTB Transcriptional Repressor 1	Protein Coding	41	GC17P002054	5.183829
CXCR6	C-X-C Motif Chemokine Receptor 6	Protein Coding	41	GC03P045982	5.169983
PDLIM1	PDZ And LIM Domain 1	Protein Coding	41	GC10M095237	5.161254
CALB2	Calbindin 2	Protein Coding	41	GC16P071392	5.15872
LMO7	LIM Domain 7	Protein Coding	41	GC13P075620	5.10566
UPF3B	UPF3B Regulator Of Nonsense Mediated MRNA Decay	Protein Coding	41	GC0XM119805	5.047145
MCF2	MCF.2 Cell Line Derived Transforming Sequence	Protein Coding	41	GC0XM139581	5.037611

FGF16	Fibroblast Growth Factor 16	Protein Coding	41	GC0XP077447	5.0021
NACC1	Nucleus Accumbens Associated 1	Protein Coding	41	GC19P013117	4.985538
NISCH	Nischarin	Protein Coding	41	GC03P052455	4.958227
SIM1	SIM BHLH Transcription Factor 1	Protein Coding	41	GC06M100386	4.905994
MCAM	Melanoma Cell Adhesion Molecule	Protein Coding	41	GC11M119308	4.894961
COL13A1	Collagen Type XIII Alpha 1 Chain	Protein Coding	41	GC10P069801	4.887111
CD69	CD69 Molecule	Protein Coding	41	GC12M015678	4.886405
RPS16	Ribosomal Protein S16	Protein Coding	41	GC19M039433	4.87712
CYP8B1	Cytochrome P450 Family 8 Subfamily B Member 1	Protein Coding	41	GC03M042856	4.844114
DPH1	Diphthamide Biosynthesis 1	Protein Coding	41	GC17P002030	4.831176
ADAMTS3	ADAM Metallopeptidase With Thrombospondin Type 1 Motif 3	Protein Coding	41	GC04M072280	4.791508
FKBP10	FKBP Prolyl Isomerase 10	Protein Coding	41	GC17P041812	4.774031
BICD2	BICD Cargo Adaptor 2	Protein Coding	41	GC09M092711	4.753612
DPP9	Dipeptidyl Peptidase 9	Protein Coding	41	GC19M004675	4.72597
PCGF2	Polycomb Group Ring Finger 2	Protein Coding	41	GC17M038733	4.721138
CHD8	Chromodomain Helicase DNA Binding Protein 8	Protein Coding	41	GC14M021385	4.701269
CRTAP	Cartilage Associated Protein	Protein Coding	41	GC03P033113	4.697422
GFM2	GTP Dependent Ribosome Recycling Factor Mitochondrial 2	Protein Coding	41	GC05M074721	4.649105
RAMP3	Receptor Activity Modifying Protein 3	Protein Coding	41	GC07P045163	4.590551
CLN5	CLN5 Intracellular Trafficking Protein	Protein Coding	41	GC13P076990	4.585827
HLA-DRB5	Major Histocompatibility Complex, Class II, DR Beta 5	Protein Coding	41	GC06M046926	4.582555
RUNX1T1	RUNX1 Partner Transcriptional Co-Repressor 1	Protein Coding	41	GC08M091954	4.578281
PMP22	Peripheral Myelin Protein 22	Protein Coding	41	GC17M015229	4.560465
ZNF148	Zinc Finger Protein 148	Protein Coding	41	GC03M125225	4.560269
DNAJB11	DnaJ Heat Shock Protein Family (Hsp40) Member B11	Protein Coding	41	GC03P186567	4.545767
ALG14	ALG14 UDP-N-Acetylglucosaminyltransferase Subunit	Protein Coding	41	GC01M094974	4.53475
SP110	SP110 Nuclear Body Protein	Protein Coding	41	GC02M230167	4.520805
ATAD1	ATPase Family AAA Domain Containing 1	Protein Coding	41	GC10M087751	4.456655
ABLIM1	Actin Binding LIM Protein 1	Protein Coding	41	GC10M114462	4.454599
RNF135	Ring Finger Protein 135	Protein Coding	41	GC17P030974	4.445644
DEGS1	Delta 4-Desaturase, Sphingolipid 1	Protein Coding	41	GC01P224175	4.442341
FIBP	FGF1 Intracellular Binding Protein	Protein Coding	41	GC11M069430	4.438021
FDX1	Ferredoxin 1	Protein Coding	41	GC11P110429	4.436577
TBCK	TBC1 Domain Containing Kinase	Protein Coding	41	GC04M106041	4.426788
D2HGDH	D-2-Hydroxyglutarate Dehydrogenase	Protein Coding	41	GC02P241734	4.413744
GRP	Gastrin Releasing Peptide	Protein Coding	41	GC18P059220	4.413229
SPATA5	Spermatogenesis Associated 5	Protein Coding	41	GC04P122923	4.410235
CDCA7L	Cell Division Cycle Associated 7 Like	Protein Coding	41	GC07M021900	4.380106
GRHL2	Grainyhead Like Transcription Factor 2	Protein Coding	41	GC08P101492	4.324091
BCAP31	B Cell Receptor Associated Protein 31	Protein Coding	41	GC0XM153701	4.282543
SCN7A	Sodium Voltage-Gated Channel Alpha Subunit 7	Protein Coding	41	GC02M166403	4.259873
CAMTA1	Calmodulin Binding Transcription Activator 1	Protein Coding	41	GC01P006806	4.228063
ANKH	ANKH Inorganic Pyrophosphate Transport Regulator	Protein Coding	41	GC05M014706	4.214299
RAB18	RAB18, Member RAS Oncogene Family	Protein Coding	41	GC10P027504	4.205029
UPF1	UPF1 RNA Helicase And ATPase	Protein Coding	41	GC19P018831	4.165004
CBY1	Chibby Family Member 1, Beta Catenin Antagonist	Protein Coding	41	GC22P038656	4.164512
FMOD	Fibromodulin	Protein Coding	41	GC01M203340	4.154638
TRAK1	Trafficking Kinesin Protein 1	Protein Coding	41	GC03P042016	4.146272
LIMS2	LIM Zinc Finger Domain Containing 2	Protein Coding	41	GC02M127638	4.140094
STC1	Stanniocalcin 1	Protein Coding	41	GC08M023841	4.128607
TERF2	Telomeric Repeat Binding Factor 2	Protein Coding	41	GC16M069355	4.121716
CLASP1	Cytoplasmic Linker Associated Protein 1	Protein Coding	41	GC02M121337	4.109975
CLN8	CLN8 Transmembrane ER And ERGIC Protein	Protein Coding	41	GC08P001755	4.096019

DHH	Desert Hedgehog Signaling Molecule	Protein Coding	41	GC12M049099	4.087865
ADAMTS17	ADAM Metallopeptidase With Thrombospondin Type 1 Motif 17	Protein Coding	41	GC15M099971	4.072352
DOCK3	Dedicator Of Cytokinesis 3	Protein Coding	41	GC03P050675	4.056298
IFRD1	Interferon Related Developmental Regulator 1	Protein Coding	41	GC07P112422	4.054091
TBCD	Tubulin Folding Cofactor D	Protein Coding	41	GC17P082752	4.049587
PPIC	Peptidylprolyl Isomerase C	Protein Coding	41	GC05M123023	4.037521
TTPA	Alpha Tocopherol Transfer Protein	Protein Coding	41	GC08M063048	4.023755
NDUFS5	NADH:Ubiquinone Oxidoreductase Subunit S5	Protein Coding	41	GC01P039026	4.0006
NUP133	Nucleoporin 133	Protein Coding	41	GC01M229441	3.994017
MYT1L	Myelin Transcription Factor 1 Like	Protein Coding	41	GC02M001789	3.987035
CADPS	Calcium Dependent Secretion Activator	Protein Coding	41	GC03M062398	3.952084
GCA	Grancalcin	Protein Coding	41	GC02P162318	3.95039
COQ5	Coenzyme Q5, Methyltransferase	Protein Coding	41	GC12M120503	3.950363
PIWIL1	Piwi Like RNA-Mediated Gene Silencing 1	Protein Coding	41	GC12P130337	3.944241
ADD2	Adducin 2	Protein Coding	41	GC02M070626	3.918452
STX11	Syntaxin 11	Protein Coding	41	GC06P144150	3.915989
ZBTB20	Zinc Finger And BTB Domain Containing 20	Protein Coding	41	GC03M114315	3.910722
ORC6	Origin Recognition Complex Subunit 6	Protein Coding	41	GC16P046689	3.907607
DDX4	DEAD-Box Helicase 4	Protein Coding	41	GC05P055738	3.898043
SLC25A22	Solute Carrier Family 25 Member 22	Protein Coding	41	GC11M001138	3.882081
AEBP1	AE Binding Protein 1	Protein Coding	41	GC07P044106	3.871586
TSEN2	TRNA Splicing Endonuclease Subunit 2	Protein Coding	41	GC03P012484	3.85692
UGT2B4	UDP Glucuronosyltransferase Family 2 Member B4	Protein Coding	41	GC04M069484	3.848104
SCAP	SREBF Chaperone	Protein Coding	41	GC03M047413	3.826256
LIMS1	LIM Zinc Finger Domain Containing 1	Protein Coding	41	GC02P108534	3.82572
PROP1	PROP Paired-Like Homeobox 1	Protein Coding	41	GC05M177992	3.814843
CELSR2	Cadherin EGF LAG Seven-Pass G-Type Receptor 2	Protein Coding	41	GC01P109250	3.802171
CCNF	Cyclin F	Protein Coding	41	GC16P002429	3.795988
RFX6	Regulatory Factor X6	Protein Coding	41	GC06P116877	3.747215
EIF6	Eukaryotic Translation Initiation Factor 6	Protein Coding	41	GC20M035278	3.74291
MID2	Midline 2	Protein Coding	41	GC0XP107825	3.737477
MED13	Mediator Complex Subunit 13	Protein Coding	41	GC17M061942	3.73686
SMOC1	SPARC Related Modular Calcium Binding 1	Protein Coding	41	GC14P069854	3.735777
CARS2	Cysteinyl-TRNA Synthetase 2, Mitochondrial	Protein Coding	41	GC13M110641	3.734596
VPS13A	Vacuolar Protein Sorting 13 Homolog A	Protein Coding	41	GC09P077177	3.724144
GADD45G	Growth Arrest And DNA Damage Inducible Gamma	Protein Coding	41	GC09P089605	3.715075
ULK4	Unc-51 Like Kinase 4	Protein Coding	41	GC03M041247	3.714417
CLTCL1	Clathrin Heavy Chain Like 1	Protein Coding	41	GC22M019180	3.712183
SIL1	SIL1 Nucleotide Exchange Factor	Protein Coding	41	GC05M138957	3.709874
HLX	H2.0 Like Homeobox	Protein Coding	41	GC01P220879	3.702824
DIO2	Iodothyronine Deiodinase 2	Protein Coding	41	GC14M080197	3.699992
RAB8A	RAB8A, Member RAS Oncogene Family	Protein Coding	41	GC19P016111	3.660326
MRPS28	Mitochondrial Ribosomal Protein S28	Protein Coding	41	GC08M079925	3.647931
SMARCA5	SWI/SNF Related, Matrix Associated, Actin Dependent Regulator Of Chromatin, Subfamily A, Member 5	Protein Coding	41	GC04P143513	3.642514
PRELP	Proline And Arginine Rich End Leucine Rich Repeat Protein	Protein Coding	41	GC01P203475	3.60346
BRPF1	Bromodomain And PHD Finger Containing 1	Protein Coding	41	GC03P009731	3.601465
SLC27A1	Solute Carrier Family 27 Member 1	Protein Coding	41	GC19P026639	3.59961
LDHAL6B	Lactate Dehydrogenase A Like 6B	Protein Coding	41	GC15P059206	3.587557
INSL3	Insulin Like 3	Protein Coding	41	GC19M017816	3.584214
SETD1A	SET Domain Containing 1A, Histone Lysine Methyltransferase	Protein Coding	41	GC16P032486	3.574986
MED17	Mediator Complex Subunit 17	Protein Coding	41	GC11P093784	3.559859
NTNG1	Netrin G1	Protein Coding	41	GC01P107140	3.55866
SHARPIN	SHANK Associated RH Domain Interactor	Protein Coding	41	GC08M144098	3.538062

MORC2	MORC Family CW-Type Zinc Finger 2	Protein Coding	41	GC22M030925	3.521695
ST8SIA4	ST8 Alpha-N-Acetyl-Neuraminide Alpha-2,8-Sialyltransferase 4	Protein Coding	41	GC05M100806	3.518319
KLF2	Kruppel Like Factor 2	Protein Coding	41	GC19P026614	3.510535
KDSR	3-Ketodihydrosphingosine Reductase	Protein Coding	41	GC18M063327	3.510212
APIP	APAF1 Interacting Protein	Protein Coding	41	GC11M034854	3.501614
INSIG2	Insulin Induced Gene 2	Protein Coding	41	GC02P118088	3.490397
SCARF2	Scavenger Receptor Class F Member 2	Protein Coding	41	GC22M020424	3.486374
PITX3	Paired Like Homeodomain 3	Protein Coding	41	GC10M102230	3.477988
AGO2	Argonaute RISC Catalytic Component 2	Protein Coding	41	GC08M140522	3.463456
GPR161	G Protein-Coupled Receptor 161	Protein Coding	41	GC01M168080	3.412977
PCSK5	Proprotein Convertase Subtilisin/Kexin Type 5	Protein Coding	41	GC09P075890	3.40686
PCSK2	Proprotein Convertase Subtilisin/Kexin Type 2	Protein Coding	41	GC20P017226	3.396117
SPRED2	Sprouty Related EVH1 Domain Containing 2	Protein Coding	41	GC02M065307	3.39125
IGSF1	Immunoglobulin Superfamily Member 1	Protein Coding	41	GC0XM131273	3.35401
STK17A	Serine/Threonine Kinase 17a	Protein Coding	41	GC07P043582	3.346904
BLVRB	Biliverdin Reductase B	Protein Coding	41	GC19M040447	3.343952
BOC	BOC Cell Adhesion Associated, Oncogene Regulated	Protein Coding	41	GC03P113211	3.338528
GPM6A	Glycoprotein M6A	Protein Coding	41	GC04M175632	3.333766
MYF5	Myogenic Factor 5	Protein Coding	41	GC12P080716	3.3089
PMM1	Phosphomannomutase 1	Protein Coding	41	GC22M041576	3.30845
NEO1	Neogenin 1	Protein Coding	41	GC15P073051	3.281571
PXDN	Peroxidasin	Protein Coding	41	GC02M001635	3.278356
FLVCR1	FLVCR Heme Transporter 1	Protein Coding	41	GC01P212858	3.264646
PDE4DIP	Phosphodiesterase 4D Interacting Protein	Protein Coding	41	GC01P148808	3.255345
TPM4	Tropomyosin 4	Protein Coding	41	GC19P026610	3.25201
ATOH7	Atonal BHLH Transcription Factor 7	Protein Coding	41	GC10M068230	3.251446
SH3BP2	SH3 Domain Binding Protein 2	Protein Coding	41	GC04P002794	3.221598
TBC1D1	TBC1 Domain Family Member 1	Protein Coding	41	GC04P037893	3.214906
TOMM40	Translocase Of Outer Mitochondrial Membrane 40	Protein Coding	41	GC19P044890	3.204642
SLC25A32	Solute Carrier Family 25 Member 32	Protein Coding	41	GC08M103398	3.193847
UBE4B	Ubiquitination Factor E4B	Protein Coding	41	GC01P010032	3.190914
WDFY3	WD Repeat And FYVE Domain Containing 3	Protein Coding	41	GC04M084669	3.18987
EDIL3	EGF Like Repeats And Discoidin Domains 3	Protein Coding	41	GC05M083940	3.174853
SIX2	SIX Homeobox 2	Protein Coding	41	GC02M045005	3.170981
KLRK1	Killer Cell Lectin Like Receptor K1	Protein Coding	41	GC12M015686	3.169229
ICA1	Islet Cell Autoantigen 1	Protein Coding	41	GC07M008119	3.16637
SUZ12	SUZ12 Polycomb Repressive Complex 2 Subunit	Protein Coding	41	GC17P031937	3.162788
PNPLA3	Patatin Like Phospholipase Domain Containing 3	Protein Coding	41	GC22P043923	3.162208
TULP3	TUB Like Protein 3	Protein Coding	41	GC12P002870	3.130789
TET3	Tet Methylcytosine Dioxygenase 3	Protein Coding	41	GC02P073986	3.129038
F13B	Coagulation Factor XIII B Chain	Protein Coding	41	GC01M197008	3.115613
MDC1	Mediator Of DNA Damage Checkpoint 1	Protein Coding	41	GC06M046797	3.113198
EXOC4	Exocyst Complex Component 4	Protein Coding	41	GC07P133253	3.109764
SPIB	Spi-B Transcription Factor	Protein Coding	41	GC19P050418	3.103874
TRAIP	TRAF Interacting Protein	Protein Coding	41	GC03M050057	3.098196
KCNIP1	Potassium Voltage-Gated Channel Interacting Protein 1	Protein Coding	41	GC05P170353	3.0918
ECI2	Enoyl-CoA Delta Isomerase 2	Protein Coding	41	GC06M004115	3.091261
PEMT	Phosphatidylethanolamine N-Methyltransferase	Protein Coding	41	GC17M017506	3.087906
SMPD2	Sphingomyelin Phosphodiesterase 2	Protein Coding	41	GC06P109440	3.085734
STRN	Striatin	Protein Coding	41	GC02M036815	3.080163
GDF11	Growth Differentiation Factor 11	Protein Coding	41	GC12P055743	3.06442
MCPH1	Microcephalin 1	Protein Coding	41	GC08P006406	3.064357
ATF7	Activating Transcription Factor 7	Protein Coding	41	GC12M053527	3.061885

GREM2	Gremlin 2, DAN Family BMP Antagonist	Protein Coding	41	GC01M240489	3.061176
WDR45	WD Repeat Domain 45	Protein Coding	41	GC0XM049074	3.055333
DEK	DEK Proto-Oncogene	Protein Coding	41	GC06M018224	3.04733
CDK20	Cyclin Dependent Kinase 20	Protein Coding	41	GC09M087966	3.039978
B3GALNT2	Beta-1,3-N-Acetylgalactosaminyltransferase 2	Protein Coding	41	GC01M235440	3.02346
NUP210	Nucleoporin 210	Protein Coding	41	GC03M016910	3.007493
PPP2R5A	Protein Phosphatase 2 Regulatory Subunit B'Alpha	Protein Coding	41	GC01P212285	2.98442
TRIM71	Tripartite Motif Containing 71	Protein Coding	41	GC03P032859	2.963795
AGXT2	Alanine--Glyoxylate Aminotransferase 2	Protein Coding	41	GC05M034998	2.95211
PAWR	Pro-Apoptotic WT1 Regulator	Protein Coding	41	GC12M079574	2.947282
PCSK6	Proprotein Convertase Subtilisin/Kexin Type 6	Protein Coding	41	GC15M107497	2.94427
DKK3	Dickkopf WNT Signaling Pathway Inhibitor 3	Protein Coding	41	GC11M011962	2.908558
MYOF	Myoferlin	Protein Coding	41	GC10M093306	2.902813
RPS18	Ribosomal Protein S18	Protein Coding	41	GC06P055262	2.895546
KANK1	KN Motif And Ankyrin Repeat Domains 1	Protein Coding	41	GC09P000474	2.887519
CXCL5	C-X-C Motif Chemokine Ligand 5	Protein Coding	41	GC04M073995	2.883241
ADI1	Acireductone Dioxygenase 1	Protein Coding	41	GC02M003501	2.876615
RENBP	Renin Binding Protein	Protein Coding	41	GC0XM153935	2.851201
DYRK3	Dual Specificity Tyrosine Phosphorylation Regulated Kinase 3	Protein Coding	41	GC01P206636	2.842779
NFIL3	Nuclear Factor, Interleukin 3 Regulated	Protein Coding	41	GC09M091409	2.839188
MACF1	Microtubule Actin Crosslinking Factor 1	Protein Coding	41	GC01P039082	2.82585
AGPAT4	1-Acylglycerol-3-Phosphate O-Acyltransferase 4	Protein Coding	41	GC06M161129	2.818216
PCMT1	Protein-L-Isoaspartate (D-Aspartate) O-Methyltransferase	Protein Coding	41	GC06P149749	2.809403
MAVS	Mitochondrial Antiviral Signaling Protein	Protein Coding	41	GC20P003850	2.801651
FOLR2	Folate Receptor Beta	Protein Coding	41	GC11P072216	2.797347
CLCF1	Cardiotrophin Like Cytokine Factor 1	Protein Coding	41	GC11M067364	2.796585
DHX16	DEAH-Box Helicase 16	Protein Coding	41	GC06M030653	2.791343
MTMR4	Myotubularin Related Protein 4	Protein Coding	41	GC17M058489	2.785777
RAD9A	RAD9 Checkpoint Clamp Component A	Protein Coding	41	GC11P067318	2.783491
ONECUT1	One Cut Homeobox 1	Protein Coding	41	GC15M067272	2.782818
ANAPC1	Anaphase Promoting Complex Subunit 1	Protein Coding	41	GC02M111611	2.780989
LHX3	LIM Homeobox 3	Protein Coding	41	GC09M136196	2.77601
PHB2	Prohibitin 2	Protein Coding	41	GC12M006965	2.772238
EBF1	EBF Transcription Factor 1	Protein Coding	41	GC05M158695	2.769814
CAPZB	Capping Actin Protein Of Muscle Z-Line Subunit Beta	Protein Coding	41	GC01M019339	2.767661
HSPE1	Heat Shock Protein Family E (Hsp10) Member 1	Protein Coding	41	GC02P197501	2.753918
BHMT2	Betaine--Homocysteine S-Methyltransferase 2	Protein Coding	41	GC05P079071	2.753463
SIPA1	Signal-Induced Proliferation-Associated 1	Protein Coding	41	GC11P065638	2.753153
LIN7A	Lin-7 Homolog A, Crumbs Cell Polarity Complex Component	Protein Coding	41	GC12M080792	2.752194
NFIC	Nuclear Factor I C	Protein Coding	41	GC19P003314	2.749523
ATP8A2	ATPase Phospholipid Transporting 8A2	Protein Coding	41	GC13P025373	2.748604
CD1C	CD1c Molecule	Protein Coding	41	GC01P158289	2.740785
GJB4	Gap Junction Protein Beta 4	Protein Coding	41	GC01P034759	2.734353
ASH1L	ASH1 Like Histone Lysine Methyltransferase	Protein Coding	41	GC01M155335	2.727309
TREM1	Triggering Receptor Expressed On Myeloid Cells 1	Protein Coding	41	GC06M041267	2.72728
PSMC4	Proteasome 26S Subunit, ATPase 4	Protein Coding	41	GC19P040529	2.725082
REEP5	Receptor Accessory Protein 5	Protein Coding	41	GC05M112876	2.724073
PHF6	PHD Finger Protein 6	Protein Coding	41	GC0XP134373	2.702299
ZFYVE16	Zinc Finger FYVE-Type Containing 16	Protein Coding	41	GC05P080407	2.700368
USP22	Ubiquitin Specific Peptidase 22	Protein Coding	41	GC17M020999	2.695438
GFI1	Growth Factor Independent 1 Transcriptional Repressor	Protein Coding	41	GC01M092474	2.693318
RHD	Rh Blood Group D Antigen	Protein Coding	41	GC01P025272	2.692035
SERPINB7	Serpin Family B Member 7	Protein Coding	41	GC18P063752	2.687873

GYG2	Glycogenin 2	Protein Coding	41	GC0XP002828	2.684029
HSD17B12	Hydroxysteroid 17-Beta Dehydrogenase 12	Protein Coding	41	GC11P043660	2.676858
GALNT1	Polypeptide N-Acetylgalactosaminyltransferase 1	Protein Coding	41	GC18P035581	2.674933
BCAM	Basal Cell Adhesion Molecule (Lutheran Blood Group)	Protein Coding	41	GC19P044810	2.669136
SUCNR1	Succinate Receptor 1	Protein Coding	41	GC03P151873	2.666822
HMGN1	High Mobility Group Nucleosome Binding Domain 1	Protein Coding	41	GC21M039342	2.666523
NUP153	Nucleoporin 153	Protein Coding	41	GC06M017615	2.656658
RHCE	Rh Blood Group CcEe Antigens	Protein Coding	41	GC01M025360	2.651588
SLC17A6	Solute Carrier Family 17 Member 6	Protein Coding	41	GC11P022359	2.64986
TIMM17A	Translocase Of Inner Mitochondrial Membrane 17A	Protein Coding	41	GC01P201955	2.634912
ZNF41	Zinc Finger Protein 41	Protein Coding	41	GC0XM047444	2.634119
CD5	CD5 Molecule	Protein Coding	41	GC11P061114	2.633167
POGLUT1	Protein O-Glucosyltransferase 1	Protein Coding	41	GC03P119468	2.618879
KCNH8	Potassium Voltage-Gated Channel Subfamily H Member 8	Protein Coding	41	GC03P019165	2.61318
FUCA2	Alpha-L-Fucosidase 2	Protein Coding	41	GC06M143494	2.594543
POLR2L	RNA Polymerase II, I And III Subunit L	Protein Coding	41	GC11M001144	2.583117
CLP1	Cleavage Factor Polyribonucleotide Kinase Subunit 1	Protein Coding	41	GC11P057648	2.576742
MBD2	Methyl-CpG Binding Domain Protein 2	Protein Coding	41	GC18M054151	2.574279
CLPX	Caseinolytic Mitochondrial Matrix Peptidase Chaperone Subunit X	Protein Coding	41	GC15M065148	2.570574
ANO1	Anoctamin 1	Protein Coding	41	GC11P069986	2.566151
PPP1R15A	Protein Phosphatase 1 Regulatory Subunit 15A	Protein Coding	41	GC19P048872	2.549433
TRAPPC4	Trafficking Protein Particle Complex Subunit 4	Protein Coding	41	GC11P119018	2.547791
SLC30A1	Solute Carrier Family 30 Member 1	Protein Coding	41	GC01M211571	2.541512
LLGL1	LLGL Scribble Cell Polarity Complex Component 1	Protein Coding	41	GC17P018225	2.538727
ALDH1L1	Aldehyde Dehydrogenase 1 Family Member L1	Protein Coding	41	GC03M126103	2.536461
GCM2	Glial Cells Missing Transcription Factor 2	Protein Coding	41	GC06M010873	2.531529
TNFAIP6	TNF Alpha Induced Protein 6	Protein Coding	41	GC02P151357	2.51895
CD1B	CD1b Molecule	Protein Coding	41	GC01M158297	2.510236
ABCD2	ATP Binding Cassette Subfamily D Member 2	Protein Coding	41	GC12M039530	2.502024
NRG2	Neuregulin 2	Protein Coding	41	GC05M139810	2.4999
UFM1	Ubiquitin Fold Modifier 1	Protein Coding	41	GC13P038349	2.497715
NTM	Neurotrimin	Protein Coding	41	GC11P131370	2.494747
KIRREL2	Kirre Like Nephrin Family Adhesion Molecule 2	Protein Coding	41	GC19P040371	2.489701
CCNC	Cyclin C	Protein Coding	41	GC06M099542	2.487818
HUS1	HUS1 Checkpoint Clamp Component	Protein Coding	41	GC07M047991	2.486726
RNF41	Ring Finger Protein 41	Protein Coding	41	GC12M056202	2.483864
MPZL1	Myelin Protein Zero Like 1	Protein Coding	41	GC01P167721	2.479838
PSMC6	Proteasome 26S Subunit, ATPase 6	Protein Coding	41	GC14P052707	2.471464
POLR1B	RNA Polymerase I Subunit B	Protein Coding	41	GC02P116682	2.470038
ASGR2	Asialoglycoprotein Receptor 2	Protein Coding	41	GC17M007101	2.462379
AGPAT3	1-Acylglycerol-3-Phosphate O-Acyltransferase 3	Protein Coding	41	GC21P043865	2.461885
RECQL	RecQ Like Helicase	Protein Coding	41	GC12M021468	2.459135
RASSF5	Ras Association Domain Family Member 5	Protein Coding	41	GC01P206507	2.445328
POLD3	DNA Polymerase Delta 3, Accessory Subunit	Protein Coding	41	GC11P074526	2.443209
SETMAR	SET Domain And Mariner Transposase Fusion Gene	Protein Coding	41	GC03P004303	2.441427
SHB	SH2 Domain Containing Adaptor Protein B	Protein Coding	41	GC09M038348	2.422335
SLC2A13	Solute Carrier Family 2 Member 13	Protein Coding	41	GC12M039755	2.41975
HERPUD1	Homocysteine Inducible ER Protein With Ubiquitin Like Domain 1	Protein Coding	41	GC16P056931	2.411633
NKX2-2	NK2 Homeobox 2	Protein Coding	41	GC20M021511	2.401984
SLC13A4	Solute Carrier Family 13 Member 4	Protein Coding	41	GC07M135681	2.395584
ATP1A4	ATPase Na+/K+ Transporting Subunit Alpha 4	Protein Coding	41	GC01P160151	2.394476
RAB6A	RAB6A, Member RAS Oncogene Family	Protein Coding	41	GC11M073676	2.394443
MCHR1	Melanin Concentrating Hormone Receptor 1	Protein Coding	41	GC22P040679	2.393994

MAGI1	Membrane Associated Guanylate Kinase, WW And PDZ Domain Containing 1	Protein Coding	41	GC03M065330	2.392512
RPS15	Ribosomal Protein S15	Protein Coding	41	GC19P001438	2.390974
BCL2A1	BCL2 Related Protein A1	Protein Coding	41	GC15M079961	2.390136
OLIG2	Oligodendrocyte Transcription Factor 2	Protein Coding	41	GC21P033025	2.381922
DCLK2	Doublecortin Like Kinase 2	Protein Coding	41	GC04P150078	2.375844
RPLP1	Ribosomal Protein Lateral Stalk Subunit P1	Protein Coding	41	GC15P077111	2.373097
INCENP	Inner Centromere Protein	Protein Coding	41	GC11P062142	2.372909
ATOX1	Antioxidant 1 Copper Chaperone	Protein Coding	41	GC05M151743	2.371395
NBEAL2	Neurobeachin Like 2	Protein Coding	41	GC03P046979	2.362885
TSN	Translin	Protein Coding	41	GC02P121737	2.355481
ARHGAP29	Rho GTPase Activating Protein 29	Protein Coding	41	GC01M094148	2.354271
NDUFB4	NADH:Ubiquinone Oxidoreductase Subunit B4	Protein Coding	41	GC03P120596	2.346928
CAPN6	Calpain 6	Protein Coding	41	GC0XM111245	2.344916
KIF5C	Kinesin Family Member 5C	Protein Coding	41	GC02P148875	2.339563
MOCS1	Molybdenum Cofactor Synthesis 1	Protein Coding	41	GC06M039899	2.335753
SKAP2	Src Kinase Associated Phosphoprotein 2	Protein Coding	41	GC07M026654	2.321721
MAT2B	Methionine Adenosyltransferase 2B	Protein Coding	41	GC05P163504	2.305022
PPA1	Inorganic Pyrophosphatase 1	Protein Coding	41	GC10M070202	2.29841
RBM4	RNA Binding Motif Protein 4	Protein Coding	41	GC11P066638	2.295989
SH3BP4	SH3 Domain Binding Protein 4	Protein Coding	41	GC02P234951	2.295574
CD1E	CD1e Molecule	Protein Coding	41	GC01P158354	2.295389
APEH	Acylaminoacyl-Peptide Hydrolase	Protein Coding	41	GC03P049673	2.291235
DBNL	Drebrin Like	Protein Coding	41	GC07P044044	2.291214
UIMC1	Ubiquitin Interaction Motif Containing 1	Protein Coding	41	GC05M176905	2.273669
TBX19	T-Box Transcription Factor 19	Protein Coding	41	GC01P168280	2.270819
AAK1	AP2 Associated Kinase 1	Protein Coding	41	GC02M069459	2.262439
DIO3	Iodothyronine Deiodinase 3	Protein Coding	41	GC14P106101	2.262369
USP18	Ubiquitin Specific Peptidase 18	Protein Coding	41	GC22P018149	2.261055
KRT6B	Keratin 6B	Protein Coding	41	GC12M052446	2.251414
GPR55	G Protein-Coupled Receptor 55	Protein Coding	41	GC02M230907	2.251086
COQ3	Coenzyme Q3, Methyltransferase	Protein Coding	41	GC06M099369	2.243931
GIT2	GIT ArfGAP 2	Protein Coding	41	GC12M109929	2.241765
BRD1	Bromodomain Containing 1	Protein Coding	41	GC22M049773	2.239884
TAF2	TATA-Box Binding Protein Associated Factor 2	Protein Coding	41	GC08M119730	2.237911
NLRX1	NLR Family Member X1	Protein Coding	41	GC11P119166	2.235436
SIGLEC1	Sialic Acid Binding Ig Like Lectin 1	Protein Coding	41	GC20M003686	2.23488
CACNA2D3	Calcium Voltage-Gated Channel Auxiliary Subunit Alpha2delta 3	Protein Coding	41	GC03P054156	2.229909
RCN2	Reticulocalbin 2	Protein Coding	41	GC15P076931	2.228472
GORASP1	Golgi Reassembly Stacking Protein 1	Protein Coding	41	GC03M039096	2.226494
PPY	Pancreatic Polypeptide	Protein Coding	41	GC17M043940	2.221364
KCNV2	Potassium Voltage-Gated Channel Modifier Subfamily V Member 2	Protein Coding	41	GC09P002717	2.219254
HLA-DMA	Major Histocompatibility Complex, Class II, DM Alpha	Protein Coding	41	GC06M046951	2.210461
NEK4	NIMA Related Kinase 4	Protein Coding	41	GC03M052708	2.209382
LSM2	LSM2 Homolog, U6 Small Nuclear RNA And MRNA Degradation Associated	Protein Coding	41	GC06M046897	2.201715
TRIOBP	TRIO And F-Actin Binding Protein	Protein Coding	41	GC22P037696	2.196589
IP6K1	Inositol Hexakisphosphate Kinase 1	Protein Coding	41	GC03M050062	2.192721
DSG4	Desmoglein 4	Protein Coding	41	GC18P031377	2.192676
GOLGA2	Golgin A2	Protein Coding	41	GC09M128255	2.192365
DNAJC3	DnaJ Heat Shock Protein Family (Hsp40) Member C3	Protein Coding	41	GC13P095677	2.191274
POLA2	DNA Polymerase Alpha 2, Accessory Subunit	Protein Coding	41	GC11P065315	2.190677
NAB2	NGFI-A Binding Protein 2	Protein Coding	41	GC12P057088	2.187266
L2HGDH	L-2-Hydroxyglutarate Dehydrogenase	Protein Coding	41	GC14M050237	2.169642
UBA52	Ubiquitin A-52 Residue Ribosomal Protein Fusion Product 1	Protein Coding	41	GC19P018563	2.169424

OXR1	Oxidation Resistance 1	Protein Coding	41	GC08P106271	2.166753
RFX2	Regulatory Factor X2	Protein Coding	41	GC19M005993	2.163565
MOV10	Mov10 RISC Complex RNA Helicase	Protein Coding	41	GC01P112673	2.156821
TFF3	Trefoil Factor 3	Protein Coding	41	GC21M042311	2.147146
KLRB1	Killer Cell Lectin Like Receptor B1	Protein Coding	41	GC12M015675	2.14109
CLDN11	Claudin 11	Protein Coding	41	GC03P170418	2.137875
RSAD2	Radical S-Adenosyl Methionine Domain Containing 2	Protein Coding	41	GC02P006865	2.132085
MVP	Major Vault Protein	Protein Coding	41	GC16P032377	2.121015
REG1A	Regenerating Family Member 1 Alpha	Protein Coding	41	GC02P079120	2.120419
CPEB1	Cytoplasmic Polyadenylation Element Binding Protein 1	Protein Coding	41	GC15M082543	2.118382
CEP63	Centrosomal Protein 63	Protein Coding	41	GC03P134485	2.115982
HS6ST2	Heparan Sulfate 6-O-Sulfotransferase 2	Protein Coding	41	GC0XM132626	2.109273
MCM3AP	Minichromosome Maintenance Complex Component 3 Associated Protein	Protein Coding	41	GC21M046235	2.102434
EME1	Essential Meiotic Structure-Specific Endonuclease 1	Protein Coding	41	GC17P050373	2.09389
CCR9	C-C Motif Chemokine Receptor 9	Protein Coding	41	GC03P045903	2.093791
AGAP1	ArfGAP With GTPase Domain, Ankyrin Repeat And PH Domain 1	Protein Coding	41	GC02P235494	2.091871
DNAL4	Dynein Axonemal Light Chain 4	Protein Coding	41	GC22M038778	2.088382
CDH17	Cadherin 17	Protein Coding	41	GC08M094127	2.084294
CXCL2	C-X-C Motif Chemokine Ligand 2	Protein Coding	41	GC04M074097	2.080524
KIF13B	Kinesin Family Member 13B	Protein Coding	41	GC08M029067	2.073838
NTNG2	Netrin G2	Protein Coding	41	GC09P132161	2.073634
LPXN	Leupaxin	Protein Coding	41	GC11M069097	2.069616
F2RL2	Coagulation Factor II Thrombin Receptor Like 2	Protein Coding	41	GC05M076615	2.064604
REEP1	Receptor Accessory Protein 1	Protein Coding	41	GC02M086213	2.059685
HS2ST1	Heparan Sulfate 2-O-Sulfotransferase 1	Protein Coding	41	GC01P086914	2.052034
KIR3DL1	Killer Cell Immunoglobulin Like Receptor, Three Ig Domains And Long Cytoplasmic Tail 1	Protein Coding	41	GC19P056447	2.051767
BCL3	BCL3 Transcription Coactivator	Protein Coding	41	GC19P044747	2.048348
STAP1	Signal Transducing Adaptor Family Member 1	Protein Coding	41	GC04P067558	2.043535
MLNR	Motilin Receptor	Protein Coding	41	GC13P049220	2.041173
LDHD	Lactate Dehydrogenase D	Protein Coding	41	GC16M075111	2.031698
GRID1	Glutamate Ionotropic Receptor Delta Type Subunit 1	Protein Coding	41	GC10M085599	2.030835
RAD51D	RAD51 Paralog D	Protein Coding	41	GC17M035092	2.029753
COL27A1	Collagen Type XXVII Alpha 1 Chain	Protein Coding	41	GC09P115748	2.024703
HLA-DOA	Major Histocompatibility Complex, Class II, DO Alpha	Protein Coding	41	GC06M033004	2.020452
GGA3	Golgi Associated, Gamma Adaptin Ear Containing, ARF Binding Protein 3	Protein Coding	41	GC17M075225	2.016376
KLRC1	Killer Cell Lectin Like Receptor C1	Protein Coding	41	GC12M015692	2.015644
CEACAM3	CEA Cell Adhesion Molecule 3	Protein Coding	41	GC19P041796	2.00562
MCC	MCC Regulator Of WNT Signaling Pathway	Protein Coding	41	GC05M113022	2.003416
CBX1	Chromobox 1	Protein Coding	41	GC17M048070	1.997302
GNL3	G Protein Nucleolar 3	Protein Coding	41	GC03P052681	1.993948
GLMN	Glomulin, FKBP Associated Protein	Protein Coding	41	GC01M092246	1.990072
GTF2H4	General Transcription Factor IIH Subunit 4	Protein Coding	41	GC06P055185	1.988373
FARSA	Phenylalanyl-TRNA Synthetase Subunit Alpha	Protein Coding	41	GC19M012922	1.986812
TMC1	Transmembrane Channel Like 1	Protein Coding	41	GC09P072521	1.984144
NCAPD2	Non-SMC Condensin I Complex Subunit D2	Protein Coding	41	GC12P006493	1.98401
SMPD3	Sphingomyelin Phosphodiesterase 3	Protein Coding	41	GC16M068358	1.977681
AK4	Adenylate Kinase 4	Protein Coding	41	GC01P065147	1.968537
HDLBP	High Density Lipoprotein Binding Protein	Protein Coding	41	GC02M241227	1.966463
DDX17	DEAD-Box Helicase 17	Protein Coding	41	GC22M038483	1.962933
BBC3	BCL2 Binding Component 3	Protein Coding	41	GC19M047220	1.961185
TP53RK	TP53 Regulating Kinase	Protein Coding	41	GC20M046684	1.948168
KDM4A	Lysine Demethylase 4A	Protein Coding	41	GC01P043650	1.947461
CRYBB3	Crystallin Beta B3	Protein Coding	41	GC22P026738	1.942741

C1GALT1	Core 1 Synthase, Glycoprotein-N-Acetylgalactosamine 3-Beta-Galactosyltransferase 1	Protein Coding	41	GC07P007156	1.942117
PLEKHG5	Pleckstrin Homology And RhoGEF Domain Containing G5	Protein Coding	41	GC01M006466	1.931427
ELMO2	Engulfment And Cell Motility 2	Protein Coding	41	GC20M046366	1.927966
SLC44A2	Solute Carrier Family 44 Member 2	Protein Coding	41	GC19P010602	1.924488
TCN1	Transcobalamin 1	Protein Coding	41	GC11M069142	1.922647
PRDM2	PR/SET Domain 2	Protein Coding	41	GC01P013755	1.92164
PHC1	Polyhomeotic Homolog 1	Protein Coding	41	GC12P008913	1.921154
KATNB1	Katanin Regulatory Subunit B1	Protein Coding	41	GC16P057735	1.919603
GPR17	G Protein-Coupled Receptor 17	Protein Coding	41	GC02P127645	1.91204
AKAP12	A-Kinase Anchoring Protein 12	Protein Coding	41	GC06P151239	1.911343
WDR1	WD Repeat Domain 1	Protein Coding	41	GC04M010075	1.909024
RPS23	Ribosomal Protein S23	Protein Coding	41	GC05M082273	1.907645
CLCA1	Chloride Channel Accessory 1	Protein Coding	41	GC01P086468	1.903008
G3BP1	G3BP Stress Granule Assembly Factor 1	Protein Coding	41	GC05P151771	1.901859
PLIN3	Perilipin 3	Protein Coding	41	GC19M004839	1.898731
CHM	CHM Rab Escort Protein	Protein Coding	41	GC0XM085861	1.897352
SLC22A11	Solute Carrier Family 22 Member 11	Protein Coding	41	GC11P064573	1.890286
ARFGAP3	ADP Ribosylation Factor GTPase Activating Protein 3	Protein Coding	41	GC22M042796	1.890168
STK16	Serine/Threonine Kinase 16	Protein Coding	41	GC02P219248	1.889902
PRSS12	Serine Protease 12	Protein Coding	41	GC04M118280	1.884284
SORBS3	Sorbin And SH3 Domain Containing 3	Protein Coding	41	GC08P022544	1.881846
FGL1	Fibrinogen Like 1	Protein Coding	41	GC08M017864	1.872157
RHBDF2	Rhomboid 5 Homolog 2	Protein Coding	41	GC17M076470	1.865701
PPIL2	Peptidylprolyl Isomerase Like 2	Protein Coding	41	GC22P027198	1.862579
GABPA	GA Binding Protein Transcription Factor Subunit Alpha	Protein Coding	41	GC21P025734	1.858689
RECK	Reversion Inducing Cysteine Rich Protein With Kazal Motifs	Protein Coding	41	GC09P036036	1.856888
CCDC88A	Coiled-Coil Domain Containing 88A	Protein Coding	41	GC02M055287	1.847725
APCS	Amyloid P Component, Serum	Protein Coding	41	GC01P159587	1.846187
CDS1	CDP-Diacylglycerol Synthase 1	Protein Coding	41	GC04P084582	1.845874
FOXD3	Forkhead Box D3	Protein Coding	41	GC01P063323	1.841787
PRG2	Proteoglycan 2, Pro Eosinophil Major Basic Protein	Protein Coding	41	GC11M057386	1.832964
ARAP3	ArfGAP With RhoGAP Domain, Ankyrin Repeat And PH Domain 3	Protein Coding	41	GC05M141653	1.829503
SDCBP	Syndecan Binding Protein	Protein Coding	41	GC08P058539	1.828465
SRGAP3	SLIT-ROBO Rho GTPase Activating Protein 3	Protein Coding	41	GC03M008980	1.825403
C4BPB	Complement Component 4 Binding Protein Beta	Protein Coding	41	GC01P207088	1.820567
SLC39A7	Solute Carrier Family 39 Member 7	Protein Coding	41	GC06P033200	1.814923
HSD17B8	Hydroxysteroid 17-Beta Dehydrogenase 8	Protein Coding	41	GC06P055257	1.810839
MYO3B	Myosin IIIB	Protein Coding	41	GC02P170178	1.802432
NPSR1	Neuropeptide S Receptor 1	Protein Coding	41	GC07P034664	1.801421
CDK14	Cyclin Dependent Kinase 14	Protein Coding	41	GC07P090471	1.796878
SLC35A1	Solute Carrier Family 35 Member A1	Protein Coding	41	GC06P087470	1.794108
PA2G4	Proliferation-Associated 2G4	Protein Coding	41	GC12P056372	1.793495
PGLYRP1	Peptidoglycan Recognition Protein 1	Protein Coding	41	GC19M047013	1.790857
PCDH19	Protocadherin 19	Protein Coding	41	GC0XM100291	1.790575
MSLN	Mesothelin	Protein Coding	41	GC16P005518	1.788268
SS18	SS18 Subunit Of BAF Chromatin Remodeling Complex	Protein Coding	41	GC18M026016	1.787967
ACSL3	Acyl-CoA Synthetase Long Chain Family Member 3	Protein Coding	41	GC02P222860	1.781419
TPPP	Tubulin Polymerization Promoting Protein	Protein Coding	41	GC05M000659	1.77804
HOMER1	Homer Scaffold Protein 1	Protein Coding	41	GC05M079372	1.775204
CLDN3	Claudin 3	Protein Coding	41	GC07M073768	1.773499
SCTR	Secretin Receptor	Protein Coding	41	GC02M119439	1.767524
SUPT3H	SPT3 Homolog, SAGA And STAGA Complex Component	Protein Coding	41	GC06M047166	1.766038
C4BPA	Complement Component 4 Binding Protein Alpha	Protein Coding	41	GC01P207105	1.761252

TBPL1	TATA-Box Binding Protein Like 1	Protein Coding	41	GC06P133952	1.759984
SLC6A14	Solute Carrier Family 6 Member 14	Protein Coding	41	GC0XP116436	1.757751
TIAL1	TIA1 Cytotoxic Granule Associated RNA Binding Protein Like 1	Protein Coding	41	GC10M119571	1.757419
DHX9	DExH-Box Helicase 9	Protein Coding	41	GC01P182839	1.746494
GUCA1B	Guanylate Cyclase Activator 1B	Protein Coding	41	GC06M047114	1.746466
TAX1BP1	Tax1 Binding Protein 1	Protein Coding	41	GC07P027739	1.742556
SNRPA	Small Nuclear Ribonucleoprotein Polypeptide A	Protein Coding	41	GC19P040750	1.74252
RPS6KC1	Ribosomal Protein S6 Kinase C1	Protein Coding	41	GC01P213051	1.738279
ANAPC2	Anaphase Promoting Complex Subunit 2	Protein Coding	41	GC09M137174	1.736365
ANGPTL1	Angiopoietin Like 1	Protein Coding	41	GC01M178818	1.73403
RPL27A	Ribosomal Protein L27a	Protein Coding	41	GC11P008682	1.733035
RIOK2	RIO Kinase 2	Protein Coding	41	GC05M097160	1.731277
FCAR	Fc Fragment Of IgA Receptor	Protein Coding	41	GC19P056455	1.731081
NKX6-1	NK6 Homeobox 1	Protein Coding	41	GC04M084491	1.729486
CYB561	Cytochrome B561	Protein Coding	41	GC17M063432	1.721108
TACC1	Transforming Acidic Coiled-Coil Containing Protein 1	Protein Coding	41	GC08P038728	1.713714
UBA6	Ubiquitin Like Modifier Activating Enzyme 6	Protein Coding	41	GC04M067612	1.710427
ARHGEF9	Cdc42 Guanine Nucleotide Exchange Factor 9	Protein Coding	41	GC0XM063634	1.709784
SSRP1	Structure Specific Recognition Protein 1	Protein Coding	41	GC11M069072	1.709244
STC2	Stanniocalcin 2	Protein Coding	41	GC05M173314	1.700171
CXCL11	C-X-C Motif Chemokine Ligand 11	Protein Coding	41	GC04M076033	1.69764
PEG10	Paternally Expressed 10	Protein Coding	41	GC07P094656	1.691223
ZP1	Zona Pellucida Glycoprotein 1	Protein Coding	41	GC11P060867	1.689579
SART1	Spliceosome Associated Factor 1, Recruiter Of U4/U6.U5 Tri-SnRNP	Protein Coding	41	GC11P066273	1.686729
CIB2	Calcium And Integrin Binding Family Member 2	Protein Coding	41	GC15M078104	1.68349
PPP1R14A	Protein Phosphatase 1 Regulatory Inhibitor Subunit 14A	Protein Coding	41	GC19M038251	1.67509
TLX1	T Cell Leukemia Homeobox 1	Protein Coding	41	GC10P101130	1.669576
MARCO	Macrophage Receptor With Collagenous Structure	Protein Coding	41	GC02P118942	1.668671
MSMB	Microseminoprotein Beta	Protein Coding	41	GC10M046033	1.665042
TSC22D1	TSC22 Domain Family Member 1	Protein Coding	41	GC13M044432	1.663192
CRYGS	Crystallin Gamma S	Protein Coding	41	GC03M186538	1.648834
BCLAF1	BCL2 Associated Transcription Factor 1	Protein Coding	41	GC06M136256	1.648264
IGF2BP1	Insulin Like Growth Factor 2 MRNA Binding Protein 1	Protein Coding	41	GC17P048997	1.643557
NFE2	Nuclear Factor, Erythroid 2	Protein Coding	41	GC12M054292	1.6427
ARHGAP6	Rho GTPase Activating Protein 6	Protein Coding	41	GC0XM011137	1.641236
POLQ	DNA Polymerase Theta	Protein Coding	41	GC03M121431	1.63634
THG1L	TRNA-Histidine Guanylyltransferase 1 Like	Protein Coding	41	GC05P157731	1.631218
STEAP2	STEAP2 Metalloreductase	Protein Coding	41	GC07P090167	1.6242
SSR1	Signal Sequence Receptor Subunit 1	Protein Coding	41	GC06M007268	1.620107
SLC24A5	Solute Carrier Family 24 Member 5	Protein Coding	41	GC15P048120	1.607274
HNRNPF	Heterogeneous Nuclear Ribonucleoprotein F	Protein Coding	41	GC10M043385	1.603731
USP6	Ubiquitin Specific Peptidase 6	Protein Coding	41	GC17P005116	1.603326
TES	Testin LIM Domain Protein	Protein Coding	41	GC07P116210	1.600321
HOXC13	Homeobox C13	Protein Coding	41	GC12P053938	1.600096
ANGPT4	Angiopoietin 4	Protein Coding	41	GC20M000869	1.595174
CHD5	Chromodomain Helicase DNA Binding Protein 5	Protein Coding	41	GC01M006104	1.593739
UBE2E1	Ubiquitin Conjugating Enzyme E2 E1	Protein Coding	41	GC03P023805	1.592813
CLIC4	Chloride Intracellular Channel 4	Protein Coding	41	GC01P024745	1.586472
OAS3	2'-5'-Oligoadenylate Synthetase 3	Protein Coding	41	GC12P112938	1.58182
PPP2R5E	Protein Phosphatase 2 Regulatory Subunit B'Epsilon	Protein Coding	41	GC14M063371	1.579501
BNIP2	BCL2 Interacting Protein 2	Protein Coding	41	GC15M059659	1.571729
PUS7	Pseudouridine Synthase 7	Protein Coding	41	GC07M105439	1.567715
TRAPPC6B	Trafficking Protein Particle Complex Subunit 6B	Protein Coding	41	GC14M039147	1.564551

UBE2D4	Ubiquitin Conjugating Enzyme E2 D4 (Putative)	Protein Coding	41	GC07P043926	1.559558
PTPN23	Protein Tyrosine Phosphatase Non-Receptor Type 23	Protein Coding	41	GC03P047397	1.546515
SLC5A11	Solute Carrier Family 5 Member 11	Protein Coding	41	GC16P024859	1.544463
WIPI2	WD Repeat Domain, Phosphoinositide Interacting 2	Protein Coding	41	GC07P005190	1.53918
ABCB10	ATP Binding Cassette Subfamily B Member 10	Protein Coding	41	GC01M229516	1.538395
SCGB3A2	Secretoglobin Family 3A Member 2	Protein Coding	41	GC05P147870	1.53497
RAB14	RAB14, Member RAS Oncogene Family	Protein Coding	41	GC09M121178	1.529082
B3GALT4	Beta-1,3-Galactosyltransferase 4	Protein Coding	41	GC06P033277	1.524655
ARAP1	ArfGAP With RhoGAP Domain, Ankyrin Repeat And PH Domain 1	Protein Coding	41	GC11M072686	1.523311
CDK12	Cyclin Dependent Kinase 12	Protein Coding	41	GC17P039461	1.522285
ASF1B	Anti-Silencing Function 1B Histone Chaperone	Protein Coding	41	GC19M014119	1.520064
SNRPD2	Small Nuclear Ribonucleoprotein D2 Polypeptide	Protein Coding	41	GC19M046999	1.518112
CLDN6	Claudin 6	Protein Coding	41	GC16M003014	1.512067
AP1M1	Adaptor Related Protein Complex 1 Subunit Mu 1	Protein Coding	41	GC19P026613	1.511436
GK2	Glycerol Kinase 2	Protein Coding	41	GC04M079406	1.510645
CDC42BPB	CDC42 Binding Protein Kinase Beta	Protein Coding	41	GC14M102932	1.505262
UBE2O	Ubiquitin Conjugating Enzyme E2 O	Protein Coding	41	GC17M076389	1.500455
MPP1	Membrane Palmitoylated Protein 1	Protein Coding	41	GC0XM154779	1.492947
CRYGD	Crystallin Gamma D	Protein Coding	41	GC02M208121	1.491575
CLPS	Colipase	Protein Coding	41	GC06M047029	1.488302
ATP6V1D	ATPase H+ Transporting V1 Subunit D	Protein Coding	41	GC14M067294	1.487888
CTDSP1	CTD Small Phosphatase 1	Protein Coding	41	GC02P218398	1.482611
PGAP1	Post-GPI Attachment To Proteins Inositol Deacylase 1	Protein Coding	41	GC02M196833	1.479507
INPP5J	Inositol Polyphosphate-5-Phosphatase J	Protein Coding	41	GC22P031134	1.478091
SGMS1	Sphingomyelin Synthase 1	Protein Coding	41	GC10M050305	1.475833
CDSN	Corneodesmosin	Protein Coding	41	GC06M031115	1.474788
CCL28	C-C Motif Chemokine Ligand 28	Protein Coding	41	GC05M043356	1.465371
GALNT13	Polypeptide N-Acetylgalactosaminyltransferase 13	Protein Coding	41	GC02P153871	1.464011
CNOT8	CCR4-NOT Transcription Complex Subunit 8	Protein Coding	41	GC05P154859	1.460303
AS3MT	Arsenite Methyltransferase	Protein Coding	41	GC10P102869	1.45917
SRSF9	Serine And Arginine Rich Splicing Factor 9	Protein Coding	41	GC12M120461	1.452701
VSNL1	Visinin Like 1	Protein Coding	41	GC02P017539	1.450018
PHLPP1	PH Domain And Leucine Rich Repeat Protein Phosphatase 1	Protein Coding	41	GC18P062715	1.448652
LAMTOR2	Late Endosomal/Lysosomal Adaptor, MAPK And MTOR Activator 2	Protein Coding	41	GC01P156054	1.448417
EHF	ETS Homologous Factor	Protein Coding	41	GC11P034621	1.443485
RNF4	Ring Finger Protein 4	Protein Coding	41	GC04P002462	1.436526
UGT1A10	UDP Glucuronosyltransferase Family 1 Member A10	Protein Coding	41	GC02P233636	1.433249
HLA-DOB	Major Histocompatibility Complex, Class II, DO Beta	Protein Coding	41	GC06M046944	1.429296
TRAPPC3	Trafficking Protein Particle Complex Subunit 3	Protein Coding	41	GC01M036136	1.423777
CPA4	Carboxypeptidase A4	Protein Coding	41	GC07P130293	1.416049
ST3GAL6	ST3 Beta-Galactoside Alpha-2,3-Sialyltransferase 6	Protein Coding	41	GC03P098732	1.41499
ACOX3	Acyl-CoA Oxidase 3, Pristanoyl	Protein Coding	41	GC04M008380	1.413535
NME6	NME/NM23 Nucleoside Diphosphate Kinase 6	Protein Coding	41	GC03M048292	1.410983
HOXB7	Homeobox B7	Protein Coding	41	GC17M048607	1.410863
ANO10	Anoctamin 10	Protein Coding	41	GC03M043355	1.408672
NUP88	Nucleoporin 88	Protein Coding	41	GC17M005451	1.407591
CRYGC	Crystallin Gamma C	Protein Coding	41	GC02M208128	1.39987
DIO1	Iodothyronine Deiodinase 1	Protein Coding	41	GC01P053891	1.399194
IL19	Interleukin 19	Protein Coding	41	GC01P206770	1.392892
CACNG4	Calcium Voltage-Gated Channel Auxiliary Subunit Gamma 4	Protein Coding	41	GC17P066964	1.390825
CCT2	Chaperonin Containing TCP1 Subunit 2	Protein Coding	41	GC12P069585	1.387347
REEP2	Receptor Accessory Protein 2	Protein Coding	41	GC05P138439	1.3867
IL27RA	Interleukin 27 Receptor Subunit Alpha	Protein Coding	41	GC19P014031	1.38503

LGALS3BP	Galectin 3 Binding Protein	Protein Coding	41	GC17M078971	1.383774
OPN4	Opsin 4	Protein Coding	41	GC10P086654	1.38146
CLK2	CDC Like Kinase 2	Protein Coding	41	GC01M155262	1.373775
TNFSF14	TNF Superfamily Member 14	Protein Coding	41	GC19M006663	1.37187
MAPK8IP3	Mitogen-Activated Protein Kinase 8 Interacting Protein 3	Protein Coding	41	GC16P001706	1.371503
HMGB3	High Mobility Group Box 3	Protein Coding	41	GC0XP150980	1.371073
COIL	Coilin	Protein Coding	41	GC17M056938	1.363018
SPAG9	Sperm Associated Antigen 9	Protein Coding	41	GC17M050962	1.356961
FYCO1	FYVE And Coiled-Coil Domain Autophagy Adaptor 1	Protein Coding	41	GC03M045917	1.356683
RGMA	Repulsive Guidance Molecule BMP Co-Receptor A	Protein Coding	41	GC15M093035	1.356027
TFCP2	Transcription Factor CP2	Protein Coding	41	GC12M051093	1.354028
PRPF4B	Pre-MRNA Processing Factor 4B	Protein Coding	41	GC06P004021	1.350763
IFITM1	Interferon Induced Transmembrane Protein 1	Protein Coding	41	GC11P000313	1.347714
EPS15L1	Epidermal Growth Factor Receptor Pathway Substrate 15 Like 1	Protein Coding	41	GC19M016333	1.34673
AMOT	Angiomotin	Protein Coding	41	GC0XM112774	1.34235
MTDH	Metadherin	Protein Coding	41	GC08P097643	1.34156
EFHC1	EF-Hand Domain Containing 1	Protein Coding	41	GC06P052362	1.336632
NFKBIE	NFKB Inhibitor Epsilon	Protein Coding	41	GC06M044258	1.328537
UBE2K	Ubiquitin Conjugating Enzyme E2 K	Protein Coding	41	GC04P039700	1.324044
CKAP5	Cytoskeleton Associated Protein 5	Protein Coding	41	GC11M068983	1.318863
ELF4	E74 Like ETS Transcription Factor 4	Protein Coding	41	GC0XM130064	1.31812
IFITM3	Interferon Induced Transmembrane Protein 3	Protein Coding	41	GC11M000319	1.316184
RPL30	Ribosomal Protein L30	Protein Coding	41	GC08M098024	1.308842
ERP44	Endoplasmic Reticulum Protein 44	Protein Coding	41	GC09M099979	1.307721
ERLIN2	ER Lipid Raft Associated 2	Protein Coding	41	GC08P037736	1.306381
GSTM4	Glutathione S-Transferase Mu 4	Protein Coding	41	GC01P109657	1.299945
CKS2	CDC28 Protein Kinase Regulatory Subunit 2	Protein Coding	41	GC09P089311	1.29546
SFXN4	Sideroflexin 4	Protein Coding	41	GC10M119140	1.295146
STK25	Serine/Threonine Kinase 25	Protein Coding	41	GC02M241492	1.293015
OTOF	Otoferlin	Protein Coding	41	GC02M026458	1.291092
RAB3D	RAB3D, Member RAS Oncogene Family	Protein Coding	41	GC19M011322	1.283477
EIF2S2	Eukaryotic Translation Initiation Factor 2 Subunit Beta	Protein Coding	41	GC20M034088	1.28227
GALK2	Galactokinase 2	Protein Coding	41	GC15P049155	1.280595
STX7	Syntaxin 7	Protein Coding	41	GC06M132445	1.279448
ECT2	Epithelial Cell Transforming 2	Protein Coding	41	GC03P172750	1.278513
HOXA2	Homeobox A2	Protein Coding	41	GC07M027100	1.273245
SMG1	SMG1 Nonsense Mediated MRNA Decay Associated PI3K Related Kinase	Protein Coding	41	GC16M018805	1.270927
STX1B	Syntaxin 1B	Protein Coding	41	GC16M030989	1.270254
TOB1	Transducer Of ERBB2, 1	Protein Coding	41	GC17M050862	1.26482
CHMP1A	Charged Multivesicular Body Protein 1A	Protein Coding	41	GC16M089644	1.263805
SERPINA4	Serpin Family A Member 4	Protein Coding	41	GC14P094561	1.262635
PPL	Periplakin	Protein Coding	41	GC16M004872	1.262248
NT5C3A	5'-Nucleotidase, Cytosolic IIIA	Protein Coding	41	GC07M033014	1.262007
NR2C1	Nuclear Receptor Subfamily 2 Group C Member 1	Protein Coding	41	GC12M095022	1.259897
ASGR1	Asialoglycoprotein Receptor 1	Protein Coding	41	GC17M007173	1.256816
CILP	Cartilage Intermediate Layer Protein	Protein Coding	41	GC15M065194	1.256511
ETNK1	Ethanolamine Kinase 1	Protein Coding	41	GC12P022625	1.253401
MICAL1	Microtubule Associated Monooxygenase, Calponin And LIM Domain Containing 1	Protein Coding	41	GC06M109444	1.251918
LILRB2	Leukocyte Immunoglobulin Like Receptor B2	Protein Coding	41	GC19M054562	1.25119
RHOC	Ras Homolog Family Member C	Protein Coding	41	GC01M112701	1.249665
PI4K2A	Phosphatidylinositol 4-Kinase Type 2 Alpha	Protein Coding	41	GC10P097640	1.24942
CNGB3	Cyclic Nucleotide Gated Channel Subunit Beta 3	Protein Coding	41	GC08M086553	1.238498
PDIA6	Protein Disulfide Isomerase Family A Member 6	Protein Coding	41	GC02M010784	1.226113

SH3BP5	SH3 Domain Binding Protein 5	Protein Coding	41	GC03M016936	1.223502
SLC38A4	Solute Carrier Family 38 Member 4	Protein Coding	41	GC12M046764	1.222766
NMT2	N-Myristoyltransferase 2	Protein Coding	41	GC10M015115	1.222521
CHST15	Carbohydrate Sulfotransferase 15	Protein Coding	41	GC10M124006	1.219729
ALDH1L2	Aldehyde Dehydrogenase 1 Family Member L2	Protein Coding	41	GC12M105019	1.217255
BHLHE41	Basic Helix-Loop-Helix Family Member E41	Protein Coding	41	GC12M026120	1.214593
NMUR1	Neuromedin U Receptor 1	Protein Coding	41	GC02M231666	1.207899
EEF1E1	Eukaryotic Translation Elongation Factor 1 Epsilon 1	Protein Coding	41	GC06M008073	1.206245
ABCB9	ATP Binding Cassette Subfamily B Member 9	Protein Coding	41	GC12M122920	1.204619
GPR183	G Protein-Coupled Receptor 183	Protein Coding	41	GC13M099296	1.204117
FMO2	Flavin Containing Dimethylaniline Monoxygenase 2	Protein Coding	41	GC01P171185	1.203148
PSMC2	Proteasome 26S Subunit, ATPase 2	Protein Coding	41	GC07P103344	1.202656
DNAJA3	DnaJ Heat Shock Protein Family (Hsp40) Member A3	Protein Coding	41	GC16P004425	1.199546
CYFIP1	Cytoplasmic FMR1 Interacting Protein 1	Protein Coding	41	GC15M022867	1.19144
INMT	Indolethylamine N-Methyltransferase	Protein Coding	41	GC07P030737	1.186301
KCNMB4	Potassium Calcium-Activated Channel Subfamily M Regulatory Beta Subunit 4	Protein Coding	41	GC12P070369	1.185012
OASL	2'-5'-Oligoadenylate Synthetase Like	Protein Coding	41	GC12M121478	1.182966
LIN28A	Lin-28 Homolog A	Protein Coding	41	GC01P026410	1.18258
NCAN	Neurocan	Protein Coding	41	GC19P026662	1.177313
CSRP2	Cysteine And Glycine Rich Protein 2	Protein Coding	41	GC12M076859	1.17311
SOCS6	Suppressor Of Cytokine Signaling 6	Protein Coding	41	GC18P070288	1.171223
RCOR1	REST Corepressor 1	Protein Coding	41	GC14P102592	1.168403
PIGR	Polymeric Immunoglobulin Receptor	Protein Coding	41	GC01M206928	1.163443
ELOVL1	ELOVL Fatty Acid Elongase 1	Protein Coding	41	GC01M043363	1.162043
HOXD9	Homeobox D9	Protein Coding	41	GC02P176122	1.158767
CD72	CD72 Molecule	Protein Coding	41	GC09M035610	1.153704
DEFA1	Defensin Alpha 1	Protein Coding	41	GC08M006977	1.152977
PCLO	Piccolo Presynaptic Cytomatrix Protein	Protein Coding	41	GC07M082754	1.152275
DIS3	DIS3 Homolog, Exosome Endoribonuclease And 3'-5' Exoribonuclease	Protein Coding	41	GC13M072752	1.149323
DYNC1I1	Dynein Cytoplasmic 1 Intermediate Chain 1	Protein Coding	41	GC07P095772	1.146687
EFNA2	Ephrin A2	Protein Coding	41	GC19P001620	1.144571
KRT3	Keratin 3	Protein Coding	41	GC12M052789	1.141082
GDI2	GDP Dissociation Inhibitor 2	Protein Coding	41	GC10M005765	1.137742
GTSE1	G2 And S-Phase Expressed 1	Protein Coding	41	GC22P046296	1.136479
UBIAD1	UbiA Prenyltransferase Domain Containing 1	Protein Coding	41	GC01P011273	1.127294
CARD8	Caspase Recruitment Domain Family Member 8	Protein Coding	41	GC19M048204	1.125466
CHRNB3	Cholinergic Receptor Nicotinic Beta 3 Subunit	Protein Coding	41	GC08P042697	1.12074
ZNF331	Zinc Finger Protein 331	Protein Coding	41	GC19P053521	1.113948
HTATIP2	HIV-1 Tat Interactive Protein 2	Protein Coding	41	GC11P020363	1.112016
RAB11FIP2	RAB11 Family Interacting Protein 2	Protein Coding	41	GC10M118004	1.109541
TAGLN2	Transgelin 2	Protein Coding	41	GC01M159918	1.107269
BAG2	BAG Cochaperone 2	Protein Coding	41	GC06P057172	1.101346
RBBP7	RB Binding Protein 7, Chromatin Remodeling Factor	Protein Coding	41	GC0XM016839	1.100329
CCNT1	Cyclin T1	Protein Coding	41	GC12M048688	1.097878
NEDD8	NEDD8 Ubiquitin Like Modifier	Protein Coding	41	GC14M024216	1.096316
SLC7A14	Solute Carrier Family 7 Member 14	Protein Coding	41	GC03M170459	1.094828
CHFR	Checkpoint With Forkhead And Ring Finger Domains	Protein Coding	41	GC12M132822	1.092921
RAPGEF5	Rap Guanine Nucleotide Exchange Factor 5	Protein Coding	41	GC07M022128	1.084147
DPEP2	Dipeptidase 2	Protein Coding	41	GC16M067987	1.077632
STYK1	Serine/Threonine/Tyrosine Kinase 1	Protein Coding	41	GC12M015695	1.075005
NCAPH	Non-SMC Condensin I Complex Subunit H	Protein Coding	41	GC02P096365	1.072944
MFNG	MFNG O-Fucosylpeptide 3-Beta-N-Acetylglucosaminyltransferase	Protein Coding	41	GC22M037469	1.059163
PTGER1	Prostaglandin E Receptor 1	Protein Coding	41	GC19M014444	1.047546

GPR37L1	G Protein-Coupled Receptor 37 Like 1	Protein Coding	41	GC01P202122	1.047388
CRYBA1	Crystallin Beta A1	Protein Coding	41	GC17P029246	1.04702
CHAF1B	Chromatin Assembly Factor 1 Subunit B	Protein Coding	41	GC21P036385	1.040581
PSMD13	Proteasome 26S Subunit, Non-ATPase 13	Protein Coding	41	GC11P000236	1.038756
RAB8B	RAB8B, Member RAS Oncogene Family	Protein Coding	41	GC15P078704	1.037944
CDYL	Chromodomain Y Like	Protein Coding	41	GC06P004706	1.024213
RABGGTA	Rab Geranylgeranyltransferase Subunit Alpha	Protein Coding	41	GC14M024265	1.022987
KIF4A	Kinesin Family Member 4A	Protein Coding	41	GC0XP070290	1.021318
KLHL9	Kelch Like Family Member 9	Protein Coding	41	GC09M021329	1.013036
NSMAF	Neutral Sphingomyelinase Activation Associated Factor	Protein Coding	41	GC08M058569	1.00375
CIAO1	Cytosolic Iron-Sulfur Assembly Component 1	Protein Coding	41	GC02P096271	0.996297
COTL1	Coactosin Like F-Actin Binding Protein 1	Protein Coding	41	GC16M084566	0.996249
CLEC1B	C-Type Lectin Domain Family 1 Member B	Protein Coding	41	GC12M015681	0.995889
TUBA1C	Tubulin Alpha 1c	Protein Coding	41	GC12P049188	0.989382
HOXA7	Homeobox A7	Protein Coding	41	GC07M027153	0.984706
P4HA1	Prolyl 4-Hydroxylase Subunit Alpha 1	Protein Coding	41	GC10M073007	0.981653
KDM3A	Lysine Demethylase 3A	Protein Coding	41	GC02P086440	0.978272
CDCA8	Cell Division Cycle Associated 8	Protein Coding	41	GC01P037692	0.973824
AGAP2	ArfGAP With GTPase Domain, Ankyrin Repeat And PH Domain 2	Protein Coding	41	GC12M057723	0.971206
ADH6	Alcohol Dehydrogenase 6 (Class V)	Protein Coding	41	GC04M099202	0.965018
PLEKHG4	Pleckstrin Homology And RhoGEF Domain Containing G4	Protein Coding	41	GC16P067277	0.954972
OMG	Oligodendrocyte Myelin Glycoprotein	Protein Coding	41	GC17M031375	0.954315
CTSV	Cathepsin V	Protein Coding	41	GC09M097029	0.953056
CPA3	Carboxypeptidase A3	Protein Coding	41	GC03P148865	0.950546
CXXC1	CXXC Finger Protein 1	Protein Coding	41	GC18M050282	0.947637
ACOT7	Acyl-CoA Thioesterase 7	Protein Coding	41	GC01M006265	0.94644
KRT12	Keratin 12	Protein Coding	41	GC17M040861	0.943415
CDC14B	Cell Division Cycle 14B	Protein Coding	41	GC09M096490	0.937231
TRIM2	Tripartite Motif Containing 2	Protein Coding	41	GC04P153152	0.936382
SPTLC3	Serine Palmitoyltransferase Long Chain Base Subunit 3	Protein Coding	41	GC20P013008	0.933554
MBOAT7	Membrane Bound O-Acyltransferase Domain Containing 7	Protein Coding	41	GC19M054173	0.928984
HOMER3	Homer Scaffold Protein 3	Protein Coding	41	GC19M018933	0.925509
TCP1	T-Complex 1	Protein Coding	41	GC06M159778	0.924356
SLC8A2	Solute Carrier Family 8 Member A2	Protein Coding	41	GC19M047428	0.917503
WASF3	WASP Family Member 3	Protein Coding	41	GC13P026557	0.911581
BIK	BCL2 Interacting Killer	Protein Coding	41	GC22P043110	0.905867
EEF1G	Eukaryotic Translation Elongation Factor 1 Gamma	Protein Coding	41	GC11M069243	0.905079
CNOT7	CCR4-NOT Transcription Complex Subunit 7	Protein Coding	41	GC08M017224	0.896346
IL36RN	Interleukin 36 Receptor Antagonist	Protein Coding	41	GC02P116693	0.894391
ATP11B	ATPase Phospholipid Transporting 11B (Putative)	Protein Coding	41	GC03P182793	0.879791
ARL1	ADP Ribosylation Factor Like GTPase 1	Protein Coding	41	GC12M101393	0.874109
DNAJA2	DnaJ Heat Shock Protein Family (Hsp40) Member A2	Protein Coding	41	GC16M046955	0.866145
HKDC1	Hexokinase Domain Containing 1	Protein Coding	41	GC10P069220	0.863471
UBE3C	Ubiquitin Protein Ligase E3C	Protein Coding	41	GC07P157138	0.862536
PIGC	Phosphatidylinositol Glycan Anchor Biosynthesis Class C	Protein Coding	41	GC01M172339	0.862127
AMY2B	Amylase Alpha 2B	Protein Coding	41	GC01P103554	0.861378
CLEC4M	C-Type Lectin Domain Family 4 Member M	Protein Coding	41	GC19P007763	0.86113
ATP6V1F	ATPase H+ Transporting V1 Subunit F	Protein Coding	41	GC07P128862	0.858963
XPOT	Exportin For TRNA	Protein Coding	41	GC12P064404	0.85282
RARRES1	Retinoic Acid Receptor Responder 1	Protein Coding	41	GC03M158696	0.849438
B4GALT6	Beta-1,4-Galactosyltransferase 6	Protein Coding	41	GC18M031622	0.848628
APLP1	Amyloid Beta Precursor Like Protein 1	Protein Coding	41	GC19P040372	0.844296
CUX2	Cut Like Homeobox 2	Protein Coding	41	GC12P111034	0.839098

POLD2	DNA Polymerase Delta 2, Accessory Subunit	Protein Coding	41	GC07M044114	0.837079
ALDH3B2	Aldehyde Dehydrogenase 3 Family Member B2	Protein Coding	41	GC11M067662	0.833246
MAST1	Microtubule Associated Serine/Threonine Kinase 1	Protein Coding	41	GC19P012903	0.83233
ZC3H14	Zinc Finger CCCH-Type Containing 14	Protein Coding	41	GC14P088562	0.822501
AIFM2	Apoptosis Inducing Factor Mitochondria Associated 2	Protein Coding	41	GC10M070098	0.821008
HOXB4	Homeobox B4	Protein Coding	41	GC17M048575	0.81919
PCYT1B	Phosphate Cytidylyltransferase 1B, Choline	Protein Coding	41	GC0XM024576	0.816391
RGS7	Regulator Of G Protein Signaling 7	Protein Coding	41	GC01M240775	0.814273
MBD1	Methyl-CpG Binding Domain Protein 1	Protein Coding	41	GC18M050266	0.807315
NUDT2	Nudix Hydrolase 2	Protein Coding	41	GC09P034329	0.806708
TLR10	Toll Like Receptor 10	Protein Coding	41	GC04M038773	0.79833
ST6GALNAC1	ST6 N-Acetylgalactosaminide Alpha-2,6-Sialyltransferase 1	Protein Coding	41	GC17M076624	0.797215
DAPP1	Dual Adaptor Of Phosphotyrosine And 3-Phosphoinositides 1	Protein Coding	41	GC04P099816	0.793575
POU3F3	POU Class 3 Homeobox 3	Protein Coding	41	GC02P104855	0.78947
MSRB1	Methionine Sulfoxide Reductase B1	Protein Coding	41	GC16M001939	0.788629
HMGB2	High Mobility Group Box 2	Protein Coding	41	GC04M173331	0.77986
CLDN18	Claudin 18	Protein Coding	41	GC03P137998	0.774854
CYP39A1	Cytochrome P450 Family 39 Subfamily A Member 1	Protein Coding	41	GC06M046549	0.772574
RAE1	Ribonucleic Acid Export 1	Protein Coding	41	GC20P057351	0.771457
MYBL1	MYB Proto-Oncogene Like 1	Protein Coding	41	GC08M066562	0.765282
FUT1	Fucosyltransferase 1 (H Blood Group)	Protein Coding	41	GC19M048748	0.762691
C8G	Complement C8 Gamma Chain	Protein Coding	41	GC09P136944	0.762463
LSR	Lipolysis Stimulated Lipoprotein Receptor	Protein Coding	41	GC19P040343	0.762193
SLC17A3	Solute Carrier Family 17 Member 3	Protein Coding	41	GC06M025833	0.7556
KLK11	Kallikrein Related Peptidase 11	Protein Coding	41	GC19M051023	0.75187
TFF2	Trefoil Factor 2	Protein Coding	41	GC21M042346	0.751613
CDKN2D	Cyclin Dependent Kinase Inhibitor 2D	Protein Coding	41	GC19M010566	0.750448
SLC4A11	Solute Carrier Family 4 Member 11	Protein Coding	41	GC20M003230	0.75013
TEP1	Telomerase Associated Protein 1	Protein Coding	41	GC14M020365	0.746506
GABRR2	Gamma-Aminobutyric Acid Type A Receptor Subunit Rho2	Protein Coding	41	GC06M089257	0.742546
AP1G1	Adaptor Related Protein Complex 1 Subunit Gamma 1	Protein Coding	41	GC16M071729	0.739455
SLC44A4	Solute Carrier Family 44 Member 4	Protein Coding	41	GC06M031863	0.739267
SLC9A2	Solute Carrier Family 9 Member A2	Protein Coding	41	GC02P102620	0.736826
CMKLR1	Chemerin Chemokine-Like Receptor 1	Protein Coding	41	GC12M108288	0.732612
VNN2	Vanin 2	Protein Coding	41	GC06M132743	0.731926
CAP1	Cyclase Associated Actin Cytoskeleton Regulatory Protein 1	Protein Coding	41	GC01P040050	0.727863
NR2F6	Nuclear Receptor Subfamily 2 Group F Member 6	Protein Coding	41	GC19M017231	0.717475
STXBP3	Syntaxin Binding Protein 3	Protein Coding	41	GC01P108746	0.711501
CIDEA	Cell Death Inducing DFFA Like Effector A	Protein Coding	41	GC18P012254	0.705353
DAD1	Defender Against Cell Death 1	Protein Coding	41	GC14M022565	0.705264
IL13RA2	Interleukin 13 Receptor Subunit Alpha 2	Protein Coding	41	GC0XM115003	0.701663
TNPO1	Transportin 1	Protein Coding	41	GC05P072816	0.694789
CSE1L	Chromosome Segregation 1 Like	Protein Coding	41	GC20P049046	0.692065
PLCD3	Phospholipase C Delta 3	Protein Coding	41	GC17M045108	0.691961
CD6	CD6 Molecule	Protein Coding	41	GC11P060971	0.691541
CADM3	Cell Adhesion Molecule 3	Protein Coding	41	GC01P159141	0.690736
TNFAIP1	TNF Alpha Induced Protein 1	Protein Coding	41	GC17P028335	0.68736
NLN	Neurolysin	Protein Coding	41	GC05P065722	0.687277
SPINT1	Serine Peptidase Inhibitor, Kunitz Type 1	Protein Coding	41	GC15P040844	0.683832
SH2D2A	SH2 Domain Containing 2A	Protein Coding	41	GC01M156807	0.680495
GNG5	G Protein Subunit Gamma 5	Protein Coding	41	GC01M084498	0.677821
POLR2H	RNA Polymerase II, I And III Subunit H	Protein Coding	41	GC03P184361	0.676896
SLC39A5	Solute Carrier Family 39 Member 5	Protein Coding	41	GC12P056420	0.662784

ATP6V1C1	ATPase H+ Transporting V1 Subunit C1	Protein Coding	41	GC08P103038	0.653553
RBP3	Retinol Binding Protein 3	Protein Coding	41	GC10P047348	0.645742
PSMD10	Proteasome 26S Subunit, Non-ATPase 10	Protein Coding	41	GC0XM108084	0.627368
HAS3	Hyaluronan Synthase 3	Protein Coding	41	GC16P069105	0.621757
SV2B	Synaptic Vesicle Glycoprotein 2B	Protein Coding	41	GC15P091099	0.611148
CHDH	Choline Dehydrogenase	Protein Coding	41	GC03M053812	0.610606
CAPG	Capping Actin Protein, Gelsolin Like	Protein Coding	41	GC02M085394	0.604802
ETNK2	Ethanolamine Kinase 2	Protein Coding	41	GC01M204100	0.594112
RFC5	Replication Factor C Subunit 5	Protein Coding	41	GC12P118013	0.577311
NCBP2	Nuclear Cap Binding Protein Subunit 2	Protein Coding	41	GC03M196935	0.576147
CHRDL1	Chordin Like 1	Protein Coding	41	GC0XM110674	0.574667
ARF5	ADP Ribosylation Factor 5	Protein Coding	41	GC07P127588	0.554396
HGFAC	HGF Activator	Protein Coding	41	GC04P003443	0.536036
PRSS2	Serine Protease 2	Protein Coding	41	GC07P145798	0.514205
ARPC1A	Actin Related Protein 2/3 Complex Subunit 1A	Protein Coding	41	GC07P099325	0.503059
S100A7	S100 Calcium Binding Protein A7	Protein Coding	41	GC01M153457	0.481928
CPA2	Carboxypeptidase A2	Protein Coding	41	GC07P130266	0.475467
TCERG1	Transcription Elongation Regulator 1	Protein Coding	41	GC05P146447	0.474219
RTN2	Reticulon 2	Protein Coding	41	GC19M045485	0.453817
B3GNT5	UDP-GlcNAc:BetaGal Beta-1,3-N-Acetylglucosaminyltransferase 5	Protein Coding	41	GC03P183253	0.451734
ARHGEF4	Rho Guanine Nucleotide Exchange Factor 4	Protein Coding	41	GC02P130836	0.446108
GPR1	G Protein-Coupled Receptor 1	Protein Coding	41	GC02M206175	0.444152
MNDA	Myeloid Cell Nuclear Differentiation Antigen	Protein Coding	41	GC01P158801	0.394457
CYB5R4	Cytochrome B5 Reductase 4	Protein Coding	41	GC06P083859	0.376766
AGR2	Anterior Gradient 2, Protein Disulphide Isomerase Family Member	Protein Coding	41	GC07M016892	0.359139
CEACAM6	CEA Cell Adhesion Molecule 6	Protein Coding	41	GC19P041750	0.359139
HTR1E	5-Hydroxytryptamine Receptor 1E	Protein Coding	41	GC06P086937	0.358924
LAIR1	Leukocyte Associated Immunoglobulin Like Receptor 1	Protein Coding	41	GC19M054351	0.333128
SLC25A14	Solute Carrier Family 25 Member 14	Protein Coding	41	GC0XP130339	0.331925
UGT2B17	UDP Glucuronosyltransferase Family 2 Member B17	Protein Coding	41	GC04M068537	0.272315
HSD17B6	Hydroxysteroid 17-Beta Dehydrogenase 6	Protein Coding	41	GC12P056752	0.269146
UBA3	Ubiquitin Like Modifier Activating Enzyme 3	Protein Coding	41	GC03M069054	0.266303
GATA5	GATA Binding Protein 5	Protein Coding	40	GC20M062464	32.18092
TRMU	TRNA Mitochondrial 2-Thiouridylase	Protein Coding	40	GC22P046330	30.82014
NBAS	NBAS Subunit Of NRZ Tethering Complex	Protein Coding	40	GC02M014998	29.40333
CRELD1	Cysteine Rich With EGF Like Domains 1	Protein Coding	40	GC03P010065	23.4958
MKKS	MKKS Centrosomal Shuttling Protein	Protein Coding	40	GC20M010424	22.32983
SRP72	Signal Recognition Particle 72	Protein Coding	40	GC04P056466	20.67737
CCN2	Cellular Communication Network Factor 2	Protein Coding	40	GC06M131948	19.00111
CATSPER1	Cation Channel Sperm Associated 1	Protein Coding	40	GC11M069444	18.37687
NEXN	Nexilin F-Actin Binding Protein	Protein Coding	40	GC01P077898	18.23752
EVC2	EvC Ciliary Complex Subunit 2	Protein Coding	40	GC04M005534	17.70354
CHPT1	Choline Phosphotransferase 1	Protein Coding	40	GC12P101696	17.35031
MCM9	Minichromosome Maintenance 9 Homologous Recombination Repair Factor	Protein Coding	40	GC06M118813	17.13508
CTNNA3	Catenin Alpha 3	Protein Coding	40	GC10M065912	17.09427
MYSM1	Myb Like, SWIRM And MPN Domains 1	Protein Coding	40	GC01M058654	16.88097
MED13L	Mediator Complex Subunit 13L	Protein Coding	40	GC12M115953	16.45687
IFT172	Intraflagellar Transport 172	Protein Coding	40	GC02M027537	15.96155
DPY19L2	Dpy-19 Like 2	Protein Coding	40	GC12M063558	15.72756
IFT140	Intraflagellar Transport 140	Protein Coding	40	GC16M001707	15.54295
CTF1	Cardiotrophin 1	Protein Coding	40	GC16P032484	15.51992
FKTN	Fukutin	Protein Coding	40	GC09P105558	15.20448
JPH2	Junctophilin 2	Protein Coding	40	GC20M044111	14.84547

CSF3	Colony Stimulating Factor 3	Protein Coding	40	GC17P040015	14.37653
LTBP4	Latent Transforming Growth Factor Beta Binding Protein 4	Protein Coding	40	GC19P040592	14.26164
DACT1	Dishevelled Binding Antagonist Of Beta Catenin 1	Protein Coding	40	GC14P058633	13.91153
RAI1	Retinoic Acid Induced 1	Protein Coding	40	GC17P017682	13.74429
IFT122	Intraflagellar Transport 122	Protein Coding	40	GC03P129440	13.63216
DNAH5	Dynein Axonemal Heavy Chain 5	Protein Coding	40	GC05M013693	13.46636
HSPB7	Heat Shock Protein Family B (Small) Member 7	Protein Coding	40	GC01M016014	13.42205
NDUFB11	NADH:Ubiquinone Oxidoreductase Subunit B11	Protein Coding	40	GC0XM047142	12.81788
GDF9	Growth Differentiation Factor 9	Protein Coding	40	GC05M132861	12.80008
ATP8B1	ATPase Phospholipid Transporting 8B1	Protein Coding	40	GC18M057646	12.78873
AKAP10	A-Kinase Anchoring Protein 10	Protein Coding	40	GC17M019904	12.77065
FOXRED1	FAD Dependent Oxidoreductase Domain Containing 1	Protein Coding	40	GC11P126269	12.691
TBL2	Transducin Beta Like 2	Protein Coding	40	GC07M073568	12.3332
HFM1	Helicase For Meiosis 1	Protein Coding	40	GC01M091260	12.32979
SON	SON DNA And RNA Binding Protein	Protein Coding	40	GC21P033542	12.03739
FANCB	FA Complementation Group B	Protein Coding	40	GC0XM014690	11.74409
DYNC2LI1	Dynein Cytoplasmic 2 Light Intermediate Chain 1	Protein Coding	40	GC02P043779	11.74138
KIF7	Kinesin Family Member 7	Protein Coding	40	GC15M089608	11.16649
TDRD9	Tudor Domain Containing 9	Protein Coding	40	GC14P106050	11.06241
HEY2	Hes Related Family BHLH Transcription Factor With YRPW Motif 2	Protein Coding	40	GC06P125730	10.99795
FASTKD2	FAST Kinase Domains 2	Protein Coding	40	GC02P206766	10.99524
PPP1R13L	Protein Phosphatase 1 Regulatory Subunit 13 Like	Protein Coding	40	GC19M045379	10.9696
COX6A2	Cytochrome C Oxidase Subunit 6A2	Protein Coding	40	GC16M031439	10.94439
RAB3GAP2	RAB3 GTPase Activating Non-Catalytic Protein Subunit 2	Protein Coding	40	GC01M220149	10.8023
TEX14	Testis Expressed 14, Intercellular Bridge Forming Factor	Protein Coding	40	GC17M058556	10.709
NUBPL	Nucleotide Binding Protein Like	Protein Coding	40	GC14P031489	10.2241
PPP2R3C	Protein Phosphatase 2 Regulatory Subunit B''Gamma	Protein Coding	40	GC14M035085	10.22046
CEP120	Centrosomal Protein 120	Protein Coding	40	GC05M123344	10.21914
DOCK6	Dedicator Of Cytokinesis 6	Protein Coding	40	GC19M011199	10.11344
NDUFAF3	NADH:Ubiquinone Oxidoreductase Complex Assembly Factor 3	Protein Coding	40	GC03P049020	9.994247
LZTFL1	Leucine Zipper Transcription Factor Like 1	Protein Coding	40	GC03M045823	9.934031
CCL3	C-C Motif Chemokine Ligand 3	Protein Coding	40	GC17M036088	9.912916
TMCO1	Transmembrane And Coiled-Coil Domains 1	Protein Coding	40	GC01M165724	9.893276
PRDM6	PR/SET Domain 6	Protein Coding	40	GC05P123089	9.731916
AK7	Adenylate Kinase 7	Protein Coding	40	GC14P096392	9.532892
CLIC2	Chloride Intracellular Channel 2	Protein Coding	40	GC0XM155276	9.45773
TACO1	Translational Activator Of Cytochrome C Oxidase I	Protein Coding	40	GC17P063600	9.451832
TTC8	Tetratricopeptide Repeat Domain 8	Protein Coding	40	GC14P089293	9.442844
TCTN3	Tectonic Family Member 3	Protein Coding	40	GC10M095663	9.440065
PEX12	Peroxisomal Biogenesis Factor 12	Protein Coding	40	GC17M035574	9.379933
IFNA1	Interferon Alpha 1	Protein Coding	40	GC09P021522	9.277035
VPS13B	Vacuolar Protein Sorting 13 Homolog B	Protein Coding	40	GC08P099011	9.251198
ZFP57	ZFP57 Zinc Finger Protein	Protein Coding	40	GC06M029672	8.987421
CFHR5	Complement Factor H Related 5	Protein Coding	40	GC01P196977	8.842339
SYNE2	Spectrin Repeat Containing Nuclear Envelope Protein 2	Protein Coding	40	GC14P063761	8.818581
RFWD3	Ring Finger And WD Repeat Domain 3	Protein Coding	40	GC16M074621	8.784787
B9D2	B9 Domain Containing 2	Protein Coding	40	GC19M041354	8.719857
SHOX2	Short Stature Homeobox 2	Protein Coding	40	GC03M158095	8.593624
ATXN7	Ataxin 7	Protein Coding	40	GC03P063864	8.590686
FAT4	FAT Atypical Cadherin 4	Protein Coding	40	GC04P125315	8.49285
MMP21	Matrix Metallopeptidase 21	Protein Coding	40	GC10M125756	8.329476
PDLIM5	PDZ And LIM Domain 5	Protein Coding	40	GC04P094451	8.295322
NEBL	Nebulette	Protein Coding	40	GC10M020779	8.174639

FREM2	FRAS1 Related Extracellular Matrix 2	Protein Coding	40	GC13P038687	8.156386
HESX1	HESX Homeobox 1	Protein Coding	40	GC03M057207	8.15638
IFT81	Intraflagellar Transport 81	Protein Coding	40	GC12P110124	8.126101
DAZL	Deleted In Azoospermia Like	Protein Coding	40	GC03M016586	8.102688
ZNF462	Zinc Finger Protein 462	Protein Coding	40	GC09P106860	8.084605
RPGRIP1	RPGR Interacting Protein 1	Protein Coding	40	GC14P021673	8.030629
SH3PXD2B	SH3 And PX Domains 2B	Protein Coding	40	GC05M172325	7.885776
CCDC22	Coiled-Coil Domain Containing 22	Protein Coding	40	GC0XP049432	7.8854
B3GALT6	Beta-1,3-Galactosyltransferase 6	Protein Coding	40	GC01P001232	7.882796
IL18BP	Interleukin 18 Binding Protein	Protein Coding	40	GC11P071998	7.850857
AUTS2	Activator Of Transcription And Developmental Regulator AUTS2	Protein Coding	40	GC07P069598	7.713887
DNAI2	Dynein Axonemal Intermediate Chain 2	Protein Coding	40	GC17P074274	7.713556
RPS28	Ribosomal Protein S28	Protein Coding	40	GC19P008332	7.351394
RRAD	RRAD, Ras Related Glycolysis Inhibitor And Calcium Channel Regulator	Protein Coding	40	GC16M066925	7.337552
GLIS2	GLIS Family Zinc Finger 2	Protein Coding	40	GC16P005656	7.3048
SERPINA7	Serpin Family A Member 7	Protein Coding	40	GC0XM106032	7.296956
DGCR2	DiGeorge Syndrome Critical Region Gene 2	Protein Coding	40	GC22M019037	7.289345
OBSCN	Obscurin, Cytoskeletal Calmodulin And Titin-Interacting RhoGEF	Protein Coding	40	GC01P228208	7.281917
TTC12	Tetratricopeptide Repeat Domain 12	Protein Coding	40	GC11P113314	7.233743
SPAG1	Sperm Associated Antigen 1	Protein Coding	40	GC08P100157	7.170633
CLMP	CXADR Like Membrane Protein	Protein Coding	40	GC11M123069	7.1517
MRPL44	Mitochondrial Ribosomal Protein L44	Protein Coding	40	GC02P223957	7.142546
IMMT	Inner Membrane Mitochondrial Protein	Protein Coding	40	GC02M086144	7.116083
NME8	NME/NM23 Family Member 8	Protein Coding	40	GC07P037889	7.093103
PPCS	Phosphopantothenoylcysteine Synthetase	Protein Coding	40	GC01P042456	7.066525
C2CD3	C2 Domain Containing 3 Centriole Elongation Regulator	Protein Coding	40	GC11M074012	7.06471
PRG4	Proteoglycan 4	Protein Coding	40	GC01P186296	7.013953
SOX18	SRY-Box Transcription Factor 18	Protein Coding	40	GC20M064047	6.844166
SLPI	Secretory Leukocyte Peptidase Inhibitor	Protein Coding	40	GC20M045252	6.760644
RSPH1	Radial Spoke Head Component 1	Protein Coding	40	GC21M042472	6.754471
IL33	Interleukin 33	Protein Coding	40	GC09P006206	6.702066
SLC26A1	Solute Carrier Family 26 Member 1	Protein Coding	40	GC04M000979	6.689681
CFHR3	Complement Factor H Related 3	Protein Coding	40	GC01P196774	6.590509
CHCHD10	Coiled-Coil-Helix-Coiled-Coil-Helix Domain Containing 10	Protein Coding	40	GC22M023765	6.58726
DOK7	Docking Protein 7	Protein Coding	40	GC04P003465	6.576873
NALCN	Sodium Leak Channel, Non-Selective	Protein Coding	40	GC13M101053	6.557722
MYOM1	Myomesin 1	Protein Coding	40	GC18M003066	6.489672
MAP3K20	Mitogen-Activated Protein Kinase Kinase Kinase 20	Protein Coding	40	GC02P173076	6.464771
MYOG	Myogenin	Protein Coding	40	GC01M203083	6.461978
TKFC	Triokinase And FMN Cyclase	Protein Coding	40	GC11P061334	6.305336
PRKN	Parkin RBR E3 Ubiquitin Protein Ligase	Protein Coding	40	GC06M161348	6.295286
TTC19	Tetratricopeptide Repeat Domain 19	Protein Coding	40	GC17P015999	6.16797
PCDH7	Protocadherin 7	Protein Coding	40	GC04P030722	6.141407
FKBP14	FKBP Prolyl Isomerase 14	Protein Coding	40	GC07M030010	6.097335
GPBAR1	G Protein-Coupled Bile Acid Receptor 1	Protein Coding	40	GC02P218259	6.032326
SEMA3D	Semaphorin 3D	Protein Coding	40	GC07M084995	6.021958
ZNF423	Zinc Finger Protein 423	Protein Coding	40	GC16M049487	5.986543
IRX5	Iroquois Homeobox 5	Protein Coding	40	GC16P054930	5.976391
SRCAP	Snf2 Related CREBBP Activator Protein	Protein Coding	40	GC16P032468	5.936203
CD68	CD68 Molecule	Protein Coding	40	GC17P007579	5.924465
AMN	Amnion Associated Transmembrane Protein	Protein Coding	40	GC14P102922	5.89209
S100A12	S100 Calcium Binding Protein A12	Protein Coding	40	GC01M153373	5.797328
PLCH2	Phospholipase C Eta 2	Protein Coding	40	GC01P002413	5.767242

CHGB	Chromogranin B	Protein Coding	40	GC20P005911	5.756404
SLC24A3	Solute Carrier Family 24 Member 3	Protein Coding	40	GC20P019212	5.730378
GNPTG	N-Acetylglucosamine-1-Phosphate Transferase Subunit Gamma	Protein Coding	40	GC16P001351	5.688295
DEAF1	DEAF1 Transcription Factor	Protein Coding	40	GC11M000644	5.681628
RBFOX2	RNA Binding Fox-1 Homolog 2	Protein Coding	40	GC22M035738	5.650754
SIX5	SIX Homeobox 5	Protein Coding	40	GC19M045764	5.595821
COG8	Component Of Oligomeric Golgi Complex 8	Protein Coding	40	GC16M069320	5.558831
OTUD6B	OTU Deubiquitinase 6B	Protein Coding	40	GC08P091070	5.529587
AP4M1	Adaptor Related Protein Complex 4 Subunit Mu 1	Protein Coding	40	GC07P100101	5.524831
OXA1L	OXA1L Mitochondrial Inner Membrane Protein	Protein Coding	40	GC14P022766	5.512979
NLGN4X	Neuroligin 4 X-Linked	Protein Coding	40	GC0XM005840	5.497977
NDRG4	NDRG Family Member 4	Protein Coding	40	GC16P058462	5.437119
THSD1	Thrombospondin Type 1 Domain Containing 1	Protein Coding	40	GC13M052377	5.382263
TELO2	Telomere Maintenance 2	Protein Coding	40	GC16P001493	5.374537
USP53	Ubiquitin Specific Peptidase 53	Protein Coding	40	GC04P119212	5.357171
SRP19	Signal Recognition Particle 19	Protein Coding	40	GC05P112862	5.349946
SC5D	Sterol-C5-Desaturase	Protein Coding	40	GC11P121292	5.309381
FREM1	FRAS1 Related Extracellular Matrix 1	Protein Coding	40	GC09M014734	5.299195
SHANK3	SH3 And Multiple Ankyrin Repeat Domains 3	Protein Coding	40	GC22P050674	5.298204
PAEP	Progestagen Associated Endometrial Protein	Protein Coding	40	GC09P135561	5.286452
ALX4	ALX Homeobox 4	Protein Coding	40	GC11M044238	5.281953
ELP4	Elongator Acetyltransferase Complex Subunit 4	Protein Coding	40	GC11P031509	5.256379
QRSL1	Glutaminyl-TRNA Amidotransferase Subunit QRSL1	Protein Coding	40	GC06P106629	5.253412
EDARADD	EDAR Associated Death Domain	Protein Coding	40	GC01P236348	5.201951
ALG11	ALG11 Alpha-1,2-Mannosyltransferase	Protein Coding	40	GC13P052012	5.121402
MAPKBP1	Mitogen-Activated Protein Kinase Binding Protein 1	Protein Coding	40	GC15P041775	5.118558
PIGT	Phosphatidylinositol Glycan Anchor Biosynthesis Class T	Protein Coding	40	GC20P045416	5.000442
PLEK	Pleckstrin	Protein Coding	40	GC02P068365	4.981941
APC2	APC Regulator Of WNT Signaling Pathway 2	Protein Coding	40	GC19P001638	4.969872
FEZF1	FEZ Family Zinc Finger 1	Protein Coding	40	GC07M122301	4.949007
SSBP1	Single Stranded DNA Binding Protein 1	Protein Coding	40	GC07P146002	4.947075
HSPA1B	Heat Shock Protein Family A (Hsp70) Member 1B	Protein Coding	40	GC06P055218	4.88595
UTRN	Utrophin	Protein Coding	40	GC06P144285	4.850117
COLQ	Collagen Like Tail Subunit Of Asymmetric Acetylcholinesterase	Protein Coding	40	GC03M016945	4.772665
BMPER	BMP Binding Endothelial Regulator	Protein Coding	40	GC07P033944	4.738254
FAM20A	FAM20A Golgi Associated Secretory Pathway Pseudokinase	Protein Coding	40	GC17M068535	4.734654
GHRH	Growth Hormone Releasing Hormone	Protein Coding	40	GC20M037251	4.733803
LMBR1	Limb Development Membrane Protein 1	Protein Coding	40	GC07M156668	4.730341
CWC27	CWC27 Spliceosome Associated Cyclophilin	Protein Coding	40	GC05P064768	4.721352
TBX6	T-Box Transcription Factor 6	Protein Coding	40	GC16M030085	4.702325
ARHGAP24	Rho GTPase Activating Protein 24	Protein Coding	40	GC04P085475	4.692191
FGF21	Fibroblast Growth Factor 21	Protein Coding	40	GC19P048781	4.68608
MRPL12	Mitochondrial Ribosomal Protein L12	Protein Coding	40	GC17P081704	4.653795
MKRN3	Makorin Ring Finger Protein 3	Protein Coding	40	GC15P027412	4.651358
IFT57	Intraflagellar Transport 57	Protein Coding	40	GC03M108160	4.647474
MEST	Mesoderm Specific Transcript	Protein Coding	40	GC07P130486	4.638647
NES	Nestin	Protein Coding	40	GC01M156668	4.628138
TNS1	Tensin 1	Protein Coding	40	GC02M217799	4.622834
RGL1	Ral Guanine Nucleotide Dissociation Stimulator Like 1	Protein Coding	40	GC01P183636	4.621198
ATAD3A	ATPase Family AAA Domain Containing 3A	Protein Coding	40	GC01P002047	4.608159
BPTF	Bromodomain PHD Finger Transcription Factor	Protein Coding	40	GC17P067825	4.548221
SORBS2	Sorbin And SH3 Domain Containing 2	Protein Coding	40	GC04M185585	4.541033
CXCL16	C-X-C Motif Chemokine Ligand 16	Protein Coding	40	GC17M004733	4.528139

RHOD	Ras Homolog Family Member D	Protein Coding	40	GC11P067057	4.51378
EXOSC9	Exosome Component 9	Protein Coding	40	GC04P121801	4.510591
PRDM5	PR/SET Domain 5	Protein Coding	40	GC04M120686	4.500328
CDH7	Cadherin 7	Protein Coding	40	GC18P065750	4.465474
CRIPT	CXXC Repeat Containing Interactor Of PDZ3 Domain	Protein Coding	40	GC02P046581	4.459215
CD83	CD83 Molecule	Protein Coding	40	GC06P014117	4.457798
NUS1	NUS1 Dehydrodolichyl Diphosphate Synthase Subunit	Protein Coding	40	GC06P117675	4.454024
AOC1	Amine Oxidase Copper Containing 1	Protein Coding	40	GC07P150824	4.427603
LDB1	LIM Domain Binding 1	Protein Coding	40	GC10M102106	4.366721
GAST	Gastrin	Protein Coding	40	GC17P041712	4.357377
RNASEH2C	Ribonuclease H2 Subunit C	Protein Coding	40	GC11M065714	4.353891
TMSB4X	Thymosin Beta 4 X-Linked	Protein Coding	40	GC0XP012975	4.352621
PCDH15	Protocadherin Related 15	Protein Coding	40	GC10M053802	4.350641
ACTN3	Actinin Alpha 3	Protein Coding	40	GC11P066546	4.3275
FAT1	FAT Atypical Cadherin 1	Protein Coding	40	GC04M186587	4.316947
ADAMTS12	ADAM Metallopeptidase With Thrombospondin Type 1 Motif 12	Protein Coding	40	GC05M033524	4.307258
SNRNP70	Small Nuclear Ribonucleoprotein U1 Subunit 70	Protein Coding	40	GC19P049085	4.298823
TNPO3	Transportin 3	Protein Coding	40	GC07M128954	4.286664
MYH4	Myosin Heavy Chain 4	Protein Coding	40	GC17M010443	4.273663
HBS1L	HBS1 Like Translational GTPase	Protein Coding	40	GC06M134960	4.197225
ERGIC1	Endoplasmic Reticulum-Golgi Intermediate Compartment 1	Protein Coding	40	GC05P172834	4.114714
CD93	CD93 Molecule	Protein Coding	40	GC20M023079	4.105604
AIF1	Allograft Inflammatory Factor 1	Protein Coding	40	GC06P055201	4.097908
PLAG1	PLAG1 Zinc Finger	Protein Coding	40	GC08M056161	4.090955
CRLS1	Cardiolipin Synthase 1	Protein Coding	40	GC20P006005	4.030003
HOOK1	Hook Microtubule Tethering Protein 1	Protein Coding	40	GC01P059814	4.003281
TOX	Thymocyte Selection Associated High Mobility Group Box	Protein Coding	40	GC08M058791	3.983482
AKAP1	A-Kinase Anchoring Protein 1	Protein Coding	40	GC17P057085	3.977193
FCGRT	Fc Fragment Of IgG Receptor And Transporter	Protein Coding	40	GC19P049506	3.975687
NAIP	NLR Family Apoptosis Inhibitory Protein	Protein Coding	40	GC05M070968	3.956545
GLRX5	Glutaredoxin 5	Protein Coding	40	GC14P095533	3.933271
ACP6	Acid Phosphatase 6, Lysophosphatidic	Protein Coding	40	GC01M147630	3.875245
RLIM	Ring Finger Protein, LIM Domain Interacting	Protein Coding	40	GC0XM074612	3.861822
SLC35C1	Solute Carrier Family 35 Member C1	Protein Coding	40	GC11P046086	3.858924
DHX36	DEAH-Box Helicase 36	Protein Coding	40	GC03M154272	3.852408
KCTD1	Potassium Channel Tetramerization Domain Containing 1	Protein Coding	40	GC18M026454	3.844034
USP3	Ubiquitin Specific Peptidase 3	Protein Coding	40	GC15P063504	3.83998
PVALB	Parvalbumin	Protein Coding	40	GC22M036800	3.83375
KCND1	Potassium Voltage-Gated Channel Subfamily D Member 1	Protein Coding	40	GC0XM048968	3.783268
CENPA	Centromere Protein A	Protein Coding	40	GC02P026765	3.747738
WAC	WW Domain Containing Adaptor With Coiled-Coil	Protein Coding	40	GC10P028557	3.737425
MAGT1	Magnesium Transporter 1	Protein Coding	40	GC0XM077827	3.730609
HEYL	Hes Related Family BHLH Transcription Factor With YRPW Motif Like	Protein Coding	40	GC01M039623	3.730052
KCNJ16	Potassium Inwardly Rectifying Channel Subfamily J Member 16	Protein Coding	40	GC17P070053	3.720028
KCNK13	Potassium Two Pore Domain Channel Subfamily K Member 13	Protein Coding	40	GC14P090061	3.716482
MED25	Mediator Complex Subunit 25	Protein Coding	40	GC19P049819	3.713994
SRSF3	Serine And Arginine Rich Splicing Factor 3	Protein Coding	40	GC06P055345	3.710813
ESM1	Endothelial Cell Specific Molecule 1	Protein Coding	40	GC05M054977	3.701277
SPG11	SPG11 Vesicle Trafficking Associated, Spatacsin	Protein Coding	40	GC15M044562	3.700195
RBP1	Retinol Binding Protein 1	Protein Coding	40	GC03M139517	3.697707
CRIM1	Cysteine Rich Transmembrane BMP Regulator 1	Protein Coding	40	GC02P036355	3.693492
FKBP6	FKBP Prolyl Isomerase Family Member 6 (Inactive)	Protein Coding	40	GC07P073328	3.669247
ATE1	Arginyltransferase 1	Protein Coding	40	GC10M121740	3.665668

UNC45B	Unc-45 Myosin Chaperone B	Protein Coding	40	GC17P035148	3.660395
MRC1	Mannose Receptor C-Type 1	Protein Coding	40	GC10P017809	3.652276
AMMECR1	AMMECR Nuclear Protein 1	Protein Coding	40	GC0XM110194	3.651332
PLXNC1	Plexin C1	Protein Coding	40	GC12P094150	3.649723
RALGAPA1	Ral GTPase Activating Protein Catalytic Subunit Alpha 1	Protein Coding	40	GC14M035538	3.644564
UNC93B1	Unc-93 Homolog B1, TLR Signaling Regulator	Protein Coding	40	GC11M067991	3.639209
CD58	CD58 Molecule	Protein Coding	40	GC01M116514	3.636823
GLIS3	GLIS Family Zinc Finger 3	Protein Coding	40	GC09M003816	3.622093
FGF20	Fibroblast Growth Factor 20	Protein Coding	40	GC08M016992	3.608797
UBQLN4	Ubiquilin 4	Protein Coding	40	GC01M156033	3.596591
CERS3	Ceramide Synthase 3	Protein Coding	40	GC15M107485	3.572669
RHOBTB2	Rho Related BTB Domain Containing 2	Protein Coding	40	GC08P022987	3.569641
GFI1B	Growth Factor Independent 1B Transcriptional Repressor	Protein Coding	40	GC09P132945	3.566253
APCDD1	APC Down-Regulated 1	Protein Coding	40	GC18P010454	3.555887
MAPRE3	Microtubule Associated Protein RP/EB Family Member 3	Protein Coding	40	GC02P026935	3.553964
MAML1	Mastermind Like Transcriptional Coactivator 1	Protein Coding	40	GC05P179732	3.549544
KIF20A	Kinesin Family Member 20A	Protein Coding	40	GC05P138189	3.536165
DAB2IP	DAB2 Interacting Protein	Protein Coding	40	GC09P121566	3.53177
RNF213	Ring Finger Protein 213	Protein Coding	40	GC17P080260	3.528949
SEMA6D	Semaphorin 6D	Protein Coding	40	GC15P047184	3.518398
AGFG1	ArfGAP With FG Repeats 1	Protein Coding	40	GC02P227473	3.514072
CYBRD1	Cytochrome B Reductase 1	Protein Coding	40	GC02P171522	3.511624
RASD1	Ras Related Dexamethasone Induced 1	Protein Coding	40	GC17M017494	3.480628
APOM	Apolipoprotein M	Protein Coding	40	GC06P055209	3.470902
NPAS3	Neuronal PAS Domain Protein 3	Protein Coding	40	GC14P032934	3.467878
KIF3B	Kinesin Family Member 3B	Protein Coding	40	GC20P032277	3.459801
MYO1A	Myosin IA	Protein Coding	40	GC12M057028	3.431561
FMN2	Formin 2	Protein Coding	40	GC01P240014	3.39554
RARRES2	Retinoic Acid Receptor Responder 2	Protein Coding	40	GC07M150333	3.38149
ANKS1B	Ankyrin Repeat And Sterile Alpha Motif Domain Containing 1B	Protein Coding	40	GC12M098726	3.371722
STARD13	StAR Related Lipid Transfer Domain Containing 13	Protein Coding	40	GC13M033103	3.359452
MBNL1	Muscleblind Like Splicing Regulator 1	Protein Coding	40	GC03P152243	3.340781
MED15	Mediator Complex Subunit 15	Protein Coding	40	GC22P020506	3.336226
NUP160	Nucleoporin 160	Protein Coding	40	GC11M069013	3.314512
ID4	Inhibitor Of DNA Binding 4, HLH Protein	Protein Coding	40	GC06P019837	3.313754
ZBTB18	Zinc Finger And BTB Domain Containing 18	Protein Coding	40	GC01P244048	3.299903
SLC45A2	Solute Carrier Family 45 Member 2	Protein Coding	40	GC05M033944	3.297187
KMT2E	Lysine Methyltransferase 2E (Inactive)	Protein Coding	40	GC07P104950	3.279382
COLEC10	Collectin Subfamily Member 10	Protein Coding	40	GC08P118952	3.278226
NANOG	Nanog Homeobox	Protein Coding	40	GC12P007787	3.268343
SLC9A3R2	SLC9A3 Regulator 2	Protein Coding	40	GC16P005577	3.263875
NEGR1	Neuronal Growth Regulator 1	Protein Coding	40	GC01M071395	3.251416
PGRMC2	Progesterone Receptor Membrane Component 2	Protein Coding	40	GC04M128269	3.245505
ALX1	ALX Homeobox 1	Protein Coding	40	GC12P085279	3.238195
VPS45	Vacuolar Protein Sorting 45 Homolog	Protein Coding	40	GC01P150068	3.228326
IL32	Interleukin 32	Protein Coding	40	GC16P005617	3.218756
TSHZ1	Teashirt Zinc Finger Homeobox 1	Protein Coding	40	GC18P075210	3.215989
ACR	Acrosin	Protein Coding	40	GC22P050738	3.213033
MYLIP	Myosin Regulatory Light Chain Interacting Protein	Protein Coding	40	GC06P016129	3.205622
SMARCA1	SWI/SNF Related, Matrix Associated, Actin Dependent Regulator Of Chromatin, Subfamily A, Member 1	Protein Coding	40	GC0XM129447	3.16734
SEC23IP	SEC23 Interacting Protein	Protein Coding	40	GC10P119892	3.142981
CNKSR2	Connector Enhancer Of Kinase Suppressor Of Ras 2	Protein Coding	40	GC0XP021392	3.13263
SRPX2	Sushi Repeat Containing Protein X-Linked 2	Protein Coding	40	GC0XP100648	3.131557

NDST2	N-Deacetylase And N-Sulfotransferase 2	Protein Coding	40	GC10M073801	3.12123
FGL2	Fibrinogen Like 2	Protein Coding	40	GC07M077193	3.111036
FLG	Filaggrin	Protein Coding	40	GC01M152274	3.102594
FBXL3	F-Box And Leucine Rich Repeat Protein 3	Protein Coding	40	GC13M076992	3.086304
ZNF202	Zinc Finger Protein 202	Protein Coding	40	GC11M123724	3.077536
GBX2	Gastrulation Brain Homeobox 2	Protein Coding	40	GC02M236165	3.076787
HSPB6	Heat Shock Protein Family B (Small) Member 6	Protein Coding	40	GC19M046669	3.073607
HPX	Hemopexin	Protein Coding	40	GC11M006435	3.063629
OPA3	Outer Mitochondrial Membrane Lipid Metabolism Regulator OPA3	Protein Coding	40	GC19M045527	3.054214
RPS8	Ribosomal Protein S8	Protein Coding	40	GC01P044775	3.052428
HNRNPH1	Heterogeneous Nuclear Ribonucleoprotein H1	Protein Coding	40	GC05M179614	3.04907
PIGG	Phosphatidylinositol Glycan Anchor Biosynthesis Class G	Protein Coding	40	GC04P000505	3.040463
PITRM1	Pitrilysin Metallopeptidase 1	Protein Coding	40	GC10M003138	3.033552
ADAMTS7	ADAM Metallopeptidase With Thrombospondin Type 1 Motif 7	Protein Coding	40	GC15M078759	3.030333
YBX1	Y-Box Binding Protein 1	Protein Coding	40	GC01P042682	3.024442
HBZ	Hemoglobin Subunit Zeta	Protein Coding	40	GC16P000142	3.022205
TTF2	Transcription Termination Factor 2	Protein Coding	40	GC01P117060	3.02165
GPR4	G Protein-Coupled Receptor 4	Protein Coding	40	GC19M045589	3.017198
RBMS1	RNA Binding Motif Single Stranded Interacting Protein 1	Protein Coding	40	GC02M160272	3.006983
RPS4X	Ribosomal Protein S4 X-Linked	Protein Coding	40	GC0XM072255	3.004582
SNIP1	Smad Nuclear Interacting Protein 1	Protein Coding	40	GC01M037534	3.00133
DBN1	Drebrin 1	Protein Coding	40	GC05M177456	2.991274
HSPB3	Heat Shock Protein Family B (Small) Member 3	Protein Coding	40	GC05P054456	2.984316
ASPN	Asporin	Protein Coding	40	GC09M092458	2.97133
SUPT5H	SPT5 Homolog, DSIF Elongation Factor Subunit	Protein Coding	40	GC19P039436	2.969807
BAZ1A	Bromodomain Adjacent To Zinc Finger Domain 1A	Protein Coding	40	GC14M034752	2.948653
CAP2	Cyclase Associated Actin Cytoskeleton Regulatory Protein 2	Protein Coding	40	GC06P017393	2.94617
KLF11	Kruppel Like Factor 11	Protein Coding	40	GC02P010044	2.942815
ERAL1	Era Like 12S Mitochondrial RRNA Chaperone 1	Protein Coding	40	GC17P029368	2.939248
SCARA5	Scavenger Receptor Class A Member 5	Protein Coding	40	GC08M027869	2.922576
HNRNPM	Heterogeneous Nuclear Ribonucleoprotein M	Protein Coding	40	GC19P008444	2.917613
PACS2	Phosphofurin Acidic Cluster Sorting Protein 2	Protein Coding	40	GC14P105300	2.909792
HPR	Haptoglobin-Related Protein	Protein Coding	40	GC16P072097	2.897083
FCN1	Ficolin 1	Protein Coding	40	GC09M135029	2.886984
RIMS1	Regulating Synaptic Membrane Exocytosis 1	Protein Coding	40	GC06P071886	2.880385
DDHD2	DDHD Domain Containing 2	Protein Coding	40	GC08P038208	2.864708
GUCA2B	Guanylate Cyclase Activator 2B	Protein Coding	40	GC01P042153	2.862292
PKP4	Plakophilin 4	Protein Coding	40	GC02P158456	2.857847
CABLES1	Cdk5 And Abl Enzyme Substrate 1	Protein Coding	40	GC18P023134	2.844138
HBE1	Hemoglobin Subunit Epsilon 1	Protein Coding	40	GC11M005268	2.836123
TNFRSF19	TNF Receptor Superfamily Member 19	Protein Coding	40	GC13P023570	2.828091
ARHGAP32	Rho GTPase Activating Protein 32	Protein Coding	40	GC11M128965	2.810831
IVNS1ABP	Influenza Virus NS1A Binding Protein	Protein Coding	40	GC01M185299	2.809672
PPP1R8	Protein Phosphatase 1 Regulatory Subunit 8	Protein Coding	40	GC01P027830	2.800304
SMG6	SMG6 Nonsense Mediated MRNA Decay Factor	Protein Coding	40	GC17M002059	2.793717
CDKAL1	CDK5 Regulatory Subunit Associated Protein 1 Like 1	Protein Coding	40	GC06P020534	2.792655
DCDC2	Doublecortin Domain Containing 2	Protein Coding	40	GC06M024171	2.787659
LRRC8A	Leucine Rich Repeat Containing 8 VRAC Subunit A	Protein Coding	40	GC09P128882	2.77161
PLXNA4	Plexin A4	Protein Coding	40	GC07M132123	2.765011
SAA4	Serum Amyloid A4, Constitutive	Protein Coding	40	GC11M018234	2.745231
BLZF1	Basic Leucine Zipper Nuclear Factor 1	Protein Coding	40	GC01P169367	2.735761
YBX3	Y-Box Binding Protein 3	Protein Coding	40	GC12M015696	2.735539
SCG2	Secretogranin II	Protein Coding	40	GC02M223596	2.734765

CNTN4	Contactin 4	Protein Coding	40	GC03P002117	2.734502
PLA2G4F	Phospholipase A2 Group IVF	Protein Coding	40	GC15M042139	2.731238
MYL12A	Myosin Light Chain 12A	Protein Coding	40	GC18P003238	2.730922
MYH13	Myosin Heavy Chain 13	Protein Coding	40	GC17M010300	2.729034
DACH1	Dachshund Family Transcription Factor 1	Protein Coding	40	GC13M071437	2.72658
ECEL1	Endothelin Converting Enzyme Like 1	Protein Coding	40	GC02M232479	2.720162
MGAT5	Alpha-1,6-Mannosylglycoprotein 6-Beta-N-Acetylglucosaminyltransferase	Protein Coding	40	GC02P134119	2.719628
P4HA3	Prolyl 4-Hydroxylase Subunit Alpha 3	Protein Coding	40	GC11M074235	2.716354
NBEA	Neurobeachin	Protein Coding	40	GC13P034942	2.710291
SACM1L	SAC1 Like Phosphatidylinositide Phosphatase	Protein Coding	40	GC03P045692	2.706651
MAN1A1	Mannosidase Alpha Class 1A Member 1	Protein Coding	40	GC06M119269	2.697021
MGRN1	Mahogunin Ring Finger 1	Protein Coding	40	GC16P005659	2.681331
LGI4	Leucine Rich Repeat LGI Family Member 4	Protein Coding	40	GC19M035124	2.674884
RPS11	Ribosomal Protein S11	Protein Coding	40	GC19P049496	2.667793
CCDC88C	Coiled-Coil Domain Containing 88C	Protein Coding	40	GC14M091271	2.663629
SERPINB1	Serpin Family B Member 1	Protein Coding	40	GC06M002833	2.652207
SPAST	Spastin	Protein Coding	40	GC02P032063	2.637121
B3GNT2	UDP-GlcNAc:BetaGal Beta-1,3-N-Acetylglucosaminyltransferase 2	Protein Coding	40	GC02P062196	2.62887
SBF2	SET Binding Factor 2	Protein Coding	40	GC11M009789	2.622749
RHOT2	Ras Homolog Family Member T2	Protein Coding	40	GC16P005512	2.618015
SPAM1	Sperm Adhesion Molecule 1	Protein Coding	40	GC07P123925	2.616661
CER1	Cerberus 1, DAN Family BMP Antagonist	Protein Coding	40	GC09M014710	2.602767
SEC24B	SEC24 Homolog B, COPII Coat Complex Component	Protein Coding	40	GC04P109433	2.598259
RDH10	Retinol Dehydrogenase 10	Protein Coding	40	GC08P073294	2.589592
TLN2	Talin 2	Protein Coding	40	GC15P062390	2.582545
RNF19A	Ring Finger Protein 19A, RBR E3 Ubiquitin Protein Ligase	Protein Coding	40	GC08M100257	2.559106
DAP	Death Associated Protein	Protein Coding	40	GC05M010679	2.554108
SENP3	SUMO Specific Peptidase 3	Protein Coding	40	GC17P009106	2.553167
WNK3	WNK Lysine Deficient Protein Kinase 3	Protein Coding	40	GC0XM054194	2.550484
KCNK2	Potassium Two Pore Domain Channel Subfamily K Member 2	Protein Coding	40	GC01P215005	2.533213
RIF1	Replication Timing Regulatory Factor 1	Protein Coding	40	GC02P151409	2.530923
ITGAE	Integrin Subunit Alpha E	Protein Coding	40	GC17M003722	2.523983
FAR2	Fatty Acyl-CoA Reductase 2	Protein Coding	40	GC12P029145	2.520049
GTF2H5	General Transcription Factor IIH Subunit 5	Protein Coding	40	GC06P158168	2.519384
RBBP6	RB Binding Protein 6, Ubiquitin Ligase	Protein Coding	40	GC16P024537	2.519065
GZMM	Granzyme M	Protein Coding	40	GC19P000544	2.515328
NRBP1	Nuclear Receptor Binding Protein 1	Protein Coding	40	GC02P027427	2.514231
TSEN15	TRNA Splicing Endonuclease Subunit 15	Protein Coding	40	GC01P184020	2.494024
LRRC7	Leucine Rich Repeat Containing 7	Protein Coding	40	GC01P069567	2.493566
ATP10A	ATPase Phospholipid Transporting 10A (Putative)	Protein Coding	40	GC15M025666	2.493468
PROZ	Protein Z, Vitamin K Dependent Plasma Glycoprotein	Protein Coding	40	GC13P113158	2.493336
UFSP2	UFM1 Specific Peptidase 2	Protein Coding	40	GC04M185399	2.491195
UQCR10	Ubiquinol-Cytochrome C Reductase, Complex III Subunit X	Protein Coding	40	GC22P029767	2.487626
RPL6	Ribosomal Protein L6	Protein Coding	40	GC12M112320	2.479955
HOXB5	Homeobox B5	Protein Coding	40	GC17M048591	2.465912
ELP3	Elongator Acetyltransferase Complex Subunit 3	Protein Coding	40	GC08P028089	2.46453
LRP1B	LDL Receptor Related Protein 1B	Protein Coding	40	GC02M140231	2.449593
HACL1	2-Hydroxyacyl-CoA Lyase 1	Protein Coding	40	GC03M016952	2.445762
TRDMT1	TRNA Aspartic Acid Methyltransferase 1	Protein Coding	40	GC10M017138	2.443298
PDPN	Podoplanin	Protein Coding	40	GC01P013583	2.443066
TNRC6A	Trinucleotide Repeat Containing Adaptor 6A	Protein Coding	40	GC16P024611	2.433573
MAGED2	MAGE Family Member D2	Protein Coding	40	GC0XP054807	2.427769
DHRS9	Dehydrogenase/Reductase 9	Protein Coding	40	GC02P169064	2.424751

NAT10	N-Acetyltransferase 10	Protein Coding	40	GC11P034105	2.422262
IP6K3	Inositol Hexakisphosphate Kinase 3	Protein Coding	40	GC06M033721	2.4181
MYL6B	Myosin Light Chain 6B	Protein Coding	40	GC12P056388	2.414246
HOXA3	Homeobox A3	Protein Coding	40	GC07M027107	2.41201
EXTL1	Exostosin Like Glycosyltransferase 1	Protein Coding	40	GC01P026032	2.409898
IGSF3	Immunoglobulin Superfamily Member 3	Protein Coding	40	GC01M116574	2.409462
PPP1R2	Protein Phosphatase 1 Regulatory Inhibitor Subunit 2	Protein Coding	40	GC03M195515	2.40921
CD200	CD200 Molecule	Protein Coding	40	GC03P112332	2.402837
PLRG1	Pleiotropic Regulator 1	Protein Coding	40	GC04M154534	2.402381
DOCK4	Dedicator Of Cytokinesis 4	Protein Coding	40	GC07M111726	2.401219
TSC22D3	TSC22 Domain Family Member 3	Protein Coding	40	GC0XM107713	2.393044
RCC2	Regulator Of Chromosome Condensation 2	Protein Coding	40	GC01M017406	2.386645
GAPVD1	GTPase Activating Protein And VPS9 Domains 1	Protein Coding	40	GC09P125261	2.386133
SLC35D1	Solute Carrier Family 35 Member D1	Protein Coding	40	GC01M066999	2.385174
KLF3	Kruppel Like Factor 3	Protein Coding	40	GC04P038668	2.375412
PREB	Prolactin Regulatory Element Binding	Protein Coding	40	GC02M027130	2.374976
NPTXR	Neuronal Pentraxin Receptor	Protein Coding	40	GC22M038818	2.367862
IL23A	Interleukin 23 Subunit Alpha	Protein Coding	40	GC12P056440	2.363673
PARG	Poly(ADP-Ribose) Glycohydrolase	Protein Coding	40	GC10M049818	2.363168
PSMD6	Proteasome 26S Subunit, Non-ATPase 6	Protein Coding	40	GC03M063973	2.360255
COL16A1	Collagen Type XVI Alpha 1 Chain	Protein Coding	40	GC01M031653	2.352489
CHL1	Cell Adhesion Molecule L1 Like	Protein Coding	40	GC03P000213	2.345257
TOMM20	Translocase Of Outer Mitochondrial Membrane 20	Protein Coding	40	GC01M235109	2.343366
NPLOC4	NPL4 Homolog, Ubiquitin Recognition Factor	Protein Coding	40	GC17M081556	2.340612
BPHL	Biphenyl Hydrolase Like	Protein Coding	40	GC06P003118	2.337816
RSU1	Ras Suppressor Protein 1	Protein Coding	40	GC10M016672	2.329188
VPS53	VPS53 Subunit Of GARP Complex	Protein Coding	40	GC17M000508	2.32144
DDX39B	DExD-Box Helicase 39B	Protein Coding	40	GC06M031530	2.317437
SMC2	Structural Maintenance Of Chromosomes 2	Protein Coding	40	GC09P104094	2.312956
BICD1	BICD Cargo Adaptor 1	Protein Coding	40	GC12P032107	2.310973
MFAP4	Microfibril Associated Protein 4	Protein Coding	40	GC17M019383	2.306705
ACOT11	Acyl-CoA Thioesterase 11	Protein Coding	40	GC01P054542	2.302419
ATP8B2	ATPase Phospholipid Transporting 8B2	Protein Coding	40	GC01P154325	2.30179
KTN1	Kinectin 1	Protein Coding	40	GC14P055559	2.298223
AP1S1	Adaptor Related Protein Complex 1 Subunit Sigma 1	Protein Coding	40	GC07P101154	2.295135
SLC26A9	Solute Carrier Family 26 Member 9	Protein Coding	40	GC01M205883	2.294967
CD109	CD109 Molecule	Protein Coding	40	GC06P073695	2.294305
ZDHHC8	Zinc Finger DHHC-Type Palmitoyltransferase 8	Protein Coding	40	GC22P020129	2.288875
RCN1	Reticulocalbin 1	Protein Coding	40	GC11P032090	2.2869
CCRL2	C-C Motif Chemokine Receptor Like 2	Protein Coding	40	GC03P046407	2.283638
OGN	Osteoglycin	Protein Coding	40	GC09M092383	2.274332
ELAVL4	ELAV Like RNA Binding Protein 4	Protein Coding	40	GC01P050025	2.274042
CTCFL	CCCTC-Binding Factor Like	Protein Coding	40	GC20M057495	2.271553
DEPTOR	DEP Domain Containing MTOR Interacting Protein	Protein Coding	40	GC08P119873	2.265261
ARIH1	Ariadne RBR E3 Ubiquitin Protein Ligase 1	Protein Coding	40	GC15P072474	2.265097
DPP10	Dipeptidyl Peptidase Like 10	Protein Coding	40	GC02P114442	2.262646
SAFB	Scaffold Attachment Factor B	Protein Coding	40	GC19P005623	2.262265
MYLPF	Myosin Light Chain, Phosphorylatable, Fast Skeletal Muscle	Protein Coding	40	GC16P030370	2.259363
HLTF	Helicase Like Transcription Factor	Protein Coding	40	GC03M149030	2.255846
GNPDA2	Glucosamine-6-Phosphate Deaminase 2	Protein Coding	40	GC04M044684	2.255096
DYNC1I2	Dynein Cytoplasmic 1 Intermediate Chain 2	Protein Coding	40	GC02P171687	2.250609
CDC42BPA	CDC42 Binding Protein Kinase Alpha	Protein Coding	40	GC01M226989	2.246504
TEAD2	TEA Domain Transcription Factor 2	Protein Coding	40	GC19M049341	2.245926

EPB41L2	Erythrocyte Membrane Protein Band 4.1 Like 2	Protein Coding	40	GC06M130820	2.24076
HNRNPL	Heterogeneous Nuclear Ribonucleoprotein L	Protein Coding	40	GC19M038836	2.238846
REG3A	Regenerating Family Member 3 Alpha	Protein Coding	40	GC02M079157	2.238078
AKT1S1	AKT1 Substrate 1	Protein Coding	40	GC19M049869	2.225423
ATP8B3	ATPase Phospholipid Transporting 8B3	Protein Coding	40	GC19M002182	2.220314
SLC9A7	Solute Carrier Family 9 Member A7	Protein Coding	40	GC0XM046599	2.220025
DCBLD2	Discoidin, CUB And LCCL Domain Containing 2	Protein Coding	40	GC03M098795	2.206206
CD320	CD320 Molecule	Protein Coding	40	GC19M008302	2.20453
SNRPD1	Small Nuclear Ribonucleoprotein D1 Polypeptide	Protein Coding	40	GC18P021612	2.203442
CTRL	Chymotrypsin Like	Protein Coding	40	GC16M067927	2.201943
FLT3LG	Fms Related Receptor Tyrosine Kinase 3 Ligand	Protein Coding	40	GC19P049475	2.19737
TRA2B	Transformer 2 Beta Homolog	Protein Coding	40	GC03M185914	2.196738
PI3	Peptidase Inhibitor 3	Protein Coding	40	GC20P045174	2.192729
ASIC2	Acid Sensing Ion Channel Subunit 2	Protein Coding	40	GC17M033013	2.181932
SYNJ2	Synaptojanin 2	Protein Coding	40	GC06P157981	2.176995
LRP12	LDL Receptor Related Protein 12	Protein Coding	40	GC08M104489	2.176916
COX6C	Cytochrome C Oxidase Subunit 6C	Protein Coding	40	GC08M099899	2.176744
ABCF1	ATP Binding Cassette Subfamily F Member 1	Protein Coding	40	GC06P030571	2.176427
BFSP2	Beaded Filament Structural Protein 2	Protein Coding	40	GC03P133399	2.168134
NDUFB6	NADH:Ubiquinone Oxidoreductase Subunit B6	Protein Coding	40	GC09M032553	2.164701
CAPN13	Calpain 13	Protein Coding	40	GC02M030722	2.16347
VSX2	Visual System Homeobox 2	Protein Coding	40	GC14P074239	2.163165
ANGPTL6	Angiopoietin Like 6	Protein Coding	40	GC19M010092	2.160412
DUSP13	Dual Specificity Phosphatase 13	Protein Coding	40	GC10M075094	2.158949
LRRC32	Leucine Rich Repeat Containing 32	Protein Coding	40	GC11M076657	2.154233
HPS6	HPS6 Biogenesis Of Lysosomal Organelles Complex 2 Subunit 3	Protein Coding	40	GC10P102065	2.152216
USP48	Ubiquitin Specific Peptidase 48	Protein Coding	40	GC01M021678	2.150779
NUP50	Nucleoporin 50	Protein Coding	40	GC22P045163	2.148602
BACE2	Beta-Secretase 2	Protein Coding	40	GC21P041168	2.138649
ATOH1	Atonal BHLH Transcription Factor 1	Protein Coding	40	GC04P093828	2.137344
INA	Internexin Neuronal Intermediate Filament Protein Alpha	Protein Coding	40	GC10P103277	2.135382
HNRNPDL	Heterogeneous Nuclear Ribonucleoprotein D Like	Protein Coding	40	GC04M082422	2.134372
PSD	Pleckstrin And Sec7 Domain Containing	Protein Coding	40	GC10M102403	2.129379
PDCD6	Programmed Cell Death 6	Protein Coding	40	GC05P000272	2.128097
CDS2	CDP-Diacylglycerol Synthase 2	Protein Coding	40	GC20P005126	2.126803
ABCE1	ATP Binding Cassette Subfamily E Member 1	Protein Coding	40	GC04P145097	2.114926
NDUFB7	NADH:Ubiquinone Oxidoreductase Subunit B7	Protein Coding	40	GC19M014566	2.114015
GSDMD	Gasdermin D	Protein Coding	40	GC08P143553	2.113107
ARHGEF16	Rho Guanine Nucleotide Exchange Factor 16	Protein Coding	40	GC01P003454	2.111348
MOXD1	Monooxygenase DBH Like 1	Protein Coding	40	GC06M132296	2.110557
IMMP2L	Inner Mitochondrial Membrane Peptidase Subunit 2	Protein Coding	40	GC07M110663	2.107028
DDX23	DEAD-Box Helicase 23	Protein Coding	40	GC12M048829	2.097578
NSUN4	NOP2/Sun RNA Methyltransferase 4	Protein Coding	40	GC01P046341	2.096464
USP12	Ubiquitin Specific Peptidase 12	Protein Coding	40	GC13M027066	2.095282
AZU1	Azurocidin 1	Protein Coding	40	GC19P000825	2.091982
PLA2R1	Phospholipase A2 Receptor 1	Protein Coding	40	GC02M159932	2.08907
IPO5	Importin 5	Protein Coding	40	GC13P097953	2.087946
COPS3	COP9 Signalosome Subunit 3	Protein Coding	40	GC17M017246	2.086375
SLC2A12	Solute Carrier Family 2 Member 12	Protein Coding	40	GC06M133987	2.081364
KCTD15	Potassium Channel Tetramerization Domain Containing 15	Protein Coding	40	GC19P033812	2.078815
IRS4	Insulin Receptor Substrate 4	Protein Coding	40	GC0XM108720	2.075074
LGR4	Leucine Rich Repeat Containing G Protein-Coupled Receptor 4	Protein Coding	40	GC11M027365	2.069799
SYMPK	Symplekin	Protein Coding	40	GC19M045815	2.069173

DSG3	Desmoglein 3	Protein Coding	40	GC18P031447	2.067504
STOM	Stomatin	Protein Coding	40	GC09M121338	2.06402
LCA5	Lebercilin LCA5	Protein Coding	40	GC06M079484	2.060655
WNK2	WNK Lysine Deficient Protein Kinase 2	Protein Coding	40	GC09P093184	2.058423
EIF3D	Eukaryotic Translation Initiation Factor 3 Subunit D	Protein Coding	40	GC22M036510	2.054898
RGN	Regucalcin	Protein Coding	40	GC0XP047189	2.050356
CHST10	Carbohydrate Sulfotransferase 10	Protein Coding	40	GC02M100391	2.038167
ELK3	ETS Transcription Factor ELK3	Protein Coding	40	GC12P096194	2.03321
PARVB	Parvin Beta	Protein Coding	40	GC22P043999	2.031839
NEIL2	Nei Like DNA Glycosylase 2	Protein Coding	40	GC08P011769	2.031561
DLX2	Distal-Less Homeobox 2	Protein Coding	40	GC02M172099	2.029769
SNRK	SNF Related Kinase	Protein Coding	40	GC03P043303	2.029488
STIM2	Stromal Interaction Molecule 2	Protein Coding	40	GC04P026859	2.021292
GINS1	GINS Complex Subunit 1	Protein Coding	40	GC20P025415	2.019546
STARD3	StAR Related Lipid Transfer Domain Containing 3	Protein Coding	40	GC17P039637	2.01662
COL8A1	Collagen Type VIII Alpha 1 Chain	Protein Coding	40	GC03P099638	2.014628
GEN1	GEN1 Holliday Junction 5' Flap Endonuclease	Protein Coding	40	GC02P017754	2.008627
HEPACAM	Hepatic And Glial Cell Adhesion Molecule	Protein Coding	40	GC11M124919	2.002409
DEFB1	Defensin Beta 1	Protein Coding	40	GC08M006870	2.000703
DPPA4	Developmental Pluripotency Associated 4	Protein Coding	40	GC03M109326	1.99389
DMBT1	Deleted In Malignant Brain Tumors 1	Protein Coding	40	GC10P122560	1.993376
TTLL5	Tubulin Tyrosine Ligase Like 5	Protein Coding	40	GC14P075633	1.992237
PSMD1	Proteasome 26S Subunit, Non-ATPase 1	Protein Coding	40	GC02P231056	1.989992
VPS25	Vacuolar Protein Sorting 25 Homolog	Protein Coding	40	GC17P042773	1.983504
RNASE1	Ribonuclease A Family Member 1, Pancreatic	Protein Coding	40	GC14M020801	1.98099
CACYBP	Calcyclin Binding Protein	Protein Coding	40	GC01P174968	1.974876
MARCKS	Myristoylated Alanine Rich Protein Kinase C Substrate	Protein Coding	40	GC06P113857	1.974645
SLAMF6	SLAM Family Member 6	Protein Coding	40	GC01M160454	1.972967
SF3B2	Splicing Factor 3b Subunit 2	Protein Coding	40	GC11P066050	1.963664
CLSPN	Claspin	Protein Coding	40	GC01M035720	1.956584
NAA15	N-Alpha-Acetyltransferase 15, NatA Auxiliary Subunit	Protein Coding	40	GC04P139301	1.955422
EGFL7	EGF Like Domain Multiple 7	Protein Coding	40	GC09P136658	1.950261
SYT7	Synaptotagmin 7	Protein Coding	40	GC11M061513	1.948028
CTDSPL	CTD Small Phosphatase Like	Protein Coding	40	GC03P037861	1.947487
AZIN1	Antizyme Inhibitor 1	Protein Coding	40	GC08M102826	1.945542
GGA2	Golgi Associated, Gamma Adaptin Ear Containing, ARF Binding Protein 2	Protein Coding	40	GC16M023464	1.943388
FAU	FAU Ubiquitin Like And Ribosomal Protein S30 Fusion	Protein Coding	40	GC11M065120	1.942667
NCKAP1	NCK Associated Protein 1	Protein Coding	40	GC02M182909	1.940047
SLC39A1	Solute Carrier Family 39 Member 1	Protein Coding	40	GC01M153960	1.936349
NLGN2	Neuroligin 2	Protein Coding	40	GC17P009089	1.936136
STAB2	Stabilin 2	Protein Coding	40	GC12P103587	1.935125
CHST12	Carbohydrate Sulfotransferase 12	Protein Coding	40	GC07P002403	1.9346
SLAMF1	Signaling Lymphocytic Activation Molecule Family Member 1	Protein Coding	40	GC01M160608	1.925656
POLM	DNA Polymerase Mu	Protein Coding	40	GC07M044143	1.923388
GIGYF2	GRB10 Interacting GYF Protein 2	Protein Coding	40	GC02P232698	1.923334
SENP2	SUMO Specific Peptidase 2	Protein Coding	40	GC03P185582	1.922148
CALCOCO2	Calcium Binding And Coiled-Coil Domain 2	Protein Coding	40	GC17P048830	1.921471
RRN3	RRN3 Homolog, RNA Polymerase I Transcription Factor	Protein Coding	40	GC16M015060	1.920301
GPRC6A	G Protein-Coupled Receptor Class C Group 6 Member A	Protein Coding	40	GC06M116793	1.916202
NUDT4	Nudix Hydrolase 4	Protein Coding	40	GC12P093392	1.908314
SNX2	Sorting Nexin 2	Protein Coding	40	GC05P122774	1.908289
ENTPD2	Ectonucleoside Triphosphate Diphosphohydrolase 2	Protein Coding	40	GC09M137048	1.905151
NTN4	Netrin 4	Protein Coding	40	GC12M095657	1.904203

ETV5	ETS Variant Transcription Factor 5	Protein Coding	40	GC03M186046	1.903677
MMRN1	Multimerin 1	Protein Coding	40	GC04P089879	1.902592
UBE4A	Ubiquitination Factor E4A	Protein Coding	40	GC11P118359	1.891393
IL34	Interleukin 34	Protein Coding	40	GC16P070656	1.891131
WDR48	WD Repeat Domain 48	Protein Coding	40	GC03P039052	1.888003
LRRK1	Leucine Rich Repeat Kinase 1	Protein Coding	40	GC15P100919	1.886927
KIF14	Kinesin Family Member 14	Protein Coding	40	GC01M200551	1.885134
TMOD3	Tropomodulin 3	Protein Coding	40	GC15P051829	1.884187
CYTH3	Cytohesin 3	Protein Coding	40	GC07M006161	1.878733
SOX14	SRY-Box Transcription Factor 14	Protein Coding	40	GC03P137764	1.873977
ACSL6	Acyl-CoA Synthetase Long Chain Family Member 6	Protein Coding	40	GC05M131949	1.873721
CCT4	Chaperonin Containing TCP1 Subunit 4	Protein Coding	40	GC02M061868	1.870541
ACSS1	Acyl-CoA Synthetase Short Chain Family Member 1	Protein Coding	40	GC20M024986	1.866293
ARTN	Artemin	Protein Coding	40	GC01P043933	1.860941
HSPA14	Heat Shock Protein Family A (Hsp70) Member 14	Protein Coding	40	GC10P014847	1.855625
RPL36	Ribosomal Protein L36	Protein Coding	40	GC19P005674	1.852962
PSMD8	Proteasome 26S Subunit, Non-ATPase 8	Protein Coding	40	GC19P038374	1.850593
KCNN1	Potassium Calcium-Activated Channel Subfamily N Member 1	Protein Coding	40	GC19P026648	1.8461
ORMDL3	ORMDL Sphingolipid Biosynthesis Regulator 3	Protein Coding	40	GC17M039921	1.838002
CETN3	Centrin 3	Protein Coding	40	GC05M090392	1.837263
PARP8	Poly(ADP-Ribose) Polymerase Family Member 8	Protein Coding	40	GC05P050665	1.836705
NPL	N-Acetylneuraminate Pyruvate Lyase	Protein Coding	40	GC01P182758	1.831124
RPL28	Ribosomal Protein L28	Protein Coding	40	GC19P056476	1.829793
PDCD5	Programmed Cell Death 5	Protein Coding	40	GC19P032581	1.820032
XYLB	Xylulokinase	Protein Coding	40	GC03P038363	1.818577
SPN	Sialophorin	Protein Coding	40	GC16P029662	1.816175
THEM4	Thioesterase Superfamily Member 4	Protein Coding	40	GC01M151870	1.814903
SRPK3	SRSF Protein Kinase 3	Protein Coding	40	GC0XP153776	1.81256
SRSF6	Serine And Arginine Rich Splicing Factor 6	Protein Coding	40	GC20P043457	1.809209
SLC25A17	Solute Carrier Family 25 Member 17	Protein Coding	40	GC22M048533	1.806607
CD7	CD7 Molecule	Protein Coding	40	GC17M082314	1.80539
USP28	Ubiquitin Specific Peptidase 28	Protein Coding	40	GC11M113797	1.804382
SESN1	Sestrin 1	Protein Coding	40	GC06M108986	1.788487
TGIF2	TGFB Induced Factor Homeobox 2	Protein Coding	40	GC20P036573	1.785674
LGALS13	Galectin 13	Protein Coding	40	GC19P039602	1.780903
HPS5	HPS5 Biogenesis Of Lysosomal Organelles Complex 2 Subunit 2	Protein Coding	40	GC11M018278	1.77861
PCOLCE	Procollagen C-Endopeptidase Enhancer	Protein Coding	40	GC07P100602	1.777273
BTN3A1	Butyrophilin Subfamily 3 Member A1	Protein Coding	40	GC06P026402	1.774847
CD276	CD276 Molecule	Protein Coding	40	GC15P073683	1.774778
LRG1	Leucine Rich Alpha-2-Glycoprotein 1	Protein Coding	40	GC19M004543	1.771306
AP3D1	Adaptor Related Protein Complex 3 Subunit Delta 1	Protein Coding	40	GC19M002223	1.769327
NLRC5	NLR Family CARD Domain Containing 5	Protein Coding	40	GC16P056990	1.765074
SEMA6A	Semaphorin 6A	Protein Coding	40	GC05M116443	1.764271
USF2	Upstream Transcription Factor 2, C-Fos Interacting	Protein Coding	40	GC19P035268	1.755463
NRXN2	Neurexin 2	Protein Coding	40	GC11M069335	1.750983
CDC5L	Cell Division Cycle 5 Like	Protein Coding	40	GC06P044387	1.74343
MYO10	Myosin X	Protein Coding	40	GC05M016661	1.741476
COX11	Cytochrome C Oxidase Copper Chaperone COX11	Protein Coding	40	GC17M054951	1.739748
DEPDC5	DEP Domain Containing 5, GATOR1 Subcomplex Subunit	Protein Coding	40	GC22P031753	1.737154
CDK18	Cyclin Dependent Kinase 18	Protein Coding	40	GC01P205504	1.736763
EBI3	Epstein-Barr Virus Induced 3	Protein Coding	40	GC19P004233	1.734586
TBL3	Transducin Beta Like 3	Protein Coding	40	GC16P005571	1.729745
FGF6	Fibroblast Growth Factor 6	Protein Coding	40	GC12M004429	1.728286

BZW2	Basic Leucine Zipper And W2 Domains 2	Protein Coding	40	GC07P016646	1.7262
EML1	EMAP Like 1	Protein Coding	40	GC14P099737	1.72575
B4GALNT2	Beta-1,4-N-Acetyl-Galactosaminyltransferase 2	Protein Coding	40	GC17P049132	1.72411
SLC25A18	Solute Carrier Family 25 Member 18	Protein Coding	40	GC22P017689	1.715596
GAS2	Growth Arrest Specific 2	Protein Coding	40	GC11P022626	1.710774
GOLPH3	Golgi Phosphoprotein 3	Protein Coding	40	GC05M032124	1.709915
PARP3	Poly(ADP-Ribose) Polymerase Family Member 3	Protein Coding	40	GC03P052036	1.708212
CCT6A	Chaperonin Containing TCP1 Subunit 6A	Protein Coding	40	GC07P056051	1.70389
HAS1	Hyaluronan Synthase 1	Protein Coding	40	GC19M051714	1.703727
RFX3	Regulatory Factor X3	Protein Coding	40	GC09M003214	1.702251
UBR2	Ubiquitin Protein Ligase E3 Component N-Recognin 2	Protein Coding	40	GC06P055398	1.700233
EXTL2	Exostosin Like Glycosyltransferase 2	Protein Coding	40	GC01M100872	1.697459
TDRKH	Tudor And KH Domain Containing	Protein Coding	40	GC01M151827	1.690692
HPS3	HPS3 Biogenesis Of Lysosomal Organelles Complex 2 Subunit 1	Protein Coding	40	GC03P149129	1.687017
IQCE	IQ Motif Containing E	Protein Coding	40	GC07P002558	1.686882
AHSA1	Activator Of HSP90 ATPase Activity 1	Protein Coding	40	GC14P077457	1.680059
LTB	Lymphotoxin Beta	Protein Coding	40	GC06M046882	1.679551
CPSF1	Cleavage And Polyadenylation Specific Factor 1	Protein Coding	40	GC08M144393	1.674399
ARHGAP15	Rho GTPase Activating Protein 15	Protein Coding	40	GC02P143070	1.673008
PARP9	Poly(ADP-Ribose) Polymerase Family Member 9	Protein Coding	40	GC03M122527	1.658417
LIMA1	LIM Domain And Actin Binding 1	Protein Coding	40	GC12M050175	1.655354
NPTX2	Neuronal Pentraxin 2	Protein Coding	40	GC07P098620	1.655312
SFRP5	Secreted Frizzled Related Protein 5	Protein Coding	40	GC10M097766	1.654312
OSBP	Oxysterol Binding Protein	Protein Coding	40	GC11M069113	1.651008
HS3ST2	Heparan Sulfate-Glucosamine 3-Sulfotransferase 2	Protein Coding	40	GC16P022814	1.650376
PRR5	Proline Rich 5	Protein Coding	40	GC22P044668	1.645131
INSIG1	Insulin Induced Gene 1	Protein Coding	40	GC07P155297	1.643924
CERKL	Ceramide Kinase Like	Protein Coding	40	GC02M181536	1.641706
P2RX5	Purinergic Receptor P2X 5	Protein Coding	40	GC17M003672	1.639605
HIF3A	Hypoxia Inducible Factor 3 Subunit Alpha	Protein Coding	40	GC19P046297	1.639411
TOR1AIP1	Torsin 1A Interacting Protein 1	Protein Coding	40	GC01P179882	1.637393
BLOC1S6	Biogenesis Of Lysosomal Organelles Complex 1 Subunit 6	Protein Coding	40	GC15P045588	1.635882
RFC3	Replication Factor C Subunit 3	Protein Coding	40	GC13P033818	1.634714
LILRB4	Leukocyte Immunoglobulin Like Receptor B4	Protein Coding	40	GC19P054643	1.634063
HOXC4	Homeobox C4	Protein Coding	40	GC12P054016	1.631279
BAIAP2L1	BAR/IMD Domain Containing Adaptor Protein 2 Like 1	Protein Coding	40	GC07M098294	1.629122
RAP2A	RAP2A, Member Of RAS Oncogene Family	Protein Coding	40	GC13P097436	1.627689
SLC7A10	Solute Carrier Family 7 Member 10	Protein Coding	40	GC19M033208	1.624462
GGA1	Golgi Associated, Gamma Adaptin Ear Containing, ARF Binding Protein 1	Protein Coding	40	GC22P037608	1.622433
NFRKB	Nuclear Factor Related To KappaB Binding Protein	Protein Coding	40	GC11M129863	1.620526
SIGLEC8	Sialic Acid Binding Ig Like Lectin 8	Protein Coding	40	GC19M051450	1.617892
ORM1	Orosomucoid 1	Protein Coding	40	GC09P114323	1.607037
NOVA1	NOVA Alternative Splicing Regulator 1	Protein Coding	40	GC14M026443	1.597659
SEMA3B	Semaphorin 3B	Protein Coding	40	GC03P050267	1.595743
AACS	Acetoacetyl-CoA Synthetase	Protein Coding	40	GC12P125065	1.594357
AAGAB	Alpha And Gamma Adaptin Binding Protein	Protein Coding	40	GC15M067200	1.594172
STK19	Serine/Threonine Kinase 19	Protein Coding	40	GC06P031971	1.582219
CREB3L3	CAMP Responsive Element Binding Protein 3 Like 3	Protein Coding	40	GC19P004153	1.580904
CLEC10A	C-Type Lectin Domain Containing 10A	Protein Coding	40	GC17M007074	1.577238
KCNIP3	Potassium Voltage-Gated Channel Interacting Protein 3	Protein Coding	40	GC02P095327	1.575697
SERPINB9	Serpin Family B Member 9	Protein Coding	40	GC06M002887	1.574493
ACYP2	Acylphosphatase 2	Protein Coding	40	GC02P053970	1.570706
SLC15A2	Solute Carrier Family 15 Member 2	Protein Coding	40	GC03P121894	1.568632

NAB1	NGFI-A Binding Protein 1	Protein Coding	40	GC02P190646	1.566214
GP5	Glycoprotein V Platelet	Protein Coding	40	GC03M194395	1.564105
RPH3A	Rabphilin 3A	Protein Coding	40	GC12P112570	1.558732
OSBPL5	Oxysterol Binding Protein Like 5	Protein Coding	40	GC11M003088	1.553821
ABCA8	ATP Binding Cassette Subfamily A Member 8	Protein Coding	40	GC17M068867	1.546915
TIAM2	TIAM Rac1 Associated GEF 2	Protein Coding	40	GC06P154832	1.546417
RCBTB1	RCC1 And BTB Domain Containing Protein 1	Protein Coding	40	GC13M049531	1.544497
MANF	Mesencephalic Astrocyte Derived Neurotrophic Factor	Protein Coding	40	GC03P051385	1.540499
KIR2DL4	Killer Cell Immunoglobulin Like Receptor, Two Ig Domains And Long Cytoplasmic Tail 4	Protein Coding	40	GC19P056446	1.53931
EPC1	Enhancer Of Polycomb Homolog 1	Protein Coding	40	GC10M032268	1.535802
LMTK2	Lemur Tyrosine Kinase 2	Protein Coding	40	GC07P098106	1.535751
TFDP2	Transcription Factor Dp-2	Protein Coding	40	GC03M141944	1.533971
CAPN11	Calpain 11	Protein Coding	40	GC06P055419	1.527709
UHRF1	Ubiquitin Like With PHD And Ring Finger Domains 1	Protein Coding	40	GC19P004910	1.526319
RAB39B	RAB39B, Member RAS Oncogene Family	Protein Coding	40	GC0XM155259	1.525211
TRIM22	Tripartite Motif Containing 22	Protein Coding	40	GC11P005689	1.523868
DNPEP	Aspartyl Aminopeptidase	Protein Coding	40	GC02M219373	1.52047
GALNT9	Polypeptide N-Acetylgalactosaminyltransferase 9	Protein Coding	40	GC12M132196	1.520127
LGALS9	Galectin 9	Protein Coding	40	GC17P027629	1.51846
RABEP1	Rabaptin, RAB GTPase Binding Effector Protein 1	Protein Coding	40	GC17P005282	1.510668
SLC16A4	Solute Carrier Family 16 Member 4	Protein Coding	40	GC01M110362	1.501627
MYBBP1A	MYB Binding Protein 1a	Protein Coding	40	GC17M004538	1.500581
ACIN1	Apoptotic Chromatin Condensation Inducer 1	Protein Coding	40	GC14M023058	1.49693
GZMH	Granzyme H	Protein Coding	40	GC14M024606	1.496504
RPL32	Ribosomal Protein L32	Protein Coding	40	GC03M012834	1.494273
EIF2A	Eukaryotic Translation Initiation Factor 2A	Protein Coding	40	GC03P150546	1.490098
NEU3	Neuraminidase 3	Protein Coding	40	GC11P074988	1.487984
NEU2	Neuraminidase 2	Protein Coding	40	GC02P233032	1.482925
NEIL1	Nei Like DNA Glycosylase 1	Protein Coding	40	GC15P075346	1.474468
TPRKB	TP53RK Binding Protein	Protein Coding	40	GC02M073730	1.46965
IFIT1	Interferon Induced Protein With Tetratricopeptide Repeats 1	Protein Coding	40	GC10P089396	1.469042
EIF3B	Eukaryotic Translation Initiation Factor 3 Subunit B	Protein Coding	40	GC07P002354	1.466162
SIGLEC7	Sialic Acid Binding Ig Like Lectin 7	Protein Coding	40	GC19P051142	1.460604
PIR	Pirin	Protein Coding	40	GC0XM015402	1.455899
NOX3	NADPH Oxidase 3	Protein Coding	40	GC06M155395	1.450855
SAV1	Salvador Family WW Domain Containing Protein 1	Protein Coding	40	GC14M050632	1.450659
C5AR2	Complement Component 5a Receptor 2	Protein Coding	40	GC19P047333	1.449766
SCUBE2	Signal Peptide, CUB Domain And EGF Like Domain Containing 2	Protein Coding	40	GC11M009019	1.449036
IL22RA1	Interleukin 22 Receptor Subunit Alpha 1	Protein Coding	40	GC01M024119	1.448395
SWAP70	Switching B Cell Complex Subunit SWAP70	Protein Coding	40	GC11P009664	1.444478
CPZ	Carboxypeptidase Z	Protein Coding	40	GC04P008583	1.44242
KDELR1	KDEL Endoplasmic Reticulum Protein Retention Receptor 1	Protein Coding	40	GC19M048382	1.44239
STAMBPL1	STAM Binding Protein Like 1	Protein Coding	40	GC10P088879	1.441415
RAD54L2	RAD54 Like 2	Protein Coding	40	GC03P051538	1.439955
PDLIM7	PDZ And LIM Domain 7	Protein Coding	40	GC05M177483	1.438821
IFIT2	Interferon Induced Protein With Tetratricopeptide Repeats 2	Protein Coding	40	GC10P089284	1.431812
ARF3	ADP Ribosylation Factor 3	Protein Coding	40	GC12M049034	1.430409
SLC9A9	Solute Carrier Family 9 Member A9	Protein Coding	40	GC03M143265	1.429197
PRX	Periaxin	Protein Coding	40	GC19M040393	1.428805
GAN	Gigaxonin	Protein Coding	40	GC16P081319	1.428718
KLHL2	Kelch Like Family Member 2	Protein Coding	40	GC04P165207	1.425846
FARP2	FERM, ARH/RhoGEF And Pleckstrin Domain Protein 2	Protein Coding	40	GC02P241357	1.424138
CRHBP	Corticotropin Releasing Hormone Binding Protein	Protein Coding	40	GC05P076952	1.420643

ABCG4	ATP Binding Cassette Subfamily G Member 4	Protein Coding	40	GC11P119150	1.419999
BST2	Bone Marrow Stromal Cell Antigen 2	Protein Coding	40	GC19M017403	1.418601
NAPRT	Nicotinate Phosphoribosyltransferase	Protein Coding	40	GC08M143574	1.41723
PJA1	Praja Ring Finger Ubiquitin Ligase 1	Protein Coding	40	GC0XM069160	1.414705
SAT2	Spermidine/Spermine N1-Acetyltransferase Family Member 2	Protein Coding	40	GC17M007626	1.409705
CC2D1A	Coiled-Coil And C2 Domain Containing 1A	Protein Coding	40	GC19P013907	1.401137
MLANA	Melan-A	Protein Coding	40	GC09P005899	1.400994
CPSF6	Cleavage And Polyadenylation Specific Factor 6	Protein Coding	40	GC12P069239	1.399291
TRAK2	Trafficking Kinesin Protein 2	Protein Coding	40	GC02M201377	1.392657
ADARB2	Adenosine Deaminase RNA Specific B2 (Inactive)	Protein Coding	40	GC10M001187	1.387382
PAG1	Phosphoprotein Membrane Anchor With Glycosphingolipid Microdomains 1	Protein Coding	40	GC08M080967	1.387234
KIAA0319	KIAA0319	Protein Coding	40	GC06M024544	1.38634
RAB11FIP5	RAB11 Family Interacting Protein 5	Protein Coding	40	GC02M073128	1.385744
GP2	Glycoprotein 2	Protein Coding	40	GC16M020309	1.38405
GTF2H2	General Transcription Factor IIH Subunit 2	Protein Coding	40	GC05M071032	1.383702
CDC16	Cell Division Cycle 16	Protein Coding	40	GC13P114234	1.383375
KCNS2	Potassium Voltage-Gated Channel Modifier Subfamily S Member 2	Protein Coding	40	GC08P098427	1.38045
GLCE	Glucuronic Acid Epimerase	Protein Coding	40	GC15P077108	1.378603
REPS1	RALBP1 Associated Eps Domain Containing 1	Protein Coding	40	GC06M138903	1.377624
SEMA4B	Semaphorin 4B	Protein Coding	40	GC15P090160	1.375879
POP4	POP4 Homolog, Ribonuclease P/MRP Subunit	Protein Coding	40	GC19P029604	1.368983
KRT86	Keratin 86	Protein Coding	40	GC12P052249	1.368324
RBBP5	RB Binding Protein 5, Histone Lysine Methyltransferase Complex Subunit	Protein Coding	40	GC01M205055	1.365414
SFXN2	Sideroflexin 2	Protein Coding	40	GC10P102714	1.364958
IL20	Interleukin 20	Protein Coding	40	GC01P206866	1.364349
DAAM2	Dishevelled Associated Activator Of Morphogenesis 2	Protein Coding	40	GC06P039792	1.363731
RASAL1	RAS Protein Activator Like 1	Protein Coding	40	GC12M113098	1.357955
CARD10	Caspase Recruitment Domain Family Member 10	Protein Coding	40	GC22M048517	1.356193
CPN2	Carboxypeptidase N Subunit 2	Protein Coding	40	GC03M194339	1.355953
HPS4	HPS4 Biogenesis Of Lysosomal Organelles Complex 3 Subunit 2	Protein Coding	40	GC22M026443	1.355371
HOXA9	Homeobox A9	Protein Coding	40	GC07M027162	1.347407
TNFRSF10C	TNF Receptor Superfamily Member 10c	Protein Coding	40	GC08P023102	1.347379
POU3F4	POU Class 3 Homeobox 4	Protein Coding	40	GC0XP083508	1.347024
KCNT2	Potassium Sodium-Activated Channel Subfamily T Member 2	Protein Coding	40	GC01M196225	1.34564
RPL24	Ribosomal Protein L24	Protein Coding	40	GC03M101681	1.338475
LAMP3	Lysosomal Associated Membrane Protein 3	Protein Coding	40	GC03M183122	1.337891
MAS1	MAS1 Proto-Oncogene, G Protein-Coupled Receptor	Protein Coding	40	GC06P159906	1.334837
PIPOX	Pipecolic Acid And Sarcosine Oxidase	Protein Coding	40	GC17P029371	1.334077
CD207	CD207 Molecule	Protein Coding	40	GC02M070830	1.33267
MGAT4B	Alpha-1,3-Mannosyl-Glycoprotein 4-Beta-N-Acetylglucosaminyltransferase B	Protein Coding	40	GC05M179797	1.332099
TUBGCP6	Tubulin Gamma Complex Associated Protein 6	Protein Coding	40	GC22M050217	1.331733
LSM4	LSM4 Homolog, U6 Small Nuclear RNA And MRNA Degradation Associated	Protein Coding	40	GC19M018306	1.331031
CDH10	Cadherin 10	Protein Coding	40	GC05M024522	1.329008
DLX4	Distal-Less Homeobox 4	Protein Coding	40	GC17P049968	1.328249
CAPN7	Calpain 7	Protein Coding	40	GC03P015950	1.328072
TXNRD3	Thioredoxin Reductase 3	Protein Coding	40	GC03M126577	1.32574
CXCL6	C-X-C Motif Chemokine Ligand 6	Protein Coding	40	GC04P073837	1.325641
HNRNPR	Heterogeneous Nuclear Ribonucleoprotein R	Protein Coding	40	GC01M023303	1.323614
MT3	Metallothionein 3	Protein Coding	40	GC16P056589	1.323379
EPS8L2	EPS8 Like 2	Protein Coding	40	GC11P000694	1.321892
MTMR6	Myotubularin Related Protein 6	Protein Coding	40	GC13M025246	1.31775
PLXNB3	Plexin B3	Protein Coding	40	GC0XP153764	1.317584
NIF3L1	NGG1 Interacting Factor 3 Like 1	Protein Coding	40	GC02P200889	1.317212

KLK7	Kallikrein Related Peptidase 7	Protein Coding	40	GC19M050977	1.312988
CD300LF	CD300 Molecule Like Family Member F	Protein Coding	40	GC17M074694	1.310721
NIT2	Nitrilase Family Member 2	Protein Coding	40	GC03P100334	1.307184
BAG5	BAG Cochaperone 5	Protein Coding	40	GC14M103556	1.307144
DLX6	Distal-Less Homeobox 6	Protein Coding	40	GC07P097005	1.302898
PTMA	Prothymosin Alpha	Protein Coding	40	GC02P231707	1.302852
RASA3	RAS P21 Protein Activator 3	Protein Coding	40	GC13M113977	1.300854
TAF4	TATA-Box Binding Protein Associated Factor 4	Protein Coding	40	GC20M061994	1.299297
KIFC1	Kinesin Family Member C1	Protein Coding	40	GC06P033391	1.297226
TXNL1	Thioredoxin Like 1	Protein Coding	40	GC18M056597	1.297082
PLA1A	Phospholipase A1 Member A	Protein Coding	40	GC03P119597	1.296894
ALPK1	Alpha Kinase 1	Protein Coding	40	GC04P112285	1.291345
METTL3	Methyltransferase Like 3	Protein Coding	40	GC14M021498	1.290836
COPZ1	COPI Coat Complex Subunit Zeta 1	Protein Coding	40	GC12P054301	1.286867
MOB1A	MOB Kinase Activator 1A	Protein Coding	40	GC02M074152	1.286797
STEAP1	STEAP Family Member 1	Protein Coding	40	GC07P090154	1.285675
NEK11	NIMA Related Kinase 11	Protein Coding	40	GC03P131026	1.285209
COX17	Cytochrome C Oxidase Copper Chaperone COX17	Protein Coding	40	GC03M119654	1.281864
MSI1	Musashi RNA Binding Protein 1	Protein Coding	40	GC12M120341	1.280927
TAF11	TATA-Box Binding Protein Associated Factor 11	Protein Coding	40	GC06M047019	1.280003
GTF2H3	General Transcription Factor IIH Subunit 3	Protein Coding	40	GC12P123633	1.277312
LCN1	Lipocalin 1	Protein Coding	40	GC09P135521	1.273111
DOK5	Docking Protein 5	Protein Coding	40	GC20P054476	1.271607
MICU1	Mitochondrial Calcium Uptake 1	Protein Coding	40	GC10M072367	1.265147
CDC23	Cell Division Cycle 23	Protein Coding	40	GC05M138198	1.264873
SIX4	SIX Homeobox 4	Protein Coding	40	GC14M060709	1.262277
FXR2	FMR1 Autosomal Homolog 2	Protein Coding	40	GC17M007592	1.261492
SF3A1	Splicing Factor 3a Subunit 1	Protein Coding	40	GC22M030331	1.261128
KCNK6	Potassium Two Pore Domain Channel Subfamily K Member 6	Protein Coding	40	GC19P038319	1.258486
NRG4	Neuregulin 4	Protein Coding	40	GC15M075935	1.258352
PCTP	Phosphatidylcholine Transfer Protein	Protein Coding	40	GC17P055750	1.258269
PHIP	Pleckstrin Homology Domain Interacting Protein	Protein Coding	40	GC06M078934	1.251297
RPL23	Ribosomal Protein L23	Protein Coding	40	GC17M038847	1.249622
STK40	Serine/Threonine Kinase 40	Protein Coding	40	GC01M036339	1.248947
NUAK2	NUAK Family Kinase 2	Protein Coding	40	GC01M205302	1.244793
CST6	Cystatin E/M	Protein Coding	40	GC11P066311	1.244318
SHC2	SHC Adaptor Protein 2	Protein Coding	40	GC19M000872	1.244237
UXS1	UDP-Glucuronate Decarboxylase 1	Protein Coding	40	GC02M106094	1.243628
GABRG1	Gamma-Aminobutyric Acid Type A Receptor Subunit Gamma1	Protein Coding	40	GC04M046035	1.243133
TSPAN8	Tetraspanin 8	Protein Coding	40	GC12M071125	1.242929
SLC16A10	Solute Carrier Family 16 Member 10	Protein Coding	40	GC06P111087	1.241387
CCDC6	Coiled-Coil Domain Containing 6	Protein Coding	40	GC10M059788	1.236772
TMEFF2	Transmembrane Protein With EGF Like And Two Follistatin Like Domains 2	Protein Coding	40	GC02M191950	1.232994
CXXC4	CXXC Finger Protein 4	Protein Coding	40	GC04M104468	1.22828
APOL2	Apolipoprotein L2	Protein Coding	40	GC22M036226	1.226899
RPL18A	Ribosomal Protein L18a	Protein Coding	40	GC19P026644	1.226899
SMC4	Structural Maintenance Of Chromosomes 4	Protein Coding	40	GC03P160399	1.224269
USP30	Ubiquitin Specific Peptidase 30	Protein Coding	40	GC12P109027	1.223373
STK26	Serine/Threonine Kinase 26	Protein Coding	40	GC0XP132023	1.218763
SLC25A29	Solute Carrier Family 25 Member 29	Protein Coding	40	GC14M100280	1.218569
RBM17	RNA Binding Motif Protein 17	Protein Coding	40	GC10P006088	1.216172
NRN1	Neuritin 1	Protein Coding	40	GC06M005997	1.21533
ORC2	Origin Recognition Complex Subunit 2	Protein Coding	40	GC02M200908	1.213348

PKN3	Protein Kinase N3	Protein Coding	40	GC09P128702	1.209458
STMN2	Stathmin 2	Protein Coding	40	GC08P079610	1.202911
AATF	Apoptosis Antagonizing Transcription Factor	Protein Coding	40	GC17P036948	1.197864
SASH1	SAM And SH3 Domain Containing 1	Protein Coding	40	GC06P148193	1.194192
MARVELD2	MARVEL Domain Containing 2	Protein Coding	40	GC05P069415	1.192826
FGD3	FYVE, RhoGEF And PH Domain Containing 3	Protein Coding	40	GC09P092947	1.190094
RFK	Riboflavin Kinase	Protein Coding	40	GC09M076385	1.190094
LAT2	Linker For Activation Of T Cells Family Member 2	Protein Coding	40	GC07P074199	1.184164
CDCA2	Cell Division Cycle Associated 2	Protein Coding	40	GC08P025458	1.181585
SLCO1A2	Solute Carrier Organic Anion Transporter Family Member 1A2	Protein Coding	40	GC12M021264	1.181377
ACBD5	Acyl-CoA Binding Domain Containing 5	Protein Coding	40	GC10M027182	1.180781
PCDH12	Protocadherin 12	Protein Coding	40	GC05M141943	1.180199
DNMBP	Dynamin Binding Protein	Protein Coding	40	GC10M099875	1.177526
RAP1GAP	RAP1 GTPase Activating Protein	Protein Coding	40	GC01M021596	1.160569
GABRP	Gamma-Aminobutyric Acid Type A Receptor Subunit Pi	Protein Coding	40	GC05P170763	1.157564
PUM2	Pumilio RNA Binding Family Member 2	Protein Coding	40	GC02M020273	1.157308
RPL37	Ribosomal Protein L37	Protein Coding	40	GC05M040825	1.152994
NBL1	NBL1, DAN Family BMP Antagonist	Protein Coding	40	GC01P019600	1.152059
THOC2	THO Complex 2	Protein Coding	40	GC0XM123600	1.150234
IMPG1	Interphotoreceptor Matrix Proteoglycan 1	Protein Coding	40	GC06M075921	1.147971
CD160	CD160 Molecule	Protein Coding	40	GC01P145719	1.1476
ZFP36	ZFP36 Ring Finger Protein	Protein Coding	40	GC19P039406	1.145049
TRIM29	Tripartite Motif Containing 29	Protein Coding	40	GC11M120111	1.14207
CTDSP2	CTD Small Phosphatase 2	Protein Coding	40	GC12M057819	1.135625
NCR2	Natural Cytotoxicity Triggering Receptor 2	Protein Coding	40	GC06P055384	1.134475
WTAP	WT1 Associated Protein	Protein Coding	40	GC06P159725	1.131924
DDX25	DEAD-Box Helicase 25	Protein Coding	40	GC11P125903	1.130582
SLCO1C1	Solute Carrier Organic Anion Transporter Family Member 1C1	Protein Coding	40	GC12P020695	1.128682
UBASH3A	Ubiquitin Associated And SH3 Domain Containing A	Protein Coding	40	GC21P042403	1.125607
LSM1	LSM1 Homolog, MRNA Degradation Associated	Protein Coding	40	GC08M038163	1.121137
TGS1	Trimethylguanosine Synthase 1	Protein Coding	40	GC08P055773	1.117591
AANAT	Aralkylamine N-Acetyltransferase	Protein Coding	40	GC17P076453	1.115726
CLC	Charcot-Leyden Crystal Galectin	Protein Coding	40	GC19M046744	1.111272
ULK3	Unc-51 Like Kinase 3	Protein Coding	40	GC15M074836	1.109167
SYN2	Synapsin II	Protein Coding	40	GC03P012020	1.108422
REG4	Regenerating Family Member 4	Protein Coding	40	GC01M119794	1.106986
SLC39A10	Solute Carrier Family 39 Member 10	Protein Coding	40	GC02P195575	1.104382
PPP3R2	Protein Phosphatase 3 Regulatory Subunit B, Beta	Protein Coding	40	GC09M101591	1.10262
CCT8	Chaperonin Containing TCP1 Subunit 8	Protein Coding	40	GC21M029055	1.095616
TMC8	Transmembrane Channel Like 8	Protein Coding	40	GC17P078130	1.095185
SEMA4G	Semaphorin 4G	Protein Coding	40	GC10P100969	1.089738
ABCF2	ATP Binding Cassette Subfamily F Member 2	Protein Coding	40	GC07M151211	1.087911
MTIF2	Mitochondrial Translational Initiation Factor 2	Protein Coding	40	GC02M055236	1.08784
SCMH1	Scm Polycomb Group Protein Homolog 1	Protein Coding	40	GC01M041027	1.080637
LILRB3	Leukocyte Immunoglobulin Like Receptor B3	Protein Coding	40	GC19M054216	1.078749
PFDN5	Prefoldin Subunit 5	Protein Coding	40	GC12P053295	1.078132
ING3	Inhibitor Of Growth Family Member 3	Protein Coding	40	GC07P120950	1.074976
PELP1	Proline, Glutamate And Leucine Rich Protein 1	Protein Coding	40	GC17M004669	1.074405
PLD4	Phospholipase D Family Member 4	Protein Coding	40	GC14P104924	1.072676
ZMYND8	Zinc Finger MYND-Type Containing 8	Protein Coding	40	GC20M047209	1.071845
SF3B3	Splicing Factor 3b Subunit 3	Protein Coding	40	GC16P070523	1.067975
KRT15	Keratin 15	Protein Coding	40	GC17M041513	1.063879
PSME4	Proteasome Activator Subunit 4	Protein Coding	40	GC02M053864	1.061084

DUSP22	Dual Specificity Phosphatase 22	Protein Coding	40	GC06P000292	1.05898
SLITRK1	SLIT And NTRK Like Family Member 1	Protein Coding	40	GC13M083877	1.058685
FKBP2	FKBP Prolyl Isomerase 2	Protein Coding	40	GC11P064240	1.058508
GIP	Gastric Inhibitory Polypeptide	Protein Coding	40	GC17M048958	1.058312
RSRC1	Arginine And Serine Rich Coiled-Coil 1	Protein Coding	40	GC03P158105	1.056873
ART3	ADP-Ribosyltransferase 3 (Inactive)	Protein Coding	40	GC04P076011	1.053474
BCAR3	BCAR3 Adaptor Protein, NSP Family Member	Protein Coding	40	GC01M093561	1.053313
CEP250	Centrosomal Protein 250	Protein Coding	40	GC20P035455	1.050416
ICAM5	Intercellular Adhesion Molecule 5	Protein Coding	40	GC19P010289	1.046906
GOSR1	Golgi SNAP Receptor Complex Member 1	Protein Coding	40	GC17P030477	1.043954
SPG21	SPG21 Abhydrolase Domain Containing, Maspardin	Protein Coding	40	GC15M064963	1.042937
VPS26A	VPS26, Retromer Complex Component A	Protein Coding	40	GC10P069123	1.041264
SLC39A6	Solute Carrier Family 39 Member 6	Protein Coding	40	GC18M036108	1.040781
CPSF4	Cleavage And Polyadenylation Specific Factor 4	Protein Coding	40	GC07P099438	1.034202
RGS16	Regulator Of G Protein Signaling 16	Protein Coding	40	GC01M182598	1.03128
DLX1	Distal-Less Homeobox 1	Protein Coding	40	GC02P172084	1.027124
POU4F3	POU Class 4 Homeobox 3	Protein Coding	40	GC05P146339	1.025195
ANAPC11	Anaphase Promoting Complex Subunit 11	Protein Coding	40	GC17P081890	1.024971
TSNAX	Translin Associated Factor X	Protein Coding	40	GC01P231529	1.023819
SUMO2	Small Ubiquitin Like Modifier 2	Protein Coding	40	GC17M075165	1.020728
TIPIN	TIMELESS Interacting Protein	Protein Coding	40	GC15M067420	1.019058
OPTC	Opticin	Protein Coding	40	GC01P203494	1.016699
MUC2	Mucin 2, Oligomeric Mucus/Gel-Forming	Protein Coding	40	GC11P001074	1.016066
CYP4F11	Cytochrome P450 Family 4 Subfamily F Member 11	Protein Coding	40	GC19M015912	1.012497
MMP25	Matrix Metallopeptidase 25	Protein Coding	40	GC16P005616	1.010622
AOC2	Amine Oxidase Copper Containing 2	Protein Coding	40	GC17P042844	1.001701
EMB	Embigin	Protein Coding	40	GC05M050396	0.996168
VIL1	Villin 1	Protein Coding	40	GC02P218419	0.99416
ACSM1	Acyl-CoA Synthetase Medium Chain Family Member 1	Protein Coding	40	GC16M020634	0.993797
LY6E	Lymphocyte Antigen 6 Family Member E	Protein Coding	40	GC08P143017	0.992629
VTI1A	Vesicle Transport Through Interaction With T-SNAREs 1A	Protein Coding	40	GC10P112446	0.988924
ISG20	Interferon Stimulated Exonuclease Gene 20	Protein Coding	40	GC15P088635	0.97767
SRSF7	Serine And Arginine Rich Splicing Factor 7	Protein Coding	40	GC02M038743	0.975059
PTPN21	Protein Tyrosine Phosphatase Non-Receptor Type 21	Protein Coding	40	GC14M088465	0.966912
RRBP1	Ribosome Binding Protein 1	Protein Coding	40	GC20M017613	0.958943
NCOA4	Nuclear Receptor Coactivator 4	Protein Coding	40	GC10M046005	0.958933
EDF1	Endothelial Differentiation Related Factor 1	Protein Coding	40	GC09M136862	0.956379
CLASP2	Cytoplasmic Linker Associated Protein 2	Protein Coding	40	GC03M033537	0.951799
STX5	Syntaxin 5	Protein Coding	40	GC11M062806	0.95049
RPL14	Ribosomal Protein L14	Protein Coding	40	GC03P040458	0.948735
CD99	CD99 Molecule (Xg Blood Group)	Protein Coding	40	GC0XP002691	0.944295
FOXA3	Forkhead Box A3	Protein Coding	40	GC19P045863	0.94303
PCDH8	Protocadherin 8	Protein Coding	40	GC13M052842	0.938413
TRAM1	Translocation Associated Membrane Protein 1	Protein Coding	40	GC08M070573	0.938058
RHBG	Rh Family B Glycoprotein	Protein Coding	40	GC01P156366	0.935599
DDX18	DEAD-Box Helicase 18	Protein Coding	40	GC02P117913	0.93539
CD300A	CD300a Molecule	Protein Coding	40	GC17P074466	0.926958
S100A3	S100 Calcium Binding Protein A3	Protein Coding	40	GC01M153547	0.926899
PRRT2	Proline Rich Transmembrane Protein 2	Protein Coding	40	GC16P029811	0.924274
ZMYM2	Zinc Finger MYM-Type Containing 2	Protein Coding	40	GC13P019958	0.924264
CEP170	Centrosomal Protein 170	Protein Coding	40	GC01M243124	0.923779
AK8	Adenylate Kinase 8	Protein Coding	40	GC09M132725	0.920931
DDX46	DEAD-Box Helicase 46	Protein Coding	40	GC05P134758	0.919869

IL1F10	Interleukin 1 Family Member 10	Protein Coding	40	GC02P113067	0.91479
TTL	Tubulin Tyrosine Ligase	Protein Coding	40	GC02P116681	0.910765
UROC1	Urocanate Hydratase 1	Protein Coding	40	GC03M126481	0.900378
PSMF1	Proteasome Inhibitor Subunit 1	Protein Coding	40	GC20P001113	0.897637
POP1	POP1 Homolog, Ribonuclease P/MRP Subunit	Protein Coding	40	GC08P098117	0.89574
CORO1C	Coronin 1C	Protein Coding	40	GC12M108645	0.89397
GAS7	Growth Arrest Specific 7	Protein Coding	40	GC17M009910	0.891576
CDKL3	Cyclin Dependent Kinase Like 3	Protein Coding	40	GC05M134242	0.887043
SLC17A1	Solute Carrier Family 17 Member 1	Protein Coding	40	GC06M025723	0.884346
FAIM	Fas Apoptotic Inhibitory Molecule	Protein Coding	40	GC03P138608	0.878508
CPNE3	Copine 3	Protein Coding	40	GC08P086514	0.877296
PMAIP1	Phorbol-12-Myristate-13-Acetate-Induced Protein 1	Protein Coding	40	GC18P059899	0.866262
MYO1D	Myosin ID	Protein Coding	40	GC17M032492	0.864671
KRT9	Keratin 9	Protein Coding	40	GC17M041565	0.863864
AP2A1	Adaptor Related Protein Complex 2 Subunit Alpha 1	Protein Coding	40	GC19P049766	0.861259
FPR3	Formyl Peptide Receptor 3	Protein Coding	40	GC19P051795	0.859179
ENOPH1	Enolase-Phosphatase 1	Protein Coding	40	GC04P082430	0.85771
XCR1	X-C Motif Chemokine Receptor 1	Protein Coding	40	GC03M046016	0.857277
STAB1	Stabilin 1	Protein Coding	40	GC03P052495	0.852123
CHST5	Carbohydrate Sulfotransferase 5	Protein Coding	40	GC16M075528	0.848512
TAS1R3	Taste 1 Receptor Member 3	Protein Coding	40	GC01P001331	0.848133
TUFT1	Tuftelin 1	Protein Coding	40	GC01P151513	0.847002
ACSBG1	Acyl-CoA Synthetase Bubblegum Family Member 1	Protein Coding	40	GC15M078167	0.835055
DDX50	DExD-Box Helicase 50	Protein Coding	40	GC10P068901	0.834723
ABCA6	ATP Binding Cassette Subfamily A Member 6	Protein Coding	40	GC17M069062	0.832467
PSIP1	PC4 And SFRS1 Interacting Protein 1	Protein Coding	40	GC09M015464	0.825898
CERS2	Ceramide Synthase 2	Protein Coding	40	GC01M150934	0.82371
RTKN	Rhotekin	Protein Coding	40	GC02M074583	0.822383
NPTN	Neuroplastin	Protein Coding	40	GC15M073560	0.820888
KATNA1	Katanin Catalytic Subunit A1	Protein Coding	40	GC06M149594	0.813684
PIGH	Phosphatidylinositol Glycan Anchor Biosynthesis Class H	Protein Coding	40	GC14M067581	0.812737
ILF2	Interleukin Enhancer Binding Factor 2	Protein Coding	40	GC01M153661	0.811287
RIMS2	Regulating Synaptic Membrane Exocytosis 2	Protein Coding	40	GC08P103500	0.795806
GPR18	G Protein-Coupled Receptor 18	Protein Coding	40	GC13M099254	0.788823
SLC30A4	Solute Carrier Family 30 Member 4	Protein Coding	40	GC15M045479	0.786455
TGM4	Transglutaminase 4	Protein Coding	40	GC03P044874	0.778646
ARIH2	Ariadne RBR E3 Ubiquitin Protein Ligase 2	Protein Coding	40	GC03P048918	0.778051
CES3	Carboxylesterase 3	Protein Coding	40	GC16P066963	0.77772
ACMSD	Aminocarboxymuconate Semialdehyde Decarboxylase	Protein Coding	40	GC02P134838	0.774628
AKAP5	A-Kinase Anchoring Protein 5	Protein Coding	40	GC14P064465	0.759567
CA7	Carbonic Anhydrase 7	Protein Coding	40	GC16P066844	0.7579
PLA2G4D	Phospholipase A2 Group IVD	Protein Coding	40	GC15M042067	0.756887
SV2C	Synaptic Vesicle Glycoprotein 2C	Protein Coding	40	GC05P075847	0.750347
PTPRR	Protein Tyrosine Phosphatase Receptor Type R	Protein Coding	40	GC12M070638	0.747357
HOXA4	Homeobox A4	Protein Coding	40	GC07M027128	0.73872
GH2	Growth Hormone 2	Protein Coding	40	GC17M063880	0.735237
TMEM59	Transmembrane Protein 59	Protein Coding	40	GC01M054031	0.73143
EN2	Engrailed Homeobox 2	Protein Coding	40	GC07P155459	0.729049
PPM1F	Protein Phosphatase, Mg2+/Mn2+ Dependent 1F	Protein Coding	40	GC22M021919	0.728886
KSR2	Kinase Suppressor Of Ras 2	Protein Coding	40	GC12M117453	0.727448
GLIPR1	GLI Pathogenesis Related 1	Protein Coding	40	GC12P075480	0.724552
SH2D1B	SH2 Domain Containing 1B	Protein Coding	40	GC01M162395	0.709265
NINJ1	Ninjurin 1	Protein Coding	40	GC09M093121	0.708757

SFXN1	Sideroflexin 1	Protein Coding	40	GC05P175477	0.700908
MTA2	Metastasis Associated 1 Family Member 2	Protein Coding	40	GC11M069248	0.691633
SIRPB1	Signal Regulatory Protein Beta 1	Protein Coding	40	GC20M001563	0.691541
OVGP1	Oviductal Glycoprotein 1	Protein Coding	40	GC01M111414	0.691541
SLC27A3	Solute Carrier Family 27 Member 3	Protein Coding	40	GC01P153786	0.688975
ST13	ST13 Hsp70 Interacting Protein	Protein Coding	40	GC22M048534	0.68553
TNFSF18	TNF Superfamily Member 18	Protein Coding	40	GC01M173009	0.68056
GRK7	G Protein-Coupled Receptor Kinase 7	Protein Coding	40	GC03P141778	0.680547
SLC45A3	Solute Carrier Family 45 Member 3	Protein Coding	40	GC01M205626	0.67783
CELSR3	Cadherin EGF LAG Seven-Pass G-Type Receptor 3	Protein Coding	40	GC03M048641	0.675741
MLLT1	MLLT1 Super Elongation Complex Subunit	Protein Coding	40	GC19M006210	0.666875
TRA2A	Transformer 2 Alpha Homolog	Protein Coding	40	GC07M023504	0.663186
GCHFR	GTP Cyclohydrolase I Feedback Regulator	Protein Coding	40	GC15P040771	0.659778
OLFM1	Olfactomedin 1	Protein Coding	40	GC09P135075	0.645742
SHC3	SHC Adaptor Protein 3	Protein Coding	40	GC09M089005	0.641684
BMF	Bcl2 Modifying Factor	Protein Coding	40	GC15M040087	0.635757
CHMP2A	Charged Multivesicular Body Protein 2A	Protein Coding	40	GC19M058551	0.629364
KLK10	Kallikrein Related Peptidase 10	Protein Coding	40	GC19M051012	0.627121
FFAR1	Free Fatty Acid Receptor 1	Protein Coding	40	GC19P040639	0.619412
CHMP1B	Charged Multivesicular Body Protein 1B	Protein Coding	40	GC18P011851	0.610606
BIRC7	Baculoviral IAP Repeat Containing 7	Protein Coding	40	GC20P063235	0.606342
ARHGEF5	Rho Guanine Nucleotide Exchange Factor 5	Protein Coding	40	GC07P144355	0.602194
SOSTDC1	Sclerostin Domain Containing 1	Protein Coding	40	GC07M016467	0.596362
OAS2	2'-5'-Oligoadenylate Synthetase 2	Protein Coding	40	GC12P112978	0.594717
ELF3	E74 Like ETS Transcription Factor 3	Protein Coding	40	GC01P202007	0.591021
ATG9A	Autophagy Related 9A	Protein Coding	40	GC02M219219	0.589217
GALNT4	Polypeptide N-Acetylgalactosaminyltransferase 4	Protein Coding	40	GC12M089520	0.58453
ATP13A1	ATPase 13A1	Protein Coding	40	GC19M019645	0.570955
RAB25	RAB25, Member RAS Oncogene Family	Protein Coding	40	GC01P156061	0.565584
FAM3C	FAM3 Metabolism Regulating Signaling Molecule C	Protein Coding	40	GC07M121349	0.562741
FNBP1	Formin Binding Protein 1	Protein Coding	40	GC09M129887	0.557847
KRT81	Keratin 81	Protein Coding	40	GC12M052286	0.547905
SHPRH	SNF2 Histone Linker PHD RING Helicase	Protein Coding	40	GC06M145863	0.544563
CSTF2	Cleavage Stimulation Factor Subunit 2	Protein Coding	40	GC0XP100820	0.527828
LALBA	Lactalbumin Alpha	Protein Coding	40	GC12M048567	0.513427
LILRB5	Leukocyte Immunoglobulin Like Receptor B5	Protein Coding	40	GC19M054553	0.513427
NPRL2	NPR2 Like, GATOR1 Complex Subunit	Protein Coding	40	GC03M050432	0.508507
COLEC12	Collectin Subfamily Member 12	Protein Coding	40	GC18M000318	0.502496
THAP1	THAP Domain Containing 1	Protein Coding	40	GC08M042836	0.500799
UNC5A	Unc-5 Netrin Receptor A	Protein Coding	40	GC05P176813	0.488359
PELO	Pelota MRNA Surveillance And Ribosome Rescue Factor	Protein Coding	40	GC05P052787	0.488359
TBCB	Tubulin Folding Cofactor B	Protein Coding	40	GC19P040382	0.464231
KLB	Klotho Beta	Protein Coding	40	GC04P039408	0.446616
EIF5A2	Eukaryotic Translation Initiation Factor 5A2	Protein Coding	40	GC03M170888	0.410437
FKBP3	FKBP Prolyl Isomerase 3	Protein Coding	40	GC14M045115	0.395524
BTN1A1	Butyrophilin Subfamily 1 Member A1	Protein Coding	40	GC06P026500	0.387482
VRK3	VRK Serine/Threonine Kinase 3	Protein Coding	40	GC19M049976	0.383915
LILRA2	Leukocyte Immunoglobulin Like Receptor A2	Protein Coding	40	GC19P054572	0.359139
USP45	Ubiquitin Specific Peptidase 45	Protein Coding	40	GC06M099432	0.353503
IL22RA2	Interleukin 22 Receptor Subunit Alpha 2	Protein Coding	40	GC06M137143	0.298894
RNF138	Ring Finger Protein 138	Protein Coding	40	GC18P032091	0.272315
RAB27B	RAB27B, Member RAS Oncogene Family	Protein Coding	40	GC18P054717	0.16595
DYNC2H1	Dynein Cytoplasmic 2 Heavy Chain 1	Protein Coding	39	GC11P103109	19.45315

NPHP3	Nephrocystin 3	Protein Coding	39	GC03M132683	15.58402
NOS1AP	Nitric Oxide Synthase 1 Adaptor Protein	Protein Coding	39	GC01P162069	14.53748
RPGRIP1L	RPGRIP1 Like	Protein Coding	39	GC16M053597	13.9985
MYOZ2	Myozenin 2	Protein Coding	39	GC04P119135	13.97896
LMOD3	Leiomodin 3	Protein Coding	39	GC03M069156	11.9347
KANSL1	KAT8 Regulatory NSL Complex Subunit 1	Protein Coding	39	GC17M046031	11.62278
TMEM126B	Transmembrane Protein 126B	Protein Coding	39	GC11P085628	10.91448
CELA2A	Chymotrypsin Like Elastase 2A	Protein Coding	39	GC01P015456	10.81616
A2ML1	Alpha-2-Macroglobulin Like 1	Protein Coding	39	GC12P008822	10.74026
DCHS1	Dachsous Cadherin-Related 1	Protein Coding	39	GC11M006621	10.6658
DNASE1L1	Deoxyribonuclease 1 Like 1	Protein Coding	39	GC0XM154401	10.5163
DISP1	Dispatched RND Transporter Family Member 1	Protein Coding	39	GC01P222814	10.34233
BBS9	Bardet-Biedl Syndrome 9	Protein Coding	39	GC07P033112	10.20506
DDX3Y	DEAD-Box Helicase 3 Y-Linked	Protein Coding	39	GC0YP012903	9.864908
BMP10	Bone Morphogenetic Protein 10	Protein Coding	39	GC02M068865	9.804852
TCTN2	Tectonic Family Member 2	Protein Coding	39	GC12P123671	9.431955
PEX16	Peroxisomal Biogenesis Factor 16	Protein Coding	39	GC11M068965	9.247545
YY1AP1	YY1 Associated Protein 1	Protein Coding	39	GC01M155659	9.223948
MYL7	Myosin Light Chain 7	Protein Coding	39	GC07M044138	9.016805
TTC37	Tetratricopeptide Repeat Domain 37	Protein Coding	39	GC05M095463	8.975733
CSPP1	Centrosome And Spindle Pole Associated Protein 1	Protein Coding	39	GC08P067055	8.929738
FBXL4	F-Box And Leucine Rich Repeat Protein 4	Protein Coding	39	GC06M098868	8.544086
BBS5	Bardet-Biedl Syndrome 5	Protein Coding	39	GC02P169480	8.408084
RNLS	Renalase, FAD Dependent Amine Oxidase	Protein Coding	39	GC10M088180	8.204286
NSMCE2	NSE2 (MMS21) Homolog, SMC5-SMC6 Complex SUMO Ligase	Protein Coding	39	GC08P125091	8.058097
COG7	Component Of Oligomeric Golgi Complex 7	Protein Coding	39	GC16M023388	7.949915
ALG12	ALG12 Alpha-1,6-Mannosyltransferase	Protein Coding	39	GC22M049859	7.929358
TRAF3IP1	TRAF3 Interacting Protein 1	Protein Coding	39	GC02P238320	7.851499
COQ4	Coenzyme Q4	Protein Coding	39	GC09P128322	7.792397
MYH15	Myosin Heavy Chain 15	Protein Coding	39	GC03M108380	7.745793
FUZ	Fuzzy Planar Cell Polarity Protein	Protein Coding	39	GC19M049806	7.740796
SYCP2	Synaptonemal Complex Protein 2	Protein Coding	39	GC20M059863	7.527241
OSTM1	Osteoclastogenesis Associated Transmembrane Protein 1	Protein Coding	39	GC06M108041	7.516975
JAZF1	JAZF Zinc Finger 1	Protein Coding	39	GC07M027830	7.492374
BTNL2	Butyrophilin Like 2	Protein Coding	39	GC06M032393	7.420308
GAS8	Growth Arrest Specific 8	Protein Coding	39	GC16P090019	7.412274
DOLK	Dolichol Kinase	Protein Coding	39	GC09M128945	7.359861
LMBRD1	LMBR1 Domain Containing 1	Protein Coding	39	GC06M069675	7.159367
IRX1	Iroquois Homeobox 1	Protein Coding	39	GC05P003596	6.92318
KMT2B	Lysine Methyltransferase 2B	Protein Coding	39	GC19P040361	6.649711
MRPS7	Mitochondrial Ribosomal Protein S7	Protein Coding	39	GC17P075262	6.499405
TBX22	T-Box Transcription Factor 22	Protein Coding	39	GC0XP080014	6.457026
MIB2	MIB E3 Ubiquitin Protein Ligase 2	Protein Coding	39	GC01P002057	6.007911
ITLN1	Intelectin 1	Protein Coding	39	GC01M160876	5.928362
CEP41	Centrosomal Protein 41	Protein Coding	39	GC07M130393	5.869094
P3H1	Prolyl 3-Hydroxylase 1	Protein Coding	39	GC01M042746	5.789468
ZP2	Zona Pellucida Glycoprotein 2	Protein Coding	39	GC16M021198	5.763949
TRMT5	TRNA Methyltransferase 5	Protein Coding	39	GC14M060971	5.757029
BRWD1	Bromodomain And WD Repeat Domain Containing 1	Protein Coding	39	GC21M039184	5.74672
XK	X-Linked Kx Blood Group	Protein Coding	39	GC0XP037685	5.608984
ZNF711	Zinc Finger Protein 711	Protein Coding	39	GC0XP085243	5.560129
LPGAT1	Lysophosphatidylglycerol Acyltransferase 1	Protein Coding	39	GC01M211743	5.491753
MYO18B	Myosin XVIIIB	Protein Coding	39	GC22P025742	5.440562

SLC52A3	Solute Carrier Family 52 Member 3	Protein Coding	39	GC20M000741	5.426104
NCAPG2	Non-SMC Condensin II Complex Subunit G2	Protein Coding	39	GC07M158631	5.253162
LEMD2	LEM Domain Nuclear Envelope Protein 2	Protein Coding	39	GC06M047009	5.232141
CDK5RAP3	CDK5 Regulatory Subunit Associated Protein 3	Protein Coding	39	GC17P047967	5.227571
EPB42	Erythrocyte Membrane Protein Band 4.2	Protein Coding	39	GC15M043271	5.158671
MYBPC2	Myosin Binding Protein C2	Protein Coding	39	GC19P050432	5.122405
SLC25A46	Solute Carrier Family 25 Member 46	Protein Coding	39	GC05P110738	5.104004
MICA	MHC Class I Polypeptide-Related Sequence A	Protein Coding	39	GC06P031399	5.060754
ANKRD26	Ankyrin Repeat Domain 26	Protein Coding	39	GC10M026938	4.974692
SMG7	SMG7 Nonsense Mediated MRNA Decay Factor	Protein Coding	39	GC01P183441	4.889922
RGS3	Regulator Of G Protein Signaling 3	Protein Coding	39	GC09P115745	4.860352
GATC	Glutamyl-TRNA Amidotransferase Subunit C	Protein Coding	39	GC12P120446	4.82232
KLF12	Kruppel Like Factor 12	Protein Coding	39	GC13M073686	4.716016
PXK	PX Domain Containing Serine/Threonine Kinase Like	Protein Coding	39	GC03P058333	4.68779
ANKLE2	Ankyrin Repeat And LEM Domain Containing 2	Protein Coding	39	GC12M132725	4.640705
NCOA6	Nuclear Receptor Coactivator 6	Protein Coding	39	GC20M034700	4.630771
ICOSLG	Inducible T Cell Costimulator Ligand	Protein Coding	39	GC21M044222	4.617815
TMEM165	Transmembrane Protein 165	Protein Coding	39	GC04P055395	4.577602
SHOX	Short Stature Homeobox	Protein Coding	39	GC0XP000624	4.575703
MUC5AC	Mucin 5AC, Oligomeric Mucus/Gel-Forming	Protein Coding	39	GC11P001151	4.552536
TRAPPC9	Trafficking Protein Particle Complex Subunit 9	Protein Coding	39	GC08M139728	4.508318
GTPBP4	GTP Binding Protein 4	Protein Coding	39	GC10P000988	4.508209
TIMM50	Translocase Of Inner Mitochondrial Membrane 50	Protein Coding	39	GC19P039480	4.474106
PIGP	Phosphatidylinositol Glycan Anchor Biosynthesis Class P	Protein Coding	39	GC21M037059	4.340335
RAB4B	RAB4B, Member RAS Oncogene Family	Protein Coding	39	GC19P041092	4.29195
ARMC9	Armadillo Repeat Containing 9	Protein Coding	39	GC02P231198	4.2658
SLC44A3	Solute Carrier Family 44 Member 3	Protein Coding	39	GC01P094820	4.241322
CXCL9	C-X-C Motif Chemokine Ligand 9	Protein Coding	39	GC04M076001	4.2304
HEPH	Hephaestin	Protein Coding	39	GC0XP066162	4.220811
HNRNPH2	Heterogeneous Nuclear Ribonucleoprotein H2	Protein Coding	39	GC0XP101408	4.148983
CELF1	CUGBP Elav-Like Family Member 1	Protein Coding	39	GC11M069003	4.14471
GPR101	G Protein-Coupled Receptor 101	Protein Coding	39	GC0XM137030	4.132441
MYO9A	Myosin IXA	Protein Coding	39	GC15M071822	4.102281
MAML3	Mastermind Like Transcriptional Coactivator 3	Protein Coding	39	GC04M139716	4.080301
SLC10A7	Solute Carrier Family 10 Member 7	Protein Coding	39	GC04M146253	4.074672
SMTN	Smoothelin	Protein Coding	39	GC22P031143	4.067614
PRDM9	PR/SET Domain 9	Protein Coding	39	GC05P023443	4.065515
FMN1	Formin 1	Protein Coding	39	GC15M032765	3.943724
COG5	Component Of Oligomeric Golgi Complex 5	Protein Coding	39	GC07M107201	3.907556
MSTO1	Misato Mitochondrial Distribution And Morphology Regulator 1	Protein Coding	39	GC01P155610	3.807073
CEP152	Centrosomal Protein 152	Protein Coding	39	GC15M048663	3.779709
MGAT4C	MGAT4 Family Member C	Protein Coding	39	GC12M085955	3.681388
RPL3L	Ribosomal Protein L3 Like	Protein Coding	39	GC16M001943	3.657843
JPH1	Junctophilin 1	Protein Coding	39	GC08M074234	3.603852
SUN1	Sad1 And UNC84 Domain Containing 1	Protein Coding	39	GC07P000815	3.581265
ISCA2	Iron-Sulfur Cluster Assembly 2	Protein Coding	39	GC14P074493	3.480239
WDR4	WD Repeat Domain 4	Protein Coding	39	GC21M042843	3.446253
PFDN1	Prefoldin Subunit 1	Protein Coding	39	GC05M140263	3.385828
STOX1	Storkhead Box 1	Protein Coding	39	GC10P068827	3.368931
ATP6V1G2	ATPase H+ Transporting V1 Subunit G2	Protein Coding	39	GC06M046877	3.35479
DYM	Dymeclin	Protein Coding	39	GC18M049041	3.354494
POFUT2	Protein O-Fucosyltransferase 2	Protein Coding	39	GC21M045263	3.343692
PLCL1	Phospholipase C Like 1 (Inactive)	Protein Coding	39	GC02P197804	3.338995

PACRG	Parkin Coregulated	Protein Coding	39	GC06P162727	3.330239
TBCE	Tubulin Folding Cofactor E	Protein Coding	39	GC01P235357	3.329387
PPP1R3C	Protein Phosphatase 1 Regulatory Subunit 3C	Protein Coding	39	GC10M091628	3.326877
SLC25A37	Solute Carrier Family 25 Member 37	Protein Coding	39	GC08P023528	3.300772
MFSD2A	Major Facilitator Superfamily Domain Containing 2A	Protein Coding	39	GC01P039955	3.295256
EXOSC10	Exosome Component 10	Protein Coding	39	GC01M011067	3.28543
MUL1	Mitochondrial E3 Ubiquitin Protein Ligase 1	Protein Coding	39	GC01M020499	3.233669
C1D	C1D Nuclear Receptor Corepressor	Protein Coding	39	GC02M068041	3.228115
EHD4	EH Domain Containing 4	Protein Coding	39	GC15M041895	3.220416
TUBGCP2	Tubulin Gamma Complex Associated Protein 2	Protein Coding	39	GC10M133278	3.219736
PPP1R10	Protein Phosphatase 1 Regulatory Subunit 10	Protein Coding	39	GC06M030600	3.194847
GRHL3	Grainyhead Like Transcription Factor 3	Protein Coding	39	GC01P024319	3.114818
SMNDC1	Survival Motor Neuron Domain Containing 1	Protein Coding	39	GC10M110290	3.110372
TSEN34	TRNA Splicing Endonuclease Subunit 34	Protein Coding	39	GC19P056807	3.103792
DUSP19	Dual Specificity Phosphatase 19	Protein Coding	39	GC02P183078	3.096402
CNNM4	Cyclin And CBS Domain Divalent Metal Cation Transport Mediator 4	Protein Coding	39	GC02P096790	3.079824
ARNTL2	Aryl Hydrocarbon Receptor Nuclear Translocator Like 2	Protein Coding	39	GC12P027332	3.076787
KANK2	KN Motif And Ankyrin Repeat Domains 2	Protein Coding	39	GC19M011165	3.04951
FIP1L1	Factor Interacting With PAPOLA And CPSF1	Protein Coding	39	GC04P053394	3.022671
PANK4	Pantothenate Kinase 4 (Inactive)	Protein Coding	39	GC01M002508	3.013085
AGTRAP	Angiotensin II Receptor Associated Protein	Protein Coding	39	GC01P011736	3.00404
ODF2	Outer Dense Fiber Of Sperm Tails 2	Protein Coding	39	GC09P128455	2.997128
GTPBP2	GTP Binding Protein 2	Protein Coding	39	GC06M043605	2.984523
ATF6B	Activating Transcription Factor 6 Beta	Protein Coding	39	GC06M032115	2.98062
CYGB	Cytoglobin	Protein Coding	39	GC17M076527	2.971547
GTPBP1	GTP Binding Protein 1	Protein Coding	39	GC22P038705	2.966242
ADGRG6	Adhesion G Protein-Coupled Receptor G6	Protein Coding	39	GC06P142301	2.961535
DCTN4	Dynactin Subunit 4	Protein Coding	39	GC05M150708	2.944941
DCP1A	Decapping MRNA 1A	Protein Coding	39	GC03M053301	2.925847
NIPAL4	NIPA Like Domain Containing 4	Protein Coding	39	GC05P157460	2.923358
SORCS1	Sortilin Related VPS10 Domain Containing Receptor 1	Protein Coding	39	GC10M106573	2.9179
SEMA5B	Semaphorin 5B	Protein Coding	39	GC03M122909	2.915459
SNX14	Sorting Nexin 14	Protein Coding	39	GC06M085505	2.908129
RCE1	Ras Converting CAAX Endopeptidase 1	Protein Coding	39	GC11P066842	2.904459
LHX8	LIM Homeobox 8	Protein Coding	39	GC01P075128	2.896402
TCF25	Transcription Factor 25	Protein Coding	39	GC16P089873	2.886785
KRR1	KRR1 Small Subunit Processome Component Homolog	Protein Coding	39	GC12M075490	2.87791
FOXP4	Forkhead Box P4	Protein Coding	39	GC06P055386	2.875857
RHOJ	Ras Homolog Family Member J	Protein Coding	39	GC14P063204	2.854737
LMF1	Lipase Maturation Factor 1	Protein Coding	39	GC16M000853	2.853245
TNNC2	Troponin C2, Fast Skeletal Type	Protein Coding	39	GC20M045823	2.848806
DR1	Down-Regulator Of Transcription 1	Protein Coding	39	GC01P093345	2.844356
RBM15	RNA Binding Motif Protein 15	Protein Coding	39	GC01P110338	2.818033
CXCL14	C-X-C Motif Chemokine Ligand 14	Protein Coding	39	GC05M135617	2.792351
TRAPPC2	Trafficking Protein Particle Complex Subunit 2	Protein Coding	39	GC0XM013712	2.784311
APBA1	Amyloid Beta Precursor Protein Binding Family A Member 1	Protein Coding	39	GC09M069427	2.783823
TRAPPC10	Trafficking Protein Particle Complex Subunit 10	Protein Coding	39	GC21P044012	2.753489
NCR3	Natural Cytotoxicity Triggering Receptor 3	Protein Coding	39	GC06M031588	2.74637
PDS5A	PDS5 Cohesin Associated Factor A	Protein Coding	39	GC04M039824	2.728449
PRSS23	Serine Protease 23	Protein Coding	39	GC11P086791	2.728083
GNLY	Granulysin	Protein Coding	39	GC02P085685	2.691793
LMCD1	LIM And Cysteine Rich Domains 1	Protein Coding	39	GC03P008518	2.673793
NUDT12	Nudix Hydrolase 12	Protein Coding	39	GC05M103548	2.667389

SESN2	Sestrin 2	Protein Coding	39	GC01P028270	2.648019
GRK3	G Protein-Coupled Receptor Kinase 3	Protein Coding	39	GC22P026762	2.608181
CIRBP	Cold Inducible RNA Binding Protein	Protein Coding	39	GC19P001259	2.601241
TARBP1	TAR (HIV-1) RNA Binding Protein 1	Protein Coding	39	GC01M234391	2.541542
HBD	Hemoglobin Subunit Delta	Protein Coding	39	GC11M005232	2.533011
EDC4	Enhancer Of MRNA Decapping 4	Protein Coding	39	GC16P067873	2.524558
RNF144B	Ring Finger Protein 144B	Protein Coding	39	GC06P018447	2.519588
RPL29	Ribosomal Protein L29	Protein Coding	39	GC03M052012	2.518294
RECQL5	RecQ Like Helicase 5	Protein Coding	39	GC17M075626	2.490489
BCL2L13	BCL2 Like 13	Protein Coding	39	GC22P017628	2.488899
ARL2BP	ADP Ribosylation Factor Like GTPase 2 Binding Protein	Protein Coding	39	GC16P057245	2.48722
PFDN4	Prefoldin Subunit 4	Protein Coding	39	GC20P054207	2.483368
MPP2	Membrane Palmitoylated Protein 2	Protein Coding	39	GC17M043875	2.479499
NACA	Nascent Polypeptide Associated Complex Subunit Alpha	Protein Coding	39	GC12M056712	2.4528
ZNF23	Zinc Finger Protein 23	Protein Coding	39	GC16M071463	2.451607
AMBRA1	Autophagy And Beclin 1 Regulator 1	Protein Coding	39	GC11M068969	2.439404
KHDRBS3	KH RNA Binding Domain Containing, Signal Transduction Associated 3	Protein Coding	39	GC08P135457	2.418975
WIPI1	WD Repeat Domain, Phosphoinositide Interacting 1	Protein Coding	39	GC17M068420	2.417856
PIGS	Phosphatidylinositol Glycan Anchor Biosynthesis Class S	Protein Coding	39	GC17M028553	2.39283
NECAP1	NECAP Endocytosis Associated 1	Protein Coding	39	GC12P011900	2.388321
FIS1	Fission, Mitochondrial 1	Protein Coding	39	GC07M101239	2.386796
SYNCRIP	Synaptotagmin Binding Cytoplasmic RNA Interacting Protein	Protein Coding	39	GC06M085607	2.382025
SLC30A7	Solute Carrier Family 30 Member 7	Protein Coding	39	GC01P100896	2.345431
SCARF1	Scavenger Receptor Class F Member 1	Protein Coding	39	GC17M001668	2.344947
DDHD1	DDHD Domain Containing 1	Protein Coding	39	GC14M053036	2.342251
ACAD10	Acyl-CoA Dehydrogenase Family Member 10	Protein Coding	39	GC12P111686	2.342232
KIF17	Kinesin Family Member 17	Protein Coding	39	GC01M020663	2.34108
GNG11	G Protein Subunit Gamma 11	Protein Coding	39	GC07P093921	2.33143
WLS	Wnt Ligand Secretion Mediator	Protein Coding	39	GC01M068098	2.324736
UBD	Ubiquitin D	Protein Coding	39	GC06M046741	2.322406
CTNNAL1	Catenin Alpha Like 1	Protein Coding	39	GC09M108942	2.316295
RBM3	RNA Binding Motif Protein 3	Protein Coding	39	GC0XP048574	2.313959
TRIM36	Tripartite Motif Containing 36	Protein Coding	39	GC05M115124	2.297405
CAND1	Cullin Associated And Neddylation Dissociated 1	Protein Coding	39	GC12P067270	2.292226
IK	IK Cytokine	Protein Coding	39	GC05P143780	2.286345
TSR1	TSR1 Ribosome Maturation Factor	Protein Coding	39	GC17M002322	2.285948
OGDHL	Oxoglutarate Dehydrogenase L	Protein Coding	39	GC10M049734	2.270671
ASPM	Assembly Factor For Spindle Microtubules	Protein Coding	39	GC01M197084	2.268691
PRPH2	Peripherin 2	Protein Coding	39	GC06M047116	2.259704
CEP135	Centrosomal Protein 135	Protein Coding	39	GC04P055948	2.248215
AGBL5	AGBL Carboxypeptidase 5	Protein Coding	39	GC02P027044	2.242949
TOM1L2	Target Of Myb1 Like 2 Membrane Trafficking Protein	Protein Coding	39	GC17M017843	2.217477
SPTBN4	Spectrin Beta, Non-Erythrocytic 4	Protein Coding	39	GC19P040466	2.21469
CD180	CD180 Molecule	Protein Coding	39	GC05M067181	2.206737
CACNG7	Calcium Voltage-Gated Channel Auxiliary Subunit Gamma 7	Protein Coding	39	GC19P053909	2.200811
RALY	RALY Heterogeneous Nuclear Ribonucleoprotein	Protein Coding	39	GC20P033993	2.188338
LPO	Lactoperoxidase	Protein Coding	39	GC17P058218	2.183235
SUMO4	Small Ubiquitin Like Modifier 4	Protein Coding	39	GC06P149401	2.182927
GALNTL5	Polypeptide N-Acetylgalactosaminyltransferase Like 5	Protein Coding	39	GC07P151956	2.182126
ADGRG1	Adhesion G Protein-Coupled Receptor G1	Protein Coding	39	GC16P057610	2.157975
NIPA1	NIPA Magnesium Transporter 1	Protein Coding	39	GC15P022773	2.155158
PEG3	Paternally Expressed 3	Protein Coding	39	GC19M056810	2.152716
FBLIM1	Filamin Binding LIM Protein 1	Protein Coding	39	GC01P015756	2.137318

PDIA2	Protein Disulfide Isomerase Family A Member 2	Protein Coding	39	GC16P005498	2.13026
SRPX	Sushi Repeat Containing Protein X-Linked	Protein Coding	39	GC0XM038149	2.1192
YTHDF2	YTH N6-Methyladenosine RNA Binding Protein 2	Protein Coding	39	GC01P028751	2.097837
CLINT1	Clathrin Interactor 1	Protein Coding	39	GC05M157785	2.084717
SUB1	SUB1 Regulator Of Transcription	Protein Coding	39	GC05P032540	2.081121
NYX	Nyctalopin	Protein Coding	39	GC0XP041447	2.067596
SERPINA10	Serpin Family A Member 10	Protein Coding	39	GC14M094280	2.062516
TXNDC5	Thioredoxin Domain Containing 5	Protein Coding	39	GC06M007893	2.054112
MRPS2	Mitochondrial Ribosomal Protein S2	Protein Coding	39	GC09P135499	2.05226
IFIT3	Interferon Induced Protein With Tetratricopeptide Repeats 3	Protein Coding	39	GC10P089327	2.052006
CTNNBL1	Catenin Beta Like 1	Protein Coding	39	GC20P037693	2.041428
ZHX2	Zinc Fingers And Homeoboxes 2	Protein Coding	39	GC08P122781	1.995812
RAD51B	RAD51 Paralog B	Protein Coding	39	GC14P067819	1.989627
CDR2	Cerebellar Degeneration Related Protein 2	Protein Coding	39	GC16M022357	1.973051
PRICKLE2	Prickle Planar Cell Polarity Protein 2	Protein Coding	39	GC03M064079	1.965639
ILF3	Interleukin Enhancer Binding Factor 3	Protein Coding	39	GC19P010625	1.956926
PHF20	PHD Finger Protein 20	Protein Coding	39	GC20P035771	1.938784
ZDHHC2	Zinc Finger DHHC-Type Palmitoyltransferase 2	Protein Coding	39	GC08P017156	1.93615
RND1	Rho Family GTPase 1	Protein Coding	39	GC12M048857	1.934883
LDB2	LIM Domain Binding 2	Protein Coding	39	GC04M016445	1.931167
MRPS25	Mitochondrial Ribosomal Protein S25	Protein Coding	39	GC03M016929	1.907322
PLXNB2	Plexin B2	Protein Coding	39	GC22M050274	1.906557
LRSAM1	Leucine Rich Repeat And Sterile Alpha Motif Containing 1	Protein Coding	39	GC09P127451	1.903893
NETO2	Neuropilin And Tolloid Like 2	Protein Coding	39	GC16M047077	1.889511
KCTD7	Potassium Channel Tetramerization Domain Containing 7	Protein Coding	39	GC07P066628	1.88715
SLC30A3	Solute Carrier Family 30 Member 3	Protein Coding	39	GC02M027254	1.874229
RSL1D1	Ribosomal L1 Domain Containing 1	Protein Coding	39	GC16M011833	1.861578
DNAJC7	DnaJ Heat Shock Protein Family (Hsp40) Member C7	Protein Coding	39	GC17M041977	1.861151
CRYBB1	Crystallin Beta B1	Protein Coding	39	GC22M026599	1.859906
WDR12	WD Repeat Domain 12	Protein Coding	39	GC02M202874	1.858005
MYO1B	Myosin IB	Protein Coding	39	GC02P191246	1.857528
AFAP1	Actin Filament Associated Protein 1	Protein Coding	39	GC04M007758	1.856341
DSC1	Desmocollin 1	Protein Coding	39	GC18M031129	1.855419
BMS1	BMS1 Ribosome Biogenesis Factor	Protein Coding	39	GC10P042782	1.853551
ACER3	Alkaline Ceramidase 3	Protein Coding	39	GC11P076860	1.841442
SIM2	SIM BHLH Transcription Factor 2	Protein Coding	39	GC21P036699	1.841012
GOLM1	Golgi Membrane Protein 1	Protein Coding	39	GC09M086026	1.836466
EN1	Engrailed Homeobox 1	Protein Coding	39	GC02M118842	1.832048
IKZF2	IKAROS Family Zinc Finger 2	Protein Coding	39	GC02M213001	1.822936
MRRF	Mitochondrial Ribosome Recycling Factor	Protein Coding	39	GC09P122264	1.814784
TRIM38	Tripartite Motif Containing 38	Protein Coding	39	GC06P025962	1.810159
PSMC3IP	PSMC3 Interacting Protein	Protein Coding	39	GC17M042572	1.796631
GJA10	Gap Junction Protein Alpha 10	Protein Coding	39	GC06P089894	1.783567
TECTA	Tectorin Alpha	Protein Coding	39	GC11P121101	1.77862
CSDE1	Cold Shock Domain Containing E1	Protein Coding	39	GC01M114716	1.771321
PPP1R7	Protein Phosphatase 1 Regulatory Subunit 7	Protein Coding	39	GC02P241150	1.756783
ITM2A	Integral Membrane Protein 2A	Protein Coding	39	GC0XM079360	1.75611
SLC15A4	Solute Carrier Family 15 Member 4	Protein Coding	39	GC12M128793	1.731609
SF1	Splicing Factor 1	Protein Coding	39	GC11M064764	1.715473
ITGB1BP1	Integrin Subunit Beta 1 Binding Protein 1	Protein Coding	39	GC02M009391	1.715325
AOAH	Acyloxyacyl Hydrolase	Protein Coding	39	GC07M036519	1.711787
YBX2	Y-Box Binding Protein 2	Protein Coding	39	GC17M007288	1.709589
CISD1	CDGSH Iron Sulfur Domain 1	Protein Coding	39	GC10P058269	1.708729

CD177	CD177 Molecule	Protein Coding	39	GC19P043353	1.704977
MAB21L2	Mab-21 Like 2	Protein Coding	39	GC04P150581	1.700228
UBR4	Ubiquitin Protein Ligase E3 Component N-Recognin 4	Protein Coding	39	GC01M019074	1.697535
SLC28A3	Solute Carrier Family 28 Member 3	Protein Coding	39	GC09M086096	1.696076
VSX1	Visual System Homeobox 1	Protein Coding	39	GC20M025070	1.691939
LY9	Lymphocyte Antigen 9	Protein Coding	39	GC01P160796	1.681278
MTMR1	Myotubularin Related Protein 1	Protein Coding	39	GC0XP150692	1.679982
MGAT4A	Alpha-1,3-Mannosyl-Glycoprotein 4-Beta-N-Acetylglucosaminyltransferase A	Protein Coding	39	GC02M098619	1.668357
SLC25A38	Solute Carrier Family 25 Member 38	Protein Coding	39	GC03P039405	1.662079
PLA2G4B	Phospholipase A2 Group IVB	Protein Coding	39	GC15P041837	1.659388
AFF1	AF4/FMR2 Family Member 1	Protein Coding	39	GC04P086934	1.658303
CCL8	C-C Motif Chemokine Ligand 8	Protein Coding	39	GC17P034319	1.658257
MPP3	Membrane Palmitoylated Protein 3	Protein Coding	39	GC17M043800	1.656898
HM13	Histocompatibility Minor 13	Protein Coding	39	GC20P031514	1.655299
CWF19L1	CWF19 Like Cell Cycle Control Factor 1	Protein Coding	39	GC10M100232	1.648197
CRLF2	Cytokine Receptor Like Factor 2	Protein Coding	39	GC0XM001190	1.643107
TOMM34	Translocase Of Outer Mitochondrial Membrane 34	Protein Coding	39	GC20M044942	1.642307
TLX3	T Cell Leukemia Homeobox 3	Protein Coding	39	GC05P171309	1.62461
TMEM106B	Transmembrane Protein 106B	Protein Coding	39	GC07P012218	1.623959
MAEA	Macrophage Erythroblast Attacher, E3 Ubiquitin Ligase	Protein Coding	39	GC04P001289	1.613736
TLL2	Tolloid Like 2	Protein Coding	39	GC10M096364	1.613047
SH2D3C	SH2 Domain Containing 3C	Protein Coding	39	GC09M127738	1.609179
USO1	USO1 Vesicle Transport Factor	Protein Coding	39	GC04P075724	1.608408
SLC30A6	Solute Carrier Family 30 Member 6	Protein Coding	39	GC02P032166	1.602112
ADGRE5	Adhesion G Protein-Coupled Receptor E5	Protein Coding	39	GC19P014381	1.581856
IL18RAP	Interleukin 18 Receptor Accessory Protein	Protein Coding	39	GC02P102418	1.580253
AHCYL2	Adenosylhomocysteinase Like 2	Protein Coding	39	GC07P129225	1.579494
TRIM44	Tripartite Motif Containing 44	Protein Coding	39	GC11P035684	1.573789
NKX6-2	NK6 Homeobox 2	Protein Coding	39	GC10M132783	1.573056
NMU	Neuromedin U	Protein Coding	39	GC04M055595	1.565892
HEXIM1	HEXIM P-TEFb Complex Subunit 1	Protein Coding	39	GC17P045147	1.559278
NOP58	NOP58 Ribonucleoprotein	Protein Coding	39	GC02P202265	1.551844
GABRE	Gamma-Aminobutyric Acid Type A Receptor Subunit Epsilon	Protein Coding	39	GC0XM151955	1.539337
CD48	CD48 Molecule	Protein Coding	39	GC01M160648	1.537692
RFNG	RFNG O-Fucosylpeptide 3-Beta-N-Acetylglucosaminyltransferase	Protein Coding	39	GC17M082047	1.531082
ING5	Inhibitor Of Growth Family Member 5	Protein Coding	39	GC02P241702	1.513069
TNFAIP2	TNF Alpha Induced Protein 2	Protein Coding	39	GC14P106039	1.50741
ZNF79	Zinc Finger Protein 79	Protein Coding	39	GC09P127424	1.497869
NUCB2	Nucleobindin 2	Protein Coding	39	GC11P017237	1.48987
GATAD2A	GATA Zinc Finger Domain Containing 2A	Protein Coding	39	GC19P026663	1.48095
DDX24	DEAD-Box Helicase 24	Protein Coding	39	GC14M094048	1.479256
DNASE2	Deoxyribonuclease 2, Lysosomal	Protein Coding	39	GC19M012875	1.477751
RNF10	Ring Finger Protein 10	Protein Coding	39	GC12P120543	1.475986
MRPS23	Mitochondrial Ribosomal Protein S23	Protein Coding	39	GC17M057834	1.472048
ATP6V1G1	ATPase H+ Transporting V1 Subunit G1	Protein Coding	39	GC09P115756	1.469687
CNOT6	CCR4-NOT Transcription Complex Subunit 6	Protein Coding	39	GC05P180494	1.46935
LAPTM5	Lysosomal Protein Transmembrane 5	Protein Coding	39	GC01M030732	1.463652
SLC25A42	Solute Carrier Family 25 Member 42	Protein Coding	39	GC19P019063	1.459955
CLSTN2	Calsyntenin 2	Protein Coding	39	GC03P139935	1.457369
TPCN2	Two Pore Segment Channel 2	Protein Coding	39	GC11P069066	1.452257
PSMD5	Proteasome 26S Subunit, Non-ATPase 5	Protein Coding	39	GC09M120815	1.450596
ZNF638	Zinc Finger Protein 638	Protein Coding	39	GC02P071276	1.449377
CRELD2	Cysteine Rich With EGF Like Domains 2	Protein Coding	39	GC22P049918	1.443032

ASMT	Acetylserotonin O-Methyltransferase	Protein Coding	39	GC0XP001595	1.438909
NRGN	Neurogranin	Protein Coding	39	GC11P124739	1.433658
SRP14	Signal Recognition Particle 14	Protein Coding	39	GC15M040035	1.433008
GCM1	Glial Cells Missing Transcription Factor 1	Protein Coding	39	GC06M053099	1.430463
CREB3	CAMP Responsive Element Binding Protein 3	Protein Coding	39	GC09P035960	1.429114
RIOK3	RIO Kinase 3	Protein Coding	39	GC18P023452	1.425996
KCNU1	Potassium Calcium-Activated Channel Subfamily U Member 1	Protein Coding	39	GC08P036784	1.420831
ZBP1	Z-DNA Binding Protein 1	Protein Coding	39	GC20M057603	1.414341
KIFC3	Kinesin Family Member C3	Protein Coding	39	GC16M057758	1.413008
PCDH10	Protocadherin 10	Protein Coding	39	GC04P133149	1.4087
GBP3	Guanylate Binding Protein 3	Protein Coding	39	GC01M089006	1.395136
TMX1	Thioredoxin Related Transmembrane Protein 1	Protein Coding	39	GC14P051240	1.389667
GLDN	Gliomedin	Protein Coding	39	GC15P051341	1.389341
NOLC1	Nucleolar And Coiled-Body Phosphoprotein 1	Protein Coding	39	GC10P102152	1.387184
PDE7B	Phosphodiesterase 7B	Protein Coding	39	GC06P135795	1.373025
POU4F1	POU Class 4 Homeobox 1	Protein Coding	39	GC13M078598	1.371869
MGAT5B	Alpha-1,6-Mannosylglycoprotein 6-Beta-N-Acetylglucosaminyltransferase B	Protein Coding	39	GC17P076868	1.368579
NAP1L1	Nucleosome Assembly Protein 1 Like 1	Protein Coding	39	GC12M076036	1.361875
EIF3L	Eukaryotic Translation Initiation Factor 3 Subunit L	Protein Coding	39	GC22P037848	1.360994
EXOSC5	Exosome Component 5	Protein Coding	39	GC19M046803	1.35379
GEMIN4	Gem Nuclear Organelle Associated Protein 4	Protein Coding	39	GC17M000744	1.351246
GADD45B	Growth Arrest And DNA Damage Inducible Beta	Protein Coding	39	GC19P002476	1.346093
XPO7	Exportin 7	Protein Coding	39	GC08P021919	1.343962
TAF7	TATA-Box Binding Protein Associated Factor 7	Protein Coding	39	GC05M141260	1.341469
RGS1	Regulator Of G Protein Signaling 1	Protein Coding	39	GC01P192575	1.328942
TUBA3E	Tubulin Alpha 3e	Protein Coding	39	GC02M130191	1.322857
USP47	Ubiquitin Specific Peptidase 47	Protein Coding	39	GC11P011819	1.314325
ZFAND5	Zinc Finger AN1-Type Containing 5	Protein Coding	39	GC09M072351	1.310489
RPS21	Ribosomal Protein S21	Protein Coding	39	GC20P062387	1.308842
MX2	MX Dynamin Like GTPase 2	Protein Coding	39	GC21P041361	1.305664
SMOX	Spermine Oxidase	Protein Coding	39	GC20P004120	1.294565
ACSM5	Acyl-CoA Synthetase Medium Chain Family Member 5	Protein Coding	39	GC16P020410	1.290009
CD53	CD53 Molecule	Protein Coding	39	GC01P110871	1.287994
COL19A1	Collagen Type XIX Alpha 1 Chain	Protein Coding	39	GC06P069866	1.284639
UGT1A7	UDP Glucuronosyltransferase Family 1 Member A7	Protein Coding	39	GC02P233681	1.276484
CD248	CD248 Molecule	Protein Coding	39	GC11M066314	1.275967
DOCK10	Dedicator Of Cytokinesis 10	Protein Coding	39	GC02M224765	1.264538
PAPOLG	Poly(A) Polymerase Gamma	Protein Coding	39	GC02P060756	1.26337
WDR36	WD Repeat Domain 36	Protein Coding	39	GC05P111091	1.261714
SRGN	Serglycin	Protein Coding	39	GC10P069088	1.259359
PLCH1	Phospholipase C Eta 1	Protein Coding	39	GC03M155381	1.257685
KLHL12	Kelch Like Family Member 12	Protein Coding	39	GC01M202891	1.256381
DNER	Delta/Notch Like EGF Repeat Containing	Protein Coding	39	GC02M229357	1.253812
PCBP4	Poly(RC) Binding Protein 4	Protein Coding	39	GC03M051957	1.248168
NELL1	Neural EGFL Like 1	Protein Coding	39	GC11P020647	1.243157
BAIAP3	BAI1 Associated Protein 3	Protein Coding	39	GC16P001333	1.242297
ACBD3	Acyl-CoA Binding Domain Containing 3	Protein Coding	39	GC01M226144	1.242161
NFE2L3	Nuclear Factor, Erythroid 2 Like 3	Protein Coding	39	GC07P026152	1.233859
PATZ1	POZ/BTB And AT Hook Containing Zinc Finger 1	Protein Coding	39	GC22M031325	1.225333
PSPN	Persephin	Protein Coding	39	GC19M006375	1.215333
ACOT12	Acyl-CoA Thioesterase 12	Protein Coding	39	GC05M081309	1.213456
FXYD6	FXYD Domain Containing Ion Transport Regulator 6	Protein Coding	39	GC11M117836	1.208196
MATN2	Matrilin 2	Protein Coding	39	GC08P097868	1.204226

CRYGB	Crystallin Gamma B	Protein Coding	39	GC02M208142	1.203976
GPA33	Glycoprotein A33	Protein Coding	39	GC01M167052	1.203174
ACCS	1-Aminocyclopropane-1-Carboxylate Synthase Homolog (Inactive)	Protein Coding	39	GC11P044045	1.202803
PPME1	Protein Phosphatase Methylesterase 1	Protein Coding	39	GC11P074170	1.199836
KLHL1	Kelch Like Family Member 1	Protein Coding	39	GC13M069700	1.188473
SLC6A18	Solute Carrier Family 6 Member 18	Protein Coding	39	GC05P001225	1.185659
MEMO1	Mediator Of Cell Motility 1	Protein Coding	39	GC02M031865	1.183226
DHX58	DExH-Box Helicase 58	Protein Coding	39	GC17M042101	1.178174
DNAJB9	DnaJ Heat Shock Protein Family (Hsp40) Member B9	Protein Coding	39	GC07P108569	1.171278
LHPP	Phospholysine Phosphohistidine Inorganic Pyrophosphate Phosphatase	Protein Coding	39	GC10P124461	1.162204
PRC1	Protein Regulator Of Cytokinesis 1	Protein Coding	39	GC15M090966	1.145916
SYTL4	Synaptotagmin Like 4	Protein Coding	39	GC0XM100674	1.138371
BTLA	B And T Lymphocyte Associated	Protein Coding	39	GC03M112463	1.133328
MT1E	Metallothionein 1E	Protein Coding	39	GC16P056625	1.133235
TPBG	Trophoblast Glycoprotein	Protein Coding	39	GC06P082363	1.130235
WDR61	WD Repeat Domain 61	Protein Coding	39	GC15M078277	1.128193
LARP1	La Ribonucleoprotein 1, Translational Regulator	Protein Coding	39	GC05P154682	1.119908
ACOT8	Acyl-CoA Thioesterase 8	Protein Coding	39	GC20M045841	1.113982
UGT1A3	UDP Glucuronosyltransferase Family 1 Member A3	Protein Coding	39	GC02P233729	1.111569
SERPINI2	Serpin Family I Member 2	Protein Coding	39	GC03M167441	1.108987
NUMBL	NUMB Like Endocytic Adaptor Protein	Protein Coding	39	GC19M040665	1.106587
HNRNPAB	Heterogeneous Nuclear Ribonucleoprotein A/B	Protein Coding	39	GC05P178204	1.097777
ATP10D	ATPase Phospholipid Transporting 10D (Putative)	Protein Coding	39	GC04P047490	1.083859
STRADB	STE20 Related Adaptor Beta	Protein Coding	39	GC02P201387	1.083821
IL20RB	Interleukin 20 Receptor Subunit Beta	Protein Coding	39	GC03P136946	1.081957
BTG3	BTG Anti-Proliferation Factor 3	Protein Coding	39	GC21M017594	1.07887
UBE2W	Ubiquitin Conjugating Enzyme E2 W	Protein Coding	39	GC08M073780	1.068848
BMP8B	Bone Morphogenetic Protein 8b	Protein Coding	39	GC01M039757	1.067117
ECE2	Endothelin Converting Enzyme 2	Protein Coding	39	GC03P184276	1.065824
SRSF4	Serine And Arginine Rich Splicing Factor 4	Protein Coding	39	GC01M029147	1.052035
CXXC5	CXXC Finger Protein 5	Protein Coding	39	GC05P139647	1.048315
NIPSNAP1	Nipsnap Homolog 1	Protein Coding	39	GC22M029554	1.04732
CXCL3	C-X-C Motif Chemokine Ligand 3	Protein Coding	39	GC04M074036	1.044178
ATP6V0D2	ATPase H+ Transporting V0 Subunit D2	Protein Coding	39	GC08P085987	1.033067
KPNA5	Karyopherin Subunit Alpha 5	Protein Coding	39	GC06P116681	1.025961
SNX27	Sorting Nexin 27	Protein Coding	39	GC01P151611	1.016699
CSTF1	Cleavage Stimulation Factor Subunit 1	Protein Coding	39	GC20P056392	1.012946
MAGEA4	MAGE Family Member A4	Protein Coding	39	GC0XP151912	1.00994
OXSM	3-Oxoacyl-ACP Synthase, Mitochondrial	Protein Coding	39	GC03P025782	1.008097
TIMELESS	Timeless Circadian Regulator	Protein Coding	39	GC12M056416	1.007468
FUBP1	Far Upstream Element Binding Protein 1	Protein Coding	39	GC01M077944	0.999619
SAP30	Sin3A Associated Protein 30	Protein Coding	39	GC04P173369	0.999299
MOS	MOS Proto-Oncogene, Serine/Threonine Kinase	Protein Coding	39	GC08M056112	0.99567
DNAJC12	DnaJ Heat Shock Protein Family (Hsp40) Member C12	Protein Coding	39	GC10M067796	0.994779
FERMT2	FERM Domain Containing Kindlin 2	Protein Coding	39	GC14M052857	0.994477
BCL2L12	BCL2 Like 12	Protein Coding	39	GC19P049742	0.990298
COPS6	COP9 Signalosome Subunit 6	Protein Coding	39	GC07P100088	0.97833
HVCN1	Hydrogen Voltage Gated Channel 1	Protein Coding	39	GC12M110627	0.97226
NEUROG2	Neurogenin 2	Protein Coding	39	GC04M112513	0.972179
DPP3	Dipeptidyl Peptidase 3	Protein Coding	39	GC11P066770	0.970733
COPB1	COPI Coat Complex Subunit Beta 1	Protein Coding	39	GC11M014436	0.967891
SLC5A10	Solute Carrier Family 5 Member 10	Protein Coding	39	GC17P018950	0.964375
EIF3E	Eukaryotic Translation Initiation Factor 3 Subunit E	Protein Coding	39	GC08M108163	0.961858

KLRG1	Killer Cell Lectin Like Receptor G1	Protein Coding	39	GC12P008950	0.956401
GDF7	Growth Differentiation Factor 7	Protein Coding	39	GC02P020666	0.933115
POLDIP3	DNA Polymerase Delta Interacting Protein 3	Protein Coding	39	GC22M042583	0.918944
STOML2	Stomatin Like 2	Protein Coding	39	GC09M035099	0.907942
BFSP1	Beaded Filament Structural Protein 1	Protein Coding	39	GC20M017493	0.905077
RAB26	RAB26, Member RAS Oncogene Family	Protein Coding	39	GC16P005585	0.88713
PRLHR	Prolactin Releasing Hormone Receptor	Protein Coding	39	GC10M118590	0.879446
ZNF644	Zinc Finger Protein 644	Protein Coding	39	GC01M090915	0.877814
ZFYVE26	Zinc Finger FYVE-Type Containing 26	Protein Coding	39	GC14M067727	0.876385
L3MBTL1	L3MBTL Histone Methyl-Lysine Binding Protein 1	Protein Coding	39	GC20P043507	0.875467
REEP6	Receptor Accessory Protein 6	Protein Coding	39	GC19P001491	0.875363
DERA	Deoxyribose-Phosphate Aldolase	Protein Coding	39	GC12P015911	0.872489
LYPLA2	Lysophospholipase 2	Protein Coding	39	GC01P023790	0.872218
POLR2G	RNA Polymerase II Subunit G	Protein Coding	39	GC11P062762	0.869094
CPA5	Carboxypeptidase A5	Protein Coding	39	GC07P130344	0.863618
TAF10	TATA-Box Binding Protein Associated Factor 10	Protein Coding	39	GC11M006608	0.861531
CD101	CD101 Molecule	Protein Coding	39	GC01P117001	0.847699
PLA2G2E	Phospholipase A2 Group IIE	Protein Coding	39	GC01M019920	0.847341
ZNF24	Zinc Finger Protein 24	Protein Coding	39	GC18M035332	0.845107
APOBEC1	Apolipoprotein B MRNA Editing Enzyme Catalytic Subunit 1	Protein Coding	39	GC12M007649	0.835296
TMEM63A	Transmembrane Protein 63A	Protein Coding	39	GC01M225845	0.824284
SURF4	Surfeit 4	Protein Coding	39	GC09M133361	0.823498
SEC24A	SEC24 Homolog A, COPII Coat Complex Component	Protein Coding	39	GC05P134647	0.822145
NSFL1C	NSFL1 Cofactor	Protein Coding	39	GC20M001442	0.814794
CD5L	CD5 Molecule Like	Protein Coding	39	GC01M157800	0.788499
MIOX	Myo-Inositol Oxygenase	Protein Coding	39	GC22P050486	0.770725
DYRK4	Dual Specificity Tyrosine Phosphorylation Regulated Kinase 4	Protein Coding	39	GC12P011798	0.770128
KLHL20	Kelch Like Family Member 20	Protein Coding	39	GC01P173714	0.761095
SLC26A6	Solute Carrier Family 26 Member 6	Protein Coding	39	GC03M048625	0.759984
SIGLEC6	Sialic Acid Binding Ig Like Lectin 6	Protein Coding	39	GC19M051517	0.75791
FBXO2	F-Box Protein 2	Protein Coding	39	GC01M011637	0.757649
RAD1	RAD1 Checkpoint DNA Exonuclease	Protein Coding	39	GC05M034905	0.757081
SERPINB4	Serpin Family B Member 4	Protein Coding	39	GC18M063637	0.75335
CALY	Calcyon Neuron Specific Vesicular Protein	Protein Coding	39	GC10M133325	0.739428
LHB	Luteinizing Hormone Subunit Beta	Protein Coding	39	GC19M049015	0.735193
NCBP1	Nuclear Cap Binding Protein Subunit 1	Protein Coding	39	GC09P097633	0.73308
HNRNPH3	Heterogeneous Nuclear Ribonucleoprotein H3	Protein Coding	39	GC10P068331	0.73143
UBAP1	Ubiquitin Associated Protein 1	Protein Coding	39	GC09P034179	0.718121
FFAR4	Free Fatty Acid Receptor 4	Protein Coding	39	GC10P093566	0.70549
SIGLEC12	Sialic Acid Binding Ig Like Lectin 12	Protein Coding	39	GC19M051491	0.68056
KLC3	Kinesin Light Chain 3	Protein Coding	39	GC19P045333	0.660631
RRAGB	Ras Related GTP Binding B	Protein Coding	39	GC0XP055717	0.660405
ULBP2	UL16 Binding Protein 2	Protein Coding	39	GC06P149941	0.655336
MT1X	Metallothionein 1X	Protein Coding	39	GC16P056768	0.655336
LY75	Lymphocyte Antigen 75	Protein Coding	39	GC02M159803	0.643257
BAG4	BAG Cochaperone 4	Protein Coding	39	GC08P038176	0.641193
VPS39	VPS39 Subunit Of HOPS Complex	Protein Coding	39	GC15M042159	0.637728
OSCAR	Osteoclast Associated Ig-Like Receptor	Protein Coding	39	GC19M054094	0.63558
GOLGA5	Golgin A5	Protein Coding	39	GC14P092794	0.633189
SNRPA1	Small Nuclear Ribonucleoprotein Polypeptide A'	Protein Coding	39	GC15M101281	0.622047
FAM3B	FAM3 Metabolism Regulating Signaling Molecule B	Protein Coding	39	GC21P041304	0.606082
ANXA9	Annexin A9	Protein Coding	39	GC01P150982	0.587517
SFXN3	Sideroflexin 3	Protein Coding	39	GC10P101031	0.576147

DUSP23	Dual Specificity Phosphatase 23	Protein Coding	39	GC01P159750	0.575836
IFNW1	Interferon Omega 1	Protein Coding	39	GC09M021140	0.57415
GMIP	GEM Interacting Protein	Protein Coding	39	GC19M019629	0.570955
ACY3	Aminoacylase 3	Protein Coding	39	GC11M067642	0.565584
TUBGCP4	Tubulin Gamma Complex Associated Protein 4	Protein Coding	39	GC15P043369	0.560094
MAP1A	Microtubule Associated Protein 1A	Protein Coding	39	GC15P043516	0.535527
RBM28	RNA Binding Motif Protein 28	Protein Coding	39	GC07M128320	0.531906
PSG1	Pregnancy Specific Beta-1-Glycoprotein 1	Protein Coding	39	GC19M042866	0.516372
MYO5C	Myosin VC	Protein Coding	39	GC15M067803	0.503059
ULBP3	UL16 Binding Protein 3	Protein Coding	39	GC06M150062	0.481928
CTRB1	Chymotrypsinogen B1	Protein Coding	39	GC16P075218	0.481282
EBAG9	Estrogen Receptor Binding Site Associated Antigen 9	Protein Coding	39	GC08P109536	0.464844
RBP2	Retinol Binding Protein 2	Protein Coding	39	GC03M139452	0.462388
USH1G	USH1 Protein Network Component Sans	Protein Coding	39	GC17M074916	0.462105
CYP2A7	Cytochrome P450 Family 2 Subfamily A Member 7	Protein Coding	39	GC19M040875	0.443525
CETN1	Centrin 1	Protein Coding	39	GC18P000580	0.430008
CDCP1	CUB Domain Containing Protein 1	Protein Coding	39	GC03M045082	0.425762
PIK3AP1	Phosphoinositide-3-Kinase Adaptor Protein 1	Protein Coding	39	GC10M096593	0.425762
REG1B	Regenerating Family Member 1 Beta	Protein Coding	39	GC02M079086	0.387003
HOXD8	Homeobox D8	Protein Coding	39	GC02P176129	0.385111
CST5	Cystatin D	Protein Coding	39	GC20M023875	0.383915
CHRNA10	Cholinergic Receptor Nicotinic Alpha 10 Subunit	Protein Coding	39	GC11M003666	0.318215
GEMIN6	Gem Nuclear Organelle Associated Protein 6	Protein Coding	39	GC02P038751	0.272315
TJP3	Tight Junction Protein 3	Protein Coding	39	GC19P003708	0.266303
TMEM67	Transmembrane Protein 67	Protein Coding	38	GC08P093754	20.46205
DNAH8	Dynein Axonemal Heavy Chain 8	Protein Coding	38	GC06P055369	20.24779
EVC	EvC Ciliary Complex Subunit 1	Protein Coding	38	GC04P005712	16.69828
WDR19	WD Repeat Domain 19	Protein Coding	38	GC04P039184	16.21738
ERCC6L2	ERCC Excision Repair 6 Like 2	Protein Coding	38	GC09P095871	15.4033
GRK2	G Protein-Coupled Receptor Kinase 2	Protein Coding	38	GC11P067266	15.14786
TTC21B	Tetratricopeptide Repeat Domain 21B	Protein Coding	38	GC02M165905	14.8514
SYCE1	Synaptonemal Complex Central Element Protein 1	Protein Coding	38	GC10M133553	14.73676
CC2D2A	Coiled-Coil And C2 Domain Containing 2A	Protein Coding	38	GC04P015471	14.57573
WDR35	WD Repeat Domain 35	Protein Coding	38	GC02M019910	14.40517
HYLS1	HYLS1 Centriolar And Ciliogenesis Associated	Protein Coding	38	GC11P125883	13.72777
BBS1	Bardet-Biedl Syndrome 1	Protein Coding	38	GC11P066772	13.3727
GATAD1	GATA Zinc Finger Domain Containing 1	Protein Coding	38	GC07P092447	13.12304
MYH7B	Myosin Heavy Chain 7B	Protein Coding	38	GC20P034956	13.06754
KLHL10	Kelch Like Family Member 10	Protein Coding	38	GC17P041835	12.90763
SLX4	SLX4 Structure-Specific Endonuclease Subunit	Protein Coding	38	GC16M003785	11.97793
BBS7	Bardet-Biedl Syndrome 7	Protein Coding	38	GC04M121824	11.87844
PKHD1	PKHD1 Ciliary IPT Domain Containing Fibrocystin/Polyductin	Protein Coding	38	GC06M051588	11.22244
IRX4	Iroquois Homeobox 4	Protein Coding	38	GC05M001877	10.87553
CLIP2	CAP-Gly Domain Containing Linker Protein 2	Protein Coding	38	GC07P074289	10.85376
HGSNAT	Heparan-Alpha-Glucosaminide N-Acetyltransferase	Protein Coding	38	GC08P043140	10.82482
SLC25A26	Solute Carrier Family 25 Member 26	Protein Coding	38	GC03P066120	10.70476
ARVCF	ARVCF Delta Catenin Family Member	Protein Coding	38	GC22M019966	10.66295
FRAS1	Fraser Extracellular Matrix Complex Subunit 1	Protein Coding	38	GC04P078056	10.65862
MYOM2	Myomesin 2	Protein Coding	38	GC08P002045	10.51568
SPINK2	Serine Peptidase Inhibitor Kazal Type 2	Protein Coding	38	GC04M056809	10.43731
TMEM70	Transmembrane Protein 70	Protein Coding	38	GC08P073972	10.31983
BOLA3	BolA Family Member 3	Protein Coding	38	GC02M074136	9.952422
ANKS6	Ankyrin Repeat And Sterile Alpha Motif Domain Containing 6	Protein Coding	38	GC09M098731	9.841045

PUS3	Pseudouridine Synthase 3	Protein Coding	38	GC11M125893	9.801666
ANO5	Anoctamin 5	Protein Coding	38	GC11P021799	9.798937
UCN	Urocortin	Protein Coding	38	GC02M027308	9.713901
SETD5	SET Domain Containing 5	Protein Coding	38	GC03P009402	9.163449
DNAH9	Dynein Axonemal Heavy Chain 9	Protein Coding	38	GC17P011598	9.086836
MSH4	MutS Homolog 4	Protein Coding	38	GC01P075796	8.868563
NDUFA11	NADH:Ubiquinone Oxidoreductase Subunit A11	Protein Coding	38	GC19M005891	8.788351
SPECC1L	Sperm Antigen With Calponin Homology And Coiled-Coil Domains 1 Like	Protein Coding	38	GC22P026707	8.461266
KIF6	Kinesin Family Member 6	Protein Coding	38	GC06M047055	8.323478
ANKRD11	Ankyrin Repeat Domain 11	Protein Coding	38	GC16M089267	8.234417
ESCO2	Establishment Of Sister Chromatid Cohesion N-Acetyltransferase 2	Protein Coding	38	GC08P027771	8.183669
FOXJ1	Forkhead Box J1	Protein Coding	38	GC17M076136	8.04356
TMEM127	Transmembrane Protein 127	Protein Coding	38	GC02M096248	7.925472
RSPH9	Radial Spoke Head Component 9	Protein Coding	38	GC06P055414	7.677914
RSPH4A	Radial Spoke Head Component 4A	Protein Coding	38	GC06P116616	7.602224
KLHL41	Kelch Like Family Member 41	Protein Coding	38	GC02P169509	7.344705
VIPAS39	VPS33B Interacting Protein, Apical-Basolateral Polarity Regulator, Spe-39 Homolog	Protein Coding	38	GC14M077426	7.305963
IQSEC2	IQ Motif And Sec7 Domain ArfGEF 2	Protein Coding	38	GC0XM053225	7.260769
TBC1D24	TBC1 Domain Family Member 24	Protein Coding	38	GC16P002475	6.993883
TCF20	Transcription Factor 20	Protein Coding	38	GC22M042160	6.909833
ARL13B	ADP Ribosylation Factor Like GTPase 13B	Protein Coding	38	GC03P093980	6.908245
COX20	Cytochrome C Oxidase Assembly Factor COX20	Protein Coding	38	GC01P244839	6.860646
WDR11	WD Repeat Domain 11	Protein Coding	38	GC10P120851	6.735459
ADAMTS6	ADAM Metallopeptidase With Thrombospondin Type 1 Motif 6	Protein Coding	38	GC05M065148	6.639142
EOGT	EGF Domain Specific O-Linked N-Acetylglucosamine Transferase	Protein Coding	38	GC03M068975	6.52559
ATPAF2	ATP Synthase Mitochondrial F1 Complex Assembly Factor 2	Protein Coding	38	GC17M017977	6.516889
FAHD1	Fumarylacetoacetate Hydrolase Domain Containing 1	Protein Coding	38	GC16P001826	6.508505
RBFOX1	RNA Binding Fox-1 Homolog 1	Protein Coding	38	GC16P005688	6.486173
USH2A	Usherin	Protein Coding	38	GC01M215622	6.377231
TTC7A	Tetratricopeptide Repeat Domain 7A	Protein Coding	38	GC02P046906	6.36997
OTULIN	OTU Deubiquitinase With Linear Linkage Specificity	Protein Coding	38	GC05P014667	6.279675
COG6	Component Of Oligomeric Golgi Complex 6	Protein Coding	38	GC13P039655	6.210562
PDLIM3	PDZ And LIM Domain 3	Protein Coding	38	GC04M185500	6.147621
NSMF	NMDA Receptor Synaptonuclear Signaling And Neuronal Migration Factor	Protein Coding	38	GC09M137447	6.075405
TSEN54	TRNA Splicing Endonuclease Subunit 54	Protein Coding	38	GC17P075515	5.923438
NSUN6	NOP2/Sun RNA Methyltransferase 6	Protein Coding	38	GC10M018530	5.874545
SUN2	Sad1 And UNC84 Domain Containing 2	Protein Coding	38	GC22M048522	5.767784
SLC51A	Solute Carrier Family 51 Subunit Alpha	Protein Coding	38	GC03P196211	5.667567
ZMYND10	Zinc Finger MYND-Type Containing 10	Protein Coding	38	GC03M050435	5.666062
FAM13A	Family With Sequence Similarity 13 Member A	Protein Coding	38	GC04M088725	5.351748
IL1RAPL2	Interleukin 1 Receptor Accessory Protein Like 2	Protein Coding	38	GC0XP104566	5.340609
FLRT2	Fibronectin Leucine Rich Transmembrane Protein 2	Protein Coding	38	GC14P085527	5.333729
CENPT	Centromere Protein T	Protein Coding	38	GC16M067828	5.2486
SRA1	Steroid Receptor RNA Activator 1	Protein Coding	38	GC05M140537	5.189541
PLVAP	Plasmalemma Vesicle Associated Protein	Protein Coding	38	GC19M017351	5.160024
WDR62	WD Repeat Domain 62	Protein Coding	38	GC19P036054	5.14066
TMOD1	Tropomodulin 1	Protein Coding	38	GC09P097501	5.108494
BANK1	B Cell Scaffold Protein With Ankyrin Repeats 1	Protein Coding	38	GC04P101411	5.094925
UBAC2	UBA Domain Containing 2	Protein Coding	38	GC13P099200	5.089686
RFT1	RFT1 Homolog	Protein Coding	38	GC03M053071	5.072164
ISCA1	Iron-Sulfur Cluster Assembly 1	Protein Coding	38	GC09M086264	5.06581
CLN6	CLN6 Transmembrane ER Protein	Protein Coding	38	GC15M068206	5.058094
PKDCC	Protein Kinase Domain Containing, Cytoplasmic	Protein Coding	38	GC02P042049	5.053758

NFKBIL1	NFKB Inhibitor Like 1	Protein Coding	38	GC06P055199	5.045069
CCL17	C-C Motif Chemokine Ligand 17	Protein Coding	38	GC16P057403	5.030348
NCAPH2	Non-SMC Condensin II Complex Subunit H2	Protein Coding	38	GC22P050508	5.027614
TRIM8	Tripartite Motif Containing 8	Protein Coding	38	GC10P102643	5.004792
THOC6	THO Complex 6	Protein Coding	38	GC16P003024	4.970749
PGAP3	Post-GPI Attachment To Proteins Phospholipase 3	Protein Coding	38	GC17M039676	4.923782
DCAF8	DDB1 And CUL4 Associated Factor 8	Protein Coding	38	GC01M160215	4.917663
DSPP	Dentin Sialophosphoprotein	Protein Coding	38	GC04P087608	4.903124
ALG13	ALG13 UDP-N-Acetylglucosaminyltransferase Subunit	Protein Coding	38	GC0XP111665	4.901147
PIEZO1	Piezo Type Mechanosensitive Ion Channel Component 1	Protein Coding	38	GC16M088715	4.890925
SULT1A3	Sulfotransferase Family 1A Member 3	Protein Coding	38	GC16P030199	4.888983
ADAMTS9	ADAM Metallopeptidase With Thrombospondin Type 1 Motif 9	Protein Coding	38	GC03M064501	4.808976
OBSL1	Obscurin Like Cytoskeletal Adaptor 1	Protein Coding	38	GC02M219550	4.752884
IBSP	Integrin Binding Sialoprotein	Protein Coding	38	GC04P087799	4.738823
HOGA1	4-Hydroxy-2-Oxoglutarate Aldolase 1	Protein Coding	38	GC10P097585	4.688
TBL1X	Transducin Beta Like 1 X-Linked	Protein Coding	38	GC0XP009463	4.622704
DDX59	DEAD-Box Helicase 59	Protein Coding	38	GC01M200594	4.567599
LRIG3	Leucine Rich Repeats And Immunoglobulin Like Domains 3	Protein Coding	38	GC12M058872	4.557309
SRRT	Serrate, RNA Effector Molecule	Protein Coding	38	GC07P100875	4.514391
AP4E1	Adaptor Related Protein Complex 4 Subunit Epsilon 1	Protein Coding	38	GC15P050908	4.502127
FOXL1	Forkhead Box L1	Protein Coding	38	GC16P086576	4.454496
LGALS4	Galectin 4	Protein Coding	38	GC19M046731	4.421554
CDAN1	Codanin 1	Protein Coding	38	GC15M042723	4.413365
CKMT1B	Creatine Kinase, Mitochondrial 1B	Protein Coding	38	GC15P043593	4.379123
SH3TC2	SH3 Domain And Tetratricopeptide Repeats 2	Protein Coding	38	GC05M148923	4.351087
NBR1	NBR1 Autophagy Cargo Receptor	Protein Coding	38	GC17P043170	4.346187
LYRM4	LYR Motif Containing 4	Protein Coding	38	GC06M005032	4.342471
KARS1	Lysyl-TRNA Synthetase 1	Protein Coding	38	GC16M075628	4.330908
LCLAT1	Lysocardiolipin Acyltransferase 1	Protein Coding	38	GC02P030447	4.320433
NCR1	Natural Cytotoxicity Triggering Receptor 1	Protein Coding	38	GC19P054906	4.293808
IRX3	Iroquois Homeobox 3	Protein Coding	38	GC16M054283	4.292522
NUP205	Nucleoporin 205	Protein Coding	38	GC07P135557	4.26369
TASP1	Taspase 1	Protein Coding	38	GC20M013105	4.255307
NOX5	NADPH Oxidase 5	Protein Coding	38	GC15P077106	4.18949
TTC14	Tetratricopeptide Repeat Domain 14	Protein Coding	38	GC03P180602	4.156258
TRIB1	Tribbles Pseudokinase 1	Protein Coding	38	GC08P125430	4.151433
PLSCR3	Phospholipid Scramblase 3	Protein Coding	38	GC17M007389	4.093365
PHACTR1	Phosphatase And Actin Regulator 1	Protein Coding	38	GC06P012717	4.082313
BOLL	Boule Homolog, RNA Binding Protein	Protein Coding	38	GC02M197726	4.043375
NUDT6	Nudix Hydrolase 6	Protein Coding	38	GC04M122888	4.036973
OTUD7A	OTU Deubiquitinase 7A	Protein Coding	38	GC15M031475	3.985746
EMILIN1	Elastin Microfibril Interfacer 1	Protein Coding	38	GC02P027078	3.905554
ZBTB24	Zinc Finger And BTB Domain Containing 24	Protein Coding	38	GC06M109462	3.865561
NADK2	NAD Kinase 2, Mitochondrial	Protein Coding	38	GC05M036194	3.844696
NAV2	Neuron Navigator 2	Protein Coding	38	GC11P019345	3.815376
AVIL	Advillin	Protein Coding	38	GC12M057793	3.801826
SPEN	Spen Family Transcriptional Repressor	Protein Coding	38	GC01P015848	3.796606
LYST	Lysosomal Trafficking Regulator	Protein Coding	38	GC01M235661	3.792261
CHD9	Chromodomain Helicase DNA Binding Protein 9	Protein Coding	38	GC16P053041	3.779923
MDN1	Midasin AAA ATPase 1	Protein Coding	38	GC06M089642	3.779104
NECTIN1	Nectin Cell Adhesion Molecule 1	Protein Coding	38	GC11M119624	3.765577
PLEKHG2	Pleckstrin Homology And RhoGEF Domain Containing G2	Protein Coding	38	GC19P039412	3.759277
ASXL2	ASXL Transcriptional Regulator 2	Protein Coding	38	GC02M025733	3.740853

CCL26	C-C Motif Chemokine Ligand 26	Protein Coding	38	GC07M075769	3.733751
SYNPO	Synaptopodin	Protein Coding	38	GC05P150601	3.705975
FXYD1	FXYD Domain Containing Ion Transport Regulator 1	Protein Coding	38	GC19P040341	3.695746
POMK	Protein O-Mannose Kinase	Protein Coding	38	GC08P043093	3.661594
PENK	Proenkephalin	Protein Coding	38	GC08M056436	3.661092
RAB3IP	RAB3A Interacting Protein	Protein Coding	38	GC12P069738	3.638921
ZNF687	Zinc Finger Protein 687	Protein Coding	38	GC01P151281	3.597318
POU2AF1	POU Class 2 Homeobox Associating Factor 1	Protein Coding	38	GC11M111352	3.589818
PSRC1	Proline And Serine Rich Coiled-Coil 1	Protein Coding	38	GC01M109279	3.572458
CMTM7	CKLF Like MARVEL Transmembrane Domain Containing 7	Protein Coding	38	GC03P032409	3.569572
TSLP	Thymic Stromal Lymphopoietin	Protein Coding	38	GC05P111070	3.565145
RHOBTB1	Rho Related BTB Domain Containing 1	Protein Coding	38	GC10M060869	3.554325
BNC2	Basonuclin 2	Protein Coding	38	GC09M016410	3.5525
ZFHX4	Zinc Finger Homeobox 4	Protein Coding	38	GC08P076681	3.542215
RACK1	Receptor For Activated C Kinase 1	Protein Coding	38	GC05M181374	3.537018
CKMT1A	Creatine Kinase, Mitochondrial 1A	Protein Coding	38	GC15P043693	3.519287
ZPBP	Zona Pellucida Binding Protein	Protein Coding	38	GC07M049850	3.506841
WDR37	WD Repeat Domain 37	Protein Coding	38	GC10P001066	3.504303
TMEM30A	Transmembrane Protein 30A	Protein Coding	38	GC06M075252	3.483837
ACAD11	Acyl-CoA Dehydrogenase Family Member 11	Protein Coding	38	GC03M132559	3.464671
ADGRL2	Adhesion G Protein-Coupled Receptor L2	Protein Coding	38	GC01P081306	3.457305
TBC1D20	TBC1 Domain Family Member 20	Protein Coding	38	GC20M000423	3.448245
MICAL2	Microtubule Associated Monooxygenase, Calponin And LIM Domain Containing 2	Protein Coding	38	GC11P012115	3.436709
CSMD1	CUB And Sushi Multiple Domains 1	Protein Coding	38	GC08M002953	3.428814
NEIL3	Nei Like DNA Glycosylase 3	Protein Coding	38	GC04P177309	3.424082
GLT8D1	Glycosyltransferase 8 Domain Containing 1	Protein Coding	38	GC03M052694	3.420405
CEBPD	CCAAT Enhancer Binding Protein Delta	Protein Coding	38	GC08M047759	3.397817
SLC52A2	Solute Carrier Family 52 Member 2	Protein Coding	38	GC08P144333	3.393648
ZNF180	Zinc Finger Protein 180	Protein Coding	38	GC19M046937	3.376842
SLC25A28	Solute Carrier Family 25 Member 28	Protein Coding	38	GC10M099610	3.360578
CALR3	Calreticulin 3	Protein Coding	38	GC19M016450	3.346812
RIPPLY2	Ripply Transcriptional Repressor 2	Protein Coding	38	GC06P083854	3.340168
ASIP	Agouti Signaling Protein	Protein Coding	38	GC20P034194	3.331712
FGGY	FGGY Carbohydrate Kinase Domain Containing	Protein Coding	38	GC01P059296	3.321005
LBX1	Ladybird Homeobox 1	Protein Coding	38	GC10M101226	3.305722
IER3IP1	Immediate Early Response 3 Interacting Protein 1	Protein Coding	38	GC18M047152	3.299881
ARHGAP10	Rho GTPase Activating Protein 10	Protein Coding	38	GC04P147732	3.296628
CNTN3	Contactin 3	Protein Coding	38	GC03M074232	3.294101
FOXI1	Forkhead Box I1	Protein Coding	38	GC05P170105	3.291411
PNPLA4	Patatin Like Phospholipase Domain Containing 4	Protein Coding	38	GC0XM007898	3.285611
PPP1R14C	Protein Phosphatase 1 Regulatory Inhibitor Subunit 14C	Protein Coding	38	GC06P150143	3.278379
SMPD4	Sphingomyelin Phosphodiesterase 4	Protein Coding	38	GC02M130151	3.278109
UCN3	Urocortin 3	Protein Coding	38	GC10P005396	3.24827
UGGT1	UDP-Glucose Glycoprotein Glucosyltransferase 1	Protein Coding	38	GC02P128091	3.206691
ABO	ABO, Alpha 1-3-N-Acetylgalactosaminyltransferase And Alpha 1-3-Galactosyltransferase	Protein Coding	38	GC09M133250	3.179779
IFI27	Interferon Alpha Inducible Protein 27	Protein Coding	38	GC14P094104	3.166371
RIN3	Ras And Rab Interactor 3	Protein Coding	38	GC14P092513	3.16357
CCDC50	Coiled-Coil Domain Containing 50	Protein Coding	38	GC03P191329	3.15869
SARM1	Sterile Alpha And TIR Motif Containing 1	Protein Coding	38	GC17P028364	3.142136
CPNE4	Copine 4	Protein Coding	38	GC03M131533	3.126773
CHD6	Chromodomain Helicase DNA Binding Protein 6	Protein Coding	38	GC20M041402	3.116465
TNS3	Tensin 3	Protein Coding	38	GC07M047281	3.097669
PCDH1	Protocadherin 1	Protein Coding	38	GC05M141872	3.079414

SSBP3	Single Stranded DNA Binding Protein 3	Protein Coding	38	GC01M054225	3.058805
UFC1	Ubiquitin-Fold Modifier Conjugating Enzyme 1	Protein Coding	38	GC01P161177	3.057997
SEMA6B	Semaphorin 6B	Protein Coding	38	GC19M004542	3.048305
REC8	REC8 Meiotic Recombination Protein	Protein Coding	38	GC14P024171	3.041111
TMOD2	Tropomodulin 2	Protein Coding	38	GC15P051751	3.025723
SLC41A1	Solute Carrier Family 41 Member 1	Protein Coding	38	GC01M205789	3.022546
NSUN5	NOP2/Sun RNA Methyltransferase 5	Protein Coding	38	GC07M073302	3.010379
COL15A1	Collagen Type XV Alpha 1 Chain	Protein Coding	38	GC09P098943	3.003441
ESCO1	Establishment Of Sister Chromatid Cohesion N-Acetyltransferase 1	Protein Coding	38	GC18M021529	2.9952
MBNL2	Muscleblind Like Splicing Regulator 2	Protein Coding	38	GC13P097141	2.973657
TINAG	Tubulointerstitial Nephritis Antigen	Protein Coding	38	GC06P055534	2.969884
PIWIL2	Piwi Like RNA-Mediated Gene Silencing 2	Protein Coding	38	GC08P022275	2.965417
ESPN	Espin	Protein Coding	38	GC01P006424	2.961941
NPNT	Nephronectin	Protein Coding	38	GC04P105894	2.901978
TRIB2	Tribbles Pseudokinase 2	Protein Coding	38	GC02P012706	2.888503
FSTL3	Follistatin Like 3	Protein Coding	38	GC19P000676	2.880682
LCOR	Ligand Dependent Nuclear Receptor Corepressor	Protein Coding	38	GC10P096832	2.864367
HNRNPUL1	Heterogeneous Nuclear Ribonucleoprotein U Like 1	Protein Coding	38	GC19P041262	2.862917
PARP10	Poly(ADP-Ribose) Polymerase Family Member 10	Protein Coding	38	GC08M143977	2.859194
SP140	SP140 Nuclear Body Protein	Protein Coding	38	GC02P230203	2.840676
GPER1	G Protein-Coupled Estrogen Receptor 1	Protein Coding	38	GC07P001368	2.839258
IL4I1	Interleukin 4 Induced 1	Protein Coding	38	GC19M049890	2.833503
BYSL	Bystin Like	Protein Coding	38	GC06P055392	2.831859
NLRP5	NLR Family Pyrin Domain Containing 5	Protein Coding	38	GC19P056513	2.827771
PDZD2	PDZ Domain Containing 2	Protein Coding	38	GC05P031639	2.823006
OSR2	Odd-Skipped Related Transciption Factor 2	Protein Coding	38	GC08P098944	2.819698
MYL12B	Myosin Light Chain 12B	Protein Coding	38	GC18P003261	2.801547
DYNLT1	Dynein Light Chain Tctex-Type 1	Protein Coding	38	GC06M158636	2.793461
PPP1R9A	Protein Phosphatase 1 Regulatory Subunit 9A	Protein Coding	38	GC07P094907	2.787821
GUCA2A	Guanylate Cyclase Activator 2A	Protein Coding	38	GC01M042162	2.784053
PKP3	Plakophilin 3	Protein Coding	38	GC11P000396	2.781391
SOX30	SRY-Box Transcription Factor 30	Protein Coding	38	GC05M157624	2.780543
GORAB	Golgin, RAB6 Interacting	Protein Coding	38	GC01P170501	2.779157
TAX1BP3	Tax1 Binding Protein 3	Protein Coding	38	GC17M003666	2.778248
LGALS7	Galectin 7	Protein Coding	38	GC19M038770	2.76796
LY86	Lymphocyte Antigen 86	Protein Coding	38	GC06P006588	2.76772
PPP1R15B	Protein Phosphatase 1 Regulatory Subunit 15B	Protein Coding	38	GC01M204400	2.767365
AEBP2	AE Binding Protein 2	Protein Coding	38	GC12P019404	2.766614
ANKRD6	Ankyrin Repeat Domain 6	Protein Coding	38	GC06P089433	2.746307
RPL38	Ribosomal Protein L38	Protein Coding	38	GC17P074204	2.740368
SPAG16	Sperm Associated Antigen 16	Protein Coding	38	GC02P213284	2.737606
ZFPM1	Zinc Finger Protein, FOG Family Member 1	Protein Coding	38	GC16P088451	2.737355
LPIN3	Lipin 3	Protein Coding	38	GC20P041340	2.730839
ZNF74	Zinc Finger Protein 74	Protein Coding	38	GC22P020394	2.722164
PNOC	Prepronociceptin	Protein Coding	38	GC08P028316	2.716502
TMOD4	Tropomodulin 4	Protein Coding	38	GC01M151169	2.711392
PAPPA2	Pappalysin 2	Protein Coding	38	GC01P176463	2.692615
TRHDE	Thyrotropin Releasing Hormone Degrading Enzyme	Protein Coding	38	GC12P072087	2.689565
MYBPH	Myosin Binding Protein H	Protein Coding	38	GC01M203136	2.68364
INPP5F	Inositol Polyphosphate-5-Phosphatase F	Protein Coding	38	GC10P119704	2.683536
GEMIN2	Gem Nuclear Organelle Associated Protein 2	Protein Coding	38	GC14P039114	2.679776
TRIM31	Tripartite Motif Containing 31	Protein Coding	38	GC06M046776	2.676269
TOE1	Target Of EGR1, Exonuclease	Protein Coding	38	GC01P045339	2.673054

DNALI1	Dynein Axonemal Light Intermediate Chain 1	Protein Coding	38 GC01P037577	2.659719
DDX10	DEAD-Box Helicase 10	Protein Coding	38 GC11P108569	2.659653
RSL24D1	Ribosomal L24 Domain Containing 1	Protein Coding	38 GC15M055180	2.658138
PACSIN3	Protein Kinase C And Casein Kinase Substrate In Neurons 3	Protein Coding	38 GC11M068990	2.653191
GPSM1	G Protein Signaling Modulator 1	Protein Coding	38 GC09P136327	2.639878
CELF4	CUGBP Elav-Like Family Member 4	Protein Coding	38 GC18M037243	2.632941
NID2	Nidogen 2	Protein Coding	38 GC14M052004	2.618981
TIMM17B	Translocase Of Inner Mitochondrial Membrane 17B	Protein Coding	38 GC0XM048893	2.606067
PIWIL4	Piwi Like RNA-Mediated Gene Silencing 4	Protein Coding	38 GC11P094543	2.600973
EYA2	EYA Transcriptional Coactivator And Phosphatase 2	Protein Coding	38 GC20P046894	2.596478
OMA1	OMA1 Zinc Metallopeptidase	Protein Coding	38 GC01M058415	2.594151
STARD4	StAR Related Lipid Transfer Domain Containing 4	Protein Coding	38 GC05M111496	2.585346
DHX37	DEAH-Box Helicase 37	Protein Coding	38 GC12M124946	2.582839
XAB2	XPA Binding Protein 2	Protein Coding	38 GC19M007619	2.551357
IER3	Immediate Early Response 3	Protein Coding	38 GC06M030743	2.545355
GPN1	GPN-Loop GTPase 1	Protein Coding	38 GC02P027628	2.517781
ERC2	ELKS/RAB6-Interacting/CAST Family Member 2	Protein Coding	38 GC03M055509	2.516027
RPS25	Ribosomal Protein S25	Protein Coding	38 GC11M119015	2.514396
PTCRA	Pre T Cell Antigen Receptor Alpha	Protein Coding	38 GC06P042915	2.509223
TSSK1B	Testis Specific Serine Kinase 1B	Protein Coding	38 GC05M113432	2.484231
EHBP1	EH Domain Binding Protein 1	Protein Coding	38 GC02P062673	2.473716
RNF220	Ring Finger Protein 220	Protein Coding	38 GC01P044405	2.472141
G6PC2	Glucose-6-Phosphatase Catalytic Subunit 2	Protein Coding	38 GC02P168901	2.468887
TSPAN2	Tetraspanin 2	Protein Coding	38 GC01M115050	2.461798
RHOF	Ras Homolog Family Member F, Filopodia Associated	Protein Coding	38 GC12M121777	2.457932
CIZ1	CDKN1A Interacting Zinc Finger Protein 1	Protein Coding	38 GC09M128166	2.446134
ZNF335	Zinc Finger Protein 335	Protein Coding	38 GC20M045948	2.44496
ELAVL3	ELAV Like RNA Binding Protein 3	Protein Coding	38 GC19M011451	2.426979
ZNF224	Zinc Finger Protein 224	Protein Coding	38 GC19P044094	2.426948
SOX8	SRY-Box Transcription Factor 8	Protein Coding	38 GC16P000981	2.420326
DSCAML1	DS Cell Adhesion Molecule Like 1	Protein Coding	38 GC11M117427	2.419775
BRAT1	BRCA1 Associated ATM Activator 1	Protein Coding	38 GC07M002630	2.418669
HSPA12A	Heat Shock Protein Family A (Hsp70) Member 12A	Protein Coding	38 GC10M116671	2.407046
SIGLEC5	Sialic Acid Binding Ig Like Lectin 5	Protein Coding	38 GC19M051611	2.393449
SDR9C7	Short Chain Dehydrogenase/Reductase Family 9C Member 7	Protein Coding	38 GC12M056923	2.391627
CACNG8	Calcium Voltage-Gated Channel Auxiliary Subunit Gamma 8	Protein Coding	38 GC19P056410	2.387938
CHAF1A	Chromatin Assembly Factor 1 Subunit A	Protein Coding	38 GC19P004402	2.385742
LUC7L	LUC7 Like	Protein Coding	38 GC16M000188	2.383016
EXOG	Exo/Endonuclease G	Protein Coding	38 GC03P038496	2.380851
NUDT21	Nudix Hydrolase 21	Protein Coding	38 GC16M056429	2.379939
RAB11FIP4	RAB11 Family Interacting Protein 4	Protein Coding	38 GC17P031391	2.379405
TSHZ3	Teashirt Zinc Finger Homeobox 3	Protein Coding	38 GC19M031150	2.376821
TXNIP	Thioredoxin Interacting Protein	Protein Coding	38 GC01M145992	2.373784
SH3PXD2A	SH3 And PX Domains 2A	Protein Coding	38 GC10M103594	2.370944
LRRFIP1	LRR Binding FLII Interacting Protein 1	Protein Coding	38 GC02P237627	2.348192
KCNA10	Potassium Voltage-Gated Channel Subfamily A Member 10	Protein Coding	38 GC01M110517	2.342495
ZNF521	Zinc Finger Protein 521	Protein Coding	38 GC18M025061	2.340013
TRMT11	TRNA Methyltransferase 11 Homolog	Protein Coding	38 GC06P125986	2.336889
TNFAIP8	TNF Alpha Induced Protein 8	Protein Coding	38 GC05P119268	2.333285
DRG2	Developmentally Regulated GTP Binding Protein 2	Protein Coding	38 GC17P018088	2.316933
BFAR	Bifunctional Apoptosis Regulator	Protein Coding	38 GC16P014637	2.308834
CCL22	C-C Motif Chemokine Ligand 22	Protein Coding	38 GC16P057359	2.308405
HEATR1	HEAT Repeat Containing 1	Protein Coding	38 GC01M236549	2.308017

RBSN	Rabenosyn, RAB Effector	Protein Coding	38	GC03M015070	2.307147
CDK5RAP1	CDK5 Regulatory Subunit Associated Protein 1	Protein Coding	38	GC20M033358	2.303442
ATG13	Autophagy Related 13	Protein Coding	38	GC11P046629	2.301823
CDCA5	Cell Division Cycle Associated 5	Protein Coding	38	GC11M069368	2.293249
SLC5A4	Solute Carrier Family 5 Member 4	Protein Coding	38	GC22M032218	2.285639
TFB2M	Transcription Factor B2, Mitochondrial	Protein Coding	38	GC01M246540	2.274625
LRRFIP2	LRR Binding FLII Interacting Protein 2	Protein Coding	38	GC03M037052	2.261034
C1QTNF3	C1q And TNF Related 3	Protein Coding	38	GC05M034017	2.259388
CD2BP2	CD2 Cytoplasmic Tail Binding Protein 2	Protein Coding	38	GC16M030350	2.255366
PPP2R3A	Protein Phosphatase 2 Regulatory Subunit B''Alpha	Protein Coding	38	GC03P135965	2.247101
EYA3	EYA Transcriptional Coactivator And Phosphatase 3	Protein Coding	38	GC01M027970	2.239656
ZNF365	Zinc Finger Protein 365	Protein Coding	38	GC10P062374	2.238266
TCEA1	Transcription Elongation Factor A1	Protein Coding	38	GC08M053966	2.229505
SIPA1L1	Signal Induced Proliferation Associated 1 Like 1	Protein Coding	38	GC14P071320	2.217801
ANAPC5	Anaphase Promoting Complex Subunit 5	Protein Coding	38	GC12M121308	2.214694
DCP2	Decapping MRNA 2	Protein Coding	38	GC05P112976	2.213503
RASSF3	Ras Association Domain Family Member 3	Protein Coding	38	GC12P064507	2.210686
SNTB2	Syntrophin Beta 2	Protein Coding	38	GC16P069187	2.2085
CALCB	Calcitonin Related Polypeptide Beta	Protein Coding	38	GC11P014904	2.191106
SEC61B	SEC61 Translocon Subunit Beta	Protein Coding	38	GC09P099222	2.179527
SSR3	Signal Sequence Receptor Subunit 3	Protein Coding	38	GC03M156540	2.173068
PLCL2	Phospholipase C Like 2	Protein Coding	38	GC03P016803	2.171759
DNAJC13	DnaJ Heat Shock Protein Family (Hsp40) Member C13	Protein Coding	38	GC03P132417	2.170658
KATNAL1	Katanin Catalytic Subunit A1 Like 1	Protein Coding	38	GC13M030202	2.164576
GTF3C2	General Transcription Factor IIIC Subunit 2	Protein Coding	38	GC02M027325	2.162302
POU6F2	POU Class 6 Homeobox 2	Protein Coding	38	GC07P038977	2.161507
TIGIT	T Cell Immunoreceptor With Ig And ITIM Domains	Protein Coding	38	GC03P114276	2.158564
MIA2	MIA SH3 Domain ER Export Factor 2	Protein Coding	38	GC14P039230	2.158157
MATN1	Matrilin 1	Protein Coding	38	GC01M030711	2.145523
PKIG	CAMP-Dependent Protein Kinase Inhibitor Gamma	Protein Coding	38	GC20P044531	2.142819
UPK3A	Uroplakin 3A	Protein Coding	38	GC22P045284	2.139251
BMP8A	Bone Morphogenetic Protein 8a	Protein Coding	38	GC01P039492	2.123825
TRIM23	Tripartite Motif Containing 23	Protein Coding	38	GC05M065589	2.112651
BANP	BTG3 Associated Nuclear Protein	Protein Coding	38	GC16P087949	2.11154
PLEKHA7	Pleckstrin Homology Domain Containing A7	Protein Coding	38	GC11M016778	2.104883
PADI6	Peptidyl Arginine Deiminase 6	Protein Coding	38	GC01P017373	2.089491
COX7C	Cytochrome C Oxidase Subunit 7C	Protein Coding	38	GC05P086617	2.0797
IL27	Interleukin 27	Protein Coding	38	GC16M028514	2.078373
FMNL2	Formin Like 2	Protein Coding	38	GC02P152335	2.071444
LGI2	Leucine Rich Repeat LGI Family Member 2	Protein Coding	38	GC04M025000	2.069979
NT5C1B	5'-Nucleotidase, Cytosolic IB	Protein Coding	38	GC02M018562	2.06914
KIF20B	Kinesin Family Member 20B	Protein Coding	38	GC10P089701	2.063117
POU2F3	POU Class 2 Homeobox 3	Protein Coding	38	GC11P120236	2.061202
FTMT	Ferritin Mitochondrial	Protein Coding	38	GC05P121851	2.057998
CBFA2T2	CBFA2/RUNX1 Partner Transcriptional Co-Repressor 2	Protein Coding	38	GC20P033490	2.057498
KCNK5	Potassium Two Pore Domain Channel Subfamily K Member 5	Protein Coding	38	GC06M047054	2.056672
WARS1	Tryptophanyl-TRNA Synthetase 1	Protein Coding	38	GC14M100334	2.048989
UTP6	UTP6 Small Subunit Processome Component	Protein Coding	38	GC17M031860	2.043595
NCOA7	Nuclear Receptor Coactivator 7	Protein Coding	38	GC06P125781	2.042864
DUSP14	Dual Specificity Phosphatase 14	Protein Coding	38	GC17P037489	2.036273
HS3ST3B1	Heparan Sulfate-Glucosamine 3-Sulfotransferase 3B1	Protein Coding	38	GC17P014301	2.035091
PHOSPHO1	Phosphoethanolamine/Phosphocholine Phosphatase 1	Protein Coding	38	GC17M049223	2.03006
WFDC2	WAP Four-Disulfide Core Domain 2	Protein Coding	38	GC20P045469	2.029098

DYNC1LI1	Dynein Cytoplasmic 1 Light Intermediate Chain 1	Protein Coding	38	GC03M032543	2.026491
ARHGAP21	Rho GTPase Activating Protein 21	Protein Coding	38	GC10M024583	2.025437
DDT	D-Dopachrome Tautomerase	Protein Coding	38	GC22M023971	2.0212
AP3M1	Adaptor Related Protein Complex 3 Subunit Mu 1	Protein Coding	38	GC10M074120	2.020309
DDX31	DEAD-Box Helicase 31	Protein Coding	38	GC09M132594	2.019132
SAP30L	SAP30 Like	Protein Coding	38	GC05P154445	2.017139
KRT71	Keratin 71	Protein Coding	38	GC12M052543	2.015783
TSHZ2	Teashirt Zinc Finger Homeobox 2	Protein Coding	38	GC20P052972	2.011648
RALGAPA2	Ral GTPase Activating Protein Catalytic Subunit Alpha 2	Protein Coding	38	GC20M020374	2.011311
USP34	Ubiquitin Specific Peptidase 34	Protein Coding	38	GC02M061187	2.008345
A1BG	Alpha-1-B Glycoprotein	Protein Coding	38	GC19M058345	2.006865
MSL3	MSL Complex Subunit 3	Protein Coding	38	GC0XP011758	2.005848
SLC25A25	Solute Carrier Family 25 Member 25	Protein Coding	38	GC09P128068	2.004268
SHPK	Sedoheptulokinase	Protein Coding	38	GC17M003608	2.000469
STX18	Syntaxin 18	Protein Coding	38	GC04M004417	1.996915
TMX3	Thioredoxin Related Transmembrane Protein 3	Protein Coding	38	GC18M068673	1.995313
PIGW	Phosphatidylinositol Glycan Anchor Biosynthesis Class W	Protein Coding	38	GC17P036534	1.990186
BTBD10	BTB Domain Containing 10	Protein Coding	38	GC11M013387	1.987176
SEC31B	SEC31 Homolog B, COPII Coat Complex Component	Protein Coding	38	GC10M100486	1.982888
NMRK2	Nicotinamide Riboside Kinase 2	Protein Coding	38	GC19P003933	1.980567
ATP11C	ATPase Phospholipid Transporting 11C	Protein Coding	38	GC0XM139726	1.977941
CLEC12A	C-Type Lectin Domain Family 12 Member A	Protein Coding	38	GC12P009951	1.971136
OSBPL3	Oxysterol Binding Protein Like 3	Protein Coding	38	GC07M024836	1.967588
AIG1	Androgen Induced 1	Protein Coding	38	GC06P143059	1.965682
PLA2G4E	Phospholipase A2 Group IVE	Protein Coding	38	GC15M041981	1.963538
ANKK1	Ankyrin Repeat And Kinase Domain Containing 1	Protein Coding	38	GC11P113387	1.962367
SUPV3L1	Suv3 Like RNA Helicase	Protein Coding	38	GC10P069182	1.955058
ALYREF	Aly/REF Export Factor	Protein Coding	38	GC17M081887	1.951527
PROKR1	Prokineticin Receptor 1	Protein Coding	38	GC02P068643	1.946632
DHX34	DExH-Box Helicase 34	Protein Coding	38	GC19P047349	1.944934
OLFM4	Olfactomedin 4	Protein Coding	38	GC13P053028	1.941931
ACSM3	Acyl-CoA Synthetase Medium Chain Family Member 3	Protein Coding	38	GC16P020610	1.937073
ELF5	E74 Like ETS Transcription Factor 5	Protein Coding	38	GC11M034500	1.936161
DRAM2	DNA Damage Regulated Autophagy Modulator 2	Protein Coding	38	GC01M111117	1.935205
NAT8	N-Acetyltransferase 8 (Putative)	Protein Coding	38	GC02M073640	1.926806
DERL1	Derlin 1	Protein Coding	38	GC08M123013	1.926381
CLCA4	Chloride Channel Accessory 4	Protein Coding	38	GC01P086547	1.925
NGB	Neuroglobin	Protein Coding	38	GC14M077265	1.924724
ZBTB32	Zinc Finger And BTB Domain Containing 32	Protein Coding	38	GC19P040360	1.921821
AGO1	Argonaute RISC Component 1	Protein Coding	38	GC01P035869	1.916052
CGNL1	Cingulin Like 1	Protein Coding	38	GC15P057375	1.911363
SEPHS2	Selenophosphate Synthetase 2	Protein Coding	38	GC16M031271	1.911065
REXO2	RNA Exonuclease 2	Protein Coding	38	GC11P114439	1.909115
FBXO3	F-Box Protein 3	Protein Coding	38	GC11M033740	1.908036
GKN1	Gastrokine 1	Protein Coding	38	GC02P068974	1.895134
USP42	Ubiquitin Specific Peptidase 42	Protein Coding	38	GC07P006078	1.893377
MUC4	Mucin 4, Cell Surface Associated	Protein Coding	38	GC03M195746	1.889988
ALKBH8	AlkB Homolog 8, TRNA Methyltransferase	Protein Coding	38	GC11M107502	1.876788
SNTB1	Syntrophin Beta 1	Protein Coding	38	GC08M120535	1.863672
MCM10	Minichromosome Maintenance 10 Replication Initiation Factor	Protein Coding	38	GC10P013161	1.863214
NARF	Nuclear Prelamin A Recognition Factor	Protein Coding	38	GC17P082458	1.857749
CENPU	Centromere Protein U	Protein Coding	38	GC04M184694	1.855075
CENPB	Centromere Protein B	Protein Coding	38	GC20M003783	1.851095

PLXNA3	Plexin A3	Protein Coding	38 GC0XP154458	1.850664
KCNH4	Potassium Voltage-Gated Channel Subfamily H Member 4	Protein Coding	38 GC17M042160	1.84653
ARFRP1	ADP Ribosylation Factor Related Protein 1	Protein Coding	38 GC20M063698	1.845925
POLR3H	RNA Polymerase III Subunit H	Protein Coding	38 GC22M041525	1.822077
E2F7	E2F Transcription Factor 7	Protein Coding	38 GC12M077021	1.819761
ACKR2	Atypical Chemokine Receptor 2	Protein Coding	38 GC03P042804	1.816848
POC1A	POC1 Centriolar Protein A	Protein Coding	38 GC03M052109	1.814603
PCDH9	Protocadherin 9	Protein Coding	38 GC13M066302	1.811791
HNRNPA3	Heterogeneous Nuclear Ribonucleoprotein A3	Protein Coding	38 GC02P177212	1.810895
RMI1	RecQ Mediated Genome Instability 1	Protein Coding	38 GC09P083980	1.8059
CREG1	Cellular Repressor Of E1A Stimulated Genes 1	Protein Coding	38 GC01M167498	1.803035
NUF2	NUF2 Component Of NDC80 Kinetochore Complex	Protein Coding	38 GC01P163266	1.802901
TAAR1	Trace Amine Associated Receptor 1	Protein Coding	38 GC06M132644	1.799131
CHPF	Chondroitin Polymerizing Factor	Protein Coding	38 GC02M219538	1.794005
NANP	N-Acetylneuraminic Acid Phosphatase	Protein Coding	38 GC20M025613	1.789577
CGN	Cingulin	Protein Coding	38 GC01P151483	1.787091
CHCHD3	Coiled-Coil-Helix-Coiled-Coil-Helix Domain Containing 3	Protein Coding	38 GC07M132784	1.785967
GPR3	G Protein-Coupled Receptor 3	Protein Coding	38 GC01P027393	1.780257
RTN1	Reticulon 1	Protein Coding	38 GC14M059595	1.777467
BTG2	BTG Anti-Proliferation Factor 2	Protein Coding	38 GC01P203305	1.776403
DDX21	DExD-Box Helicase 21	Protein Coding	38 GC10P068956	1.770859
DPYSL5	Dihydropyrimidinase Like 5	Protein Coding	38 GC02P026847	1.770388
HIGD1A	HIG1 Hypoxia Inducible Domain Family Member 1A	Protein Coding	38 GC03M042782	1.76928
LONP2	Lon Peptidase 2, Peroxisomal	Protein Coding	38 GC16P048244	1.767413
SMC1B	Structural Maintenance Of Chromosomes 1B	Protein Coding	38 GC22M045344	1.764935
MADCAM1	Mucosal Vascular Addressin Cell Adhesion Molecule 1	Protein Coding	38 GC19P000499	1.759793
MIA3	MIA SH3 Domain ER Export Factor 3	Protein Coding	38 GC01P222618	1.756257
CLEC4A	C-Type Lectin Domain Family 4 Member A	Protein Coding	38 GC12P011898	1.751025
VAX1	Ventral Anterior Homeobox 1	Protein Coding	38 GC10M117128	1.750784
TUBGCP3	Tubulin Gamma Complex Associated Protein 3	Protein Coding	38 GC13M112485	1.749771
SSX2	SSX Family Member 2	Protein Coding	38 GC0XM052696	1.739332
DENND1A	DENN Domain Containing 1A	Protein Coding	38 GC09M123379	1.736536
KIF16B	Kinesin Family Member 16B	Protein Coding	38 GC20M016272	1.735478
RPS27L	Ribosomal Protein S27 Like	Protein Coding	38 GC15M067355	1.732973
IL17D	Interleukin 17D	Protein Coding	38 GC13P020702	1.732513
CELA3B	Chymotrypsin Like Elastase 3B	Protein Coding	38 GC01P021976	1.728904
TCEA2	Transcription Elongation Factor A2	Protein Coding	38 GC20P064517	1.724752
CCL13	C-C Motif Chemokine Ligand 13	Protein Coding	38 GC17P034356	1.72385
ARHGEF19	Rho Guanine Nucleotide Exchange Factor 19	Protein Coding	38 GC01M016197	1.72366
VPS41	VPS41 Subunit Of HOPS Complex	Protein Coding	38 GC07M038786	1.721553
GCC2	GRIP And Coiled-Coil Domain Containing 2	Protein Coding	38 GC02P108432	1.71087
SEMA4C	Semaphorin 4C	Protein Coding	38 GC02M096859	1.709488
DPH2	Diphthamide Biosynthesis 2	Protein Coding	38 GC01P043969	1.706518
NME5	NME/NM23 Family Member 5	Protein Coding	38 GC05M138115	1.704951
VPREB1	V-Set Pre-B Cell Surrogate Light Chain 1	Protein Coding	38 GC22P022244	1.697004
SEMA6C	Semaphorin 6C	Protein Coding	38 GC01M151131	1.694191
FASTK	Fas Activated Serine/Threonine Kinase	Protein Coding	38 GC07M151077	1.69406
CHCHD2	Coiled-Coil-Helix-Coiled-Coil-Helix Domain Containing 2	Protein Coding	38 GC07M056101	1.693059
TWSG1	Twisted Gastrulation BMP Signaling Modulator 1	Protein Coding	38 GC18P009334	1.687479
KRT83	Keratin 83	Protein Coding	38 GC12M052314	1.685899
MFAP3	Microfibril Associated Protein 3	Protein Coding	38 GC05P154014	1.685246
MTMR7	Myotubularin Related Protein 7	Protein Coding	38 GC08M017296	1.684799
SNX18	Sorting Nexin 18	Protein Coding	38 GC05P054517	1.674425

ARID3B	AT-Rich Interaction Domain 3B	Protein Coding	38	GC15P074541	1.673388
SPOCK3	SPARC (Osteonectin), Cwcv And Kazal Like Domains Proteoglycan 3	Protein Coding	38	GC04M166733	1.67285
PPM1L	Protein Phosphatase, Mg2+/Mn2+ Dependent 1L	Protein Coding	38	GC03P160755	1.66583
AP4S1	Adaptor Related Protein Complex 4 Subunit Sigma 1	Protein Coding	38	GC14P031025	1.661359
RAB3IL1	RAB3A Interacting Protein Like 1	Protein Coding	38	GC11M061897	1.660887
MRPL40	Mitochondrial Ribosomal Protein L40	Protein Coding	38	GC22P020060	1.659527
PHLDA2	Pleckstrin Homology Like Domain Family A Member 2	Protein Coding	38	GC11M002928	1.658861
RP1	RP1 Axonemal Microtubule Associated	Protein Coding	38	GC08P054555	1.657778
MYEF2	Myelin Expression Factor 2	Protein Coding	38	GC15M048134	1.655289
ADHFE1	Alcohol Dehydrogenase Iron Containing 1	Protein Coding	38	GC08P066432	1.651309
CBLC	Cbl Proto-Oncogene C	Protein Coding	38	GC19P044777	1.643264
MAN2C1	Mannosidase Alpha Class 2C Member 1	Protein Coding	38	GC15M075355	1.640669
CSH1	Chorionic Somatomammotropin Hormone 1	Protein Coding	38	GC17M063894	1.640039
MKLN1	Muskelin 1	Protein Coding	38	GC07P131110	1.634224
CTBS	Chitobiase	Protein Coding	38	GC01M084549	1.633714
NKX2-3	NK2 Homeobox 3	Protein Coding	38	GC10P099532	1.625218
SLC8B1	Solute Carrier Family 8 Member B1	Protein Coding	38	GC12M113298	1.613594
MARCKSL1	MARCKS Like 1	Protein Coding	38	GC01M032334	1.606867
MFF	Mitochondrial Fission Factor	Protein Coding	38	GC02P227325	1.606024
DHX15	DEAH-Box Helicase 15	Protein Coding	38	GC04M024519	1.599242
RNF111	Ring Finger Protein 111	Protein Coding	38	GC15P058866	1.590254
SOBP	Sine Oculis Binding Protein Homolog	Protein Coding	38	GC06P107489	1.587398
ALKBH3	AlkB Homolog 3, Alpha-Ketoglutarate Dependent Dioxygenase	Protein Coding	38	GC11P043902	1.587264
DMAP1	DNA Methyltransferase 1 Associated Protein 1	Protein Coding	38	GC01P044214	1.583629
ZFP64	ZFP64 Zinc Finger Protein	Protein Coding	38	GC20M052051	1.580563
HOXD11	Homeobox D11	Protein Coding	38	GC02P176104	1.578791
TAF1C	TATA-Box Binding Protein Associated Factor, RNA Polymerase I Subunit C	Protein Coding	38	GC16M084177	1.578764
MMP28	Matrix Metallopeptidase 28	Protein Coding	38	GC17M035756	1.576061
FBXW5	F-Box And WD Repeat Domain Containing 5	Protein Coding	38	GC09M136941	1.575883
SLC16A12	Solute Carrier Family 16 Member 12	Protein Coding	38	GC10M089430	1.572978
NAA50	N-Alpha-Acetyltransferase 50, NatE Catalytic Subunit	Protein Coding	38	GC03M113716	1.568468
OSBPL6	Oxysterol Binding Protein Like 6	Protein Coding	38	GC02P178194	1.56588
RDH8	Retinol Dehydrogenase 8	Protein Coding	38	GC19P010014	1.560013
DEGS2	Delta 4-Desaturase, Sphingolipid 2	Protein Coding	38	GC14M100143	1.559837
GPS1	G Protein Pathway Suppressor 1	Protein Coding	38	GC17P082050	1.557114
SYNGR2	Synaptogyrin 2	Protein Coding	38	GC17P078168	1.550035
IRGM	Immunity Related GTPase M	Protein Coding	38	GC05P150846	1.549413
DAZAP2	DAZ Associated Protein 2	Protein Coding	38	GC12P051238	1.549306
SCFD1	Sec1 Family Domain Containing 1	Protein Coding	38	GC14P030622	1.539344
NUTF2	Nuclear Transport Factor 2	Protein Coding	38	GC16P067846	1.534571
NPRL3	NPR3 Like, GATOR1 Complex Subunit	Protein Coding	38	GC16M000084	1.532747
PRPF40A	Pre-MRNA Processing Factor 40 Homolog A	Protein Coding	38	GC02M152651	1.527202
RPL36AL	Ribosomal Protein L36a Like	Protein Coding	38	GC14M049619	1.514938
THRAP3	Thyroid Hormone Receptor Associated Protein 3	Protein Coding	38	GC01P036224	1.513174
DAZAP1	DAZ Associated Protein 1	Protein Coding	38	GC19P001407	1.508952
CEACAM8	CEA Cell Adhesion Molecule 8	Protein Coding	38	GC19M042580	1.504607
ACOT13	Acyl-CoA Thioesterase 13	Protein Coding	38	GC06P024667	1.503268
IPO7	Importin 7	Protein Coding	38	GC11P009384	1.499292
IST1	IST1 Factor Associated With ESCRT-III	Protein Coding	38	GC16P071849	1.497787
MEF2B	Myocyte Enhancer Factor 2B	Protein Coding	38	GC19M019150	1.496724
TET1	Tet Methylcytosine Dioxygenase 1	Protein Coding	38	GC10P068560	1.496385
IFNK	Interferon Kappa	Protein Coding	38	GC09P027514	1.496315
TOX3	TOX High Mobility Group Box Family Member 3	Protein Coding	38	GC16M052440	1.492241

PGM5	Phosphoglucomutase 5	Protein Coding	38	GC09P068328	1.491284
AMOTL1	Angiomotin Like 1	Protein Coding	38	GC11P094686	1.490198
OLIG3	Oligodendrocyte Transcription Factor 3	Protein Coding	38	GC06M137492	1.481456
AIFM3	Apoptosis Inducing Factor Mitochondria Associated 3	Protein Coding	38	GC22P020965	1.475006
DNAJB4	DnaJ Heat Shock Protein Family (Hsp40) Member B4	Protein Coding	38	GC01P077979	1.468965
MMP23B	Matrix Metallopeptidase 23B	Protein Coding	38	GC01P001631	1.468053
MYLK4	Myosin Light Chain Kinase Family Member 4	Protein Coding	38	GC06M002663	1.466642
EXOSC7	Exosome Component 7	Protein Coding	38	GC03P045602	1.464787
MYCBP2	MYC Binding Protein 2	Protein Coding	38	GC13M077044	1.463336
TM9SF2	Transmembrane 9 Superfamily Member 2	Protein Coding	38	GC13P099446	1.462943
TRAPPC12	Trafficking Protein Particle Complex Subunit 12	Protein Coding	38	GC02P003383	1.462228
RNH1	Ribonuclease/Angiogenin Inhibitor 1	Protein Coding	38	GC11M001079	1.461787
TRIM68	Tripartite Motif Containing 68	Protein Coding	38	GC11M004577	1.458789
SEMA3G	Semaphorin 3G	Protein Coding	38	GC03M052433	1.45574
PBX2	PBX Homeobox 2	Protein Coding	38	GC06M032184	1.452837
PDPR	Pyruvate Dehydrogenase Phosphatase Regulatory Subunit	Protein Coding	38	GC16P070113	1.450446
KLK14	Kallikrein Related Peptidase 14	Protein Coding	38	GC19M051077	1.445767
TAGAP	T Cell Activation RhoGTPase Activating Protein	Protein Coding	38	GC06M159034	1.441298
PHC3	Polyhomeotic Homolog 3	Protein Coding	38	GC03M170086	1.438625
LIN7C	Lin-7 Homolog C, Crumbs Cell Polarity Complex Component	Protein Coding	38	GC11M027494	1.434341
NCKAP1L	NCK Associated Protein 1 Like	Protein Coding	38	GC12P054497	1.430686
SERPINA12	Serpin Family A Member 12	Protein Coding	38	GC14M095825	1.429433
ERI1	Exoribonuclease 1	Protein Coding	38	GC08P008892	1.424631
DIDO1	Death Inducer-Obliterator 1	Protein Coding	38	GC20M062877	1.42115
ATMIN	ATM Interactor	Protein Coding	38	GC16P081035	1.42065
EML4	EMAP Like 4	Protein Coding	38	GC02P042169	1.420025
KLHL13	Kelch Like Family Member 13	Protein Coding	38	GC0XM117897	1.415081
UBXN6	UBX Domain Protein 6	Protein Coding	38	GC19M004444	1.410908
RD3	RD3 Regulator Of GUCY2D	Protein Coding	38	GC01M211476	1.406819
RFFL	Ring Finger And FYVE Like Domain Containing E3 Ubiquitin Protein Ligase	Protein Coding	38	GC17M035006	1.402697
ERAP2	Endoplasmic Reticulum Aminopeptidase 2	Protein Coding	38	GC05P096875	1.399983
CCNI	Cyclin I	Protein Coding	38	GC04M077047	1.399914
SEMA4F	Ssemaphorin 4F	Protein Coding	38	GC02P074654	1.399176
PEX11A	Peroxisomal Biogenesis Factor 11 Alpha	Protein Coding	38	GC15M089677	1.396778
U2AF2	U2 Small Nuclear RNA Auxiliary Factor 2	Protein Coding	38	GC19P055654	1.389836
THEMIS	Thymocyte Selection Associated	Protein Coding	38	GC06M127708	1.389043
IL20RA	Interleukin 20 Receptor Subunit Alpha	Protein Coding	38	GC06M136999	1.387386
DDRGK1	DDRGK Domain Containing 1	Protein Coding	38	GC20M003191	1.382816
RGS8	Regulator Of G Protein Signaling 8	Protein Coding	38	GC01M182642	1.380693
SNAPIN	SNAP Associated Protein	Protein Coding	38	GC01P153660	1.378446
TCEAL1	Transcription Elongation Factor A Like 1	Protein Coding	38	GC0XP103628	1.378421
VAX2	Ventral Anterior Homeobox 2	Protein Coding	38	GC02P070900	1.377004
DLG5	Discs Large MAGUK Scaffold Protein 5	Protein Coding	38	GC10M077790	1.373522
TUBB8	Tubulin Beta 8 Class VIII	Protein Coding	38	GC10M000048	1.371938
RAX	Retina And Anterior Neural Fold Homeobox	Protein Coding	38	GC18M059267	1.371878
NUP35	Nucleoporin 35	Protein Coding	38	GC02P183117	1.364638
NXPH1	Neurexophilin 1	Protein Coding	38	GC07P008440	1.363724
FOLR3	Folate Receptor Gamma	Protein Coding	38	GC11P072114	1.358078
GLG1	Golgi Glycoprotein 1	Protein Coding	38	GC16M074448	1.352974
GBP5	Guanylate Binding Protein 5	Protein Coding	38	GC01M089259	1.351462
PRND	Prion Like Protein Doppel	Protein Coding	38	GC20P004702	1.348959
GLCCI1	Glucocorticoid Induced 1	Protein Coding	38	GC07P007974	1.348031
ADCK1	AarF Domain Containing Kinase 1	Protein Coding	38	GC14P077800	1.34727

FGD5	FYVE, RhoGEF And PH Domain Containing 5	Protein Coding	38	GC03P014810	1.344213
CHST1	Carbohydrate Sulfotransferase 1	Protein Coding	38	GC11M045647	1.343337
SLC52A1	Solute Carrier Family 52 Member 1	Protein Coding	38	GC17M005032	1.334939
CLPTM1	CLPTM1 Regulator Of GABA Type A Receptor Forward Trafficking	Protein Coding	38	GC19P044954	1.330407
RFX1	Regulatory Factor X1	Protein Coding	38	GC19M013961	1.328761
CLYBL	Citramalyl-CoA Lyase	Protein Coding	38	GC13P099606	1.328634
GYPB	Glycophorin B (MNS Blood Group)	Protein Coding	38	GC04M143996	1.324846
RXFP4	Relaxin Family Peptide/INSL5 Receptor 4	Protein Coding	38	GC01P155959	1.319256
ITM2C	Integral Membrane Protein 2C	Protein Coding	38	GC02P230864	1.317899
JAKMIP1	Janus Kinase And Microtubule Interacting Protein 1	Protein Coding	38	GC04M006026	1.309503
RNASE2	Ribonuclease A Family Member 2	Protein Coding	38	GC14P021633	1.309497
SLC39A12	Solute Carrier Family 39 Member 12	Protein Coding	38	GC10P017951	1.308379
CADPS2	Calcium Dependent Secretion Activator 2	Protein Coding	38	GC07M122318	1.306067
SCGN	Secretagogin, EF-Hand Calcium Binding Protein	Protein Coding	38	GC06P025652	1.303936
NIP7	Nucleolar Pre-RRNA Processing Protein NIP7	Protein Coding	38	GC16P069367	1.303412
OLIG1	Oligodendrocyte Transcription Factor 1	Protein Coding	38	GC21P033070	1.302778
CCL27	C-C Motif Chemokine Ligand 27	Protein Coding	38	GC09M034662	1.29784
URI1	URI1 Prefoldin Like Chaperone	Protein Coding	38	GC19P029923	1.297276
FEZ1	Fasciculation And Elongation Protein Zeta 1	Protein Coding	38	GC11M125445	1.296743
DHX8	DEAH-Box Helicase 8	Protein Coding	38	GC17P043483	1.295377
QSOX1	Quiescin Sulfhydryl Oxidase 1	Protein Coding	38	GC01P180154	1.292331
SCIN	Scinderin	Protein Coding	38	GC07P012570	1.291139
ERRFI1	ERBB Receptor Feedback Inhibitor 1	Protein Coding	38	GC01M008004	1.28884
THAP11	THAP Domain Containing 11	Protein Coding	38	GC16P067844	1.286822
HIVEP2	HIVEP Zinc Finger 2	Protein Coding	38	GC06M142751	1.285229
KCNG4	Potassium Voltage-Gated Channel Modifier Subfamily G Member 4	Protein Coding	38	GC16M084255	1.283629
MXD1	MAX Dimerization Protein 1	Protein Coding	38	GC02P069897	1.283047
SLC39A11	Solute Carrier Family 39 Member 11	Protein Coding	38	GC17M072645	1.28195
CHAMP1	Chromosome Alignment Maintaining Phosphoprotein 1	Protein Coding	38	GC13P114314	1.280449
SETD3	SET Domain Containing 3, Actin Histidine Methyltransferase	Protein Coding	38	GC14M099397	1.279475
CAPRIN1	Cell Cycle Associated Protein 1	Protein Coding	38	GC11P034051	1.278319
ZDHHC7	Zinc Finger DHHC-Type Palmitoyltransferase 7	Protein Coding	38	GC16M084975	1.276282
RBM39	RNA Binding Motif Protein 39	Protein Coding	38	GC20M035703	1.274518
ZFAND3	Zinc Finger AN1-Type Containing 3	Protein Coding	38	GC06P055364	1.274026
RNF185	Ring Finger Protein 185	Protein Coding	38	GC22P031160	1.273238
HMG20A	High Mobility Group 20A	Protein Coding	38	GC15P077420	1.272277
CKAP4	Cytoskeleton Associated Protein 4	Protein Coding	38	GC12M106237	1.269501
NTAN1	N-Terminal Asparagine Amidase	Protein Coding	38	GC16M015037	1.261559
ACYP1	Acylphosphatase 1	Protein Coding	38	GC14M075053	1.260136
SPIN1	Spindlin 1	Protein Coding	38	GC09P088388	1.259897
BABAM1	BRISC And BRCA1 A Complex Member 1	Protein Coding	38	GC19P026636	1.252258
GLIPR2	GLI Pathogenesis Related 2	Protein Coding	38	GC09P036137	1.251413
TSPAN6	Tetraspanin 6	Protein Coding	38	GC0XM100627	1.249514
CHST2	Carbohydrate Sulfotransferase 2	Protein Coding	38	GC03P143119	1.246828
ENTPD7	Ectonucleoside Triphosphate Diphosphohydrolase 7	Protein Coding	38	GC10P099659	1.239708
FHL5	Four And A Half LIM Domains 5	Protein Coding	38	GC06P096562	1.239209
NOB1	NIN1 (RPN12) Binding Protein 1 Homolog	Protein Coding	38	GC16M069747	1.229406
KIF2B	Kinesin Family Member 2B	Protein Coding	38	GC17P053822	1.222254
ENPP5	Ectonucleotide Pyrophosphatase/Phosphodiesterase Family Member 5	Protein Coding	38	GC06M046159	1.221977
FCRLA	Fc Receptor Like A	Protein Coding	38	GC01P161708	1.220814
MAB21L1	Mab-21 Like 1	Protein Coding	38	GC13M035473	1.22036
SUPT16H	SPT16 Homolog, Facilitates Chromatin Remodeling Subunit	Protein Coding	38	GC14M021351	1.218832
AJUBA	Ajuba LIM Protein	Protein Coding	38	GC14M022971	1.215682

XPO6	Exportin 6	Protein Coding	38	GC16M028097	1.212859
GSPT2	G1 To S Phase Transition 2	Protein Coding	38	GC0XP051743	1.209181
ASCC3	Activating Signal Cointegrator 1 Complex Subunit 3	Protein Coding	38	GC06M100508	1.207211
CTR9	CTR9 Homolog, Paf1/RNA Polymerase II Complex Component	Protein Coding	38	GC11P010772	1.205783
CDH22	Cadherin 22	Protein Coding	38	GC20M046173	1.204497
GBP6	Guanylate Binding Protein Family Member 6	Protein Coding	38	GC01P089363	1.20182
GLRX2	Glutaredoxin 2	Protein Coding	38	GC01M193065	1.201168
CCL25	C-C Motif Chemokine Ligand 25	Protein Coding	38	GC19P008052	1.200507
TAF5	TATA-Box Binding Protein Associated Factor 5	Protein Coding	38	GC10P103368	1.197443
IPO11	Importin 11	Protein Coding	38	GC05P062403	1.196272
LSAMP	Limbic System Associated Membrane Protein	Protein Coding	38	GC03M115802	1.192803
VGF	VGF Nerve Growth Factor Inducible	Protein Coding	38	GC07M101162	1.18686
SRRM1	Serine And Arginine Repetitive Matrix 1	Protein Coding	38	GC01P024631	1.185542
COPS2	COP9 Signalosome Subunit 2	Protein Coding	38	GC15M049106	1.180198
ABI3	ABI Family Member 3	Protein Coding	38	GC17P049210	1.177429
SH2B2	SH2B Adaptor Protein 2	Protein Coding	38	GC07P102286	1.17496
IPPK	Inositol-Pentakisphosphate 2-Kinase	Protein Coding	38	GC09M092613	1.172119
RNF25	Ring Finger Protein 25	Protein Coding	38	GC02M218663	1.167648
ADAMTS16	ADAM Metallopeptidase With Thrombospondin Type 1 Motif 16	Protein Coding	38	GC05P005140	1.166147
CNNM3	Cyclin And CBS Domain Divalent Metal Cation Transport Mediator 3	Protein Coding	38	GC02P096815	1.158478
GPM6B	Glycoprotein M6B	Protein Coding	38	GC0XM013789	1.155788
DDX39A	DExD-Box Helicase 39A	Protein Coding	38	GC19M014408	1.154835
ENAM	Enamelin	Protein Coding	38	GC04P070628	1.154751
SENP6	SUMO Specific Peptidase 6	Protein Coding	38	GC06P075601	1.141295
FHOD1	Formin Homology 2 Domain Containing 1	Protein Coding	38	GC16M067230	1.134483
SIN3B	SIN3 Transcription Regulator Family Member B	Protein Coding	38	GC19P016829	1.12934
KIN	Kin17 DNA And RNA Binding Protein	Protein Coding	38	GC10M007750	1.12641
OXER1	Oxoeicosanoid Receptor 1	Protein Coding	38	GC02M042762	1.115681
IL25	Interleukin 25	Protein Coding	38	GC14P026307	1.113322
CHMP7	Charged Multivesicular Body Protein 7	Protein Coding	38	GC08P023243	1.11287
RAP2B	RAP2B, Member Of RAS Oncogene Family	Protein Coding	38	GC03P153162	1.111151
SYT14	Synaptotagmin 14	Protein Coding	38	GC01P209900	1.110502
CLEC11A	C-Type Lectin Domain Containing 11A	Protein Coding	38	GC19P050723	1.108385
FBXO38	F-Box Protein 38	Protein Coding	38	GC05P148383	1.108207
LMAN2L	Lectin, Mannose Binding 2 Like	Protein Coding	38	GC02M096706	1.106204
FCRL5	Fc Receptor Like 5	Protein Coding	38	GC01M157514	1.10615
SLC43A2	Solute Carrier Family 43 Member 2	Protein Coding	38	GC17M001573	1.105604
EBNA1BP2	EBNA1 Binding Protein 2	Protein Coding	38	GC01M043165	1.105337
SLC38A5	Solute Carrier Family 38 Member 5	Protein Coding	38	GC0XM048458	1.103899
CHP1	Calcineurin Like EF-Hand Protein 1	Protein Coding	38	GC15P041230	1.101093
SOX13	SRY-Box Transcription Factor 13	Protein Coding	38	GC01P204074	1.100566
ARID4A	AT-Rich Interaction Domain 4A	Protein Coding	38	GC14P058298	1.100149
ULBP1	UL16 Binding Protein 1	Protein Coding	38	GC06P149963	1.096383
XCL1	X-C Motif Chemokine Ligand 1	Protein Coding	38	GC01P168576	1.096345
STAU1	Staufen Double-Stranded RNA Binding Protein 1	Protein Coding	38	GC20M049113	1.095409
GBP4	Guanylate Binding Protein 4	Protein Coding	38	GC01M089181	1.093943
THADA	THADA Armadillo Repeat Containing	Protein Coding	38	GC02M043193	1.089677
IPO13	Importin 13	Protein Coding	38	GC01P043947	1.085432
OSBP2	Oxysterol Binding Protein 2	Protein Coding	38	GC22P030693	1.085142
MT1A	Metallothionein 1A	Protein Coding	38	GC16P056638	1.084485
SDF4	Stromal Cell Derived Factor 4	Protein Coding	38	GC01M001216	1.083048
ATXN2L	Ataxin 2 Like	Protein Coding	38	GC16P032257	1.082656
SLC26A11	Solute Carrier Family 26 Member 11	Protein Coding	38	GC17P080219	1.080336

DDX54	DEAD-Box Helicase 54	Protein Coding	38	GC12M113157	1.077462
MS4A6A	Membrane Spanning 4-Domains A6A	Protein Coding	38	GC11M069149	1.073482
ZNF133	Zinc Finger Protein 133	Protein Coding	38	GC20P018288	1.073349
PSMA8	Proteasome 20S Subunit Alpha 8	Protein Coding	38	GC18P026133	1.0702
NAP1L4	Nucleosome Assembly Protein 1 Like 4	Protein Coding	38	GC11M002944	1.06511
OAZ1	Ornithine Decarboxylase Antizyme 1	Protein Coding	38	GC19P002269	1.06476
EXOSC1	Exosome Component 1	Protein Coding	38	GC10M097435	1.061748
ELP2	Elongator Acetyltransferase Complex Subunit 2	Protein Coding	38	GC18P036129	1.06079
CEBPG	CCAAT Enhancer Binding Protein Gamma	Protein Coding	38	GC19P033373	1.059745
WDR3	WD Repeat Domain 3	Protein Coding	38	GC01P117929	1.055343
IVL	Involucrin	Protein Coding	38	GC01P152881	1.054877
FBXO8	F-Box Protein 8	Protein Coding	38	GC04M174236	1.054132
CARD6	Caspase Recruitment Domain Family Member 6	Protein Coding	38	GC05P040841	1.050229
CCR10	C-C Motif Chemokine Receptor 10	Protein Coding	38	GC17M042678	1.04757
INTS6	Integrator Complex Subunit 6	Protein Coding	38	GC13M051354	1.047019
CCL1	C-C Motif Chemokine Ligand 1	Protein Coding	38	GC17M034418	1.044601
CDC42BPG	CDC42 Binding Protein Kinase Gamma	Protein Coding	38	GC11M064823	1.044119
NLRP6	NLR Family Pyrin Domain Containing 6	Protein Coding	38	GC11P000269	1.043139
ROGDI	Rogdi Atypical Leucine Zipper	Protein Coding	38	GC16M004796	1.040412
AQP6	Aquaporin 6	Protein Coding	38	GC12P049967	1.035787
STXBP4	Syntaxin Binding Protein 4	Protein Coding	38	GC17P054968	1.029569
LHX6	LIM Homeobox 6	Protein Coding	38	GC09M122202	1.023819
SNRPB2	Small Nuclear Ribonucleoprotein Polypeptide B2	Protein Coding	38	GC20P016730	1.023241
STRN4	Striatin 4	Protein Coding	38	GC19M046719	1.022666
NUB1	Negative Regulator Of Ubiquitin Like Proteins 1	Protein Coding	38	GC07P151341	1.020728
COMTD1	Catechol-O-Methyltransferase Domain Containing 1	Protein Coding	38	GC10M075233	1.020563
ZDHHC13	Zinc Finger DHHC-Type Palmitoyltransferase 13	Protein Coding	38	GC11P019095	1.016699
IRX2	Iroquois Homeobox 2	Protein Coding	38	GC05M002708	1.016321
EIF3C	Eukaryotic Translation Initiation Factor 3 Subunit C	Protein Coding	38	GC16P032207	1.011858
DLGAP2	DLG Associated Protein 2	Protein Coding	38	GC08P000739	1.010813
AMDHD1	Amidohydrolase Domain Containing 1	Protein Coding	38	GC12P095943	1.010619
CREB3L4	CAMP Responsive Element Binding Protein 3 Like 4	Protein Coding	38	GC01P153967	1.007919
HNRNPA0	Heterogeneous Nuclear Ribonucleoprotein A0	Protein Coding	38	GC05M137750	1.007412
TNIP2	TNFAIP3 Interacting Protein 2	Protein Coding	38	GC04M002741	1.006743
ARHGEF15	Rho Guanine Nucleotide Exchange Factor 15	Protein Coding	38	GC17P008310	1.000101
THTPA	Thiamine Triphosphatase	Protein Coding	38	GC14P026310	0.999804
ATP9B	ATPase Phospholipid Transporting 9B (Putative)	Protein Coding	38	GC18P079069	0.999591
EXOSC4	Exosome Component 4	Protein Coding	38	GC08P144079	0.997609
DGKK	Diacylglycerol Kinase Kappa	Protein Coding	38	GC0XM050365	0.996069
CBX7	Chromobox 7	Protein Coding	38	GC22M048525	0.992425
PES1	Pescadillo Ribosomal Biogenesis Factor 1	Protein Coding	38	GC22M030576	0.992039
UGGT2	UDP-Glucose Glycoprotein Glucosyltransferase 2	Protein Coding	38	GC13M095801	0.989762
DENND5A	DENN Domain Containing 5A	Protein Coding	38	GC11M009162	0.988247
DTL	Denticleless E3 Ubiquitin Protein Ligase Homolog	Protein Coding	38	GC01P212035	0.97526
S100A14	S100 Calcium Binding Protein A14	Protein Coding	38	GC01M153614	0.969508
TRIM62	Tripartite Motif Containing 62	Protein Coding	38	GC01M033147	0.958858
TYW1	TRNA-YW Synthesizing Protein 1 Homolog	Protein Coding	38	GC07P066995	0.957943
MTPAP	Mitochondrial Poly(A) Polymerase	Protein Coding	38	GC10M031114	0.957799
ACOT9	Acyl-CoA Thioesterase 9	Protein Coding	38	GC0XM023701	0.956163
RNF20	Ring Finger Protein 20	Protein Coding	38	GC09P101533	0.955984
GPX5	Glutathione Peroxidase 5	Protein Coding	38	GC06P028525	0.949548
MAGOH	Mago Homolog, Exon Junction Complex Subunit	Protein Coding	38	GC01M053226	0.948679
SYNJ2BP	Synaptojanin 2 Binding Protein	Protein Coding	38	GC14M070366	0.939924

NPTX1	Neuronal Pentraxin 1	Protein Coding	38	GC17M080466	0.929675
NSL1	NSL1 Component Of MIS12 Kinetochore Complex	Protein Coding	38	GC01M212726	0.925307
DDX42	DEAD-Box Helicase 42	Protein Coding	38	GC17P063773	0.92043
ATCAY	ATCAY Kinesin Light Chain Interacting Caytaxin	Protein Coding	38	GC19P003880	0.911633
NTPCR	Nucleoside-Triphosphatase, Cancer-Related	Protein Coding	38	GC01P232950	0.910965
DERL2	Derlin 2	Protein Coding	38	GC17M005471	0.910166
MTMR9	Myotubularin Related Protein 9	Protein Coding	38	GC08P011284	0.906573
HCAR1	Hydroxycarboxylic Acid Receptor 1	Protein Coding	38	GC12M122726	0.905378
SHANK1	SH3 And Multiple Ankyrin Repeat Domains 1	Protein Coding	38	GC19M050659	0.903565
PYGO2	Pygopus Family PHD Finger 2	Protein Coding	38	GC01M154957	0.900501
NUDT7	Nudix Hydrolase 7	Protein Coding	38	GC16P077722	0.899977
TRERF1	Transcriptional Regulating Factor 1	Protein Coding	38	GC06M042224	0.899597
COPS7A	COP9 Signalosome Subunit 7A	Protein Coding	38	GC12P011846	0.889831
CHIC2	Cysteine Rich Hydrophobic Domain 2	Protein Coding	38	GC04M053996	0.88737
OTUD4	OTU Deubiquitinase 4	Protein Coding	38	GC04M145110	0.886136
RNPS1	RNA Binding Protein With Serine Rich Domain 1	Protein Coding	38	GC16M002253	0.88526
MED4	Mediator Complex Subunit 4	Protein Coding	38	GC13M048053	0.882997
PIGM	Phosphatidylinositol Glycan Anchor Biosynthesis Class M	Protein Coding	38	GC01M160027	0.879791
ARHGAP35	Rho GTPase Activating Protein 35	Protein Coding	38	GC19P046860	0.876307
HOOK2	Hook Microtubule Tethering Protein 2	Protein Coding	38	GC19M013062	0.87453
KMT5A	Lysine Methyltransferase 5A	Protein Coding	38	GC12P123621	0.873968
UGT3A1	UDP Glycosyltransferase Family 3 Member A1	Protein Coding	38	GC05M035951	0.872761
SDR16C5	Short Chain Dehydrogenase/Reductase Family 16C Member 5	Protein Coding	38	GC08M056300	0.872724
EXOC8	Exocyst Complex Component 8	Protein Coding	38	GC01M231332	0.872508
SCYL2	SCY1 Like Pseudokinase 2	Protein Coding	38	GC12P100267	0.868968
BRMS1	BRMS1 Transcriptional Repressor And Anoikis Regulator	Protein Coding	38	GC11M069459	0.859179
DBP	D-Box Binding PAR BZIP Transcription Factor	Protein Coding	38	GC19M048630	0.858134
CPSF2	Cleavage And Polyadenylation Specific Factor 2	Protein Coding	38	GC14P092121	0.857825
PRPF19	Pre-MRNA Processing Factor 19	Protein Coding	38	GC11M060890	0.857325
PAN2	Poly(A) Specific Ribonuclease Subunit PAN2	Protein Coding	38	GC12M056316	0.854777
LCMT2	Leucine Carboxyl Methyltransferase 2	Protein Coding	38	GC15M043323	0.848274
CDHR5	Cadherin Related Family Member 5	Protein Coding	38	GC11M001088	0.848114
SIGLEC10	Sialic Acid Binding Ig Like Lectin 10	Protein Coding	38	GC19M051410	0.847002
HCAR2	Hydroxycarboxylic Acid Receptor 2	Protein Coding	38	GC12M122701	0.845475
PGP	Phosphoglycolate Phosphatase	Protein Coding	38	GC16M003074	0.845162
TIMM8B	Translocase Of Inner Mitochondrial Membrane 8 Homolog B	Protein Coding	38	GC11M112084	0.84133
PPP4R1	Protein Phosphatase 4 Regulatory Subunit 1	Protein Coding	38	GC18M009546	0.837157
P2RY8	P2Y Receptor Family Member 8	Protein Coding	38	GC0XM001462	0.836151
TUBA3C	Tubulin Alpha 3c	Protein Coding	38	GC13M019173	0.835055
IFI6	Interferon Alpha Inducible Protein 6	Protein Coding	38	GC01M027666	0.83484
STX2	Syntaxin 2	Protein Coding	38	GC12M130789	0.832254
LY6D	Lymphocyte Antigen 6 Family Member D	Protein Coding	38	GC08M142784	0.832231
MRGPRX2	MAS Related GPR Family Member X2	Protein Coding	38	GC11M019077	0.830833
TPPP3	Tubulin Polymerization Promoting Protein Family Member 3	Protein Coding	38	GC16M067389	0.830653
TBC1D15	TBC1 Domain Family Member 15	Protein Coding	38	GC12P071839	0.827142
NDFIP1	Nedd4 Family Interacting Protein 1	Protein Coding	38	GC05P142108	0.825649
MAGEA1	MAGE Family Member A1	Protein Coding	38	GC0XP153179	0.820414
COPS7B	COP9 Signalosome Subunit 7B	Protein Coding	38	GC02P231781	0.818472
SCG5	Secretogranin V	Protein Coding	38	GC15P032641	0.813643
BCL2L14	BCL2 Like 14	Protein Coding	38	GC12P012049	0.81328
TAF12	TATA-Box Binding Protein Associated Factor 12	Protein Coding	38	GC01M028683	0.804841
TRO	Trophinin	Protein Coding	38	GC0XP054920	0.7972
MED7	Mediator Complex Subunit 7	Protein Coding	38	GC05M157137	0.795577

HINT2	Histidine Triad Nucleotide Binding Protein 2	Protein Coding	38	GC09M035812	0.79483
FBXO5	F-Box Protein 5	Protein Coding	38	GC06M152970	0.78462
SUPT6H	SPT6 Homolog, Histone Chaperone And Transcription Elongation Factor	Protein Coding	38	GC17P028662	0.780913
MPC2	Mitochondrial Pyruvate Carrier 2	Protein Coding	38	GC01M167916	0.779749
THRSP	Thyroid Hormone Responsive	Protein Coding	38	GC11P078063	0.778513
TSPAN1	Tetraspanin 1	Protein Coding	38	GC01P046175	0.777596
ZW10	Zw10 Kinetochore Protein	Protein Coding	38	GC11M113733	0.77732
PLEKHH2	Pleckstrin Homology, MyTH4 And FERM Domain Containing H2	Protein Coding	38	GC02P043600	0.774854
PMEL	Premelanosome Protein	Protein Coding	38	GC12M055954	0.774854
FAM3D	FAM3 Metabolism Regulating Signaling Molecule D	Protein Coding	38	GC03M058633	0.771904
ADO	2-Aminoethanethiol Dioxygenase	Protein Coding	38	GC10P062804	0.766435
NUP54	Nucleoporin 54	Protein Coding	38	GC04M076114	0.763963
TUBD1	Tubulin Delta 1	Protein Coding	38	GC17M059859	0.760483
OSBPL8	Oxysterol Binding Protein Like 8	Protein Coding	38	GC12M076354	0.748165
TGM6	Transglutaminase 6	Protein Coding	38	GC20P002380	0.745927
ADGRE2	Adhesion G Protein-Coupled Receptor E2	Protein Coding	38	GC19M014733	0.744092
IMPG2	Interphotoreceptor Matrix Proteoglycan 2	Protein Coding	38	GC03M101222	0.739799
GRSF1	G-Rich RNA Sequence Binding Factor 1	Protein Coding	38	GC04M070815	0.739638
CBX8	Chromobox 8	Protein Coding	38	GC17M079794	0.739428
GDF10	Growth Differentiation Factor 10	Protein Coding	38	GC10P047300	0.737441
MOB1B	MOB Kinase Activator 1B	Protein Coding	38	GC04P070902	0.735013
GIMAP4	GTPase, IMAP Family Member 4	Protein Coding	38	GC07P150568	0.734298
PGLYRP4	Peptidoglycan Recognition Protein 4	Protein Coding	38	GC01M153327	0.733315
SRSF11	Serine And Arginine Rich Splicing Factor 11	Protein Coding	38	GC01P070206	0.73143
CSTF3	Cleavage Stimulation Factor Subunit 3	Protein Coding	38	GC11M033077	0.729733
XPO4	Exportin 4	Protein Coding	38	GC13M020777	0.725556
TSPAN31	Tetraspanin 31	Protein Coding	38	GC12P057738	0.725296
GUF1	GTP Binding Elongation Factor GUF1	Protein Coding	38	GC04P044680	0.712482
ARL8B	ADP Ribosylation Factor Like GTPase 8B	Protein Coding	38	GC03P005122	0.711501
XAF1	XIAP Associated Factor 1	Protein Coding	38	GC17P006757	0.711501
ZC3HC1	Zinc Finger C3HC-Type Containing 1	Protein Coding	38	GC07M130026	0.708781
GOLGA7	Golgin A7	Protein Coding	38	GC08P041467	0.705367
NOC2L	NOC2 Like Nucleolar Associated Transcriptional Repressor	Protein Coding	38	GC01M002649	0.70213
SF3A3	Splicing Factor 3a Subunit 3	Protein Coding	38	GC01M037956	0.701477
LMAN2	Lectin, Mannose Binding 2	Protein Coding	38	GC05M177493	0.697993
APOBEC3F	Apolipoprotein B MRNA Editing Enzyme Catalytic Subunit 3F	Protein Coding	38	GC22P039030	0.694162
CERS4	Ceramide Synthase 4	Protein Coding	38	GC19P008206	0.692771
RNF43	Ring Finger Protein 43	Protein Coding	38	GC17M058352	0.691541
SECTM1	Secreted And Transmembrane 1	Protein Coding	38	GC17M082321	0.691541
YEATS4	YEATS Domain Containing 4	Protein Coding	38	GC12P069359	0.687927
INHBE	Inhibin Subunit Beta E	Protein Coding	38	GC12P057452	0.686946
ESYT1	Extended Synaptotagmin 1	Protein Coding	38	GC12P056379	0.68553
MRS2	Magnesium Transporter MRS2	Protein Coding	38	GC06P024402	0.684884
ASB1	Ankyrin Repeat And SOCS Box Containing 1	Protein Coding	38	GC02P238426	0.683832
CDKL2	Cyclin Dependent Kinase Like 2	Protein Coding	38	GC04M075576	0.683441
MRPL28	Mitochondrial Ribosomal Protein L28	Protein Coding	38	GC16M000357	0.682305
RABIF	RAB Interacting Factor	Protein Coding	38	GC01M202878	0.681549
TNS4	Tensin 4	Protein Coding	38	GC17M040475	0.68056
ILDR1	Immunoglobulin Like Domain Containing Receptor 1	Protein Coding	38	GC03M121987	0.679634
LRP3	LDL Receptor Related Protein 3	Protein Coding	38	GC19P033195	0.679104
EIF5B	Eukaryotic Translation Initiation Factor 5B	Protein Coding	38	GC02P099320	0.677164
TPTE	Transmembrane Phosphatase With Tensin Homology	Protein Coding	38	GC21P010521	0.674341
IL36G	Interleukin 36 Gamma	Protein Coding	38	GC02P112973	0.666772

GSTA5	Glutathione S-Transferase Alpha 5	Protein Coding	38	GC06M052831	0.663409
KRT6C	Keratin 6C	Protein Coding	38	GC12M052468	0.661723
TADA3	Transcriptional Adaptor 3	Protein Coding	38	GC03M009779	0.660631
LRRC4	Leucine Rich Repeat Containing 4	Protein Coding	38	GC07M128027	0.655577
ORC3	Origin Recognition Complex Subunit 3	Protein Coding	38	GC06P087590	0.65114
EIF1	Eukaryotic Translation Initiation Factor 1	Protein Coding	38	GC17P041688	0.646513
ZFX	Zinc Finger Protein X-Linked	Protein Coding	38	GC0XP024148	0.643505
SIGLEC9	Sialic Acid Binding Ig Like Lectin 9	Protein Coding	38	GC19P051124	0.643257
SCAMP3	Secretory Carrier Membrane Protein 3	Protein Coding	38	GC01M155255	0.641233
MRPL46	Mitochondrial Ribosomal Protein L46	Protein Coding	38	GC15M088466	0.634632
RPL17	Ribosomal Protein L17	Protein Coding	38	GC18M049488	0.629364
PARP12	Poly(ADP-Ribose) Polymerase Family Member 12	Protein Coding	38	GC07M140023	0.62447
GMFG	Glia Maturation Factor Gamma	Protein Coding	38	GC19M039328	0.605745
HSD17B14	Hydroxysteroid 17-Beta Dehydrogenase 14	Protein Coding	38	GC19M048813	0.600728
AP2A2	Adaptor Related Protein Complex 2 Subunit Alpha 2	Protein Coding	38	GC11P000924	0.596669
IMP3	IMP U3 Small Nucleolar Ribonucleoprotein 3	Protein Coding	38	GC15M075639	0.577311
USP39	Ubiquitin Specific Peptidase 39	Protein Coding	38	GC02P085637	0.577311
SAMM50	SAMM50 Sorting And Assembly Machinery Component	Protein Coding	38	GC22P043955	0.577026
RBM7	RNA Binding Motif Protein 7	Protein Coding	38	GC11P114401	0.576147
KIR3DL2	Killer Cell Immunoglobulin Like Receptor, Three Ig Domains And Long Cytoplasmic Tail 2	Protein Coding	38	GC19P056451	0.572813
ANAPC7	Anaphase Promoting Complex Subunit 7	Protein Coding	38	GC12M110372	0.572813
NOXA1	NADPH Oxidase Activator 1	Protein Coding	38	GC09P137423	0.568537
ADAM33	ADAM Metallopeptidase Domain 33	Protein Coding	38	GC20M003596	0.56001
OXGR1	Oxoglutarate Receptor 1	Protein Coding	38	GC13M096985	0.557382
BCL2L10	BCL2 Like 10	Protein Coding	38	GC15M067449	0.554982
RANBP17	RAN Binding Protein 17	Protein Coding	38	GC05P170861	0.554982
CDK11B	Cyclin Dependent Kinase 11B	Protein Coding	38	GC01M002721	0.551905
MUC6	Mucin 6, Oligomeric Mucus/Gel-Forming	Protein Coding	38	GC11M001012	0.540426
CATSPER4	Cation Channel Sperm Associated 4	Protein Coding	38	GC01P026201	0.537362
ARC	Activity Regulated Cytoskeleton Associated Protein	Protein Coding	38	GC08M142611	0.533219
DDX47	DEAD-Box Helicase 47	Protein Coding	38	GC12P012826	0.531273
BCAS2	BCAS2 Pre-MRNA Processing Factor	Protein Coding	38	GC01M114567	0.527828
CUEDC2	CUE Domain Containing 2	Protein Coding	38	GC10M102424	0.481928
NAPB	NSF Attachment Protein Beta	Protein Coding	38	GC20M023374	0.481928
EXPH5	Exophilin 5	Protein Coding	38	GC11M108505	0.47794
TOR1B	Torsin Family 1 Member B	Protein Coding	38	GC09P129803	0.47794
SOX21	SRY-Box Transcription Factor 21	Protein Coding	38	GC13M094709	0.47794
NECTIN4	Nectin Cell Adhesion Molecule 4	Protein Coding	38	GC01M161071	0.47794
INSL5	Insulin Like 5	Protein Coding	38	GC01M066797	0.457159
BTN2A1	Butyrophilin Subfamily 2 Member A1	Protein Coding	38	GC06P026457	0.443525
SEZ6L2	Seizure Related 6 Homolog Like 2	Protein Coding	38	GC16M031163	0.443441
SRPRB	SRP Receptor Subunit Beta	Protein Coding	38	GC03P133784	0.436642
DEFA5	Defensin Alpha 5	Protein Coding	38	GC08M007057	0.425762
HOXB3	Homeobox B3	Protein Coding	38	GC17M048548	0.425762
SPON2	Spondin 2	Protein Coding	38	GC04M001166	0.391536
HAO2	Hydroxyacid Oxidase 2	Protein Coding	38	GC01P119368	0.391115
SNF8	SNF8 Subunit Of ESCRT-II	Protein Coding	38	GC17M048929	0.359139
TREML2	Triggering Receptor Expressed On Myeloid Cells Like 2	Protein Coding	38	GC06M047083	0.333456
SPAG5	Sperm Associated Antigen 5	Protein Coding	38	GC17M028577	0.298286
PIP	Prolactin Induced Protein	Protein Coding	38	GC07P143132	0.290578
IFNA5	Interferon Alpha 5	Protein Coding	38	GC09M021304	0.272315
PRAME	PRAME Nuclear Receptor Transcriptional Regulator	Protein Coding	38	GC22M022547	0.236781
ERN2	Endoplasmic Reticulum To Nucleus Signaling 2	Protein Coding	38	GC16M023690	0.191922

INHBC	Inhibin Subunit Beta C	Protein Coding	38	GC12P057434	0.172975
DNAH1	Dynein Axonemal Heavy Chain 1	Protein Coding	37	GC03P052314	40.51577
NKX2-6	NK2 Homeobox 6	Protein Coding	37	GC08M023702	35.63402
GDF1	Growth Differentiation Factor 1	Protein Coding	37	GC19M018843	34.27211
RINT1	RAD50 Interactor 1	Protein Coding	37	GC07P105532	21.52678
WDPCP	WD Repeat Containing Planar Cell Polarity Effector	Protein Coding	37	GC02M063121	20.87762
LARS1	Leucyl-TRNA Synthetase 1	Protein Coding	37	GC05M146114	19.29784
CFC1	Cripto, FRL-1, Cryptic Family 1	Protein Coding	37	GC02M130592	15.69469
FIGLA	Folliculogenesis Specific BHLH Transcription Factor	Protein Coding	37	GC02M070741	15.52475
APLN	Apelin	Protein Coding	37	GC0XM129645	14.83912
SUN5	Sad1 And UNC84 Domain Containing 5	Protein Coding	37	GC20M032983	12.90633
HYDIN	HYDIN Axonemal Central Pair Apparatus Protein	Protein Coding	37	GC16M070849	12.47148
EPG5	Ectopic P-Granules Autophagy Protein 5 Homolog	Protein Coding	37	GC18M045800	12.43397
DNAH17	Dynein Axonemal Heavy Chain 17	Protein Coding	37	GC17M078423	12.34229
ATP5F1A	ATP Synthase F1 Subunit Alpha	Protein Coding	37	GC18M046081	12.33098
HRC	Histidine Rich Calcium Binding Protein	Protein Coding	37	GC19M049151	11.92581
TEX11	Testis Expressed 11	Protein Coding	37	GC0XM070512	11.73335
PMFBP1	Polyamine Modulated Factor 1 Binding Protein 1	Protein Coding	37	GC16M072112	11.58751
NUP188	Nucleoporin 188	Protein Coding	37	GC09P128947	11.41923
IFT80	Intraflagellar Transport 80	Protein Coding	37	GC03M160256	11.29587
IFT43	Intraflagellar Transport 43	Protein Coding	37	GC14P075902	11.08651
USP9Y	Ubiquitin Specific Peptidase 9 Y-Linked	Protein Coding	37	GC0YP012546	10.51155
DNAH2	Dynein Axonemal Heavy Chain 2	Protein Coding	37	GC17P007717	10.23056
FOXE3	Forkhead Box E3	Protein Coding	37	GC01P047416	10.04901
TMEM231	Transmembrane Protein 231	Protein Coding	37	GC16M075536	10.02417
MESP1	Mesoderm Posterior BHLH Transcription Factor 1	Protein Coding	37	GC15M089748	9.709144
NELFA	Negative Elongation Factor Complex Member A	Protein Coding	37	GC04M001985	9.394647
GUCY1A1	Guanylate Cyclase 1 Soluble Subunit Alpha 1	Protein Coding	37	GC04P155667	9.265461
KLHL40	Kelch Like Family Member 40	Protein Coding	37	GC03P042685	9.230223
TECRL	Trans-2,3-Enoyl-CoA Reductase Like	Protein Coding	37	GC04M064275	9.172943
RMND1	Required For Meiotic Nuclear Division 1 Homolog	Protein Coding	37	GC06M151404	9.158224
RANGRF	RAN Guanine Nucleotide Release Factor	Protein Coding	37	GC17P008288	9.044003
CCDC40	Coiled-Coil Domain Containing 40	Protein Coding	37	GC17P080037	8.889267
DZIP1	DAZ Interacting Zinc Finger Protein 1	Protein Coding	37	GC13M095578	8.882645
INTU	Inturned Planar Cell Polarity Protein	Protein Coding	37	GC04P127623	8.715021
MEGF8	Multiple EGF Like Domains 8	Protein Coding	37	GC19P042325	8.524505
NEK10	NIMA Related Kinase 10	Protein Coding	37	GC03M027128	8.521601
DNAAF1	Dynein Axonemal Assembly Factor 1	Protein Coding	37	GC16P084145	8.520301
IFT27	Intraflagellar Transport 27	Protein Coding	37	GC22M036759	8.512031
FHOD3	Formin Homology 2 Domain Containing 3	Protein Coding	37	GC18P036297	8.444327
B9D1	B9 Domain Containing 1	Protein Coding	37	GC17M019334	8.407229
TSR2	TSR2 Ribosome Maturation Factor	Protein Coding	37	GC0XP054441	8.323228
NDUFAF5	NADH:Ubiquinone Oxidoreductase Complex Assembly Factor 5	Protein Coding	37	GC20P013806	8.1383
HARS1	Histidyl-TRNA Synthetase 1	Protein Coding	37	GC05M140673	7.835157
MRAP	Melanocortin 2 Receptor Accessory Protein	Protein Coding	37	GC21P032291	7.614647
TCTN1	Tectonic Family Member 1	Protein Coding	37	GC12P110614	7.579936
EPRS1	Glutamyl-Prolyl-TRNA Synthetase 1	Protein Coding	37	GC01M219969	7.566165
TMEM237	Transmembrane Protein 237	Protein Coding	37	GC02M201620	7.484797
MYOZ1	Myozenin 1	Protein Coding	37	GC10M073631	7.439993
DNAAF2	Dynein Axonemal Assembly Factor 2	Protein Coding	37	GC14M049625	7.354517
BICC1	BicC Family RNA Binding Protein 1	Protein Coding	37	GC10P058513	7.317528
KCNA7	Potassium Voltage-Gated Channel Subfamily A Member 7	Protein Coding	37	GC19M049067	7.212942
MRPS34	Mitochondrial Ribosomal Protein S34	Protein Coding	37	GC16M001771	7.198709

HES7	Hes Family BHLH Transcription Factor 7	Protein Coding	37	GC17M008120	7.186095
ANKRD2	Ankyrin Repeat Domain 2	Protein Coding	37	GC10P097572	7.168242
KIAA0319L	KIAA0319 Like	Protein Coding	37	GC01M035433	7.04331
COG1	Component Of Oligomeric Golgi Complex 1	Protein Coding	37	GC17P073193	7.011631
MAGEL2	MAGE Family Member L2	Protein Coding	37	GC15M023643	6.937697
TMEM107	Transmembrane Protein 107	Protein Coding	37	GC17M009197	6.877822
TIMMDC1	Translocase Of Inner Mitochondrial Membrane Domain Containing 1	Protein Coding	37	GC03P119498	6.835277
DZIP1L	DAZ Interacting Zinc Finger Protein 1 Like	Protein Coding	37	GC03M138061	6.784328
NAV1	Neuron Navigator 1	Protein Coding	37	GC01P201547	6.691055
AARS1	Alanyl-TRNA Synthetase 1	Protein Coding	37	GC16M070343	6.629658
TGDS	TDP-Glucose 4,6-Dehydratase	Protein Coding	37	GC13M094574	6.402716
AMER1	APC Membrane Recruitment Protein 1	Protein Coding	37	GC0XM064185	6.361203
ITGB1BP2	Integrin Subunit Beta 1 Binding Protein 2	Protein Coding	37	GC0XP071302	6.309895
MUC16	Mucin 16, Cell Surface Associated	Protein Coding	37	GC19M008848	6.273042
COX7A1	Cytochrome C Oxidase Subunit 7A1	Protein Coding	37	GC19M047084	6.259264
TRIM55	Tripartite Motif Containing 55	Protein Coding	37	GC08P066225	6.252094
RLN2	Relaxin 2	Protein Coding	37	GC09M005306	6.144354
KCNE4	Potassium Voltage-Gated Channel Subfamily E Regulatory Subunit 4	Protein Coding	37	GC02P223051	6.139114
ADAMTS19	ADAM Metallopeptidase With Thrombospondin Type 1 Motif 19	Protein Coding	37	GC05P129460	6.109543
TMEM138	Transmembrane Protein 138	Protein Coding	37	GC11P061362	6.083316
PTCD3	Pentatricopeptide Repeat Domain 3	Protein Coding	37	GC02P086106	6.061646
LYRM7	LYR Motif Containing 7	Protein Coding	37	GC05P131170	5.887405
ASTN2	Astrotactin 2	Protein Coding	37	GC09M116425	5.803585
FAN1	FANCD2 And FANCI Associated Nuclease 1	Protein Coding	37	GC15P032043	5.744034
GATB	Glutamyl-TRNA Amidotransferase Subunit B	Protein Coding	37	GC04M151670	5.599076
HOPX	HOP Homeobox	Protein Coding	37	GC04M056647	5.497233
GARS1	Glycyl-TRNA Synthetase 1	Protein Coding	37	GC07P030580	5.48901
HMCN1	Hemicentin 1	Protein Coding	37	GC01P185734	5.416734
TRIM54	Tripartite Motif Containing 54	Protein Coding	37	GC02P027289	5.366031
CASZ1	Castor Zinc Finger 1	Protein Coding	37	GC01M010642	5.314163
MBD5	Methyl-CpG Binding Domain Protein 5	Protein Coding	37	GC02P148021	5.292869
SYNM	Synemin	Protein Coding	37	GC15P099098	5.045196
CCDC28B	Coiled-Coil Domain Containing 28B	Protein Coding	37	GC01P032200	4.939856
DPM2	Dolichyl-Phosphate Mannosyltransferase Subunit 2, Regulatory	Protein Coding	37	GC09M127935	4.937171
TRAPPC2L	Trafficking Protein Particle Complex Subunit 2L	Protein Coding	37	GC16P088856	4.827066
IFT74	Intraflagellar Transport 74	Protein Coding	37	GC09P026947	4.771288
USB1	U6 SnRNA Biogenesis Phosphodiesterase 1	Protein Coding	37	GC16P057999	4.757728
MMEL1	Membrane Metalloendopeptidase Like 1	Protein Coding	37	GC01M002590	4.733079
CH25H	Cholesterol 25-Hydroxylase	Protein Coding	37	GC10M089205	4.696079
ODF1	Outer Dense Fiber Of Sperm Tails 1	Protein Coding	37	GC08P102551	4.694699
FAM111A	FAM111 Trypsin Like Peptidase A	Protein Coding	37	GC11P059142	4.622735
TXNDC15	Thioredoxin Domain Containing 15	Protein Coding	37	GC05P134873	4.592937
DPPA2	Developmental Pluripotency Associated 2	Protein Coding	37	GC03M109293	4.557045
NAALADL2	N-Acetylated Alpha-Linked Acidic Dipeptidase Like 2	Protein Coding	37	GC03P174438	4.475307
SMPX	Small Muscle Protein X-Linked	Protein Coding	37	GC0XM021706	4.366266
C12orf57	Chromosome 12 Open Reading Frame 57	Protein Coding	37	GC12P011870	4.352124
RFXAP	Regulatory Factor X Associated Protein	Protein Coding	37	GC13P036819	4.321361
THSD4	Thrombospondin Type 1 Domain Containing 4	Protein Coding	37	GC15P071096	4.314867
WDR72	WD Repeat Domain 72	Protein Coding	37	GC15M068731	4.283144
PAM16	Presequence Translocase Associated Motor 16	Protein Coding	37	GC16M004332	4.159547
KIF15	Kinesin Family Member 15	Protein Coding	37	GC03P045589	4.147649
CKAP2L	Cytoskeleton Associated Protein 2 Like	Protein Coding	37	GC02M112736	4.10223
RNF207	Ring Finger Protein 207	Protein Coding	37	GC01P006205	4.068367

MCU	Mitochondrial Calcium Uniporter	Protein Coding	37	GC10P072692	4.039841
YARS1	Tyrosyl-TRNA Synthetase 1	Protein Coding	37	GC01M032776	4.038589
TARS1	Threonyl-TRNA Synthetase 1	Protein Coding	37	GC05P033441	4.015557
GNB1L	G Protein Subunit Beta 1 Like	Protein Coding	37	GC22M019783	4.009463
GPR182	G Protein-Coupled Receptor 182	Protein Coding	37	GC12P056994	3.992162
ARV1	ARV1 Homolog, Fatty Acid Homeostasis Modulator	Protein Coding	37	GC01P230978	3.986987
RNASEH2B	Ribonuclease H2 Subunit B	Protein Coding	37	GC13P050909	3.976773
CD52	CD52 Molecule	Protein Coding	37	GC01P026317	3.964773
MFSD8	Major Facilitator Superfamily Domain Containing 8	Protein Coding	37	GC04M127917	3.901806
TMTC1	Transmembrane O-Mannosyltransferase Targeting Cadherins 1	Protein Coding	37	GC12M029500	3.888387
VPS54	VPS54 Subunit Of GARP Complex	Protein Coding	37	GC02M063892	3.861558
SHROOM3	Shroom Family Member 3	Protein Coding	37	GC04P076435	3.805844
MED12L	Mediator Complex Subunit 12L	Protein Coding	37	GC03P151085	3.792529
FAM50A	Family With Sequence Similarity 50 Member A	Protein Coding	37	GC0XP154445	3.776574
STAC3	SH3 And Cysteine Rich Domain 3	Protein Coding	37	GC12M057243	3.707941
PROK1	Prokineticin 1	Protein Coding	37	GC01P110451	3.628652
CNTLN	Centlein	Protein Coding	37	GC09P017124	3.588391
PIDD1	P53-Induced Death Domain Protein 1	Protein Coding	37	GC11M001141	3.588022
METTL5	Methyltransferase Like 5	Protein Coding	37	GC02M169811	3.543219
LIPI	Lipase I	Protein Coding	37	GC21M014108	3.462119
LUC7L3	LUC7 Like 3 Pre-MRNA Splicing Factor	Protein Coding	37	GC17P050719	3.461307
ARMC5	Armadillo Repeat Containing 5	Protein Coding	37	GC16P032572	3.427654
ELMOD2	ELMO Domain Containing 2	Protein Coding	37	GC04P140524	3.41486
MPLKIP	M-Phase Specific PLK1 Interacting Protein	Protein Coding	37	GC07M040126	3.398091
PHF12	PHD Finger Protein 12	Protein Coding	37	GC17M028905	3.395181
PURB	Purine Rich Element Binding Protein B	Protein Coding	37	GC07M044879	3.395086
GXYLT2	Glucoside Xylosyltransferase 2	Protein Coding	37	GC03P072848	3.390328
WBP11	WW Domain Binding Protein 11	Protein Coding	37	GC12M014784	3.358112
LUC7L2	LUC7 Like 2, Pre-MRNA Splicing Factor	Protein Coding	37	GC07P139344	3.323713
TCEA3	Transcription Elongation Factor A3	Protein Coding	37	GC01M023382	3.317545
DCAF17	DDB1 And CUL4 Associated Factor 17	Protein Coding	37	GC02P171434	3.316889
CFHR2	Complement Factor H Related 2	Protein Coding	37	GC01P196943	3.29767
GULP1	GULP PTB Domain Containing Engulfment Adaptor 1	Protein Coding	37	GC02P188291	3.293763
ACKR1	Atypical Chemokine Receptor 1 (Duffy Blood Group)	Protein Coding	37	GC01P159203	3.28479
ATG14	Autophagy Related 14	Protein Coding	37	GC14M055366	3.281351
DACH2	Dachshund Family Transcription Factor 2	Protein Coding	37	GC0XP086148	3.272121
FIGN	Fidgetin, Microtubule Severing Factor	Protein Coding	37	GC02M163593	3.265266
TMTC3	Transmembrane O-Mannosyltransferase Targeting Cadherins 3	Protein Coding	37	GC12P088142	3.264204
RNF113A	Ring Finger Protein 113A	Protein Coding	37	GC0XM119870	3.260269
C19orf12	Chromosome 19 Open Reading Frame 12	Protein Coding	37	GC19M029699	3.218226
RAD9B	RAD9 Checkpoint Clamp Component B	Protein Coding	37	GC12P110502	3.205786
SUGCT	Succinyl-CoA:Glutarate-CoA Transferase	Protein Coding	37	GC07P040134	3.156547
FEZ2	Fasciculation And Elongation Protein Zeta 2	Protein Coding	37	GC02M036519	3.132654
HLA-DQA2	Major Histocompatibility Complex, Class II, DQ Alpha 2	Protein Coding	37	GC06P032741	3.103945
GPR137B	G Protein-Coupled Receptor 137B	Protein Coding	37	GC01P236142	3.091147
LAX1	Lymphocyte Transmembrane Adaptor 1	Protein Coding	37	GC01P203768	3.080384
PI16	Peptidase Inhibitor 16	Protein Coding	37	GC06P055355	3.063711
VPS51	VPS51 Subunit Of GARP Complex	Protein Coding	37	GC11P065089	3.049909
BUD13	BUD13 Homolog	Protein Coding	37	GC11M116749	3.041792
CNTN5	Contactin 5	Protein Coding	37	GC11P099020	3.03593
SNX16	Sorting Nexin 16	Protein Coding	37	GC08M081799	3.029479
FKBPL	FKBP Prolyl Isomerase Like	Protein Coding	37	GC06M046906	3.006682
AKAP4	A-Kinase Anchoring Protein 4	Protein Coding	37	GC0XM050190	2.984419

ERAS	ES Cell Expressed Ras	Protein Coding	37	GC0XP048827	2.980674
NKAP	NFKB Activating Protein	Protein Coding	37	GC0XM119994	2.970986
KLRC4	Killer Cell Lectin Like Receptor C4	Protein Coding	37	GC12M015687	2.960657
BTBD3	BTB Domain Containing 3	Protein Coding	37	GC20P011890	2.944651
EMCN	Endomucin	Protein Coding	37	GC04M100395	2.93104
NECTIN2	Nectin Cell Adhesion Molecule 2	Protein Coding	37	GC19P044849	2.925053
STAC2	SH3 And Cysteine Rich Domain 2	Protein Coding	37	GC17M039236	2.912885
ATP13A3	ATPase 13A3	Protein Coding	37	GC03M194402	2.902083
AUP1	AUP1 Lipid Droplet Regulating VLDL Assembly Factor	Protein Coding	37	GC02M074526	2.899714
TAPT1	Transmembrane Anterior Posterior Transformation 1	Protein Coding	37	GC04M016162	2.876887
SCHIP1	Schwannomin Interacting Protein 1	Protein Coding	37	GC03P159273	2.869594
KRT74	Keratin 74	Protein Coding	37	GC12M052565	2.865534
ADPRH	ADP-Ribosylarginine Hydrolase	Protein Coding	37	GC03P119579	2.856733
EIF4ENIF1	Eukaryotic Translation Initiation Factor 4E Nuclear Import Factor 1	Protein Coding	37	GC22M031436	2.853664
POC1B	POC1 Centriolar Protein B	Protein Coding	37	GC12M089419	2.840952
RSPRY1	Ring Finger And SPRY Domain Containing 1	Protein Coding	37	GC16P057187	2.840692
SLITRK3	SLIT And NTRK Like Family Member 3	Protein Coding	37	GC03M165184	2.826707
SPO11	SPO11 Initiator Of Meiotic Double Stranded Breaks	Protein Coding	37	GC20P057329	2.818558
CCL18	C-C Motif Chemokine Ligand 18	Protein Coding	37	GC17P036064	2.813076
SAMD9L	Sterile Alpha Motif Domain Containing 9 Like	Protein Coding	37	GC07M093130	2.797845
IFT20	Intraflagellar Transport 20	Protein Coding	37	GC17M031076	2.794327
TRIM17	Tripartite Motif Containing 17	Protein Coding	37	GC01M228407	2.788845
PAF1	PAF1 Homolog, Paf1/RNA Polymerase II Complex Component	Protein Coding	37	GC19M039385	2.785974
TONSL	Tonsoku Like, DNA Repair Protein	Protein Coding	37	GC08M144428	2.784888
MED20	Mediator Complex Subunit 20	Protein Coding	37	GC06M041905	2.783226
TMEM38A	Transmembrane Protein 38A	Protein Coding	37	GC19P016661	2.775588
TLX2	T Cell Leukemia Homeobox 2	Protein Coding	37	GC02P074514	2.774603
BCO2	Beta-Carotene Oxygenase 2	Protein Coding	37	GC11P112175	2.770006
BCL7B	BAF Chromatin Remodeling Complex Subunit BCL7B	Protein Coding	37	GC07M073536	2.767446
LAMTOR5	Late Endosomal/Lysosomal Adaptor, MAPK And MTOR Activator 5	Protein Coding	37	GC01M110401	2.764493
PDS5B	PDS5 Cohesin Associated Factor B	Protein Coding	37	GC13P032586	2.762954
RHOU	Ras Homolog Family Member U	Protein Coding	37	GC01P228644	2.742556
PPRC1	PPARG Related Coactivator 1	Protein Coding	37	GC10P102136	2.737819
ERCC6L	ERCC Excision Repair 6 Like, Spindle Assembly Checkpoint Helicase	Protein Coding	37	GC0XM072204	2.734531
FRMD4A	FERM Domain Containing 4A	Protein Coding	37	GC10M013643	2.716914
PHF11	PHD Finger Protein 11	Protein Coding	37	GC13P049495	2.715304
ECD	Ecdysoneless Cell Cycle Regulator	Protein Coding	37	GC10M073130	2.709134
DPT	Dermatopontin	Protein Coding	37	GC01M168696	2.708972
ZSWIM6	Zinc Finger SWIM-Type Containing 6	Protein Coding	37	GC05P061332	2.703116
ZNF45	Zinc Finger Protein 45	Protein Coding	37	GC19M046924	2.698787
RASIP1	Ras Interacting Protein 1	Protein Coding	37	GC19M048720	2.694478
MYL5	Myosin Light Chain 5	Protein Coding	37	GC04P000673	2.693931
P3H2	Prolyl 3-Hydroxylase 2	Protein Coding	37	GC03M189956	2.673287
LAG3	Lymphocyte Activating 3	Protein Coding	37	GC12P011853	2.639895
CNTROB	Centrobin, Centriole Duplication And Spindle Assembly Protein	Protein Coding	37	GC17P007932	2.632777
GAR1	GAR1 Ribonucleoprotein	Protein Coding	37	GC04P109815	2.632667
ZNF607	Zinc Finger Protein 607	Protein Coding	37	GC19M037696	2.624465
POMGNT2	Protein O-Linked Mannose N-Acetylglucosaminyltransferase 2 (Beta 1,4-)	Protein Coding	37	GC03M043121	2.60763
NSUN3	NOP2/Sun RNA Methyltransferase 3	Protein Coding	37	GC03P094062	2.60419
GANC	Glucosidase Alpha, Neutral C	Protein Coding	37	GC15P042273	2.584931
CUTC	CutC Copper Transporter	Protein Coding	37	GC10P099702	2.578959
EGFLAM	EGF Like, Fibronectin Type III And Laminin G Domains	Protein Coding	37	GC05P038295	2.578254
EHD3	EH Domain Containing 3	Protein Coding	37	GC02P031234	2.575096

FADS3	Fatty Acid Desaturase 3	Protein Coding	37	GC11M061873	2.572872
PPFIA2	PTPRF Interacting Protein Alpha 2	Protein Coding	37	GC12M081257	2.564592
TBX10	T-Box Transcription Factor 10	Protein Coding	37	GC11M067631	2.55938
MPPED2	Metallophosphoesterase Domain Containing 2	Protein Coding	37	GC11M030406	2.554156
ATP9A	ATPase Phospholipid Transporting 9A (Putative)	Protein Coding	37	GC20M051596	2.538993
PLB1	Phospholipase B1	Protein Coding	37	GC02P028460	2.536083
TRMT10A	TRNA Methyltransferase 10A	Protein Coding	37	GC04M099546	2.535224
PPP1R3B	Protein Phosphatase 1 Regulatory Subunit 3B	Protein Coding	37	GC08M009136	2.530024
LRFN4	Leucine Rich Repeat And Fibronectin Type III Domain Containing 4	Protein Coding	37	GC11P066856	2.530024
CPAMD8	C3 And PZP Like Alpha-2-Macroglobulin Domain Containing 8	Protein Coding	37	GC19M016892	2.522286
CCP110	Centriolar Coiled-Coil Protein 110	Protein Coding	37	GC16P019536	2.520321
VARS1	Valyl-TRNA Synthetase 1	Protein Coding	37	GC06M046896	2.505378
SNX17	Sorting Nexin 17	Protein Coding	37	GC02P027370	2.498716
RAI14	Retinoic Acid Induced 14	Protein Coding	37	GC05P034656	2.492419
PPM1H	Protein Phosphatase, Mg2+/Mn2+ Dependent 1H	Protein Coding	37	GC12M062643	2.484498
AQR	Aquarius Intron-Binding Spliceosomal Factor	Protein Coding	37	GC15M034851	2.481889
MBOAT1	Membrane Bound O-Acyltransferase Domain Containing 1	Protein Coding	37	GC06M020102	2.475805
CFDP1	Craniofacial Development Protein 1	Protein Coding	37	GC16M075294	2.471191
STARD10	StAR Related Lipid Transfer Domain Containing 10	Protein Coding	37	GC11M072774	2.460157
FBXO31	F-Box Protein 31	Protein Coding	37	GC16M087326	2.444783
INTS4	Integrator Complex Subunit 4	Protein Coding	37	GC11M077878	2.443795
NRIP2	Nuclear Receptor Interacting Protein 2	Protein Coding	37	GC12M002824	2.431969
TENM3	Teneurin Transmembrane Protein 3	Protein Coding	37	GC04P181448	2.431275
RND2	Rho Family GTPase 2	Protein Coding	37	GC17P044670	2.429949
GTF3A	General Transcription Factor IIIA	Protein Coding	37	GC13P027427	2.428806
INTS8	Integrator Complex Subunit 8	Protein Coding	37	GC08P094813	2.410869
WDR73	WD Repeat Domain 73	Protein Coding	37	GC15M084639	2.410012
FNDC3B	Fibronectin Type III Domain Containing 3B	Protein Coding	37	GC03P172039	2.407704
SLC2A11	Solute Carrier Family 2 Member 11	Protein Coding	37	GC22P023856	2.40692
OVOL2	Ovo Like Zinc Finger 2	Protein Coding	37	GC20M017956	2.404111
ADGRA3	Adhesion G Protein-Coupled Receptor A3	Protein Coding	37	GC04M022345	2.40212
NUP37	Nucleoporin 37	Protein Coding	37	GC12M102073	2.400702
PLCXD3	Phosphatidylinositol Specific Phospholipase C X Domain Containing 3	Protein Coding	37	GC05M041306	2.389665
SASS6	SAS-6 Centriolar Assembly Protein	Protein Coding	37	GC01M100083	2.385521
MRPS27	Mitochondrial Ribosomal Protein S27	Protein Coding	37	GC05M072219	2.380829
SRGAP2	SLIT-ROBO Rho GTPase Activating Protein 2	Protein Coding	37	GC01P206203	2.365086
ENOX1	Ecto-NOX Disulfide-Thiol Exchanger 1	Protein Coding	37	GC13M043213	2.364772
L3MBTL3	L3MBTL Histone Methyl-Lysine Binding Protein 3	Protein Coding	37	GC06P130013	2.359062
PRPS1L1	Phosphoribosyl Pyrophosphate Synthetase 1 Like 1	Protein Coding	37	GC07M018026	2.358344
DEFB4A	Defensin Beta 4A	Protein Coding	37	GC08P007895	2.351131
APPBP2	Amyloid Beta Precursor Protein Binding Protein 2	Protein Coding	37	GC17M060443	2.334525
TAF8	TATA-Box Binding Protein Associated Factor 8	Protein Coding	37	GC06P042050	2.333429
CAPN8	Calpain 8	Protein Coding	37	GC01M223538	2.331818
BTBD9	BTB Domain Containing 9	Protein Coding	37	GC06M038168	2.309549
DUS1L	Dihydrouridine Synthase 1 Like	Protein Coding	37	GC17M082057	2.305705
TRUB1	TruB Pseudouridine Synthase Family Member 1	Protein Coding	37	GC10P114938	2.304406
WDR81	WD Repeat Domain 81	Protein Coding	37	GC17P001716	2.300623
HIVEP1	HIVEP Zinc Finger 1	Protein Coding	37	GC06P012009	2.297144
NAGPA	N-Acetylglucosamine-1-Phosphodiester Alpha-N-Acetylglucosaminidase	Protein Coding	37	GC16M005014	2.296788
ZIC4	Zic Family Member 4	Protein Coding	37	GC03M147386	2.284126
PPP1R1C	Protein Phosphatase 1 Regulatory Inhibitor Subunit 1C	Protein Coding	37	GC02P181954	2.275167
ZWINT	ZW10 Interacting Kinetochore Protein	Protein Coding	37	GC10M056357	2.25427
CAPN12	Calpain 12	Protein Coding	37	GC19M038730	2.246662

TMPRSS13	Transmembrane Serine Protease 13	Protein Coding	37	GC11M117900	2.240934
THAP5	THAP Domain Containing 5	Protein Coding	37	GC07M108554	2.237974
HORMAD1	HORMA Domain Containing 1	Protein Coding	37	GC01M150835	2.232543
PCDH18	Protocadherin 18	Protein Coding	37	GC04M137518	2.222903
PNPLA1	Patatin Like Phospholipase Domain Containing 1	Protein Coding	37	GC06P055343	2.216533
B3GNT7	UDP-GlcNAc:BetaGal Beta-1,3-N-Acetylglucosaminyltransferase 7	Protein Coding	37	GC02P231395	2.173859
TMED2	Transmembrane P24 Trafficking Protein 2	Protein Coding	37	GC12P123584	2.169025
SENP7	SUMO Specific Peptidase 7	Protein Coding	37	GC03M101324	2.155961
CDCA4	Cell Division Cycle Associated 4	Protein Coding	37	GC14M105009	2.146827
MBNL3	Muscleblind Like Splicing Regulator 3	Protein Coding	37	GC0XM132369	2.145676
VASH1	Vasohibin 1	Protein Coding	37	GC14P076761	2.144872
GINS2	GINS Complex Subunit 2	Protein Coding	37	GC16M085676	2.142493
PLEKHB1	Pleckstrin Homology Domain Containing B1	Protein Coding	37	GC11P073647	2.140986
PTPMT1	Protein Tyrosine Phosphatase Mitochondrial 1	Protein Coding	37	GC11P047568	2.14024
SKA2	Spindle And Kinetochore Associated Complex Subunit 2	Protein Coding	37	GC17M059109	2.134342
S100A13	S100 Calcium Binding Protein A13	Protein Coding	37	GC01M153618	2.128762
ASZ1	Ankyrin Repeat, SAM And Basic Leucine Zipper Domain Containing 1	Protein Coding	37	GC07M117363	2.125531
RARS1	Arginyl-TRNA Synthetase 1	Protein Coding	37	GC05P168487	2.116516
CNIH3	Cornichon Family AMPA Receptor Auxiliary Protein 3	Protein Coding	37	GC01P224435	2.116357
HS3ST3A1	Heparan Sulfate-Glucosamine 3-Sulfotransferase 3A1	Protein Coding	37	GC17M013494	2.105883
APOL3	Apolipoprotein L3	Protein Coding	37	GC22M036140	2.101099
ATG2A	Autophagy Related 2A	Protein Coding	37	GC11M069349	2.092396
APOL4	Apolipoprotein L4	Protein Coding	37	GC22M036190	2.089134
EBF2	EBF Transcription Factor 2	Protein Coding	37	GC08M025841	2.086679
ZGPAT	Zinc Finger CCCH-Type And G-Patch Domain Containing	Protein Coding	37	GC20P063707	2.083934
AHNAK	AHNAK Nucleoprotein	Protein Coding	37	GC11M069238	2.081163
AMELX	Amelogenin X-Linked	Protein Coding	37	GC0XP011293	2.078025
BARX1	BARX Homeobox 1	Protein Coding	37	GC09M093951	2.076395
SYT9	Synaptotagmin 9	Protein Coding	37	GC11P007238	2.075945
MTMR12	Myotubularin Related Protein 12	Protein Coding	37	GC05M032227	2.062305
TAS1R2	Taste 1 Receptor Member 2	Protein Coding	37	GC01M018839	2.056725
VSIG4	V-Set And Immunoglobulin Domain Containing 4	Protein Coding	37	GC0XM066021	2.048203
TAS2R1	Taste 2 Receptor Member 1	Protein Coding	37	GC05M009630	2.047443
SNRNP40	Small Nuclear Ribonucleoprotein U5 Subunit 40	Protein Coding	37	GC01M031259	2.046014
ZNF77	Zinc Finger Protein 77	Protein Coding	37	GC19M002933	2.042707
TCFL5	Transcription Factor Like 5	Protein Coding	37	GC20M062841	2.041928
TAF1A	TATA-Box Binding Protein Associated Factor, RNA Polymerase I Subunit A	Protein Coding	37	GC01M222557	2.039574
TRAT1	T Cell Receptor Associated Transmembrane Adaptor 1	Protein Coding	37	GC03P108823	2.02569
HELQ	Helicase, POLQ Like	Protein Coding	37	GC04M083407	2.024795
UXT	Ubiquitously Expressed Prefoldin Like Chaperone	Protein Coding	37	GC0XM047651	2.012905
SCOC	Short Coiled-Coil Protein	Protein Coding	37	GC04P140257	2.003818
FTSJ3	FtsJ RNA 2'-O-Methyltransferase 3	Protein Coding	37	GC17M063819	1.997971
SNX6	Sorting Nexin 6	Protein Coding	37	GC14M034561	1.996382
MNT	MAX Network Transcriptional Repressor	Protein Coding	37	GC17M002384	1.993197
ZNF513	Zinc Finger Protein 513	Protein Coding	37	GC02M027377	1.985161
ACER2	Alkaline Ceramidase 2	Protein Coding	37	GC09P019408	1.982394
MTUS1	Microtubule Associated Scaffold Protein 1	Protein Coding	37	GC08M017643	1.973903
MND1	Meiotic Nuclear Divisions 1	Protein Coding	37	GC04P153344	1.973567
WDR45B	WD Repeat Domain 45B	Protein Coding	37	GC17M082614	1.964821
SLC41A2	Solute Carrier Family 41 Member 2	Protein Coding	37	GC12M104802	1.960734
SPATA7	Spermatogenesis Associated 7	Protein Coding	37	GC14P088384	1.96054
HSCB	HscB Mitochondrial Iron-Sulfur Cluster Cochaperone	Protein Coding	37	GC22P028747	1.958953
KNSTRN	Kinetochore Localized Astrin (SPAG5) Binding Protein	Protein Coding	37	GC15P040382	1.954746

DMRTA1	DMRT Like Family A1	Protein Coding	37	GC09P022436	1.946029
ZRANB3	Zinc Finger RANBP2-Type Containing 3	Protein Coding	37	GC02M135136	1.944059
MLEC	Malectin	Protein Coding	37	GC12P120687	1.943704
WDR59	WD Repeat Domain 59	Protein Coding	37	GC16M074871	1.942758
DOCK11	Dedicator Of Cytokinesis 11	Protein Coding	37	GC0XP118496	1.939499
RBM14	RNA Binding Motif Protein 14	Protein Coding	37	GC11P066785	1.932078
ADAMTSL5	ADAMTS Like 5	Protein Coding	37	GC19M002139	1.919236
STYX	Serine/Threonine/Tyrosine Interacting Protein	Protein Coding	37	GC14P052730	1.913979
KRT25	Keratin 25	Protein Coding	37	GC17M040748	1.901998
RBP5	Retinol Binding Protein 5	Protein Coding	37	GC12M007194	1.901452
CLEC16A	C-Type Lectin Domain Containing 16A	Protein Coding	37	GC16P010944	1.901381
ENTPD4	Ectonucleoside Triphosphate Diphosphohydrolase 4	Protein Coding	37	GC08M023385	1.900681
SPARCL1	SPARC Like 1	Protein Coding	37	GC04M087473	1.898904
LIPN	Lipase Family Member N	Protein Coding	37	GC10P088760	1.894174
PRCC	Proline Rich Mitotic Checkpoint Control Factor	Protein Coding	37	GC01P156750	1.891959
COPS8	COP9 Signalosome Subunit 8	Protein Coding	37	GC02P237085	1.891506
LYZL1	Lysozyme Like 1	Protein Coding	37	GC10P029297	1.890326
DMTN	Dematin Actin Binding Protein	Protein Coding	37	GC08P022048	1.884934
NIPA2	NIPA Magnesium Transporter 2	Protein Coding	37	GC15P027247	1.884919
GOLGB1	Golgin B1	Protein Coding	37	GC03M121663	1.882595
FOXD1	Forkhead Box D1	Protein Coding	37	GC05M073444	1.876955
ETV7	ETS Variant Transcription Factor 7	Protein Coding	37	GC06M047035	1.876668
ERLEC1	Endoplasmic Reticulum Lectin 1	Protein Coding	37	GC02P053786	1.85556
HECTD2	HECT Domain E3 Ubiquitin Protein Ligase 2	Protein Coding	37	GC10P091409	1.852835
ZNF597	Zinc Finger Protein 597	Protein Coding	37	GC16M003782	1.844087
TSPYL2	TSPY Like 2	Protein Coding	37	GC0XP053082	1.843389
BRPF3	Bromodomain And PHD Finger Containing 3	Protein Coding	37	GC06P055341	1.837246
DPY30	Dpy-30 Histone Methyltransferase Complex Regulatory Subunit	Protein Coding	37	GC02M031867	1.835194
FNIP1	Folliculin Interacting Protein 1	Protein Coding	37	GC05M131641	1.833463
CLEC4E	C-Type Lectin Domain Family 4 Member E	Protein Coding	37	GC12M008535	1.833163
SETD1B	SET Domain Containing 1B, Histone Lysine Methyltransferase	Protein Coding	37	GC12P123586	1.832527
ECM2	Extracellular Matrix Protein 2	Protein Coding	37	GC09M092493	1.830746
DENND3	DENN Domain Containing 3	Protein Coding	37	GC08P141117	1.830589
PSPC1	Paraspeckle Component 1	Protein Coding	37	GC13M019674	1.828933
DNAJC15	DnaJ Heat Shock Protein Family (Hsp40) Member C15	Protein Coding	37	GC13P043023	1.828206
RLN3	Relaxin 3	Protein Coding	37	GC19P014038	1.82779
WDR82	WD Repeat Domain 82	Protein Coding	37	GC03M052254	1.825142
IL37	Interleukin 37	Protein Coding	37	GC02P116690	1.824267
ZC3H12A	Zinc Finger CCCH-Type Containing 12A	Protein Coding	37	GC01P037474	1.823758
CEP70	Centrosomal Protein 70	Protein Coding	37	GC03M138494	1.821882
ARHGAP28	Rho GTPase Activating Protein 28	Protein Coding	37	GC18P006730	1.819437
FAM83H	Family With Sequence Similarity 83 Member H	Protein Coding	37	GC08M143723	1.816323
TCF19	Transcription Factor 19	Protein Coding	37	GC06P055190	1.807692
PLXDC2	Plexin Domain Containing 2	Protein Coding	37	GC10P019769	1.801404
HMBOX1	Homeobox Containing 1	Protein Coding	37	GC08P028890	1.792357
PLA2G15	Phospholipase A2 Group XV	Protein Coding	37	GC16P068245	1.787323
LECT2	Leukocyte Cell Derived Chemotaxin 2	Protein Coding	37	GC05M135922	1.783898
CRISPLD2	Cysteine Rich Secretory Protein LCCL Domain Containing 2	Protein Coding	37	GC16P084819	1.779552
UBAC1	UBA Domain Containing 1	Protein Coding	37	GC09M135932	1.779484
ZNF318	Zinc Finger Protein 318	Protein Coding	37	GC06M047133	1.775566
SPAG6	Sperm Associated Antigen 6	Protein Coding	37	GC10P022345	1.772429
HP1BP3	Heterochromatin Protein 1 Binding Protein 3	Protein Coding	37	GC01M020742	1.771282
SMC5	Structural Maintenance Of Chromosomes 5	Protein Coding	37	GC09P070258	1.756742

HIRIP3	HIRA Interacting Protein 3	Protein Coding	37	GC16M029992	1.755762
MANEA	Mannosidase Endo-Alpha	Protein Coding	37	GC06P095577	1.755049
PYROXD1	Pyridine Nucleotide-Disulphide Oxidoreductase Domain 1	Protein Coding	37	GC12P021437	1.751352
SNRPD3	Small Nuclear Ribonucleoprotein D3 Polypeptide	Protein Coding	37	GC22P024555	1.749698
ARID4B	AT-Rich Interaction Domain 4B	Protein Coding	37	GC01M235133	1.74709
RILP	Rab Interacting Lysosomal Protein	Protein Coding	37	GC17M001646	1.738933
UST	Uronyl 2-Sulfotransferase	Protein Coding	37	GC06P148721	1.731143
MKX	Mohawk Homeobox	Protein Coding	37	GC10M027682	1.728532
NAA30	N-Alpha-Acetyltransferase 30, NatC Catalytic Subunit	Protein Coding	37	GC14P057390	1.71975
SSX2IP	SSX Family Member 2 Interacting Protein	Protein Coding	37	GC01M084643	1.714728
GALNT16	Polypeptide N-Acetylgalactosaminyltransferase 16	Protein Coding	37	GC14P069259	1.710231
CHSY3	Chondroitin Sulfate Synthase 3	Protein Coding	37	GC05P129904	1.709934
KIR2DL1	Killer Cell Immunoglobulin Like Receptor, Two Ig Domains And Long Cytoplasmic Tail 1	Protein Coding	37	GC19P056443	1.700325
CDC42EP2	CDC42 Effector Protein 2	Protein Coding	37	GC11P065332	1.699562
SLC37A1	Solute Carrier Family 37 Member 1	Protein Coding	37	GC21P042531	1.69473
SNX4	Sorting Nexin 4	Protein Coding	37	GC03M125446	1.69204
NDFIP2	Nedd4 Family Interacting Protein 2	Protein Coding	37	GC13P079481	1.690301
YTHDF1	YTH N6-Methyladenosine RNA Binding Protein 1	Protein Coding	37	GC20M063195	1.680471
LYPD3	LY6/PLAUR Domain Containing 3	Protein Coding	37	GC19M043460	1.679876
PIF1	PIF1 5'-To-3' DNA Helicase	Protein Coding	37	GC15M064815	1.678489
CPEB4	Cytoplasmic Polyadenylation Element Binding Protein 4	Protein Coding	37	GC05P173888	1.676754
TMEM132D	Transmembrane Protein 132D	Protein Coding	37	GC12M129071	1.672584
THOC7	THO Complex 7	Protein Coding	37	GC03M063833	1.670861
SPCS3	Signal Peptidase Complex Subunit 3	Protein Coding	37	GC04P176319	1.662521
MEI1	Meiotic Double-Stranded Break Formation Protein 1	Protein Coding	37	GC22P041699	1.657642
RAB21	RAB21, Member RAS Oncogene Family	Protein Coding	37	GC12P071754	1.656084
MRPL43	Mitochondrial Ribosomal Protein L43	Protein Coding	37	GC10M100969	1.653761
WDR83	WD Repeat Domain 83	Protein Coding	37	GC19P012666	1.64821
UACA	Uveal Autoantigen With Coiled-Coil Domains And Ankyrin Repeats	Protein Coding	37	GC15M070654	1.645795
LPCAT1	Lysophosphatidylcholine Acyltransferase 1	Protein Coding	37	GC05M001456	1.625302
IGDCC3	Immunoglobulin Superfamily DCC Subclass Member 3	Protein Coding	37	GC15M065327	1.622865
RNF11	Ring Finger Protein 11	Protein Coding	37	GC01P051236	1.619308
RALGPS1	Ral GEF With PH Domain And SH3 Binding Motif 1	Protein Coding	37	GC09P126914	1.619263
AVEN	Apoptosis And Caspase Activation Inhibitor	Protein Coding	37	GC15M033853	1.619222
EAPP	E2F Associated Phosphoprotein	Protein Coding	37	GC14M034516	1.618658
TNRC6B	Trinucleotide Repeat Containing Adaptor 6B	Protein Coding	37	GC22P040044	1.613678
CSHL1	Chorionic Somatomammotropin Hormone Like 1	Protein Coding	37	GC17M063909	1.611853
NEURL1	Neuralized E3 Ubiquitin Protein Ligase 1	Protein Coding	37	GC10P103493	1.61118
KCTD3	Potassium Channel Tetramerization Domain Containing 3	Protein Coding	37	GC01P215567	1.608166
GNL2	G Protein Nucleolar 2	Protein Coding	37	GC01M037566	1.602343
FAT2	FAT Atypical Cadherin 2	Protein Coding	37	GC05M151504	1.602045
HLA-DQB2	Major Histocompatibility Complex, Class II, DQ Beta 2	Protein Coding	37	GC06M032756	1.59709
NT5M	5',3'-Nucleotidase, Mitochondrial	Protein Coding	37	GC17P017303	1.595949
ISLR	Immunoglobulin Superfamily Containing Leucine Rich Repeat	Protein Coding	37	GC15P074173	1.593922
SCARA3	Scavenger Receptor Class A Member 3	Protein Coding	37	GC08P027633	1.59246
ZNF592	Zinc Finger Protein 592	Protein Coding	37	GC15P085360	1.591021
NKX3-2	NK3 Homeobox 2	Protein Coding	37	GC04M013542	1.589777
ATP8B4	ATPase Phospholipid Transporting 8B4 (Putative)	Protein Coding	37	GC15M049858	1.588688
TBC1D5	TBC1 Domain Family Member 5	Protein Coding	37	GC03M017157	1.583051
SNX13	Sorting Nexin 13	Protein Coding	37	GC07M017798	1.57628
ATPAF1	ATP Synthase Mitochondrial F1 Complex Assembly Factor 1	Protein Coding	37	GC01M046632	1.572468
SCUBE1	Signal Peptide, CUB Domain And EGF Like Domain Containing 1	Protein Coding	37	GC22M043197	1.56843
VEPH1	Ventricular Zone Expressed PH Domain Containing 1	Protein Coding	37	GC03M157259	1.56775

CCL24	C-C Motif Chemokine Ligand 24	Protein Coding	37	GC07M075815	1.56484
AGBL2	AGBL Carboxypeptidase 2	Protein Coding	37	GC11M069008	1.559746
RABGAP1	RAB GTPase Activating Protein 1	Protein Coding	37	GC09P122932	1.550257
GJA9	Gap Junction Protein Alpha 9	Protein Coding	37	GC01M038874	1.549404
PNMA2	PNMA Family Member 2	Protein Coding	37	GC08M026504	1.54898
TIMM22	Translocase Of Inner Mitochondrial Membrane 22	Protein Coding	37	GC17P000997	1.54897
ENC1	Ectodermal-Neural Cortex 1	Protein Coding	37	GC05M074627	1.548955
SGMS2	Sphingomyelin Synthase 2	Protein Coding	37	GC04P107824	1.548548
GIPC2	GIPC PDZ Domain Containing Family Member 2	Protein Coding	37	GC01P077987	1.542108
ATP10B	ATPase Phospholipid Transporting 10B (Putative)	Protein Coding	37	GC05M160499	1.538703
PLXDC1	Plexin Domain Containing 1	Protein Coding	37	GC17M039063	1.525165
SLC15A3	Solute Carrier Family 15 Member 3	Protein Coding	37	GC11M069181	1.523404
SCPEP1	Serine Carboxypeptidase 1	Protein Coding	37	GC17P056978	1.513433
COL20A1	Collagen Type XX Alpha 1 Chain	Protein Coding	37	GC20P063293	1.510853
COLGALT1	Collagen Beta(1-O)Galactosyltransferase 1	Protein Coding	37	GC19P017555	1.508884
TSPAN3	Tetraspanin 3	Protein Coding	37	GC15M077042	1.507367
RBMS2	RNA Binding Motif Single Stranded Interacting Protein 2	Protein Coding	37	GC12P056619	1.503564
ARSD	Arylsulfatase D	Protein Coding	37	GC0XM002903	1.501168
TEKT2	Tektin 2	Protein Coding	37	GC01P036084	1.500346
ATP6V0E2	ATPase H+ Transporting V0 Subunit E2	Protein Coding	37	GC07P149873	1.498723
CENPP	Centromere Protein P	Protein Coding	37	GC09P092325	1.49599
GAL3ST4	Galactose-3-O-Sulfotransferase 4	Protein Coding	37	GC07M100159	1.494705
KLHDC2	Kelch Domain Containing 2	Protein Coding	37	GC14P049767	1.493211
OMP	Olfactory Marker Protein	Protein Coding	37	GC11P077102	1.491036
SLITRK5	SLIT And NTRK Like Family Member 5	Protein Coding	37	GC13P087671	1.483882
NASP	Nuclear Autoantigenic Sperm Protein	Protein Coding	37	GC01P045583	1.48355
MON1A	MON1 Homolog A, Secretory Trafficking Associated	Protein Coding	37	GC03M050082	1.474198
PODN	Podocan	Protein Coding	37	GC01P053062	1.47359
WRNIP1	WRN Helicase Interacting Protein 1	Protein Coding	37	GC06P002766	1.471641
DPH5	Diphthamide Biosynthesis 5	Protein Coding	37	GC01M100989	1.465684
PLP2	Proteolipid Protein 2	Protein Coding	37	GC0XP049171	1.462448
SAMD12	Sterile Alpha Motif Domain Containing 12	Protein Coding	37	GC08M118131	1.462382
NXPH4	Neurexophilin 4	Protein Coding	37	GC12P057218	1.451353
MRPL10	Mitochondrial Ribosomal Protein L10	Protein Coding	37	GC17M047824	1.44723
UBXN11	UBX Domain Protein 11	Protein Coding	37	GC01M026281	1.447044
AKAP3	A-Kinase Anchoring Protein 3	Protein Coding	37	GC12M004614	1.444338
HUNK	Hormonally Up-Regulated Neu-Associated Kinase	Protein Coding	37	GC21P031873	1.443532
METAP1D	Methionyl Aminopeptidase Type 1D, Mitochondrial	Protein Coding	37	GC02P172000	1.443224
TBC1D9	TBC1 Domain Family Member 9	Protein Coding	37	GC04M140621	1.440594
NEMF	Nuclear Export Mediator Factor	Protein Coding	37	GC14M049782	1.439471
PPFIBP2	PPFIA Binding Protein 2	Protein Coding	37	GC11P007491	1.438455
CHPF2	Chondroitin Polymerizing Factor 2	Protein Coding	37	GC07P151232	1.438131
ARHGAP18	Rho GTPase Activating Protein 18	Protein Coding	37	GC06M129576	1.426045
GLB1L3	Galactosidase Beta 1 Like 3	Protein Coding	37	GC11P134269	1.425825
UPK2	Uroplakin 2	Protein Coding	37	GC11P118925	1.424726
NPDC1	Neural Proliferation, Differentiation And Control 1	Protein Coding	37	GC09M137039	1.423456
NCOA5	Nuclear Receptor Coactivator 5	Protein Coding	37	GC20M046060	1.421263
TNFSF8	TNF Superfamily Member 8	Protein Coding	37	GC09M114893	1.42065
SEC16A	SEC16 Homolog A, Endoplasmic Reticulum Export Factor	Protein Coding	37	GC09M136440	1.418905
ATRN	Attractin	Protein Coding	37	GC20P003471	1.416347
FBXW2	F-Box And WD Repeat Domain Containing 2	Protein Coding	37	GC09M120751	1.405096
ESRP2	Epithelial Splicing Regulatory Protein 2	Protein Coding	37	GC16M068229	1.393461
SRP68	Signal Recognition Particle 68	Protein Coding	37	GC17M076038	1.391349

ABHD10	Abhydrolase Domain Containing 10, Depalmitoylase	Protein Coding	37	GC03P111978	1.387608
VTA1	Vesicle Trafficking 1	Protein Coding	37	GC06P142147	1.374471
CSNK1A1L	Casein Kinase 1 Alpha 1 Like	Protein Coding	37	GC13M037103	1.372919
FUT4	Fucosyltransferase 4	Protein Coding	37	GC11P094544	1.372788
ST7	Suppression Of Tumorigenicity 7	Protein Coding	37	GC07P117037	1.365533
ZNF408	Zinc Finger Protein 408	Protein Coding	37	GC11P046700	1.363019
TMBIM6	Transmembrane BAX Inhibitor Motif Containing 6	Protein Coding	37	GC12P049707	1.35704
KLHL22	Kelch Like Family Member 22	Protein Coding	37	GC22M020442	1.352476
TFCP2L1	Transcription Factor CP2 Like 1	Protein Coding	37	GC02M121216	1.344754
AAMP	Angio Associated Migratory Cell Protein	Protein Coding	37	GC02M218264	1.340949
DLGAP3	DLG Associated Protein 3	Protein Coding	37	GC01M034865	1.33882
FRMPD4	FERM And PDZ Domain Containing 4	Protein Coding	37	GC0XP011822	1.326522
ZC3H11A	Zinc Finger CCCH-Type Containing 11A	Protein Coding	37	GC01P203795	1.325282
RNF146	Ring Finger Protein 146	Protein Coding	37	GC06P127266	1.325044
FBXO25	F-Box Protein 25	Protein Coding	37	GC08P000406	1.323298
MED28	Mediator Complex Subunit 28	Protein Coding	37	GC04P017616	1.322444
CBX6	Chromobox 6	Protein Coding	37	GC22M038861	1.322188
DHRS11	Dehydrogenase/Reductase 11	Protein Coding	37	GC17P036888	1.31918
PIGU	Phosphatidylinositol Glycan Anchor Biosynthesis Class U	Protein Coding	37	GC20M034560	1.318271
PNO1	Partner Of NOB1 Homolog	Protein Coding	37	GC02P068157	1.316725
NPY4R	Neuropeptide Y Receptor Y4	Protein Coding	37	GC10M046461	1.316635
MRM1	Mitochondrial RRNA Methyltransferase 1	Protein Coding	37	GC17P036892	1.316447
MATN4	Matrilin 4	Protein Coding	37	GC20M045293	1.315854
SLC22A17	Solute Carrier Family 22 Member 17	Protein Coding	37	GC14M023346	1.308296
BCAS3	BCAS3 Microtubule Associated Cell Migration Factor	Protein Coding	37	GC17P060678	1.301883
SNX11	Sorting Nexin 11	Protein Coding	37	GC17P048103	1.299386
MGA	MAX Dimerization Protein MGA	Protein Coding	37	GC15P041621	1.295425
TMEM230	Transmembrane Protein 230	Protein Coding	37	GC20M005064	1.291128
FAM126A	Family With Sequence Similarity 126 Member A	Protein Coding	37	GC07M022889	1.290146
CCDC8	Coiled-Coil Domain Containing 8	Protein Coding	37	GC19M046410	1.289202
UPK1B	Uroplakin 1B	Protein Coding	37	GC03P119173	1.287894
PKNOX2	PBX/Knotted 1 Homeobox 2	Protein Coding	37	GC11P125164	1.285806
MXD4	MAX Dimerization Protein 4	Protein Coding	37	GC04M002338	1.283047
ZBTB33	Zinc Finger And BTB Domain Containing 33	Protein Coding	37	GC0XP120250	1.278906
CAPNS2	Calpain Small Subunit 2	Protein Coding	37	GC16P055566	1.275327
DOLPP1	Dolichyldiphosphatase 1	Protein Coding	37	GC09P129081	1.274974
PCDHB4	Protocadherin Beta 4	Protein Coding	37	GC05P143796	1.274773
UBXN4	UBX Domain Protein 4	Protein Coding	37	GC02P135741	1.266914
DNAJC9	DnaJ Heat Shock Protein Family (Hsp40) Member C9	Protein Coding	37	GC10M073183	1.25949
AP5Z1	Adaptor Related Protein Complex 5 Subunit Zeta 1	Protein Coding	37	GC07P004775	1.256671
UBR7	Ubiquitin Protein Ligase E3 Component N-Recognin 7	Protein Coding	37	GC14P093207	1.256454
CPPED1	Calcineurin Like Phosphoesterase Domain Containing 1	Protein Coding	37	GC16M012659	1.250324
NAA20	N-Alpha-Acetyltransferase 20, NatB Catalytic Subunit	Protein Coding	37	GC20P020018	1.249786
HEPHL1	Hephaestin Like 1	Protein Coding	37	GC11P094021	1.246186
ARHGAP44	Rho GTPase Activating Protein 44	Protein Coding	37	GC17P012789	1.243256
SUGT1	SGT1 Homolog, MIS12 Kinetochore Complex Assembly Cochaperone	Protein Coding	37	GC13P052652	1.239112
MAMDC2	MAM Domain Containing 2	Protein Coding	37	GC09P070043	1.238291
MRPL18	Mitochondrial Ribosomal Protein L18	Protein Coding	37	GC06P159789	1.238025
SURF2	Surfeit 2	Protein Coding	37	GC09P133590	1.236411
BASP1	Brain Abundant Membrane Attached Signal Protein 1	Protein Coding	37	GC05P017065	1.234601
DUSP12	Dual Specificity Phosphatase 12	Protein Coding	37	GC01P161749	1.234312
RTCA	RNA 3'-Terminal Phosphate Cyclase	Protein Coding	37	GC01P100266	1.231286
KBTBD8	Kelch Repeat And BTB Domain Containing 8	Protein Coding	37	GC03P066998	1.227895

RANBP10	RAN Binding Protein 10	Protein Coding	37	GC16M067723	1.225201
TNIP3	TNFAIP3 Interacting Protein 3	Protein Coding	37	GC04M121131	1.222737
CITED1	Cbp/P300 Interacting Transactivator With Glu/Asp Rich Carboxy-Terminal Domain 1	Protein Coding	37	GC0XM072301	1.220404
TRIM26	Tripartite Motif Containing 26	Protein Coding	37	GC06M030184	1.219795
SMU1	SMU1 DNA Replication Regulator And Spliceosomal Factor	Protein Coding	37	GC09M033041	1.219045
DCD	Dermcidin	Protein Coding	37	GC12M054644	1.218994
CYP20A1	Cytochrome P450 Family 20 Subfamily A Member 1	Protein Coding	37	GC02P203238	1.207448
PDCD2	Programmed Cell Death 2	Protein Coding	37	GC06M170589	1.206902
ZFR	Zinc Finger RNA Binding Protein	Protein Coding	37	GC05M032390	1.205732
ARHGAP11A	Rho GTPase Activating Protein 11A	Protein Coding	37	GC15P032615	1.195604
ANKRD49	Ankyrin Repeat Domain 49	Protein Coding	37	GC11P094493	1.189669
IL17C	Interleukin 17C	Protein Coding	37	GC16P088638	1.184252
RHBDL2	Rhomboid Like 2	Protein Coding	37	GC01M038885	1.17353
NHS	NHS Actin Remodeling Regulator	Protein Coding	37	GC0XP017393	1.173404
PLEKHA8	Pleckstrin Homology Domain Containing A8	Protein Coding	37	GC07P030027	1.172321
IMMP1L	Inner Mitochondrial Membrane Peptidase Subunit 1	Protein Coding	37	GC11M031432	1.167514
NKX2-8	NK2 Homeobox 8	Protein Coding	37	GC14M036580	1.165671
LNX2	Ligand Of Numb-Protein X 2	Protein Coding	37	GC13M027545	1.162913
MAGEA3	MAGE Family Member A3	Protein Coding	37	GC0XP152698	1.162134
NEDD1	NEDD1 Gamma-Tubulin Ring Complex Targeting Factor	Protein Coding	37	GC12P096907	1.161059
ABHD11	Abhydrolase Domain Containing 11	Protein Coding	37	GC07M073736	1.158555
PPFIA1	PTPRF Interacting Protein Alpha 1	Protein Coding	37	GC11P070270	1.158157
GEMIN5	Gem Nuclear Organelle Associated Protein 5	Protein Coding	37	GC05M154887	1.158085
AP1G2	Adaptor Related Protein Complex 1 Subunit Gamma 2	Protein Coding	37	GC14M023559	1.155474
SNAPC4	Small Nuclear RNA Activating Complex Polypeptide 4	Protein Coding	37	GC09M136375	1.155066
SLC46A3	Solute Carrier Family 46 Member 3	Protein Coding	37	GC13M028700	1.149463
PEAR1	Platelet Endothelial Aggregation Receptor 1	Protein Coding	37	GC01P156893	1.149385
SAFB2	Scaffold Attachment Factor B2	Protein Coding	37	GC19M005587	1.148959
TOR2A	Torsin Family 2 Member A	Protein Coding	37	GC09M127745	1.148097
CKLF	Chemokine Like Factor	Protein Coding	37	GC16P066552	1.143353
CCAR2	Cell Cycle And Apoptosis Regulator 2	Protein Coding	37	GC08P022604	1.139653
GSDMB	Gasdermin B	Protein Coding	37	GC17M039904	1.138119
AQP10	Aquaporin 10	Protein Coding	37	GC01P154321	1.12711
POLR3GL	RNA Polymerase III Subunit GL	Protein Coding	37	GC01P145959	1.120378
TNFSF9	TNF Superfamily Member 9	Protein Coding	37	GC19P006531	1.119401
CERS5	Ceramide Synthase 5	Protein Coding	37	GC12M050129	1.118772
